# Directing the Stereoselectivity of the Claisen Rearrangement to Form Cyclic Ketones with Full Substitution at the α-Positions

**DOI:** 10.1021/acs.orglett.3c02752

**Published:** 2023-10-13

**Authors:** Fatimat O. Badmus, Raju S. Thombal, Satish Chandra Philkhana, Joshua A. Malone, Christian E. Bailey, Estefania Armendariz-Gonzalez, Edward W. Mureka, Cale M. Locicero, Frank R. Fronczek, Rendy Kartika

**Affiliations:** Department of Chemistry, Louisiana State University, 232 Choppin Hall, Baton Rouge, Louisiana 70803, United States

## Abstract

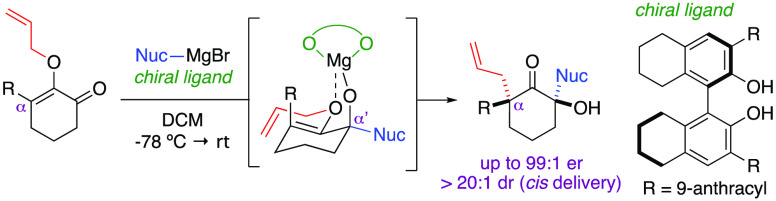

We report an enantioselective synthesis of cyclic ketones with full substitutions at the α-positions in a highly diastereoselective manner. Our method is achieved by subjecting substrate motifs in 2-allyloxyenones to chiral organomagnesium reagents, which trigger the Claisen rearrangement upon direct 1,2-carbonyl addition. The observed diastereoselectivity of the allyl migration is proposed to originate from the intramolecular chelation of the magnesium alkoxide to the allyloxy moiety.

The Claisen [3,3] sigmatropic rearrangement is a type of pericyclic reaction that involves the transposition of allyl vinyl ethers to furnish α-allyl carbonyl compounds. First reported in 1912,^1^ the Claisen rearrangement has emerged as a powerful method to construct carbon–carbon bonds,^2^ including sterically congested all-carbon quaternary centers.^3^ In regards to total synthesis of complex natural products,^4^ elegant applications of the Claisen rearrangement as a key step in the forging of quaternary centers are known.^5−9^ Such endeavors in polycyclic systems can be accomplished in a diastereoselective manner if the substrates readily adopt conformations that regulate the facial delivery of the allyl group.^5^ As exemplified in Scheme 1, Xu demonstrated that treatment of allyl vinyl ether **1b** in xylene at 175 °C under microwave irradiation furnished α-allyl diketone **1c** as a single diastereomer.^6^ Using *cis*-fused bicyclic precursor **2a**, Xie also showed a thermal-induced Claisen rearrangement that selectively formed stereoisomer **2b**.^7^ Nevertheless, the absence of an advantageous conformational bias in the allyl vinyl ether precursors could lead to an unproductive stereoinduction. For instance, Takemoto conveyed that the allyl group migration in *trans*-fused bicyclic starting material **3a** produced the corresponding ketones **3b** and **3c** as a mixture of diastereomers.^8^ Controlling the diastereoselectivity in simpler monocyclic systems is even more challenging. As illustrated in the work of Soós,^9^ thermal [3,3] rearrangement of precursor **4a** resulted in quaternary centers **4b** and **4c** as a 1:1 mixture of diastereomers despite the rich stereochemical motif within the structure of the substrate.

**Scheme 1:**
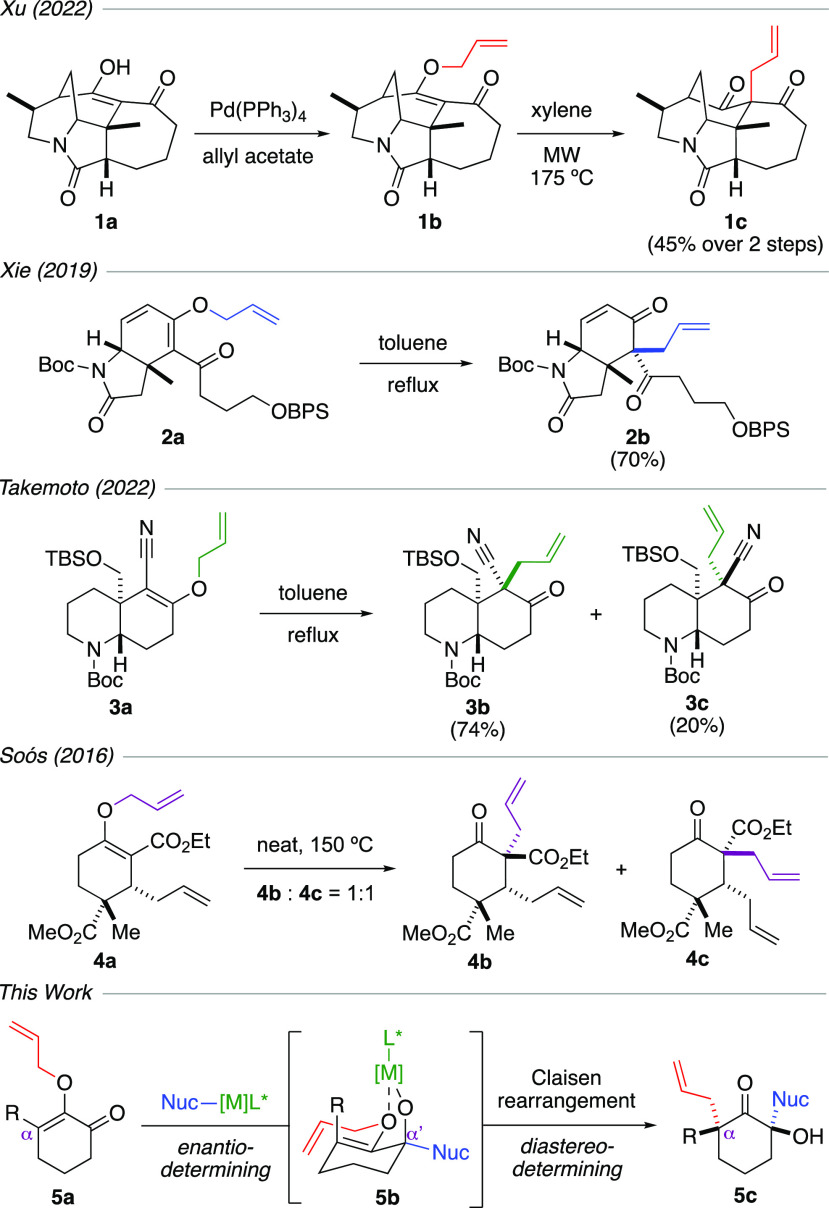
Governing Diastereoselectivity in the Formation of Quaternary Centers via the Claisen Rearrangement

These intriguing synthetic challenges inspired us to investigate new strategies to dictate the diastereoselectivity in the Claisen rearrangement. Specifically, we sought to establish chemistries that introduced transient conformational stability in simple cyclic allyl vinyl ethers, thereby enabling the Claisen rearrangement to proceed diastereoselectively en route to the formation of quaternary centers. As detailed in this Letter, we have developed a novel synthetic method to create two congested stereocenters at both α-positions of cyclic ketones, in which diastereocontrol in the α-allyl quaternary center formation via the Claisen rearrangement was assisted strategically by the opposing α-stereocenter. Our hypothesis is as follows: commencing with 2-allyloxyenone **5a** as a simple substrate motif, direct nucleophilic addition of an organometallic reagent to the carbonyl group should form intermediate **5b**, in which chelation of the emerging metal alkoxide to the allyloxy group would trigger the Claisen rearrangement to occur *in situ*.^10^ Moreover, this intramolecular interaction should provide the critical conformational stability to reactive intermediate **5b** to allow the allyl group to migrate in a diastereochemically defined manner, thereby furnishing ketone product **5c** with full substitution at the α-positions. In the presence of in chiral ligands, we envision that this method should offer an opportunity for enantioselective synthesis.

Our pilot experiments are depicted in Table 1. We began by subjecting substrate **6a** to a solution of methylmagnesium bromide in various reaction media. As shown in entry 1, the use of highly coordinating THF was not effective, as an inseparable mixture of products was generated. However, performing the reaction in Et_2_O or noncoordinating solvents, such as toluene and DCM, afforded tetrasubstituted α-quaternary ketone **7a** as a single diastereomer, in which both the methyl nucleophile and the allyl group were delivered with the relative *cis* configuration (entries 2–4). We chose DCM to proceed with the reaction optimization. Entries 4–6 revealed that the use of excess methylmagnesium bromide did not result in 1,2-carbonyl addition to the forming ketone product **7a**. While these reactions typically required over 20 h to reach completion at room temperature, the reaction time could be shortened considerably by warming the mixture to reflux. Entry 8 identified the crucial role of the magnesium metal in initiating the Claisen rearrangement and providing stereoinduction, as the use of methyl lithium generated a mixture of products.

**Table 1:**
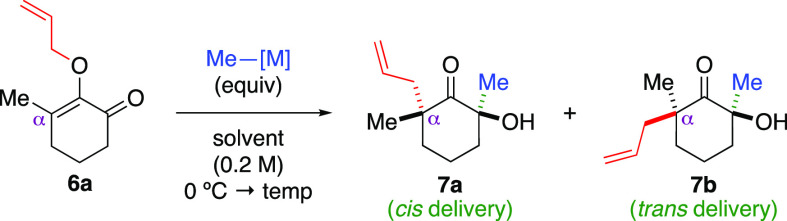
Proof of Concepts

entry	nuc	solvent	temperature	equiv	time (h)	yield (%)	**7a**/ **7b**^a^
1	MeMgBr	THF	rt	1.3	72	mix	
2	MeMgBr	ether	rt	1.3	23	77	>20:1
3	MeMgBr	toluene	rt	1.3	23	74	>20:1
4	MeMgBr	DCM	rt	1.3	23	82	>20:1
5	MeMgBr	DCM	rt	1.2	22	80	>20:1
6	MeMgBr	DCM	rt	1.1	22	78	>20:1
7	MeMgBr	DCM	reflux	1.3	5	76	>20:1
8	MeLi	toluene	reflux^b^	1.3	48	mix	

a The diastereomeric ratio was determined by ^1^H NMR of the crude reaction mixture.

b The Claisen rearrangement did not proceed at room temperature.

For operational simplicity, conditions in entry 4 were selected as we proceeded to evaluate the scope of reactions. Scheme 2 shows an extensive library of tetrasubstituted α-quaternary ketone products that were synthesized using our chemistry, including an example of a scaled-up synthesis. Our study commenced with subjecting substrate **6a** to a diverse selection of Grignard reagents. Unbranched organomagnesium halides in ethyl and octyl R groups furnished ketones **8a** and **8b** as single diastereomers in high yields. While a branched isobutyl Grignard reagent afforded product **8c** also as a single diastereomer, the cyclohexyl counterpart generated ketone **8d** in 72% yield with a 9:1 dr. The erosion in diastereoselectivity in this sterically congested example could have been caused by the much higher temperature that was needed for the Claisen rearrangement to occur, which was accommodated by performing the reaction in toluene. Other interesting results were noted when benzyl versus allyl groups were compared. While benzyl ketone **8e** was produced as a single diastereomer, the allyl counterpart **8f** surprisingly led to a 3:1 dr. As shown in ketones **8g**–**8i**, vinyl and propynyl nucleophiles were compatible. Evaluation of the use of aromatic Grignard reagents to form ketones **8j**–**8m** revealed that a reduced level of diastereoselectivity might occur. Furthermore, the Claisen rearrangement required an elevated temperature to reflux to expedite the allyl migration. The compatibility of five-membered substrates was examined using methyl and phenyl magnesium halides, which afforded ketones **8n** and **8o** in their respective 71% and 61% yields as single diastereomers.^11^

**Scheme 2:**
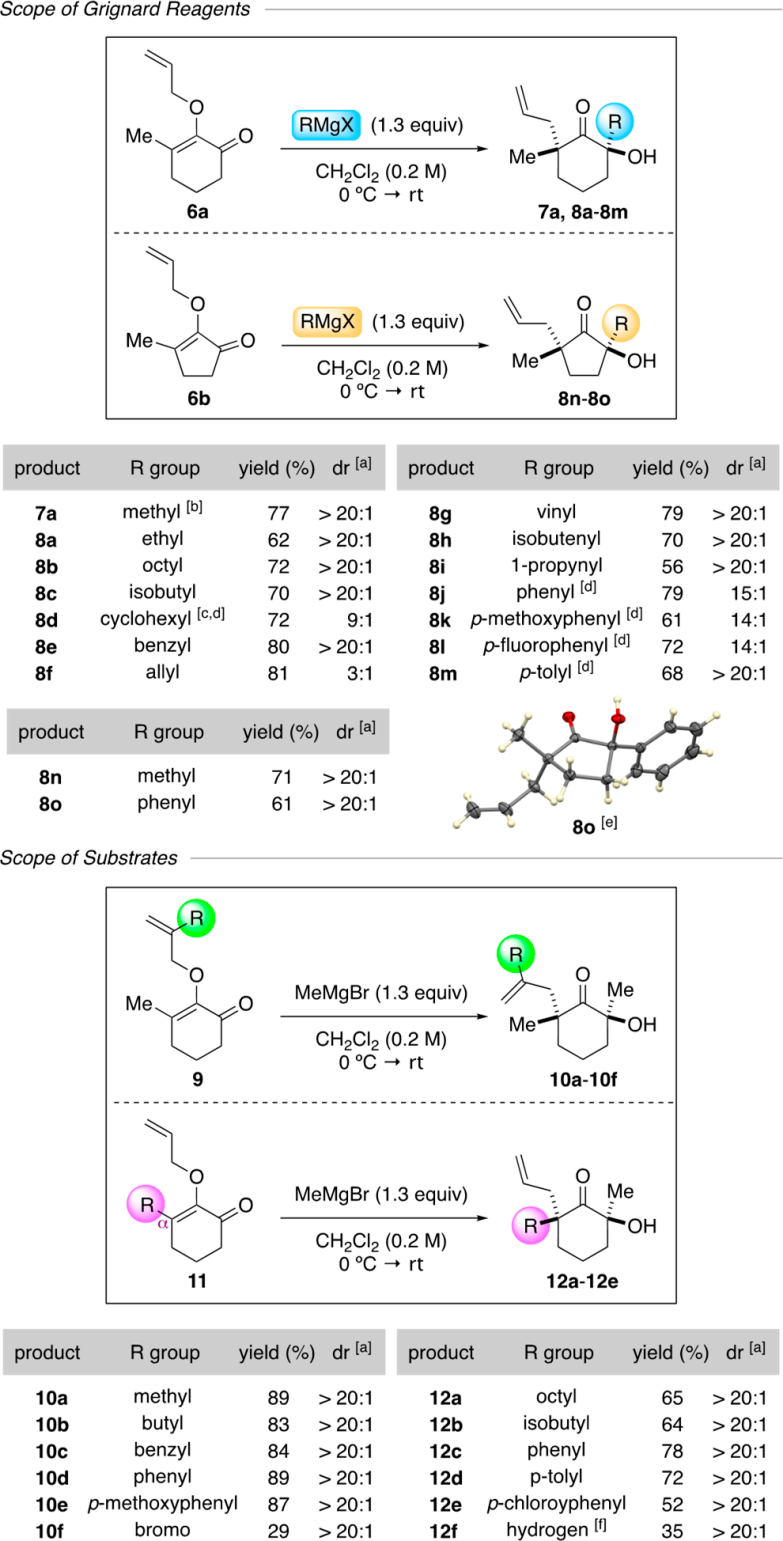
Scope of Reactions The diastereomeric ratio was determined by ^1^H NMR of the crude reaction mixture. The reaction was performed in a one-gram scale. The reaction was performed in toluene. The reaction mixture was warmed to reflux. The ellipsoid contour was set at a 50% probability level. 1.05 equiv of methylmagnesium bromide was employed.

We continued our studies by examining substituent effects at the C2 position of the allyloxy group in substrate **9** and at the α-position in substrate **11**. As showcased in products **10a**–**10c**, aliphatic groups, such as methyl and butyl and benzyl, at the C2 position were well tolerated. Incorporation of aromatic groups, such as phenyl and *p*-methoxyphenyl, formed ketones **10d** and **10e** in 89% and 87% yields, respectively, again as single diastereomers. Nonetheless, we encountered a reduced yield with the bromo variant **10f**, as the Claisen rearrangement step led to decomposition. Continuing with the α-substituent, we found both aliphatic and aromatic groups to be well tolerated. Ketone products **12a**–**12e**, which contained various substituted α-quaternary centers, i.e., octyl, isobutyl, phenyl, toluyl, and *p*-chlorophenyl, were produced in good yields as single diastereomers. The presence of a substituent at the α-position was crucial for the efficacy of the reaction, as treatment of the unsubstituted substrate with methylmagnesium bromide generated ketone **12f** in a 35% yield. In this case, we noted the formation of multiple byproducts as the Claisen rearrangement progressed. The relative configuration of the four substituents at the opposing α-positions in the ketone products were assigned by comparison based on the unambiguous structural elucidation of compounds **8e** and **8o** using X-ray crystallography.^12^

The development of an asymmetric variant of this method required control in the enantiodetermining Grignard addition to ketones, which could be realized by introducing chiral ligands to the reaction mixture.^13^ Inspired by the elegant work of Nakajima,^14^ we were drawn to 3,3′-substituted BINOL-derived ligands and identified that 9-anthracyl octahydro-BINOL ligand **14** provided the strongest enantioinduction.^14b^ The optimized protocol featured the combination of 1.4 equiv of ligand **14** and 3.9 equiv of Grignard reagents at −78 °C to create the chiral organomagnesium reagent *in situ* in the presence of 2-allyloxyenone substrates.^15^ Upon complete consumption of the starting material, warming the reaction mixture to room temperature prompted the Claisen rearrangement.

A representative scope of the enantioselective method is depicted in Scheme 3. We assessed various Grignard reagents. As shown in **(−)-7a**, **(−)-8a**, and **(−)-8b**, straight-chain aliphatic reagents furnished the ketone products in excellent enantiomeric ratios. However, the branched isobutyl in **(−)-8c** resulted in lowered enantioselectivity. In contrast to vinyl and propynyl nucleophiles that produced ketones **(−)-8g** and **(−)-8i** in 98:2 and 97:3 er, respectively, the aromatic series, such as phenyl **(−)-8j** and *p*-methoxyphenyl **(−)-8k**, surprisingly led to a considerable loss of enantioselectivity.^14c^ The five-membered substrate was tolerated to produce **(+)-8n** in a 79% yield with 98:2 er. Our next series focused on the scope of the allyloxy group at the C2 position. Substituents including aliphatic methyl and butyl, benzyl, and aromatic phenyl, and *p*-methoxy phenyl led to the production of ketones **(−)-10a** through **(+)-10e** in excellent yields and enantioselectivity. Similar to the racemic synthesis, bromo variant **(−)-10f** was isolated in a lower yield despite the high enantioselectivity at 95:5 er. Lastly, substituent effects at the α-carbon were evaluated, as exemplified in ketones **(−)-12a** to **(−)-12e**. These studies underscored that various linear and branched aliphatic and aromatic groups were tolerated to furnish the target products in good yields with high enantiomeric ratios. It is significant to note that the presence of chiral ligand **14** did not affect the diastereoselectivity outcome in the Claisen rearrangement. Except for a reduced diastereomeric ratio in compound **(−)-8k**, these tetrasubstituted ketone products were generated with >20:1 dr. The absolute and relative stereochemistry at the opposing α-positions were assigned by comparison based on the X-ray crystallography of *p*-nitrobenzoate ester derivatives **((−)-12c)-BzNO**_**2**_ and **((−)-12e)-BzNO**_**2**_.^12^

**Scheme 3:**
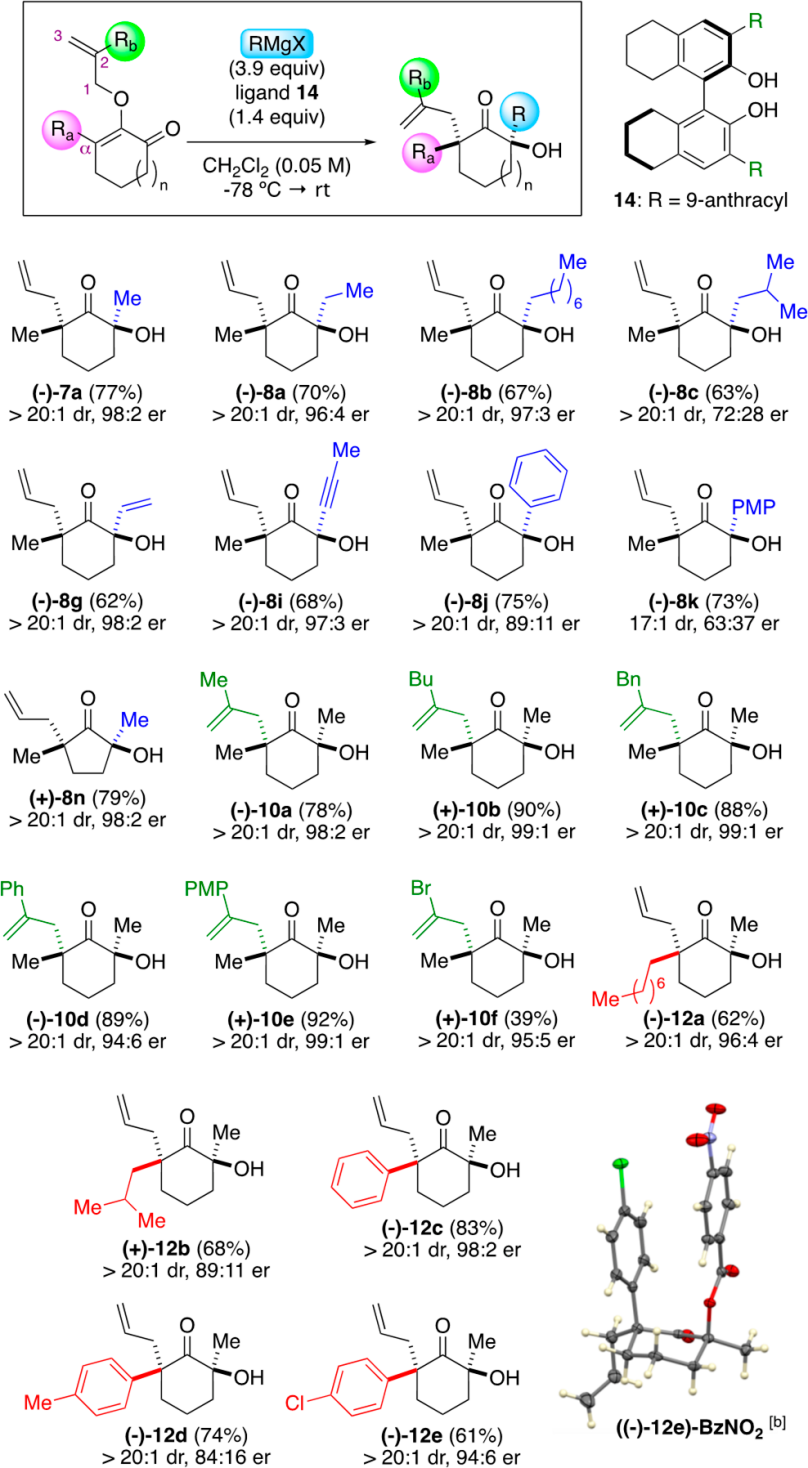
Enantioselective Method The diastereomeric ratio was determined by ^1^H NMR of the crude reaction mixture. the enantiomeric ratio was determined by chiral HPLC of the *p*-nitrobenzoate ester derivative of the products. The ellipsoid contour was set at a 50% probability level.

As depicted in Scheme 4, we showcased the suitability of the enantioselective method on a one-gram scale using substrate **6a**, which produced the enantioenriched product **(−)-7a** with 98:2 er as a single diastereomer. Equally significant was the fact that pure chiral ligand **14** was readily reclaimed from the reaction mixture in a 93% mass recovery yield after one round of simple flash column chromatography. An application of this method to a complex substrate was exemplified by subjecting *O*-allylformestane **(+)-15** to 2.6 equiv of methylmagnesium bromide in DCM at −78 °C, followed by warming the mixture to reflux. This reaction afforded new steroid structure **(+)-16** as a single diastereomer in a 69% yield. In one synthetic step, our method constructed two tertiary OH groups at the C3 and C17 positions, as well as an allyl quaternary center at the C5 position that was in the *cis* stereoconfiguration relative to C3. The stereochemistry of compound **(+)-16** was confirmed by X-ray crystallography.^12^

**Scheme 4:**
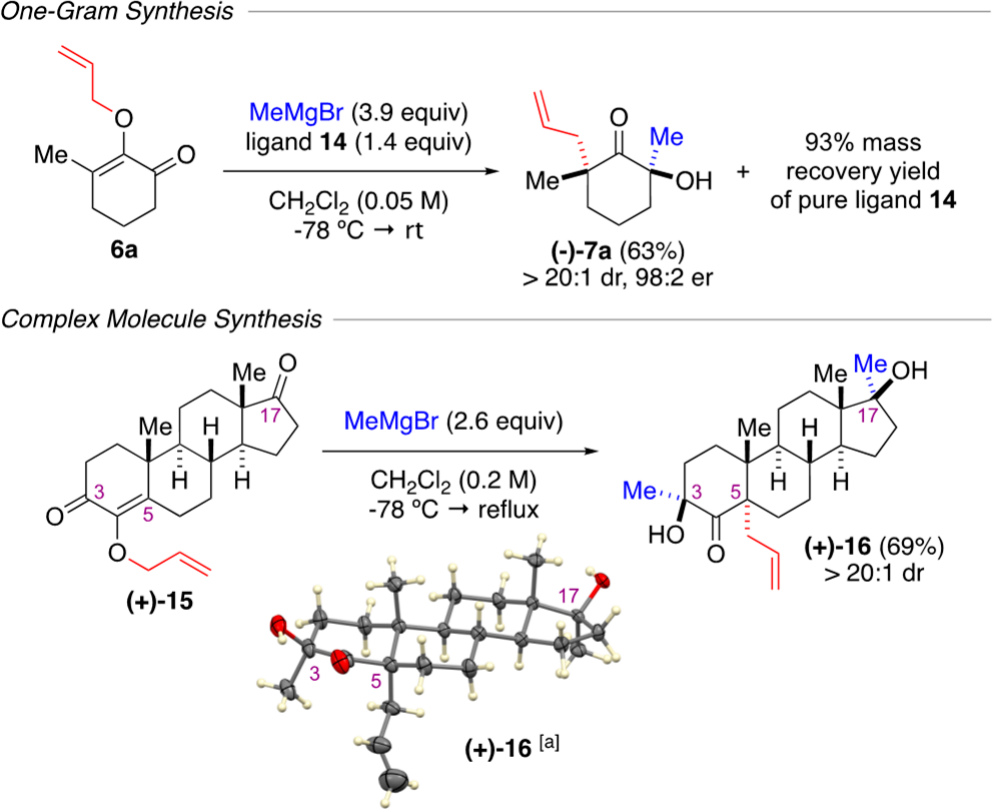
Applications The ellipsoid contour was set at a 50% probability level.

In summary, we demonstrated a new strategy to regulate stereoselectivity in the creation of an α-quaternary center via the Claisen rearrangement from simple monocyclic 2-allyloxyenone systems. The asymmetric method of this chemistry was achieved by employing chiral organomagnesium nucleophiles that were generated *in situ* by combining the Grignard reagents and octahydro-BINOL ligands. These conditions led to the production of tetrasubstituted cyclic ketones at the opposing α-positions with high enantio- and diastereomeric ratios. Further studies of this chemistry are currently ongoing in our laboratory. Our results will be reported in due course.

## Data Availability

The data underlying this study are available in the published article and its Supporting Information.

## Supporting Information Available

Experimental procedures, characterization data, chiral HPLC chromatograms, X-ray crystallographic data, and ^1^H and ^13^ NMR spectra for all new compounds (PDF)
