# Supplementary material for: Directing the Stereoselectivity of the Claisen Rearrangement to Form Cyclic Ketones with Full Substitution at the α-Positions
Source: Org Lett. 2023 Oct 13;25(42):7622–7. doi: 10.1021/acs.orglett.3c02752 (PMC10616857; doi:10.1021/acs.orglett.3c02752)
Supplement: Supplementary file 1 — ol3c02752_si_001.pdf [file ol3c02752_si_001.pdf]

Directing Stereoselectivity of the Claisen Rearrangement to Form Cyclic Ketones with Full Substitution at the α-Positions

Fatimat O. Badmus, Raju S. Thombal,[‡] Satish Chandra Philkhana,[‡] Joshua A. Malone, Christian E. Bailey, Estefania Armendariz-Gonzalez, Edward W. Mureka, Cale M. Locicero, Frank R. Fronczek, and Rendy Kartika*

[‡] Equal Contribution

Department of Chemistry

232 Choppin Hall

Louisiana State University

Baton Rouge, LA 70803, USA

Email: rkartika@lsu.edu

SUPPORTING INFORMATION

TABLE OF CONTENT

1. General Information………………………………………………………………. SI-3

2. Experimental Data for Scheme 2 and Table 1 ………………………….…………... SI-4

3. Screening of Chiral Ligands and Reaction Optimization …………………………... SI-35

4. Experimental Data for Scheme 3 ……………………………………….…………... SI-37

5. Experimental Data for Scheme 4 ……………………………………….…………... SI-94

6. Synthesis of Substrates …….…………………………………………………………. SI-98

7. X-Ray Crystallography Data ……….……………………………………………….. SI-110

8. ^{1}H And ^{13}C NMR Spectra …………………….………….……….…….………. SI-163

GENERAL INFORMATION

Unless otherwise noted, all materials were used as received from commercial suppliers without further purification. All anhydrous reactions were performed using oven-dried glassware, which was then cooled under vacuum and purged with nitrogen gas. Tetrahydrofuran (THF), dichloromethane (CH_2Cl_2), acetonitrile, toluene, diethyl ether (Et_2O), dimethylformamide (DMF), and *n*-hexanes were filtered through activated silica or 3Å molecular sieves under argon contained in a Solvent Purification System. All reactions were monitored by analytical thin layer chromatography (TLC Silica Gel 60 F_{254}, Glass Plates) and analyzed with 254 nm UV light and / or anisaldehyde – sulfuric acid or potassium permanganate treatment. Column chromatography was completed using silica gel (32-63 μ).

Unless otherwise noted, all ^{1}H and ^{13}C NMR spectra were recorded in $CDCl_3$ using a Bruker Ascend 400 spectrometer operating at 400 MHz for ^{1}H and 100 MHz for ^{13}C or Bruker Ascend 500 spectrometer operating at 500 MHz for ^{1}H and 125 MHz for ^{13}C. Chemical shifts (δ) are reported in ppm relative to residual $CHCl_3$ as an internal reference (^{1}H: 7.26 ppm, ^{13}C: 77.00 ppm). Coupling constants (*J*) are reported in Hertz (Hz). Peak multiplicity is indicated as follows: s (singlet), d (doublet), t (triplet), q (quartet), p (pentet), (septet), h (heptet), b (broad), and m (multiplet). FT-IR spectra were recorded on Bruker Tensor 27 spectrometer and OPUS 6.5 Data Collection Program, and absorption frequencies were reported in reciprocal centimeters (cm^{-1}). High Resolution Mass Spectrometry (HRMS) analyses were performed by the Louisiana State University Mass Spectrometry Facility. X-ray structure analyses were performed by the Louisiana State University X-ray Structure Facility. HPLC analyses were performed using Dionex Ultimate 3000 system.

EXPERIMENTAL DATA FOR SCHEME 2 AND TABLE 1

Compound (±)-7a

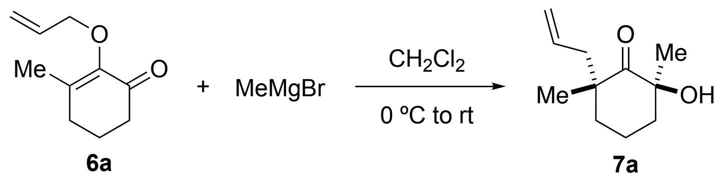

Procedure: Ketone **6a** (150 mg, 0.902 mmol) and CH_2Cl_2 (4.5 mL) were added into a round bottom flask. After cooling the solution to 0 ºC, methylmagnesium bromide (0.39 mL, 1.17 mmol, 3.0 M in Et_2O) was added dropwise. The reaction mixture was allowed to warm to room temperature and stirred for 22 hours, at which the Claisen rearrangement of the carbonyl addition intermediate was complete as monitored by TLC. After cooling to 0 °C, the reaction was quenched with saturated NH_4Cl (5 mL), and the resulting mixture was extracted with CH_2Cl_2 (3 x 5 mL). The organic layers were combined, dried over Na_2SO_4, filtered, and then concentrated under vacuum. The crude material was purified with column chromatography using 100% hexanes to 95:5 hexanes : EtOAc to yield **7a** in 82% yield (135 mg, 0.741 mmol) as a colorless oil. ^{1}H NMR analysis of the crude reaction mixture indicated > 20:1 dr.

Rf: 0.4 in 80:20 Hexanes : EtOAc

^{1}H NMR: (500 MHz, $CDCl_3$) δ = 5.58 (ddt, *J* = 17.1, 10.2, 7.3 Hz, 1H), 5.10 – 5.02 (m, 2H), 3.89 (s, 1H), 2.52 (dd, *J* = 14.1, 6.9 Hz, 1H), 2.17 (dd, *J* = 14.1, 7.6 Hz, 1H), 2.11 – 2.05 (m, 1H), 1.90 – 1.79 (m, 2H), 1.79 – 1.70 (m, 2H), 1.61 – 1.54 (m, 1H), 1.39 (s, 3H), 1.10 (s, 3H).

^{13}C NMR: (125 MHz, $CDCl_3$) δ = 217.5, 133.0, 118.7, 75.8, 47.8, 42.1, 40.5, 38.9, 26.8, 24.2, 18.4.

IR: *f* (cm^{-1}) = 3487, 3076, 2934, 2871, 1698, 1639, 1460, 1374, 1206, 1158, 1012, 977, 918, 872.

HRMS (ESI-TOF): *m/z* $[M-OH]^+$ = 165.1274 calculated for $C_{11}H_{17}O$; found 165.1269.

Compound (±)-8a

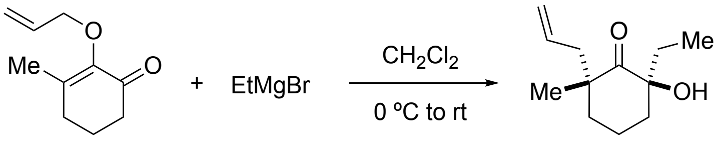

Procedure: Ketone **6a** (147 mg, 0.884 mmol) and CH_2Cl_2 (4.4 mL) were added into a round bottom flask. After cooling the solution to 0 ºC, ethylmagnesium bromide (0.40 mL, 1.19 mmol, 3.0 M in Et_2O) was added dropwise. The reaction mixture was allowed to warm to room temperature and stirred for 22 hours, at which the Claisen rearrangement of the carbonyl addition intermediate was complete as monitored by TLC. After cooling to 0 °C, the reaction was quenched with saturated NH_4Cl (5 mL), and the resulting mixture was extracted with CH_2Cl_2 (3 x 5 mL). The organic layers were combined, dried over Na_2SO_4, filtered, and then concentrated under vacuum. The crude material was purified with column chromatography using 100% hexanes to 95:5 hexanes : EtOAc to yield **8a** in 62% yield (107 mg, 0.545 mmol) as a pale yellow oil. ^{1}H NMR analysis of the crude reaction mixture indicated > 20:1 dr.

Rf: 0.5 in 80:20 Hexanes : EtOAc

^{1}H NMR: (400 MHz, $CDCl_3$) δ = 5.64 (ddt, *J* = 17.0, 10.2, 7.3 Hz, 1H), 5.13 – 5.03 (m, 2H), 3.88 (br s, 1H), 2.38 (dd, *J* = 14.0, 6.8 Hz, 1H), 2.29 (dd, *J* = 14.0, 7.7 Hz, 1H), 2.20 – 2.13 (m, 1H), 1.94 – 1.88 (m, 1H), 1.84 – 1.65 (m, 5H), 1.58 – 1.50 (m, 1H), 1.10 (s, 3H), 0.85 (t, *J* = 7.4 Hz, 3H).

^{13}C NMR: (125 MHz, $CDCl_3$) δ = 217.8, 132.8, 118.8, 78.4, 47.7, 41.2, 38.2, 38.1, 31.6, 24.3, 18.1, 7.4.

IR: *f* (cm^{-1}) = 3482, 2927, 2855, 1696, 1459, 1377, 1260, 1148, 980, 916, 885, 800, 556, 466.

HRMS (ESI-TOF): *m/z* [M-OH]$^+$ = 179.1436 calculated for C12H19O; found 179.1428.

Compound (±)-8b

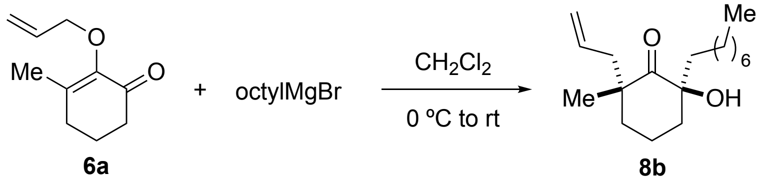

Procedure: Ketone **6a** (153 mg, 0.920 mmol) and CH_2Cl_2 (4.6 mL) were added into a round bottom flask. After cooling the solution to 0 ºC, octylmagnesium bromide (0.60 mL, 1.20 mmol, 2.0 M in Et_2O) was added dropwise. The reaction mixture was allowed to warm to room temperature and stirred for 24 hours, at which the Claisen rearrangement of the carbonyl addition intermediate was complete as monitored by TLC. After cooling to 0 °C, the reaction was quenched with saturated NH_4Cl (5 mL), and the resulting mixture was extracted with CH_2Cl_2 (3 x 5 mL). The organic layers were combined, dried over Na_2SO_4, filtered, and then concentrated under vacuum. The crude material was purified with column chromatography using 100% hexanes to 70:30 hexanes : CH_2Cl_2 to yield **8b** in 72% yield (185 mg, 0.66 mmol) as a colorless oil. ^{1}H NMR analysis of the crude reaction mixture indicated > 20:1 dr.

Rf: 0.6 in 80:20 Hexanes : EtOAc

^{1}H NMR: (500 MHz, $CDCl_3$) δ = 5.64 (ddt, *J* = 17.2, 10.1, 7.3 Hz, 1H), 5.13 – 5.03 (m, 2H), 3.81 (s, 1H), 2.37 (dd, *J* = 13.9, 6.8 Hz, 1H), 2.29 (dd, *J* = 14.0, 7.6 Hz, 1H), 2.18 – 2.12 (m, 1H), 1.93–1.89 (m, 1H), 1.82 – 1.74 (m, 1H), 1.72 – 1.64 (m, 4H), 1.53 (ddd, *J* = 13.8, 11.9, 4.1 Hz, 1H), 1.39 (ddd, *J* = 13.5, 11.8, 5.1 Hz, 1H), 1.30 – 1.22 (m, 10H), 1.13-1.10 (m, 1H), 1.09 (s, 3H), 0.87 (t, *J* = 7.0 Hz, 3H).

^{13}C NMR: (125 MHz, $CDCl_3$) δ = 217.7, 132.9, 118.7, 78.3, 47.7, 41.3, 39.0, 38.6, 38.1, 31.8, 29.8, 29.4, 29.2, 24.4, 22.9, 22.6, 18.1, 14.1.

IR: *f* (cm^{-1}) = 3498, 3077, 2925, 2854, 1695, 1639, 1459, 1378, 1260, 1171, 1094, 996, 804, 772.

HRMS (ESI-TOF): *m/z* $[M+H]^+$ = 281.2475 calculated for $C_{18}H_{33}O_2$; found 281.2467.

Compound (±)-8c

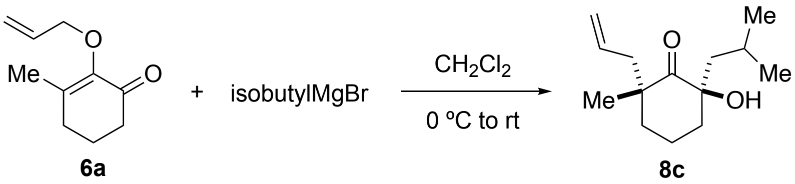

Procedure: Ketone **6a** (156 mg, 0.939 mmol) and CH_2Cl_2 (4.7 mL) were added into a round bottom flask. After cooling the solution to 0 ºC, isobutylmagnesium bromide (0.61 mL, 1.22 mmol, 2.0 M in Et_2O) was added dropwise. The reaction mixture was allowed to warm to room temperature and stirred for 17 hours, at which the Claisen rearrangement of the carbonyl addition intermediate was complete as monitored by TLC. After cooling to 0 °C, the reaction was quenched with saturated NH_4Cl (5 mL), and the resulting mixture was extracted with CH_2Cl_2 (3 x 5 mL). The organic layers were combined, dried over Na_2SO_4, filtered, and then concentrated under vacuum. The crude material was purified with column chromatography using 100% hexanes to 60:40 hexanes : CH_2Cl_2 to yield **8c** in 70% yield (148 mg, 0.66 mmol) as a colorless oil. ^{1}H NMR analysis of the crude reaction mixture indicated > 20:1 dr.

Rf: 0.7 in 80:20 Hexanes : EtOAc

^{1}H NMR: (400 MHz, $CDCl_3$) δ = 5.63 (ddt, *J* = 17.2, 10.2, 7.2 Hz, 1H), 5.13 – 5.04 (m, 2H), 3.71 (s, 1H), 2.36 (dd, *J* = 13.9, 6.7 Hz, 1H), 2.27 (dd, *J* = 14.0, 7.8 Hz, 1H), 2.24 – 2.16 (m, 1H), 1.90 (ddd, *J* = 13.8, 6.7, 3.9 Hz, 1H), 1.83 – 1.71 (m, 2H), 1.69 (d, *J* = 3.9 Hz, 1H), 1.66 (t, *J* = 3.6 Hz, 1H), 1.63 (d, *J* = 6.4 Hz, 1H), 1.58 – 1.48 (m, 2H), 1.09 (s, 3H), 0.98 (d, *J* = 6.4 Hz, 3H), 0.91 (d, *J* = 6.5 Hz, 3H).

^{13}C NMR: (100 MHz, $CDCl_3$) δ = 218.0, 132.9, 118.8, 78.9, 47.7, 46.5, 41.6, 38.5, 37.9, 24.7, 24.6, 24.5, 24.2, 18.1.

IR: *f* (cm^{-1}) = 3495, 2952, 2934, 2870, 1694, 1639, 1459, 1377, 1342, 1168, 1147, 1083, 916.

HRMS (ESI-TOF): *m/z* [M+H]$^+$ = 225.1849 calculated for $C_{14}H_{25}O_2$; found 225.1846.

Compound (±)-8d

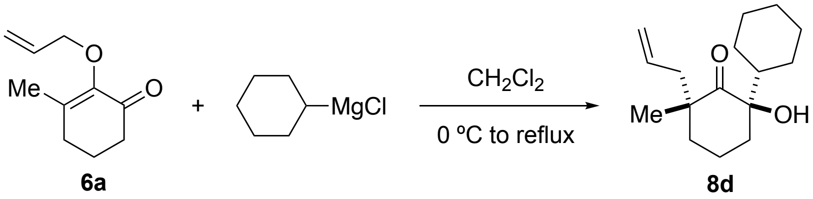

Procedure: Ketone **6a** (153 mg, 0.920 mmol) and toluene (4.6 mL) were added into a pressure vessel. After cooling the solution to 0 ºC, cyclohexylmagnesium chloride (0.92 mL, 1.20 mmol, 1.3 M in THF) was added dropwise. The reaction mixture was allowed to warm to room temperature and stirred for 2 hours until the starting material was fully consumed as monitored by TLC. The pressure vessel was then heated to bring the reaction mixture to gentle reflux for 20 hours, at which the Claisen rearrangement of the carbonyl addition intermediate was complete as monitored by TLC. After cooling to 0 °C, the reaction was quenched with saturated NH_4Cl (5 mL), and the resulting mixture was extracted with CH_2Cl_2 (3 x 5 mL). The organic layers were combined, dried over Na_2SO_4, filtered, and then concentrated under vacuum. The crude material was purified with column chromatography using 100% hexanes to 98:2 hexanes : EtOAc to yield **8d** in 72% yield (167 mg, 0.667 mmol) as a pale yellow oil. ^{1}H NMR analysis of the crude reaction mixture indicated 9:1 dr.

Rf: 0.7 in 80:20 Hexanes : EtOAc

^{1}H NMR: (500 MHz, $CDCl_3$) δ = 5.68 – 5.63 (m, 1H), 5.14 – 5.03 (m, 2H), 3.68 (s, 1H), 2.38 (dd, *J* = 14.1, 7.2 Hz, 1H), 2.34 – 2.27 (m, 1H), 1.95 (dtd, *J* = 13.8, 4.5, 2.2 Hz, 1H), 1.85 – 1.78 (m, 3H), 1.73 (ddd, *J* = 14.4, 9.2, 2.8 Hz, 2H), 1.65 (ddd, *J* = 13.9, 9.2, 4.4 Hz, 2H), 1.53 – 1.46 (m, 2H), 1.30 – 1.21 (m, 2H), 1.15 (tt, *J* = 10.7, 5.1 Hz, 5H), 1.10 (s, 3H).

^{13}C NMR: (125 MHz, $CDCl_3$) δ = 218.1,132.9, 118.8, 80.7, 47.8, 41.9, 41.1, 37.6, 35.0, 26.7, 26.7, 26.2, 26.1, 25.8, 25.0, 17.5.

IR: *f* (cm^{-1}) = 3488, 3076, 2930, 2853, 1692, 1451, 1374, 1232, 1154, 1093, 1072, 989, 916, 879.

HRMS (ESI-TOF): *m/z* $[M+H]^+$ = 251.2006 calculated for $C_{16}H_{27}O_2$; found 251.2004.

Compound (±)-8e

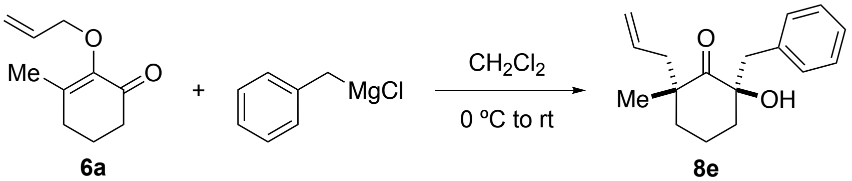

Procedure: Ketone **6a** (152 mg, 0.914 mmol) and CH_2Cl_2 (4.6 mL) were added into a round bottom flask. After cooling the solution to 0 ºC, benzylmagnesium chloride (1.50 mL, 1.20 mmol, 0.8 M in THF) was added dropwise. The reaction mixture was allowed to warm to room temperature and stirred for 30 hours, at which the Claisen rearrangement of the carbonyl addition intermediate was complete as monitored by TLC. After cooling to 0 °C, the reaction was quenched with saturated NH_4Cl (5 mL), and the resulting mixture was extracted with CH_2Cl_2 (3 x 5 mL). The organic layers were combined, dried over Na_2SO_4, filtered, and then concentrated under vacuum. The crude material was purified with column chromatography using 100% hexanes to 60:40 hexanes : CH_2Cl_2 to yield **8e** in 80% yield (188 mg, 0.728 mmol) as a white crystalline solid. ^1H NMR analysis of the crude reaction mixture indicated > 20:1 dr.

Rf: 0.7 in 80:20 Hexanes : EtOAc

^{1}H NMR: (400 MHz, $CDCl_3$) δ = 7.33 – 7.29 (m, 1H), 7.29 (s, 1H), 7.27 – 7.22 (m, 3H), 5.67 (ddt, *J* = 17.4, 10.4, 7.2 Hz, 1H), 5.15 – 5.08 (m, 2H), 3.58 (s, 1H), 3.08 (d, *J* = 13.7 Hz, 1H), 2.80 (d, *J* = 13.7 Hz, 1H), 2.53 (dd, *J* = 14.0, 7.0 Hz, 1H), 2.36 (dd, *J* = 13.9, 7.4 Hz, 1H), 2.04 – 1.98 (m, 1H), 1.97 – 1.84 (m, 2H), 1.81 – 1.72 (m, 1H), 1.67 – 1.56 (m, 2H), 1.17 (s, 3H).

^{13}C NMR: (100 MHz, $CDCl_3$) δ = 217.0, 135.5, 133.1, 130.8, 128.0, 126.7, 118.7, 77.8, 47.8, 43.6, 42.6, 37.8, 36.2, 24.7, 17.6.

IR: *f* (cm^{-1}) = 3490, 3064, 3028, 2934, 2869, 1694, 1639, 1495, 1454, 1376, 1098, 1001, 918.

HRMS (ESI-TOF): *m/z* $[M\text{-}OH]^+$ = 241.1587 calculated for $C_{17}H_{21}O$; found 241.1588.

X-Ray Structure: The ellipsoid contour was set at a 50% probability level.

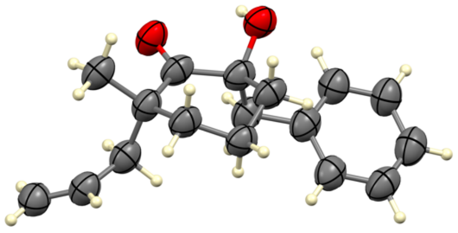

Compound (±)-8f

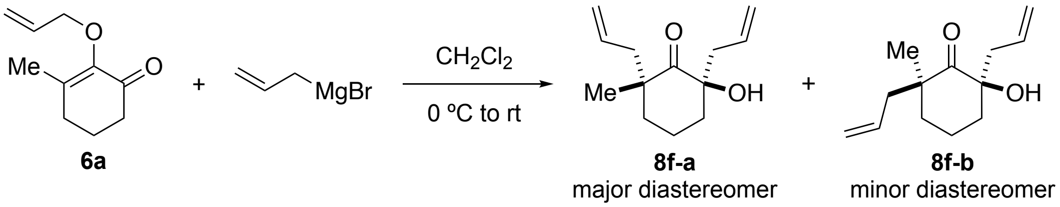

Procedure: Ketone **6a** (154 mg, 0.927 mmol) and CH_2Cl_2 (4.6 mL) were added into a round bottom flask. After cooling the solution to 0 ºC, allylmagnesium bromide (1.20 mL, 1.20 mmol, 1.0 M in Et_2O) was added dropwise. The reaction mixture was allowed to warm to room temperature and stirred for 17 hours, at which the Claisen rearrangement of the carbonyl addition intermediate was complete as monitored by TLC. After cooling to 0 °C, the reaction was quenched with saturated NH_4Cl (5 mL), and the resulting mixture was extracted with CH_2Cl_2 (3 x 5 mL). The organic layers were combined, dried over Na_2SO_4, filtered, and then concentrated under vacuum. The crude material was purified with column chromatography using 100% hexanes to 70:30 hexanes : CH_2Cl_2 to yield the combined diastereomers of **8f** in 81% yield (156 mg, 0.749 mmol) as a colorless oil. ^1H NMR analysis of the crude reaction mixture indicated 3:1 dr.

Major Diastereomer (**8f-a**) – 111 mg

Rf: 0.6 in 80:20 Hexanes : EtOAc

^{1}H NMR: (400 MHz, $CDCl_3$) δ = 5.74 (dddd, *J* = 16.8, 10.3, 7.8, 6.4 Hz, 1H), 5.69 – 5.57 (m, 1H), 5.17 – 5.02 (m, 4H), 3.71 (s, 1H), 2.52 – 2.35 (m, 3H), 2.32 – 2.24 (m, 1H), 2.19 – 2.12 (m, 1H), 1.93 – 1.86 (m, 1H), 1.83 – 1.66 (m, 3H), 1.56 (ddd, *J* = 13.6, 11.0, 4.2 Hz, 1H), 1.10 (s, 3H).

^{13}C NMR: (100 MHz, $CDCl_3$) δ = 216.8, 132.9, 132.2, 119.3, 118.7, 77.5, 47.7, 43.2, 41.6, 37.9, 37.8, 24.3, 17.8.

IR: *f* (cm^{-1}) = 3489, 3077, 2934, 2870, 1698, 1639, 1456, 1376, 1264, 1164, 999, 915, 559.

HRMS (ESI-TOF): *m/z* $[M+H]^+$ = 209.1536 calculated for $C_{13}H_{21}O_2$; found 209.1532.

Minor Diastereomer (**8f-b**) – 45 mg

Rf: 0.7 in 80:20 Hexanes : EtOAc

^{1}H NMR: (400 MHz, $CDCl_3$) δ = 5.93 – 5.81 (m, 1H), 5.61 (dddd, *J* = 17.1, 10.1, 8.4, 5.8 Hz, 1H), 5.14 – 5.02 (m, 4H), 3.93 (s, 1H), 2.68 – 2.56 (m, 2H), 2.47 – 2.37 (m, 3H), 2.16 (dd, *J* = 13.5, 7.7 Hz, 1H), 2.01 – 1.92 (m, 1H), 1.84 – 1.73 (m, 2H), 1.69 – 1.61 (m, 1H), 0.80 (s, 3H).

^{13}C NMR: (100 MHz, $CDCl_3$) δ = 213.7, 134.6, 132.2, 118.1, 117.5, 84.0, 45.5, 41.3, 38.6, 37.7, 33.4, 21.9, 18.5.

IR: *f* (cm^{-1}) = 3473, 3076, 2925, 2856, 1711, 1639, 1451, 1381, 1308, 1150, 1059, 915, 801, 607.

HRMS (ESI-TOF): *m/z* $[M+H]^+$ = 209.1536 calculated for $C_{13}H_{21}O_2$; found 209.1534.

Compound (±)-8g

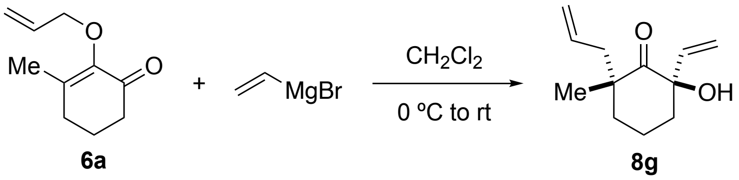

Procedure: Ketone **6a** (152 mg, 0.914 mmol) and CH_2Cl_2 (4.6 mL) were added into a round bottom flask. After cooling the solution to 0 ºC, vinylmagnesium bromide (2.60 mL, 1.17 mmol, 0.45 M in THF) was added dropwise. The reaction mixture was allowed to warm to room temperature and stirred for 20 hours, at which the Claisen rearrangement of the carbonyl addition intermediate was complete as monitored by TLC. After cooling to 0 °C, the reaction was quenched with saturated NH_4Cl (5 mL), and the resulting mixture was extracted with CH_2Cl_2 (3 x 5 mL). The organic layers were combined, dried over Na_2SO_4, filtered, and then concentrated under vacuum. The crude material was purified with column chromatography using 100% hexanes to 98:2 hexanes : EtOAc to yield **8g** in 79% yield (141 mg, 0.726 mmol) as a colorless oil. ^{1}H NMR analysis of the crude reaction mixture indicated > 20:1 dr.

Rf: 0.6 in 80:20 hexanes : EtOAc

^{1}H NMR: (500 MHz, $CDCl_3$) δ = 6.05 (dd, *J* = 17.1, 10.6 Hz, 1H), 5.50 (dddd, *J* = 16.9, 10.3, 8.2, 6.4 Hz, 1H), 5.46 (d, *J* = 17.1 Hz, 1H), 5.26 (d, *J* = 10.6 Hz, 1H), 5.08 – 5.01 (m, 2H), 4.35 (s, 1H), 2.51 (dd, *J* = 14.0, 6.3 Hz, 1H), 2.37– 2.32 (m, 1H), 2.14 (dd, *J* = 14.1, 8.3 Hz, 1H), 2.00 – 1.88 (m, 2H), 1.79 – 1.69 (m, 2H), 1.64 – 1.57 (m, 1H), 1.08 (s, 3H).

^{13}C NMR: (125 MHz, $CDCl_3$) δ = 214.8, 137.8, 132.7, 118.8, 116.2, 78.0, 48.0, 41.5, 39.0, 38.6, 23.7, 18.0.

IR: *f* (cm^{-1}) = 3475, 3078, 2935, 2873, 1698, 1639, 1455, 1377, 1261, 1142, 1093, 994, 894, 683.

HRMS (ESI-TOF): *m/z* $[M-OH]^+$ = 177.1274 calculated for $C_{12}H_{17}O$; found 177.1272.

Compound (±)-8h

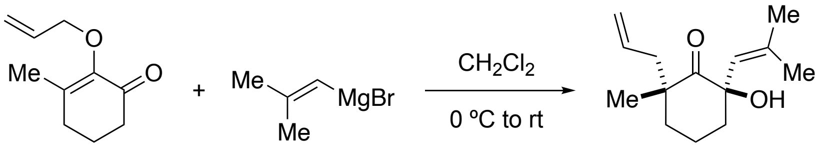

Procedure: Ketone **6a** (151 mg, 0.908 mmol) and CH_2Cl_2 (4.5 mL) were added into a round bottom flask. After cooling the solution to 0 ºC, 2-methyl-1-propenylmagnesium bromide (3.9 mL, 1.17 mmol, 0.3 M in THF) was added dropwise. The reaction mixture was allowed to warm to room temperature and stirred for 28 hours, at which the Claisen rearrangement of the carbonyl addition intermediate was complete as monitored by TLC. After cooling to 0 °C, the reaction was quenched with saturated NH_4Cl (5 mL), and the resulting mixture was extracted with CH_2Cl_2 (3 x 5 mL). The organic layers were combined, dried over Na_2SO_4, filtered, and then concentrated under vacuum. The crude material was purified with column chromatography using 100% hexanes to 70:30 hexanes : CH_2Cl_2 to yield **8h** in 70% yield (141 mg, 0.63 mmol) as a colorless oil. ^{1}H NMR analysis of the crude reaction mixture indicated > 20:1 dr.

Rf: 0.7 in 80:20 hexanes : EtOAc

^{1}H NMR: (400 MHz, $CDCl_3$) δ = 5.48 – 5.37 (m, 1H), 5.47 (s, 1H), 5.03 (d, *J* = 9.5 Hz, 1H), 4.99 (d, *J* = 17 Hz, 1H), 4.42 (s, 1H), 2.58 (dd, *J* = 14.2, 6.0 Hz, 1H), 2.30 – 2.25 (m, 1H), 2.15 (dd, *J* = 14.1, 8.1 Hz, 1H), 1.98 – 1.84 (m, 2H), 1.73 (s, 3H), 1.69 – 1.49 (m, 6H), 1.07 (s, 3H).

^{13}C NMR: (100 MHz, $CDCl_3$) δ = 214.8, 140.2, 133.1, 124.6, 118.3, 48.0, 43.1, 42.1, 40.0, 27.1, 23.6, 19.0, 17.8.

IR: *f* (cm^{-1}) = 3482, 3076, 2969, 2933, 2857, 1693, 1451, 1377, 1344, 1205, 1073, 1008, 907.

HRMS (ESI-TOF): *m/z* $[M-OH]^+$ = 205.1587 calculated for $C_{14}H_{21}O$; found 205.1582.

Compound (±)-8i

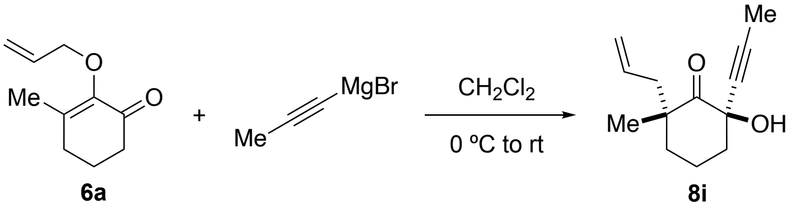

Procedure: Ketone **6a** (153 mg, 0.921 mmol) and CH_2Cl_2 (4.6 mL) were added into a round bottom flask. After cooling the solution to 0 ºC, propyn-1-ylmagnesium bromide (2.40 mL, 1.20 mmol, 0.5 M in THF) was added dropwise. The reaction mixture was allowed to warm to room temperature and stirred for 27 hours, at which the Claisen rearrangement of the carbonyl addition intermediate was complete as monitored by TLC. After cooling to 0 °C, the reaction was quenched with saturated NH_4Cl (5 mL), and the resulting mixture was extracted with CH_2Cl_2 (3 x 5 mL). The organic layers were combined, dried over Na_2SO_4, filtered, and then concentrated under vacuum. The crude material was purified with column chromatography using 100% hexanes to 90:10 hexanes : CH_2Cl_2 to yield **8i** in 56% yield (106 mg, 0.514 mmol) as a colorless oil. ^{1}H NMR analysis of the crude reaction mixture indicated > 20:1 dr.

Rf: 0.6 in 80:20 hexanes : EtOAc

^{1}H NMR: (400 MHz, $CDCl_3$) δ = 5.67 – 5.55 (m, 1H), 5.09 (s, 1H), 5.07 – 5.03 (d, *J* = 6.1Hz, 1H), 4.35 (s, 1H), 3.03 (dd, *J* = 13.9, 5.9 Hz, 1H), 2.44 – 2.38 (m, 1H), 2.20 (dd, *J* = 13.9, 8.7 Hz, 1H), 2.16 – 2.06 (m, 1H), 1.92 – 1.86 (m, 1H), 1.84 (d, *J* = 0.6 Hz, 3H), 1.71 – 1.62 (m, 2H), 1.59 – 1.50 (m, 1H), 1.06 (s, 3H).

^{13}C NMR: (100 MHz, $CDCl_3$) δ = 210.6, 133.4, 118.4, 83.4, 79.2, 72.2, 48.6, 43.6, 41.7, 39.9, 23.6, 18.3, 3.6.

IR: *f* (cm^{-1}) = 3475, 3077, 2937, 2858, 1707, 1640, 1451, 1377, 1213, 1107, 1066, 991, 915, 887.

HRMS (ESI-TOF): *m/z* $[M+H]^+$ = 207.1380 calculated for $C_{13}H_{19}O_2$; found 207.1375.

Compound (±)-8j

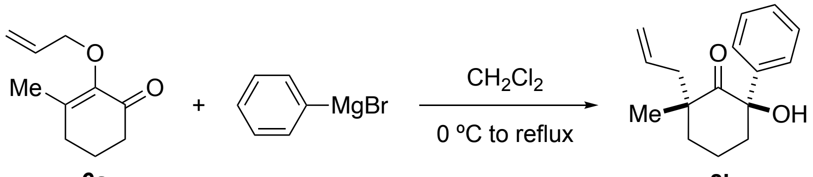

Procedure: Ketone **6a** (143 mg, 0.860 mmol) and CH_2Cl_2 (4.3 mL) were added into a pressure vessel. After cooling the solution to 0 ºC, phenylmagnesium bromide (0.37 mL, 1.11 mmol, 3.0 M in Et_2O) was added dropwise. The reaction mixture was allowed to warm to room temperature and stirred for 1 hour until the starting material was fully consumed as monitored by TLC. The pressure vessel was then heated to bring the reaction mixture to gentle reflux for 28 hours, at which the Claisen rearrangement of the carbonyl addition intermediate was complete as monitored by TLC. After cooling to 0 °C, the reaction was quenched with saturated NH_4Cl (5 mL), and the resulting mixture was extracted with CH_2Cl_2 (3 x 5 mL). The organic layers were combined, dried over Na_2SO_4, filtered, and then concentrated under vacuum. The crude material was purified with column chromatography using 100% hexanes to 95:5 hexanes : EtOAc to yield **8j** in 79% yield (167 mg, 0.68 mmol) as a colorless oil. ^1H NMR analysis of the crude reaction mixture indicated 15:1 dr.

Rf: 0.5 in 80:20 hexanes : EtOAc

^{1}H NMR: (400 MHz, $CDCl_3$) δ = 7.38 – 7.27 (m, 5H), 5.35 (ddt, J = 16.8, 10.1, 7.3 Hz, 1H), 4.90 (dd, J = 10.1, 2.1 Hz, 1H), 4.77 (s, 1H), 4.72 – 4.67 (m, 1H), 3.00 – 2.94 (m, 1H), 2.09 – 1.93 (m, 2H), 1.91 – 1.79 (m, 3H), 1.75 – 1.68 (m, 1H), 1.63 – 1.54 (m, 1H), 1.10 (s, 3H).

^{13}C NMR: (100 MHz, $CDCl_3$) δ = 215.6, 140.1, 132.5, 128.8, 128.2, 125.8, 118.5, 78.3, 48.3, 41.7, 38.1, 36.8, 23.4, 18.5.

IR: f (cm^{-1}) = 3466, 2935, 1695, 1639, 1449, 1376, 1265, 1189, 989, 918, 885, 771, 730, 699.

HRMS (ESI-TOF): *m/z* $[M-OH]^+$ = 227.1430 calculated for $C_{16}H_{19}O$; found 227.1427.

Compound (±)-8k

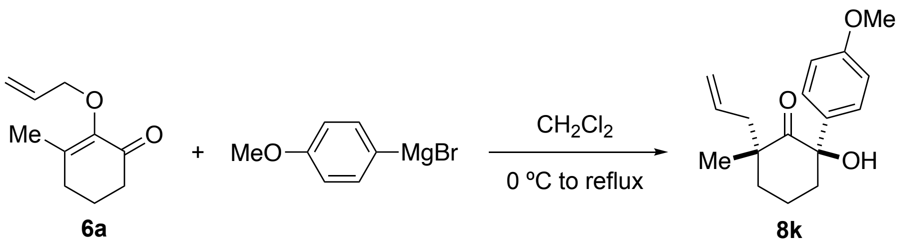

Procedure: Ketone **6a** (152 mg, 0.914 mmol) and CH_2Cl_2 (4.6 mL) were added into a pressure vessel. After cooling the solution to 0 ºC, p-methoxyphenylmagnesium bromide (1.20 mL, 1.20 mmol, 1.0 M in THF) was added dropwise. The reaction mixture was allowed to warm to room temperature and stirred for 2 hours until the starting material was fully consumed as monitored by TLC. The pressure vessel was then heated to bring the reaction mixture to gentle reflux for 44 hours, at which the Claisen rearrangement of the carbonyl addition intermediate was complete as monitored by TLC. After cooling to 0 °C, the reaction was quenched with saturated NH_4Cl (5 mL), and the resulting mixture was extracted with CH_2Cl_2 (3 x 5 mL). The organic layers were combined, dried over Na_2SO_4, filtered, and then concentrated under vacuum. The crude material was purified with column chromatography using 100% hexanes to 95:5 hexanes : EtOAc to yield **8k** in 61% yield (152 mg, 0.554 mmol) as a pale yellow oil. ^1H NMR analysis of the crude reaction mixture indicated 14:1 dr.

Rf: 0.4 in 80:20 hexanes : EtOAc

^{1}H NMR: (400 MHz, $CDCl_3$) δ = 7.30 – 7.25 (m, 2H), 6.89 – 6.84 (m, 2H), 5.36 (dddd, *J* = 16.8, 10.2, 8.2, 6.5 Hz, 1H), 4.94 – 4.90 (m, 1H), 4.77 – 4.68 (m, 2H), 3.79 (s, 3H), 2.93 (dq, *J* = 13.5, 3.0 Hz, 1H), 2.05 – 1.90 (m, 2H), 1.89 – 1.76 (m, 3H), 1.76 – 1.68 (m, 1H), 1.62 – 1.52 (m, 1H), 1.08 (s, 3H).

^{13}C NMR: (125 MHz, $CDCl_3$) δ = 215.8, 159.3, 132.6, 132.2, 127.1, 118.5, 114.1, 77.8, 55.2, 48.2, 41.8, 38.0, 36.9, 23.4, 18.5.

IR: *f* (cm^{-1}) = 3478, 3075, 2935, 2873, 2837, 1695, 1639, 1511, 1296, 1181, 1033, 989, 827, 542.

HRMS (ESI-TOF): *m/z* $[M+H]^+$ = 275.1642 calculated for $C_{17}H_{23}O_3$; found 275.1641.

Compound (±)-8l

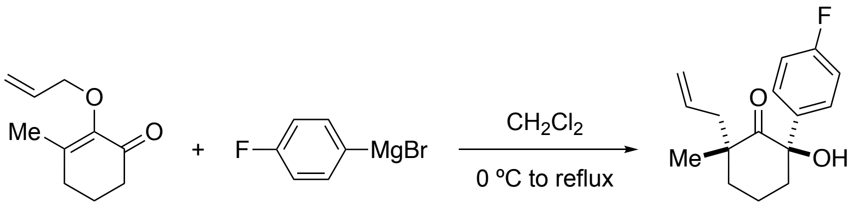

6a **8l**

Procedure: Ketone **6a** (152 mg, 0.914 mmol) and CH_2Cl_2 (4.6 mL) were added into a pressure vessel. After cooling the solution to 0 ºC, p-fluorophenylmagnesium bromide (1.80 mL, 1.19 mmol, 0.66 M in THF) was added dropwise. The reaction mixture was allowed to warm to room temperature and stirred for 1 hour until the starting material was fully consumed as monitored by TLC. The pressure vessel was then placed in a warm sand bath to bring the reaction mixture to gentle reflux for 26 hours, at which the Claisen rearrangement of the carbonyl addition intermediate was complete as monitored by TLC. After cooling to 0 °C, the reaction was quenched with saturated NH_4Cl (5 mL), and the resulting mixture was extracted with CH_2Cl_2 (3 x 5 mL). The organic layers were combined, dried over Na_2SO_4, filtered, and then concentrated under vacuum. The crude material was purified with column chromatography using 100% hexanes to 50:50 hexanes : CH_2Cl_2 to yield **8l** in 72% yield (173 mg, 0.659 mmol) as a colorless oil. ^{1}H NMR analysis of the crude reaction mixture indicated 14:1 dr.

Rf: 0.5 in 80:20 hexanes : EtOAc

^{1}**H NMR:** (400 MHz, $CDCl_3$) δ = 7.37 – 7.32 (m, 2H), 7.09 – 6.96 (m, 2H), 5.40 – 5.29 (m, 1H), 4.94 – 4.94 (m, 1H), 4.79 – 4.67 (m, 2H), 2.97 – 2.86 (m, 1H), 2.03 – 1.93 (m, 2H), 1.93 – 1.77 (m, 4H), 1.77 – 1.64 (m, 1H), 1.64 – 1.53 (m, 1H), 1.09 (s, 3H).

^{13}**C NMR:** (100 MHz, $CDCl_3$) δ = 215.4, 162.4 (d, J = 246.3 Hz), 136.2 (d, J = 3.3 Hz), 132.3, 127.8 (d, J = 8.1 Hz), 118.7, 115.7 (d, J = 21.3 Hz), 77.8, 48.4, 41.7, 38.0, 37.1, 23.4, 18.4.

IR: f (cm^{-1}) = 3468, 3076, 2936, 2873, 1697, 1602, 1509, 1460, 1226, 1164, 1098, 990, 745, 595.

HRMS (ESI-TOF): *m/z* $[M+H]^+$ = 263.1442 calculated for $C_{16}H_{20}FO_2$; found 263.1433.

Compound (±)-8m

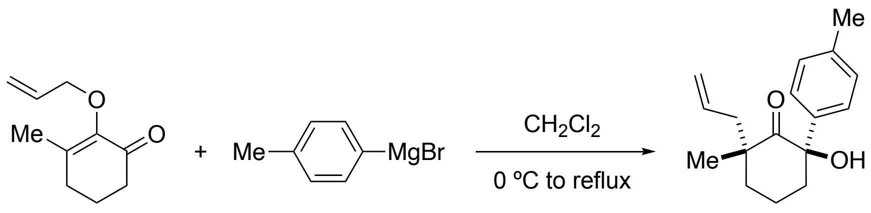

6a **8m**

Procedure: Ketone **6a** (152 mg, 0.914 mmol) and CH_2Cl_2 (4.6 mL) were added into a pressure vessel. After cooling the solution to 0 ºC, p-methylphenylmagnesium bromide (1.20 mL, 1.20 mmol, 1.0 M in THF) was added dropwise. The reaction mixture was allowed to warm to room temperature and stirred for 16 hour until the starting material was fully consumed as monitored by TLC. The pressure vessel was then heated to bring the reaction mixture to gentle reflux for 48 hours, at which the Claisen rearrangement of the carbonyl addition intermediate was complete as monitored by TLC. After cooling to 0 °C, the reaction was quenched with saturated NH_4Cl (5 mL) and the resulting mixture was extracted with CH_2Cl_2 (3 x 5 mL). The organic layers were combined, dried over Na_2SO_4, filtered, and then concentrated under vacuum. The crude material was purified with column chromatography using 100% hexanes to 50:50 hexanes : CH_2Cl_2 to yield

8m in 68% yield (161 mg, 0.62 mmol) as a pale yellow oil. ^{1}H NMR analysis of the crude reaction mixture indicated > 20:1 dr.

Rf: 0.5 in 80:20 hexanes : EtOAc

^{1}H NMR: (400 MHz, $CDCl_3$) δ = 7.33 – 7.28 (m, 2H), 7.04 (t, *J* = 8.6 Hz, 2H), 5.77 (ddt, *J* = 17.3, 10.2, 7.4 Hz, 1H), 5.09 – 5.01 (m, 2H), 2.91 (ddd, *J* = 10.5, 4.0, 2.7 Hz, 1H), 2.38 – 2.26 (m, 2H), 1.96 – 1.80 (m, 4H), 1.78 – 1.70 (m, 1H), 1.67 – 1.61 (m, 1H), 0.65 (s, 3H).

^{13}C NMR: (100 MHz, $CDCl_3$) δ = 215.6, 163.4, 161.4, 136.4, 136.4, 134.1, 127.7, 127.6, 118.4, 115.8, 115.6, 78.0, 48.0, 43.6, 37.8, 36.9, 24.1, 18.6.

IR: *f* (cm^{-1}) = 3476, 2936, 2872, 1695, 1453, 1377, 989, 917, 813, 542.

HRMS (ESI-TOF): *m/z* $[M+H]^+$ = 259.1693 calculated for $C_{17}H_{23}O_2$; found 259.1677.

Compound (±)-8n

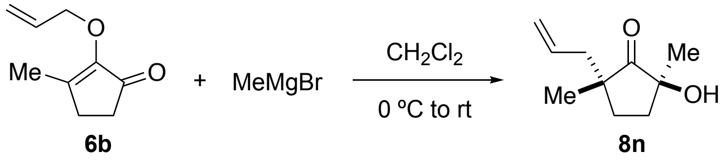

Procedure: Ketone **6b** (152 mg, 0.999 mmol) and CH_2Cl_2 (5.0 mL) were added into a round bottom flask. After cooling the solution to 0 ºC, methylmagnesium bromide (0.43 mL, 1.30 mmol, 3.0 M in Et_2O) was added dropwise. The reaction mixture was allowed to warm to room temperature and stirred for 14 hours, at which the Claisen rearrangement of the carbonyl addition intermediate was complete as monitored by TLC. After cooling to 0 °C, the reaction was quenched with saturated NH_4Cl (5 mL), and the resulting mixture was extracted with CH_2Cl_2 (3 x 5 mL). The organic layers were combined, dried over Na_2SO_4, filtered, and then concentrated under vacuum. The crude material was purified with column chromatography using 100% hexanes to

90:10 hexanes : EtOAc to yield **8n** in 71% yield (119 mg, 0.707 mmol) as a colorless oil. ^{1}H NMR analysis of the crude reaction mixture indicated > 20:1 dr.

Rf: 0.5 in 80:20 hexanes : EtOAc

^{1}H NMR: (400 MHz, $CDCl_3$) δ = 5.70 – 5.58 (m, 1H), 5.10 – 4.99 (m, 2H), 2.67 (s, 1H), 2.20 (dd, *J* = 13.7, 6.9 Hz, 1H), 2.13 (dd, *J* = 13.7, 8.0 Hz, 1H), 2.02 – 1.90 (m, 2H), 1.85 – 1.74 (m, 1H), 1.73 – 1.65 (m, 1H), 1.20 (s, 3H), 1.07 (s, 3H).

^{13}C NMR: (100 MHz, $CDCl_3$) δ = 223.5, 133.4, 118.6, 77.4, 47.0, 42.1, 33.9, 30.3, 23.5, 23.4.

IR: *f* (cm^{-1}) = 3448, 3078, 2963, 2927, 2873, 1740, 1640, 1455, 1373, 1303, 1064, 1034, 916.

HRMS (ESI-TOF): *m/z* $[M-OH]^+$ = 151.1123 calculated for $C_{10}H_{15}O$; found 151.1121.

Compound (±)-8o

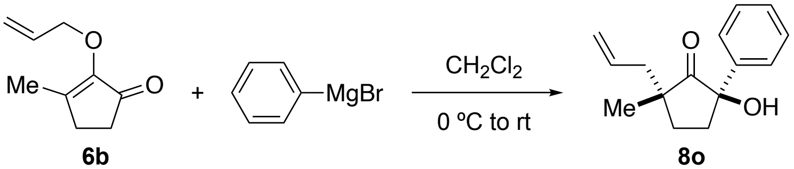

6b 8o

Procedure: Ketone **6b** (156 mg, 1.03 mmol) and CH_2Cl_2 (5.1 mL) were added into a round bottom flask. After cooling the solution to 0 ºC, phenylmagnesium bromide (0.44 mL, 1.33 mmol, 3.0 M in Et_2O) was added dropwise. The reaction mixture was allowed to warm to room temperature and stirred for 29 hours, at which the Claisen rearrangement of the carbonyl addition intermediate was complete as monitored by TLC. After cooling to 0 °C, the reaction was quenched with saturated NH_4Cl (5 mL), and the resulting mixture was extracted with CH_2Cl_2 (3 x 5 mL). The organic layers were combined, dried over Na_2SO_4, filtered, and then concentrated under vacuum. The crude material was purified with column chromatography using 100% hexanes to 97:3 hexanes : EtOAc to yield **8o** in 61% yield (145 mg, 0.63 mmol) as a white crystalline solid. ^{1}H NMR analysis of the crude reaction mixture indicated > 20:1 dr.

Rf: 0.5 in 80:20 hexanes : EtOAc

^{1}H NMR: (400 MHz, $CDCl_3$) δ = 7.83 – 7.35 (m, 4H), 7.34 – 7.28 (m, 1H), 5.61 (ddt, *J* = 16.5, 10.6, 7.4 Hz, 1H), 5.05 – 4.96 (m, 2H), 2.89 (s, 1H), 2.50 (dt, *J* = 13.7, 5.9 Hz, 1H), 2.27 – 2.06 (m, 3H), 1.85 (dd, *J* = 8.1, 6.0 Hz, 2H), 1.19 (s, 3H).

^{13}C NMR: (100 MHz, $CDCl_3$) δ = 221.4, 140.3, 133.0, 128.6, 128.2, 126.0, 118.5, 81.1, 47.3, 41.8, 34.3, 31.2, 23.4.

IR: *f* (cm^{-1}) = 3467, 2958, 2925, 2855, 1740, 1457, 1375, 1052, 915, 763, 699, 550, 410.

HRMS (ESI-TOF): *m/z* $[M-OH]^+$ = 213.1279 calculated for $C_{15}H_{17}O$; found 213.1281.

X-Ray Structure: The ellipsoid contour was set at a 50% probability level.

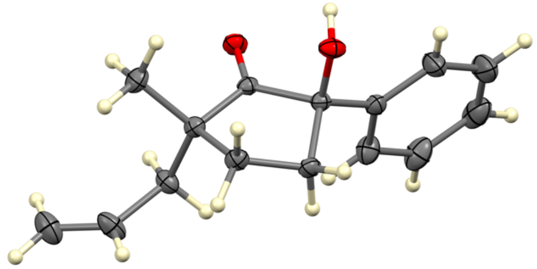

Compound (±)-10a

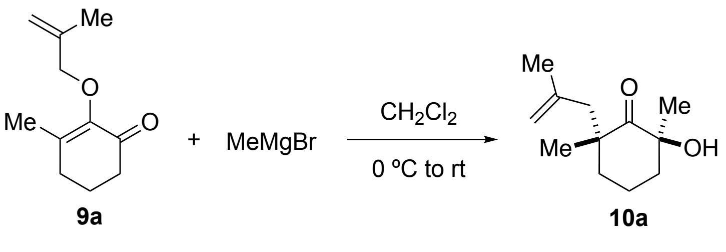

Procedure: Ketone **9a** (61 mg, 0.34 mmol) and CH_2Cl_2 (1.7 mL) were added into a round bottom flask. After cooling the solution to 0 ºC, methylmagnesium bromide (0.15 mL, 0.44 mmol, 3.0 M in Et_2O) was added dropwise. The reaction mixture was allowed to warm to room temperature and stirred for 44 hours, at which the Claisen rearrangement of the carbonyl addition intermediate

was complete as monitored by TLC. After cooling to 0 °C, the reaction was quenched with saturated NH_4Cl (5 mL), and the resulting mixture was extracted with CH_2Cl_2 (3 x 5 mL). The organic layers were combined, dried over Na_2SO_4, filtered, and then concentrated under vacuum. The crude material was purified with column chromatography using 100% hexanes to 98:2 hexanes : EtOAc to yield **10a** in 89% yield (59 mg, 0.30 mmol) as a colorless oil. ^{1}H NMR analysis of the crude reaction mixture indicated > 20:1 dr.

Rf: 0.4 in 80:20 hexanes : EtOAc

^{1}H NMR: (400 MHz, $CDCl_3$) δ = 4.89 – 4.84 (m, 1H), 4.66 (s, 1H), 3.92 (b s, 1H), 2.41 (d, *J* = 14.3 Hz, 1H), 2.33 (d, *J* = 14.3 Hz, 1H), 2.12 – 2.04 (m, 1H), 1.98 – 1.92 (m, 1H), 1.87 – 1.70 (m, 3H), 1.69 (s, 3H), 1.59 – 1.49 (m, 1H), 1.42 (s, 3H), 1.15 (s, 3H).

^{13}C NMR: (100 MHz, $CDCl_3$) δ = 218.2, 141.5, 115.2, 76.0, 47.9, 45.1, 40.8, 39.4, 27.2, 24.9, 24.7, 18.3.

IR: *f* (cm^{-1}) = 3495, 3075, 2926, 2856, 1701, 1643, 1459, 1375, 1159, 1010, 977, 893.

HRMS (ESI-TOF): *m/z* $[M+H]^+$ = 197.1541 calculated for $C_{12}H_{21}O_2$; found 197.1544.

Compound (±)-10b

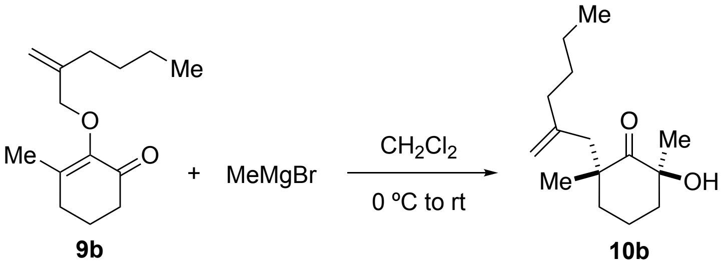

Procedure: Ketone **9b** (121 mg, 0.544 mmol) and CH_2Cl_2 (2.7 mL) were added into a round bottom flask. After cooling the solution to 0 ºC, methylmagnesium bromide (0.23 mL, 0.71 mmol, 3.0 M in Et_2O) was added dropwise. The reaction mixture was allowed to warm to room

temperature and stirred for 19 hours, at which the Claisen rearrangement of the carbonyl addition intermediate was complete as monitored by TLC. After cooling to 0 °C, the reaction was quenched with saturated NH_4Cl (5 mL), and the resulting mixture was extracted with CH_2Cl_2 (3 x 5 mL). The organic layers were combined, dried over Na_2SO_4, filtered, and then concentrated under vacuum. The crude material was purified with column chromatography using 100% hexanes to 98:2 hexanes : EtOAc to yield **10b** in 83% yield (108 mg, 0.453 mmol) as a pale yellow oil. ^{1}H NMR analysis of the crude reaction mixture indicated > 20:1 dr.

Rf: 0.4 in 80:20 hexanes : EtOAc

^{1}H NMR: (400 MHz, $CDCl_3$) δ = 4.88 (s, 1H), 4.68 (s, 1H), 3.86 (br s, 1H), 2.35 (s, 2H), 2.11 – 2.07 (m, 1H), 2.00 – 1.96 (m, 3H), 1.87 – 1.68 (m, 3H), 1.56 – 1.48 (m, 1H), 1.42 (s, 3H), 1.41 – 1.36 (m, 2H), 1.36 – 1.24 (m, 2H), 1.14 (s, 3H), 0.89 (t, *J* = 7.2 Hz, 3H).

^{13}C NMR: (100 MHz, $CDCl_3$) δ = 218.4, 145.6, 113.5, 76.0, 48.1, 43.0, 40.8, 39.1, 37.7, 30.3, 27.1, 24.9, 22.4, 18.3, 14.0.

IR: *f* (cm^{-1}) = 3483, 2931, 2868, 1699, 1640, 1459, 1260, 1373, 1158, 1013, 895.

HRMS (ESI-TOF): *m/z* $[M+H]^+$ = 239.2006 calculated for $C_{15}H_{27}O_2$; found 239.1994.

Compound (±)-10c

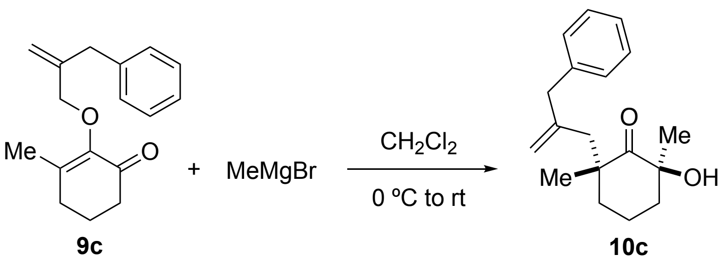

Procedure: Ketone **9c** (143 mg, 0.558 mmol) and CH_2Cl_2 (2.8 mL) were added into a round bottom flask. After cooling the solution to 0 ºC, methylmagnesium bromide (0.24 mL, 0.73 mmol,

3.0 M in Et_2O) was added dropwise. The reaction mixture was allowed to warm to room temperature and stirred for 20 hours, at which the Claisen rearrangement of the carbonyl addition intermediate was complete as monitored by TLC. After cooling to 0 °C, the reaction was quenched with saturated NH_4Cl (5 mL), and the resulting mixture was extracted with CH_2Cl_2 (3 x 5 mL). The organic layers were combined, dried over Na_2SO_4, filtered, and then concentrated under vacuum. The crude material was purified with column chromatography using 100% hexanes to 98:2 hexanes : EtOAc to yield **10c** in 84% yield (127 mg, 0.466 mmol) as a pale yellow oil. ^{1}H NMR analysis of the crude reaction mixture indicated > 20:1 dr.

Rf: 0.5 in 80:20 hexanes : EtOAc

^{1}H NMR: (500 MHz, $CDCl_3$) δ = 7.29 (t, *J* = 7.4 Hz, 2H), 7.23 – 7.19 (m, 1H), 7.19 – 7.11 (m, 2H), 4.88 (d, *J* = 1.4 Hz, 1H), 4.82 (s, 1H), 3.83 (br s, 1H), 3.30 (s, 2H), 2.37 (d, *J* = 14.9 Hz, 1H), 2.26 (d, *J* = 14.9 Hz, 1H), 2.10 – 2.03 (m, 1H), 1.98 (dq, *J* = 14.1, 3.2 Hz, 1H), 1.76 – 1.69 (m, 3H), 1.58 – 1.51 (m, 1H), 1.32 (s, 3H), 1.19 (s, 3H).

^{13}C NMR: (125 MHz, $CDCl_3$) δ = 218.3, 144.4, 139.1, 129.0, 128.3, 126.2, 116.0, 76.1, 48.1, 44.9, 42.0, 40.9, 39.5, 26.8, 24.9, 18.3.

IR: *f* (cm^{-1}) = 3480, 3027, 2932, 2869, 1697, 1640, 1454, 1372, 1261, 1158, 1013, 903, 804, 741.

HRMS (ESI-TOF): *m/z* $[M+H]^+$ = 273.1849 calculated for $C_{18}H_{25}O_2$; found 273.1847.

Compound (±)-10d

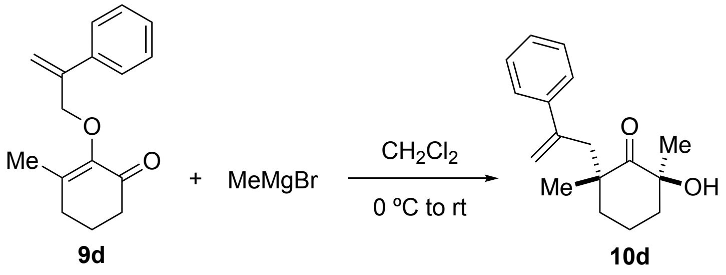

Procedure: Ketone **9d** (144 mg, 0.594 mmol) and CH_2Cl_2 (2.9 mL) were added into a round bottom flask. After cooling the solution to 0 ºC, methylmagnesium bromide (0.25 mL, 0.77 mmol, 3.0 M in Et_2O) was added dropwise. The reaction mixture was allowed to warm to room temperature and stirred for 23 hours, at which the Claisen rearrangement of the carbonyl addition intermediate was complete as monitored by TLC. After cooling to 0 °C, the reaction was quenched with saturated NH_4Cl (5 mL) and the resulting mixture was extracted with CH_2Cl_2 (3 x 5 mL). The organic layers were combined, dried over Na_2SO_4, filtered, and then concentrated under vacuum. The crude material was purified with column chromatography using 100% hexanes to 95:5 hexanes : EtOAc to yield **10d** in 89% yield (136 mg, 0.526 mmol) as a colorless oil. ^{1}H NMR analysis of the crude reaction mixture indicated > 20:1 dr.

Rf: 0.4 in 80:20 hexanes : EtOAc

^{1}H NMR: (500 MHz, $CDCl_3$) δ = 7.36 – 7.23 (m, 5H), 5.30 (d, *J* = 1.4 Hz, 1H), 5.06 (s, 1H), 3.74 (b s, 1H), 2.96 (d, *J* = 14.0 Hz, 1H), 2.67 (d, *J* = 14.0 Hz, 1H), 2.05 – 1.99 (m, 1H), 1.87 – 1.79 (m, 1H), 1.78 – 1.68 (m, 2H), 1.66 – 1.61 (m, 1H), 1.42 – 1.32 (m, 4H), 1.00 (s, 3H).

^{13}C NMR: (125 MHz, $CDCl_3$) δ = 218.1, 145.0, 142.9, 128.3, 127.4, 126.5, 118.0, 75.8, 48.3, 41.6, 39.8, 37.1, 27.2, 25.0, 18.0.

IR: *f* (cm^{-1}) = 3479, 2932, 1697, 1623, 1574, 1493, 1454, 1372, 1151, 1013, 905, 778, 699.

HRMS (ESI-TOF): *m/z* $[M+H]^+$ = 259.1698 calculated for $C_{17}H_{23}O_2$; found 259.1697.

Compound (±)-10e

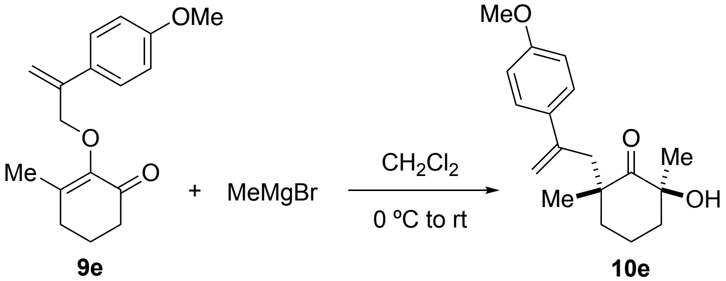

Procedure: Ketone **9e** (125 mg, 0.459 mmol) and CH_2Cl_2 (2.3 mL) were added into a round bottom flask. After cooling the solution to 0 ºC, methylmagnesium bromide (0.20 mL, 0.60 mmol, 3.0 M in Et_2O) was added dropwise. The reaction mixture was allowed to warm to room temperature and stirred for 40 hours, at which the Claisen rearrangement of the carbonyl addition intermediate was complete as monitored by TLC. After cooling to 0 °C, the reaction was quenched with saturated NH_4Cl (5 mL), and the resulting mixture was extracted with CH_2Cl_2 (3 x 5 mL). The organic layers were combined, dried over Na_2SO_4, filtered, and then concentrated under vacuum. The crude material was purified with column chromatography using 100% hexanes to 97:3 hexanes : EtOAc to yield **10e** in 87% yield (115 mg, 0.399 mmol) as a pale yellow oil. ^{1}H NMR analysis of the crude reaction mixture indicated > 20:1 dr.

Rf: 0.3 in 80:20 hexanes : EtOAc

^{1}H NMR: (400 MHz, $CDCl_3$) δ = 7.29 – 7.23 (m, 2H), 6.87 – 6.81 (m, 2H), 5.23 (d, *J* = 1.6 Hz, 1H), 4.98 (d, *J* = 1.4 Hz, 1H), 3.81 (s, 3H), 3.81 (s, 1H), 2.96 – 2.90 (m, 1H), 2.63 (d, *J* = 13.9 Hz, 1H), 2.06 – 1.97 (m, 1H), 1.90 – 1.81 (m, 1H), 1.81 – 1.70 (m, 2H), 1.68 – 1.61 (m, 1H), 1.39-1.34 (m, 1H), 1.36 (s, 3H), 1.01 (s, 3H).

^{13}C NMR: (100 MHz, $CDCl_3$) δ = 218.1, 159.1, 144.4, 135.4, 127.6, 116.6, 113.6, 75.8, 55.2, 48.3, 41.7, 39.7, 37.0, 27.2, 25.1, 18.0.

IR: *f* (cm^{-1}) = 3481, 2928, 1699, 1607, 1511, 1459, 1285, 1245, 1179, 1034, 1013, 903, 836.

HRMS (ESI-TOF): *m/z* $[M+H]^+$ = 289.1803 calculated for $C_{18}H_{25}O_3$; found 289.1799.

Compound (±)-10f

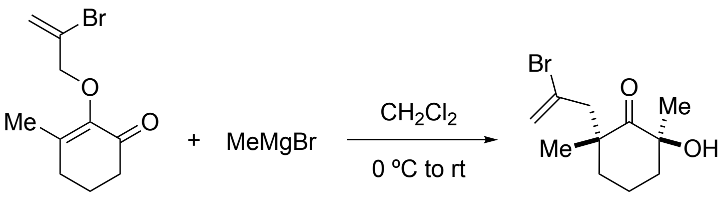

Procedure: Ketone **9f** (150 mg, 0.612 mmol) and CH_2Cl_2 (3.0 mL) were added into a round bottom flask. After cooling the solution to 0 ºC, methylmagnesium bromide (0.26 mL, 0.78 mmol, 3.0 M in Et_2O) was added dropwise. The reaction mixture was allowed to warm to room temperature and stirred for 58 hours, at which the Claisen rearrangement of the carbonyl addition intermediate was complete as monitored by TLC. After cooling to 0 °C, the reaction was quenched with saturated NH_4Cl (5 mL), and the resulting mixture was extracted with CH_2Cl_2 (3 x 5 mL). The organic layers were combined, dried over Na_2SO_4, filtered, and then concentrated under vacuum. The crude material was purified with column chromatography using 100% hexanes to 95:5 hexanes : EtOAc to yield **10f** in 29% yield (47 mg, 0.180 mmol) as a pale yellow oil. ^1H NMR analysis of the crude reaction mixture indicated > 20:1 dr.

Rf: 0.2 in 80:20 hexanes : EtOAc

^{1}H NMR: (500 MHz, $CDCl_3$) δ = 5.57 (s, 2H), 3.58 (br s, 1H), 2.88 (d, *J* = 15.2 Hz, 1H), 2.80 (d, *J* = 15.3 Hz, 1H), 2.15 – 2.01 (m, 2H), 1.86 – 1.75 (m, 3H), 1.66 – 1.56 (m, 1H), 1.43 (s, 3H), 1.26 (s, 3H).

^{13}C NMR: (125 MHz, $CDCl_3$) δ = 216.6, 128.2, 121.1, 75.9, 48.1, 48.0, 40.2, 38.1, 26.9, 24.8, 18.0.

IR: *f* (cm^{-1}) = 3470, 2932, 1701, 1623, 1456, 1374, 1164, 1040, 1012, 977, 899, 829, 549.

HRMS (ESI-TOF): *m/z* $[M\text{-}OH]^+$ = 243.0384 calculated for $C_{11}H_{16}BrO$; found 243.0387.

Compound (±)-12a

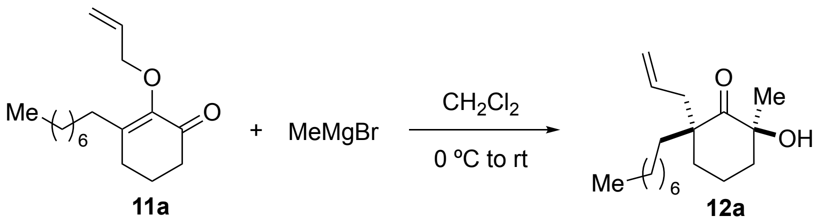

Procedure: Ketone **11a** (121 mg, 0.458 mmol) and CH_2Cl_2 (2.3 mL) were added into a round bottom flask. After cooling the solution to 0 ºC, methylmagnesium bromide (0.20 mL, 0.59 mmol, 3.0 M in Et_2O) was added dropwise. The reaction mixture was allowed to warm to room temperature and stirred for 43 hours, at which the Claisen rearrangement of the carbonyl addition intermediate was complete as monitored by TLC. After cooling to 0 °C, the reaction was quenched with saturated NH_4Cl (5 mL), and the resulting mixture was extracted with CH_2Cl_2 (3 x 5 mL). The organic layers were combined, dried over Na_2SO_4, filtered, and then concentrated under vacuum. The crude material was purified with column chromatography using 100% hexanes to 99:1 hexanes : EtOAc to yield **12a** in 65% yield (84 mg, 0.30 mmol) as a pale yellow oil. ^1H NMR analysis of the crude reaction mixture indicated > 20:1 dr.

Rf: 0.6 in 80:20 hexanes : EtOAc

^{1}H NMR: (400 MHz, $CDCl_3$) δ = 5.55 (ddt, *J* = 17.2, 10.3, 7.2 Hz, 1H), 5.10 – 4.99 (m, 2H), 3.65 (s, 1H), 2.48 (ddt, *J* = 14.4, 7.0, 1.3 Hz, 1H), 2.22 (ddt, *J* = 14.4, 7.6, 1.3 Hz, 1H), 2.02 (m, 1H), 1.83 – 1.68 (m, 5H), 1.62 – 1.47 (m, 2H), 1.37 (s, 3H), 1.25 (m, 10H), 1.20 – 1.12 (m, 2H), 0.87 (t, *J* = 6.8 Hz, 3H).

^{13}C NMR: (100 MHz, $CDCl_3$) δ = 217.0, 133.2, 118.3, 75.6, 50.8, 40.4, 39.5, 36.1, 35.2, 31.9, 30.3, 29.5, 29.3, 27.1, 23.6, 22.7, 18.1, 14.1.

IR: *f* (cm^{-1}) = 3489, 2926, 2855, 1699, 1639, 1461, 1370, 1157, 982, 913, 442.

HRMS (ESI-TOF): *m/z* $[M+H]^+$ = 281.2475 calculated for $C_{18}H_{33}O_2$; found 281.2473.

Compound (±)-12b

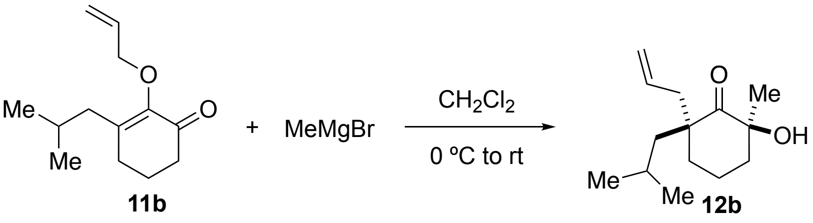

Procedure: Ketone **11b** (121 mg, 0.581 mmol) and CH_2Cl_2 (2.9 mL) were added into a round bottom flask. After cooling the solution to 0 ºC, methylmagnesium bromide (0.25 mL, 0.76 mmol, 3.0 M in Et_2O) was added dropwise. The reaction mixture was allowed to warm to room temperature and stirred for 20 hours, at which the Claisen rearrangement of the carbonyl addition intermediate was complete as monitored by TLC. After cooling to 0 °C, the reaction was quenched with saturated NH_4Cl (5 mL), and the resulting mixture was extracted with CH_2Cl_2 (3 x 5 mL). The organic layers were combined, dried over Na_2SO_4, filtered, and then concentrated under vacuum. The crude material was purified with column chromatography using 100% hexanes to 98:2 hexanes : EtOAc to yield **12b** in 64% yield (84 mg, 0.37 mmol) as a pale yellow oil. ^1H NMR analysis of the crude reaction mixture indicated > 20:1 dr.

Rf: 0.7 in 80:20 hexanes : EtOAc

^{1}H NMR: (400 MHz, $CDCl_3$) δ = 5.56 (ddt, *J* = 17.3, 10.2, 7.2 Hz, 1H), 5.09 – 5.05 (m, 1H), 5.01 (dq, *J* = 16.9, 1.6 Hz, 1H), 3.64 (s, 1H), 2.46 (ddt, *J* = 14.4, 7.0, 1.3 Hz, 1H), 2.20 (ddt, *J* = 14.5, 7.5, 1.3 Hz, 1H), 2.08 – 2.01 (m, 1H), 1.87 – 1.71 (m, 6H), 1.61 – 1.51 (m, 1H), 1.36 (s, 3H), 1.36 – 1.30 (m, 1H), 0.90 (d, *J* = 6.6 Hz, 3H), 0.84 (d, *J* = 6.7 Hz, 3H).

^{13}**C NMR:** (100 MHz, $CDCl_3$) δ = 217.1, 133.2, 118.4, 75.5, 51.3, 44.3, 41.8, 39.1, 34.3, 27.6, 25.1, 24.5, 24.4, 18.0.

IR: *f* (cm^{-1}) = 3498, 3077, 2952, 2870, 1698, 1639, 1463, 1367, 1261, 1200, 1153, 1068, 989.

HRMS (APCI-TOF): *m/z* $[M+H]^+$ = 225.1849 calculated for $C_{14}H_{25}O_2$; found 225.1842.

Compound (±)-12c

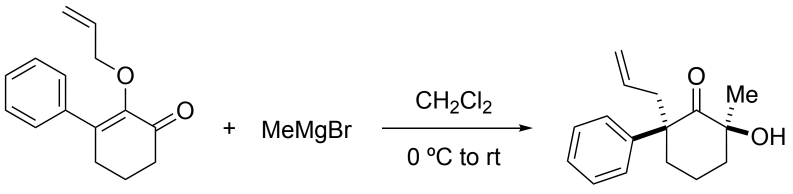

Procedure: Ketone **11c** (147 mg, 0.644 mmol) and CH_2Cl_2 (3.2 mL) were added into a round bottom flask. After cooling the solution to 0 ºC, methylmagnesium bromide (0.28 mL, 0.84 mmol, 3.0 M in Et_2O) was added dropwise. The reaction mixture was allowed to warm to room temperature and stirred for 22 hours, at which the Claisen rearrangement of the carbonyl addition intermediate was complete as monitored by TLC. After cooling to 0 °C, the reaction was quenched with saturated NH_4Cl (5 mL), and the resulting mixture was extracted with CH_2Cl_2 (3 x 5 mL). The organic layers were combined, dried over Na_2SO_4, filtered, and then concentrated under vacuum. The crude material was purified with column chromatography using 100% hexanes to 98:2 hexanes : EtOAc to yield **12c** in 78% yield (123 mg, 0.503 mmol) as a pale yellow oil. ^{1}H NMR analysis of the crude reaction mixture indicated > 20:1 dr.

Rf: 0.6 in 80:20 hexanes : EtOAc

^{1}**H NMR:** (400 MHz, $CDCl_3$) δ = 7.36 – 7.32 (m, 2H), 7.28 – 7.22 (m, 3H), 5.39 (ddt, *J* = 17.4, 10.4, 7.3 Hz, 1H), 4.98 – 4.89 (m, 2H), 2.62 – 2.50 (m, 3H), 2.07 – 1.96 (m, 2H), 1.88 – 1.77 (m, 2H), 1.77 – 1.66 (m, 2H), 1.32 (s, 3H).

^{13}C NMR: (100 MHz, $CDCl_3$) δ = 212.8, 134.0, 133.8, 128.7, 126.9, 126.2, 118.1, 76.2, 55.0, 45.5, 37.8, 31.8, 25.6, 16.8.

IR: *f* (cm^{-1}) = 3486, 3071, 2932, 2871, 1705, 1495, 1446, 1097, 1036, 984, 942, 858, 761, 533.

HRMS (APCI-TOF): *m/z* $[M+H]^+$ = 245.1536 calculated for $C_{16}H_{21}O_2$; found 245.1530.

Compound (±)-12d

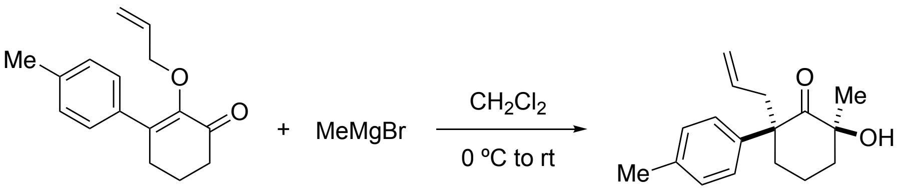

11d 12d

Procedure: Ketone **11d** (94 mg, 0.39 mmol) and CH_2Cl_2 (2.0 mL) were added into a round bottom flask. After cooling the solution to 0 ºC, methylmagnesium bromide (0.17 mL, 0.50 mmol, 3.0 M in Et_2O) was added dropwise. The reaction mixture was allowed to warm to room temperature and stirred for 22 hours, at which the Claisen rearrangement of the carbonyl addition intermediate was complete as monitored by TLC. After cooling to 0 °C, the reaction was quenched with saturated NH_4Cl (5 mL), and the resulting mixture was extracted with CH_2Cl_2 (3 x 5 mL). The organic layers were combined, dried over Na_2SO_4, filtered, and then concentrated under vacuum. The crude material was purified with column chromatography using 100% hexanes to 98:2 hexanes : EtOAc to yield **12d** in 72% yield (72 mg, 0.28 mmol) as a colorless oil. ^{1}H NMR analysis of the crude reaction mixture indicated > 20:1 dr.

Rf: 0.5 in 80:20 hexanes : EtOAc

^{1}H NMR: (500 MHz, $CDCl_3$) δ = 7.19 – 7.12 (m, 4H), 5.41 (ddt, *J* = 17.2, 10.3, 7.2 Hz, 1H), 4.97 – 4.90 (m, 2H), 3.79 (b s, 1H), 2.57 – 2.49 (m, 3H), 2.32 (s, 3H), 2.05 – 1.95 (m, 1H), 1.87 – 1.78 (m, 2H), 1.75 – 1.65 (m, 2H), 1.32 (s, 3H).

^{13}C NMR: (100 MHz, $CDCl_3$) δ = 212.8, 137.0, 136.7, 134.0, 129.5, 126.2, 117.9, 76.3, 54.8, 45.5, 37.9, 31.9, 25.6, 20.9, 16.9.

IR: *f* (cm^{-1}) = 3473, 2934, 1705, 1640, 1512, 1446, 1371, 1126, 914, 859, 812, 738, 672, 536.

HRMS (ESI-TOF): *m/z* $[M+H]^+$ = 259.1693 calculated for $C_{17}H_{23}O_2$; found 259.1693.

Compound (±)-12e

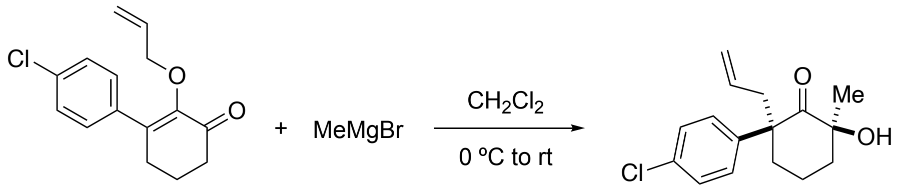

11e **12e**

Procedure: Ketone **11e** (201 mg, 0.765 mmol) and CH_2Cl_2 (4.0 mL) were added into a round bottom flask. After cooling the solution to 0 ºC, methylmagnesium bromide (0.30 mL, 0.90 mmol, 3.0 M in Et_2O) was added dropwise. The reaction mixture was allowed to warm to room temperature and stirred for 26 hours, at which the Claisen rearrangement of the carbonyl addition intermediate was complete as monitored by TLC. After cooling to 0 °C, the reaction was quenched with saturated NH_4Cl (5 mL), and the resulting mixture was extracted with CH_2Cl_2 (3 x 5 mL). The organic layers were combined, dried over Na_2SO_4, filtered, and then concentrated under vacuum. The crude material was purified with column chromatography using 100% hexanes to 95:5 hexanes : EtOAc to yield **12e** in 52% yield (112 mg, 0.402 mmol) as a colorless oil. ^{1}H NMR analysis of the crude reaction mixture indicated > 20:1 dr.

Rf: 0.5 in 80:20 hexanes : EtOAc

^{1}H NMR: (400 MHz, $CDCl_3$) δ = 7.30 (d, *J* = 8.6 Hz, 2H), 7.19 (d, *J* = 8.6 Hz, 2H), 5.44 – 5.28 (m, 1H), 5.00 – 4.89 (m, 2H), 2.57 – 2.46 (m, 3H), 2.03 – 1.91 (m, 1H), 1.89 – 1.77 (m, 2H), 1.77 – 1.67 (m, 2H), 1.33 (s, 3H).

^{13}C NMR: (100 MHz, $CDCl_3$) δ = 212.6, 138.8, 133.4, 132.7, 128.8, 127.7, 118.5, 76.0, 54.6, 45.3, 37.7, 31.9, 25.7, 16.8.

IR: *f* (cm^{-1}) = 3494, 3073, 2931, 1706, 1638, 1494, 1462, 1401, 1374, 1096, 1013, 942, 917, 822, 734, 534.

HRMS (ESI-TOF): *m/z* $[M+H]^+$ = 279.1146 calculated for $C_{16}H_{20}ClO_2$; found 279.1148.

Compound (±)-12f

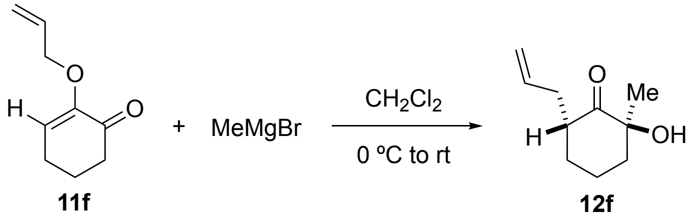

Procedure: Ketone **11f** (103 mg, 0.679 mmol) and CH_2Cl_2 (3.4 mL) were added into a round bottom flask. After cooling the solution to 0 ºC, methylmagnesium bromide (0.24 mL, 0.71 mmol, 3.0 M in Et_2O) was added dropwise. The reaction mixture was allowed to warm slowly to room temperature and stirred for 14 hours, at which the Claisen rearrangement of the carbonyl addition intermediate was complete as monitored by TLC. After cooling to 0 °C, the reaction was quenched with saturated NH_4Cl (5 mL), and the resulting mixture was extracted with CH_2Cl_2 (3 x 5 mL). The organic layers were combined, dried over Na_2SO_4, filtered, and then concentrated under vacuum. The crude material was purified with column chromatography using 100% hexanes to 95:5 hexanes : EtOAc to yield **12f** in 35% yield (41 mg, 0.24 mmol) as a colorless oil. ^{1}H NMR analysis of the crude reaction mixture indicated > 20:1 dr.

Rf: 0.5 in 80:20 hexanes : EtOAc

^{1}H NMR: (400 MHz, $CDCl_3$) δ = 5.76 (dddd, *J* = 16.2, 12.7, 7.6, 6.3 Hz, 1H), 5.06 – 4.98 (m, 2H), 3.01 (dq, *J* = 7.7, 5.8 Hz, 1H), 2.48 (dtt, *J* = 14.4, 6.1, 1.3 Hz, 1H), 2.10 – 1.88 (m, 5H), 1.68 – 1.60 (m, 2H), 1.31 (s, 3H).

^{13}C NMR: (100 MHz, $CDCl_3$) δ = 213.3, 136.3, 116.3, 76.1, 45.9, 41.7, 33.9, 33.4, 24.6, 20.2.

IR: *f* (cm^{-1}) = 3456, 3077, 2977, 2932, 2855, 1709, 1448, 1375, 1128, 913.

HRMS (ESI-TOF): *m/z* $[M+H]^+$ = 169.1223 calculated for $C_{10}H_{17}O_2$; found 169.1224.

SCREENING OF CHIRAL LIGANDS AND REACTION OPTIMIZATION

Table SI-1 summarizes our screening studies. Our initial experimental setup involved the mixing of 1.0 equiv of chiral ligand and 3.1 equiv of methylmagnesium bromide to create the chiral organomagnesium reagent at -78 °C in the presence of 2-allyloxyenone **6a**. Upon complete consumption of the starting material, warming of the reaction mixture to room temperature then prompted the Claisen rearrangement.

Table SI-1. Screening of Chiral Ligands and Reaction Optimization

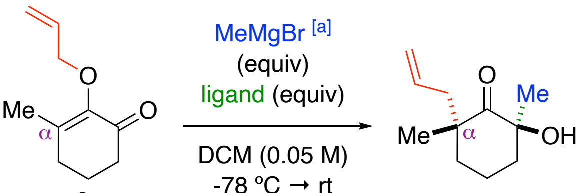

6a → **(-)-7a**

entry	MeMgBr	ligand	equiv	er [a]
1	3.1	**13a**	1.0	66:34
2	3.1	**13b**	1.0	70:30
3	3.1	**13c**	1.0	52:48
4	3.1	**13d**	1.0	52:48
5	3.1	**13e**	1.0	83:17
6	3.1	**13f**	1.0	82:18
7	3.1	**13g**	1.0	94:6
8	3.1	**14**	1.0	93:7
9	3.5	**14**	1.2	97:3
10	3.9	**14**	1.4	98:2

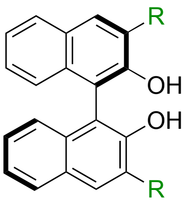

13a: R = H
13b: R = neopentyl
13c: R = benzhydryl
13d: R = 2,4,6-(*i*-Pr)$_3$phenyl
13e: R = 9-phenanthryl
13f: R = 1-pyrenyl
13g: R = 9-anthracyl

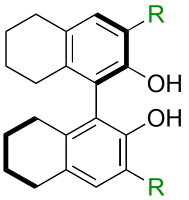

14: R = 9-anthracyl

[a] Enantiomeric ratio was determined by chiral HPLC of the *p*-nitrobenzoate ester derivative of product **(-)-7a**.

Evaluation on a series of aliphatic and aromatic substituents at the 3,3'-positions (entries 1-7) revealed that 9-anthracyl BINOL ligand **13g** provided the strongest enantioinduction, in which ketone **(-)-7a** was isolated with 94:6 er. We also explored the octahydro-BINOL variant **14** due to a much simpler synthetic preparation to this class of chiral ligands. A similar level of enantioselectivity was noted, *i.e.* 93:7 er. As shown in entries 8-10, increasing the molar amount of the chiral ligand and the corresponding Grignard reagent further improved enantioselectivity. In fact, the use of 3.9 equiv of methylmagnesium bromide and 1.4 equiv of BINOL derivative **14** furnished ketone **(-)-7a** with 98:2 er as a single diastereomer.

EXPERIMENTAL DATA FOR SCHEME 3

Compound (-)-7a

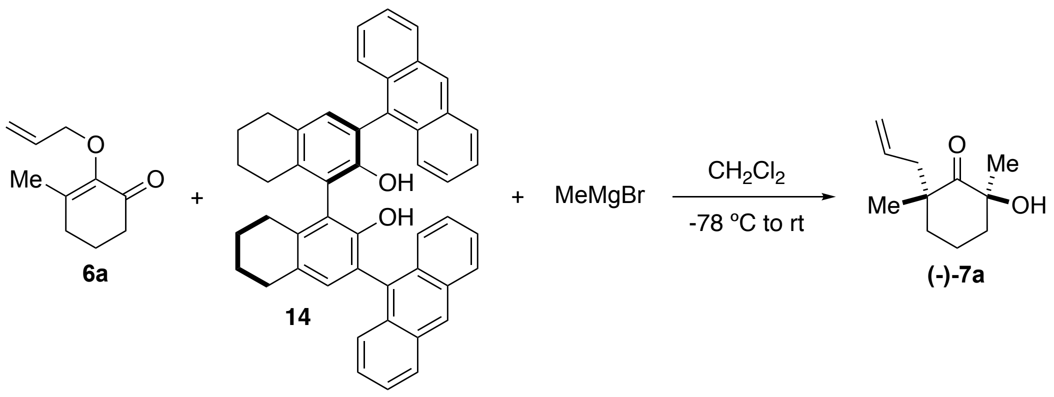

Procedure: Ligand **14** (272 mg, 0.421 mmol) and ketone **6a** (50 mg, 0.30 mmol) were dissolved in CH_2Cl_2 (6.0 mL) in a round bottom flask. After cooling the solution to -78 °C, methylmagnesium bromide (0.39 mL, 1.17 mmol, 3.0 M in Et_2O) was added dropwise. After stirring at -78 °C until the starting material was fully consumed as monitored by TLC, the reaction mixture was warmed to room temperature and stirred until the Claisen rearrangement of the carbonyl addition intermediate was complete as monitored by TLC for a total reaction time of 19 hours. After cooling to 0 °C, the reaction was quenched with a saturated NH_4Cl solution (8 mL) and diluted with DI water (8 mL). The aqueous layer was extracted with CH_2Cl_2 (3 x 10 mL), and the combined organic layers were washed with brine, dried over Na_2SO_4, and concentrated under vacuum. The crude material was purified by column chromatography using 100% hexanes to 50:50 hexanes : CH_2Cl_2 to afford compound **(-)-7a** in 77% yield (42 mg, 0.23 mmol) as a colorless oil. ^{1}H NMR analysis of the crude reaction mixture indicated > 20:1 dr.

Rf: 0.4 in 50:50 hexanes : CH_2Cl_2 then 80:20 hexanes : EtOAc

^{1}H NMR: (500 MHz, $CDCl_3$) δ = 5.62 – 5.54 (m, 1H), 5.09 – 5.03 (m, 2H), 3.88 (b s, 1H), 2.53 (dd, J = 14.2, 6.9 Hz, 1H), 2.20 – 2.15 (m, 1H), 2.10 – 2.03 (m, 1H), 1.91 – 1.69 (m, 4H), 1.64 – 1.53 (m, 1H), 1.39 (s, 3H), 1.10 (s, 3H).

^{13}C NMR: (125 MHz, $CDCl_3$) δ = 217.6, 133.0, 118.7, 75.8, 47.8, 42.1, 40.5, 38.9, 26.8, 24.2, 18.4.

IR: f (cm^{-1}) = 3479, 3076, 2934, 2871, 1699, 1640, 1460, 1373, 1261, 1158, 1012, 993, 872, 799.

HRMS (ESI-TOF): m/z $[M+H]^+$ = 183.1379 calculated for $C_{11}H_{19}O_2$; found 183.1380.

Specific Rotation: $[\alpha]_{25}^{D}$ = -5.5 (c = 1.00 in $CHCl_3$)

HPLC ((-)-7a)-BzNO$_2$: OD-H, hexane/isopropanol = 80/20, flow rate = 1.0 mL/min, λ = 254 nm, t_R = 6.6 min (major), 11.7 min (minor).

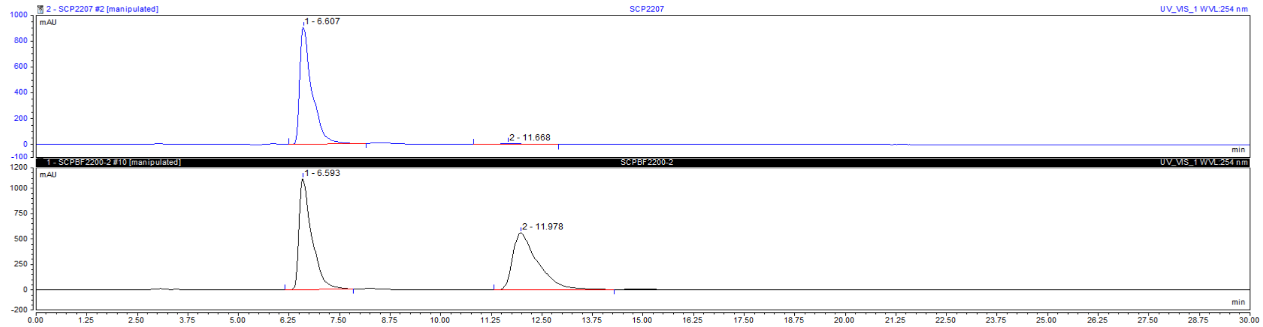

Retention Time (min)	Relative Area (%)	Area (mAU*min)	Height (mAU)
6.607	98.12	312.6254	903.01
11.668	1.88	5.98	9.82

Compound ((-)-7a)-BzNO₂:

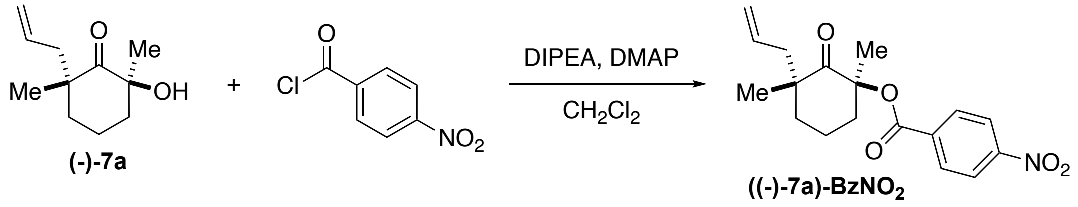

Procedure: Alcohol **(-)-7a** (21 mg, 0.12 mmol) was dissolved in CH_2Cl_2 (0.6 mL). *N,N*-Diisopropylethylamine (60 µL, 0.35 mmol) was then added to the solution, followed by 4-nitrobenzoyl chloride (75 mg, 0.40 mmol) and 4-(dimethylamino)pyridine (14 mg, 0.12 mmol). The reaction mixture was then stirred at room temperature until complete consumption of the the starting material as monitored by TLC. The crude reaction mixture was concentrated under vacuum and then purified by column chromatography using 100% hexanes to 97:3 hexanes : EtOAc to afford compound **((-)-7a)-BzNO₂** in 94% yield (36 mg, 0.11 mmol) as a white solid.

Rf: 0.6 in 80:20 hexanes : EtOAc

^{1}H NMR: (500 MHz, $CDCl_3$) δ = 8.27 – 8.22 (m, 2H), 8.18 – 8.16 (m, 2H), 5.76 – 5.67 (m, 1H), 5.11 – 5.03 (m, 2H), 2.58 – 2.52 (m, 1H), 2.34 – 2.29 (m, 1H), 2.24 – 2.19 (m, 1H), 2.03 – 1.96 (m, 1H), 1.91 – 1.75 (m, 4H), 1.61 (s, 3H), 1.20 (s, 3H).

^{13}C NMR: (125 MHz, $CDCl_3$) δ = 210.5, 163.5, 150.5, 135.7, 133.1, 130.8, 123.4, 118.6, 118.6, 83.4, 48.5, 42.6, 37.4, 35.9, 25.0, 24.9, 23.6, 23.6, 18.3.

IR: *ƒ* (cm^{-1}) = 2935, 1708, 1607, 1527, 1462, 1375, 1349, 1320, 1285, 1211, 1167, 1115, 1103, 1077, 1006, 863, 720.

HRMS (ESI-TOF): *m/z* $[M+H]^+$ = 332.1492 calculated for $C_{18}H_{22}NO_5$; found 332.1503.

Compound (-)-8a

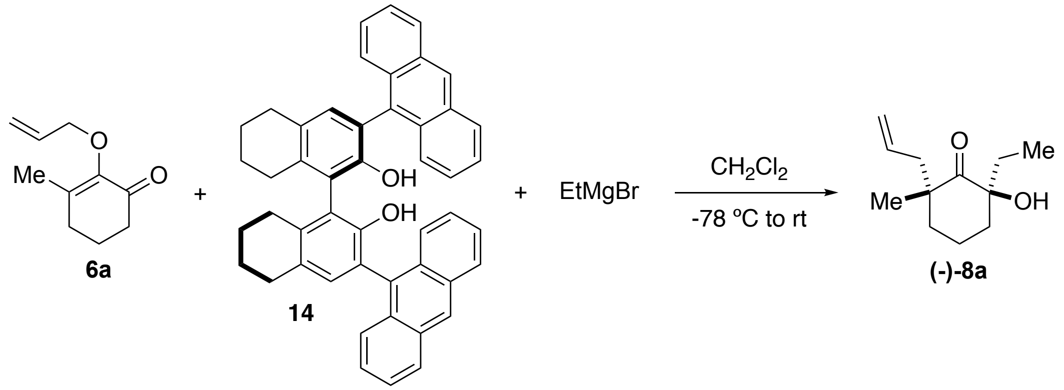

Procedure: Ligand **14** (864 mg, 1.34 mmol) and ketone **6a** (159 mg, 0.957 mmol) were dissolved in CH_2Cl_2 (19 mL) in a round bottom flask. After cooling the solution to -78 °C, ethylmagnesium bromide (1.24 mL, 3.73 mmol, 3.0 M in Et_2O) was added dropwise. After stirring at -78 °C until the starting material was fully consumed as monitored by TLC, the reaction mixture was warmed to room temperature and stirred until the Claisen rearrangement of the carbonyl addition intermediate was complete as monitored by TLC for a total reaction time of 24 hours. After cooling to 0 °C, the reaction was quenched with a saturated NH_4Cl solution (10 mL) and diluted with DI water (10 mL). The aqueous layer was extracted with CH_2Cl_2 (3 x 15 mL), and the combined organic layers were washed with brine, dried over Na_2SO_4, and concentrated under vacuum. The crude material was purified by column chromatography using 100% hexanes to 50:50 hexanes : CH_2Cl_2 to afford compound **(-)-8a** in 70% yield (131 mg, 0.667 mmol) as a pale yellow oil. ^{1}H NMR analysis of the crude reaction mixture indicated > 20:1 dr.

Rf: 0.5 in 50:50 hexanes : CH_2Cl_2 then 80:20 hexanes : EtOAc

^{1}H NMR: (400 MHz, $CDCl_3$) δ = 5.63 (ddt, *J* = 17.0, 10.2, 7.2 Hz, 1H), 5.12 – 5.03 (m, 2H), 3.82 (s, 1H), 2.37 (dd, *J* = 14.0, 6.8 Hz, 1H), 2.28 (dd, *J* = 14.0, 7.7 Hz, 1H), 2.19 – 2.12 (m, 1H), 1.90 – 1.87 (m, 1H), 1.82 – 1.65 (m, 5H), 1.58 – 1.48 (m, 1H), 1.09 (s, 3H), 0.84 (t, *J* = 7.4 Hz, 3H).

^{13}C NMR: (100 MHz, $CDCl_3$) δ = 217.8, 132.8, 118.8, 78.4, 47.7, 41.2, 38.2, 38.1, 31.6, 24.3, 18.1, 7.4.

IR: *f* (cm^{-1}) = 3495, 3076, 2936, 2875, 1695, 1639, 1459, 1377, 1262, 1149, 1015, 981, 918, 885.

HRMS (ESI-TOF): *m/z* $[M+H]^+$ = 197.1536 calculated for $C_{12}H_{21}O_2$; found 197.1541.

Specific Rotation: $[\alpha]_{25}^{D}$ = -6.2 (c = 1.00 in $CHCl_3$)

HPLC ((-)-8a)-BzNO$_2$: OD-H, hexane/isopropanol = 98/2, flow rate = 1.0 mL/min, λ = 254 nm, t_R = 13.0 min (major), 17.3 min (minor).

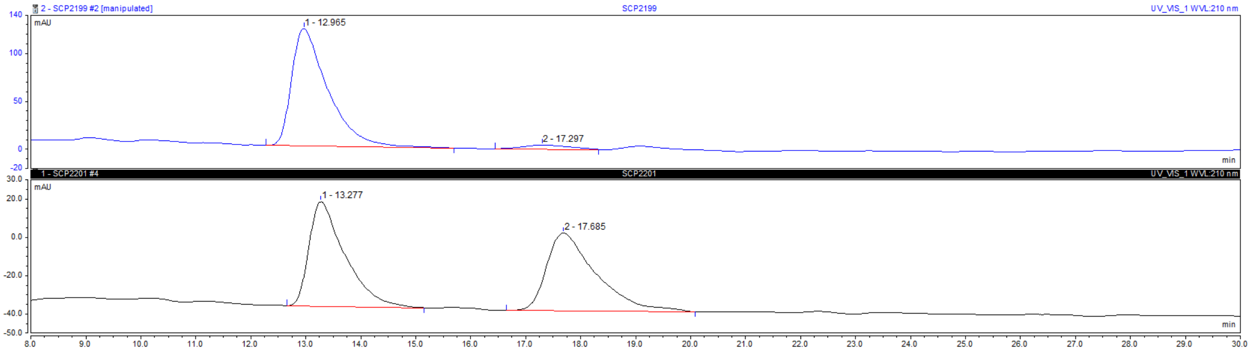

Retention Time (min)	Relative Area (%)	Area (mAU*min)	Height (mAU)
12.965	96.01	92.9509	122.04
17.297	3.99	3.8679	3.99

Compound ((-)-8a)-BzNO$_2$

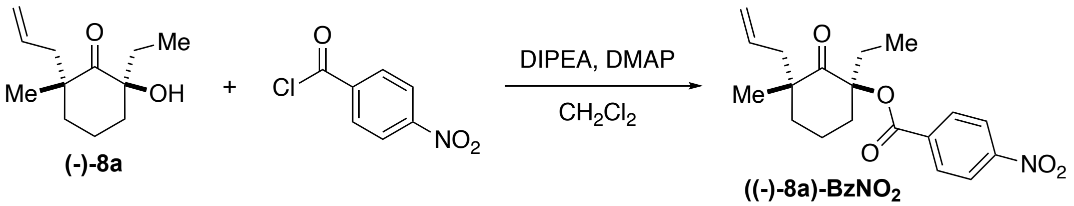

Procedure: Alcohol **(-)-8a** (23 mg, 0.12 mmol) was dissolved in CH_2Cl_2 (0.7 mL). *N*,*N*-Diisopropylethylamine (0.10 mL, 0.59 mmol) was then added to the solution, followed by 4-

nitrobenzoyl chloride (70 mg, 0.38 mmol) and 4-(dimethylamino)pyridine (14 mg, 0.12 mmol). The reaction mixture was then stirred at room temperature until complete consumption of the the starting material as monitored by TLC. The crude reaction mixture was concentrated under vacuum and then purified by column chromatography using 100% hexanes to 97:3 hexanes : EtOAc to afford compound **((-)-8a)-BzNO$_2$** in 91% yield (37 mg, 0.11 mmol) as a pale yellow oil.

Rf: 0.6 in 80:20 hexanes : EtOAc

^{1}H NMR: (500 MHz, $CDCl_3$) δ = 8.27 (d, *J* = 8.7 Hz, 2H), 8.16 (d, *J* = 8.7 Hz, 2H), 5.79 – 5.66 (m, 1H), 5.12 – 5.02 (m, 2H), 2.54 – 2.45 (m, 1H), 2.30 – 2.21 (m, 2H), 2.13 – 2.09 (m, 1H), 2.08 – 2.00 (m, 1H), 1.98 – 1.91 (m, 1H), 1.88 – 1.75 (m, 4H), 1.20 (s, 3H), 1.06 (t, *J* = 7.4 Hz, 3H).

^{13}C NMR: (125 MHz, $CDCl_3$) δ = 210.0, 163.4, 150.5, 135.9, 133.0, 130.7, 123.5, 118.6, 85.5, 48.5, 42.2, 35.5, 34.0, 30.1, 24.0, 18.2, 7.7.

IR: *f* (cm^{-1}) = 2935, 1707, 1607, 1527, 1460, 1348, 1320, 1282, 1116, 1103, 1079, 1014, 872, 720.

HRMS (ESI-TOF): *m/z* $[M+H]^+$ = 346.1649 calculated for $C_{19}H_{24}O_5$; found 346.1660.

Compound (-)-8b

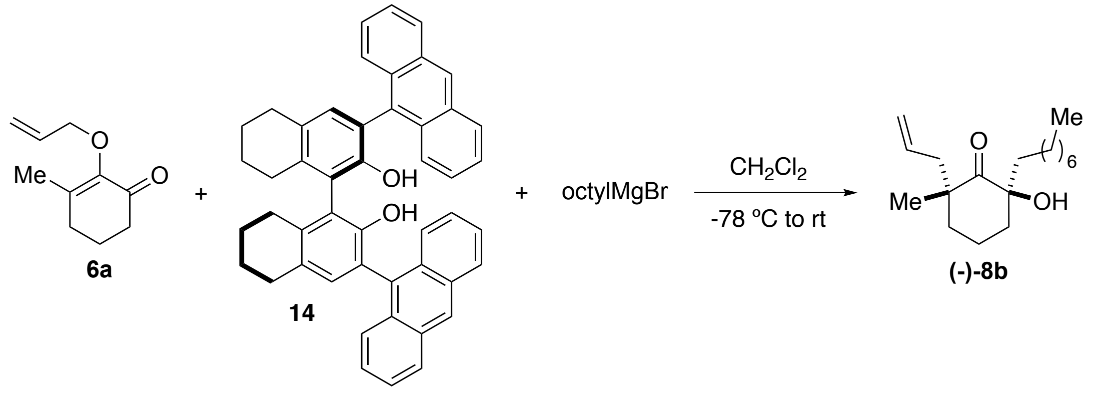

Procedure: Ligand **14** (829 mg, 1.28 mmol) and ketone **6a** (152 mg, 0.914 mmol) were dissolved in CH_2Cl_2 (18 mL) in a round bottom flask. After cooling the solution to -78 °C, octylmagnesium

bromide (1.79 mL, 3.57 mmol, 2.0 M in Et_2O) was added dropwise. After stirring at -78 °C until the starting material was fully consumed as monitored by TLC, the reaction mixture was warmed to room temperature and stirred until the Claisen rearrangement of the carbonyl addition intermediate was complete as monitored by TLC for a total reaction time of 22 hours. After cooling to 0 °C, the reaction was quenched with a saturated NH_4Cl solution (10 mL) and diluted with DI water (10 mL). The aqueous layer was extracted with CH_2Cl_2 (3 x 15 mL), and the combined organic layers were washed with brine, dried over Na_2SO_4, and concentrated under vacuum. The crude material was purified by column chromatography using 100% hexanes to 50:50 hexanes : CH_2Cl_2 to afford compound **(-)-8b** in 67% yield (172 mg, 0.613 mmol) as a colorless oil. ^{1}H NMR analysis of the crude reaction mixture indicated > 20:1 dr.

Rf: 0.5 in 50:50 hexanes : CH_2Cl_2 then 80:20 hexanes : EtOAc

^{1}H NMR: (400 MHz, $CDCl_3$) δ = 5.63 (ddt, *J* = 17.0, 10.2, 7.3 Hz, 1H), 5.13 – 5.02 (m, 2H), 3.84 (s, 1H), 2.37 (dd, *J* = 14.0, 6.8 Hz, 1H), 2.28 (dd, *J* = 14.0, 7.7 Hz, 1H), 2.19 – 2.11 (m, 1H), 1.91 (dt, *J* = 13.1, 3.5 Hz, 1H), 1.86 – 1.74 (m, 1H), 1.73 – 1.61 (m, 4H), 1.58 – 1.47 (m, 1H), 1.45 - 1.33 (m, 1H), 1.31 – 1.16 (m, 10H), 1.13 – 1.09 (m, 1H), 1.08 (s, 3H), 0.86 (t, *J* = 6.8 Hz, 3H).

^{13}C NMR: (125 MHz, $CDCl_3$) δ = 217.8, 132.8, 118.7, 78.3, 47.7, 41.2, 39.0, 38.6, 38.1, 31.8, 29.8, 29.4, 29.2, 24.3, 22.9, 22.6, 18.1, 14.1.

IR: *f* (cm^{-1}) = 3497, 3077, 2925, 2854, 1695, 1639, 1460, 1378, 1260, 1171, 1094, 1004, 915, 801, 561.

HRMS (ESI-TOF): *m/z* $[M+H]^+$ = 281.2475 calculated for $C_{18}H_{33}O_2$; found 281.2485.

Specific Rotation: $[\alpha]_{25}^{D}$ = -8.0 (c = 1.00 in $CHCl_3$)

HPLC ((-)-8b)-BzNO$_2$: OD-H, hexane/isopropanol = 95/5, flow rate = 1.0 mL/min, λ = 254 nm, t_R = 5.5 min (minor), 7.7 min (major).

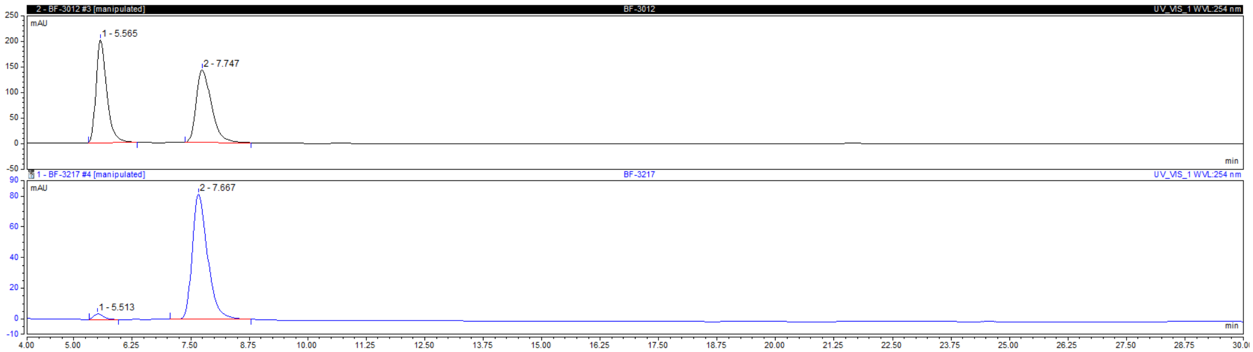

Retention Time (min)	Relative Area (%)	Area (mAU*min)	Height (mAU)
5.513	2.78	0.8805	3.59
7.667	97.22	30.7840	81.10

Compound ((-)-8b)-BzNO₂

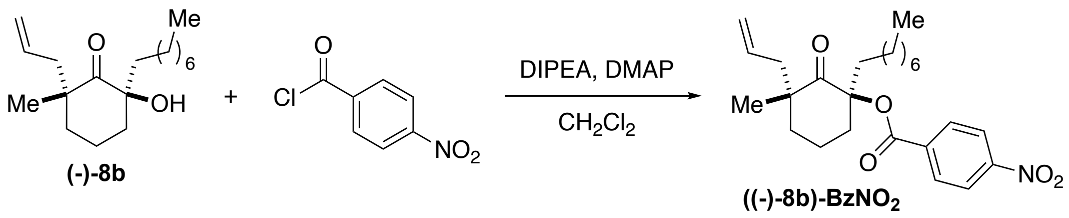

Procedure: Alcohol **(-)-8b** (21 mg, 0.075 mmol) was dissolved in CH_2Cl_2 (0.4 mL). *N*,*N*-Diisopropylethylamine (70 µL, 0.40 mmol) was then added to the solution, followed by 4-nitrobenzoyl chloride (45 mg, 0.24 mmol) and 4-(dimethylamino)pyridine (9 mg, 0.075 mmol). The reaction mixture was then stirred at room temperature until complete consumption of the starting material as monitored by TLC. The crude reaction mixture was concentrated under vacuum and then purified by column chromatography using 100% hexanes to 97:3 hexanes : EtOAc to afford compound **((-)-8b)-BzNO₂** in 89% yield (28 mg, 0.063 mmol) as a colorless oil.

Rf: 0.8 in 80:20 hexanes : EtOAc

^{1}**H NMR:** (500 MHz, $CDCl_3$) δ = 8.27 (d, J = 8.8 Hz, 2H), 8.15 (d, J = 8.8 Hz, 2H), 5.73 (ddt, J = 17.2, 10.0, 7.3 Hz, 1H), 5.13 – 5.04 (m, 2H), 2.55 – 2.47 (m, 1H), 2.31 – 2.21 (m, 2H), 2.11 (d, J = 13.5 Hz, 1H), 1.99 – 1.91 (m, 1H), 1.90 – 1.74 (m, 5H), 1.60 – 1.51 (m, 1H), 1.44 – 1.27 (m, 11H), 1.20 (s, 3H), 0.89 (t, J = 6.9 Hz, 3H).

^{13}**C NMR:** (125 MHz, $CDCl_3$) δ = 210.1, 163.5, 150.5, 136.0, 133.0, 130.7, 123.5, 118.6, 85.6, 48.5, 42.3, 37.1, 35.5, 34.4, 31.8, 29.8, 29.4, 29.2, 24.0, 23.1, 22.7, 18.3, 14.10.

IR: f (cm^{-1}) = 2932, 1709, 1606, 1527, 1495, 1454, 1374, 1349, 1320, 1287, 1102, 1010, 864, 720.

HRMS (ESI-TOF): m/z $[M+Na]^+$ =452.2407 calculated for $C_{25}H_{35}NO_5Na$; found 452.2423.

Compound (-)-8c

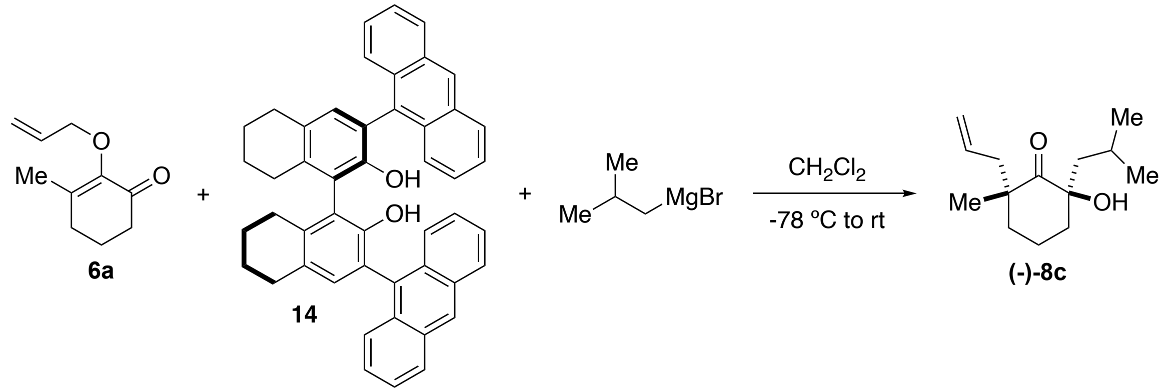

Procedure: Ligand **14** (855 mg, 1.32 mmol) and ketone **6a** (157 mg, 0.944 mmol) were dissolved in CH_2Cl_2 (19 mL) in a round bottom flask. After cooling the solution to -78 °C, isobutylmagnesium bromide (1.84 mL, 3.68 mmol, 2.0 M in Et_2O) was added dropwise. After stirring at -78 °C until the starting material was fully consumed as monitored by TLC, the reaction mixture was warmed to room temperature and stirred until the Claisen rearrangement of the carbonyl addition intermediate was complete as monitored by TLC for a total reaction time of 24 hours. After cooling to 0 °C, the reaction was quenched with a saturated NH_4Cl solution (10 mL)

and diluted with DI water (10 mL). The aqueous layer was extracted with CH_2Cl_2 (3 x 15 mL), and the combined organic layers were washed with brine, dried over Na_2SO_4, and concentrated under vacuum. The crude material was purified by column chromatography using 100% hexanes to 50:50 hexanes : CH_2Cl_2 to afford compound **(-)-8c** in 63% yield (134 mg, 0.597 mmol) as a pale yellow oil. ^{1}H NMR analysis of the crude reaction mixture indicated > 20:1 dr.

Rf: 0.5 in 50:50 hexanes : CH_2Cl_2 then 80:20 hexanes : EtOAc

^{1}H NMR: (400 MHz, $CDCl_3$) δ = 5.63 (ddt, *J* = 17.2, 10.2, 7.2 Hz, 1H), 5.13 – 5.03 (m, 2H), 3.71 (s, 1H), 2.36 (dd, *J* = 13.9, 6.7 Hz, 1H), 2.27 (dd, *J* = 13.9, 7.8 Hz, 1H), 2.23 – 2.15 (m, 1H), 1.95 – 1.86 (m, 1H), 1.83 – 1.61 (m, 5H), 1.58 – 1.48 (m, 2H), 1.09 (s, 3H), 0.98 (d, *J* = 6.5 Hz, 3H), 0.91 (d, *J* = 6.5 Hz, 3H).

^{13}C NMR: (100 MHz, $CDCl_3$) δ =217.9, 132.9, 118.8, 78.9, 47.7, 46.5, 41.6, 38.4, 37.8, 24.7, 24.6, 24.4, 24.2, 18.0.

IR: *f* (cm^{-1}) = 3494, 3078, 2933, 2869, 1694, 1459, 1377, 1261, 1167, 1149, 1090, 1055, 916, 737, 427.

HRMS (ESI-TOF): *m/z* $[M+H]^+$ = 225.1849 calculated for $C_{14}H_{25}O_2$; found 225.1857.

Specific Rotation: $[\alpha]_{25}^{D}$ = -8.1 (c = 1.00 in $CHCl_3$)

HPLC ((-)-8c)-BzNO$_2$: OD-H, hexane/isopropanol = 95/5, flow rate = 1.0 mL/min, λ = 254 nm, t_R = 5.1 min (major), 6.7 min (minor).

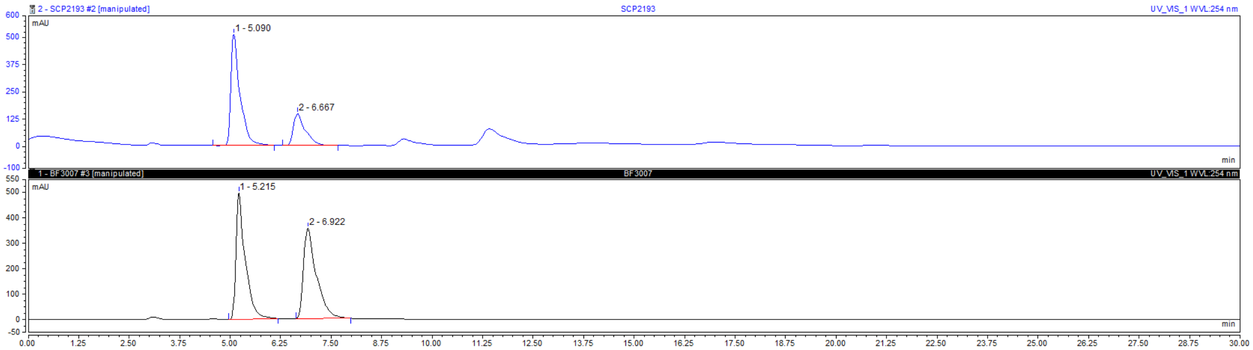

Retention Time (min)	Relative Area (%)	Area (mAU*min)	Height (mAU)
5.090	72.50	130.5365	507.22
6.667	27.50	49.5088	145.47

Compound ((-)-8c)-BzNO$_2$

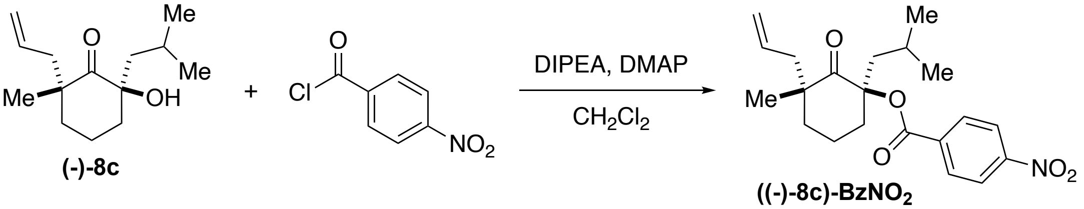

Procedure: Alcohol **(-)-8c** (22 mg, 0.098 mmol) was dissolved in CH_2Cl_2 (0.5 mL). *N*,*N*-Diisopropylethylamine (80 μL, 0.46 mmol) was then added to the solution, followed by 4-nitrobenzoyl chloride (57 mg, 0.31 mmol) and 4-(dimethylamino)pyridine (12 mg, 0.098 mmol). The reaction mixture was then stirred at room temperature until complete consumption of the starting material as monitored by TLC. The crude reaction mixture was concentrated under vacuum and then purified by column chromatography using 100% hexanes to 97:3 hexanes : EtOAc to afford compound **((-)-8c)-BzNO$_2$** in 82% yield (30 mg, 0.080 mmol) as a colorless oil.

Rf: 0.8 in 80:20 hexanes : EtOAc

^{1}H NMR: (400 MHz, $CDCl_3$) δ = 8.29 – 8.24 (m, 2H), 8.18 – 8.12 (m, 2H), 5.74 (ddt, *J* = 17.3, 10.1, 7.3 Hz, 1H), 5.15 – 5.02 (m, 2H), 2.51 (td, *J* = 12.9, 4.8 Hz, 1H), 2.25 (d, *J* = 7.3 Hz, 2H), 2.14 (dt, *J* = 16.2, 2.7 Hz, 1H), 1.93 – 1.74 (m, 7H), 1.20 (s, 3H), 1.09 (d, *J* = 6.2 Hz, 3H), 1.04 (d, *J* = 6.3 Hz, 3H).

^{13}C NMR: (100 MHz, $CDCl_3$) δ = 210.0, 163.5, 150.5, 135.9, 132.9, 130.7, 123.5, 118.7, 86.2, 48.4, 45.0, 42.3, 35.2, 34.5, 24.4, 24.4, 24.2, 24.1, 18.3.

IR: *ƒ* (cm^{-1}) = 2932, 2871, 1708, 1607, 1528, 1463, 1349, 1319, 1286, 1164, 1115, 1102, 1015, 872, 720.

HRMS (ESI-TOF): *m/z* $[M+Na]^+$ = 396.1781 calculated for $C_{21}H_{27}NO_5Na$; found for 396.1787.

Compound (-)-8g

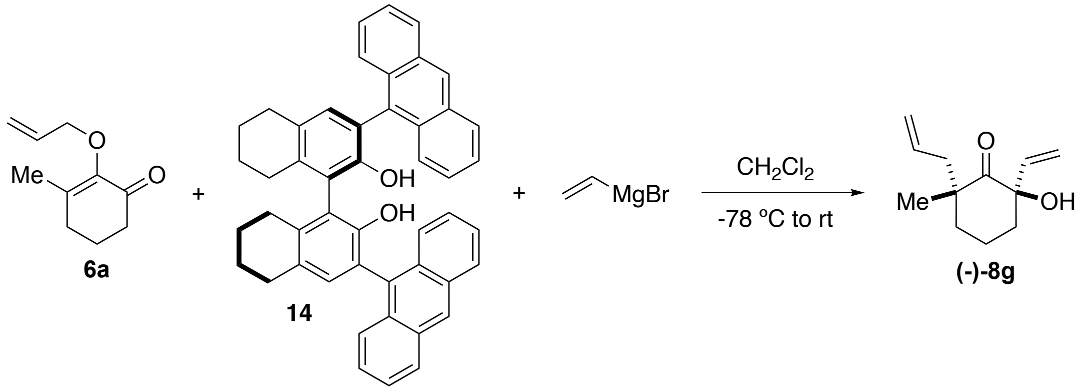

Procedure: Ligand **14** (830 mg, 1.28 mmol) and ketone **6a** (152 mg, 0.914 mmol) were dissolved in CH_2Cl_2 (18.2 mL) in a round bottom flask. After cooling the solution to -78 °C, vinylmagnesium bromide (7.80 mL, 3.51 mmol, 0.45 M in THF) was added dropwise. After stirring at -78 °C until the starting material was fully consumed as monitored by TLC, the reaction mixture was warmed to room temperature and stirred until the Claisen rearrangement of the carbonyl addition intermediate was complete as monitored by TLC for a total reaction time of 24 hours. After cooling to 0 °C, the reaction was quenched with a saturated NH_4Cl solution (10 mL) and diluted with DI water (10 mL). The aqueous layer was extracted with CH_2Cl_2 (3 x 15 mL), and the combined organic layers were washed with brine, dried over Na_2SO_4, and concentrated under vacuum. The crude material was purified by column chromatography using 100% hexanes to 50:50 hexanes : CH_2Cl_2 to afford compound **(-)-8g** in 62% yield (110 mg, 0.566 mmol) as a pale yellow oil. ^1H NMR analysis of the crude reaction mixture indicated > 20:1 dr.

Rf: 0.5 in 90:10 hexanes : EtOAc

^{1}H NMR: (500 MHz, $CDCl_3$) δ = 6.05 (dd, *J* = 17.1, 10.5 Hz, 1H), 5.54-5.41 (m, 2H), 5.26 (d, *J* = 10.6 Hz, 1H), 5.08 – 5.01 (m, 2H), 2.50 (dd, *J* = 14.2, 6.3 Hz, 1H), 2.38– 2.28 (m, 1H), 2.14 (dd, *J* = 14.1, 8.2 Hz, 1H), 1.96 – 1.85 (m, 2H), 1.79 – 1.69 (m, 2H), 1.64 – 1.57 (m, 1H), 1.08 (s, 3H).

^{13}C NMR: (125 MHz, $CDCl_3$) δ = 214.8, 137.8, 132.7, 118.7, 116.2, 78.0, 48.0, 41.5, 38.9, 38.6, 23.7, 18.0.

IR: *f* (cm^{-1}) = 3482, 2935, 1699, 1457, 1379, 1239, 996, 25, 755.

HRMS (ESI-TOF): *m/z* $[M+H]^+$ =195.1380 calculated for $C_{12}H_{19}O_2$; found for 195.1376.

Specific Rotation: $[\alpha]_{25}^{D}$ = -10.1 (c = 1.00 in $CHCl_3$).

HPLC ((-)-8g)-BzNO$_2$: OD-H, hexane/isopropanol = 90/10, flow rate = 1.0 mL/min, λ = 254 nm, t_R = 4.9 min (major), 6.9 min (minor).

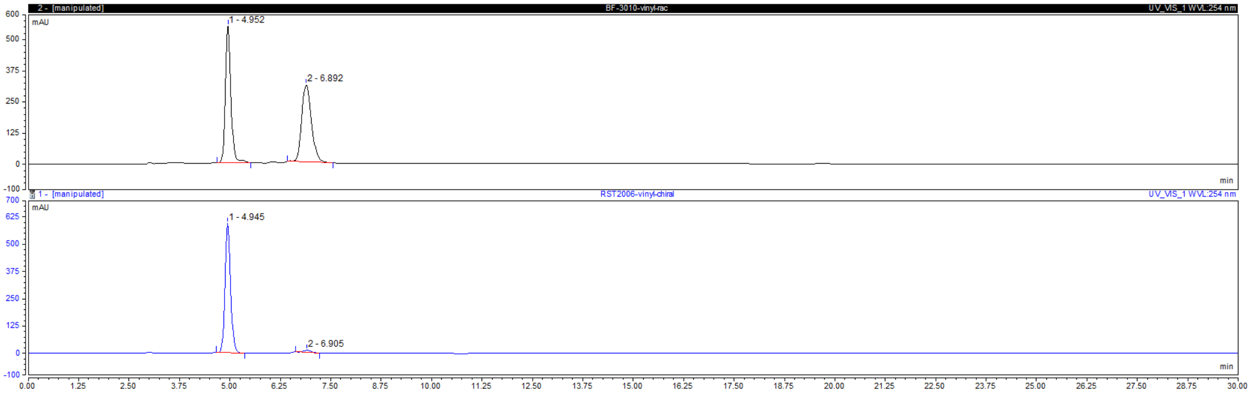

Retention Time (min)	Relative Area (%)	Area (mAU*min)	Height (mAU)
4.945	97.66	97.0541	591.57
6.905	2.34	2.3304	10.02

Compound ((-)-8g)-BzNO₂

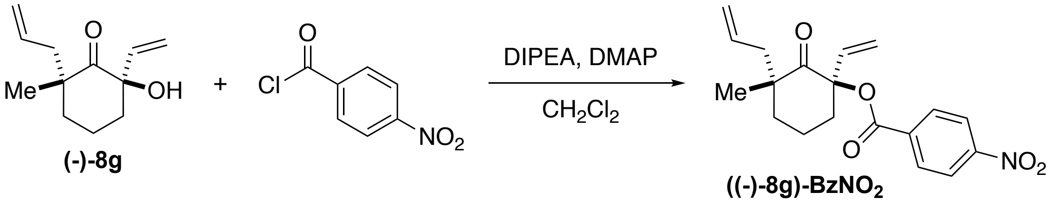

Procedure: Alcohol **(-)-8g** (50 mg, 0.26 mmol) was dissolved in CH_2Cl_2 (1.4 mL). *N*,*N*-Diisopropylethylamine (0.22 mL, 1.29 mmol) was then added to the solution, followed by 4-nitrobenzoyl chloride (152 mg, 0.82 mmol) and 4-(dimethylamino)pyridine (31 mg, 0.26 mmol). The reaction mixture was then stirred at room temperature until complete consumption of the starting material as monitored by TLC. The crude reaction mixture was concentrated under vacuum and then purified by column chromatography using 100% hexanes to 96:4 hexanes : EtOAc to afford compound **((-)-8g)-BzNO$_2$** in 84% yield (74 mg, 0.22 mmol) as a colorless oil.

Rf: 0.7 in 80:20 hexanes : EtOAc

^{1}H NMR: (400 MHz, $CDCl_3$) δ = 8.26 (d, *J* = 8.9 Hz, 2H), 8.17 (d, *J* = 8.9 Hz, 2H), 6.09 (dd, *J* = 17.3, 10.7 Hz, 1H), 5.70 – 5.58 (m, 2H), 5.47 (d, *J* = 10.7 Hz, 1H), 5.10 – 4.98 (m, 2H), 2.65 – 2.52 (m, 1H), 2.38 – 2.30 (m, 1H), 2.27 (dd, *J* = 13.9, 6.8 Hz, 1H), 2.18 (dd, *J* = 13.9, 7.9 Hz, 1H), 1.95 – 1.78 (m, 4H), 1.17 (s, 3H).

^{13}C NMR: (100 MHz, $CDCl_3$) δ = 207.5, 163.4, 150.5, 135.7, 135.3, 132.7, 130.8, 123.4, 119.0, 118.6, 84.4, 48.8, 42.2, 35.6, 34.5, 23.4, 18.1.

IR: *f* (cm^{-1}) = 2935, 2874, 1708, 1525, 1276, 1100, 871, 718.

HRMS (ESI-TOF): *m/z* $[M+H]^+$ =344.1492 calculated for $C_{19}H_{22}NO_5$; found for 344.1503.

Compound (-)-8i

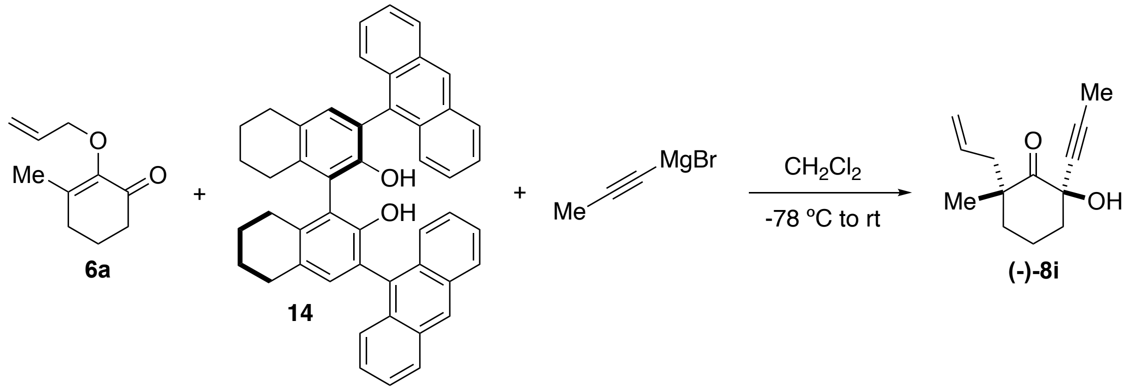

Procedure: Ligand **14** (832 mg, 1.29 mmol) and ketone **6a** (153 mg, 0.920 mmol) were dissolved in CH_2Cl_2 (18.4 mL) in a round bottom flask. After cooling the solution to -78 °C, propyn-1-ylmagnesium bromide (7.20 mL, 3.60 mmol, 0.5 M in THF) was added dropwise. After stirring at -78 °C until the starting material was fully consumed as monitored by TLC, the reaction mixture was warmed to room temperature and stirred until the Claisen rearrangement of the carbonyl addition intermediate was complete as monitored by TLC for a total reaction time of 24 hours. After cooling to 0 °C, the reaction was quenched with a saturated NH_4Cl solution (10 mL) and diluted with DI water (10 mL). The aqueous layer was extracted with CH_2Cl_2 (3 x 15 mL), and the combined organic layers were washed with brine, dried over Na_2SO_4, and concentrated under vacuum. The crude material was purified by column chromatography using 100% hexanes to 50:50 hexanes : CH_2Cl_2 to afford compound **(-)-8i** in 68% yield (129 mg, 0.625 mmol) as a pale yellow oil. ^{1}H NMR analysis of the crude reaction mixture indicated > 20:1 dr.

Rf: 0.3 in 80:20 hexanes : EtOAc

^{1}H NMR: (400 MHz, $CDCl_3$) δ = 5.58 (dtd, J = 20.9, 8.8, 5.9 Hz, 1H), 5.08 – 4.98 (m, 2H), 4.18 (s, 1H), 2.98 (dd, J = 13.9, 5.9 Hz, 1H), 2.36 (dt, J = 12.4, 3.0 Hz, 1H), 2.18 (dd, J = 13.9, 8.7 Hz, 1H), 2.13 – 2.04 (m, 1H), 1.91 – 1.84 (m, 1H), 1.80 (s, 3H), 1.63 (ddd, J = 12.4, 9.2, 5.5 Hz, 2H), 1.51 (td, J = 13.6, 3.9 Hz, 1H), 1.03 (s, 3H).

^{13}C NMR: (100 MHz, $CDCl_3$) δ = 210.4, 133.3, 118.3, 83.3, 79.2, 72.1, 48.5, 43.5, 41.6, 39.8, 23.5, 18.2, 3.5.

IR: *f* (cm^{-1}) =3480, 2936, 2857, 2110, 1706, 1640, 1458, 1377, 1213, 1108, 1029, 990, 732.

HRMS (ESI-TOF): *m/z* $[M+H]^+$ =207.1380 calculated for $C_{13}H_{19}O_2$; found for 207.1381.

Specific Rotation: $[\alpha]_{25}^{D}$ = -12.3 (c = 1.00 in $CHCl_3$).

HPLC ((-)-8i)-BzNO₂: OD-H, hexane/isopropanol = 97/3, flow rate = 1.0 mL/min, λ = 254 nm, t_R = 4.7 min (minor), 6.3 min (major).

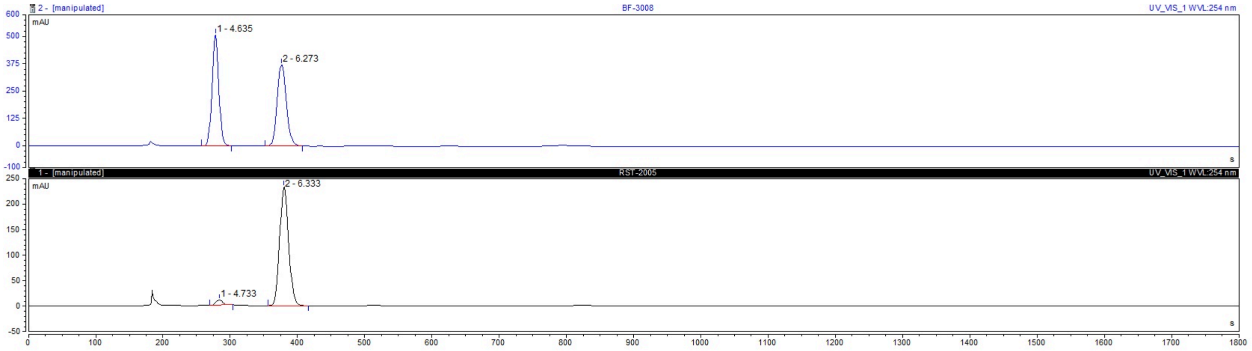

Retention Time (min)	Relative Area (%)	Area (mAU*min)	Height (mAU)
4.733	3.12	1.9532	9.93
6.333	96.88	60.5919	233.74

Compound ((-)-8i)-BzNO₂

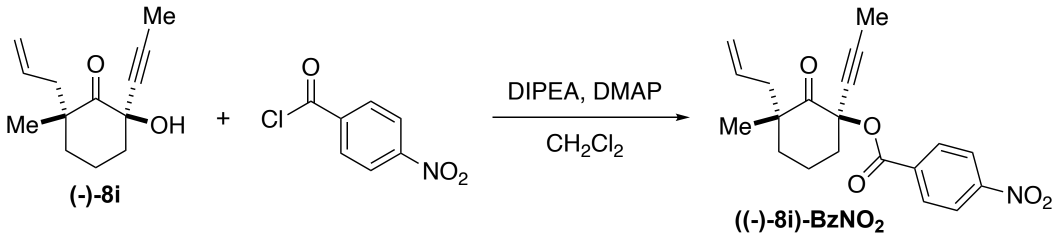

Procedure: Alcohol **(-)-8i** (50 mg, 0.24 mmol) was dissolved in CH_2Cl_2 (1.4 mL). *N*,*N*-Diisopropylethylamine (0.21 mL, 1.21 mmol) was then added to the solution, followed by 4-

nitrobenzoyl chloride (143 mg, 0.77 mmol) and 4-(dimethylamino)pyridine (29 mg, 0.24 mmol). The reaction mixture was then stirred at room temperature until complete consumption of the starting material as monitored by TLC. The crude reaction mixture was concentrated under vacuum and then purified by column chromatography using 100% hexanes to 97:3 hexanes : EtOAc to afford compound **((-)-8i)-BzNO$_2$** in 73% yield (63 mg, 0.18 mmol) as a colorless oil.

Rf: 0.3 in 90:10 hexanes : EtOAc

^{1}H NMR: (400 MHz, $CDCl_3$) δ = 8.31 – 8.12 (m, 4H), 5.74 (dddd, *J* = 16.6, 10.4, 8.4, 6.2 Hz, 1H), 5.16 – 5.02 (m, 2H), 2.83 (dd, *J* = 13.9, 5.9 Hz, 1H), 2.56 – 2.41 (m, 2H), 2.28 (dd, *J* = 13.8, 8.4 Hz, 1H), 2.17 (dddd, *J* = 13.9, 11.5, 6.8, 1.5 Hz, 1H), 1.93 (s, 3H), 1.92 – 1.86 (m, 1H), 1.85 – 1.69 (m, 2H), 1.13 (s, 3H).

^{13}C NMR: (100 MHz, $CDCl_3$) δ = 203.0, 162.8, 150.6, 135.6, 133.3, 130.9, 123.4, 118.4, 87.0, 80.1, 75.5, 49.5, 43.7, 38.7, 37.7, 23.6, 18.3, 3.9.

IR: *f* (cm^{-1}) = 3114, 2930, 2110, 1737, 1717, 1524, 1348, 1269, 1096, 872, 714.

HRMS (ESI-TOF): *m/z* $[M+H]^+$ =356.1492 calculated for $C_{20}H_{22}NO_5$; found for 356.1491.

Compound (-)-8j

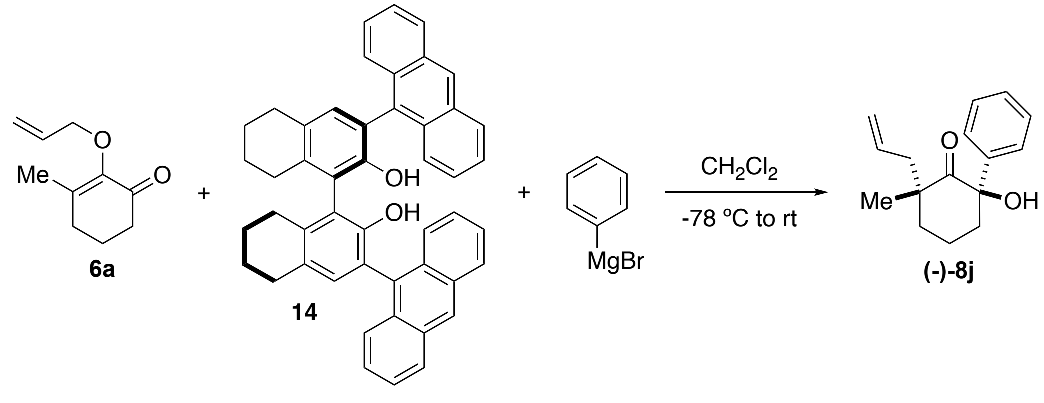

Procedure: Ligand **14** (877 mg, 1.36 mmol) and ketone **6a** (161 mg, 0.967 mmol) were dissolved in CH_2Cl_2 (19.9 mL) in a round bottom flask. After cooling the solution to -78 °C,

phenylmagnesium bromide (1.26 mL, 3.78 mmol, 3.0 M in Et_2O) was added dropwise. After stirring at -78 °C until the starting material was fully consumed as monitored by TLC, the reaction mixture was warmed to room temperature and stirred until the Claisen rearrangement of the carbonyl addition intermediate was complete as monitored by TLC for a total reaction time of 40 hours. After cooling to 0 °C, the reaction was quenched with a saturated NH_4Cl solution (5 mL) and diluted with DI water (5 mL). The aqueous layer was extracted with CH_2Cl_2 (3 x 5 mL), and the combined organic layers were washed with brine, dried over Na_2SO_4, and concentrated under vacuum. The crude material was purified by column chromatography using 100% hexanes to 50:50 hexanes : CH_2Cl_2 to afford compound **(-)-8j** in 75% yield (178 mg, 0.729 mmol) as a pale yellow oil. ^1H NMR analysis of the crude reaction mixture indicated > 20:1 dr.

Rf: 0.4 in 90:10 hexanes : EtOAc

^{1}H NMR: (500 MHz, $CDCl_3$) δ = 7.38 – 7.33 (m, 4H), 7.32 – 7.27 (m, 1H), 5.35 (dddd, *J* = 16.8, 10.1, 8.1, 6.5 Hz, 1H), 4.93 – 4.87 (m, 1H), 4.77 (s, 1H), 4.70 (dd, *J* = 16.9, 1.7 Hz, 1H), 2.97 (dq, *J* = 13.9, 3.0 Hz, 1H), 2.08 – 1.98 (m, 1H), 1.96 (dd, *J* = 13.8, 4.3 Hz, 1H), 1.92 – 1.79 (m, 4H), 1.71 (dd, *J* = 14.0, 8.1 Hz, 1H), 1.63 – 1.55 (m, 1H), 1.10 (s, 3H).

^{13}C NMR: (125 MHz, $CDCl_3$) δ = 215.6, 140.1, 132.5, 128.8, 128.8, 128.2, 125.8, 118.5, 78.3, 48.4, 41.7, 38.2, 36.8, 23.4, 18.5.

IR: *f* (cm^{-1}) = 3464, 2935, 1697, 1449, 1377, 990, 922, 730, 683.

HRMS (ESI-TOF): *m/z* $[M+H]^+$ =245.1536 calculated for $C_{16}H_{21}O_2$; found for 245.1525.

Specific Rotation: $[\alpha]_{25}^{D}$ = -5.3 (c = 1.00 in $CHCl_3$)

HPLC ((-)-8j)-BzNO$_2$: OD-H, hexane/isopropanol = 90/10, flow rate = 1.0 mL/min, λ = 254 nm, t_R = 6.2 min (major), 8.0 min (minor).

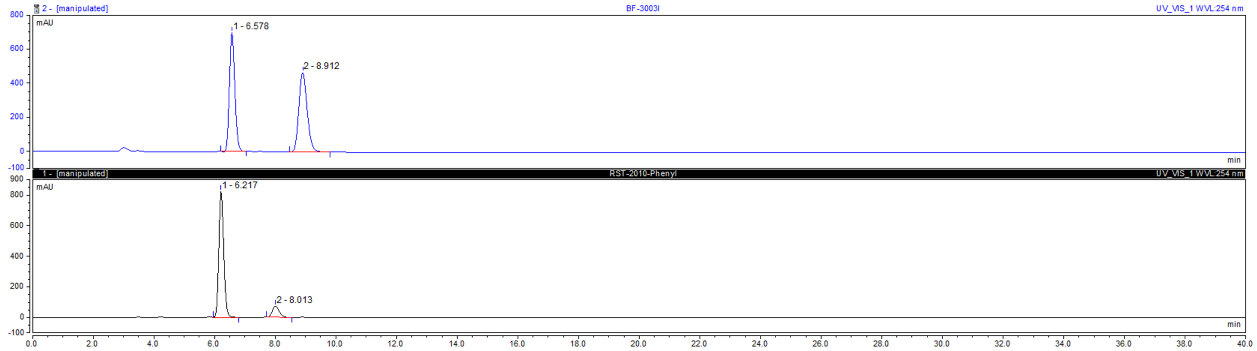

Retention Time (min)	Relative Area (%)	Area (mAU*min)	Height (mAU)
6.217	89.02	155.1294	821.12
8.013	10.98	19.1392	72.46

Compound ((-)-8j)-BzNO$_2$

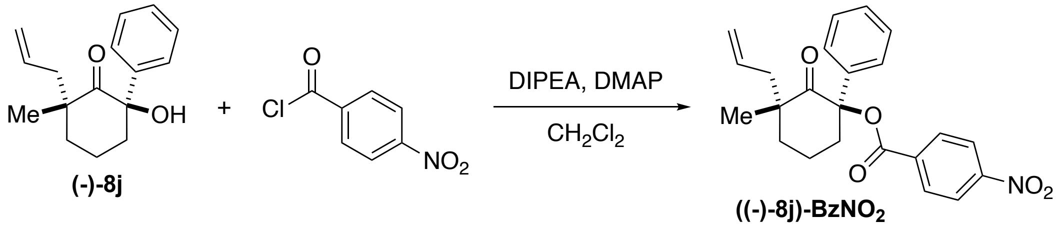

Procedure: Alcohol **(-)-8j** (50 mg, 0.20 mmol) was dissolved in CH_2Cl_2 (1.1 mL). *N*,*N*-Diisopropylethylamine (0.17 mL, 0.98 mmol) was then added to the solution, followed by 4-nitrobenzoyl chloride (121 mg, 0.65 mmol) and 4-(dimethylamino)pyridine (25 mg, 0.20 mmol). The reaction mixture was then stirred at room temperature until complete consumption of the starting material as monitored by TLC. The crude reaction mixture was concentrated under vacuum and then purified by column chromatography using 100% hexanes to 98:2 hexanes : EtOAc to afford compound **((-)-8j)-BzNO$_2$** in 93% yield (75 mg, 0.19 mmol) as a colorless oil.

Rf: 0.5 in 90:10 hexanes : EtOAc

^{1}H NMR: (400 MHz, $CDCl_3$) δ = 8.27 – 8.21 (m, 2H), 8.18 – 8.12 (m, 2H), 7.75 – 7.68 (m, 2H), 7.48 – 7.38 (m, 3H), 5.39 – 5.25 (m, 1H), 4.89 – 4.83 (m, 1H), 4.59 (dq, *J* = 16.9, 1.2 Hz, 1H), 3.03 – 2.87 (m, 2H), 2.25 – 2.08 (m, 1H), 2.05 – 1.93 (m, 2H), 1.91 – 1.82 (m, 2H), 1.78 (dd, *J* = 13.9, 7.8 Hz, 1H), 1.18 (s, 3H).

^{13}C NMR: (125 MHz, $CDCl_3$) δ = 207.2, 163.6, 150.5, 136.2, 135.9, 132.3, 130.8, 129.2, 128.7, 127.3, 123.4, 118.5, 85.5, 49.3, 41.9, 35.7, 33.2, 23.5, 19.2.

IR: *f* (cm^{-1}) = 2932, 1725, 1710, 1527, 1349, 1320, 1277, 1100, 1015, 800, 720, 700.

HRMS (ESI-TOF): *m/z* $[M+Na]^+$ =416.1474 calculated for $C_{23}H_{23}NO_5Na$; found for 416.1449.

Compound (-)-8k

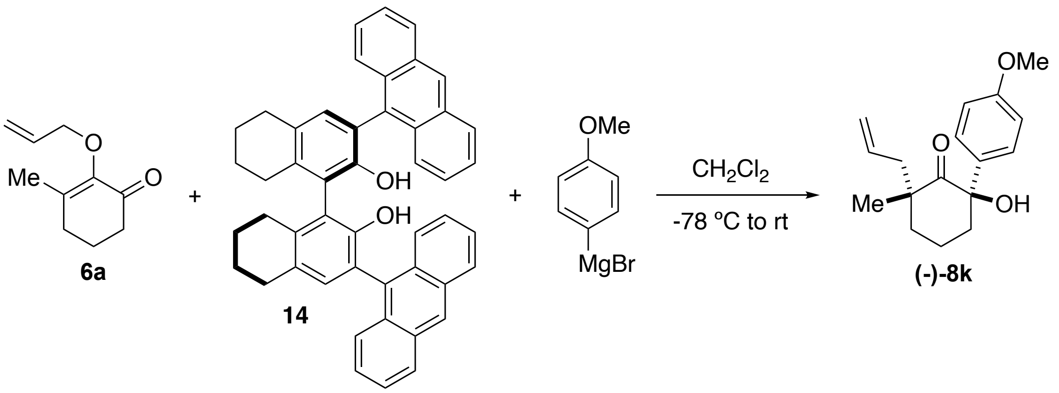

Procedure: Ligand **14** (546 mg, 0.842 mmol) and ketone **6a** (100 mg, 0.602 mmol) were dissolved in CH_2Cl_2 (12.0 mL) in a round bottom flask. After cooling the solution to -78 °C, 4-methoxyphenylmagnesium bromide (5.20 mL, 2.34 mmol, 0.45 M in Et_2O) was added dropwise. After stirring at -78 °C until the starting material was fully consumed as monitored by TLC, the reaction mixture was warmed to room temperature and stirred until the Claisen rearrangement of the carbonyl addition intermediate was complete as monitored by TLC for a total reaction time of 22 hours. After cooling to 0 °C, the reaction was quenched with a saturated NH_4Cl solution (5 mL) and diluted with DI water (5 mL). The aqueous layer was extracted with CH_2Cl_2 (3 x 5 mL),

and the combined organic layers were washed with brine, dried over Na_2SO_4, and concentrated under vacuum. The crude material was purified by column chromatography using 100% hexanes to 50:50 hexanes : CH_2Cl_2 to afford compound **(-)-8k** in 73% yield (120 mg, 0.437 mmol) as a pale yellow oil. ^1H NMR analysis of the crude reaction mixture indicated 17:1 dr.

Rf: 0.5 in 50:50 hexanes : CH_2Cl_2 then 80:20 hexanes : EtOAc

^{1}H NMR: (400 MHz, $CDCl_3$) δ = 7.29 (d, *J* = 8.8 Hz, 2H), 6.89 (d, *J* = 8.9 Hz, 2H), 5.44 – 5.31 (m, 1H), 4.96 – 4.90 (m, 1H), 4.75 (dd, *J* = 16.9, 1.8 Hz, 2H), 3.80 (s, 4H), 2.94 (dd, *J* = 13.2, 3.0 Hz, 1H), 2.07 – 1.71 (m, 7H), 1.58 (ddd, *J* = 12.7, 11.3, 3.8 Hz, 1H), 1.10 (s, 3H).

^{13}C NMR: (100 MHz, $CDCl_3$) δ = 215.7, 159.3, 132.6, 132.2, 127.1, 118.5, 114.0, 77.8, 55.2, 48.1, 41.8, 37.9, 36.8, 23.3, 18.4.

IR: *f* (cm^{-1}) = 3468, 2935, 1694, 1609, 1511, 1251, 1181, 1033, 911, 729.

HRMS (ESI-TOF): *m/z* $[M+Na]^+$ =297.1467 calculated for $C_{17}H_{22}O_3Na$; found for 297.1458.

Specific Rotation: $[\alpha]_{25}^{D}$ = -15.2 (c = 1.00 in $CHCl_3$)

HPLC ((-)-8k)-BzNO$_2$: OD-H, hexane/isopropanol = 92.5/7.5, flow rate = 1.0 mL/min, λ = 254 nm, t_R = 4.6 min (major), 5.7 min (minor).

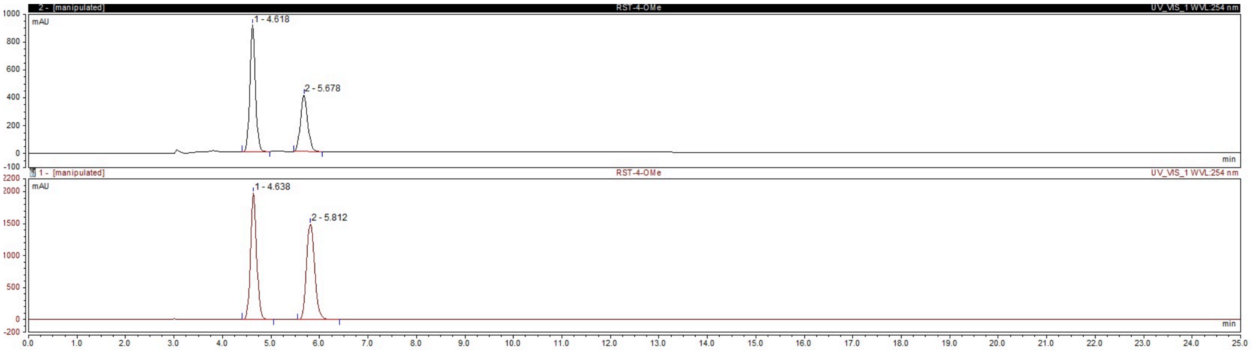

Retention Time (min)	Relative Area (%)	Area (mAU*min)	Height (mAU)
4.618	63.40	122.4774	906.90
5.678	36.60	70.7099	404.22

Compound ((-)-8k)-BzNO₂

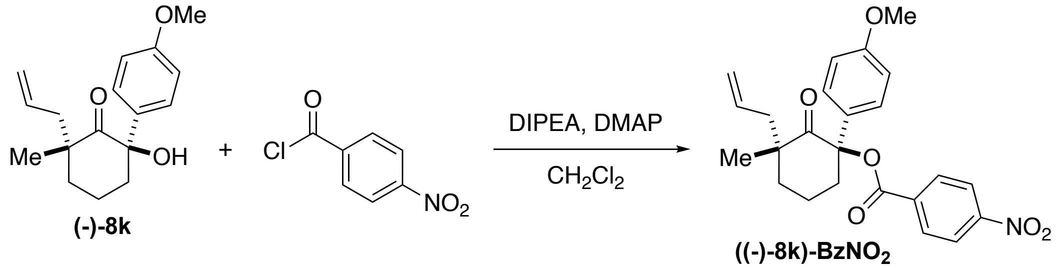

Procedure: Alcohol **(-)-8k** (50 mg, 0.18 mmol) was dissolved in CH_2Cl_2 (1.0 mL). *N*,*N*-Diisopropylethylamine (0.16 mL, 0.91 mmol) was then added to the solution, followed by 4-nitrobenzoyl chloride (108 mg, 0.58 mmol) and 4-(dimethylamino)pyridine (22 mg, 0.18 mmol). The reaction mixture was then stirred at room temperature until complete consumption of the starting material as monitored by TLC. The crude reaction mixture was concentrated under vacuum and then purified by column chromatography using 100% hexanes to 98:2 hexanes : EtOAc to afford compound **((-)-8k)-BzNO₂** in 86% yield (66 mg, 0.16 mmol) as a colorless oil.

Rf: 0.4 in 90:10 hexanes : EtOAc

^{1}H NMR: (400 MHz, $CDCl_3$) δ = 8.22 (d, *J* = 9.0 Hz, 2H), 8.13 (d, *J* = 8.9 Hz, 2H), 7.61 (d, *J* = 9.0 Hz, 2H), 6.95 (d, *J* = 8.9 Hz, 2H), 5.40 – 5.29 (m, 1H), 4.92 – 4.85 (m, 1H), 4.70 – 4.61 (m, 1H), 3.84 (s, 4H), 2.97 – 2.86 (m, 2H), 2.18 – 2.04 (m, 1H), 2.02 – 1.91 (m, 2H), 1.89 – 1.75 (m, 4H), 1.16 (s, 3H).

^{13}C NMR: (100 MHz, $CDCl_3$) δ = 207.4, 163.6, 160.1, 150.4, 136.0, 132.4, 130.8, 128.7, 127.8, 123.4, 118.5, 114.0, 85.4, 49.1, 42.0, 35.6, 33.0, 33.0, 23.4, 19.0.

IR: *f* (cm^{-1}) = 2934, 1720, 1706, 1607, 1527, 1276, 1254, 1098, 830, 718.

HRMS (ESI-TOF): *m/z* $[M+Na]^+$ =446.1580 calculated for $C_{24}H_{25}NO_6Na$; found for 446.1566.

Compound (+)-8n

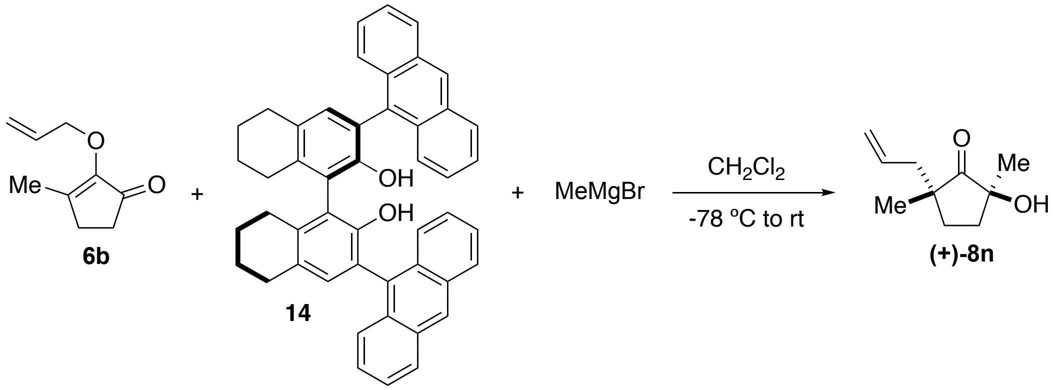

Procedure: Ligand **14** (279 mg, 0.432 mmol) and ketone **6b** (47 mg, 0.31 mmol) were dissolved in CH_2Cl_2 (6.2 mL) in a round bottom flask. After cooling the solution to -78 °C, methylmagnesium bromide (0.40 mL, 1.20 mmol, 3.0 M in Et_2O) was added dropwise. After stirring at -78 °C until the starting material was fully consumed as monitored by TLC, the reaction mixture was warmed to room temperature and stirred until the Claisen rearrangement of the carbonyl addition intermediate was complete as monitored by TLC for a total reaction time of 26 hours. After cooling to 0 °C, the reaction was quenched with a saturated NH_4Cl solution (5 mL) and diluted with DI water (5 mL). The aqueous layer was extracted with CH_2Cl_2 (3 x 10 mL), and the combined organic layers were washed with brine, dried over Na_2SO_4, and concentrated under vacuum. The crude material was purified by column chromatography using 100% hexanes to 50:50 hexanes : CH_2Cl_2 to afford compound **(+)-8n** in 79% yield (41 mg, 0.24 mmol) as a pale yellow oil. ^{1}H NMR analysis of the crude reaction mixture indicated > 20:1 dr.

Rf: 0.3 in 50:50 hexanes : CH_2Cl_2 then 80:20 hexanes : EtOAc

^{1}H NMR: (500 MHz, DMSO-d_6) δ = 5.72 – 5.61 (m, 1H), 5.25 (s, 1H), 5.10 – 5.01 (m, 2H), 2.09 (dd, J = 13.5, 7.0 Hz, 1H), 2.03 (dd, J = 13.5, 7.8 Hz, 1H), 1.84 (dt, J = 12.1, 7.7 Hz, 1H), 1.80 – 1.68 (m, 2H), 1.65 – 1.60 (m, 1H), 1.07 (s, 3H), 0.99 (s, 3H).

^{13}C NMR: (125 MHz, DMSO-d_6) δ = 221.8, 134.1, 118.2, 75.9, 46.3, 41.6, 34.5, 29.8, 23.3, 22.7.

IR: f (cm^{-1}) = 3448, 2959, 2925, 2855, 1742, 1460, 1374, 1259, 1179, 1064, 1035, 916, 796.

HRMS (ESI-TOF): m/z $[M\text{-}OH]^+$ = 151.1123 calculated for $C_{10}H_{15}O$; found 151.1121.

Specific Rotation: $[\alpha]_{25}^{D}$ = +14.2 (c = 1.00 in $CHCl_3$)

HPLC ((+)-8n)-BzNO$_2$: OD-H, hexane/isopropanol = 95/5, flow rate = 1.0 mL/min, λ = 254 nm, t_R = 11.7 min (major), 15.1 min (minor).

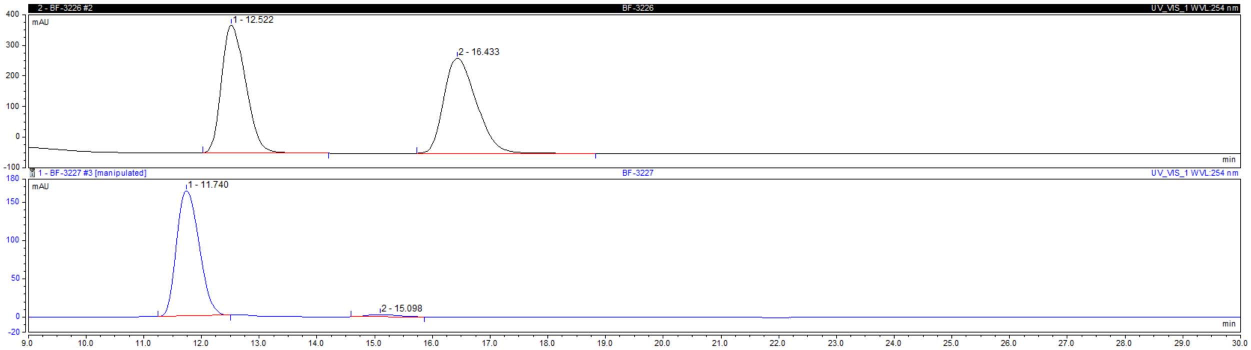

Retention Time (min)	Relative Area (%)	Area (mAU*min)	Height (mAU)
11.740	97.78	73.9040	163.03
15.098	2.22	1.6816	2.91

Compound ((+)-8n)-BzNO$_2$

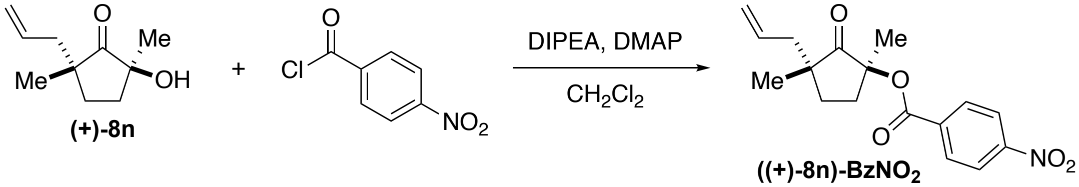

Procedure: Alcohol **(+)-8n** (22 mg, 0.13 mmol) was dissolved in CH_2Cl_2 (0.7 mL). *N,N*-Diisopropylethylamine (0.11 mL, 0.65 mmol) was then added to the solution, followed by 4-nitrobenzoyl chloride (77 mg, 0.42 mmol) and 4-(dimethylamino)pyridine (16 mg, 0.13 mmol). The reaction mixture was then stirred at room temperature until complete consumption of the starting material as monitored by TLC. The crude reaction mixture was concentrated under vacuum and then purified by column chromatography using 100% hexanes to 99:1 hexanes : EtOAc to afford compound **((+)-8n)-BzNO$_2$** in 72% yield (30 mg, 0.095 mmol) as a colorless oil.

Rf: 0.6 in 80:20 hexanes : EtOAc

^{1}H NMR: (500 MHz, $CDCl_3$) δ = 8.33 – 8.24 (m, 2H), 8.23 – 8.14 (m, 2H), 5.78 – 5.65 (m, 1H), 5.18 – 5.05 (m, 2H), 2.71 – 2.59 (m, 1H), 2.29 (dd, *J* = 13.6, 6.8 Hz, 1H), 2.21 (dd, *J* = 13.7, 8.1 Hz, 1H), 2.09 – 2.05 (m, 1H), 1.97 – 1.85 (m, 2H), 1.50 (s, 3H), 1.36 (s, 3H).

^{13}C NMR: (125 MHz, $CDCl_3$) δ = 217.0, 162.8, 150.6, 135.3, 133.4, 130.9, 123.5, 118.9, 84.6, 47.3, 43.0, 30.9, 30.4, 25.1, 21.6.

IR: *f* (cm^{-1}) = 2978, 1751, 1724, 1607, 1528, 1461, 1377, 1348, 1321, 1287, 1188, 1119, 1104, 1031, 843, 719.

HRMS (ESI-TOF): *m/z* $[M+H]^+$ = 318.1343 calculated for $C_{17}H_{20}NO_5$; found 318.1345.

Compound (-)-10a

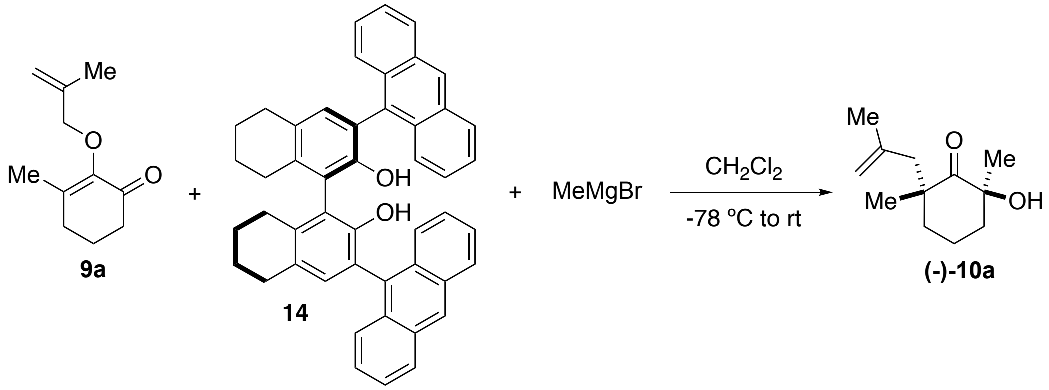

Procedure: Ligand **14** (305 mg, 0.47 mmol) and ketone **9a** (60 mg, 0.332 mmol) were dissolved in CH_2Cl_2 (6.7 mL) in a round bottom flask. After cooling the solution to -78 °C, methylmagnesium bromide (0.44 mL, 1.32 mmol, 3.0 M in Et_2O) was added dropwise. After stirring at -78 °C until the starting material was fully consumed as monitored by TLC, the reaction mixture was warmed to room temperature and stirred until the Claisen rearrangement of the carbonyl addition intermediate was complete as monitored by TLC for a total reaction time of 44 hours. After cooling to 0 °C, the reaction was quenched with a saturated NH_4Cl solution (8 mL) and diluted with DI water (8 mL). The aqueous layer was extracted with CH_2Cl_2 (3 x 10 mL), and the combined organic layers were washed with brine, dried over Na_2SO_4, and concentrated under vacuum. The crude material was purified by column chromatography using 100% hexanes to 50:50 hexanes : CH_2Cl_2 to afford compound **(-)-10a** in 78% yield (51 mg, 0.260 mmol) as a colorless oil. ^{1}H NMR analysis of the crude reaction mixture indicated > 20:1 dr.

Rf: 0.5 in 50:50 hexanes : CH_2Cl_2 then 80:20 hexanes : EtOAc

^{1}H NMR: (500 MHz, $CDCl_3$) δ = 4.86 – 4.85 (m, 1H), 4.66 – 4.65 (m, 1H), 3.85 (br s, 1H), 2.41 (d, *J* = 14.3 Hz, 1H), 2.32 (dd, *J* = 14.2, 1.0 Hz, 1H), 2.11 – 2.04 (m, 1H), 1.98 – 1.92 (m, 1H), 1.84 – 1.70 (m, 3H), 1.68 (s, 3H), 1.57 – 1.50 (m, 1H), 1.42 (s, 3H), 1.15 (s, 3H).

^{13}C NMR: (125 MHz, $CDCl_3$) δ = 218.3, 141.5, 115.2, 76.0, 47.9, 45.0, 40.8, 39.4, 27.2, 24.9, 24.7, 18.3.

IR: *f* (cm^{-1}) = 3484, 3075, 2935, 2872, 1699, 1643, 1456, 1374, 1259, 1160, 1011, 977, 893.

HRMS (ESI-TOF): *m/z* $[M+H]^+$ = 197.1536 calculated for $C_{12}H_{21}O_2$; found 197.1545.

Specific Rotation: $[\alpha]_{25}^{D}$ = -6.0 (c = 1.00 in $CHCl_3$)

HPLC ((-)-10a)-BzNO$_2$: OD-H, hexane/isopropanol = 80/20, flow rate = 1.0 mL/min, λ = 254 nm, t_R = 6.1 min (major), 9.3 min (minor).

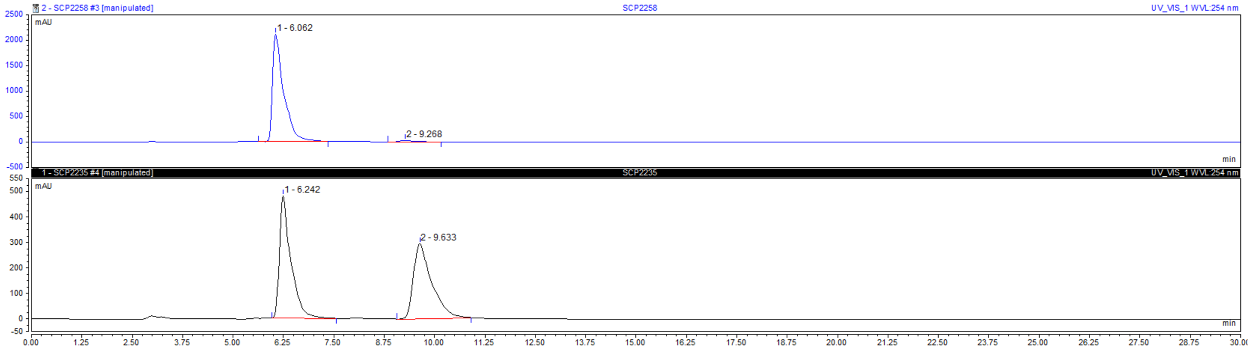

Retention Time (min)	Relative Area (%)	Area (mAU*min)	Height (mAU)
6.062	98.20	672.1463	2108.50
9.268	1.80	12.2965	26.88

Compound ((-)-10a)-BzNO$_2$

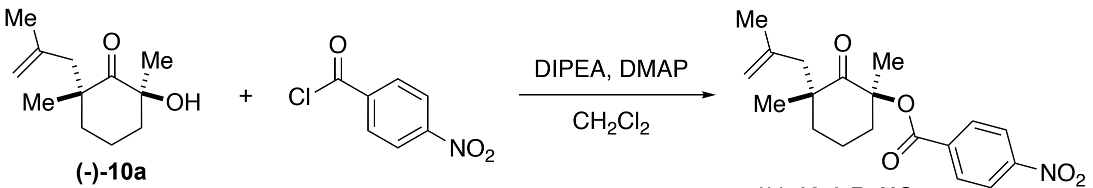

(-)-10a **((-)-10a)-BzNO$_2$**

Procedure: Alcohol **(-)-10a** (19 mg, 0.097 mmol) was dissolved in CH_2Cl_2 (0.5 mL). *N*,*N*-Diisopropylethylamine (51 µL, 0.29 mmol) was then added to the solution, followed by 4-

nitrobenzoyl chloride (54 mg, 0.29 mmol) and 4-(dimethylamino)pyridine (12 mg, 0.097 mmol). The reaction mixture was then stirred at room temperature until complete consumption of the starting material as monitored by TLC. The crude reaction mixture was concentrated under vacuum and then purified by column chromatography using 100% hexanes to 97:3 hexanes : EtOAc to afford compound **((-)-10a)-BzNO$_2$** in 69% yield (23 mg, 0.067 mmol) as a white solid.

Rf: 0.6 in 80:20 hexanes : EtOAc

^{1}H NMR: (500 MHz, $CDCl_3$) δ = 8.30 – 8.24 (m, 2H), 8.20 – 8.14 (m, 2H), 4.92 (q, J = 1.7 Hz, 1H), 4.71 – 4.67 (m, 1H), 2.57 – 2.51 (m, 1H), 2.31 (d, J = 13.4 Hz, 1H), 2.23 (d, J = 13.4 Hz, 1H), 2.04 – 1.97 (m, 1H), 1.96 – 1.81 (m, 4H), 1.78 (s, 3H), 1.64 (s, 3H), 1.24 (s, 3H).

^{13}C NMR: (125 MHz, $CDCl_3$) δ = 211.2, 163.5, 150.5, 141.5, 135.7, 130.8 123.4, 116.1, 83.5, 49.0, 45.3, 37.5, 35.9, 25.4, 25.1, 24.2, 18.4.

IR: f (cm^{-1}) = 2937, 1709, 1607, 1528, 1461, 1376, 1349, 1320, 1287, 1103, 1015, 865, 720.

HRMS (ESI-TOF): m/z $[M+H]^+$ = 346.1656 calculated for $C_{19}H_{24}NO_5$; found 346.1657.

Compound (+)-10b

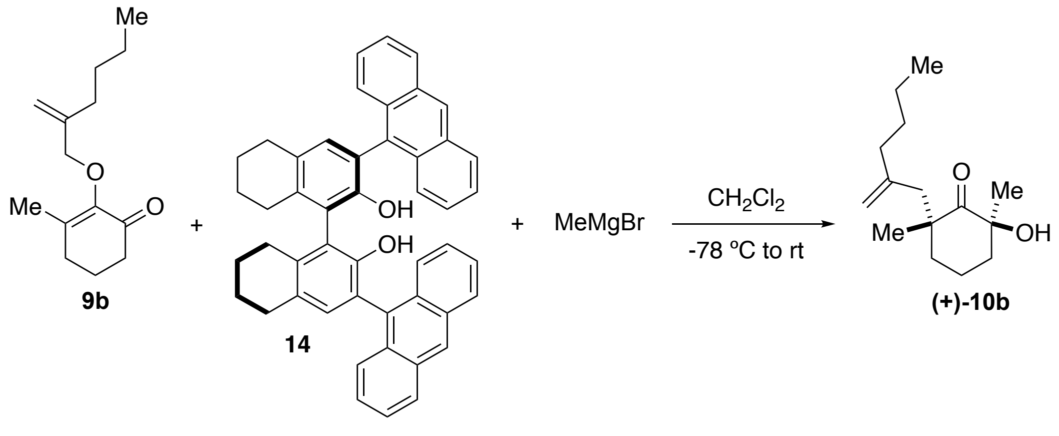

Procedure: Ligand **14** (420 mg, 0.649 mmol) and ketone **9b** (103 mg, 0.463 mmol) were dissolved in CH_2Cl_2 (9.3 mL) in a round bottom flask. After cooling the solution to -78 °C,

methylmagnesium bromide (0.60 mL, 1.81 mmol, 3.0 M in Et_2O) was added dropwise. After stirring at -78 °C until the starting material was fully consumed as monitored by TLC, the reaction mixture was warmed to room temperature and stirred until the Claisen rearrangement of the carbonyl addition intermediate was complete as monitored by TLC for a total reaction time of 20 hours. After cooling to 0 °C, the reaction was quenched with a saturated NH_4Cl solution (8 mL) and diluted with DI water (8 mL). The aqueous layer was extracted with CH_2Cl_2 (3 x 10 mL), and the combined organic layers were washed with brine, dried over Na_2SO_4, and concentrated under vacuum. The crude material was purified by column chromatography using 100% hexanes to 50:50 hexanes : CH_2Cl_2 to afford compound **(+)-10b** in 90% yield (99 mg, 0.415 mmol) as a colorless oil. ^{1}H NMR analysis of the crude reaction mixture indicated > 20:1 dr.

Rf: 0.5 in 50:50 hexanes : CH_2Cl_2 then 80:20 hexanes : EtOAc

^{1}H NMR: (500 MHz, $CDCl_3$) δ = 4.87 (d, *J* = 1.6 Hz, 1H), 4.67 (d, *J* = 1.6 Hz, 1H), 3.91 (s, 1H), 2.34 (s, 2H), 2.11 – 2.07 (m, 1H), 2.01 – 1.96 (m, 3H), 1.85 – 1.69 (m, 3H), 1.56 – 1.47 (m, 1H), 1.41 (s, 3H), 1.41 – 1.36 (m, 2H), 1.36 – 1.28 (m, 2H), 1.14 (s, 3H), 0.89 (t, *J* = 7.3 Hz, 3H).

^{13}C NMR: (125 MHz, $CDCl_3$) δ = 218.3, 145.6, 113.5, 76.0, 48.0, 43.0, 40.8, 39.1, 37.7, 30.2, 27.0, 24.9, 22.4, 18.3, 14.0.

IR: *f* (cm^{-1}) = 3481, 2930, 2871, 1699, 1639, 1458, 1373, 1260, 1160, 1012, 976, 894, 802, 555.

HRMS (ESI-TOF): *m/z* $[M+H]^+$ = 239.2006 calculated for $C_{15}H_{27}O_2$; found 239.1994.

Specific Rotation: $[\alpha]_{25}^{D}$ = +7.9 (c = 1.00 in $CHCl_3$)

HPLC ((+)-10b)-$BzNO_2$: OD-H, hexane/isopropanol = 98/2 to 97/3 over 60 min, flow rate = 1.0 mL/min, λ = 254 nm, t_R = 8.9 min (major), 14.0 min (minor).

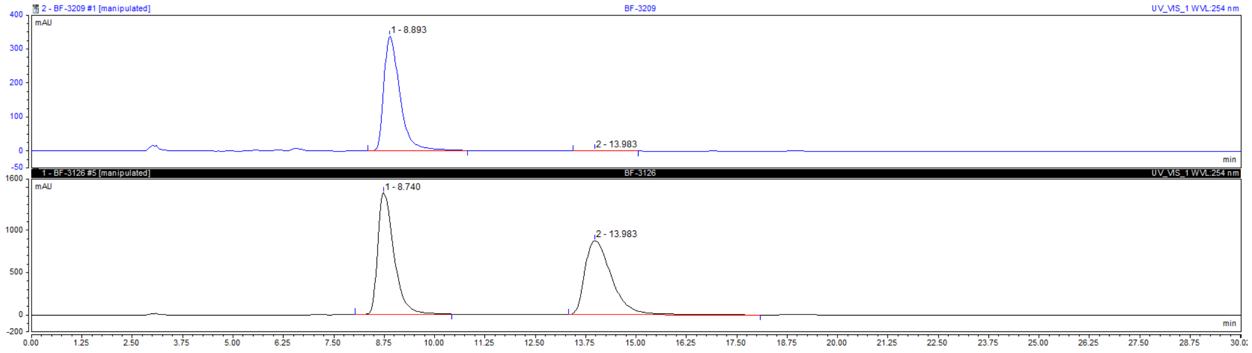

Retention Time (min)	Relative Area (%)	Area (mAU*min)	Height (mAU)
8.893	99.37	154.6244	336.83
13.983	0.63	0.9741	1.54

Compound ((+)-10b)-BzNO$_2$

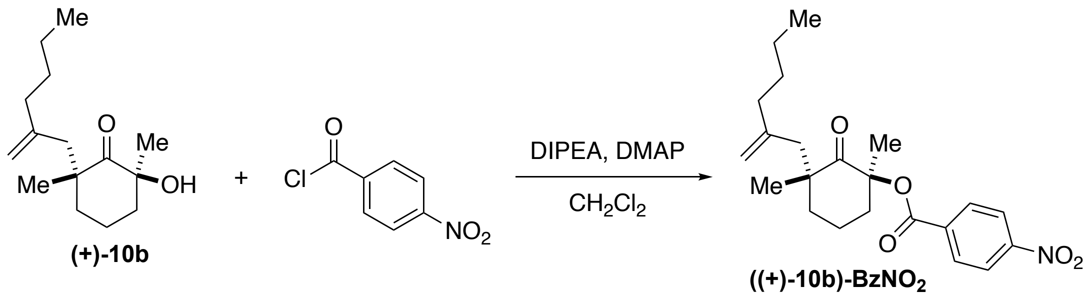

Procedure: Alcohol **(+)-10b** (28 mg, 0.12 mmol) was dissolved in CH_2Cl_2 (0.7 mL). *N*,*N*-Diisopropylethylamine (0.10 mL, 0.59 mmol) was then added to the solution, followed by 4-nitrobenzoyl chloride (71 mg, 0.38 mmol) and 4-(dimethylamino)pyridine (15 mg, 0.12 mmol). The reaction mixture was then stirred at room temperature until complete consumption of the starting material as monitored by TLC. The crude reaction mixture was concentrated under vacuum and then purified by column chromatography using 100% hexanes to 98:2 hexanes : EtOAc to afford compound **((+)-10b)-BzNO$_2$** in 84% yield (38 mg, 0.098 mmol) as a colorless oil.

Rf: 0.8 in 80:20 hexanes : EtOAc

^{1}H NMR: (400 MHz, $CDCl_3$) δ = 8.29 – 8.25 (m, 2H), 8.20 – 8.16 (m, 2H), 4.94 (d, *J* = 1.5 Hz, 1H), 4.73 (s, 1H), 2.62 – 2.49 (m, 1H), 2.32 (d, *J* = 13.5 Hz, 1H), 2.19 (d, *J* = 13.5 Hz, 1H), 2.02 (m, 3H), 1.98 – 1.79 (m, 4H), 1.64 (s, 3H), 1.47 – 1.37 (m, 2H), 1.34 – 1.27 (m, 2H), 1.23 (s, 3H), 0.90 (t, *J* = 7.2 Hz, 3H).

^{13}C NMR: (125 MHz, $CDCl_3$) δ = 211.4, 163.5, 150.5, 145.5, 135.7, 130.8, 123.4, 114.7, 83.5, 49.2, 43.2, 37.7, 37.4, 35.4, 30.5, 25.5, 24.2, 22.4, 18.4, 14.0.

IR: *f* (cm^{-1}) = 2931, 2871, 1708, 1527, 1461, 1374, 1348, 1320, 1284, 1166, 1113, 1102, 1008, 863, 720.

HRMS (ESI-TOF): *m/z* $[M+H]^+$ = 388.2126 calculated for $C_{22}H_{30}NO_5$; found 388.2127.

Compound (+)-10c

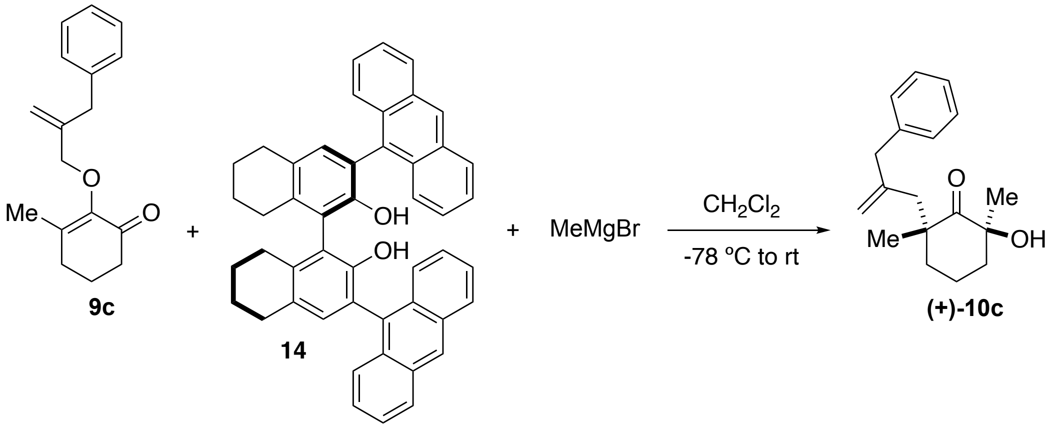

Procedure: Ligand **14** (360 mg, 0.557 mmol) and ketone **9c** (102 mg, 0.398 mmol) were dissolved in CH_2Cl_2 (8.0 mL) in a round bottom flask. After cooling the solution to -78 °C, methylmagnesium bromide (0.52 mL, 1.55 mmol, 3.0 M in Et_2O) was added dropwise. After stirring at -78 °C until the starting material was fully consumed as monitored by TLC, the reaction mixture was warmed to room temperature and stirred until the Claisen rearrangement of the carbonyl addition intermediate was complete as monitored by TLC for a total reaction time of 18

hours. After cooling to 0 °C, the reaction was quenched with a saturated NH_4Cl solution (8 mL) and diluted with DI water (8 mL). The aqueous layer was extracted with CH_2Cl_2 (3 x 10 mL), and the combined organic layers were washed with brine, dried over Na_2SO_4, and concentrated under vacuum. The crude material was purified by column chromatography using 100% hexanes to 50:50 hexanes : CH_2Cl_2 to afford compound **(+)-10c** in 88% yield (95 mg, 0.349 mmol) as a colorless oil. ^{1}H NMR analysis of the crude reaction mixture indicated > 20:1 dr.

Rf: 0.4 in 50:50 hexanes : CH_2Cl_2 then 80:20 hexanes : EtOAc

^{1}H NMR: (500 MHz, $CDCl_3$) δ = 7.29 (t, *J* = 7.4 Hz, 2H), 7.23 – 7.19 (m, 1H), 7.18 – 7.13 (m, 2H), 4.89 (d, *J* = 1.5 Hz, 1H), 4.82 (d, *J* = 1.5 Hz, 1H), 3.68 (s, 1H), 3.30 (s, 2H), 2.37 (d, *J* = 15.0 Hz, 1H), 2.27 (d, *J* = 15.0 Hz, 1H), 2.11 – 2.04 (m, 1H), 2.04 – 1.95 (m, 1H), 1.79 – 1.67 (m, 3H), 1.58 – 1.51 (m, 1H), 1.32 (s, 3H), 1.20 (s, 3H).

^{13}C NMR: (125 MHz, $CDCl_3$) δ = 218.2, 144.5, 139.1, 129.0, 128.3, 126.3, 116.0, 76.1, 48.1, 45.0, 42.1, 40.9, 39.5, 26.8, 25.0, 18.3.

IR: *f* (cm^{-1}) = 3476, 3027, 2928, 2870, 1698, 1495, 1454, 1373, 1160, 1013, 903, 740, 700, 494.

HRMS (ESI-TOF): *m/z* $[M+H]^+$ = 273.1849 calculated for $C_{18}H_{25}O_2$; found 273.1854.

Specific Rotation: $[\alpha]_{25}^{D}$ = +4.9 (c = 1.00 in $CHCl_3$)

HPLC ((+)-10c)-BzNO$_2$: OD-H, hexane/isopropanol = 95/5, flow rate = 1.0 mL/min, λ = 254 nm, t_R = 13.4 min (major), 18.5 min (minor).

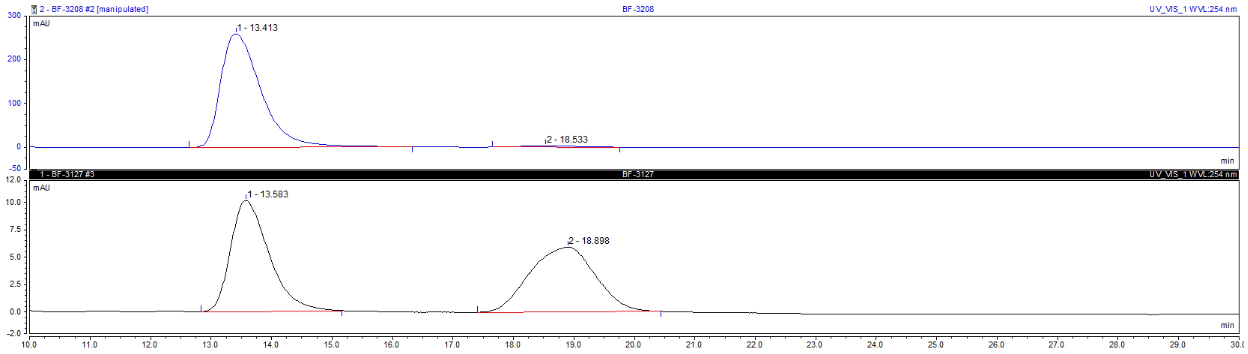

Retention Time (min)	Relative Area (%)	Area (mAU*min)	Height (mAU)
13.413	98.52	199.5388	258.96
18.533	1.48	2.9891	2.60

Compound ((+)-10c)-BzNO$_2$

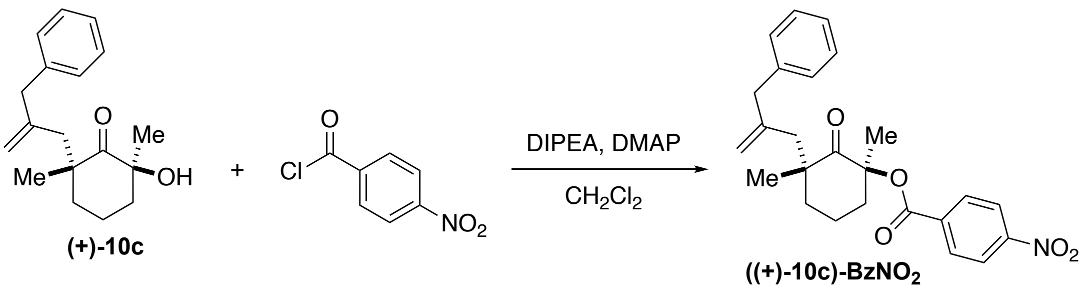

Procedure: Alcohol **(+)-10c** (35 mg, 0.13 mmol) was dissolved in CH_2Cl_2 (0.7 mL). *N*,*N*-Diisopropylethylamine (0.11 mL, 0.64 mmol) was then added to the solution, followed by 4-nitrobenzoyl chloride (76 mg, 0.41 mmol) and 4-(dimethylamino)pyridine (15 mg, 0.13 mmol). The reaction mixture was then stirred at room temperature until complete consumption of the starting material as monitored by TLC. The crude reaction mixture was concentrated under vacuum and then purified by column chromatography using 100% hexanes to 98:2 hexanes : EtOAc to afford compound **((+)-10c)-BzNO$_2$** in 76% yield (41 mg, 0.097 mmol) as a colorless oil.

Rf: 0.5 in 80:20 hexanes : EtOAc

^{1}H NMR: (500 MHz, $CDCl_3$) δ = 8.27 (d, *J* = 8.7 Hz, 2H), 8.21 – 8.15 (m, 2H), 7.29 (t, *J* = 7.4 Hz, 2H), 7.23 – 7.17 (m, 1H), 4.94 – 4.92 (m, 1H), 4.87 (s, 1H), 3.38 (s, 2H), 2.57 – 2.51 (m, 1H), 2.28 – 2.17 (m, 2H), 2.03 – 1.94 (m, 2H), 1.92 – 1.84 (m, 2H), 1.84 – 1.73 (m, 1H), 1.58 (s, 3H), 1.30 (s, 3H).

^{13}**C NMR:** (125 MHz, $CDCl_3$) δ = 211.1, 163.5, 150.6, 144.5, 139.4, 135.6, 130.8, 129.1, 128.4, 126.2, 123.5, 117.4, 83.5, 49.2, 44.8, 42.5, 37.5, 36.0, 25.4, 24.4, 18.4.

IR: *f* (cm^{-1}) = 2934, 1709, 1606, 1527, 1454, 1348, 1320, 1287, 1102, 1010, 864, 720, 701.

HRMS (ESI-TOF): *m/z* $[M+H]^+$ = 422.1969 calculated for $C_{25}H_{28}NO_5$; found 422.1974.

Compound (-)-10d

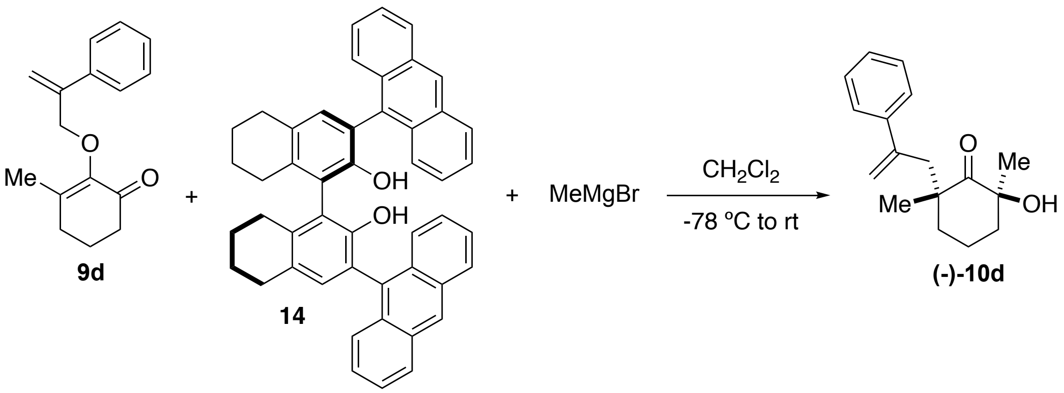

Procedure: Ligand **14** (373 mg, 0.578 mmol) and ketone **9d** (100 mg, 0.413 mmol) were dissolved in CH_2Cl_2 (8.3 mL) in a round bottom flask. After cooling the solution to -78 °C, methylmagnesium bromide (0.54 mL, 1.61 mmol, 3.0 M in Et_2O) was added dropwise. After stirring at -78 °C until the starting material was fully consumed as monitored by TLC, the reaction mixture was warmed to room temperature and stirred until the Claisen rearrangement of the carbonyl addition intermediate was complete as monitored by TLC for a total reaction time of 24 hours. After cooling to 0 °C, the reaction was quenched with a saturated NH_4Cl solution (8 mL) and diluted with DI water (8 mL). The aqueous layer was extracted with CH_2Cl_2 (3 x 10 mL), and the combined organic layers were washed with brine, dried over Na_2SO_4, and concentrated under vacuum. The crude material was purified by column chromatography using 100% hexanes to 50:50 hexanes : CH_2Cl_2 to afford compound **(-)-10d** in 89% yield (95 mg, 0.368 mmol) as a colorless oil. ^{1}H NMR analysis of the crude reaction mixture indicated > 20:1 dr.

Rf: 0.3 in 80:20 hexanes : EtOAc

^{1}H NMR: (400 MHz, $CDCl_3$) δ = 7.36 – 7.28 (m, 4H), 7.27 – 7.24 (m, 1H), 5.30 (d, J = 1.4 Hz, 1H), 5.06 (s, 1H), 3.76 (s, 1H), 2.96 (d, J = 14.0 Hz, 1H), 2.67 (d, J = 14.0 Hz, 1H), 2.05 – 2.00 (m, 1H), 1.86 – 1.81 (m, 1H), 1.76 – 1.68 (m, 2H), 1.66 – 1.60 (m, 1H), 1.41 – 1.34 (m, 4H), 1.01 (s, 3H).

^{13}C NMR: (100 MHz, $CDCl_3$) δ = 217.9, 145.1, 142.9, 128.3, 127.4, 126.5, 117.9, 75.8, 48.3, 41.7, 39.7, 37.1, 27.2, 25.1, 18.0.

IR: f (cm^{-1}) = 3484, 3079, 2934, 2870, 1698, 1623, 1455, 1373, 1153, 1013, 906, 778, 700.

HRMS (ESI-TOF): m/z $[M+H]^+$ = 259.1693 calculated for $C_{17}H_{23}O_2$; found 259.1701.

Specific Rotation: $[\alpha]_{25}^{D}$ = -2.3 (c = 1.00 in $CHCl_3$)

HPLC ((-)-10d)-BzNO$_2$): OD-H, hexane/isopropanol = 95/5, flow rate = 1.0 mL/min, λ = 254 nm, t_R = 12.8 min (major), 17.2 min (minor).

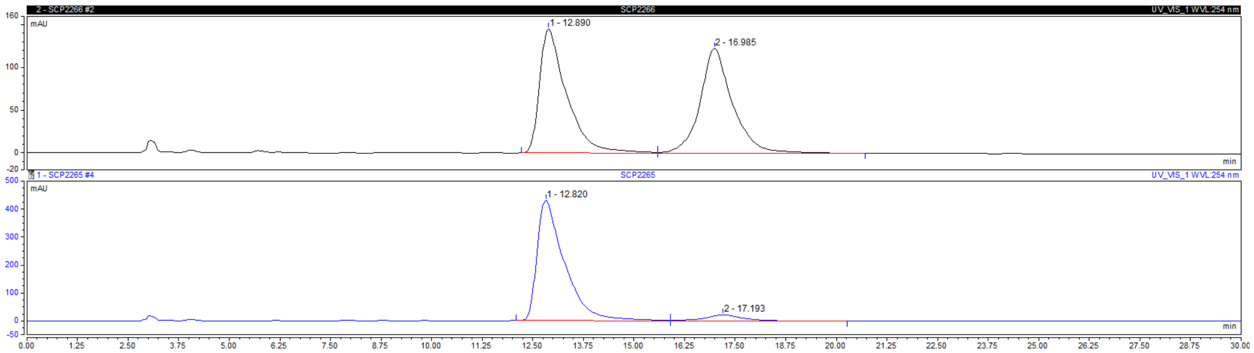

Retention Time (min)	Relative Area (%)	Area (mAU*min)	Height (mAU)
12.820	93.53	339.2754	429.40
17.193	6.47	23.4854	22.13

Compound ((-)-10d)-BzNO$_2$

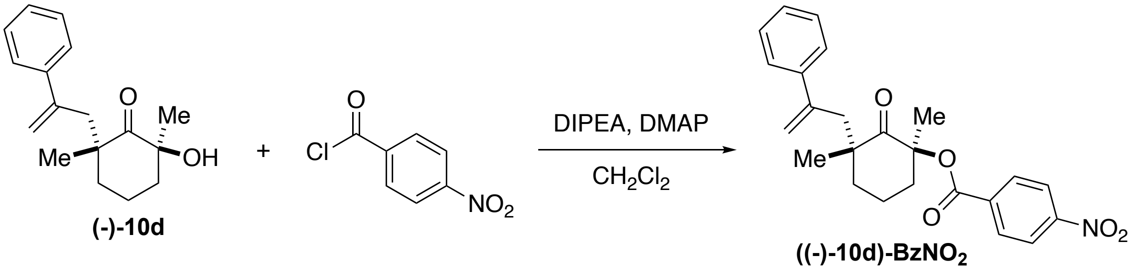

Procedure: Alcohol **(-)-10d** (16 mg, 0.062 mmol) was dissolved in CH_2Cl_2 (0.3 mL). *N*,*N*-Diisopropylethylamine (32 μL, 0.19 mmol) was then added to the solution, followed by 4-nitrobenzoyl chloride (35 mg, 0.19 mmol) and 4-(dimethylamino)pyridine (8 mg, 0.062 mmol). The reaction mixture was then stirred at room temperature until complete consumption of the starting material as monitored by TLC. The crude reaction mixture was concentrated under vacuum and then purified by column chromatography using 100% hexanes to 97:3 hexanes : EtOAc to afford compound **((-)-10d)-BzNO$_2$** in 71% yield (18 mg, 0.044 mmol) as a white solid.

Rf: 0.3 in 90:10 hexanes : EtOAc

^{1}H NMR: (500 MHz, $CDCl_3$) δ = 8.29 – 8.26 (m, 2H), 8.21 – 8.16 (m, 2H), 7.39 – 7.26 (m, 5H), 5.35 (d, *J* = 1.7 Hz, 1H), 5.12 (d, *J* = 1.7 Hz, 1H), 2.89 (d, *J* = 13.5 Hz, 1H), 2.71 (d, *J* = 13.5 Hz, 1H), 2.53 – 2.47 (m, 1H), 2.00 – 1.94 (m, 1H), 1.80 – 1.66 (m, 4H), 1.63 (s, 3H), 1.07 (s, 3H).

^{13}C NMR: (125 MHz, $CDCl_3$) δ = 211.1, 163.4, 150.5, 145.0, 143.1, 135.6, 130.8, 128.3, 127.4, 126.5, 123.4, 118.6, 83.3, 49.5, 42.4, 37.2, 34.9, 25.3, 24.3, 18.2.

IR: *f* (cm^{-1}) = 2936, 2871, 1707, 1606, 1526, 1493, 1460, 1375, 1348, 1320, 1285, 1211, 1167, 1115, 1103, 1009, 863, 780, 720, 707.

HRMS (ESI-TOF): *m/z* $[M+H]^+$ = 408.1813 calculated for $C_{24}H_{26}NO_5$; found 408.1820.

Compound (+)-10e

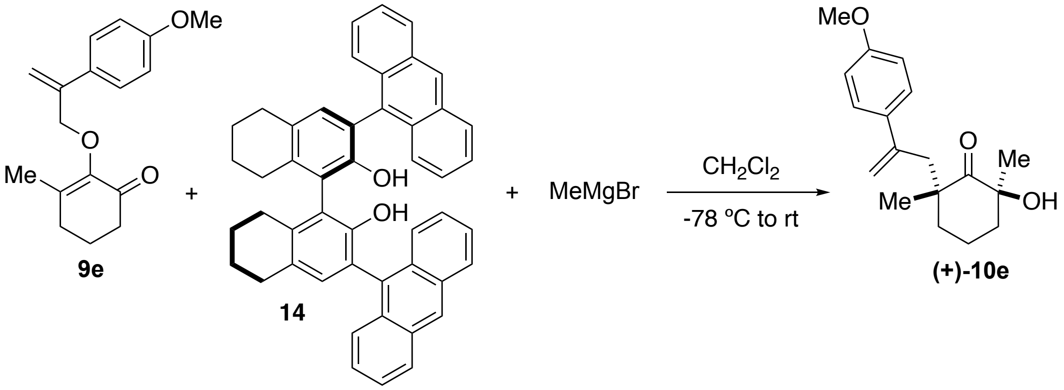

Procedure: Ligand **14** (336 mg, 0.519 mmol) and ketone **9e** (101 mg, 0.371 mmol) were dissolved in CH_2Cl_2 (7.4 mL) in a round bottom flask. After cooling the solution to -78 °C, methylmagnesium bromide (0.48 mL, 1.45 mmol, 3.0 M in Et_2O) was added dropwise. After stirring at -78 °C until the starting material was fully consumed as monitored by TLC, the reaction mixture was warmed to room temperature and stirred until the Claisen rearrangement of the carbonyl addition intermediate was complete as monitored by TLC for a total reaction time of 23 hours. After cooling to 0 °C, the reaction was quenched with a saturated NH_4Cl solution (8 mL) and diluted with DI water (8 mL). The aqueous layer was extracted with CH_2Cl_2 (3 x 10 mL), and the combined organic layers were washed with brine, dried over Na_2SO_4, and concentrated under vacuum. The crude material was purified by column chromatography using 100% hexanes to 50:50 hexanes : CH_2Cl_2 to afford compound **(+)-10e** in 92% yield (98 mg, 0.34 mmol) as a pale yellow oil. ^{1}H NMR analysis of the crude reaction mixture indicated > 20:1 dr.

Rf: 0.4 in 50:50 hexanes : CH_2Cl_2 then 80:20 hexanes : EtOAc

^{1}H NMR: (400 MHz, $CDCl_3$) δ = 7.29 – 7.24 (m, 2H), 6.84 (d, *J* = 8.7 Hz, 2H), 5.23 (d, *J* = 1.5 Hz, 1H), 4.98 (d, *J* = 1.4 Hz, 1H), 3.81 (s, 3H), 3.76 (s, 1H), 2.93 (dd, J = 13.9, 1.0 Hz, 1H), 2.63 (d, *J* = 14.0 Hz, 1H), 2.06 – 1.96 (m, 1H), 1.90 – 1. 81 (m, 1H), 1.78 – 1.57 (m, 3H), 1.39 – 1.33 (m, 1H), 1.36 (s, 3H), 1.01 (s, 3H).

^{13}C NMR: (125 MHz, $CDCl_3$) δ = 218.1, 159.1, 144.4, 135.4, 127.6, 116.6, 113.6, 75.8, 55.3, 48.3, 41.7, 39.7, 37.0, 27.2, 25.1, 18.0.

IR: *f* (cm^{-1}) = 3493, 2930, 1698, 1607, 1510, 1457, 1285, 1244, 1178, 1033, 1012, 900, 835.

HRMS (ESI-TOF): *m/z* $[M+H]^+$ = 289.1803 calculated for $C_{18}H_{25}O_3$; found 289.1798.

Specific Rotation: $[\alpha]_{25}^{D}$ = +10 (c = 1.00 in $CHCl_3$)

HPLC ((+)-10e)-BzNO$_2$: OD-H, hexane/isopropanol = 98/2 to 97/3 over 60 min, flow rate = 1.0 mL/min, λ = 254 nm, t_R = 26.7 min (major), 34.8 min (minor).

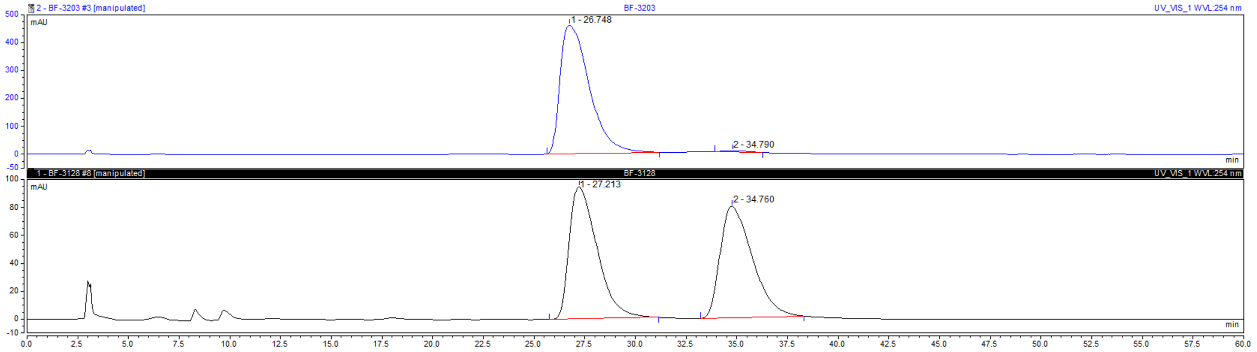

Retention Time (min)	Relative Area (%)	Area (mAU*min)	Height (mAU)
26.748	99.14	768.3022	459.55
34.790	0.86	6.7034	5.06

Compound ((+)-10e)-BzNO$_2$

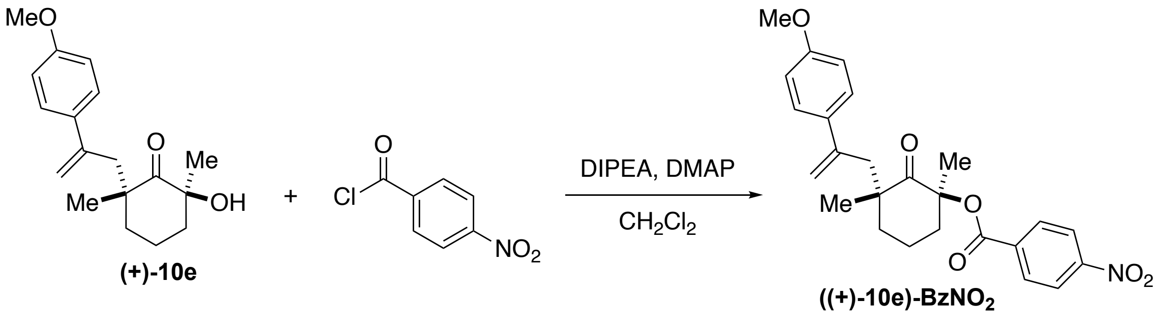

Procedure: Alcohol **(+)-10e** (30 mg, 0.10 mmol) was dissolved in CH_2Cl_2 (0.6 mL). *N*,*N*-Diisopropylethylamine (90 μL, 0.51 mmol) was then added to the solution, followed by 4-nitrobenzoyl chloride (61 mg, 0.33 mmol) and 4-(dimethylamino)pyridine (13 mg, 0.11 mmol). The reaction mixture was then stirred at room temperature until complete consumption of the starting material as monitored by TLC. The crude reaction mixture was concentrated under vacuum and then purified by column chromatography using 100% hexanes to 98:2 hexanes : EtOAc to afford compound **((+)-10e)-BzNO$_2$** in 90% yield (41 mg, 0.094 mmol) as a pale yellow oil.

Rf: 0.5 in 80:20 hexanes : EtOAc

^{1}H NMR: (400 MHz, $CDCl_3$) δ = 8.26 (d, *J* = 8.9 Hz, 2H), 8.16 (d, *J* = 8.9 Hz, 2H), 7.30 (d, *J* = 8.8 Hz, 2H), 6.85 (d, *J* = 8.8 Hz, 2H), 5.26 (d, *J* = 1.7 Hz, 1H), 5.01 (d, *J* = 1.7 Hz, 1H), 3.81 (s, 3H), 2.83 (d, *J* = 13.5 Hz, 1H), 2.64 (d, *J* = 13.5 Hz, 1H), 2.51 – 2.45 (m, 1H), 1.96 (dd, *J* = 13.6, 1.9 Hz, 1H), 1.80 – 1.66 (m, 4H), 1.61 (s, 3H), 1.05 (s, 3H).

^{13}C NMR: (100 MHz, $CDCl_3$) δ = 211.2, 163.5, 159.0, 150.6, 144.4, 135.6, 135.6, 130.8, 127.6, 123.4, 117.2, 113.7, 83.3, 55.3, 49.5, 42.4, 37.3, 34.8, 25.4, 24.4, 18.2.

IR: *f* (cm^{-1}) = 2934, 1708, 1607, 1527, 1511, 1461, 1348, 1320, 1286, 1245, 1180, 1115, 1103, 1033, 1009, 864, 837, 720.

HRMS (ESI-TOF): *m/z* $[M+H]^+$ = 438.1918 calculated for $C_{25}H_{28}NO_6$; found 438.1922.

Compound (+)-10f

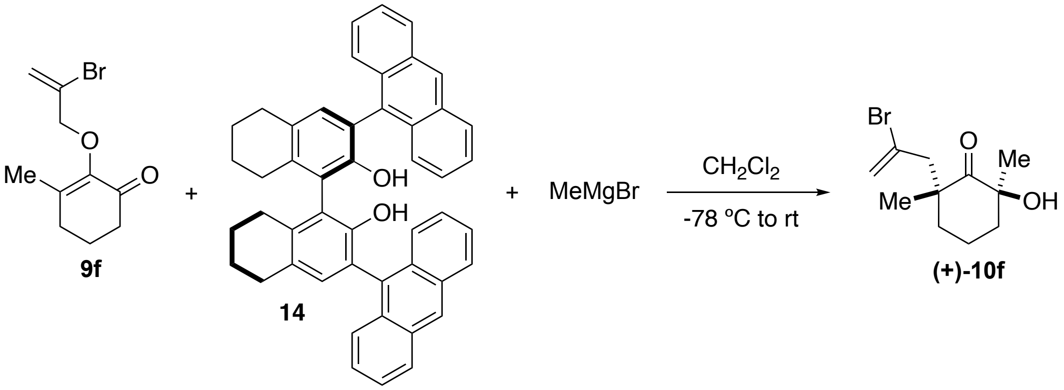

Procedure: Ligand **14** (369 mg, 0.571 mmol) and ketone **9f** (100 mg, 0.408 mmol) were dissolved in CH_2Cl_2 (8.1 mL) in a round bottom flask. After cooling the solution to -78 °C, methylmagnesium bromide (0.53 mL, 1.59 mmol, 3.0 M in Et_2O) was added dropwise. After stirring at -78 °C until the starting material was fully consumed as monitored by TLC, the reaction mixture was warmed to room temperature and stirred until the Claisen rearrangement of the carbonyl addition intermediate was complete as monitored by TLC for a total reaction time of 46 hours. After cooling to 0 °C, the reaction was quenched with a saturated NH_4Cl solution (8 mL) and diluted with DI water (8 mL). The aqueous layer was extracted with CH_2Cl_2 (3 x 10 mL), and the combined organic layers were washed with brine, dried over Na_2SO_4, and concentrated under vacuum. The crude material was purified by column chromatography using 100% hexanes to 50:50 hexanes : CH_2Cl_2 to afford compound **(+)-10f** in 39% yield (42 mg, 0.16 mmol) as a pale brown crystalline solid. ^1H NMR analysis of the crude reaction mixture indicated > 20:1 dr.

Rf: 0.3 in 50:50 hexanes : CH_2Cl_2 then 80:20 hexanes : EtOAc

^{1}H NMR: (500 MHz, $CDCl_3$) δ = 5.57 (s, 2H), 3.58 (s, 1H), 2.88 (d, J = 15.3 Hz, 1H), 2.79 (d, J = 15.3 Hz, 1H), 2.14 – 2.02 (m, 2H), 1.85 – 1.76 (m, 3H), 1.65 – 1.56 (m, 1H), 1.43 (s, 3H), 1.25 (s, 3H).

^{13}C NMR: (125 MHz, $CDCl_3$) δ = 216.6, 128.2, 121.1, 75.9, 48.1, 48.0, 40.2, 38.1, 26.9, 24.8, 18.0.

IR: *f* (cm^{-1}) = 3478, 2925, 2854, 1702, 1624, 1458, 1377, 1164, 1013, 992, 900, 814, 472.

HRMS (ESI-TOF): *m/z* $[M-OH]^+$ = 243.0379 calculated for $C_{11}H_{16}BrO_2$; found 243.0382.

Specific Rotation: $[\alpha]_{25}^{D}$ = +5.6 (c = 1.00 in $CHCl_3$)

HPLC ((+)-10f)-BzNO₂: OD-H, hexane/isopropanol = 80/20, flow rate = 1.0 mL/min, λ = 254 nm, t_R = 7.9 min (major), 12.7 min (minor).

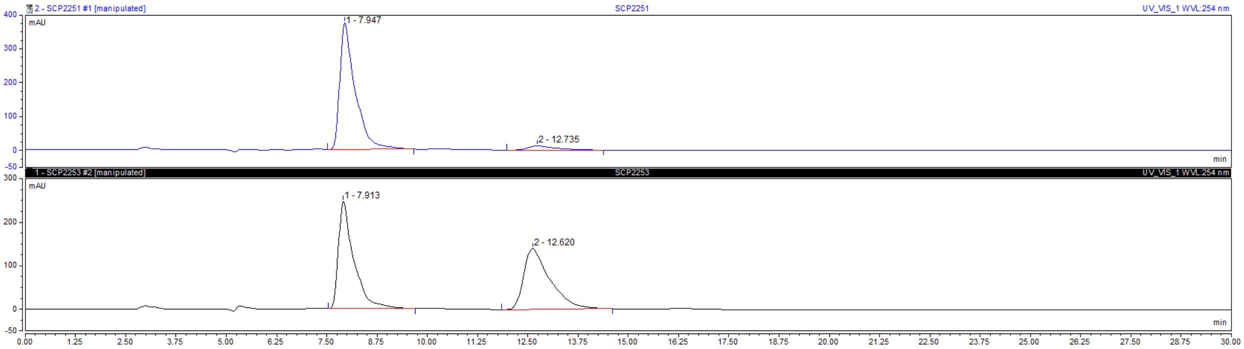

Retention Time (min)	Relative Area (%)	Area (mAU*min)	Height (mAU)
7.947	94.63	165.7626	373.44
12.735	5.37	9.4029	12.21

Compound ((+)-10f)-BzNO₂

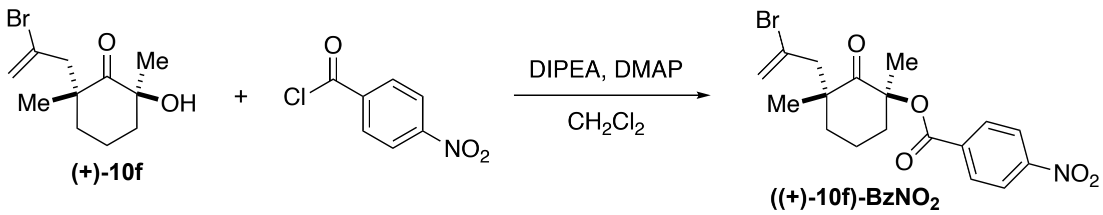

Procedure: Alcohol **(+)-10f** (12 mg, 0.046 mmol) was dissolved in CH_2Cl_2 (0.3 mL). *N,N*-Diisopropylethylamine (24 µL, 0.14 mmol) was then added to the solution, followed by 4-

nitrobenzoyl chloride (25 mg, 0.14 mmol) and 4-(dimethylamino)pyridine (6 mg, 0.046 mmol). The reaction mixture was then stirred at room temperature until complete consumption of the starting material as monitored by TLC. The crude reaction mixture was concentrated under vacuum and then purified by column chromatography using 100% hexanes to 98:2 hexanes : EtOAc to afford compound **((+)-10f)-BzNO$_2$** in 64% yield (12 mg, 0.029 mmol) as a pale yellow oil.

Rf: 0.5 in 80:20 hexanes : EtOAc

^{1}H NMR: (500 MHz, $CDCl_3$) δ = 8.32 – 8.26 (m, 2H), 8.23 – 8.15 (m, 2H), 5.64 (d, *J* = 1.5 Hz, 1H), 5.62 (s, 1H), 2.80 (d, *J* = 14.6 Hz, 1H), 2.68 (d, *J* = 14.6 Hz, 1H), 2.60 – 2.52 (m, 1H), 2.16 – 2.09 (m, 1H), 2.02 – 1.80 (m, 4H), 1.63 (s, 3H), 1.34 (s, 3H).

^{13}C NMR: (125 MHz, $CDCl_3$) δ = 210.1, 163.6, 150.6, 135.5, 130.8, 127.4, 123.5, 122.3, 83.5, 49.3, 48.2, 37.5, 35.3, 25.0, 23.6, 18.2.

IR: *f* (cm^{-1}) = 2937, 1710, 1621, 1607, 1526, 1461, 1376, 1348, 1320, 1285, 1167, 1115, 1102, 1009, 864, 720.

HRMS (ESI-TOF): *m/z* $[M+H]^+$ = 412.0598 calculated for $C_{18}H_{21}Br^{[81]}NO_5$; found for 412.0582.

Compound (-)-12a

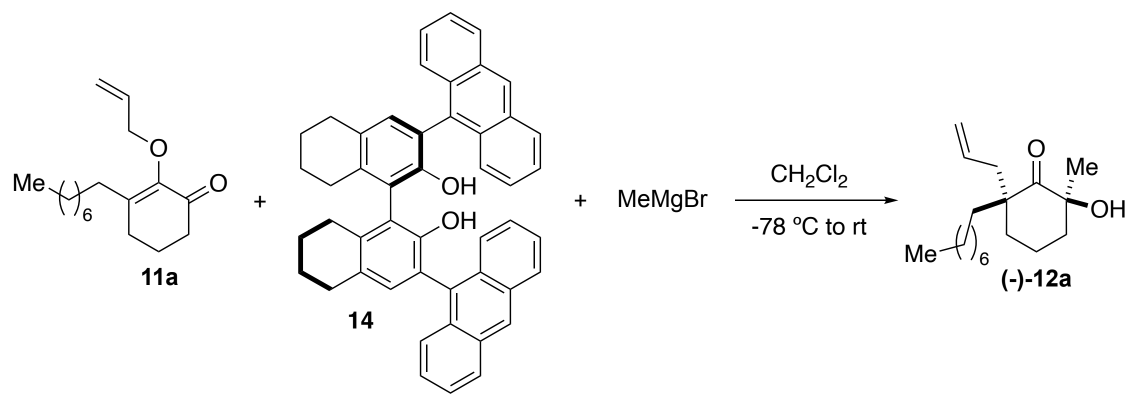

Procedure: Ligand **14** (342 mg, 0.529 mmol) and ketone **11a** (100 mg, 0.378 mmol) were dissolved in CH_2Cl_2 (5.0 mL) in a round bottom flask. After cooling the solution to -78 °C, methylmagnesium bromide (0.52 mL, 1.56 mmol, 3.0 M in Et_2O) was added dropwise. After stirring at -78 °C until the starting material was fully consumed as monitored by TLC, the reaction mixture was warmed to room temperature and stirred until the Claisen rearrangement of the carbonyl addition intermediate was complete as monitored by TLC for a total reaction time of 20 hours. After cooling to 0 °C, the reaction was quenched with a saturated NH_4Cl solution (8 mL) and diluted with DI water (8 mL). The aqueous layer was extracted with CH_2Cl_2 (3 x 10 mL), and the combined organic layers were washed with brine, dried over Na_2SO_4, and concentrated under vacuum. The crude material was purified by column chromatography using 100% hexanes to 50:50 hexanes : CH_2Cl_2 to afford compound **(-)-12a** in 62% yield (66 mg, 0.21 mmol) as a pale yellow oil. ^{1}H NMR analysis of the crude reaction mixture indicated > 20:1 dr.

Rf: 0.5 in 50:50 hexanes : CH_2Cl_2 then 80:20 hexanes : EtOAc

^{1}H NMR: (400 MHz, $CDCl_3$) δ = 5.59 – 5.48 (m, 1H), 5.10 – 4.97 (m, 2H), 3.23 (s, 1H), 2.46 (dd, *J* = 14.4, 7.0 Hz, 1H), 2.21 (dd, *J* = 14.4, 7.6 Hz, 1H), 2.01 (ddt, *J* = 9.6, 4.2, 2.5 Hz, 1H), 1.82 – 1.63 (m, 5H), 1.54 (qdd, *J* = 13.7, 10.6, 5.2 Hz, 2H), 1.36 (s, 3H), 1.24 (s, 11H), 1.14 (tdd, *J* = 10.6, 4.9, 3.2 Hz, 2H), 0.91 – 0.82 (m, 3H).

^{13}C NMR: (100 MHz, $CDCl_3$) δ = 216.9, 133.2, 118.3, 75.6, 50.8, 40.4, 39.5, 36.1, 35.2, 31.8, 30.3, 29.5, 29.3, 27.1, 23.6, 22.6, 18.0, 14.1.

IR: *f* (cm^{-1}) = 3452, 2925, 2855, 1706, 1495, 1459, 1376, 1123, 999, 913, 702, 478, 430.

HRMS (ESI-TOF): *m/z* $[M+Na]^+$ = 303.2295 calculated for $C_{18}H_{32}O_2Na$; found for 303.2283.

Specific Rotation: $[\alpha]_{25}^{D}$ = -5.1 (c = 1.00 in $CHCl_3$)

HPLC ((-)-12a)-BzNO₂: OD-H, hexane/isopropanol = 95/5, flow rate = 1.0 mL/min, λ = 254 nm, t_R = 4.8 min (major), 6.5 min (minor).

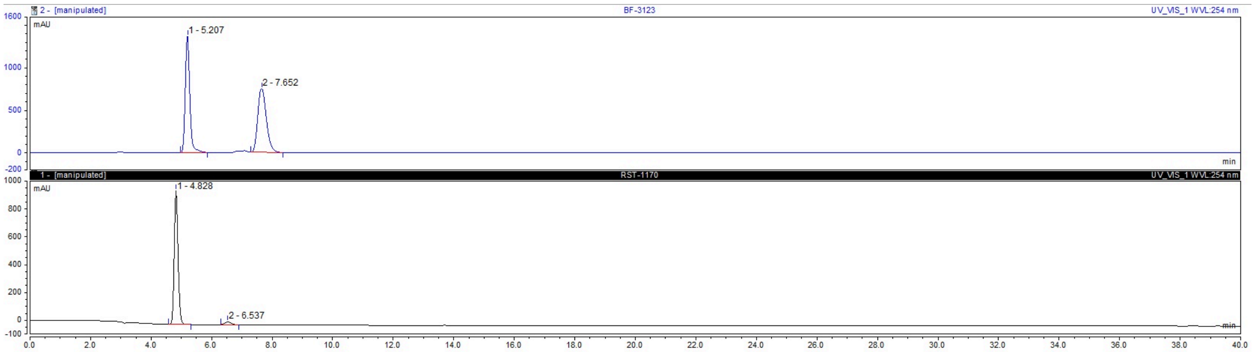

Retention Time (min)	Relative Area (%)	Area (mAU*min)	Height (mAU)
4.828	96.44	140.8729	960.37
6.537	3.56	5.1996	21.79

Compound ((-)-12a)-BzNO₂

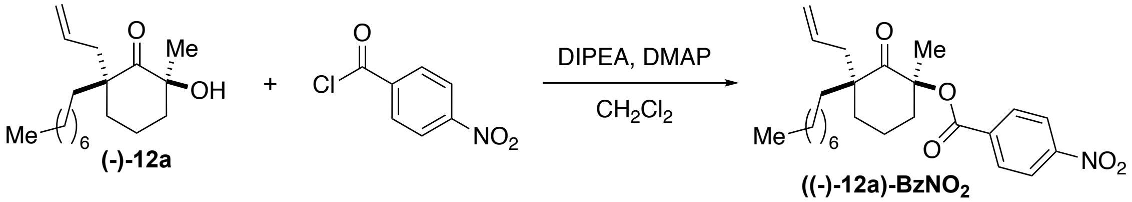

Procedure: Alcohol **(-)-12a** (40 mg, 0.14 mmol) was dissolved in CH_2Cl_2 (0.8 mL). *N*,*N*-Diisopropylethylamine (0.12 mL, 0.69 mmol) was then added to the solution, followed by 4-nitrobenzoyl chloride (84 mg, 0.45 mmol) and 4-(dimethylamino)pyridine (17 mg, 0.14 mmol). The reaction mixture was then stirred at room temperature until complete consumption of the starting material as monitored by TLC. The crude reaction mixture was concentrated under vacuum and then purified by column chromatography using 100% hexanes to 98:2 hexanes :

EtOAc to afford compound **((-)-12a)-BzNO₂** in 87% yield (52 mg, 0.12 mmol) as a pale yellow oil.

Rf: 0.6 in 90:10 hexanes : EtOAc

^{1}H NMR: (400 MHz, $CDCl_3$) δ = 8.26 (d, *J* = 8.5 Hz, 2H), 8.17 (d, *J* = 8.4 Hz, 2H), 5.66 (ddt, *J* = 17.2, 10.3, 7.3 Hz, 1H), 5.05 (dd, *J* = 18.1, 13.6 Hz, 2H), 2.63 (td, *J* = 12.0, 5.2 Hz, 1H), 2.39 (dd, *J* = 14.4, 7.0 Hz, 1H), 2.24 (dd, *J* = 14.4, 7.5 Hz, 1H), 1.99 (ddt, *J* = 19.3, 8.8, 4.2 Hz, 2H), 1.87 (dtd, *J* = 14.6, 10.9, 10.1, 4.2 Hz, 2H), 1.79 – 1.71 (m, 1H), 1.69 – 1.54 (m, 5H), 1.26 (d, *J* = 8.3 Hz, 13H), 0.87 (t, *J* = 6.7 Hz, 3H).

^{13}C NMR: (100 MHz, $CDCl_3$) δ = 209.3, 163.2, 150.5, 135.9, 133.3, 130.8, 123.4, 118.1, 83.8, 51.7, 40.7, 36.6, 36.0, 33.5, 31.9, 30.3, 29.5, 29.3, 25.1, 23.0, 22.7, 18.2, 14.1.

IR: *f* (cm^{-1}) =2934, 1708, 1702, 1607, 1511, 1461, 1348, 1245, 1180, 1115, 1009, 864, 720.

HRMS (ESI-TOF): *m/z* $[M+H]^+$ = 430.2588 calculated for $C_{25}H_{36}NO_5$; found for 430.2597.

Compound (+)-12b

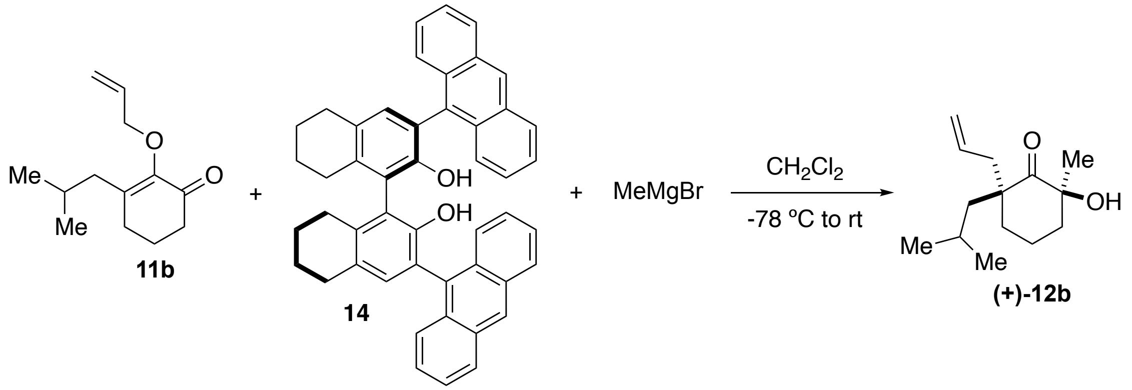

Procedure: Ligand **14** (521 mg, 0.807 mmol) and ketone **11b** (120 mg, 0.576 mmol) were dissolved in CH_2Cl_2 (11.5 mL) in a round bottom flask. After cooling the solution to -78 °C, methylmagnesium bromide (0.75 mL, 2.25 mmol, 3.0 M in Et_2O) was added dropwise. After stirring at -78 °C until the starting material was fully consumed as monitored by TLC, the reaction

mixture was warmed to room temperature and stirred until the Claisen rearrangement of the carbonyl addition intermediate was complete as monitored by TLC for a total reaction time of 44 hours. After cooling to 0 °C, the reaction was quenched with a saturated NH_4Cl solution (8 mL) and diluted with DI water (8 mL). The aqueous layer was extracted with CH_2Cl_2 (3 x 10 mL), and the combined organic layers were washed with brine, dried over Na_2SO_4, and concentrated under vacuum. The crude material was purified by column chromatography using 100% hexanes to 50:50 hexanes : CH_2Cl_2 to afford compound **(+)-12b** in 68% yield (88 mg, 0.392 mmol) as a pale yellow oil. ^1H NMR analysis of the crude reaction mixture indicated > 20:1 dr.

Rf: 0.5 in 50:50 hexanes : CH_2Cl_2 then 80:20 hexanes : EtOAc

^{1}H NMR: (400 MHz, $CDCl_3$) δ = 5.56 (ddt, *J* = 17.2, 10.2, 7.2 Hz, 1H), 5.11 – 4.92 (m, 2H), 3.36 (s, 1H), 2.45 (dd, *J* = 14.4, 7.0 Hz, 1H), 2.19 (dd, *J* = 14.4, 7.5 Hz, 1H), 2.03 (ddt, *J* = 9.6, 4.0, 1.9 Hz, 1H), 1.87 – 1.68 (m, 6H), 1.55 (dqd, *J* = 13.3, 6.6, 4.9 Hz, 1H), 1.35 (s, 3H), 1.31 (d, *J* = 5.0 Hz, 1H), 0.86 (dd, *J* = 23.0, 6.6 Hz, 7H).

^{13}C NMR: (100 MHz, $CDCl_3$) δ = 217.0, 133.2, 118.4, 51.3, 44.3, 41.8, 39.1, 34.3, 27.6, 25.0, 24.5, 24.4, 18.0.

IR: *f* (cm^{-1}) =3440, 2928, 1700, 1490, 1459, 1376, 1123, 999, 913, 702.

HRMS (ESI-TOF): *m/z* $[M+H]^+$ = 225.1849 calculated for $C_{14}H_{25}O_2$; found for 225.1848.

Specific Rotation: $[\alpha]_{25}^{D}$ = +5.8 (c = 1.00 in $CHCl_3$)

HPLC ((+)-12b)-BzNO$_2$: OD-H, hexane/isopropanol = 95/5, flow rate = 1.0 mL/min, λ = 254 nm, t_R = 5.7 min (major), 7.7 min (minor).

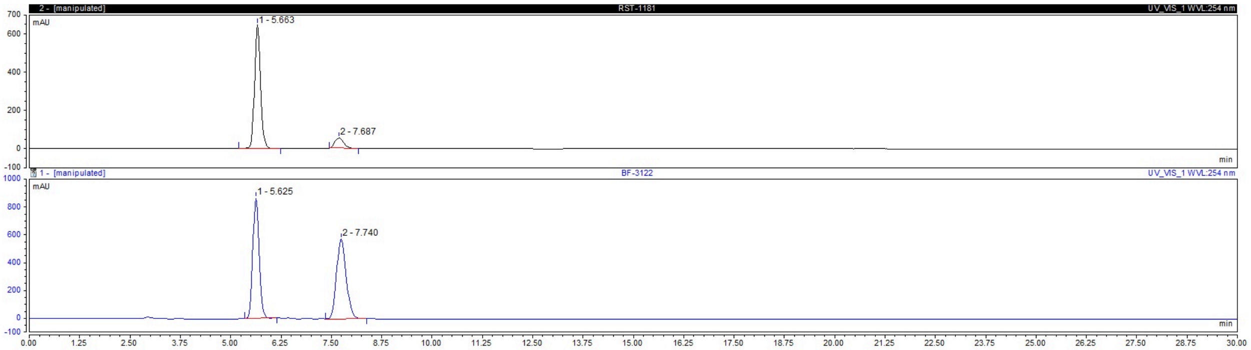

Retention Time (min)	Relative Area (%)	Area (mAU*min)	Height (mAU)
5.663	89.01	112.7005	647.13
7.687	10.99	13.9182	53.22

Compound ((+)-12b)-BzNO$_2$

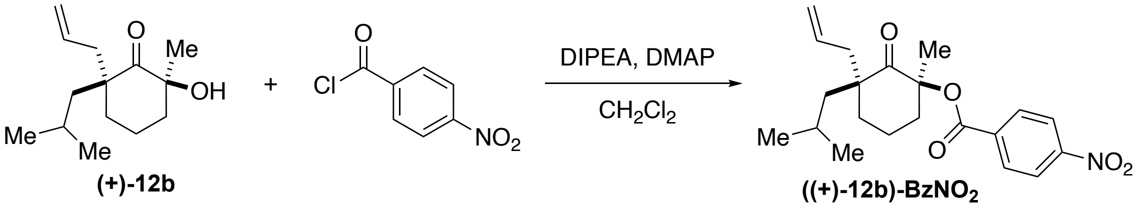

Procedure: Alcohol **(+)-12b** (30 mg, 0.13 mmol) was dissolved in CH_2Cl_2 (0.8 mL). *N*,*N*-Diisopropylethylamine (0.12 mL, 0.68 mmol) was then added to the solution, followed by 4-nitrobenzoyl chloride (81 mg, 0.43 mmol) and 4-(dimethylamino)pyridine (16 mg, 0.13 mmol). The reaction mixture was then stirred at room temperature until complete consumption of the starting material as monitored by TLC. The crude reaction mixture was concentrated under vacuum and then purified by column chromatography using 100% hexanes to 98:2 hexanes : EtOAc to afford compound **((+)-12b)-BzNO$_2$** in 72% yield (36 mg, 0.096 mmol) as a pale yellow oil.

Rf: 0.3 in 90:10 hexanes : EtOAc

^{1}H NMR: (400 MHz, $CDCl_3$) δ = 8.25 (d, *J* = 8.9 Hz, 2H), 8.16 (d, *J* = 8.9 Hz, 2H), 5.66 (ddt, *J* = 17.2, 10.2, 7.2 Hz, 1H), 5.11 – 4.97 (m, 2H), 2.69 – 2.59 (m, 1H), 2.39 (dd, *J* = 14.5, 7.0 Hz, 1H), 2.28 (dd, *J* = 14.4, 7.4 Hz, 1H), 2.16 – 1.97 (m, 2H), 1.92 – 1.71 (m, 5H), 1.62 (s, 3H), 1.49 – 1.42 (m, 1H), 0.94 (dd, *J* = 6.6, 2.5 Hz, 6H).

^{13}C NMR: (100 MHz, $CDCl_3$) δ = 209.1, 163.2, 150.5, 136.0, 133.4, 130.8, 123.4, 118.2, 83.8, 52.8, 44.5, 41.3, 36.3, 33.3, 25.7, 25.5, 25.4, 23.5, 18.3.

IR: *f* (cm^{-1}) =2940, 1710, 1695, 1607, 1511, 1461, 1348, 1245, 1180, 1115, 1009, 864, 720.

HRMS (ESI-TOF): *m/z* $[M+H]^+$ =374.1962 calculated for $C_{21}H_{28}NO_5$; found for 374.1976.

Compound (-)-12c

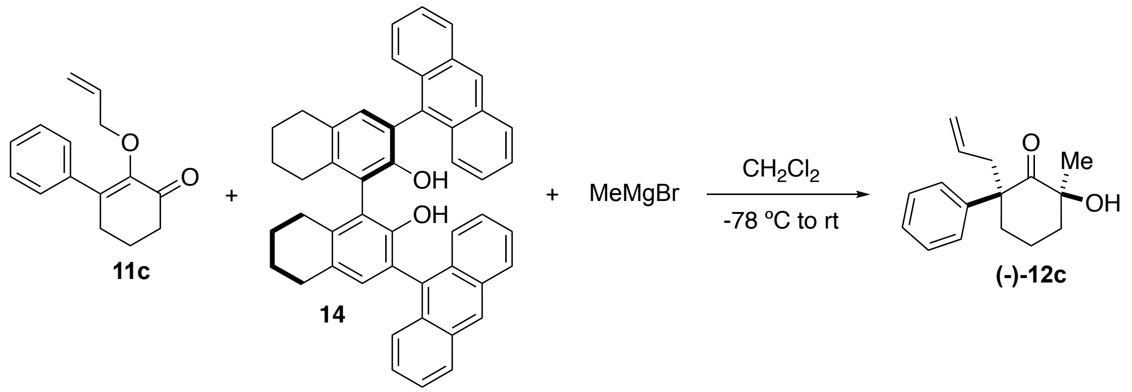

Procedure: Ligand **14** (223 mg, 0.345 mmol) and ketone **11c** (56 mg, 0.25 mmol) were dissolved in CH_2Cl_2 (4.9 mL) in a round bottom flask. After cooling the solution to -78 °C, methylmagnesium bromide (0.31 mL, 0.93 mmol, 3.0 M in Et_2O) was added dropwise. After stirring at -78 °C until the starting material was fully consumed as monitored by TLC, the reaction mixture was warmed to room temperature and stirred until the Claisen rearrangement of the carbonyl addition intermediate was complete as monitored by TLC for a total reaction time of 44 hours. After cooling to 0 °C, the reaction was quenched with a saturated NH_4Cl solution (8 mL) and diluted with DI water (8 mL). The aqueous layer was extracted with CH_2Cl_2 (3 x 10 mL), and

the combined organic layers were washed with brine, dried over Na_2SO_4, and concentrated under vacuum. The crude material was purified by column chromatography using 100% hexanes to 50:50 hexanes : CH_2Cl_2 to afford compound **(-)-12c** in 83% yield (50 mg, 0.20 mmol) as a pale yellow oil. ^{1}H NMR analysis of the crude reaction mixture indicated > 20:1 dr.

Rf: 0.4 in 50:50 hexanes : CH_2Cl_2 then 80:20 hexanes : EtOAc

^{1}H NMR: (400 MHz, $CDCl_3$) δ = 7.38 – 7.30 (m, 2H), 7.29 – 7.21 (m, 3H), 5.39 (ddt, *J* = 17.3, 10.3, 7.3 Hz, 1H), 4.98 – 4.88 (m, 2H), 2.62 – 2.49 (m, 3H), 2.08 – 1.88 (m, 2H), 1.88 – 1.77 (m, 2H), 1.76-1.65 (m, 2H), 1.32 (s, 3H).

^{13}C NMR: (125 MHz, $CDCl_3$) δ = 212.8, 139.9, 133.8, 128.7, 127.0, 126.2, 118.1, 76.2, 55.0, 45.5, 37.8, 31.8, 25.6, 16.8.

IR: *f* (cm^{-1}) = 3452, 2925, 2855, 1706, 1495, 1459, 1376, 1123, 999, 913, 702, 478, 430.

HRMS (ESI-TOF): *m/z* $[M+H]^+$ = 245.1536 calculated for $C_{16}H_{21}O_2$; found 245.1538.

Specific Rotation: $[\alpha]_{25}^{D}$ = -56.8 (c = 1.00 in $CHCl_3$)

HPLC ((-)-12c)-BzNO$_2$: OD-H, hexane/isopropanol = 99.5/0.5 to 97/3 over 60 min, flow rate = 1.0 mL/min, λ = 254 nm, t_R = 18.1 min (minor), 22.7 min (major).

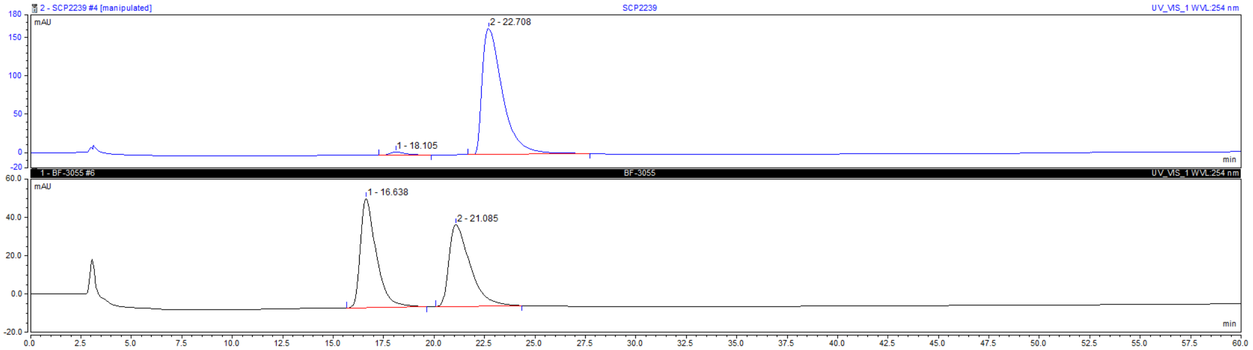

Retention Time (min)	Relative Area (%)	Area (mAU*min)	Height (mAU)
18.105	1.73	3.3723	3.99
22.708	98.27	191.2900	164.29

Compound ((-)-12c)-BzNO$_2$

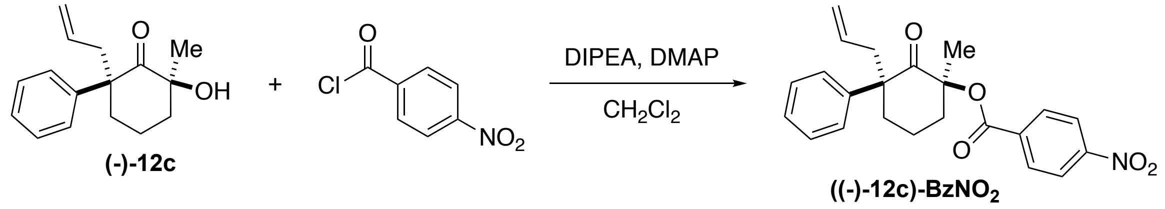

Procedure: Alcohol **(-)-12c** (25 mg, 0.10 mmol) was dissolved in CH_2Cl_2 (0.6 mL). *N*,*N*-Diisopropylethylamine (53 μL, 0.31 mmol) was then added to the solution, followed by 4-nitrobenzoyl chloride (57 mg, 0.31 mmol) and 4-(dimethylamino)pyridine (12 mg, 0.10 mmol). The reaction mixture was then stirred at room temperature until complete consumption of the starting material as monitored by TLC. The crude reaction mixture was concentrated under vacuum and then purified by column chromatography using 100% hexanes to 98:2 hexanes : EtOAc to afford compound **((-)-12c)-BzNO$_2$** in 82% yield (33 mg, 0.084 mmol) as a colorless oil.

Rf: 0.6 in 80:20 hexanes : EtOAc

^{1}H NMR: (500 MHz, $CDCl_3$) δ = 8.04 – 7.99 (m, 2H), 7.49 – 7.44 (m, 2H), 7.21 – 7.17 (m, 2H), 7.04 (t, *J* = 7.8 Hz, 2H), 6.76 (t, *J* = 7.4 Hz, 1H), 5.25 – 5.14 (m, 1H), 4.91 – 4.85 (m, 2H), 2.78 – 2.63 (m, 3H), 2.48 (dd, *J* = 14.1, 8.2 Hz, 1H), 2.36 – 2.24 (m, 1H), 1.83 – 1.71 (m, 3H), 1.70 (s, 3H).

^{13}C NMR: (125 MHz, $CDCl_3$) δ = 205.1, 162.8, 150.1, 138.9, 135.0, 133.5, 130.4, 128.3, 126.7, 126.2, 122.6, 118.1, 85.2, 55.6, 46.8, 40.4, 34.8, 21.6, 17.7.

IR: *f* (cm^{-1}) = 2930, 2869, 1731, 1607, 1525, 1495, 1446, 1376, 1348, 1320, 1278, 1180, 1096, 1015, 801, 717, 703.

HRMS (ESI-TOF): *m/z* $[M+H]^+$ =394.1649 calculated for $C_{23}H_{24}NO_5$; found 394.1666.

X-Ray Structure: The ellipsoid contour was set at a 50% probability level.

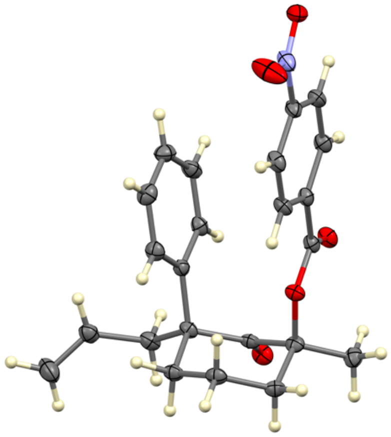

Compound (-)-12d

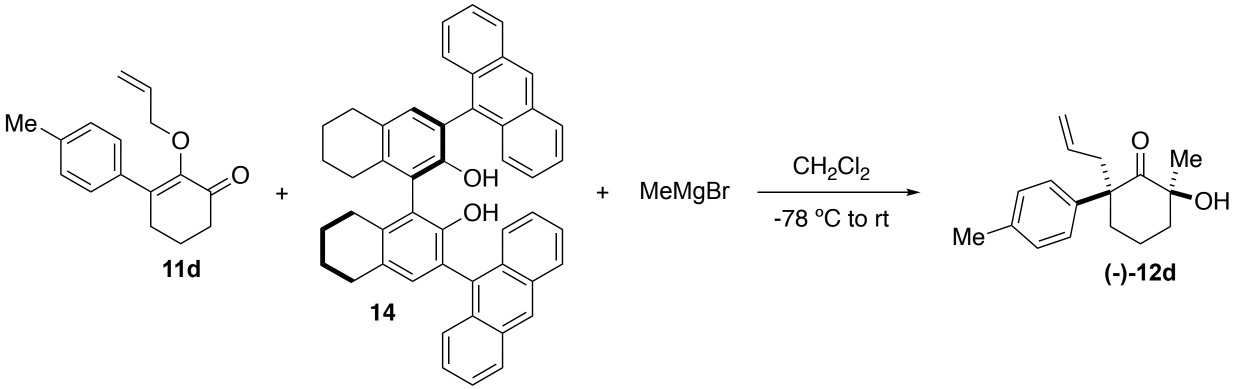

Procedure: Ligand **14** (262 mg, 0.40 mmol) and ketone **11d** (70 mg, 0.29 mmol) were dissolved in CH_2Cl_2 (5.8 mL) in a round bottom flask. After cooling the solution to -78 °C, methylmagnesium bromide (0.37 mL, 1.13 mmol, 3.0 M in Et_2O) was added dropwise. After stirring at -78 °C until the starting material was fully consumed as monitored by TLC, the reaction mixture was warmed to room temperature and stirred until the Claisen rearrangement of the carbonyl addition intermediate was complete as monitored by TLC for a total reaction time of 21 hours. After cooling to 0 °C, the reaction was quenched with a saturated NH_4Cl solution (5 mL) and diluted with DI water (5 mL). The aqueous layer was extracted with CH_2Cl_2 (3 x 10 mL), and

the combined organic layers were washed with brine, dried over Na_2SO_4, and concentrated under vacuum. The crude material was purified by column chromatography using 100% hexanes to 50:50 hexanes : CH_2Cl_2 to afford compound **(-)-12d** in 74% yield (55 mg, 0.21 mmol) as a pale yellow oil. ^{1}H NMR analysis of the crude reaction mixture indicated > 20:1 dr.

Rf: in 0.4 in 50:50 hexanes : CH_2Cl_2 then 80:20 hexanes : EtOAc

^{1}H NMR: (400 MHz, $CDCl_3$) δ = 7.15 (s, 4H), 5.39 (ddt, *J* = 17.4, 10.4, 7.3 Hz, 1H), 4.98 – 4.89 (m, 2H), 3.61 (b s, 1H), 2.59 – 2.46 (m, 3H), 2.32 (s, 3H), 2.06 – 1.95 (m, 1H), 1.87 – 1.77 (m, 2H), 1.75 – 1.65 (m, 2H), 1.31 (s, 3H).

^{13}C NMR: (100 MHz, $CDCl_3$) δ = 212.9, 136.8, 136.7, 134.0, 129.5, 126.1, 118.0, 76.3, 54.6, 45.5, 37.9, 31.9, 25.5, 20.9, 16.8.

IR: *f* (cm^{-1}) = 3485, 2926, 1705, 1639, 1513, 1460, 1375, 1125, 984, 914, 859, 740, 677, 535.

HRMS (ESI-TOF): *m/z* $[M-OH]^+$ = 241.1594 calculated for $C_{17}H_{21}O$; found 241.1591.

Specific Rotation: $[\alpha]_{25}^{D}$ = -20.6 (c = 1.00 in $CHCl_3$)

HPLC ((-)-12d)-BzNO$_2$: (S,S)-Whelk-O1, hexane/isopropanol = 97/3, flow rate = 1.0 mL/min, λ = 254 nm, t_R = 13.4 min (major), 18.3 min (minor).

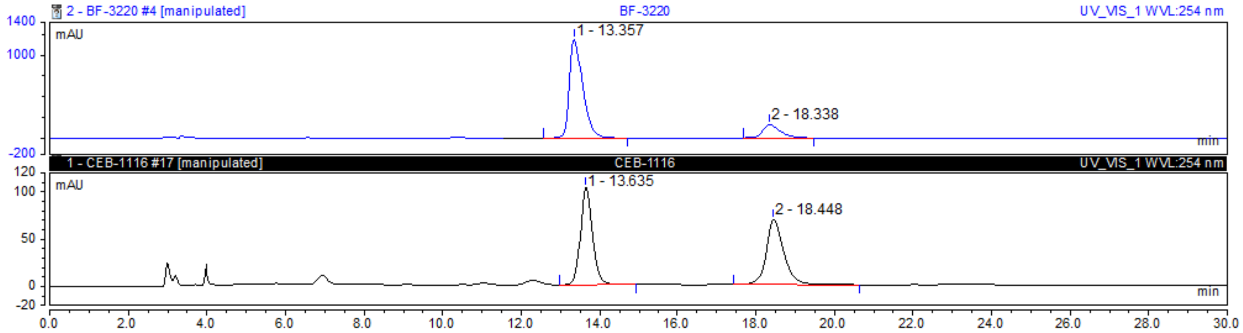

Retention Time (min)	Relative Area (%)	Area (mAU*min)	Height (mAU)
13.357	84.34	449.8726	1196.75
18.338	15.66	83.5379	160.13

Compound ((-)-12d)-BzNO$_2$

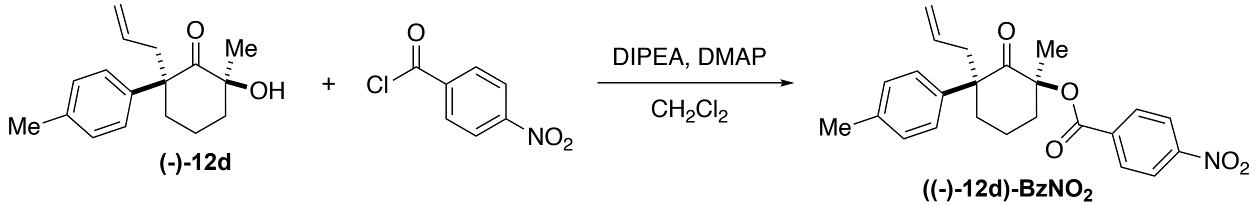

Procedure: Alcohol **(-)-12d** (29 mg, 0.11 mmol) was dissolved in CH_2Cl_2 (0.6 mL). *N*,*N*-Diisopropylethylamine (0.10 mL, 0.57 mmol) was then added to the solution, followed by 4-nitrobenzoyl chloride (68 mg, 0.36 mmol) and 4-(dimethylamino)pyridine (14 mg, 0.11 mmol). The reaction mixture was then stirred at room temperature until complete consumption of the starting material as monitored by TLC. The crude reaction mixture was concentrated under vacuum and then purified by column chromatography using 100% hexanes to 98:2 hexanes : EtOAc to afford compound **((-)-12d)-BzNO$_2$** in 94% yield (43 mg, 0.11 mmol) as a pale yellow oil.

Rf: 0.6 in 80:20 hexanes : EtOAc

^{1}H NMR: (400 MHz, $CDCl_3$) δ = 8.07 – 7.99 (m, 2H), 7.57 – 7.49 (m, 2H), 7.05 (d, *J* = 8.2 Hz, 2H), 6.80 (d, *J* = 8.0 Hz, 2H), 5.27 – 5.14 (m, 1H), 4.93 – 4.85 (m, 2H), 2.77 – 2.58 (m, 3H), 2.45 (dd, *J* = 14.1, 8.3 Hz, 1H), 2.39 – 2.25 (m, 1H), 1.88 (s, 3H), 1.81 – 1.70 (m, 3H), 1.69 (s, 3H).

^{13}C NMR: (100 MHz, $CDCl_3$) δ = 205.2, 162.8, 150.1, 135.9, 135.0, 133.7, 130.5, 129.0, 126.7, 122.5, 117.9, 85.1, 55.2, 46.7, 40.8, 34.9, 21.6, 20.5, 17.7.

IR: *f* (cm^{-1}) =2936, 1729, 1703, 1610, 1522, 1490, 1376, 1320, 1180, 1096, 1015, 717, 703.

HRMS (ESI-TOF): *m/z* $[M+Na]^+$ =430.1630 calculated for $C_{24}H_{25}NO_5Na$; found 430.1692.

Compound (-)-12e

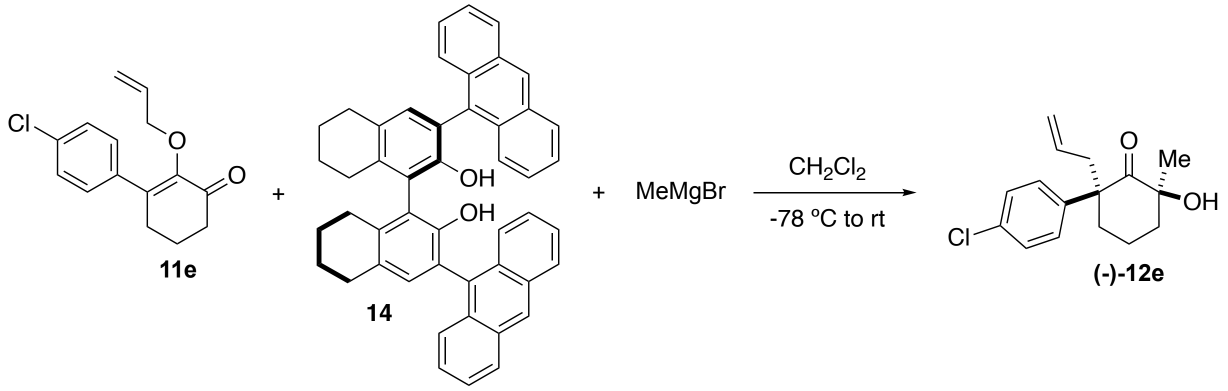

Procedure: Ligand **14** (344 mg, 0.532 mmol) and ketone **11e** (102 mg, 0.388 mmol) were dissolved in CH_2Cl_2 (8.0 mL) in a round bottom flask. After cooling the solution to -78 °C, methylmagnesium bromide (0.5 mL, 1.51 mmol, 3.0 M in Et_2O) was added dropwise. After stirring at -78 °C until the starting material was fully consumed as monitored by TLC, the reaction mixture was warmed to room temperature and stirred until the Claisen rearrangement of the carbonyl addition intermediate was complete as monitored by TLC for a total reaction time of 23 hours. After cooling to 0 °C, the reaction was quenched with a saturated NH_4Cl solution (8 mL) and diluted with DI water (8 mL). The aqueous layer was extracted with CH_2Cl_2 (3 x 10 mL), and the combined organic layers were washed with brine, dried over Na_2SO_4, and concentrated under vacuum. The crude material was purified by column chromatography using 100% hexanes to 50:50 hexanes : CH_2Cl_2 to afford compound **(-)-12e** in 61% yield (66 mg, 0.24 mmol) as a pale yellow oil. ^{1}H NMR analysis of the crude reaction mixture indicated > 20:1 dr.

Rf: 0.4 in 80:20 hexanes : EtOAc

^{1}H NMR: (400 MHz, $CDCl_3$) δ = 7.33 – 7.28 (m, 2H), 7.21 – 7.16 (m, 2H), 5.42 – 5.29 (m, 1H), 5.00 – 4.88 (m, 2H), 2.60 – 2.43 (m, 3H), 1.99 – 1.94 (m, 2H), 1.88 – 1.77 (m, 2H), 1.77 – 1.67 (m, 2H), 1.33 (s, 3H).

^{13}C NMR: (125 MHz, $CDCl_3$) δ = 212.6, 138.8, 133.4, 132.7, 128.8, 127.7, 118.5, 76.1, 54.6, 45.3, 37.7, 31.9, 25.7, 16.8.

IR: f (cm^{-1}) = 3496, 3075, 2934, 1706, 1640, 1493, 1461, 1401, 1373, 1097, 1013, 917, 824, 736, 533.

HRMS (ESI-TOF): *m/z* $[M+H]^+$ = 279.1146 calculated for $C_{16}H_{20}ClO_2$; found 279.1148.

Specific Rotation: $[\alpha]_{25}^{D}$ = -13.5 (c = 1.00 in $CHCl_3$)

HPLC ((-)-12e)-BzNO$_2$: OD-H, hexane/isopropanol = 95/5, flow rate = 1.0 mL/min, λ = 254 nm, t_R = 8.8 min (major), 10.3 min (minor).

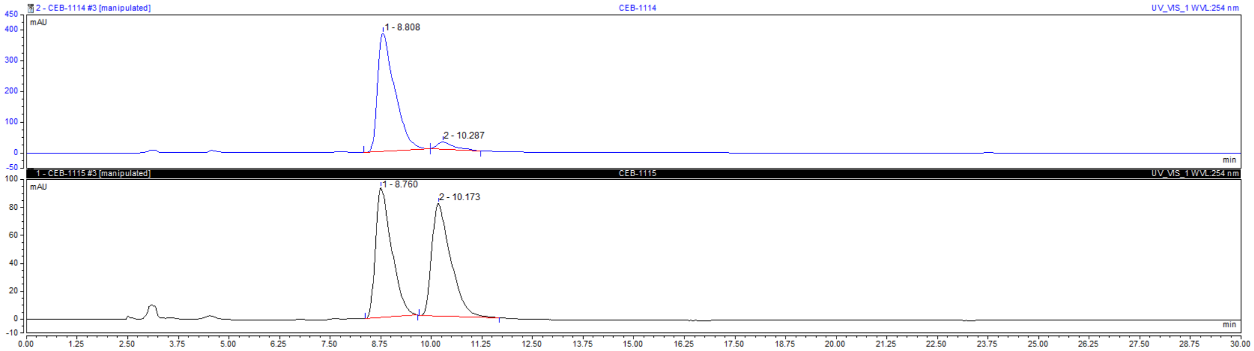

Retention Time (min)	Relative Area (%)	Area (mAU*min)	Height (mAU)
8.808	94.16	188.7500	383.06
10.287	5.84	11.7022	23.31

Compound ((-)-12e)-BzNO₂

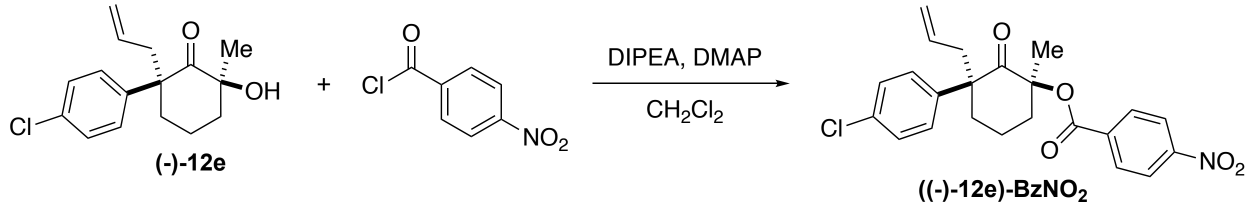

Procedure: Alcohol **(-)-12e** (21 mg, 0.075 mmol) was dissolved in CH_2Cl_2 (0.5 mL). *N*,*N*-Diisopropylethylamine (70 μL, 0.40 mmol) was then added to the solution, followed by 4-nitrobenzoyl chloride (45 mg, 0.24 mmol) and 4-(dimethylamino)pyridine (9 mg, 0.075 mmol). The reaction mixture was then stirred at room temperature until complete consumption of the starting material as monitored by TLC. The crude reaction mixture was concentrated under vacuum and then purified by column chromatography using 100% hexanes to 98:2 hexanes : EtOAc to afford compound **((-)-12e)-BzNO$_2$** in 99% yield (32 mg, 0.074 mmol) as a pale yellow oil.

Rf: 0.4 in 80:20 hexanes : EtOAc

^{1}H NMR: (400 MHz, $CDCl_3$) δ = 8.11 (d, *J* = 8.8 Hz, 2H), 7.58 (d, *J* = 8.8 Hz, 2H), 7.09 (d, *J* = 8.6 Hz, 2H), 6.95 (d, *J* = 8.6 Hz, 2H), 5.25 – 5.12 (m, 1H), 4.93 – 4.84 (m, 2H), 2.78 – 2.56 (m, 3H), 2.46 (dd, *J* = 14.2, 8.2 Hz, 1H), 2.36 – 2.20 (m, 1H), 1.86 – 1.72 (m, 3H), 1.69 (s, 3H).

^{13}C NMR: (100 MHz, $CDCl_3$) δ = 204.8, 162.9, 150.3, 137.4, 134.6, 133.0, 132.3, 130.3, 128.4, 128.3, 122.9, 118.6, 85.0, 55.2, 46.4, 40.7, 35.0, 21.7, 17.6.

IR: *f* (cm^{-1}) = 2935, 1725, 1710, 1608, 1522, 1490, 1376, 1320, 1180, 1096, 1015, 717, 703.

HRMS (ESI-TOF): *m/z* [M+Na]$^+$ =450.1084 calculated for $C_{23}H_{22}ClNO_5Na$; found 450.1064.

X-Ray Structure: The ellipsoid contour was set at a 50% probability level.

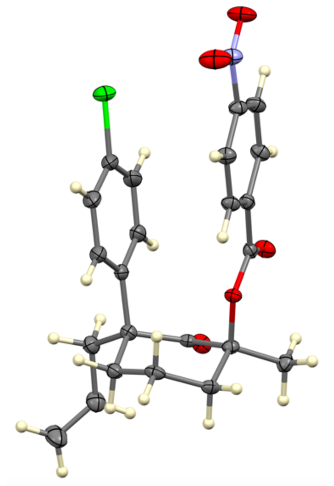

EXPERIMENTAL DATA FOR SCHEME 4

Compound (±)-7a

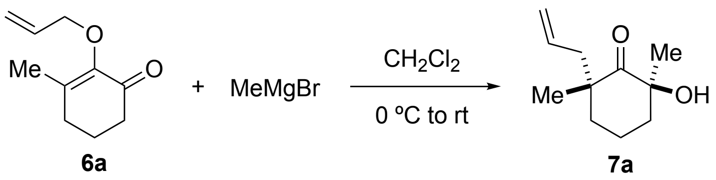

Procedure: Ketone **6a** (1.00 g, 6.02 mmol) and CH_2Cl_2 (30 mL) were added into a round bottom flask. After cooling the solution to 0 ºC, methylmagnesium bromide (2.61 mL, 7.82 mmol, 3 M in Et_2O) was added dropwise. The reaction mixture was allowed to warm to room temperature and stirred for 22 hours, at which the Claisen rearrangement of the carbonyl addition intermediate was complete as monitored by TLC. After cooling to 0 °C, the reaction was quenched with saturated NH_4Cl (50 mL), and the resulting mixture was extracted with CH_2Cl_2 (3 x 50 mL). The organic layers were combined, dried over Na_2SO_4, filtered, and then concentrated under vacuum. The crude material was purified with column chromatography using 100% hexanes to 95:5 hexanes : EtOAc to yield **7a** in 77% yield (845 mg, 4.64 mmol) as a colorless oil. ^{1}H NMR analysis of the crude reaction mixture indicated > 20:1 dr.

Compound (-)-7a

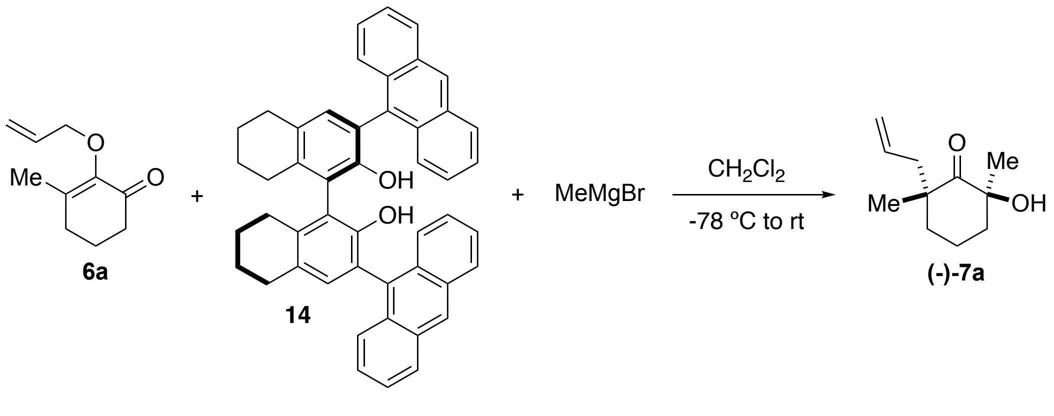

Procedure: Ligand **14** (5.40 g, 8.36 mmol) and ketone **6a** (1.00 g, 6.02 mmol) were dissolved in CH_2Cl_2 (30 mL) in a round bottom flask. After cooling the solution to -78 °C, methylmagnesium bromide (7.82 mL, 23.46 mmol, 3 M in Et_2O) was added dropwise. After stirring at -78 °C until the starting material was fully consumed as monitored by TLC, the reaction mixture was warmed to room temperature and stirred until the Claisen rearrangement of the carbonyl addition intermediate was complete as monitored by TLC for a total reaction time of 24 hours. After cooling to 0 °C, the reaction was quenched with a saturated NH_4Cl solution (20 mL) and diluted with DI water (20 mL). The aqueous layer was extracted with CH_2Cl_2 (3 x 30 mL), and the combined organic layers were washed with brine, dried over Na_2SO_4, and concentrated under vacuum. The crude material was purified by column chromatography using 100% hexanes to 50:50 hexanes : CH_2Cl_2 to afford compound **(-)-7a** in 63% yield (695 mg, 3.81 mmol) as a colorless oil. ^{1}H NMR analysis of the crude reaction mixture indicated > 20:1 dr. Percent recovery of pure ligand **14** after column chromatography was 93% (5.03 g, 7.78 mmol).

HPLC ((-)-7a)-BzNO₂: OD-H, hexane/isopropanol = 80/20, flow rate = 1.0 mL/min, λ = 254 nm, t_R = 6.4 min (minor), 11.6 min (major).

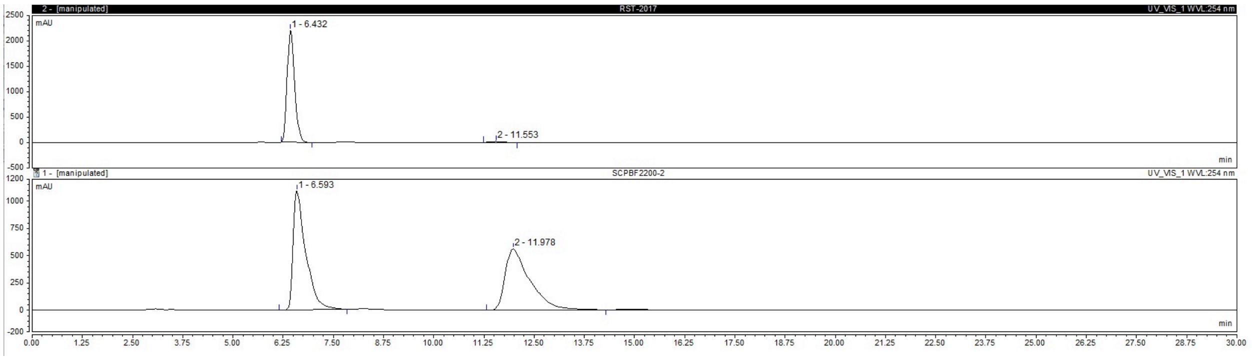

Retention Time (min)	Relative Area (%)	Area (mAU*min)	Height (mAU)
6.432	98.35	474.1409	2197.63
11.553	1.65	7.9416	20.24

Compound (+)-16

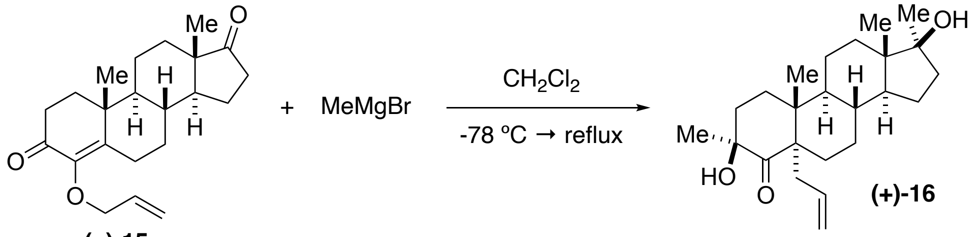

Procedure: Ketone **(+)-15a** (100 mg, 0.291 mmol) and CH_2Cl_2 (1.5 mL) were added into a pressure vessel. After cooling the solution to -78 ºC, methylmagnesium bromide (0.25 mL, 0.76 mmol, 3 M in Et_2O) was added dropwise. The reaction mixture was allowed to stir at -78 ºC for 4 hours until the starting material was fully consumed as monitored by TLC. The pressure vessel was warmed to room temperature and then heated to bring the reaction mixture to gentle reflux for 48 hours, at which the Claisen rearrangement of the carbonyl addition intermediate was complete as monitored by TLC. After cooling to 0 °C, the reaction was quenched with saturated NH_4Cl (5 mL), and the resulting mixture was extracted with CH_2Cl_2 (3 x 5 mL). The organic layers were combined, dried over Na_2SO_4, filtered, and then concentrated under vacuum. The crude material was purified with column chromatography using 100% hexanes to 80:20 hexanes : EtOAc to yield **(+)-16** in 69% yield (76 mg, 0.202 mmol) as a white crystalline solid. ^{1}H NMR analysis of the crude reaction mixture indicated > 20:1 dr.

Rf: 0.3 in 70:30 hexanes : EtOAc

^{1}H NMR: (500 MHz, DMSO-d_6) δ = 5.26 – 5.15 (m, 1H), 5.08 (d, *J* = 16.9 Hz, 1H), 5.02 – 4.97 (m, 1H), 4.90 (s, 1H), 4.03 (s, 1H), 2.67 (dd, *J* = 15.4, 9.0 Hz, 1H), 2.61 – 2.54 (m, 1H), 1.98 – 1.91 (m, 1H), 1.86 (td, *J* = 13.5, 6.3 Hz, 1H), 1.79 – 1.67 (m, 2H), 1.51 (dtd, *J* = 12.9, 9.8, 4.2 Hz, 3H), 1.47 – 1.40 (m, 3H), 1.35 (dd, *J* = 12.9, 9.8 Hz, 4H), 1.28 (s, 3H), 1.26 – 1.11 (m, 4H), 1.10 (s, 3H), 1.01 (qd, *J* = 14.3, 12.5, 4.7 Hz, 1H), 0.82 (s, 3H), 0.72 (s, 3H).

^{13}C NMR: (125 MHz, DMSO-d_6) δ = 217.1, 133.9, 117.9, 79.6, 73.5, 54.1, 49.8, 45.4, 44.9, 40.9, 38.4, 36.6, 34.8, 31.7, 31.6, 29.8, 27.6, 26.2, 23.7, 23.0, 22.8, 21.0, 17.4, 14.4.

IR: *f* (cm^{-1}) = 3430, 2910, 2828, 1730, 1670, 1435, 1150, 1020, 986, 940.

HRMS (ESI-TOF): *m/z* $[M+H]^+$ =375.2894 calculated for $C_{24}H_{39}O_3$; found 375.2895.

Specific Rotation: $[\alpha]_{25}^{D}$ = +86 (c = 1.00 in $CHCl_3$)

X-Ray Structure: The ellipsoid contour was set at a 50% probability level.

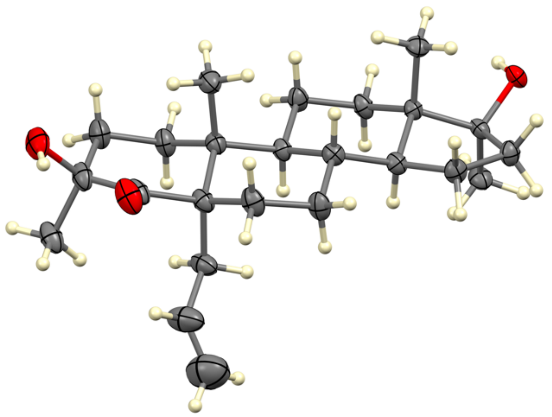

SYNTHESIS OF SUBSTRATES

Compound 6a

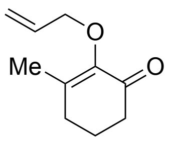

6a

This compound is known: Graßl, R.; Jandl, C.; and Bach, T. *J. Org. Chem.* **2020**, *85*, 11426-11439.

Compound 6b

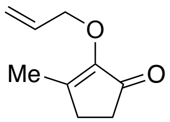

6b

This compound is known: Graßl, R.; Jandl, C.; and Bach, T. *J. Org. Chem.* **2020**, *85*, 11426-11439.

Compound 9a

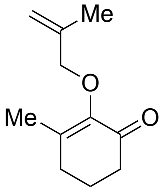

9a

This compound is known: Graßl, R.; Jandl, C.; and Bach, T. *J. Org. Chem.* **2020**, *85*, 11426-11439.

Compound 9b

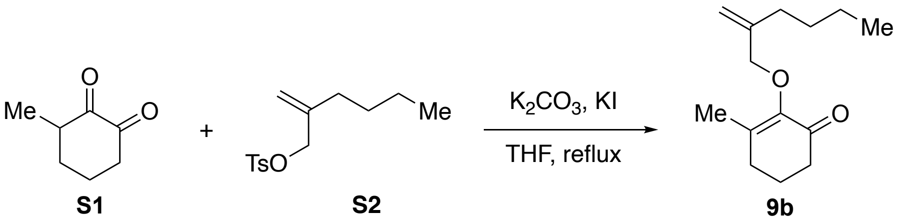

Procedure: Starting material **S1** (236 mg, 1.87 mmol) was dissolved in THF (3.7 mL). Allylic tosylate **S2** (500 mg, 1.87 mmol), K_2CO_3 (517 mg, 3.74 mmol), and KI (310 mg, 1.87 mmol) were added to the solution. The reaction mixture was then warmed to reflux until the starting material was completely consumed as monitored by TLC. After cooled to room temperature, the reaction mixture was concentrated under vacuum and diluted with DI water (30 mL). The aqueous layer was then extracted with CH_2Cl_2 (3 x 30 mL). The combined organic layers were washed with brine, dried over Na_2SO_4, and concentrated under vacuum. The crude material was purified with column chromatography using 100% hexanes to 96:4 hexanes : EtOAc to yield compound **9b** in 55% yield (230 mg, 1.03 mmol) as a colorless oil.

Rf: 0.5 in 80:20 (Hexanes : EtOAc)

^{1}H NMR: (400 MHz, $CDCl_3$) δ = 5.05 (s, 1H), 4.89 (s, 1H), 4.23 (s, 2H), 2.44 – 2.35 (m, 4H), 2.12 (t, J = 7.8 Hz, 2H), 1.96 – 1.88 (m, 2H), 1.91 (s, 3H), 1.48 – 1.42 (m, 2H), 1.36 – 1.29 (m, 2H), 0.90 (t, J = 7.4 Hz, 3H).

^{13}C NMR: (100 MHz, $CDCl_3$) δ = 194.6, 148.1, 146.0, 145.6, 111.6, 74.5, 38.8, 32.9, 31.5, 29.8, 22.5, 22.2, 17.7, 13.9.

IR: f (cm^{-1}) = 2929, 2868, 1677, 1632, 1456, 1379, 1304, 1194, 1153, 1031, 998, 929, 899.

HRMS (ESI-TOF): m/z $[M+H]^+$ = 223.1693 calculated for $C_{14}H_{23}O_2$; found 223.1687.

Compound 9c

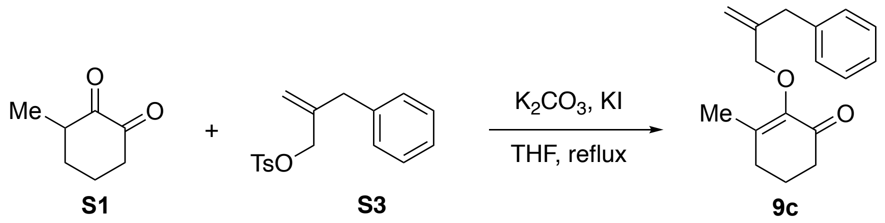

Procedure: Starting material **S1** (594 mg, 4.71 mmol) was dissolved in THF (9.4 mL). Allylic tosylate **S3** (1.42 g, 4.71 mmol), K_2CO_3 (1.30 g, 9.42 mmol), and KI (781 mg, 4.71) were added to the solution. The reaction mixture was then warmed to reflux until the starting material was completely consumed as monitored by TLC. After cooled to room temperature, the reaction mixture was concentrated under vacuum and diluted with DI water (30 mL). The aqueous layer was then extracted with CH_2Cl_2 (3 x 30 mL). The combined organic layers were washed with brine, dried over Na_2SO_4, and concentrated under vacuum. The crude material was purified with column chromatography using 100% hexanes to 96:4 hexanes : EtOAc to yield compound **9c** in 66% yield (799 mg, 3.12 mmol) as a colorless oil.

Rf: 0.4 in 80:20 (Hexanes : EtOAc)

^{1}H NMR: (500 MHz, $CDCl_3$) δ = 7.30 – 7.27 (m, 2H), 7.24 – 7.18 (m, 3H), 5.19 (s, 1H), 4.90 (d, *J* = 1.4 Hz, 1H), 4.22 (s, 2H), 3.49 (s, 2H), 2.44 – 2.41 (m, 2H), 2.40 – 2.35 (m, 2H), 1.94 – 1.89 (m, 5H).

^{13}C NMR: (125 MHz, $CDCl_3$) δ = 194.5, 148.0, 145.7, 145.1, 139.0, 129.1, 128.3, 126.1, 114.1, 73.9, 39.9, 38.7, 31.5, 22.1, 17.8.

IR: *f* (cm^{-1}) = 3027, 2925, 1674, 1631, 1453, 1432, 1379, 1304, 1193, 1151, 1032, 991, 928, 744, 701.

HRMS (ESI-TOF): *m/z* $[M+H]^+$ = 257.1536 calculated for $C_{17}H_{21}O_2$; found 257.1540.

Compound 9d

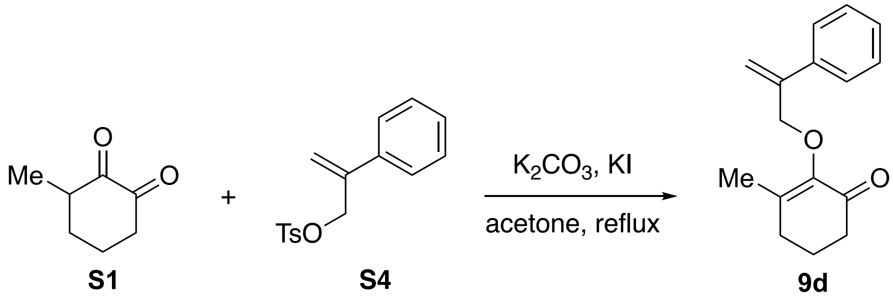

Procedure: Starting material **S1** (218 mg, 1.73 mmol) was dissolved in acetone (7.5 mL). Allylic tosylate **S4** (500 mg, 1.73 mmol), K_2CO_3 (479 mg, 3.46 mmol), and KI (29 mg, 0.173 mmol) were added to the solution. The reaction mixture was then warmed to reflux until the starting material was completely consumed as monitored by TLC. After cooled to room temperature, the reaction mixture was concentrated under vacuum and diluted with DI water (30 mL). The aqueous layer was then extracted with CH_2Cl_2 (3 x 30 mL). The combined organic layers were washed with brine, dried over Na_2SO_4, and concentrated under vacuum. The crude material was purified with column chromatography using 100% hexanes to 96:5 hexanes : EtOAc to yield compound **9d** in 65% yield (274 mg, 1.13 mmol) as a colorless oil.

Rf: 0.5 in 80:20 (Hexanes : EtOAc)

^{1}H NMR: (500 MHz, $CDCl_3$) δ = 7.48 – 7.43 (m, 2H), 7.27 (t, *J* = 7.4 Hz, 2H), 7.22 – 7.18 (m, 1H), 5.46 (s, 1H), 5.29 (s, 1H), 4.73 (s, 2H), 2.36 (dd, *J* = 7.4, 6.0 Hz, 2H), 2.27 – 2.21 (m, 2H), 1.86 – 1.80 (m, 2H), 1.60 (s, 3H).

^{13}C NMR: (125 MHz, $CDCl_3$) δ = 194.7, 147.4, 146.6, 144.3, 138.6, 128.3, 127.7, 126.2, 115.4, 73.1, 38.8, 31.5, 22.1, 17.7.

IR: *f* (cm^{-1}) = 3020, 2928, 1670, 1630, 1450, 1438, 1300, 1198, 1145, 1028, 991, 928, 744, 701.

HRMS (ESI-TOF): *m/z* $[M+H]^+$ =243.1380 calculated for $C_{16}H_{19}O_2$; found 243.1375.

Compound 9e

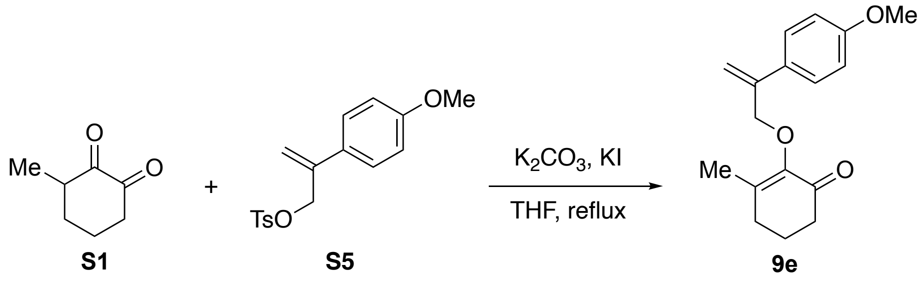

Procedure: Starting material **S1** (400 mg, 3.17 mmol) was dissolved in THF (6.3 mL). Allylic tosylate **S5** (1.01 g, 3.17 mmol), K_2CO_3 (877 mg, 6.34 mmol), and KI (527 mg, 3.17) were added to the solution. The reaction mixture was then warmed to reflux until the starting material was completely consumed as monitored by TLC. After cooled to room temperature, the reaction mixture was concentrated under vacuum and diluted with DI water (30 mL). The aqueous layer was then extracted with CH_2Cl_2 (3 x 30 mL). The combined organic layers were washed with brine, dried over Na_2SO_4, and concentrated under vacuum. The crude material was purified with column chromatography using 100% hexanes to 96:4 hexanes : EtOAc to yield compound **9e** in 43% yield (368 mg, 1.35 mmol) as a pale yellow oil.

Rf: 0.4 in 80:20 hexanes : EtOAc

^{1}H NMR: (400 MHz, $CDCl_3$) δ = 7.49 (d, J = 8.3 Hz, 2H), 6.90 – 6.83 (m, 2H), 5.44 (s, 1H), 5.24 (s, 1H), 4.75 (s, 2H), 3.80 (s, 3H), 2.43 (t, J = 6.7 Hz, 2H), 2.32 (t, J = 6.1 Hz, 2H), 1.92 – 1.88 (m, 2H), 1.70 (s, 3H).

^{13}C NMR: (100 MHz, $CDCl_3$) δ = 194.6, 159.3, 147.4, 146.5, 143.5, 131.1, 127.3, 113.7, 113.6, 73.3, 55.2, 38.8, 31.5, 22.1, 17.7.

IR: f (cm^{-1}) = 2936, 1732, 1671, 1629, 1605, 1513, 1457, 1303, 1248, 1182, 1151, 1029, 927, 835, 556.

HRMS (ESI-TOF): m/z $[M+H]^+$ = 273.1485 calculated for $C_{17}H_{21}O_3$; found 273.1473.

Compound 9f

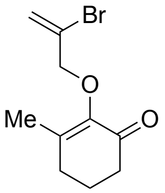

9f

This compound is known: Malone, J. A.; Philkhana, S. C.; Stepherson, J. R.; Badmus, F. O.; Fronczek; F. R.; Kartika, R. *Org. Lett.* **2022**, *23*, 4810-4815.

Compound 11a

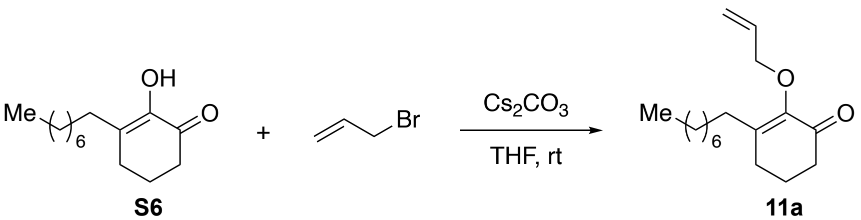

Procedure: Starting material **S6** (640 mg, 2.85 mmol) was dissolved in THF (7.1 mL). Allyl bromide (0.99 mL, 11.4 mmol) and Cs_2CO_3 (3.73 g, 11.4 mmol) were added to the solution. The reaction mixture was then stirred at room temperature until the starting material was completely consumed as monitored by TLC. The reaction mixture was diluted with DI water (20 mL). The aqueous layer was then extracted with EtOAc (3 x 20 mL). The combined organic layers were washed with brine, dried over Na_2SO_4, and concentrated under vacuum. The crude material was purified with column chromatography using 100% hexanes to 98:2 hexanes : EtOAc to yield compound **11a** in 78% yield (585 mg, 2.21 mmol) as a pale yellow oil.

Rf: 0.7 in 80:20 hexanes : EtOAc

^{1}H NMR: (400 MHz, $CDCl_3$) δ = 5.98 (ddt, *J* = 17.3, 10.3, 5.9 Hz, 1H), 5.28 (dq, *J* = 17.2, 1.7 Hz, 1H), 5.16 (dq, *J* = 10.4, 1.4 Hz, 1H), 4.32 (dt, *J* = 5.9, 1.4 Hz, 2H), 2.42 (t, *J* = 6.5 Hz, 2H),

2.37 (t, J = 6.0 Hz, 2H), 2.33 – 2.26 (m, 2H), 1.92 (p, J = 6.2 Hz, 2H), 1.47-1.40 (m, 2H), 1.34 – 1.22 (m, 10H), 0.92 – 0.82 (m, 3H).

^{13}C NMR: (100 MHz, $CDCl_3$) δ = 195.0, 150.1, 147.5, 134.4, 117.3, 72.9, 38.8, 31.8, 31.4, 29.7, 29.4, 29.4, 29.1, 27.5, 22.6, 22.4, 14.1.

IR: f (cm^{-1}) = 2925, 2855, 1677, 1624, 1457, 1365, 1327, 1305, 1192, 1145, 1095, 986, 926.

HRMS (ESI-TOF): *m/z* $[M+H]^+$ = 265.2162 calculated for $C_{17}H_{29}O_2$; found 265.2155.

Compound 11b

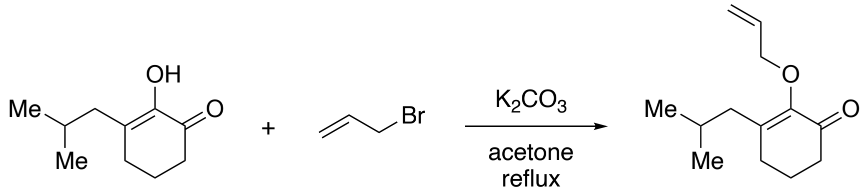

Procedure: Starting material **S7** (151 mg, 0.898 mmol) was dissolved in anhydrous acetone (2.3 mL). Allyl bromide (0.31 mL, 3.59 mmol) and K_2CO_3 (498 mg, 3.60 mmol) were added to the solution. The reaction mixture was then warmed to reflux until the starting material was completely consumed as monitored by TLC. After cooled to room temperature, the reaction mixture was concentrated under vacuum and diluted with DI water (20 mL). The aqueous layer was then extracted with CH_2Cl_2 (3 x 20 mL). The combined organic layers were washed with brine, dried over Na_2SO_4, and concentrated under vacuum. The crude material was purified with column chromatography using 100% hexanes to 98:2 hexanes : EtOAc to yield compound **11b** in 64% yield (120 mg, 0.576 mmol) as a colorless oil.

Rf: 0.5 in 80:20 hexanes : EtOAc

¹H NMR: (500 MHz, $CDCl_3$) δ = 6.03 – 5.94 (m, 1H), 5.31 – 5.27 (m, 1H), 5.18 – 5.15 (m, 1H), 4.32 (dd, *J* = 5.9, 1.5 Hz, 2H), 2.43 (t, *J* = 7.0 Hz, 2H), 2.35 (t, *J* = 6.0 Hz, 2H), 2.20 (d, *J* = 7.6 Hz, 2H), 1.92 (p, *J* = 6.4 Hz, 2H), 1.89 – 1.82 (m, 1H), 0.91 (d, *J* = 6.7 Hz, 6H).

¹³C NMR (125 MHz, $CDCl_3$) δ = 195.0, 148.9, 148.3, 134.4, 117.1, 72.7, 40.4, 38.8, 29.7, 26.8, 22.7, 22.4.

IR: *f* (cm^{-1}) = 2955, 2868, 1675, 1622, 1462, 1367, 1307, 1187, 1150, 1087, 987, 920, 558.

HRMS (APCI-TOF): *m/z* $[M+H]^+$ = 209.1536 calculated for $C_{13}H_{21}O_2$; found 209.1540.

Compound 11c

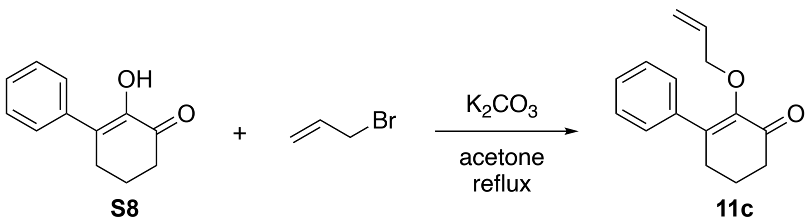

Procedure: Starting material **S8** (862 mg, 4.58 mmol) was dissolved in anhydrous acetone (11.4 mL). Allyl bromide (1.60 mL, 18.5 mmol) and K_2CO_3 (2.53 g, 18.3 mmol) were added to the solution. The reaction mixture was then warmed to reflux until the starting material was completely consumed as monitored by TLC. After cooled to room temperature, the reaction mixture was concentrated under vacuum and diluted with DI water (20 mL). The aqueous layer was then extracted with CH_2Cl_2 (3 x 20 mL). The combined organic layers were washed with brine, dried over Na_2SO_4, and concentrated under vacuum. The crude material was purified with column chromatography using 100% hexanes to 98:2 hexanes : EtOAc to yield compound **11c** in 53% yield (550 mg, 2.41 mmol) as a pale yellow oil.

Rf: 0.4 in 80:20 hexanes : EtOAc

^{1}H NMR: (500 MHz, $CDCl_3$) δ = 7.53 – 7.49 (m, 2H), 7.39 – 7.35 (m, 2H), 7.34 – 7.30 (m, 1H), 5.74 (ddt, *J* = 17.3, 10.3, 5.9 Hz, 1H), 5.11 (dq, *J* = 17.2, 1.6 Hz, 1H), 5.04 (dq, *J* = 10.4, 1.6 Hz, 1H), 4.21 (dt, *J* = 5.9, 1.4 Hz, 2H), 2.77 (t, *J* = 6.0 Hz, 2H), 2.57 (t, *J* = 6.0 Hz, 2H), 2.09 (p, *J* = 6.0 Hz, 2H).

^{13}C NMR: (125 MHz, $CDCl_3$) δ = 195.9, 147.5, 144.5, 137.5, 134.0, 128.5, 128.1, 128.0, 117.4, 72.9, 38.7, 31.0, 22.6.

IR: *f* (cm^{-1}) = 2942, 1673, 1601, 1443, 1365, 1328, 1304, 1192, 1149, 1091, 982, 926, 762, 697.

HRMS (APCI-TOF): *m/z* $[M+H]^+$ = 229.1223 calculated for $C_{15}H_{17}O_2$; found 229.1235.

Compound 11d

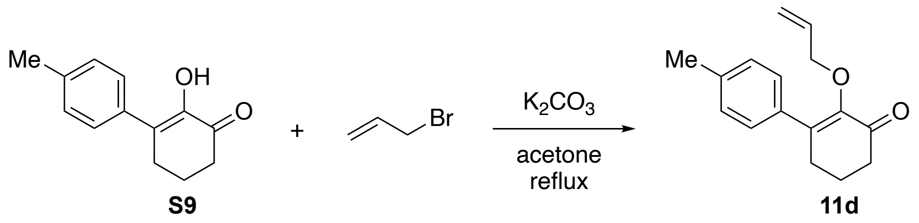

Procedure: Starting material **S9** (138 mg, 0.682 mmol) was dissolved in anhydrous acetone (1.7 mL). Allyl bromide (0.24 mL, 2.75 mmol) and K_2CO_3 (378 mg, 2.73 mmol) were added to the solution. The reaction mixture was then warmed to reflux until the starting material was completely consumed as monitored by TLC. After cooled to room temperature, the reaction mixture was concentrated under vacuum and diluted with DI water (20 mL). The aqueous layer was then extracted with CH_2Cl_2 (3 x 20 mL). The combined organic layers were washed with brine, dried over Na_2SO_4, and concentrated under vacuum. The crude material was purified with column chromatography using 100% hexanes to 96:4 hexanes: EtOAc to yield compound **11d** in 25% yield (42 mg, 0.17 mmol) as a pale yellow oil.

Rf: 0.5 in 80:20 hexanes : EtOAc

^{1}**H NMR:** (500 MHz, $CDCl_3$) δ = 7.44 (d, J = 8.2 Hz, 2H), 7.18 (d, J = 7.8 Hz, 2H), 5.82 – 5.72 (m, 1H), 5.13 (dt, J = 17.1, 1.7 Hz, 1H), 5.06 (dt, J = 10.4, 1.4 Hz, 1H), 4.21 (dd, J = 5.9, 1.5 Hz, 2H), 2.76 (t, J = 6.0 Hz, 2H), 2.55 (t, J = 6.6 Hz, 2H), 2.37 (s, 3H), 2.07 (p, J = 6.2 Hz, 2H).

^{13}**C NMR:** (125 MHz, $CDCl_3$) δ = 195.8, 147.3, 144.4, 138.6, 134.5, 134.1, 128.7, 128.1, 117.4, 72.8, 38.7, 30.9, 22.5, 21.3.

IR: f (cm^{-1}) = 2941, 1672, 1604, 1511, 1453, 1363, 1327, 1304, 1191, 1151, 1025, 982, 923, 814, 508.

HRMS (APCI-TOF): m/z $[M+H]^+$ = 243.1380 calculated for $C_{16}H_{19}O_2$; found 243.1381.

Compound 11e

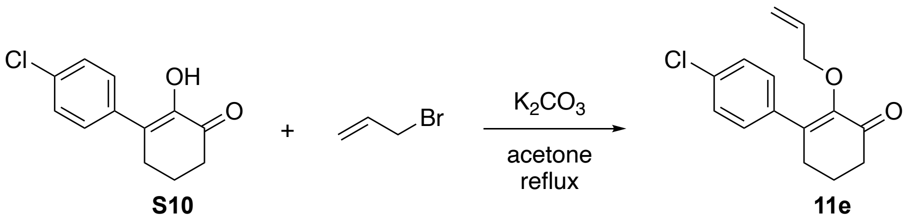

Procedure: Starting material **S10** (250 mg, 1.12 mmol) was dissolved in anhydrous acetone (2.8 mL). Allyl bromide (0.39 mL, 4.52 mmol) and K_2CO_3 (620 mg, 4.49 mmol) were added to the solution. The reaction mixture was then warmed to reflux until the starting material was completely consumed as monitored by TLC. After cooled to room temperature, the reaction mixture was concentrated under vacuum and diluted with DI water (20 mL). The aqueous layer was then extracted with CH_2Cl_2 (3 x 20 mL). The combined organic layers were washed with brine, dried over Na_2SO_4, and concentrated under vacuum. The crude material was purified with column chromatography using 100% hexanes to 98:2 hexanes : EtOAc to yield compound **11e** in 63% yield (186 mg, 0.708 mmol) as a pale yellow oil.

Rf: 0.4 in 80:20 hexanes : EtOAc

^{1}H NMR: (500 MHz, $CDCl_3$) δ = 7.47 (d, *J* = 7.9 Hz, 2H), 7.34 (d, *J* = 7.9 Hz, 2H), 5.74 (ddt, *J* = 16.4, 11.0, 5.9 Hz, 1H), 5.12 (d, *J* = 17.2 Hz, 1H), 5.07 (d, *J* = 10.2 Hz, 1H), 4.23 (d, *J* = 6.0 Hz, 2H), 2.74 (t, *J* = 6.0 Hz, 2H), 2.56 (t, *J* = 6.7 Hz, 2H), 2.09 (p, *J* = 6.3 Hz, 2H).

^{13}C NMR: (125 MHz, $CDCl_3$) δ = 195.7, 147.6, 142.8, 135.8, 134.3, 133.8, 129.6, 128.3, 117.7, 72.9, 38.7, 30.7, 22.5.

IR: *f* (cm^{-1}) = 2944, 1677, 1592, 1491, 1364, 1309, 1192, 1151, 1093, 1013, 981, 925, 824, 709.

HRMS (APCI-TOF): *m/z* $[M+H]^+$ = 263.0833 calculated for $C_{15}H_{16}ClO_2$; found 263.0839.

Compound 11f

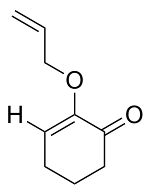

11f

This compound is known: Graßl, R.; Jandl, C.; and Bach, T. *J. Org. Chem.* **2020**, *85*, 11426-11439.

Compound (+)-15

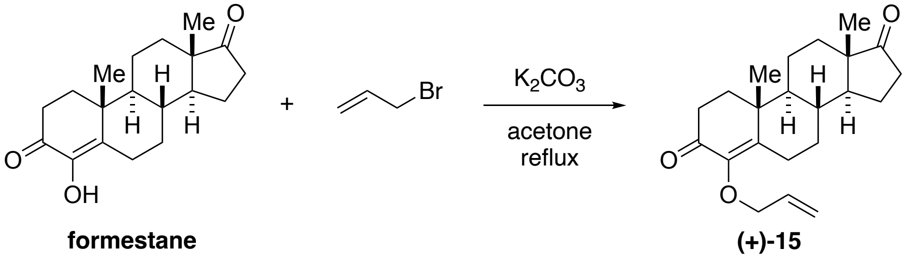

Procedure: Formestane (500 mg, 1.65 mmol) was dissolved in anhydrous acetone (20 mL). Allyl bromide (0.57 mL, 6.60 mmol) and K_2CO_3 (1.14 g, 8.27 mmol) were added to the solution. The reaction mixture was then warmed to reflux until the starting material was completely consumed

as monitored by TLC. After cooled to room temperature, the reaction mixture was concentrated under vacuum and diluted with DI water (30 mL). The aqueous layer was then extracted with CH_2Cl_2 (3 x 40 mL). The combined organic layers were washed with brine, dried over Na_2SO_4, and concentrated under vacuum. The crude material was purified with column chromatography using 77% hexanes to 80:20 hexanes : EtOAc to yield compound **11d** in 77% yield (435 mg, 1.27 mmol) as a white solid.

Rf: 0.5 in 50:50 hexanes : EtOAc

^{1}H NMR: (400 MHz, $CDCl_3$) δ = δ 6.03 – 5.90 (m, 1H), 5.30 – 5.15 (m, 2H), 4.30 (dddd, *J* = 13.5, 12.2, 11.0, 6.1 Hz, 2H), 3.21 – 3.09 (m, 1H), 2.52 – 2.42 (m, 2H), 2.10 (dt, *J* = 19.1, 9.0 Hz, 1H), 2.02 – 1.92 (m, 3H), 1.92 – 1.82 (m, 1H), 1.75 – 1.63 (m, 3H), 1.61 – 1.52 (m, 5H), 1.45 – 1.39 (m, 1H), 1.21 (s, 3H), 1.05 – 0.97 (m, 2H), 0.91 (s, 3H).

^{13}C NMR: (125 MHz, $CDCl_3$) δ = 220.5, 194.0, 154.5, 145.0, 134.1, 118.0, 77.2, 73.2, 54.3, 50.9, 47.5, 38.8, 35.8, 34.8, 34.8, 34.2, 31.3, 30.3, 29.7, 23.5, 21.7, 20.3, 17.5, 13.7.

IR: *f* (cm^{-1}) = 2910, 2828, 1725, 1705, 1660, 1320, 1235, 1140, 1020, 986, 940.

HRMS (ESI-TOF): *m/z* $[M+H]^+$ = 343.2268 calculated for $C_{22}H_{31}O_3$; found 343.2269.

Specific Rotation: $[\alpha]_{25}^{D}$= +86 (c = 1.00 in $CHCl_3$)

X-RAY CRYSTALLOGRAPHY DATA

Compound (±)-8e

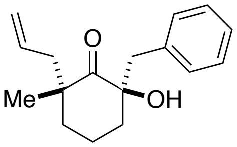

8e

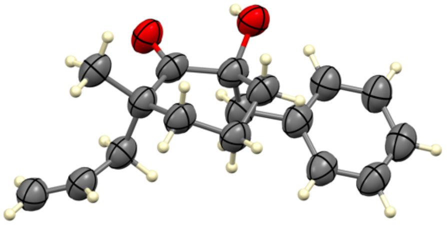

Sample Name: CCDC 2280303

Crystal Growth: Slow evaporation using a mixture of hexanes and dichloromethane.

Crystal data

$C_{17}H_{22}O_2$	$Z = 4$
$M_r = 258.34$	$F(000) = 560$
Triclinic, P-1	$D_x = 1.184$ Mg m^{-3}
$a = 11.2549$ (5) Å	Cu $K\alpha$ radiation, $\lambda = 1.54184$ Å
$b = 11.7198$ (6) Å	Cell parameters from 1815 reflections
$c = 12.3410$ (7) Å	$\theta = 4.0–59.0°$
$\alpha = 114.818$ (4)°	$\mu = 0.59$ mm^{-1}
$\beta = 92.311$ (4)°	$T = 90$ K
$\gamma = 98.850$ (4)°	Plate, colourless
$V = 1449.89$ (14) Å^3	0.15 × 0.14 × 0.02 mm

Data collection

Bruker Kappa APEX-II DUO diffractometer	4179 independent reflections
Radiation source: IμS microfocus	2448 reflections with $I > 2\sigma(I)$
QUAZAR multilayer optics monochromator	$R_{int} = 0.087$

ϕ and ω scans	θ_{max} = 59.2°, θ_{min} = 4.0°
Absorption correction: multi-scan *SADABS* (Krause *et al.*, 2015)	*h* = -12 – 12
T_{min} = 0.688, T_{max} = 0.988	*k* = -12 – 13
13051 measured reflections	*l* = -13 – 13

Refinement

Refinement on F^2	Hydrogen site location: mixed
Least-squares matrix: full	H atoms treated by a mixture of independent and constrained refinement
$R[F^2 > 2\sigma(F^2)]$ = 0.092	$w = 1/[\sigma^2(F_o^2) + (0.0943P)^2]$ where $P = (F_o^2 + 2F_c^2)/3$
$wR(F^2)$ = 0.260	$(\Delta/\sigma)_{max}$ < 0.001
S = 0.97	$\Delta\rho_{max}$ = 0.32 e Å^{-3}
4179 reflections	$\Delta\rho_{min}$ = -0.27 e Å^{-3}
352 parameters	Extinction correction: *SHELXL2018*/1 (Sheldrick 2018), $Fc^* = kFc[1+0.001xFc^2\lambda^3/\sin(2\theta)]^{-1/4}$
2 restraints	Extinction coefficient: 0.013 (2)

Fractional atomic coordinates and isotropic or equivalent isotropic displacement parameters (Å^2) for (Badmus5)

	x	*y*	*z*	U_{iso}*/U_{eq}
O1	0.5386 (3)	0.7909 (3)	0.4514 (3)	0.0731 (9)
O2	0.8282 (3)	0.7895 (3)	0.4876 (3)	0.0707 (9)
H2	0.791 (5)	0.714 (3)	0.464 (6)	0.106*
C1	0.6361 (4)	0.8582 (5)	0.5038 (5)	0.0676 (12)
C2	0.7497 (4)	0.8440 (5)	0.4372 (4)	0.0656 (12)
C3	0.8185 (4)	0.9765 (5)	0.4602 (4)	0.0714 (12)
H3A	0.896155	0.967748	0.425429	0.086*
H3B	0.770470	1.013771	0.418857	0.086*
C4	0.8438 (4)	1.0667 (5)	0.5929 (4)	0.0726 (13)

H4A	0.888749	1.150893	0.603389	0.087*
H4B	0.894746	1.031852	0.634290	0.087*
C5	0.7254 (4)	1.0835 (5)	0.6487 (5)	0.0717 (12)
H5A	0.677691	1.123733	0.610323	0.086*
H5B	0.744296	1.142658	0.735229	0.086*
C6	0.6476 (4)	0.9572 (5)	0.6357 (5)	0.0691 (12)
C7	0.7050 (5)	0.9047 (5)	0.7160 (4)	0.0773 (14)
H7A	0.788067	0.895959	0.698248	0.116*
H7B	0.657481	0.820794	0.700385	0.116*
H7C	0.705920	0.963810	0.800652	0.116*
C8	0.5196 (4)	0.9771 (5)	0.6701 (5)	0.0745 (13)
H8A	0.477709	0.998082	0.610570	0.089*
H8B	0.473285	0.895710	0.664980	0.089*
C9	0.5178 (5)	1.0797 (5)	0.7921 (5)	0.0795 (14)
H9	0.556531	1.164127	0.808507	0.095*
C10	0.4665 (5)	1.0621 (6)	0.8796 (5)	0.0902 (16)
H10A	0.426935	0.979014	0.866578	0.108*
H10B	0.469100	1.132619	0.955564	0.108*
C11	0.7131 (4)	0.7587 (5)	0.3031 (4)	0.0671 (12)
H11A	0.671142	0.673494	0.292747	0.081*
H11B	0.654845	0.796198	0.271955	0.081*
C12	0.8186 (4)	0.7416 (5)	0.2286 (4)	0.0707 (13)
C13	0.8857 (4)	0.6481 (5)	0.2146 (4)	0.0705 (12)
H13	0.863326	0.590743	0.249635	0.085*
C14	0.9845 (4)	0.6364 (5)	0.1508 (4)	0.0723 (13)
H14	1.028520	0.570423	0.141307	0.087*
C15	1.0199 (5)	0.7197 (5)	0.1007 (4)	0.0763 (14)
H15	1.088985	0.713095	0.058249	0.092*
C16	0.9528 (4)	0.8131 (5)	0.1134 (4)	0.0720 (13)
H16	0.975940	0.870657	0.078775	0.086*
C17	0.8533 (4)	0.8239 (5)	0.1752 (4)	0.0695 (12)
H17	0.807722	0.887869	0.181822	0.083*
O3	0.5520 (3)	0.5544 (3)	0.6413 (3)	0.0734 (9)

O4	0.6879 (3)	0.5591 (3)	0.4685 (3)	0.0699 (9)
H4	0.6131 (19)	0.537 (6)	0.455 (6)	0.105*
C18	0.6561 (4)	0.5430 (5)	0.6539 (4)	0.0655 (12)
C19	0.7273 (4)	0.5031 (5)	0.5434 (4)	0.0657 (12)
C20	0.8624 (4)	0.5528 (5)	0.5759 (4)	0.0690 (12)
H20A	0.879661	0.645105	0.595044	0.083*
H20B	0.904554	0.507619	0.505792	0.083*
C21	0.9118 (4)	0.5343 (5)	0.6831 (4)	0.0674 (12)
H21A	1.000737	0.563862	0.699159	0.081*
H21B	0.894057	0.442464	0.665561	0.081*
C22	0.8521 (4)	0.6115 (5)	0.7929 (4)	0.0685 (12)
H22A	0.869745	0.702739	0.808359	0.082*
H22B	0.888484	0.604775	0.863884	0.082*
C23	0.7133 (4)	0.5672 (5)	0.7790 (4)	0.0661 (12)
C24	0.6564 (4)	0.6689 (5)	0.8741 (5)	0.0749 (13)
H24A	0.568974	0.638277	0.865653	0.112*
H24B	0.671563	0.747946	0.863454	0.112*
H24C	0.692275	0.685688	0.954283	0.112*
C25	0.6805 (4)	0.4386 (5)	0.7902 (4)	0.0696 (12)
H25A	0.593394	0.402759	0.762565	0.084*
H25B	0.727165	0.376550	0.736985	0.084*
C26	0.7065 (4)	0.4543 (5)	0.9159 (4)	0.0703 (13)
H26	0.787422	0.488635	0.953499	0.084*
C27	0.6255 (5)	0.4242 (5)	0.9784 (5)	0.0740 (13)
H27A	0.543743	0.389611	0.943745	0.089*
H27B	0.648968	0.436964	1.058093	0.089*
C28	0.6923 (4)	0.3563 (5)	0.4760 (4)	0.0688 (12)
H28A	0.713503	0.319254	0.531409	0.083*
H28B	0.603423	0.332721	0.454617	0.083*
C29	0.7514 (4)	0.2960 (5)	0.3633 (5)	0.0700 (12)
C30	0.7069 (5)	0.2955 (5)	0.2559 (4)	0.0744 (13)
H30	0.640988	0.336977	0.254621	0.089*
C31	0.7575 (5)	0.2355 (5)	0.1519 (5)	0.0778 (14)

H31	0.727930	0.238077	0.079937	0.093*
C32	0.8513 (5)	0.1714 (5)	0.1514 (5)	0.0815 (15)
H32	0.884439	0.127577	0.078969	0.098*
C33	0.8958 (5)	0.1719 (5)	0.2569 (5)	0.0766 (14)
H33	0.961284	0.129719	0.257612	0.092*
C34	0.8462 (4)	0.2331 (5)	0.3623 (5)	0.0709 (13)
H34	0.877339	0.231925	0.434473	0.085*

Atomic displacement parameters (Å^2) for (Badmus)

	U^{11}	U^{22}	U^{33}	U^{12}	U^{13}	U^{23}
O1	0.053 (2)	0.082 (2)	0.082 (2)	0.0116 (17)	0.0128 (16)	0.0336 (17)
O2	0.0542 (19)	0.081 (2)	0.081 (2)	0.0229 (17)	0.0137 (15)	0.0342 (18)
C1	0.053 (3)	0.070 (3)	0.089 (3)	0.020 (2)	0.012 (2)	0.040 (3)
C2	0.048 (2)	0.071 (3)	0.082 (3)	0.013 (2)	0.010 (2)	0.035 (2)
C3	0.059 (3)	0.072 (3)	0.083 (3)	0.016 (2)	0.012 (2)	0.031 (3)
C4	0.051 (3)	0.086 (3)	0.086 (3)	0.018 (2)	0.016 (2)	0.039 (3)
C5	0.058 (3)	0.072 (3)	0.085 (3)	0.017 (2)	0.011 (2)	0.032 (3)
C6	0.048 (3)	0.073 (3)	0.091 (3)	0.018 (2)	0.013 (2)	0.037 (3)
C7	0.067 (3)	0.084 (3)	0.080 (3)	0.024 (3)	0.016 (2)	0.032 (3)
C8	0.057 (3)	0.083 (3)	0.086 (3)	0.019 (3)	0.016 (2)	0.036 (3)
C9	0.061 (3)	0.077 (3)	0.099 (4)	0.021 (3)	0.016 (3)	0.034 (3)
C10	0.087 (4)	0.089 (4)	0.091 (4)	0.027 (3)	0.025 (3)	0.030 (3)
C11	0.050 (3)	0.072 (3)	0.082 (3)	0.016 (2)	0.010 (2)	0.035 (2)
C12	0.051 (3)	0.079 (3)	0.077 (3)	0.020 (2)	0.008 (2)	0.026 (3)
C13	0.055 (3)	0.079 (3)	0.075 (3)	0.017 (2)	0.009 (2)	0.029 (2)
C14	0.058 (3)	0.079 (3)	0.075 (3)	0.022 (3)	0.006 (2)	0.026 (3)
C15	0.058 (3)	0.096 (4)	0.070 (3)	0.020 (3)	0.013 (2)	0.029 (3)
C16	0.057 (3)	0.080 (3)	0.074 (3)	0.010 (3)	0.008 (2)	0.030 (2)
C17	0.060 (3)	0.072 (3)	0.075 (3)	0.019 (2)	0.011 (2)	0.029 (2)
O3	0.051 (2)	0.088 (2)	0.081 (2)	0.0223 (17)	0.0118 (15)	0.0322 (18)

O4	0.0512 (18)	0.079 (2)	0.084 (2)	0.0164 (17)	0.0072 (16)	0.0385 (18)
C18	0.047 (3)	0.068 (3)	0.081 (3)	0.018 (2)	0.009 (2)	0.029 (2)
C19	0.045 (2)	0.070 (3)	0.084 (3)	0.015 (2)	0.010 (2)	0.035 (2)
C20	0.050 (3)	0.072 (3)	0.083 (3)	0.017 (2)	0.009 (2)	0.030 (2)
C21	0.047 (3)	0.075 (3)	0.082 (3)	0.016 (2)	0.009 (2)	0.036 (2)
C22	0.049 (3)	0.076 (3)	0.083 (3)	0.017 (2)	0.010 (2)	0.034 (3)
C23	0.051 (3)	0.075 (3)	0.074 (3)	0.020 (2)	0.014 (2)	0.031 (2)
C24	0.056 (3)	0.080 (3)	0.085 (3)	0.022 (2)	0.013 (2)	0.029 (3)
C25	0.050 (3)	0.076 (3)	0.080 (3)	0.016 (2)	0.012 (2)	0.030 (2)
C26	0.057 (3)	0.078 (3)	0.077 (3)	0.020 (2)	0.011 (2)	0.031 (3)
C27	0.066 (3)	0.076 (3)	0.084 (3)	0.017 (3)	0.017 (3)	0.036 (3)
C28	0.046 (2)	0.079 (3)	0.081 (3)	0.013 (2)	0.011 (2)	0.033 (3)
C29	0.050 (3)	0.071 (3)	0.086 (3)	0.007 (2)	0.014 (2)	0.032 (3)
C30	0.064 (3)	0.081 (3)	0.075 (3)	0.016 (3)	0.013 (2)	0.030 (3)
C31	0.062 (3)	0.089 (3)	0.078 (3)	0.015 (3)	0.016 (2)	0.031 (3)
C32	0.070 (3)	0.079 (3)	0.088 (4)	0.012 (3)	0.025 (3)	0.028 (3)
C33	0.057 (3)	0.072 (3)	0.098 (4)	0.017 (2)	0.017 (3)	0.031 (3)
C34	0.056 (3)	0.068 (3)	0.088 (3)	0.007 (2)	0.016 (2)	0.033 (3)

Geometric parameters (Å, º) for (Badmus)

O1—C1	1.222 (6)	O3—C18	1.210 (5)
O2—C2	1.428 (6)	O4—C19	1.432 (6)
O2—H2	0.83 (2)	O4—H4	0.83 (2)
C1—C6	1.538 (7)	C18—C23	1.541 (7)
C1—C2	1.541 (7)	C18—C19	1.546 (7)
C2—C3	1.527 (7)	C19—C20	1.515 (6)
C2—C11	1.529 (7)	C19—C28	1.540 (7)
C3—C4	1.516 (7)	C20—C21	1.525 (7)
C3—H3A	0.9900	C20—H20A	0.9900
C3—H3B	0.9900	C20—H20B	0.9900
C4—C5	1.526 (6)	C21—C22	1.529 (6)

C4—H4A	0.9900	C21—H21A	0.9900
C4—H4B	0.9900	C21—H21B	0.9900
C5—C6	1.541 (7)	C22—C23	1.546 (6)
C5—H5A	0.9900	C22—H22A	0.9900
C5—H5B	0.9900	C22—H22B	0.9900
C6—C7	1.537 (7)	C23—C24	1.526 (7)
C6—C8	1.544 (6)	C23—C25	1.562 (7)
C7—H7A	0.9800	C24—H24A	0.9800
C7—H7B	0.9800	C24—H24B	0.9800
C7—H7C	0.9800	C24—H24C	0.9800
C8—C9	1.485 (8)	C25—C26	1.493 (7)
C8—H8A	0.9900	C25—H25A	0.9900
C8—H8B	0.9900	C25—H25B	0.9900
C9—C10	1.319 (8)	C26—C27	1.318 (7)
C9—H9	0.9500	C26—H26	0.9500
C10—H10A	0.9500	C27—H27A	0.9500
C10—H10B	0.9500	C27—H27B	0.9500
C11—C12	1.517 (7)	C28—C29	1.506 (7)
C11—H11A	0.9900	C28—H28A	0.9900
C11—H11B	0.9900	C28—H28B	0.9900
C12—C13	1.381 (7)	C29—C34	1.385 (7)
C12—C17	1.399 (7)	C29—C30	1.395 (7)
C13—C14	1.380 (7)	C30—C31	1.375 (7)
C13—H13	0.9500	C30—H30	0.9500
C14—C15	1.379 (8)	C31—C32	1.386 (7)
C14—H14	0.9500	C31—H31	0.9500
C15—C16	1.383 (7)	C32—C33	1.373 (8)
C15—H15	0.9500	C32—H32	0.9500
C16—C17	1.372 (7)	C33—C34	1.385 (7)
C16—H16	0.9500	C33—H33	0.9500
C17—H17	0.9500	C34—H34	0.9500
C2—O2—H2	103 (5)	C19—O4—H4	106 (5)

O1—C1—C6	121.1 (4)	O3—C18—C23	119.2 (4)
O1—C1—C2	119.7 (4)	O3—C18—C19	118.9 (4)
C6—C1—C2	119.2 (4)	C23—C18—C19	121.9 (4)
O2—C2—C3	106.6 (4)	O4—C19—C20	106.0 (4)
O2—C2—C11	110.7 (4)	O4—C19—C28	109.4 (4)
C3—C2—C11	111.9 (4)	C20—C19—C28	114.0 (4)
O2—C2—C1	108.4 (4)	O4—C19—C18	108.0 (4)
C3—C2—C1	109.4 (4)	C20—C19—C18	113.1 (4)
C11—C2—C1	109.8 (4)	C28—C19—C18	106.2 (4)
C4—C3—C2	112.5 (4)	C19—C20—C21	112.2 (4)
C4—C3—H3A	109.1	C19—C20—H20A	109.2
C2—C3—H3A	109.1	C21—C20—H20A	109.2
C4—C3—H3B	109.1	C19—C20—H20B	109.2
C2—C3—H3B	109.1	C21—C20—H20B	109.2
H3A—C3—H3B	107.8	H20A—C20—H20B	107.9
C3—C4—C5	110.3 (4)	C20—C21—C22	108.7 (4)
C3—C4—H4A	109.6	C20—C21—H21A	109.9
C5—C4—H4A	109.6	C22—C21—H21A	109.9
C3—C4—H4B	109.6	C20—C21—H21B	109.9
C5—C4—H4B	109.6	C22—C21—H21B	109.9
H4A—C4—H4B	108.1	H21A—C21—H21B	108.3
C4—C5—C6	113.7 (4)	C21—C22—C23	113.8 (4)
C4—C5—H5A	108.8	C21—C22—H22A	108.8
C6—C5—H5A	108.8	C23—C22—H22A	108.8
C4—C5—H5B	108.8	C21—C22—H22B	108.8
C6—C5—H5B	108.8	C23—C22—H22B	108.8
H5A—C5—H5B	107.7	H22A—C22—H22B	107.7
C7—C6—C1	109.0 (4)	C24—C23—C18	108.5 (4)
C7—C6—C5	111.4 (4)	C24—C23—C22	110.5 (4)
C1—C6—C5	108.7 (4)	C18—C23—C22	110.7 (4)

C7—C6—C8	108.5 (4)	C24—C23—C25	109.2 (4)
C1—C6—C8	108.5 (4)	C18—C23—C25	106.8 (4)
C5—C6—C8	110.6 (4)	C22—C23—C25	111.1 (4)
C6—C7—H7A	109.5	C23—C24—H24A	109.5
C6—C7—H7B	109.5	C23—C24—H24B	109.5
H7A—C7—H7B	109.5	H24A—C24—H24B	109.5
C6—C7—H7C	109.5	C23—C24—H24C	109.5
H7A—C7—H7C	109.5	H24A—C24—H24C	109.5
H7B—C7—H7C	109.5	H24B—C24—H24C	109.5
C9—C8—C6	114.2 (4)	C26—C25—C23	112.7 (4)
C9—C8—H8A	108.7	C26—C25—H25A	109.1
C6—C8—H8A	108.7	C23—C25—H25A	109.1
C9—C8—H8B	108.7	C26—C25—H25B	109.1
C6—C8—H8B	108.7	C23—C25—H25B	109.1
H8A—C8—H8B	107.6	H25A—C25—H25B	107.8
C10—C9—C8	124.8 (6)	C27—C26—C25	124.8 (5)
C10—C9—H9	117.6	C27—C26—H26	117.6
C8—C9—H9	117.6	C25—C26—H26	117.6
C9—C10—H10A	120.0	C26—C27—H27A	120.0
C9—C10—H10B	120.0	C26—C27—H27B	120.0
H10A—C10—H10B	120.0	H27A—C27—H27B	120.0
C12—C11—C2	114.0 (4)	C29—C28—C19	115.3 (4)
C12—C11—H11A	108.8	C29—C28—H28A	108.4
C2—C11—H11A	108.8	C19—C28—H28A	108.4
C12—C11—H11B	108.8	C29—C28—H28B	108.4
C2—C11—H11B	108.8	C19—C28—H28B	108.4
H11A—C11—H11B	107.7	H28A—C28—H28B	107.5
C13—C12—C17	117.9 (4)	C34—C29—C30	118.6 (5)

C13—C12—C11	121.6 (5)	C34—C29—C28	120.6 (5)
C17—C12—C11	120.5 (4)	C30—C29—C28	120.7 (4)
C14—C13—C12	121.3 (5)	C31—C30—C29	120.6 (5)
C14—C13—H13	119.4	C31—C30—H30	119.7
C12—C13—H13	119.4	C29—C30—H30	119.7
C15—C14—C13	120.4 (5)	C30—C31—C32	120.5 (5)
C15—C14—H14	119.8	C30—C31—H31	119.8
C13—C14—H14	119.8	C32—C31—H31	119.8
C14—C15—C16	118.7 (5)	C33—C32—C31	119.2 (5)
C14—C15—H15	120.6	C33—C32—H32	120.4
C16—C15—H15	120.6	C31—C32—H32	120.4
C17—C16—C15	121.0 (5)	C32—C33—C34	120.7 (5)
C17—C16—H16	119.5	C32—C33—H33	119.6
C15—C16—H16	119.5	C34—C33—H33	119.6
C16—C17—C12	120.5 (5)	C33—C34—C29	120.4 (5)
C16—C17—H17	119.7	C33—C34—H34	119.8
C12—C17—H17	119.7	C29—C34—H34	119.8
O1—C1—C2—O2	-110.9 (5)	O3—C18—C19—O4	34.7 (6)
C6—C1—C2—O2	69.1 (5)	C23—C18—C19—O4	-146.0 (4)
O1—C1—C2—C3	133.2 (5)	O3—C18—C19—C20	151.7 (4)
C6—C1—C2—C3	-46.8 (6)	C23—C18—C19—C20	-28.9 (6)
O1—C1—C2—C11	10.1 (6)	O3—C18—C19—C28	-82.6 (5)
C6—C1—C2—C11	-169.9 (4)	C23—C18—C19—C28	96.8 (5)
O2—C2—C3—C4	-64.9 (5)	O4—C19—C20—C21	163.3 (4)
C11—C2—C3—C4	174.0 (4)	C28—C19—C20—C21	-76.3 (5)
C1—C2—C3—C4	52.1 (5)	C18—C19—	45.1 (6)

		C20—C21	
C2—C3—C4—C5	-59.6 (5)	C19—C20—C21—C22	-62.4 (5)
C3—C4—C5—C6	58.5 (6)	C20—C21—C22—C23	62.7 (5)
O1—C1—C6—C7	103.4 (5)	O3—C18—C23—C24	-31.5 (6)
C2—C1—C6—C7	-76.6 (5)	C19—C18—C23—C24	149.1 (4)
O1—C1—C6—C5	-135.0 (4)	O3—C18—C23—C22	-152.9 (4)
C2—C1—C6—C5	45.0 (5)	C19—C18—C23—C22	27.7 (6)
O1—C1—C6—C8	-14.6 (6)	O3—C18—C23—C25	86.0 (5)
C2—C1—C6—C8	165.4 (4)	C19—C18—C23—C25	-93.3 (5)
C4—C5—C6—C7	70.8 (5)	C21—C22—C23—C24	-164.3 (4)
C4—C5—C6—C1	-49.3 (5)	C21—C22—C23—C18	-44.1 (5)
C4—C5—C6—C8	-168.3 (4)	C21—C22—C23—C25	74.4 (5)
C7—C6—C8—C9	67.1 (6)	C24—C23—C25—C26	-50.4 (5)
C1—C6—C8—C9	-174.6 (4)	C18—C23—C25—C26	-167.5 (4)
C5—C6—C8—C9	-55.5 (6)	C22—C23—C25—C26	71.7 (5)
C6—C8—C9—C10	-119.2 (6)	C23—C25—C26—C27	121.6 (5)
O2—C2—C11—C12	-62.0 (5)	O4—C19—C28—C29	63.3 (5)
C3—C2—C11—C12	56.8 (5)	C20—C19—C28—C29	-55.2 (6)
C1—C2—C11—C12	178.4 (4)	C18—C19—C28—C29	179.6 (4)
C2—C11—C12—	85.9 (6)	C19—C28—	104.0 (5)

C13		C29—C34	
C2—C11—C12—C17	-91.9 (6)	C19—C28—C29—C30	-80.0 (6)
C17—C12—C13—C14	0.4 (7)	C34—C29—C30—C31	-1.1 (8)
C11—C12—C13—C14	-177.4 (5)	C28—C29—C30—C31	-177.1 (5)
C12—C13—C14—C15	1.0 (8)	C29—C30—C31—C32	1.9 (8)
C13—C14—C15—C16	-1.4 (7)	C30—C31—C32—C33	-2.1 (8)
C14—C15—C16—C17	0.4 (7)	C31—C32—C33—C34	1.4 (8)
C15—C16—C17—C12	1.0 (8)	C32—C33—C34—C29	-0.6 (8)
C13—C12—C17—C16	-1.4 (7)	C30—C29—C34—C33	0.4 (7)
C11—C12—C17—C16	176.4 (4)	C28—C29—C34—C33	176.4 (5)

Hydrogen-bond geometry (Å, º) for (Badmus)

D—H···*A*	*D*—H	H···*A*	*D*···*A*	*D*—H···*A*
O2—H2···O4	0.83 (2)	2.03 (3)	2.823 (5)	159 (7)
O4—H4···O3^{i}	0.83 (2)	2.04 (3)	2.825 (4)	158 (6)

Symmetry code: (i) -*x*+1, -*y*+1, -*z*+1.

Compound (±)-8o

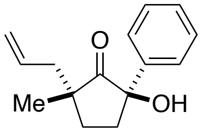

8o

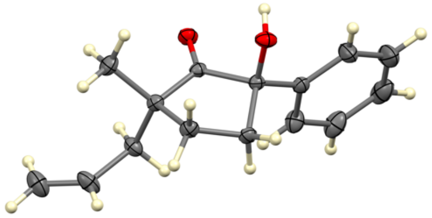

Sample Name: CCDC 2280307

Crystal Growth: Slow evaporation using a mixture of hexanes and dichloromethane.

Crystal data

$C_{15}H_{18}O_2$	$F(000) = 992$
$M_r = 230.29$	$D_x = 1.194$ Mg m^{-3}
Monoclinic, $P2_1/n$	Mo $K\alpha$ radiation, $\lambda = 0.71073$ Å
$a = 13.4405$ (12) Å	Cell parameters from 9930 reflections
$b = 8.9121$ (8) Å	$\theta = 2.7–36.3°$
$c = 21.4181$ (18) Å	$\mu = 0.08$ mm^{-1}
$\beta = 92.867$ (2)°	$T = 150$ K
$V = 2562.3$ (4) Å^3	Fragment, colourless
$Z = 8$	0.39 × 0.28 × 0.25 mm

Data collection

Bruker Kappa APEX-II DUO diffractometer	12476 independent reflections
Radiation source: fine-focus sealed tube	8348 reflections with $I > 2\sigma(I)$
TRIUMPH curved graphite monochromator	$R_{int} = 0.049$
ϕ and ω scans	$\theta_{max} = 36.5°$, $\theta_{min} = 1.8°$
Absorption correction: multi-scan *SADABS* (Krause *et al.*, 2015)	$h = -22 – 22$
$T_{min} = 0.958$, $T_{max} = 0.981$	$k = -14 – 14$

84060 measured reflections	l = -35 – 35

Refinement

Refinement on F^2	0 restraints
Least-squares matrix: full	Hydrogen site location: mixed
$R[F^2 > 2\sigma(F^2)] = 0.051$	H atoms treated by a mixture of independent and constrained refinement
$wR(F^2) = 0.145$	$w = 1/[\sigma^2(F_o^2) + (0.0687P)^2 + 0.3626P]$ where $P = (F_o^2 + 2F_c^2)/3$
S = 1.03	$(\Delta/\sigma)_{max} < 0.001$
12476 reflections	$\Delta\rho_{max}$ = 0.45 e Å^{-3}
315 parameters	$\Delta\rho_{min}$ = -0.21 e Å^{-3}

Fractional atomic coordinates and isotropic or equivalent isotropic displacement parameters (Å^2) for (BF3132)

	x	y	z	$U_{iso}*/U_{eq}$
O1	0.37409 (5)	0.37528 (7)	0.28290 (3)	0.02198 (13)
O2	0.51558 (5)	0.63651 (7)	0.26777 (3)	0.02196 (13)
H2O	0.5415 (10)	0.5499 (16)	0.2595 (6)	0.033*
C1	0.37279 (6)	0.50815 (9)	0.29560 (4)	0.01699 (14)
C2	0.41053 (6)	0.63439 (9)	0.25329 (4)	0.01770 (14)
C3	0.36827 (6)	0.77545 (9)	0.28272 (4)	0.02034 (15)
H3A	0.299197	0.794609	0.266441	0.024*
H3B	0.409783	0.864242	0.274306	0.024*
C4	0.37114 (7)	0.73953 (10)	0.35294 (4)	0.02145 (16)
H4A	0.324516	0.804910	0.374766	0.026*
H4B	0.439119	0.753803	0.371985	0.026*
C5	0.33925 (6)	0.57373 (9)	0.35685 (4)	0.01826 (14)
C6	0.22401 (6)	0.55447 (11)	0.35440 (4)	0.02300 (17)
H6A	0.195551	0.601738	0.315671	0.028*
H6B	0.207956	0.446105	0.352375	0.028*
C7	0.17572 (8)	0.62181 (13)	0.40933 (5)	0.0313 (2)

H7	0.172996	0.728145	0.411845	0.038*
C8	0.13681 (10)	0.54446 (17)	0.45434 (6)	0.0447 (3)
H8A	0.138162	0.437906	0.453406	0.054*
H8B	0.107355	0.595111	0.487800	0.054*
C9	0.38743 (8)	0.49018 (11)	0.41270 (4)	0.02618 (18)
H9A	0.366508	0.384842	0.411286	0.039*
H9B	0.366641	0.536160	0.451521	0.039*
H9C	0.460083	0.495865	0.411187	0.039*
C10	0.38392 (6)	0.61535 (9)	0.18425 (4)	0.01914 (15)
C11	0.45672 (8)	0.61943 (13)	0.14029 (5)	0.0300 (2)
H11	0.524733	0.631660	0.153585	0.036*
C12	0.43006 (9)	0.60560 (15)	0.07676 (5)	0.0373 (2)
H12	0.480223	0.608122	0.047057	0.045*
C13	0.33128 (9)	0.58825 (12)	0.05654 (5)	0.0319 (2)
H13	0.313600	0.578923	0.013212	0.038*
C14	0.25834 (8)	0.58460 (11)	0.10012 (5)	0.02658 (18)
H14	0.190369	0.573286	0.086580	0.032*
C15	0.28439 (7)	0.59744 (10)	0.16361 (4)	0.02239 (16)
H15	0.234041	0.594000	0.193166	0.027*
O3	0.62121 (5)	0.38177 (7)	0.22844 (3)	0.02134 (13)
O4	0.47743 (5)	0.12285 (7)	0.24409 (3)	0.02157 (13)
H4O	0.4549 (10)	0.2063 (16)	0.2534 (6)	0.032*
C16	0.62065 (6)	0.24924 (9)	0.21507 (4)	0.01583 (13)
C17	0.58285 (6)	0.12283 (9)	0.25736 (4)	0.01651 (14)
C18	0.62271 (6)	-0.01837 (9)	0.22681 (4)	0.01906 (15)
H18A	0.580637	-0.106444	0.235400	0.023*
H18B	0.692010	-0.039362	0.242161	0.023*
C19	0.61798 (7)	0.01912 (9)	0.15666 (4)	0.02010 (15)
H19A	0.549288	0.007374	0.138399	0.024*
H19B	0.662985	-0.047018	0.133910	0.024*
C20	0.65196 (6)	0.18399 (9)	0.15335 (4)	0.01716 (14)
C21	0.76724 (6)	0.19813 (11)	0.15508 (4)	0.02149 (16)
H21A	0.785673	0.304514	0.162254	0.026*

H21B	0.795783	0.139171	0.190833	0.026*
C22	0.81236 (8)	0.14523 (12)	0.09658 (5)	0.0298 (2)
H22	0.804715	0.042574	0.085385	0.036*
C23	0.86202 (8)	0.23277 (15)	0.05974 (5)	0.0355 (2)
H23A	0.870849	0.335912	0.069755	0.043*
H23B	0.889074	0.192866	0.023116	0.043*
C24	0.60409 (7)	0.27117 (11)	0.09837 (4)	0.02456 (17)
H24A	0.531549	0.271037	0.101237	0.037*
H24B	0.621424	0.223681	0.059095	0.037*
H24C	0.628557	0.374768	0.099452	0.037*
C25	0.61169 (7)	0.14170 (9)	0.32602 (4)	0.01945 (15)
C26	0.54041 (8)	0.14153 (13)	0.37069 (5)	0.0332 (2)
H26	0.472036	0.129888	0.358171	0.040*
C27	0.56853 (11)	0.15836 (16)	0.43390 (5)	0.0441 (3)
H27	0.519172	0.157783	0.464131	0.053*
C28	0.66788 (11)	0.17591 (13)	0.45289 (5)	0.0398 (3)
H28	0.686677	0.188315	0.495932	0.048*
C29	0.73972 (9)	0.17524 (13)	0.40860 (5)	0.0338 (2)
H29	0.808042	0.186338	0.421344	0.041*
C30	0.71190 (8)	0.15833 (11)	0.34550 (5)	0.02687 (19)
H30	0.761481	0.158097	0.315402	0.032*

Atomic displacement parameters (Å^2) for (BF3132)

	U^{11}	U^{22}	U^{33}	U^{12}	U^{13}	U^{23}
O1	0.0227 (3)	0.0141 (3)	0.0292 (3)	0.0006 (2)	0.0019 (2)	-0.0013 (2)
O2	0.0151 (3)	0.0180 (3)	0.0325 (3)	-0.0001 (2)	-0.0010 (2)	-0.0020 (2)
C1	0.0137 (3)	0.0156 (3)	0.0214 (4)	0.0014 (2)	-0.0022 (3)	0.0005 (3)
C2	0.0156 (3)	0.0148 (3)	0.0226 (4)	0.0001 (3)	0.0003 (3)	0.0009 (3)
C3	0.0210 (4)	0.0139 (3)	0.0261 (4)	0.0019 (3)	0.0006 (3)	0.0007 (3)
C4	0.0231 (4)	0.0168 (3)	0.0242 (4)	0.0007 (3)	-0.0014	-0.0032

					(3)	(3)
C5	0.0181 (3)	0.0165 (3)	0.0199 (3)	0.0012 (3)	-0.0022 (3)	-0.0001 (3)
C6	0.0185 (4)	0.0258 (4)	0.0246 (4)	-0.0002 (3)	0.0001 (3)	-0.0023 (3)
C7	0.0233 (4)	0.0336 (5)	0.0372 (5)	0.0002 (4)	0.0052 (4)	-0.0092 (4)
C8	0.0394 (6)	0.0598 (8)	0.0358 (6)	0.0009 (6)	0.0116 (5)	-0.0044 (6)
C9	0.0299 (5)	0.0249 (4)	0.0231 (4)	0.0042 (3)	-0.0054 (3)	0.0025 (3)
C10	0.0204 (4)	0.0158 (3)	0.0212 (4)	0.0004 (3)	0.0007 (3)	0.0023 (3)
C11	0.0244 (4)	0.0392 (5)	0.0269 (5)	0.0007 (4)	0.0052 (4)	0.0045 (4)
C12	0.0383 (6)	0.0491 (7)	0.0253 (5)	0.0015 (5)	0.0092 (4)	0.0055 (4)
C13	0.0439 (6)	0.0307 (5)	0.0209 (4)	-0.0016 (4)	0.0002 (4)	0.0047 (4)
C14	0.0303 (5)	0.0236 (4)	0.0252 (4)	-0.0039 (3)	-0.0045 (3)	0.0030 (3)
C15	0.0216 (4)	0.0214 (4)	0.0240 (4)	-0.0024 (3)	-0.0003 (3)	0.0017 (3)
O3	0.0224 (3)	0.0151 (3)	0.0268 (3)	-0.0001 (2)	0.0040 (2)	-0.0018 (2)
O4	0.0145 (3)	0.0185 (3)	0.0316 (3)	-0.0002 (2)	0.0008 (2)	-0.0019 (2)
C16	0.0129 (3)	0.0153 (3)	0.0192 (3)	0.0007 (2)	-0.0003 (2)	0.0004 (3)
C17	0.0150 (3)	0.0153 (3)	0.0192 (3)	0.0004 (2)	0.0010 (3)	0.0005 (3)
C18	0.0207 (4)	0.0137 (3)	0.0229 (4)	0.0018 (3)	0.0021 (3)	0.0009 (3)
C19	0.0226 (4)	0.0167 (3)	0.0209 (4)	-0.0004 (3)	0.0001 (3)	-0.0026 (3)
C20	0.0179 (3)	0.0165 (3)	0.0170 (3)	0.0003 (3)	-0.0002 (3)	-0.0004 (3)
C21	0.0177 (3)	0.0263 (4)	0.0207 (4)	-0.0006 (3)	0.0025 (3)	-0.0029 (3)
C22	0.0271 (4)	0.0310 (5)	0.0322 (5)	-0.0036 (4)	0.0109 (4)	-0.0120 (4)
C23	0.0334 (5)	0.0470 (6)	0.0267 (5)	-0.0055	0.0088 (4)	-0.0064

				(5)		(4)
C24	0.0280 (4)	0.0248 (4)	0.0204 (4)	0.0008 (3)	-0.0031 (3)	0.0038 (3)
C25	0.0231 (4)	0.0162 (3)	0.0192 (4)	0.0020 (3)	0.0026 (3)	0.0016 (3)
C26	0.0305 (5)	0.0460 (6)	0.0238 (4)	0.0122 (4)	0.0077 (4)	0.0041 (4)
C27	0.0534 (7)	0.0578 (8)	0.0221 (5)	0.0238 (6)	0.0115 (5)	0.0033 (5)
C28	0.0661 (8)	0.0326 (5)	0.0198 (4)	0.0137 (5)	-0.0052 (5)	-0.0025 (4)
C29	0.0435 (6)	0.0303 (5)	0.0264 (5)	-0.0051 (4)	-0.0108 (4)	0.0016 (4)
C30	0.0278 (4)	0.0293 (5)	0.0230 (4)	-0.0053 (4)	-0.0027 (3)	0.0021 (3)

Geometric parameters (Å, º) for (BF3132)

O1—C1	1.2153 (10)	O3—C16	1.2152 (10)
O2—C2	1.4305 (10)	O4—C17	1.4310 (10)
O2—H2O	0.869 (14)	O4—H4O	0.831 (14)
C1—C5	1.5246 (12)	C16—C20	1.5227 (11)
C1—C2	1.5461 (11)	C16—C17	1.5472 (11)
C2—C10	1.5133 (12)	C17—C25	1.5112 (12)
C2—C3	1.5285 (11)	C17—C18	1.5276 (11)
C3—C4	1.5363 (13)	C18—C19	1.5375 (12)
C3—H3A	0.9900	C18—H18A	0.9900
C3—H3B	0.9900	C18—H18B	0.9900
C4—C5	1.5420 (12)	C19—C20	1.5414 (12)
C4—H4A	0.9900	C19—H19A	0.9900
C4—H4B	0.9900	C19—H19B	0.9900
C5—C9	1.5256 (12)	C20—C24	1.5259 (12)
C5—C6	1.5566 (12)	C20—C21	1.5531 (12)
C6—C7	1.4974 (14)	C21—C22	1.4955 (13)
C6—H6A	0.9900	C21—H21A	0.9900
C6—H6B	0.9900	C21—H21B	0.9900
C7—C8	1.3146 (17)	C22—C23	1.3150 (15)

C7—H7	0.9500	C22—H22	0.9500
C8—H8A	0.9500	C23—H23A	0.9500
C8—H8B	0.9500	C23—H23B	0.9500
C9—H9A	0.9800	C24—H24A	0.9800
C9—H9B	0.9800	C24—H24B	0.9800
C9—H9C	0.9800	C24—H24C	0.9800
C10—C11	1.3918 (13)	C25—C26	1.3875 (13)
C10—C15	1.3969 (12)	C25—C30	1.3979 (13)
C11—C12	1.3952 (15)	C26—C27	1.3953 (16)
C11—H11	0.9500	C26—H26	0.9500
C12—C13	1.3844 (17)	C27—C28	1.385 (2)
C12—H12	0.9500	C27—H27	0.9500
C13—C14	1.3875 (15)	C28—C29	1.3870 (18)
C13—H13	0.9500	C28—H28	0.9500
C14—C15	1.3917 (13)	C29—C30	1.3924 (14)
C14—H14	0.9500	C29—H29	0.9500
C15—H15	0.9500	C30—H30	0.9500
C2—O2—H2O	110.3 (9)	C17—O4—H4O	108.8 (9)
O1—C1—C5	125.05 (8)	O3—C16—C20	125.23 (7)
O1—C1—C2	124.68 (8)	O3—C16—C17	124.62 (7)
C5—C1—C2	110.19 (7)	C20—C16—C17	110.08 (6)
O2—C2—C10	113.31 (7)	O4—C17—C25	113.35 (7)
O2—C2—C3	106.43 (7)	O4—C17—C18	106.26 (7)
C10—C2—C3	114.87 (7)	C25—C17—C18	115.47 (7)
O2—C2—C1	103.39 (6)	O4—C17—C16	103.56 (6)
C10—C2—C1	115.01 (7)	C25—C17—C16	114.38 (7)
C3—C2—C1	102.54 (7)	C18—C17—C16	102.51 (6)
C2—C3—C4	103.93 (7)	C17—C18—C19	103.97 (7)
C2—C3—H3A	111.0	C17—C18—H18A	111.0
C4—C3—H3A	111.0	C19—C18—H18A	111.0
C2—C3—H3B	111.0	C17—C18—H18B	111.0
C4—C3—H3B	111.0	C19—C18—H18B	111.0

H3A—C3—H3B	109.0	H18A—C18—H18B	109.0
C3—C4—C5	105.03 (7)	C18—C19—C20	104.73 (7)
C3—C4—H4A	110.7	C18—C19—H19A	110.8
C5—C4—H4A	110.7	C20—C19—H19A	110.8
C3—C4—H4B	110.7	C18—C19—H19B	110.8
C5—C4—H4B	110.7	C20—C19—H19B	110.8
H4A—C4—H4B	108.8	H19A—C19—H19B	108.9
C1—C5—C9	110.82 (7)	C16—C20—C24	110.58 (7)
C1—C5—C4	103.04 (7)	C16—C20—C19	103.22 (6)
C9—C5—C4	113.70 (7)	C24—C20—C19	113.98 (7)
C1—C5—C6	105.39 (7)	C16—C20—C21	105.48 (6)
C9—C5—C6	110.80 (7)	C24—C20—C21	111.03 (7)
C4—C5—C6	112.50 (7)	C19—C20—C21	111.94 (7)
C7—C6—C5	113.52 (8)	C22—C21—C20	113.59 (7)
C7—C6—H6A	108.9	C22—C21—H21A	108.8
C5—C6—H6A	108.9	C20—C21—H21A	108.8
C7—C6—H6B	108.9	C22—C21—H21B	108.8
C5—C6—H6B	108.9	C20—C21—H21B	108.8
H6A—C6—H6B	107.7	H21A—C21—H21B	107.7
C8—C7—C6	124.74 (11)	C23—C22—C21	123.71 (10)
C8—C7—H7	117.6	C23—C22—H22	118.1
C6—C7—H7	117.6	C21—C22—H22	118.1
C7—C8—H8A	120.0	C22—C23—H23A	120.0
C7—C8—H8B	120.0	C22—C23—H23B	120.0
H8A—C8—H8B	120.0	H23A—C23—H23B	120.0
C5—C9—H9A	109.5	C20—C24—H24A	109.5
C5—C9—H9B	109.5	C20—C24—H24B	109.5
H9A—C9—H9B	109.5	H24A—C24—H24B	109.5
C5—C9—H9C	109.5	C20—C24—H24C	109.5
H9A—C9—H9C	109.5	H24A—C24—H24C	109.5
H9B—C9—H9C	109.5	H24B—C24—H24C	109.5
C11—C10—C15	118.91 (9)	C26—C25—C30	118.85 (9)
C11—C10—C2	121.22 (8)	C26—C25—C17	121.24 (8)

C15—C10—C2	119.85 (8)	C30—C25—C17	119.91 (8)
C10—C11—C12	120.17 (10)	C25—C26—C27	120.40 (11)
C10—C11—H11	119.9	C25—C26—H26	119.8
C12—C11—H11	119.9	C27—C26—H26	119.8
C13—C12—C11	120.69 (10)	C28—C27—C26	120.50 (11)
C13—C12—H12	119.7	C28—C27—H27	119.8
C11—C12—H12	119.7	C26—C27—H27	119.8
C12—C13—C14	119.41 (10)	C27—C28—C29	119.50 (10)
C12—C13—H13	120.3	C27—C28—H28	120.3
C14—C13—H13	120.3	C29—C28—H28	120.3
C13—C14—C15	120.25 (9)	C28—C29—C30	120.14 (11)
C13—C14—H14	119.9	C28—C29—H29	119.9
C15—C14—H14	119.9	C30—C29—H29	119.9
C14—C15—C10	120.58 (9)	C29—C30—C25	120.61 (10)
C14—C15—H15	119.7	C29—C30—H30	119.7
C10—C15—H15	119.7	C25—C30—H30	119.7
O1—C1—C2—O2	-83.15 (10)	O3—C16—C17—O4	83.38 (9)
C5—C1—C2—O2	93.74 (7)	C20—C16—C17—O4	-93.83 (7)
O1—C1—C2—C10	40.91 (11)	O3—C16—C17—C25	-40.47 (11)
C5—C1—C2—C10	-142.20 (7)	C20—C16—C17—C25	142.32 (7)
O1—C1—C2—C3	166.31 (8)	O3—C16—C17—C18	-166.22 (8)
C5—C1—C2—C3	-16.80 (8)	C20—C16—C17—C18	16.57 (8)
O2—C2—C3—C4	-74.18 (8)	O4—C17—C18—C19	74.19 (8)
C10—C2—C3—C4	159.56 (7)	C25—C17—C18—C19	-159.20 (7)
C1—C2—C3—C4	34.06 (8)	C16—C17—C18—C19	-34.17 (8)
C2—C3—C4—C5	-39.99 (8)	C17—C18—C19—C20	40.29 (8)
O1—C1—C5—C9	47.77 (11)	O3—C16—C20—C24	-47.32 (11)
C2—C1—C5—C9	-129.11 (7)	C17—C16—C20—	129.87 (7)

		C24	
O1—C1—C5—C4	169.75 (8)	O3—C16—C20—C19	-169.60 (8)
C2—C1—C5—C4	-7.14 (8)	C17—C16—C20—C19	7.59 (8)
O1—C1—C5—C6	-72.14 (10)	O3—C16—C20—C21	72.79 (10)
C2—C1—C5—C6	110.98 (7)	C17—C16—C20—C21	-110.02 (7)
C3—C4—C5—C1	28.50 (8)	C18—C19—C20—C16	-28.93 (8)
C3—C4—C5—C9	148.51 (8)	C18—C19—C20—C24	-148.91 (7)
C3—C4—C5—C6	-84.50 (8)	C18—C19—C20—C21	84.04 (8)
C1—C5—C6—C7	-176.29 (8)	C16—C20—C21—C22	-177.53 (8)
C9—C5—C6—C7	63.79 (10)	C24—C20—C21—C22	-57.72 (10)
C4—C5—C6—C7	-64.74 (10)	C19—C20—C21—C22	70.90 (10)
C5—C6—C7—C8	-108.36 (12)	C20—C21—C22—C23	117.10 (12)
O2—C2—C10—C11	-7.64 (11)	O4—C17—C25—C26	6.74 (12)
C3—C2—C10—C11	114.99 (9)	C18—C17—C25—C26	-116.18 (10)
C1—C2—C10—C11	-126.29 (9)	C16—C17—C25—C26	125.18 (9)
O2—C2—C10—C15	174.15 (7)	O4—C17—C25—C30	-173.86 (8)
C3—C2—C10—C15	-63.21 (10)	C18—C17—C25—C30	63.22 (10)
C1—C2—C10—C15	55.51 (10)	C16—C17—C25—C30	-55.42 (10)
C15—C10—C11—C12	-0.07 (15)	C30—C25—C26—C27	0.28 (16)
C2—C10—C11—C12	-178.29 (9)	C17—C25—C26—C27	179.68 (10)
C10—C11—C12—	0.23 (17)	C25—C26—C27—	0.20 (19)

C13		C28	
C11—C12—C13—C14	-0.02 (17)	C26—C27—C28—C29	-0.61 (19)
C12—C13—C14—C15	-0.35 (16)	C27—C28—C29—C30	0.55 (18)
C13—C14—C15—C10	0.52 (14)	C28—C29—C30—C25	-0.08 (16)
C11—C10—C15—C14	-0.30 (13)	C26—C25—C30—C29	-0.33 (15)
C2—C10—C15—C14	177.94 (8)	C17—C25—C30—C29	-179.75 (9)

Hydrogen-bond geometry (Å, º) for (BF3132)

D—H···*A*	*D*—H	H···*A*	*D*···*A*	*D*—H···*A*
O2—H2*O*···O3	0.869 (14)	1.977 (14)	2.8283 (9)	166.4 (13)
O4—H4*O*···O1	0.831 (14)	1.978 (14)	2.7923 (9)	166.0 (13)

Compound ((-)-12c)-$BzNO_2$

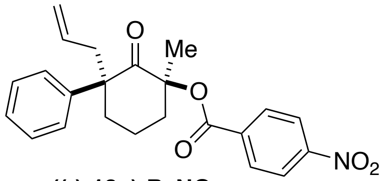

((-)-12c)-$BzNO_2$

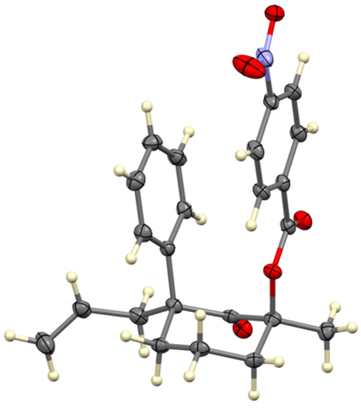

Sample Name: CCDC 2280309

Crystal Growth: Slow evaporation using a mixture of hexanes and dichloromethane.

Crystal data

$C_{23}H_{23}NO_5$	D_x = 1.286 Mg m^{-3}
M_r = 393.42	Cu $K\alpha$ radiation, λ = 1.54184 Å
Orthorhombic, $P2_12_12_1$	Cell parameters from 9880 reflections
a = 6.9282 (2) Å	θ = 2.3–69.1°
b = 7.6886 (2) Å	μ = 0.74 mm^{-1}
c = 38.1575 (12) Å	T = 90 K
V = 2032.58 (10) Å^3	Lath, colourless
Z = 4	0.36 × 0.10 × 0.03 mm
F(000) = 832	

Data collection

Bruker Kappa APEX-II DUO diffractometer	3790 independent reflections
Radiation source: IμS microfocus	3731 reflections with $I > 2\sigma(I)$
QUAZAR multilayer optics monochromator	R_{int} = 0.028
ϕ and ω scans	θ_{max} = 69.3°, θ_{min} = 2.3°

Absorption correction: multi-scan *SADABS* (Krause *et al.*, 2015)	h = -8 – 8
T_{min} = 0.862, T_{max} = 0.978	k = -9 – 9
15905 measured reflections	l = -46 – 45

Refinement

Refinement on F^2	Hydrogen site location: inferred from neighbouring sites
Least-squares matrix: full	H-atom parameters constrained
$R[F^2 > 2\sigma(F^2)]$ = 0.026	$w = 1/[\sigma^2(F_o^2) + (0.0381P)^2 + 0.2975P]$ where $P = (F_o^2 + 2F_c^2)/3$
$wR(F^2)$ = 0.069	$(\Delta/\sigma)_{max}$ < 0.001
S = 1.08	$\Delta\rho_{max}$ = 0.15 e Å^{-3}
3790 reflections	$\Delta\rho_{min}$ = -0.15 e Å^{-3}
263 parameters	Absolute structure: Flack x determined using 1526 quotients [(I+)-(I-)]/[(I+)+(I-)] (Parsons, Flack and Wagner, Acta Cryst. B69 (2013) 249-259).
0 restraints	Absolute structure parameter: 0.07 (4)

Fractional atomic coordinates and isotropic or equivalent isotropic displacement parameters (Å^2) for (SCP2239)

	x	y	z	U_{iso}*/U_{eq}
O1	0.10316 (15)	0.25086 (15)	0.42925 (3)	0.0230 (2)
O2	0.41053 (15)	0.07825 (14)	0.36641 (3)	0.0179 (2)
O3	0.17306 (16)	0.22976 (15)	0.33939 (3)	0.0219 (2)
O4	1.0353 (2)	0.2080 (3)	0.23195 (3)	0.0506 (4)
O5	0.82589 (18)	0.36335 (17)	0.20426 (3)	0.0293 (3)
N1	0.8752 (2)	0.2732 (2)	0.22925 (3)	0.0266 (3)
C1	0.2634 (2)	0.2178 (2)	0.41872 (4)	0.0177 (3)
C2	0.2987 (2)	0.0461 (2)	0.39830 (4)	0.0182 (3)
C3	0.4374 (2)	-0.0631 (2)	0.42049 (4)	0.0205 (3)
H3A	0.372755	-0.095899	0.442662	0.025*

H3B	0.468997	-0.171519	0.407708	0.025*
C4	0.6233 (2)	0.0336 (2)	0.42887 (4)	0.0206 (3)
H4A	0.692598	0.059990	0.406813	0.025*
H4B	0.707554	-0.041430	0.443388	0.025*
C5	0.5820 (2)	0.2025 (2)	0.44845 (4)	0.0201 (3)
H5A	0.705525	0.264408	0.452310	0.024*
H5B	0.527700	0.173724	0.471731	0.024*
C6	0.4408 (2)	0.3265 (2)	0.42933 (4)	0.0179 (3)
C7	0.3746 (2)	0.4682 (2)	0.45570 (4)	0.0219 (3)
H7A	0.278597	0.544495	0.444167	0.026*
H7B	0.310522	0.411335	0.475860	0.026*
C8	0.5383 (3)	0.5774 (2)	0.46888 (4)	0.0238 (3)
H8	0.620520	0.629102	0.451905	0.029*
C9	0.5775 (3)	0.6074 (3)	0.50211 (5)	0.0366 (4)
H9A	0.498742	0.558099	0.519910	0.044*
H9B	0.684410	0.678420	0.508278	0.044*
C10	0.1138 (2)	-0.0516 (2)	0.39029 (4)	0.0228 (3)
H10A	0.021611	0.027070	0.378969	0.034*
H10B	0.058082	-0.095956	0.412144	0.034*
H10C	0.142090	-0.149176	0.374577	0.034*
C11	0.3333 (2)	0.16925 (19)	0.33985 (4)	0.0176 (3)
C12	0.4772 (2)	0.19000 (19)	0.31087 (4)	0.0175 (3)
C13	0.6656 (2)	0.1285 (2)	0.31413 (4)	0.0199 (3)
H13	0.703797	0.068035	0.334701	0.024*
C14	0.7973 (2)	0.1557 (2)	0.28731 (4)	0.0221 (3)
H14	0.926426	0.115190	0.289223	0.027*
C15	0.7357 (2)	0.2432 (2)	0.25771 (4)	0.0207 (3)
C16	0.5488 (2)	0.3045 (2)	0.25363 (4)	0.0217 (3)
H16	0.510722	0.363405	0.232861	0.026*
C17	0.4195 (2)	0.2775 (2)	0.28066 (4)	0.0206 (3)
H17	0.290795	0.318850	0.278645	0.025*
C18	0.5272 (2)	0.41746 (19)	0.39698 (4)	0.0173 (3)
C19	0.4051 (2)	0.5175 (2)	0.37593 (4)	0.0192 (3)

H19	0.271171	0.522106	0.381152	0.023*
C20	0.4761 (3)	0.6101 (2)	0.34756 (4)	0.0229 (3)
H20	0.390463	0.676806	0.333493	0.027*
C21	0.6713 (3)	0.6060 (2)	0.33958 (4)	0.0247 (3)
H21	0.720566	0.670503	0.320317	0.030*
C22	0.7936 (2)	0.5063 (2)	0.36018 (4)	0.0249 (3)
H22	0.927281	0.501257	0.354731	0.030*
C23	0.7228 (2)	0.4134 (2)	0.38872 (4)	0.0217 (3)
H23	0.808820	0.346688	0.402698	0.026*

Atomic displacement parameters (Å^2) for (SCP2239)

	U^{11}	U^{22}	U^{33}	U^{12}	U^{13}	U^{23}
O1	0.0166 (5)	0.0295 (6)	0.0229 (5)	0.0045 (5)	0.0013 (4)	-0.0011 (5)
O2	0.0158 (5)	0.0225 (5)	0.0153 (5)	0.0039 (4)	0.0014 (4)	0.0014 (4)
O3	0.0166 (5)	0.0271 (6)	0.0219 (5)	0.0051 (5)	-0.0015 (4)	-0.0008 (4)
O4	0.0225 (6)	0.1012 (13)	0.0281 (7)	0.0125 (8)	0.0063 (6)	0.0117 (7)
O5	0.0328 (6)	0.0358 (6)	0.0192 (5)	-0.0093 (6)	-0.0002 (5)	0.0046 (5)
N1	0.0210 (7)	0.0417 (8)	0.0172 (6)	-0.0049 (6)	-0.0008 (5)	-0.0030 (6)
C1	0.0170 (7)	0.0220 (8)	0.0140 (6)	0.0033 (6)	-0.0009 (6)	0.0044 (5)
C2	0.0149 (7)	0.0218 (7)	0.0178 (7)	0.0005 (6)	0.0026 (6)	0.0011 (6)
C3	0.0218 (8)	0.0198 (7)	0.0199 (7)	0.0022 (6)	0.0010 (6)	0.0036 (6)
C4	0.0178 (7)	0.0228 (7)	0.0213 (7)	0.0049 (6)	-0.0011 (6)	0.0046 (6)
C5	0.0184 (7)	0.0234 (8)	0.0184 (7)	0.0024 (6)	-0.0026 (6)	0.0039 (6)
C6	0.0163 (7)	0.0203 (7)	0.0170 (7)	0.0036 (6)	-0.0016 (6)	0.0007 (6)
C7	0.0242 (8)	0.0241 (8)	0.0175 (7)	0.0048 (6)	-0.0009 (6)	0.0002 (6)

C8	0.0275 (8)	0.0222 (7)	0.0217 (7)	0.0034 (7)	-0.0036 (6)	0.0006 (6)
C9	0.0332 (10)	0.0517 (11)	0.0248 (8)	-0.0055 (9)	-0.0056 (7)	-0.0045 (8)
C10	0.0184 (7)	0.0257 (8)	0.0242 (7)	-0.0022 (6)	0.0020 (6)	-0.0011 (6)
C11	0.0182 (7)	0.0178 (7)	0.0167 (7)	0.0017 (6)	-0.0019 (6)	-0.0026 (5)
C12	0.0175 (7)	0.0180 (7)	0.0170 (7)	0.0009 (6)	-0.0008 (6)	-0.0027 (5)
C13	0.0195 (7)	0.0242 (7)	0.0161 (7)	0.0028 (6)	-0.0030 (6)	-0.0003 (6)
C14	0.0164 (7)	0.0301 (8)	0.0198 (7)	0.0030 (6)	-0.0009 (6)	-0.0032 (6)
C15	0.0197 (7)	0.0267 (8)	0.0157 (7)	-0.0046 (6)	0.0014 (6)	-0.0030 (6)
C16	0.0230 (8)	0.0241 (8)	0.0181 (7)	-0.0009 (7)	-0.0050 (6)	0.0026 (6)
C17	0.0174 (7)	0.0229 (7)	0.0214 (7)	0.0026 (6)	-0.0030 (6)	0.0004 (6)
C18	0.0201 (7)	0.0159 (7)	0.0158 (6)	-0.0002 (6)	-0.0016 (6)	-0.0005 (5)
C19	0.0191 (7)	0.0202 (7)	0.0183 (7)	0.0029 (6)	-0.0018 (6)	-0.0032 (6)
C20	0.0295 (8)	0.0206 (7)	0.0186 (7)	0.0053 (7)	-0.0030 (6)	0.0009 (6)
C21	0.0316 (9)	0.0221 (8)	0.0202 (7)	-0.0002 (7)	0.0049 (7)	0.0020 (6)
C22	0.0202 (8)	0.0255 (8)	0.0291 (8)	0.0003 (7)	0.0029 (7)	-0.0010 (7)
C23	0.0190 (7)	0.0220 (8)	0.0241 (7)	0.0028 (6)	-0.0023 (6)	0.0007 (6)

Geometric parameters (Å, º) for (SCP2239)

O1—C1	1.2077 (19)	C9—H9A	0.9500
O2—C11	1.3426 (18)	C9—H9B	0.9500
O2—C2	1.4636 (17)	C10—H10A	0.9800

O3—C11	1.204 (2)	C10—H10B	0.9800
O4—N1	1.221 (2)	C10—H10C	0.9800
O5—N1	1.2275 (19)	C11—C12	1.497 (2)
N1—C15	1.472 (2)	C12—C17	1.393 (2)
C1—C6	1.541 (2)	C12—C13	1.394 (2)
C1—C2	1.552 (2)	C13—C14	1.387 (2)
C2—C10	1.516 (2)	C13—H13	0.9500
C2—C3	1.532 (2)	C14—C15	1.382 (2)
C3—C4	1.521 (2)	C14—H14	0.9500
C3—H3A	0.9900	C15—C16	1.387 (2)
C3—H3B	0.9900	C16—C17	1.381 (2)
C4—C5	1.526 (2)	C16—H16	0.9500
C4—H4A	0.9900	C17—H17	0.9500
C4—H4B	0.9900	C18—C23	1.392 (2)
C5—C6	1.549 (2)	C18—C19	1.397 (2)
C5—H5A	0.9900	C19—C20	1.386 (2)
C5—H5B	0.9900	C19—H19	0.9500
C6—C18	1.540 (2)	C20—C21	1.387 (3)
C6—C7	1.553 (2)	C20—H20	0.9500
C7—C8	1.498 (2)	C21—C22	1.387 (2)
C7—H7A	0.9900	C21—H21	0.9500
C7—H7B	0.9900	C22—C23	1.392 (2)
C8—C9	1.317 (2)	C22—H22	0.9500
C8—H8	0.9500	C23—H23	0.9500
C11—O2—C2	120.30 (11)	C8—C9—H9B	120.0
O4—N1—O5	123.39 (14)	H9A—C9—H9B	120.0
O4—N1—C15	118.03 (14)	C2—C10—H10A	109.5
O5—N1—C15	118.58 (14)	C2—C10—H10B	109.5
O1—C1—C6	122.13 (14)	H10A—C10—H10B	109.5
O1—C1—C2	119.40 (14)	C2—C10—H10C	109.5
C6—C1—C2	117.88 (12)	H10A—C10—H10C	109.5
O2—C2—C10	111.31 (12)	H10B—C10—H10C	109.5

O2—C2—C3	102.70 (11)	O3—C11—O2	125.44 (14)
C10—C2—C3	111.71 (13)	O3—C11—C12	124.19 (14)
O2—C2—C1	110.92 (12)	O2—C11—C12	110.35 (12)
C10—C2—C1	112.93 (12)	C17—C12—C13	120.39 (14)
C3—C2—C1	106.71 (12)	C17—C12—C11	118.14 (13)
C4—C3—C2	112.31 (13)	C13—C12—C11	121.44 (13)
C4—C3—H3A	109.1	C14—C13—C12	119.96 (14)
C2—C3—H3A	109.1	C14—C13—H13	120.0
C4—C3—H3B	109.1	C12—C13—H13	120.0
C2—C3—H3B	109.1	C15—C14—C13	118.22 (14)
H3A—C3—H3B	107.9	C15—C14—H14	120.9
C3—C4—C5	111.13 (13)	C13—C14—H14	120.9
C3—C4—H4A	109.4	C14—C15—C16	123.08 (14)
C5—C4—H4A	109.4	C14—C15—N1	118.42 (14)
C3—C4—H4B	109.4	C16—C15—N1	118.51 (14)
C5—C4—H4B	109.4	C17—C16—C15	118.05 (14)
H4A—C4—H4B	108.0	C17—C16—H16	121.0
C4—C5—C6	114.32 (12)	C15—C16—H16	121.0
C4—C5—H5A	108.7	C16—C17—C12	120.30 (14)
C6—C5—H5A	108.7	C16—C17—H17	119.8
C4—C5—H5B	108.7	C12—C17—H17	119.8
C6—C5—H5B	108.7	C23—C18—C19	118.13 (14)
H5A—C5—H5B	107.6	C23—C18—C6	123.36 (13)
C18—C6—C1	110.25 (12)	C19—C18—C6	118.41 (13)
C18—C6—C5	114.32 (12)	C20—C19—C18	121.17 (15)
C1—C6—C5	107.09 (12)	C20—C19—H19	119.4
C18—C6—C7	108.40 (12)	C18—C19—H19	119.4
C1—C6—C7	108.38 (12)	C19—C20—C21	120.36 (15)
C5—C6—C7	108.26 (12)	C19—C20—H20	119.8
C8—C7—C6	112.78 (13)	C21—C20—H20	119.8
C8—C7—H7A	109.0	C22—C21—C20	118.95 (15)
C6—C7—H7A	109.0	C22—C21—H21	120.5
C8—C7—H7B	109.0	C20—C21—H21	120.5

C6—C7—H7B	109.0	C21—C22—C23	120.81 (16)
H7A—C7—H7B	107.8	C21—C22—H22	119.6
C9—C8—C7	125.28 (16)	C23—C22—H22	119.6
C9—C8—H8	117.4	C22—C23—C18	120.59 (15)
C7—C8—H8	117.4	C22—C23—H23	119.7
C8—C9—H9A	120.0	C18—C23—H23	119.7
C11—O2—C2—C10	59.03 (17)	O3—C11—C12—C13	174.87 (15)
C11—O2—C2—C3	178.70 (12)	O2—C11—C12—C13	-3.6 (2)
C11—O2—C2—C1	-67.61 (16)	C17—C12—C13—C14	0.5 (2)
O1—C1—C2—O2	132.75 (14)	C11—C12—C13—C14	-177.57 (14)
C6—C1—C2—O2	-55.92 (16)	C12—C13—C14—C15	-0.4 (2)
O1—C1—C2—C10	7.0 (2)	C13—C14—C15—C16	-0.1 (2)
C6—C1—C2—C10	178.35 (12)	C13—C14—C15—N1	179.65 (15)
O1—C1—C2—C3	-116.12 (15)	O4—N1—C15—C14	5.4 (2)
C6—C1—C2—C3	55.22 (16)	O5—N1—C15—C14	-173.94 (15)
O2—C2—C3—C4	60.86 (15)	O4—N1—C15—C16	-174.84 (17)
C10—C2—C3—C4	-179.75 (12)	O5—N1—C15—C16	5.8 (2)
C1—C2—C3—C4	-55.88 (16)	C14—C15—C16—C17	0.5 (2)
C2—C3—C4—C5	58.52 (16)	N1—C15—C16—C17	-179.23 (14)
C3—C4—C5—C6	-55.49 (17)	C15—C16—C17—C12	-0.4 (2)
O1—C1—C6—C18	-115.67 (16)	C13—C12—C17—C16	-0.1 (2)
C2—C1—C6—C18	73.24 (16)	C11—C12—C17—C16	178.06 (14)

O1—C1—C6—C5	119.40 (15)	C1—C6—C18—C23	-131.09 (15)
C2—C1—C6—C5	-51.69 (16)	C5—C6—C18—C23	-10.4 (2)
O1—C1—C6—C7	2.82 (19)	C7—C6—C18—C23	110.43 (16)
C2—C1—C6—C7	-168.27 (12)	C1—C6—C18—C19	52.72 (17)
C4—C5—C6—C18	-72.62 (17)	C5—C6—C18—C19	173.40 (13)
C4—C5—C6—C1	49.81 (16)	C7—C6—C18—C19	-65.76 (17)
C4—C5—C6—C7	166.47 (13)	C23—C18—C19—C20	0.1 (2)
C18—C6—C7—C8	-62.66 (16)	C6—C18—C19—C20	176.49 (14)
C1—C6—C7—C8	177.68 (12)	C18—C19—C20—C21	-0.4 (2)
C5—C6—C7—C8	61.86 (16)	C19—C20—C21—C22	0.8 (2)
C6—C7—C8—C9	-127.93 (19)	C20—C21—C22—C23	-0.9 (3)
C2—O2—C11—O3	-0.4 (2)	C21—C22—C23—C18	0.6 (2)
C2—O2—C11—C12	178.02 (12)	C19—C18—C23—C22	-0.2 (2)
O3—C11—C12—C17	-3.3 (2)	C6—C18—C23—C22	-176.43 (14)
O2—C11—C12—C17	178.30 (13)		

Compound ((-)-12e)-$BzNO_2$

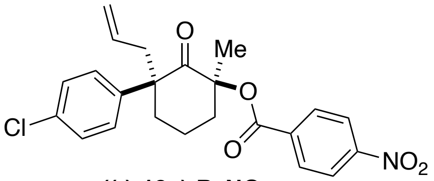

((-)-12e)-$BzNO_2$

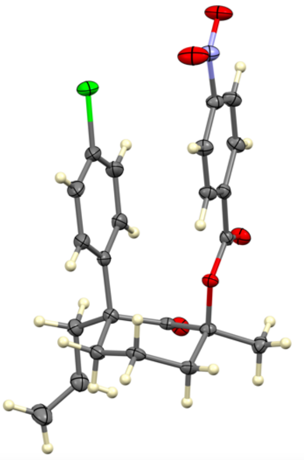

Sample Name: CCDC 2280310

Crystal Growth: Slow evaporation using a mixture of hexanes and dichloromethane.

Crystal data

$C_{23}H_{22}ClNO_5$	$F(000) = 896$
$M_r = 427.86$	$D_x = 1.368$ Mg m^{-3}
Monoclinic, *I*2	Cu *K*α radiation, λ = 1.54184 Å
a = 10.2913 (5) Å	Cell parameters from 9921 reflections
b = 7.2116 (4) Å	θ = 3.2–69.2°
c = 28.3090 (16) Å	μ = 1.93 mm^{-1}
β = 98.699 (2)°	*T* = 90 K
V = 2076.83 (19) Å^3	Needle, colourless
Z = 4	0.27 × 0.07 × 0.06 mm

Data collection

Bruker Kappa APEX-II DUO diffractometer	3840 independent reflections
Radiation source: IμS microfocus	3747 reflections with $I > 2\sigma(I)$
QUAZAR multilayer optics monochromator	$R_{int} = 0.032$
ϕ and ω scans	$\theta_{max} = 69.4°$, $\theta_{min} = 3.2°$

Absorption correction: multi-scan *SADABS* (Krause *et al.*, 2015)	$h = -12 – 12$
T_{min} = 0.753, T_{max} = 0.893	$k = -8 – 8$
15228 measured reflections	$l = -34 – 34$

Refinement

Refinement on F^2	Hydrogen site location: inferred from neighbouring sites
Least-squares matrix: full	H-atom parameters constrained
$R[F^2 > 2\sigma(F^2)] = 0.026$	$w = 1/[\sigma^2(F_o^2) + (0.0367P)^2 + 0.2615P]$ where $P = (F_o^2 + 2F_c^2)/3$
$wR(F^2) = 0.066$	$(\Delta/\sigma)_{max} = 0.001$
$S = 1.05$	$\Delta\rho_{max}$ = 0.12 e Å^{-3}
3840 reflections	$\Delta\rho_{min}$ = -0.18 e Å^{-3}
272 parameters	Absolute structure: Flack x determined using 1655 quotients [(I+)-(I-)]/[(I+)+(I-)] (Parsons, Flack and Wagner, Acta Cryst. B69 (2013) 249-259).
1 restraint	Absolute structure parameter: 0.021 (5)

Fractional atomic coordinates and isotropic or equivalent isotropic displacement parameters (Å^2) for (CEB1114)

	x	*y*	*z*	U_{iso}*/U_{eq}
Cl1	0.09676 (5)	0.44853 (11)	0.56095 (2)	0.04576 (18)
O1	0.65823 (12)	1.01622 (18)	0.64683 (5)	0.0213 (3)
O2	0.61020 (11)	0.58216 (17)	0.68311 (4)	0.0155 (3)
O3	0.47202 (12)	0.80168 (19)	0.70339 (5)	0.0220 (3)
O4	0.11564 (14)	-0.0625 (2)	0.67023 (6)	0.0380 (4)
O5	-0.01908 (14)	0.1487 (2)	0.68632 (6)	0.0331 (3)
N1	0.09041 (15)	0.0971 (2)	0.68047 (6)	0.0237 (3)
C1	0.67088 (15)	0.8530 (2)	0.63885 (6)	0.0160 (3)
C2	0.71656 (17)	0.7164 (3)	0.68040 (6)	0.0163 (3)
C3	0.82835 (16)	0.5968 (3)	0.66741 (6)	0.0182 (3)

H3A	0.906035	0.676282	0.665852	0.022*
H3B	0.852935	0.503668	0.692883	0.022*
C4	0.79180 (16)	0.4976 (3)	0.61993 (6)	0.0198 (4)
H4A	0.715783	0.414888	0.621458	0.024*
H4B	0.866572	0.420532	0.613297	0.024*
C5	0.75701 (17)	0.6389 (3)	0.57998 (6)	0.0196 (4)
H5A	0.730485	0.571332	0.549602	0.024*
H5B	0.836755	0.711507	0.576670	0.024*
C6	0.64611 (16)	0.7745 (2)	0.58747 (6)	0.0175 (4)
C7	0.75733 (18)	0.8165 (3)	0.72731 (6)	0.0215 (4)
H7A	0.687244	0.901619	0.733159	0.032*
H7B	0.838057	0.886914	0.725810	0.032*
H7C	0.773082	0.725818	0.753325	0.032*
C8	0.64389 (17)	0.9365 (3)	0.55117 (6)	0.0231 (4)
H8A	0.624828	0.885158	0.518420	0.028*
H8B	0.571092	1.021497	0.555526	0.028*
C9	0.7690 (2)	1.0465 (3)	0.55542 (7)	0.0246 (4)
H9	0.797268	1.107992	0.584868	0.030*
C10	0.8421 (2)	1.0642 (4)	0.52166 (8)	0.0377 (5)
H10A	0.817114	1.004760	0.491706	0.045*
H10B	0.920152	1.136359	0.527096	0.045*
C11	0.50845 (17)	0.6860 (3)	0.58005 (6)	0.0189 (4)
C12	0.40188 (18)	0.7916 (3)	0.58992 (6)	0.0232 (4)
H12	0.416413	0.915485	0.600862	0.028*
C13	0.27534 (19)	0.7198 (3)	0.58411 (7)	0.0285 (4)
H13	0.204157	0.792610	0.591511	0.034*
C14	0.25479 (19)	0.5411 (4)	0.56741 (7)	0.0308 (5)
C15	0.35695 (19)	0.4336 (3)	0.55620 (6)	0.0290 (4)
H15	0.340950	0.311430	0.544134	0.035*
C16	0.48385 (18)	0.5066 (3)	0.56282 (6)	0.0237 (4)
H16	0.554609	0.432772	0.555469	0.028*
C17	0.49530 (17)	0.6427 (3)	0.69432 (6)	0.0156 (3)
C18	0.39579 (16)	0.4914 (3)	0.69280 (6)	0.0164 (3)

C19	0.42254 (16)	0.3077 (3)	0.68267 (6)	0.0187 (4)
H19	0.508684	0.271997	0.678172	0.022*
C20	0.32232 (18)	0.1765 (3)	0.67918 (7)	0.0207 (4)
H20	0.338828	0.050441	0.672406	0.025*
C21	0.19827 (17)	0.2340 (3)	0.68579 (6)	0.0190 (4)
C22	0.16961 (16)	0.4144 (3)	0.69696 (6)	0.0213 (4)
H22	0.083821	0.448634	0.702232	0.026*
C23	0.26973 (17)	0.5435 (3)	0.70021 (6)	0.0197 (4)
H23	0.252687	0.668895	0.707562	0.024*

Atomic displacement parameters (Å^2) for (CEB1114)

	U^{11}	U^{22}	U^{33}	U^{12}	U^{13}	U^{23}
Cl1	0.0213 (2)	0.0810 (5)	0.0353 (3)	-0.0219 (3)	0.00549 (17)	-0.0133 (3)
O1	0.0246 (6)	0.0150 (6)	0.0253 (6)	0.0017 (5)	0.0075 (5)	0.0003 (5)
O2	0.0131 (5)	0.0148 (6)	0.0192 (5)	0.0000 (5)	0.0043 (4)	-0.0008 (5)
O3	0.0209 (6)	0.0161 (7)	0.0302 (7)	0.0019 (5)	0.0080 (5)	-0.0019 (5)
O4	0.0262 (7)	0.0181 (7)	0.0699 (11)	-0.0026 (6)	0.0085 (7)	-0.0056 (8)
O5	0.0165 (7)	0.0286 (8)	0.0556 (9)	-0.0022 (5)	0.0102 (6)	-0.0011 (7)
N1	0.0182 (7)	0.0209 (8)	0.0320 (8)	-0.0017 (7)	0.0038 (6)	0.0025 (7)
C1	0.0114 (7)	0.0174 (9)	0.0197 (8)	-0.0023 (6)	0.0041 (6)	0.0005 (7)
C2	0.0148 (8)	0.0150 (8)	0.0189 (8)	-0.0016 (7)	0.0026 (6)	0.0009 (7)
C3	0.0126 (7)	0.0196 (9)	0.0224 (8)	0.0007 (7)	0.0030 (6)	0.0013 (7)
C4	0.0157 (7)	0.0172 (9)	0.0280 (9)	0.0000 (7)	0.0085 (6)	-0.0024 (7)
C5	0.0175 (8)	0.0217 (9)	0.0208 (8)	-0.0020 (7)	0.0070 (6)	-0.0029 (7)
C6	0.0160 (7)	0.0196 (9)	0.0173 (8)	-0.0003	0.0038 (6)	0.0001 (7)

				(7)		
C7	0.0231 (8)	0.0216 (9)	0.0192 (8)	-0.0036 (7)	0.0007 (7)	-0.0028 (7)
C8	0.0221 (8)	0.0273 (10)	0.0199 (8)	-0.0022 (8)	0.0037 (6)	0.0048 (8)
C9	0.0306 (9)	0.0201 (10)	0.0237 (9)	-0.0034 (8)	0.0056 (7)	0.0018 (7)
C10	0.0421 (11)	0.0399 (13)	0.0341 (11)	-0.0175 (10)	0.0155 (9)	-0.0046 (10)
C11	0.0163 (8)	0.0269 (10)	0.0132 (7)	-0.0027 (7)	0.0013 (6)	0.0035 (7)
C12	0.0194 (8)	0.0317 (11)	0.0180 (8)	0.0011 (8)	0.0019 (6)	0.0034 (7)
C13	0.0174 (9)	0.0466 (13)	0.0216 (9)	0.0017 (9)	0.0028 (7)	0.0025 (9)
C14	0.0194 (8)	0.0549 (15)	0.0177 (8)	-0.0118 (9)	0.0014 (7)	-0.0021 (9)
C15	0.0263 (9)	0.0389 (12)	0.0217 (9)	-0.0137 (9)	0.0035 (7)	-0.0063 (9)
C16	0.0203 (8)	0.0326 (11)	0.0182 (8)	-0.0056 (8)	0.0032 (6)	-0.0032 (8)
C17	0.0158 (8)	0.0175 (9)	0.0139 (7)	0.0027 (7)	0.0036 (6)	0.0013 (6)
C18	0.0161 (7)	0.0181 (9)	0.0151 (7)	0.0016 (7)	0.0030 (6)	0.0024 (6)
C19	0.0152 (7)	0.0185 (9)	0.0226 (8)	0.0029 (7)	0.0035 (6)	0.0013 (7)
C20	0.0186 (9)	0.0172 (9)	0.0263 (9)	0.0022 (7)	0.0035 (7)	0.0001 (7)
C21	0.0170 (8)	0.0179 (9)	0.0221 (8)	-0.0021 (7)	0.0028 (6)	0.0029 (7)
C22	0.0155 (8)	0.0224 (9)	0.0266 (9)	0.0039 (7)	0.0055 (6)	0.0022 (8)
C23	0.0195 (8)	0.0166 (9)	0.0239 (8)	0.0033 (7)	0.0064 (6)	0.0010 (7)

Geometric parameters (Å, º) for (CEB1114)

Cl1—C14	1.742 (2)	C8—H8A	0.9900
O1—C1	1.210 (2)	C8—H8B	0.9900
O2—C17	1.343 (2)	C9—C10	1.309 (3)
O2—C2	1.472 (2)	C9—H9	0.9500

O3—C17	1.207 (2)	C10—H10A	0.9500
O4—N1	1.224 (3)	C10—H10B	0.9500
O5—N1	1.221 (2)	C11—C16	1.392 (3)
N1—C21	1.476 (2)	C11—C12	1.398 (3)
C1—C6	1.546 (2)	C12—C13	1.388 (3)
C1—C2	1.552 (2)	C12—H12	0.9500
C2—C7	1.514 (2)	C13—C14	1.378 (4)
C2—C3	1.527 (2)	C13—H13	0.9500
C3—C4	1.519 (2)	C14—C15	1.381 (3)
C3—H3A	0.9900	C15—C16	1.394 (3)
C3—H3B	0.9900	C15—H15	0.9500
C4—C5	1.524 (3)	C16—H16	0.9500
C4—H4A	0.9900	C17—C18	1.493 (2)
C4—H4B	0.9900	C18—C19	1.392 (3)
C5—C6	1.542 (2)	C18—C23	1.397 (2)
C5—H5A	0.9900	C19—C20	1.392 (3)
C5—H5B	0.9900	C19—H19	0.9500
C6—C11	1.539 (2)	C20—C21	1.382 (3)
C6—C8	1.554 (2)	C20—H20	0.9500
C7—H7A	0.9800	C21—C22	1.381 (3)
C7—H7B	0.9800	C22—C23	1.381 (3)
C7—H7C	0.9800	C22—H22	0.9500
C8—C9	1.502 (3)	C23—H23	0.9500
C17—O2—C2	119.12 (14)	C6—C8—H8B	108.6
O5—N1—O4	123.46 (17)	H8A—C8—H8B	107.6
O5—N1—C21	118.51 (17)	C10—C9—C8	124.98 (19)
O4—N1—C21	118.02 (15)	C10—C9—H9	117.5
O1—C1—C6	121.70 (16)	C8—C9—H9	117.5
O1—C1—C2	120.32 (16)	C9—C10—H10A	120.0
C6—C1—C2	117.97 (15)	C9—C10—H10B	120.0
O2—C2—C7	112.19 (14)	H10A—C10—H10B	120.0
O2—C2—C3	103.54 (14)	C16—C11—C12	118.03 (17)

C7—C2—C3	111.20 (14)	C16—C11—C6	123.22 (16)
O2—C2—C1	108.33 (13)	C12—C11—C6	118.73 (17)
C7—C2—C1	112.00 (15)	C13—C12—C11	121.6 (2)
C3—C2—C1	109.19 (14)	C13—C12—H12	119.2
C4—C3—C2	112.61 (14)	C11—C12—H12	119.2
C4—C3—H3A	109.1	C14—C13—C12	118.82 (19)
C2—C3—H3A	109.1	C14—C13—H13	120.6
C4—C3—H3B	109.1	C12—C13—H13	120.6
C2—C3—H3B	109.1	C13—C14—C15	121.40 (19)
H3A—C3—H3B	107.8	C13—C14—Cl1	119.12 (17)
C3—C4—C5	109.93 (15)	C15—C14—Cl1	119.47 (18)
C3—C4—H4A	109.7	C14—C15—C16	119.2 (2)
C5—C4—H4A	109.7	C14—C15—H15	120.4
C3—C4—H4B	109.7	C16—C15—H15	120.4
C5—C4—H4B	109.7	C11—C16—C15	120.99 (19)
H4A—C4—H4B	108.2	C11—C16—H16	119.5
C4—C5—C6	114.68 (14)	C15—C16—H16	119.5
C4—C5—H5A	108.6	O3—C17—O2	124.72 (16)
C6—C5—H5A	108.6	O3—C17—C18	122.90 (15)
C4—C5—H5B	108.6	O2—C17—C18	112.35 (15)
C6—C5—H5B	108.6	C19—C18—C23	120.28 (17)
H5A—C5—H5B	107.6	C19—C18—C17	123.07 (15)
C11—C6—C5	113.80 (15)	C23—C18—C17	116.60 (16)
C11—C6—C1	107.32 (13)	C20—C19—C18	119.60 (16)
C5—C6—C1	109.97 (13)	C20—C19—H19	120.2
C11—C6—C8	107.48 (14)	C18—C19—H19	120.2
C5—C6—C8	108.86 (14)	C21—C20—C19	118.47 (17)
C1—C6—C8	109.30 (15)	C21—C20—H20	120.8
C2—C7—H7A	109.5	C19—C20—H20	120.8
C2—C7—H7B	109.5	C22—C21—C20	123.17 (17)
H7A—C7—H7B	109.5	C22—C21—N1	118.04 (15)
C2—C7—H7C	109.5	C20—C21—N1	118.79 (17)
H7A—C7—H7C	109.5	C23—C22—C21	117.85 (16)

H7B—C7—H7C	109.5	C23—C22—H22	121.1
C9—C8—C6	114.68 (15)	C21—C22—H22	121.1
C9—C8—H8A	108.6	C22—C23—C18	120.60 (17)
C6—C8—H8A	108.6	C22—C23—H23	119.7
C9—C8—H8B	108.6	C18—C23—H23	119.7
C17—O2—C2—C7	59.63 (19)	C8—C6—C11—C12	-64.85 (19)
C17—O2—C2—C3	179.63 (13)	C16—C11—C12—C13	1.7 (3)
C17—O2—C2—C1	-64.52 (18)	C6—C11—C12—C13	-179.80 (16)
O1—C1—C2—O2	116.84 (17)	C11—C12—C13—C14	-1.1 (3)
C6—C1—C2—O2	-64.74 (18)	C12—C13—C14—C15	-0.4 (3)
O1—C1—C2—C7	-7.4 (2)	C12—C13—C14—Cl1	178.94 (15)
C6—C1—C2—C7	171.00 (14)	C13—C14—C15—C16	1.2 (3)
O1—C1—C2—C3	-131.04 (17)	Cl1—C14—C15—C16	-178.08 (15)
C6—C1—C2—C3	47.38 (19)	C12—C11—C16—C15	-0.8 (3)
O2—C2—C3—C4	60.60 (17)	C6—C11—C16—C15	-179.24 (17)
C7—C2—C3—C4	-178.72 (15)	C14—C15—C16—C11	-0.6 (3)
C1—C2—C3—C4	-54.63 (19)	C2—O2—C17—O3	-1.3 (2)
C2—C3—C4—C5	60.23 (18)	C2—O2—C17—C18	176.71 (13)
C3—C4—C5—C6	-56.34 (19)	O3—C17—C18—C19	-179.54 (17)
C4—C5—C6—C11	-73.49 (19)	O2—C17—C18—C19	2.4 (2)
C4—C5—C6—C1	46.9 (2)	O3—C17—C18—C23	3.0 (2)
C4—C5—C6—C8	166.67 (14)	O2—C17—C18—	-175.06 (14)

		C23	
O1—C1—C6—C11	-100.62 (18)	C23—C18—C19—C20	1.1 (3)
C2—C1—C6—C11	80.98 (17)	C17—C18—C19—C20	-176.26 (15)
O1—C1—C6—C5	135.10 (17)	C18—C19—C20—C21	0.2 (3)
C2—C1—C6—C5	-43.30 (19)	C19—C20—C21—C22	-1.8 (3)
O1—C1—C6—C8	15.6 (2)	C19—C20—C21—N1	177.88 (16)
C2—C1—C6—C8	-162.75 (14)	O5—N1—C21—C22	-0.3 (3)
C11—C6—C8—C9	176.50 (16)	O4—N1—C21—C22	179.49 (18)
C5—C6—C8—C9	-59.8 (2)	O5—N1—C21—C20	-179.96 (18)
C1—C6—C8—C9	60.3 (2)	O4—N1—C21—C20	-0.2 (3)
C6—C8—C9—C10	119.3 (2)	C20—C21—C22—C23	1.9 (3)
C5—C6—C11—C16	-7.0 (2)	N1—C21—C22—C23	-177.72 (16)
C1—C6—C11—C16	-128.97 (17)	C21—C22—C23—C18	-0.6 (3)
C8—C6—C11—C16	113.57 (18)	C19—C18—C23—C22	-0.9 (3)
C5—C6—C11—C12	174.54 (15)	C17—C18—C23—C22	176.60 (15)
C1—C6—C11—C12	52.6 (2)		

Compound (+)-16

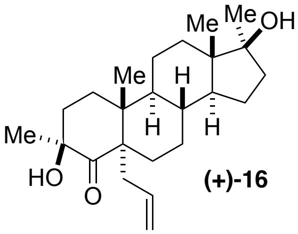

(+)-16

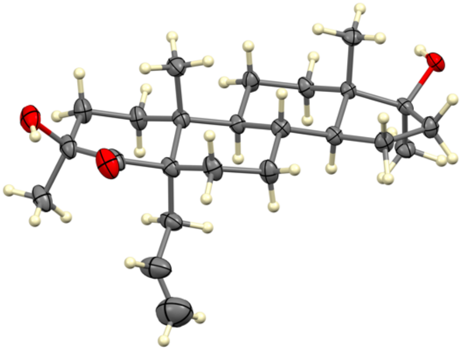

Sample Name: CCDC 2280311

Crystal Growth: Slow crystallization in DMSO-d_6.

Crystal data

$C_{24}H_{38}O_3$	D_x = 1.186 Mg m^{-3}
M_r = 374.54	Cu *K*α radiation, λ = 1.54184 Å
Trigonal, *R*3:*H*	Cell parameters from 8043 reflections
a = 29.0823 (10) Å	θ = 3.0–67.0°
c = 6.4447 (4) Å	μ = 0.59 mm^{-1}
V = 4720.5 (4) Å^3	*T* = 100 K
Z = 9	Needle, colourless
F(000) = 1854	0.30 × 0.04 × 0.03 mm

Data collection

Bruker Kappa APEX-II DUO CCD diffractometer	3849 independent reflections
Radiation source: IμS microfocus	3336 reflections with *I* > 2σ(*I*)
QUAZAR multilayer optics monochromator	R_{int} = 0.077
ϕ and ω scans	$θ_{max}$ = 68.5°, $θ_{min}$ = 3.0°
Absorption correction: multi-scan	*h* = -34 – 35

SADABS (Krause *et al.*, 2015)	
T_{min} = 0.885, T_{max} = 0.983	k = -34 – 34
38264 measured reflections	l = -7 – 7

Refinement

Refinement on F^2	Hydrogen site location: mixed
Least-squares matrix: full	H atoms treated by a mixture of independent and constrained refinement
$R[F^2 > 2\sigma(F^2)]$ = 0.040	$w = 1/[\sigma^2(F_o^2) + (0.0605P)^2 + 0.0158P]$ where $P = (F_o^2 + 2F_c^2)/3$
$wR(F^2)$ = 0.101	$(\Delta/\sigma)_{max}$ < 0.001
S = 1.08	$\Delta\rho_{max}$ = 0.14 e Å^{-3}
3849 reflections	$\Delta\rho_{min}$ = -0.19 e Å^{-3}
264 parameters	Absolute structure: Flack x determined using 1473 quotients [(I+)-(I-)]/[(I+)+(I-)] (Parsons, Flack and Wagner, Acta Cryst. B69 (2013) 249-259).
14 restraints	Absolute structure parameter: 0.18 (13)

Fractional atomic coordinates and isotropic or equivalent isotropic displacement parameters (Å^2) for (RST1285P2)

	x	y	z	U_{iso}*/U_{eq}	Occ. (<1)
O1	0.55604 (9)	0.53279 (11)	0.3768 (4)	0.0399 (6)	
H10H	0.5375 (19)	0.5054 (19)	0.460 (7)	0.060*	
O2	0.57201 (8)	0.47093 (10)	0.6231 (3)	0.0401 (6)	
O3	0.96354 (7)	0.63127 (8)	0.7119 (3)	0.0224 (4)	
H30H	0.9745 (15)	0.6552 (14)	0.587 (6)	0.034*	
C1	0.69456 (12)	0.56865 (14)	0.2415 (5)	0.0283 (7)	
H1A	0.724055	0.604828	0.210616	0.034*	
H1B	0.695134	0.544257	0.135748	0.034*	
C2	0.64201 (13)	0.56826 (14)	0.2269 (5)	0.0336 (7)	

H2A	0.636011	0.573850	0.080141	0.040*	
H2B	0.645172	0.598682	0.307572	0.040*	
C3	0.59320 (12)	0.51758 (14)	0.3046 (5)	0.0303 (7)	
C4	0.60561 (12)	0.49181 (12)	0.4877 (4)	0.0280 (7)	
C5	0.65834 (11)	0.49260 (12)	0.4983 (5)	0.0251 (6)	
C6	0.66633 (12)	0.47508 (13)	0.7141 (5)	0.0312 (7)	
H6A	0.662800	0.497556	0.821058	0.037*	
H6B	0.638169	0.437888	0.738868	0.037*	
C7	0.72054 (12)	0.47917 (13)	0.7360 (5)	0.0303 (7)	
H7A	0.724720	0.469963	0.879971	0.036*	
H7B	0.722171	0.453080	0.642082	0.036*	
C8	0.76611 (12)	0.53438 (12)	0.6852 (4)	0.0229 (6)	
H8	0.767598	0.559330	0.795237	0.027*	
C9	0.75807 (11)	0.55456 (12)	0.4720 (4)	0.0208 (6)	
H9	0.757720	0.529511	0.364926	0.025*	
C10	0.70335 (11)	0.55165 (12)	0.4580 (4)	0.0224 (6)	
C11	0.80505 (11)	0.60941 (12)	0.4190 (5)	0.0259 (7)	
H11A	0.800075	0.619301	0.277191	0.031*	
H11B	0.805369	0.635884	0.516365	0.031*	
C12	0.85877 (12)	0.61164 (12)	0.4300 (4)	0.0237 (6)	
H12A	0.860603	0.588924	0.319698	0.028*	
H12B	0.887622	0.648539	0.405480	0.028*	
C13	0.86673 (11)	0.59284 (11)	0.6411 (4)	0.0182 (6)	
C14	0.81984 (11)	0.53654 (11)	0.6801 (4)	0.0211 (6)	
H14	0.818716	0.514185	0.560179	0.025*	
C15	0.83761	0.51758	0.8694 (5)	0.0258 (7)	

	(12)	(12)			
H15A	0.821244	0.478446	0.868956	0.031*	
H15B	0.827875	0.528618	0.999809	0.031*	
C16	0.89862 (12)	0.54476 (13)	0.8464 (4)	0.0251 (6)	
H16A	0.909364	0.517885	0.820797	0.030*	
H16B	0.916289	0.564429	0.974657	0.030*	
C17	0.91453 (11)	0.58341 (11)	0.6605 (4)	0.0203 (6)	
C18	0.87156 (12)	0.63222 (12)	0.8101 (4)	0.0251 (6)	
H18A	0.901181	0.667614	0.776989	0.038*	
H18B	0.878034	0.620703	0.944470	0.038*	
H18C	0.838547	0.633489	0.816804	0.038*	
C19	0.69973 (13)	0.58896 (14)	0.6190 (5)	0.0304 (7)	
H19A	0.720119	0.625715	0.569944	0.046*	
H19B	0.714265	0.585638	0.751699	0.046*	
H19C	0.662495	0.579217	0.637618	0.046*	
C20	0.92481 (12)	0.56028 (13)	0.4653 (5)	0.0261 (7)	
H20A	0.937589	0.586896	0.354443	0.039*	
H20B	0.891791	0.528848	0.421304	0.039*	
H20C	0.951671	0.550214	0.494815	0.039*	
C21	0.56592 (13)	0.47645 (14)	0.1320 (5)	0.0347 (8)	
H21A	0.535824	0.444362	0.190147	0.052*	
H21B	0.591332	0.467498	0.071101	0.052*	
H21C	0.553166	0.491183	0.024526	0.052*	
C22	0.65679 (12)	0.45284 (12)	0.3343 (5)	0.0318 (7)	
H22A	0.655683	0.466372	0.194482	0.038*	
H22B	0.690119	0.451596	0.344376	0.038*	
C23	0.61085 (16)	0.39748 (15)	0.3547 (7)	0.0511 (10)	
H23	0.577958	0.392424	0.405369	0.061*	0.38 (2)

H23A	0.593944	0.389332	0.486457	0.061*	0.62 (2)
C24	0.6144 (9)	0.3582 (6)	0.308 (4)	0.077 (6)	0.38 (2)
H24A	0.647008	0.362576	0.256738	0.093*	0.38 (2)
H24B	0.584510	0.323583	0.322402	0.093*	0.38 (2)
C24A	0.5914 (4)	0.3608 (3)	0.2285 (12)	0.047 (3)	0.62 (2)
H24C	0.606056	0.365769	0.093030	0.056*	0.62 (2)
H24D	0.561850	0.327631	0.266539	0.056*	0.62 (2)

Atomic displacement parameters (Å^2) for (RST1285P2)

	U^{11}	U^{22}	U^{33}	U^{12}	U^{13}	U^{23}
O1	0.0285 (13)	0.0572 (17)	0.0411 (14)	0.0266 (12)	0.0000 (10)	-0.0068 (11)
O2	0.0244 (12)	0.0621 (17)	0.0253 (11)	0.0154 (12)	0.0055 (10)	0.0000 (11)
O3	0.0191 (10)	0.0238 (11)	0.0184 (9)	0.0064 (9)	-0.0034 (8)	-0.0006 (8)
C1	0.0228 (16)	0.0353 (17)	0.0246 (15)	0.0129 (14)	-0.0025 (12)	0.0038 (13)
C2	0.0238 (17)	0.0415 (19)	0.0347 (18)	0.0157 (15)	-0.0044 (14)	0.0013 (15)
C3	0.0218 (15)	0.046 (2)	0.0276 (16)	0.0198 (15)	-0.0021 (12)	-0.0063 (14)
C4	0.0232 (16)	0.0363 (18)	0.0195 (14)	0.0112 (14)	-0.0021 (12)	-0.0069 (13)
C5	0.0195 (15)	0.0293 (16)	0.0227 (14)	0.0094 (13)	0.0001 (11)	-0.0029 (12)
C6	0.0204 (15)	0.0357 (18)	0.0290 (16)	0.0078 (14)	0.0021 (12)	0.0049 (14)
C7	0.0216 (16)	0.0317 (17)	0.0317 (17)	0.0088 (14)	0.0012 (13)	0.0088 (14)
C8	0.0227 (15)	0.0236 (15)	0.0201 (13)	0.0101 (12)	0.0013 (11)	0.0011 (11)
C9	0.0173 (14)	0.0243 (15)	0.0193 (14)	0.0094 (12)	0.0003 (11)	0.0004 (11)
C10	0.0193 (14)	0.0263 (15)	0.0208 (13)	0.0109 (13)	-0.0009 (11)	-0.0021 (12)

C11	0.0224 (15)	0.0264 (16)	0.0268 (15)	0.0105 (13)	-0.0025 (12)	0.0061 (13)
C12	0.0201 (15)	0.0266 (15)	0.0186 (13)	0.0074 (12)	-0.0013 (11)	0.0030 (12)
C13	0.0182 (14)	0.0201 (14)	0.0154 (13)	0.0088 (12)	0.0001 (10)	0.0007 (11)
C14	0.0214 (15)	0.0233 (15)	0.0165 (13)	0.0096 (13)	0.0006 (11)	0.0011 (11)
C15	0.0256 (16)	0.0249 (16)	0.0235 (14)	0.0101 (13)	0.0007 (12)	0.0065 (12)
C16	0.0246 (16)	0.0284 (15)	0.0223 (14)	0.0133 (13)	-0.0014 (12)	0.0046 (12)
C17	0.0159 (14)	0.0219 (15)	0.0184 (13)	0.0061 (12)	-0.0024 (11)	-0.0048 (11)
C18	0.0280 (16)	0.0271 (16)	0.0213 (14)	0.0145 (13)	-0.0015 (12)	-0.0050 (12)
C19	0.0273 (17)	0.0343 (17)	0.0320 (17)	0.0171 (15)	-0.0031 (13)	-0.0074 (13)
C20	0.0259 (15)	0.0314 (17)	0.0215 (14)	0.0146 (14)	0.0003 (12)	-0.0052 (12)
C21	0.0262 (17)	0.052 (2)	0.0219 (15)	0.0166 (16)	-0.0019 (13)	-0.0055 (15)
C22	0.0227 (16)	0.0289 (17)	0.0374 (18)	0.0082 (14)	0.0004 (14)	-0.0098 (14)
C23	0.041 (2)	0.036 (2)	0.063 (3)	0.0100 (18)	0.009 (2)	-0.0128 (19)
C24	0.070 (8)	0.055 (7)	0.096 (9)	0.022 (6)	0.008 (7)	-0.001 (6)
C24A	0.049 (4)	0.031 (3)	0.040 (3)	0.005 (3)	-0.009 (3)	-0.002 (3)

Geometric parameters (Å, º) for (RST1285P2)

O1—C3	1.435 (4)	C12—H12B	0.9900
O1—H10H	0.89 (5)	C13—C18	1.535 (4)
O2—C4	1.221 (4)	C13—C14	1.540 (4)
O3—C17	1.447 (3)	C13—C17	1.550 (4)
O3—H30H	1.01 (4)	C14—C15	1.531 (4)
C1—C2	1.526 (4)	C14—H14	1.0000

C1—C10	1.543 (4)	C15—C16	1.547 (4)
C1—H1A	0.9900	C15—H15A	0.9900
C1—H1B	0.9900	C15—H15B	0.9900
C2—C3	1.532 (5)	C16—C17	1.547 (4)
C2—H2A	0.9900	C16—H16A	0.9900
C2—H2B	0.9900	C16—H16B	0.9900
C3—C21	1.532 (4)	C17—C20	1.525 (4)
C3—C4	1.535 (4)	C18—H18A	0.9800
C4—C5	1.524 (4)	C18—H18B	0.9800
C5—C6	1.539 (4)	C18—H18C	0.9800
C5—C22	1.550 (4)	C19—H19A	0.9800
C5—C10	1.576 (4)	C19—H19B	0.9800
C6—C7	1.527 (4)	C19—H19C	0.9800
C6—H6A	0.9900	C20—H20A	0.9800
C6—H6B	0.9900	C20—H20B	0.9800
C7—C8	1.521 (4)	C20—H20C	0.9800
C7—H7A	0.9900	C21—H21A	0.9800
C7—H7B	0.9900	C21—H21B	0.9800
C8—C14	1.532 (4)	C21—H21C	0.9800
C8—C9	1.557 (4)	C22—C23	1.498 (5)
C8—H8	1.0000	C22—H22A	0.9900
C9—C11	1.533 (4)	C22—H22B	0.9900
C9—C10	1.553 (4)	C23—C24A	1.230 (8)
C9—H9	1.0000	C23—C24	1.235 (15)
C10—C19	1.543 (4)	C23—H23	0.9500
C11—C12	1.532 (4)	C23—H23A	0.9500
C11—H11A	0.9900	C24—H24A	0.9500
C11—H11B	0.9900	C24—H24B	0.9500
C12—C13	1.526 (4)	C24A—H24C	0.9500
C12—H12A	0.9900	C24A—H24D	0.9500
C3—O1—H10H	98 (3)	C12—C13—C14	108.3 (2)
C17—O3—H30H	107 (2)	C18—C13—C14	112.3 (2)

C2—C1—C10	112.3 (3)	C12—C13—C17	116.4 (2)
C2—C1—H1A	109.1	C18—C13—C17	108.6 (2)
C10—C1—H1A	109.1	C14—C13—C17	101.2 (2)
C2—C1—H1B	109.1	C15—C14—C8	119.3 (2)
C10—C1—H1B	109.1	C15—C14—C13	103.5 (2)
H1A—C1—H1B	107.9	C8—C14—C13	113.0 (2)
C1—C2—C3	115.5 (3)	C15—C14—H14	106.8
C1—C2—H2A	108.4	C8—C14—H14	106.8
C3—C2—H2A	108.4	C13—C14—H14	106.8
C1—C2—H2B	108.4	C14—C15—C16	104.3 (2)
C3—C2—H2B	108.4	C14—C15—H15A	110.9
H2A—C2—H2B	107.5	C16—C15—H15A	110.9
O1—C3—C2	106.7 (3)	C14—C15—H15B	110.9
O1—C3—C21	107.5 (2)	C16—C15—H15B	110.9
C2—C3—C21	112.5 (3)	H15A—C15—H15B	108.9
O1—C3—C4	107.4 (3)	C15—C16—C17	107.2 (2)
C2—C3—C4	113.0 (2)	C15—C16—H16A	110.3
C21—C3—C4	109.5 (3)	C17—C16—H16A	110.3
O2—C4—C5	121.7 (3)	C15—C16—H16B	110.3
O2—C4—C3	117.9 (3)	C17—C16—H16B	110.3
C5—C4—C3	120.5 (3)	H16A—C16—H16B	108.5
C4—C5—C6	110.7 (2)	O3—C17—C20	107.3 (2)
C4—C5—C22	107.7 (2)	O3—C17—C16	108.3 (2)
C6—C5—C22	108.8 (3)	C20—C17—C16	111.8 (2)
C4—C5—C10	106.9 (2)	O3—C17—C13	112.8 (2)
C6—C5—C10	109.2 (2)	C20—C17—C13	113.7 (2)
C22—C5—C10	113.5 (2)	C16—C17—C13	102.8 (2)
C7—C6—C5	112.2 (2)	C13—C18—H18A	109.5
C7—C6—H6A	109.2	C13—C18—H18B	109.5
C5—C6—H6A	109.2	H18A—C18—H18B	109.5
C7—C6—H6B	109.2	C13—C18—H18C	109.5
C5—C6—H6B	109.2	H18A—C18—H18C	109.5
H6A—C6—H6B	107.9	H18B—C18—H18C	109.5

C8—C7—C6	112.5 (3)	C10—C19—H19A	109.5
C8—C7—H7A	109.1	C10—C19—H19B	109.5
C6—C7—H7A	109.1	H19A—C19—H19B	109.5
C8—C7—H7B	109.1	C10—C19—H19C	109.5
C6—C7—H7B	109.1	H19A—C19—H19C	109.5
H7A—C7—H7B	107.8	H19B—C19—H19C	109.5
C7—C8—C14	112.4 (2)	C17—C20—H20A	109.5
C7—C8—C9	111.8 (2)	C17—C20—H20B	109.5
C14—C8—C9	107.9 (2)	H20A—C20—H20B	109.5
C7—C8—H8	108.2	C17—C20—H20C	109.5
C14—C8—H8	108.2	H20A—C20—H20C	109.5
C9—C8—H8	108.2	H20B—C20—H20C	109.5
C11—C9—C10	113.6 (2)	C3—C21—H21A	109.5
C11—C9—C8	111.4 (2)	C3—C21—H21B	109.5
C10—C9—C8	112.0 (2)	H21A—C21—H21B	109.5
C11—C9—H9	106.4	C3—C21—H21C	109.5
C10—C9—H9	106.4	H21A—C21—H21C	109.5
C8—C9—H9	106.4	H21B—C21—H21C	109.5
C19—C10—C1	107.6 (2)	C23—C22—C5	114.8 (3)
C19—C10—C9	110.5 (2)	C23—C22—H22A	108.6
C1—C10—C9	111.4 (2)	C5—C22—H22A	108.6
C19—C10—C5	110.1 (2)	C23—C22—H22B	108.6
C1—C10—C5	108.5 (2)	C5—C22—H22B	108.6
C9—C10—C5	108.7 (2)	H22A—C22—H22B	107.5
C12—C11—C9	113.2 (2)	C24A—C23—C22	130.5 (5)
C12—C11—H11A	108.9	C24—C23—C22	122.0 (9)
C9—C11—H11A	108.9	C24—C23—H23	119.0
C12—C11—H11B	108.9	C22—C23—H23	119.0
C9—C11—H11B	108.9	C24A—C23—H23A	114.8
H11A—C11—H11B	107.8	C22—C23—H23A	114.8
C13—C12—C11	111.1 (2)	C23—C24—H24A	120.0
C13—C12—H12A	109.4	C23—C24—H24B	120.0

C11—C12—H12A	109.4	H24A—C24—H24B	120.0
C13—C12—H12B	109.4	C23—C24A—H24C	120.0
C11—C12—H12B	109.4	C23—C24A—H24D	120.0
H12A—C12—H12B	108.0	H24C—C24A—H24D	120.0
C12—C13—C18	109.8 (2)		
C10—C1—C2—C3	-49.3 (4)	C22—C5—C10—C1	58.7 (3)
C1—C2—C3—O1	151.5 (3)	C4—C5—C10—C9	178.8 (2)
C1—C2—C3—C21	-90.8 (3)	C6—C5—C10—C9	59.0 (3)
C1—C2—C3—C4	33.8 (4)	C22—C5—C10—C9	-62.6 (3)
O1—C3—C4—O2	26.3 (4)	C10—C9—C11—C12	-179.1 (2)
C2—C3—C4—O2	143.7 (3)	C8—C9—C11—C12	53.3 (3)
C21—C3—C4—O2	-90.1 (3)	C9—C11—C12—C13	-54.5 (3)
O1—C3—C4—C5	-153.7 (3)	C11—C12—C13—C18	-66.8 (3)
C2—C3—C4—C5	-36.3 (4)	C11—C12—C13—C14	56.2 (3)
C21—C3—C4—C5	89.9 (3)	C11—C12—C13—C17	169.3 (2)
O2—C4—C5—C6	-11.7 (4)	C7—C8—C14—C15	-54.8 (4)
C3—C4—C5—C6	168.2 (3)	C9—C8—C14—C15	-178.6 (2)
O2—C4—C5—C22	107.1 (3)	C7—C8—C14—C13	-176.8 (2)
C3—C4—C5—C22	-72.9 (3)	C9—C8—C14—C13	59.5 (3)
O2—C4—C5—C10	-130.5 (3)	C12—C13—C14—C15	168.5 (2)
C3—C4—C5—C10	49.4 (3)	C18—C13—C14—C15	-70.1 (3)
C4—C5—C6—C7	-175.9 (3)	C17—C13—C14—C15	45.6 (3)
C22—C5—C6—	66.0 (3)	C12—C13—C14—C8	-61.1 (3)

C7			
C10—C5—C6—C7	-58.5 (3)	C18—C13—C14—C8	60.3 (3)
C5—C6—C7—C8	54.8 (4)	C17—C13—C14—C8	176.0 (2)
C6—C7—C8—C14	-172.7 (3)	C8—C14—C15—C16	-158.8 (3)
C6—C7—C8—C9	-51.1 (3)	C13—C14—C15—C16	-32.2 (3)
C7—C8—C9—C11	-178.1 (3)	C14—C15—C16—C17	6.6 (3)
C14—C8—C9—C11	-54.0 (3)	C15—C16—C17—O3	140.8 (2)
C7—C8—C9—C10	53.5 (3)	C15—C16—C17—C20	-101.2 (3)
C14—C8—C9—C10	177.6 (2)	C15—C16—C17—C13	21.2 (3)
C2—C1—C10—C19	-56.4 (3)	C12—C13—C17—O3	85.8 (3)
C2—C1—C10—C9	-177.7 (3)	C18—C13—C17—O3	-38.7 (3)
C2—C1—C10—C5	62.8 (3)	C14—C13—C17—O3	-157.0 (2)
C11—C9—C10—C19	-63.5 (3)	C12—C13—C17—C20	-36.6 (3)
C8—C9—C10—C19	63.8 (3)	C18—C13—C17—C20	-161.1 (2)
C11—C9—C10—C1	56.1 (3)	C14—C13—C17—C20	80.5 (3)
C8—C9—C10—C1	-176.7 (2)	C12—C13—C17—C16	-157.7 (2)
C11—C9—C10—C5	175.6 (2)	C18—C13—C17—C16	77.8 (3)
C8—C9—C10—C5	-57.2 (3)	C14—C13—C17—C16	-40.6 (3)
C4—C5—C10—C19	57.6 (3)	C4—C5—C22—C23	-55.5 (4)
C6—C5—C10—C19	-62.2 (3)	C6—C5—C22—C23	64.5 (4)

C22—C5—C10—C19	176.2 (3)	C10—C5—C22—C23	-173.6 (3)
C4—C5—C10—C1	-59.9 (3)	C5—C22—C23—C24A	161.2 (8)
C6—C5—C10—C1	-179.7 (2)	C5—C22—C23—C24	-147.6 (15)

Hydrogen-bond geometry (Å, º) for (RST1285P2)

D—H···*A*	*D*—H	H···*A*	*D*···*A*	*D*—H···*A*
O1—H10*H*···O2	0.89 (5)	2.03 (5)	2.609 (4)	122 (4)
O3—H30*H*···O3^{i}	1.01 (4)	1.81 (4)	2.809 (2)	173 (3)

Symmetry code: (i) -*x*+*y*+4/3, -*x*+5/3, *z*-1/3.

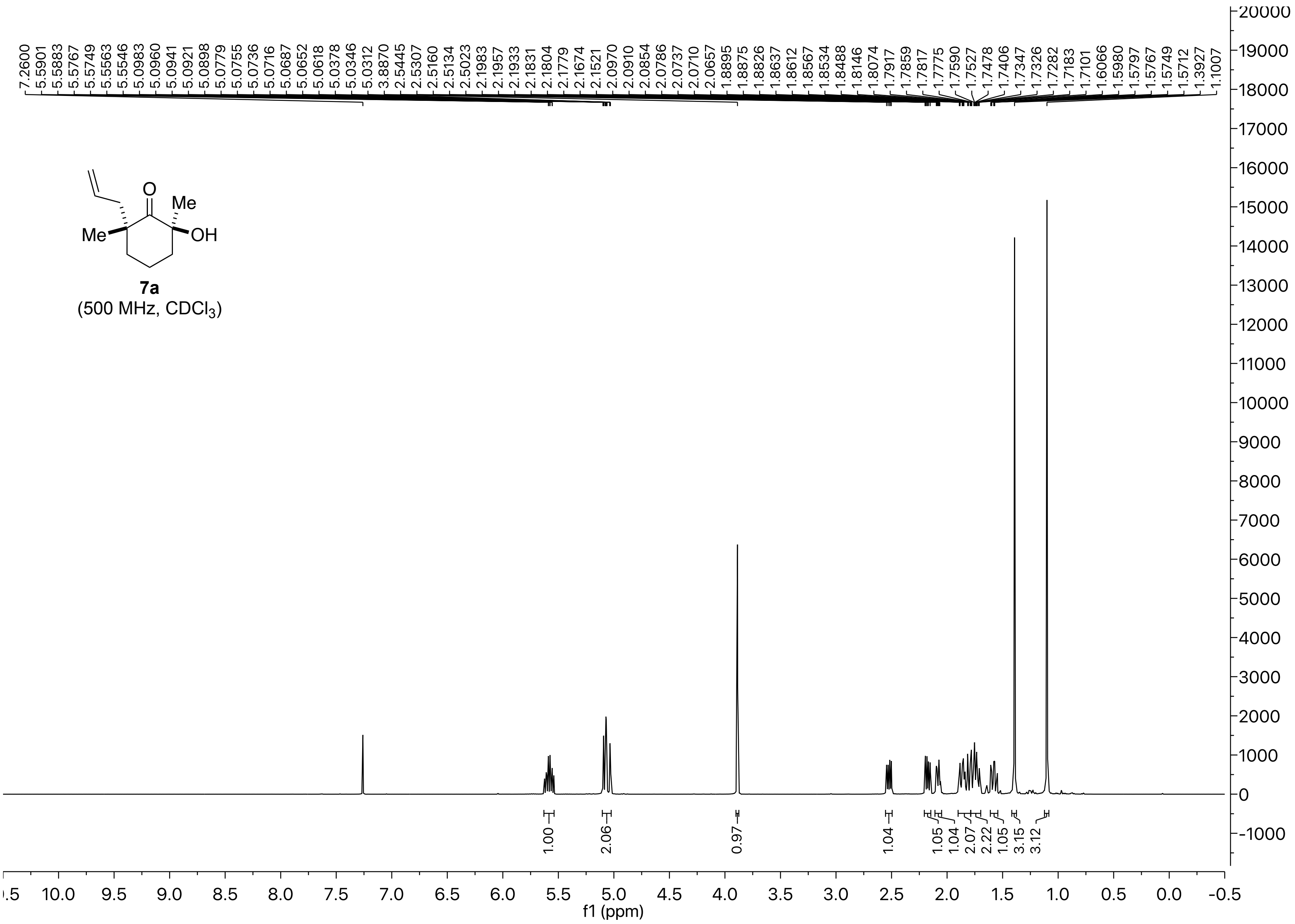
7a
(500 MHz, CDCl3)
f1 (ppm)

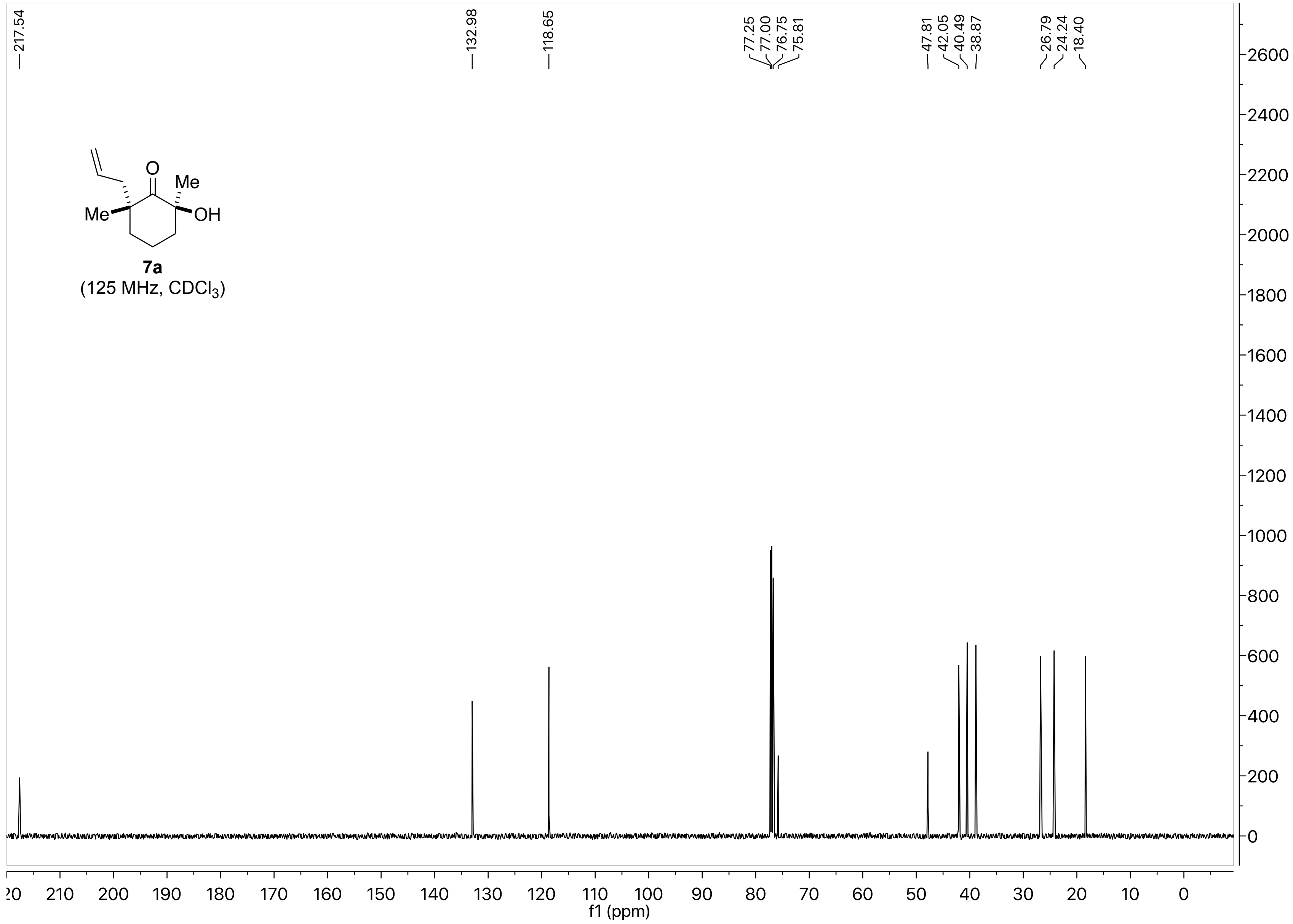
217.54
132.98
118.65
77.25
77.00
76.75
75.81
47.81
42.05
40.49
38.87
26.79
24.24
18.40
O
Me
Me
OH
7a
(125 MHz, CDCl3)
f1 (ppm)

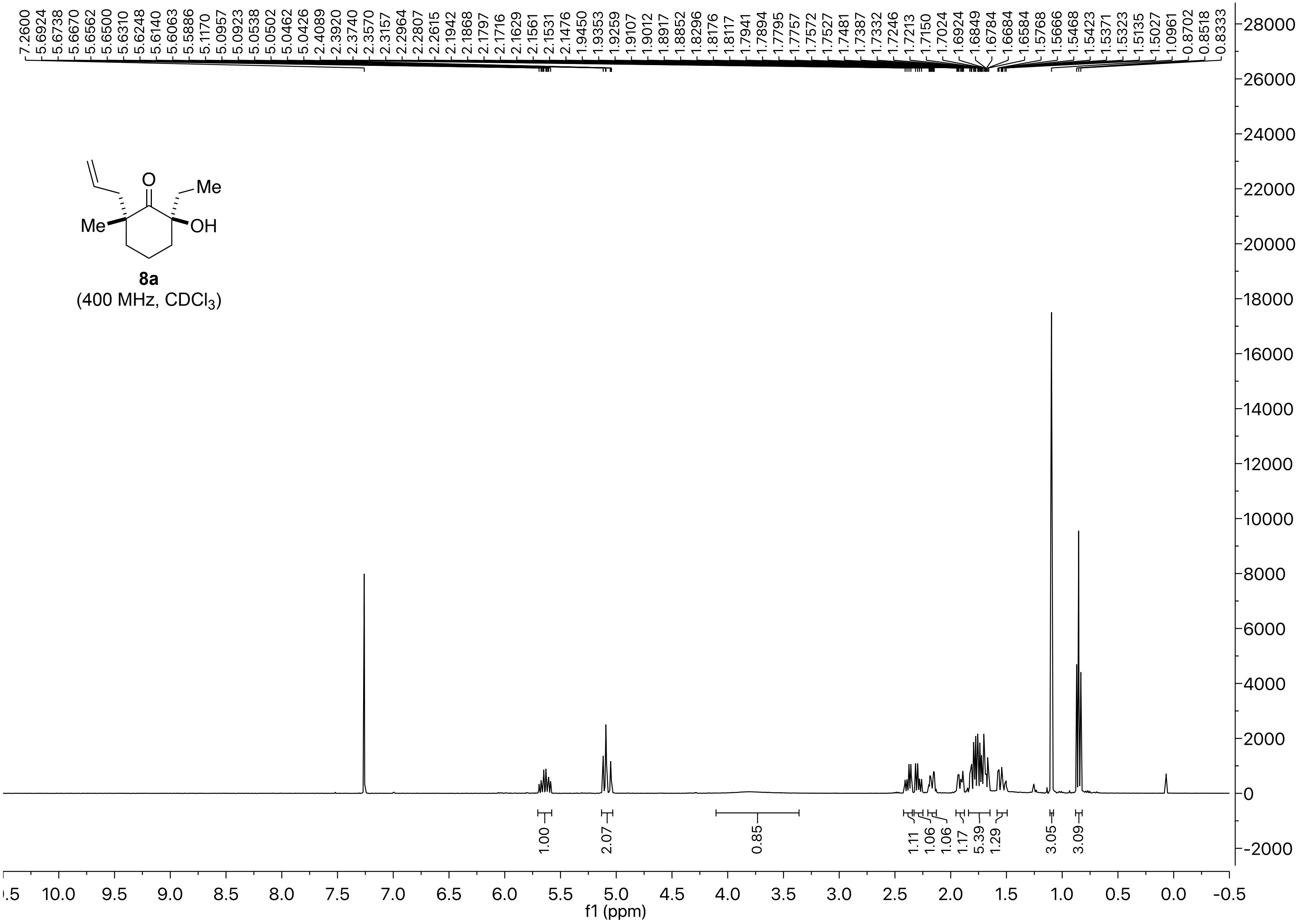
O
Me
Me
OH
8a
(400 MHz, CDCl3)
f1 (ppm)
1.00
2.07
0.85
1.11
1.06
1.06
1.17
5.39
1.29
3.05
3.09

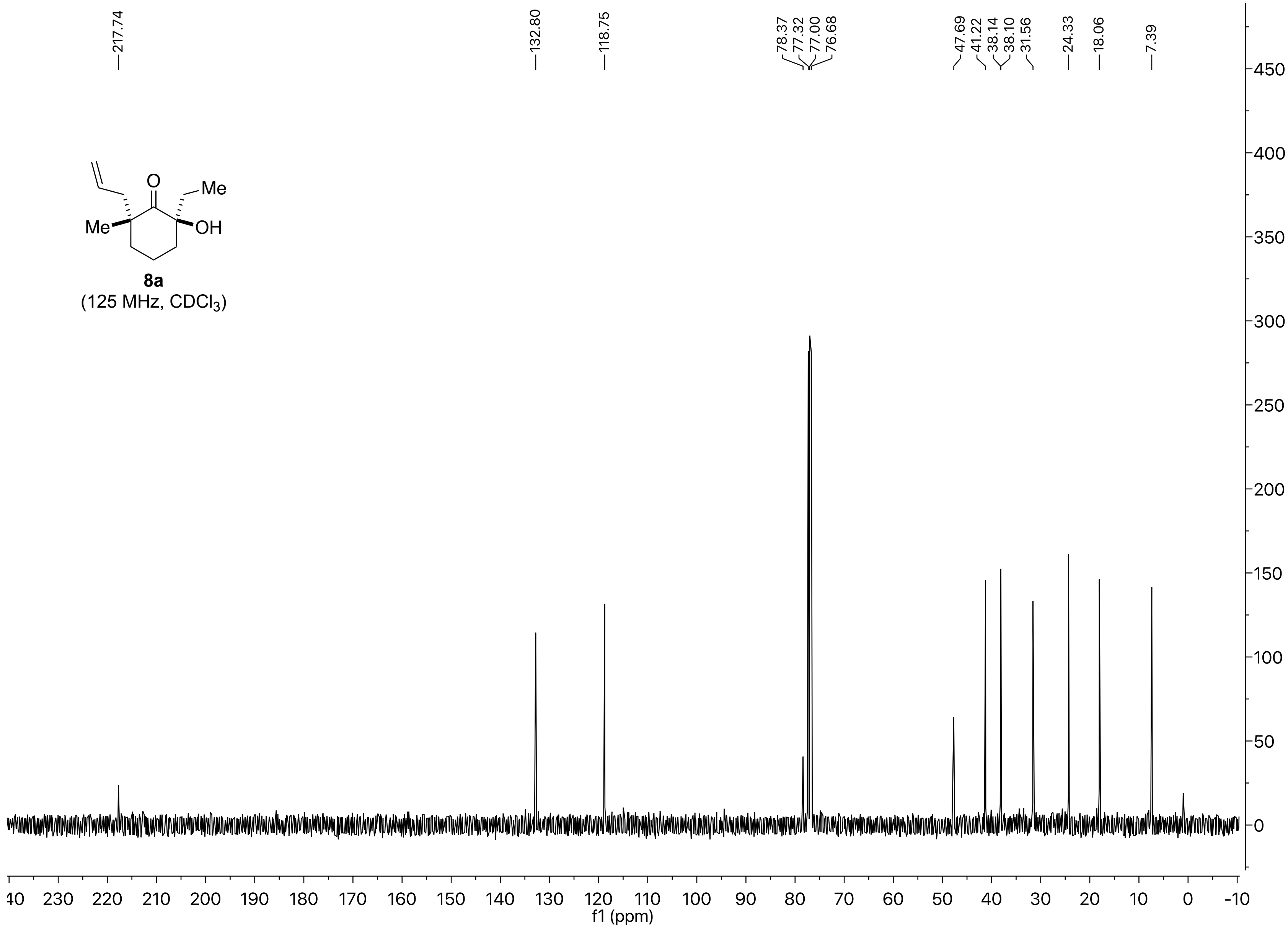
217.74
132.80
118.75
78.37
77.32
77.00
76.68
47.69
41.22
38.14
38.10
31.56
24.33
18.06
7.39
O
Me
Me
OH
8a
(125 MHz, CDCl3)
450
400
350
300
250
200
150
100
50
0
40 230 220 210 200 190 180 170 160 150 140 130 120 110 100 90 80 70 60 50 40 30 20 10 0 -10
f1 (ppm)

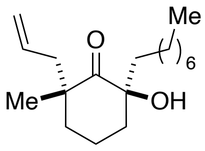

8b
(500 MHz, $CDCl_3$)

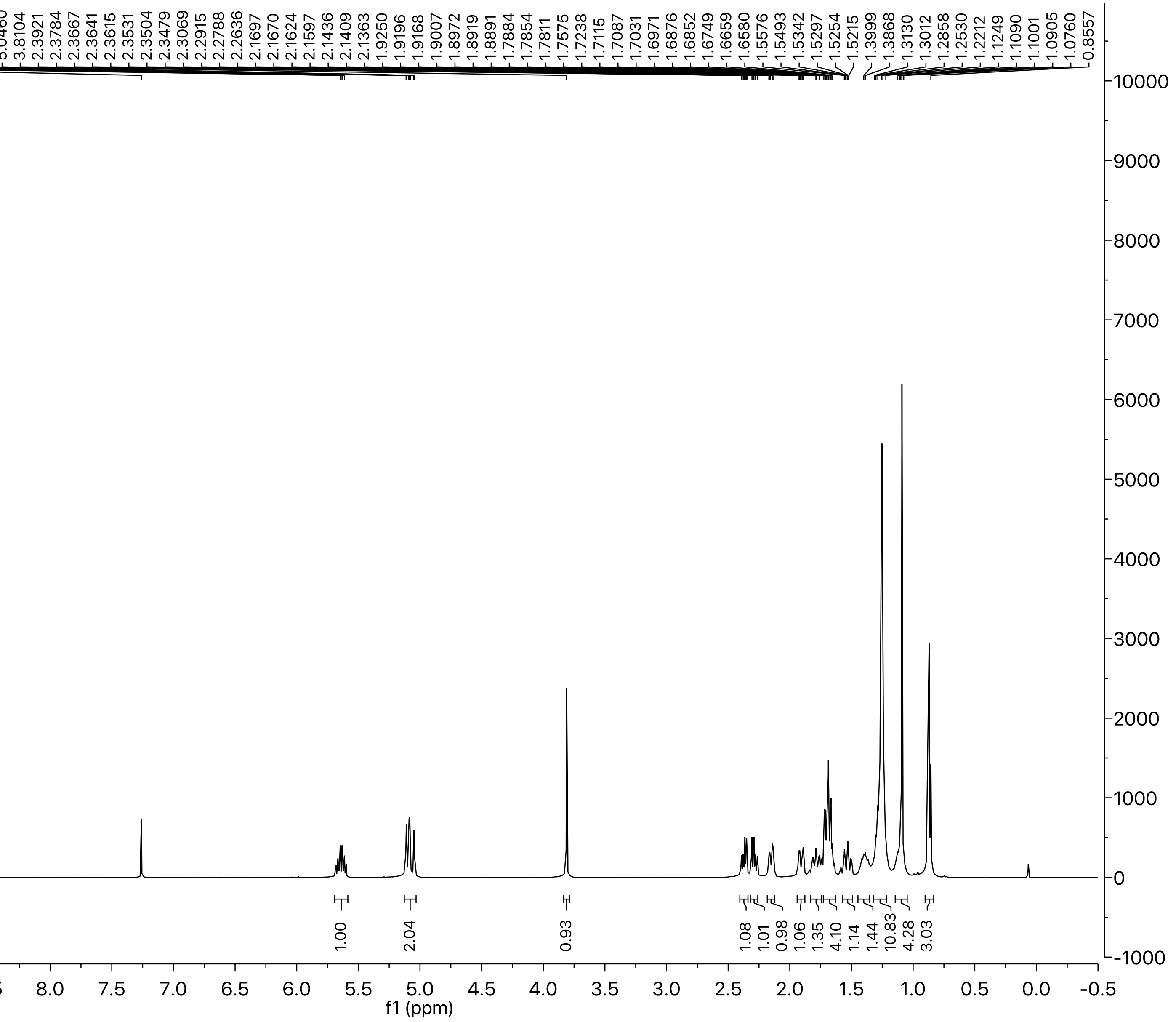

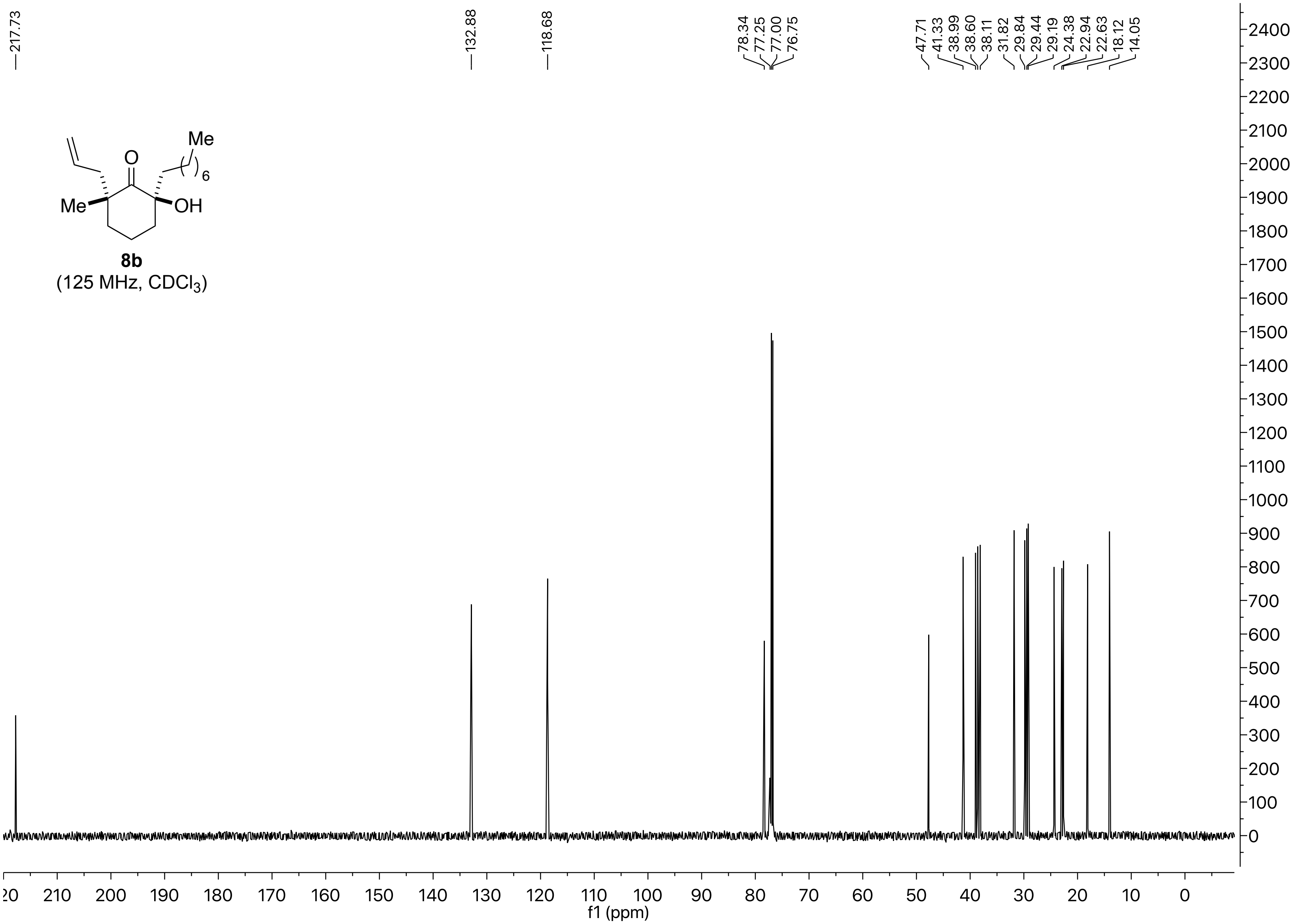
217.73
132.88
118.68
78.34
77.25
77.00
76.75
47.71
41.33
38.99
38.60
38.11
31.82
29.84
29.44
29.19
24.38
22.94
22.63
18.12
14.05
Me
O
6
Me
OH
8b
(125 MHz, CDCl3)
f1 (ppm)

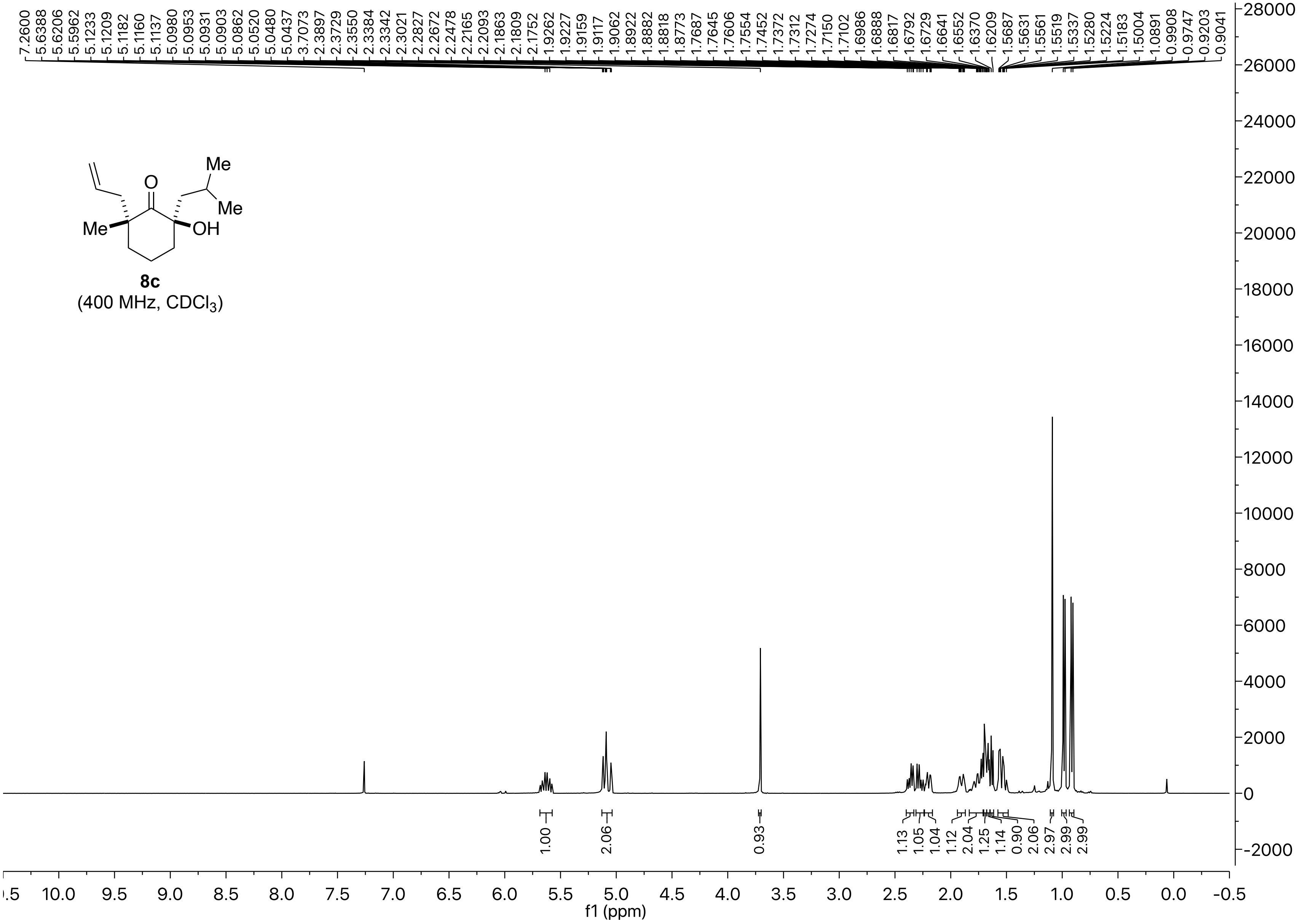
Me
O
Me
Me
OH
8c
(400 MHz, CDCl3)
f1 (ppm)

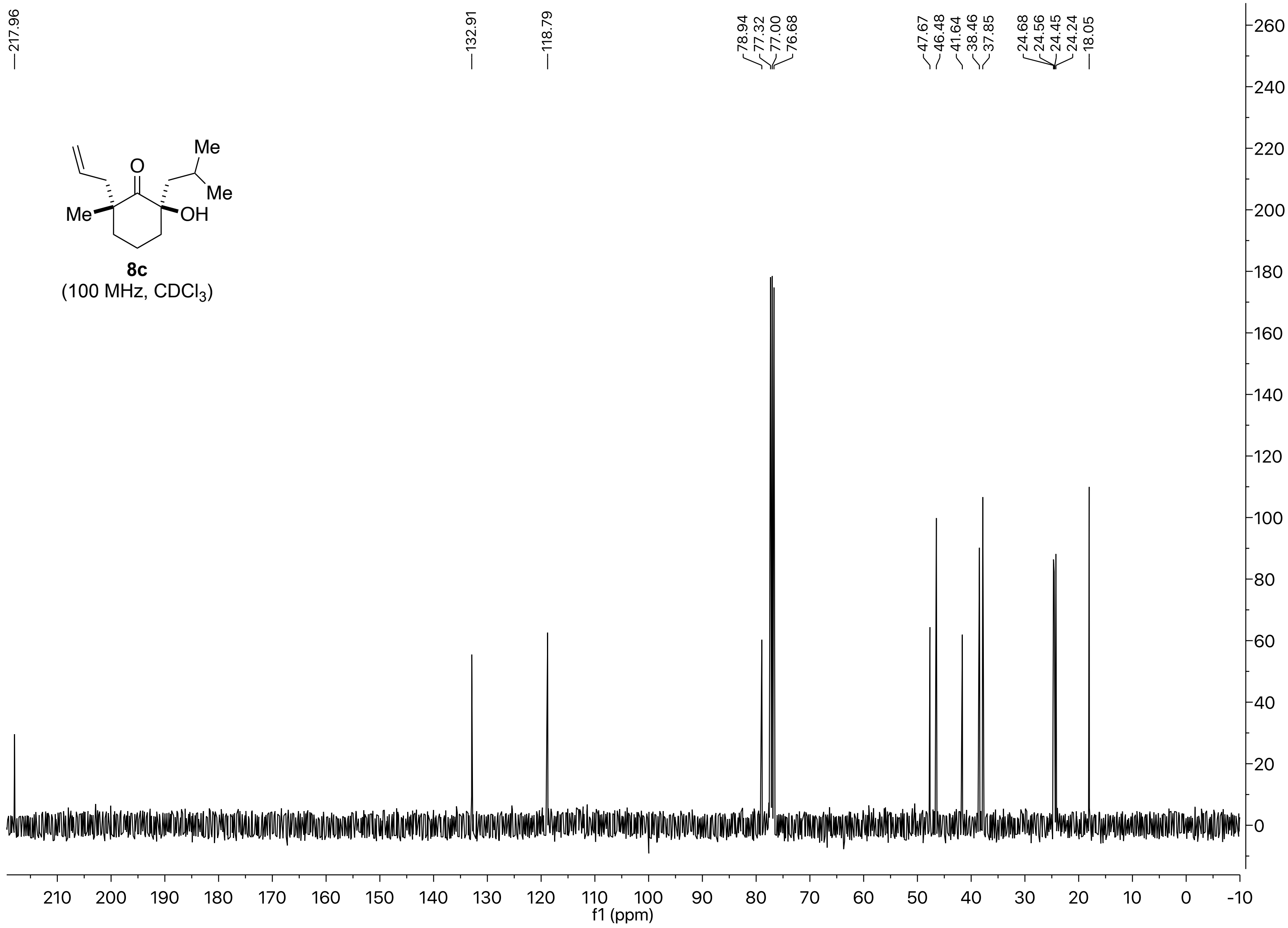

217.96
132.91
118.79
78.94
77.32
77.00
76.68
47.67
46.48
41.64
38.46
37.85
24.68
24.56
24.45
24.24
18.05
Me
O
Me
Me
OH
8c
(100 MHz, $CDCl_3$)
f1 (ppm)

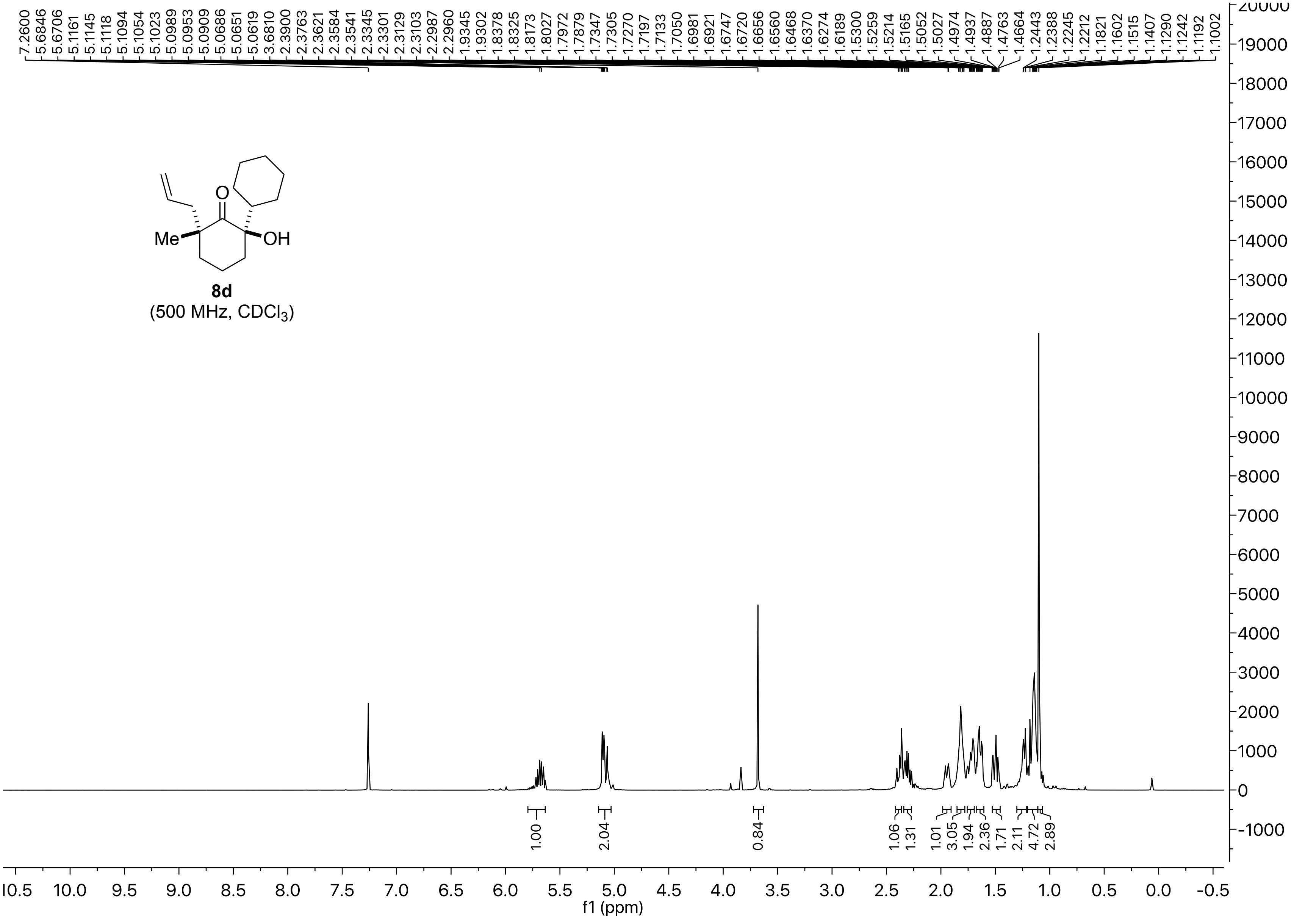

8d
(500 MHz, $CDCl_3$)

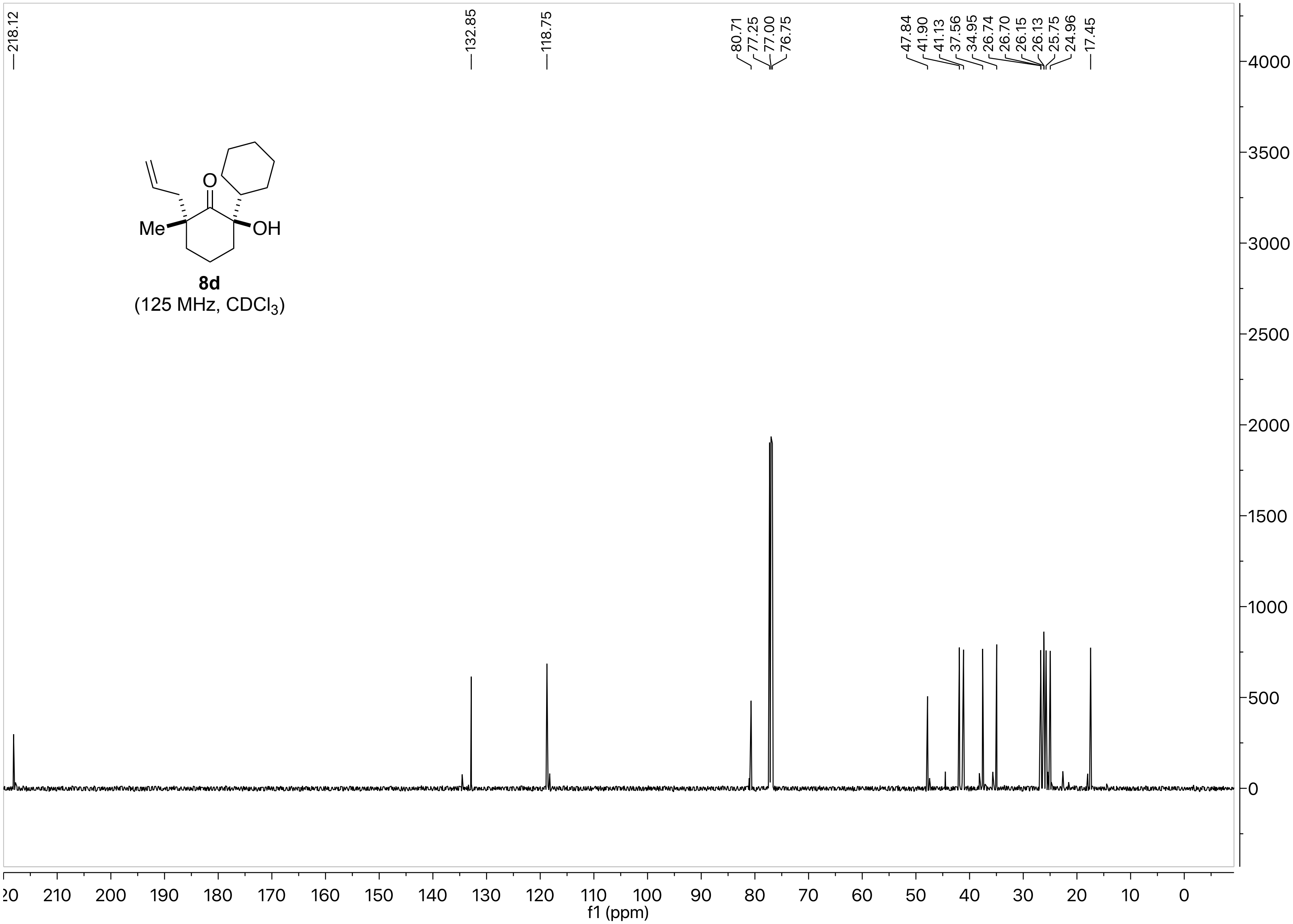

218.12
132.85
118.75
80.71
77.25
77.00
76.75
47.84
41.90
41.13
37.56
34.95
26.74
26.70
26.15
26.13
25.75
24.96
17.45
Me
O
OH
8d
(125 MHz, CDCl3)
f1 (ppm)

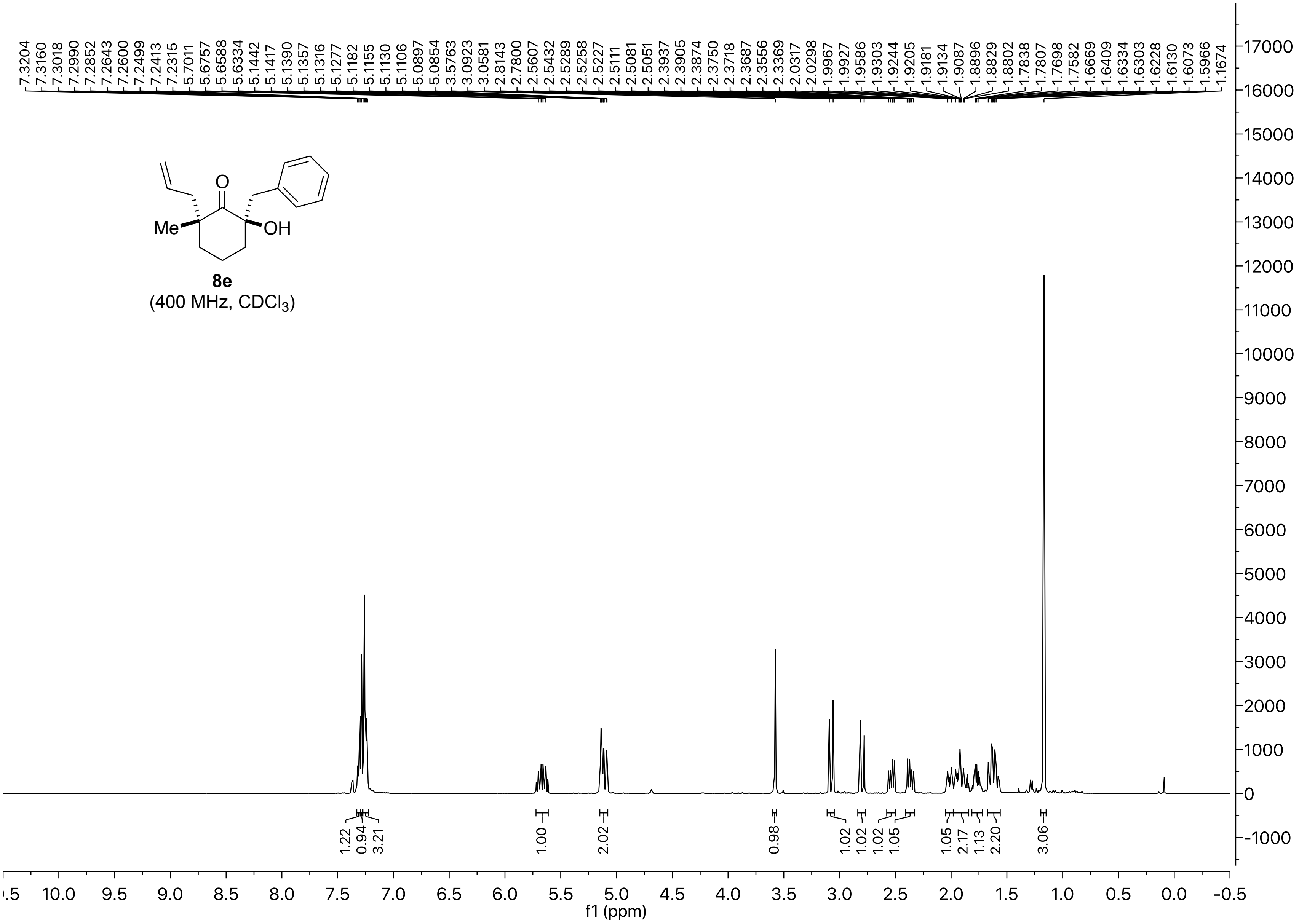
8e
(400 MHz, CDCl3)
f1 (ppm)

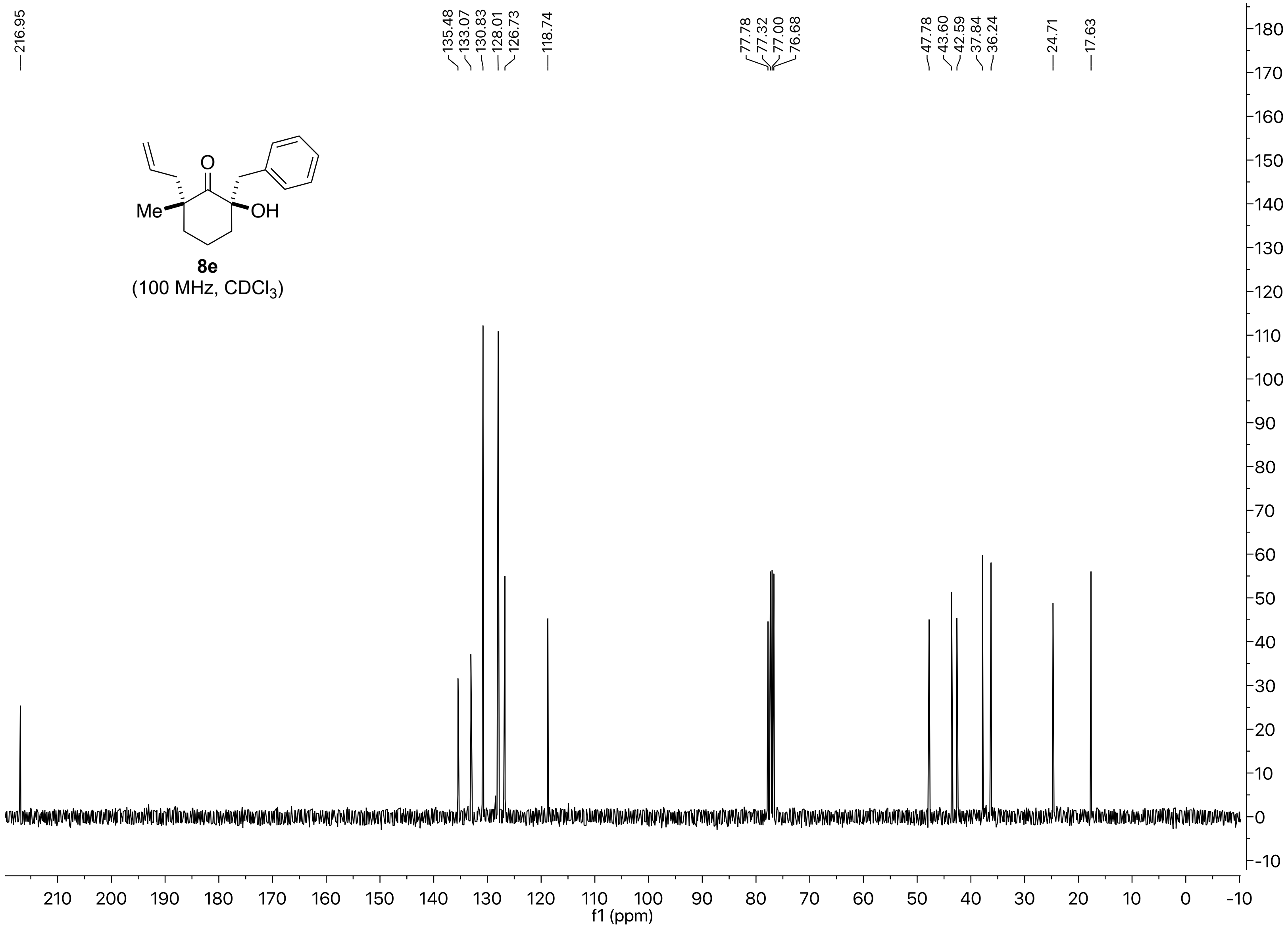

216.95
135.48
133.07
130.83
128.01
126.73
118.74
77.78
77.32
77.00
76.68
47.78
43.60
42.59
37.84
36.24
24.71
17.63
Me
O
OH
8e
(100 MHz, CDCl3)
f1 (ppm)

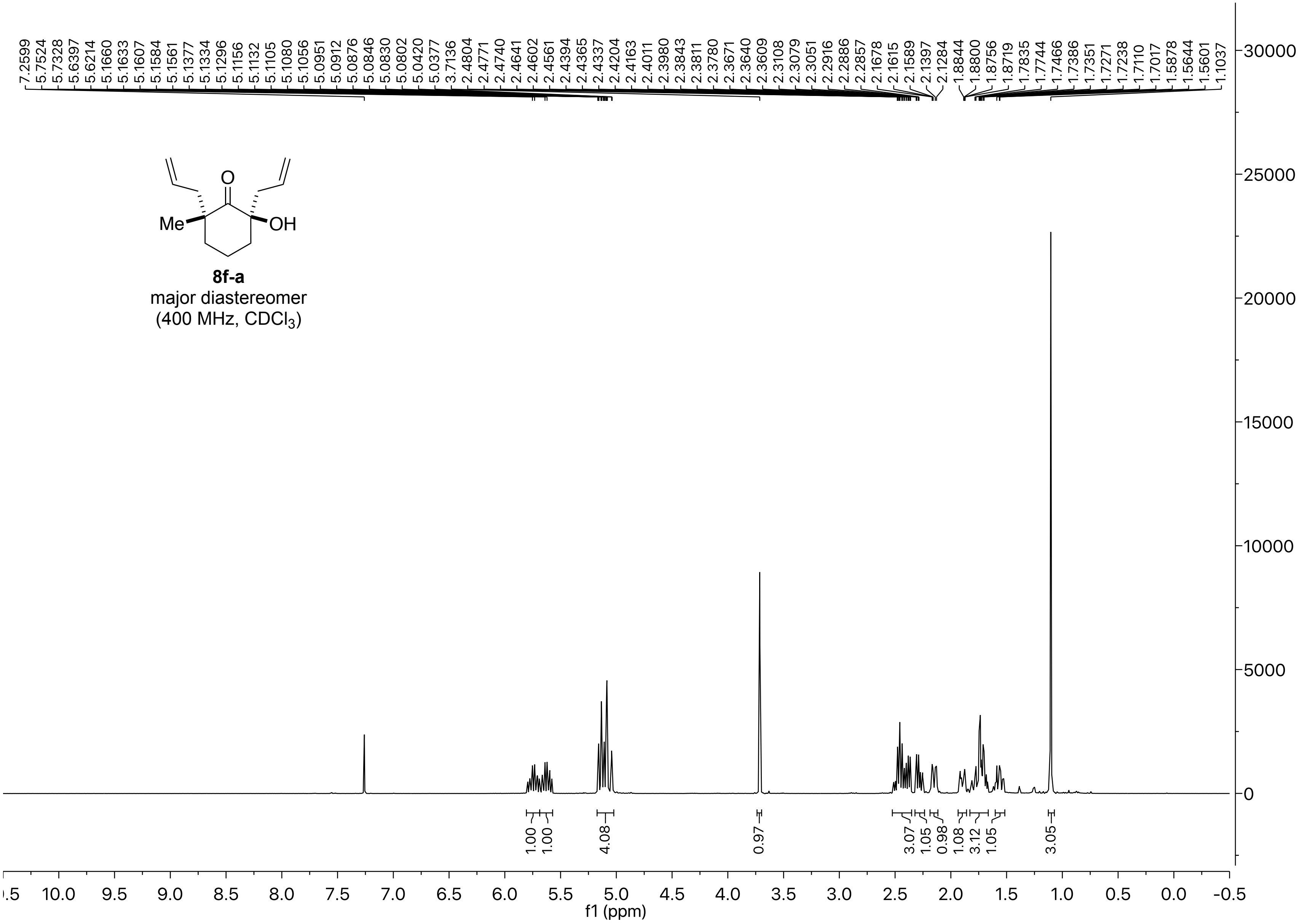
8f-a
major diastereomer
(400 MHz, CDCl3)
f1 (ppm)

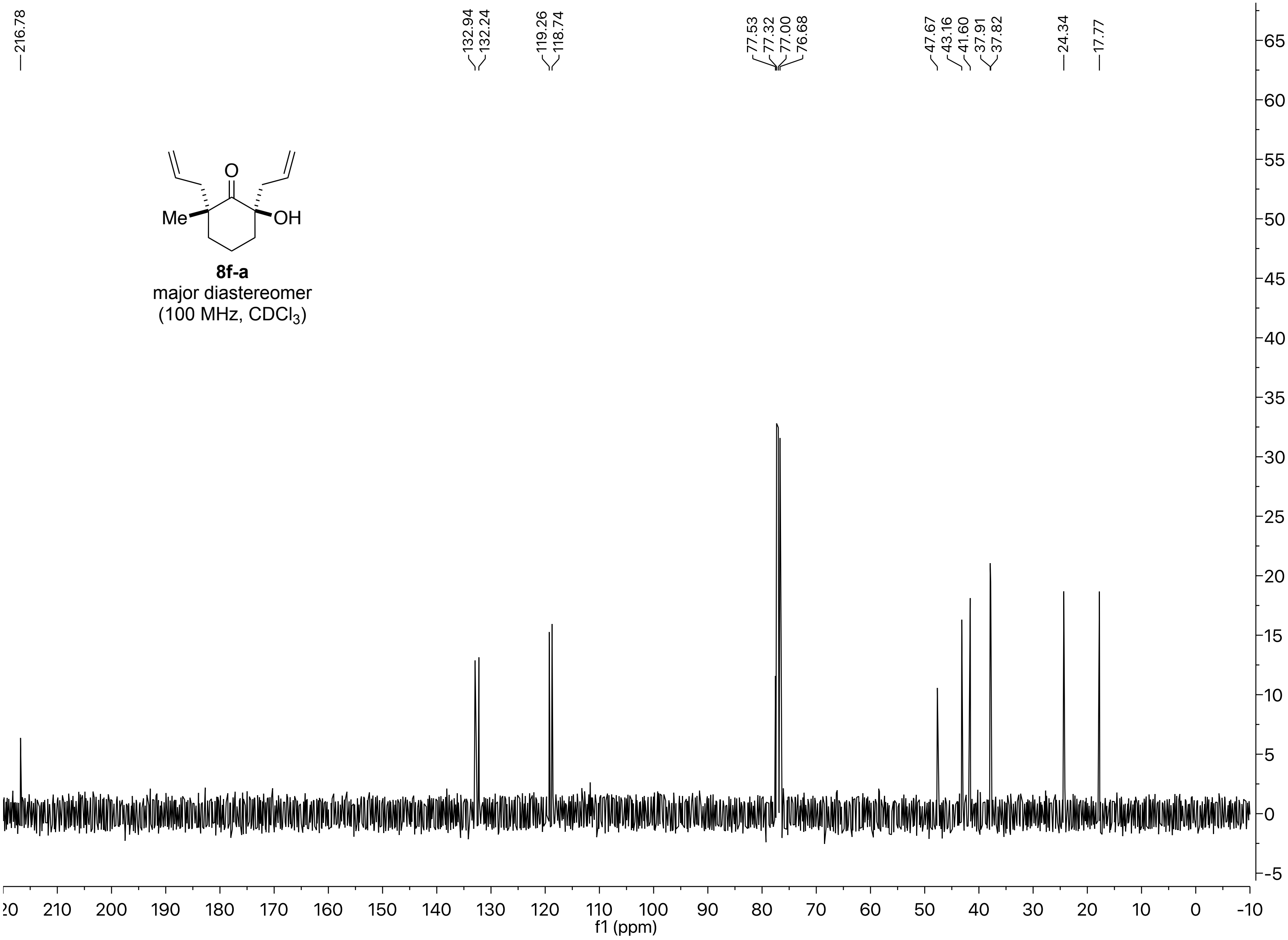
216.78
132.94
132.24
119.26
118.74
77.53
77.32
77.00
76.68
47.67
43.16
41.60
37.91
37.82
24.34
17.77
O
Me
OH
8f-a
major diastereomer
(100 MHz, $CDCl_3$)
f1 (ppm)

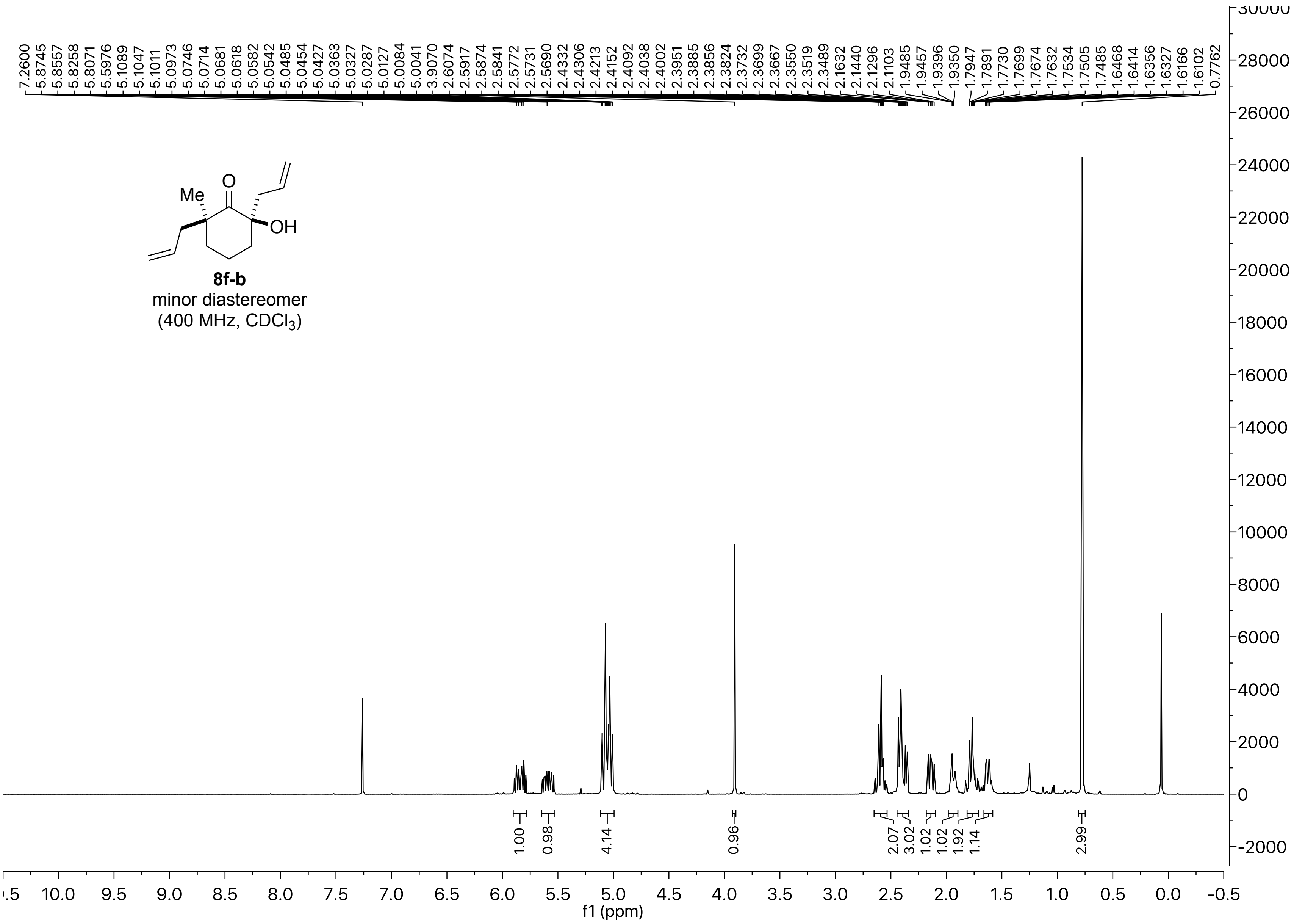
8f-b
minor diastereomer
(400 MHz, CDCl3)
f1 (ppm)

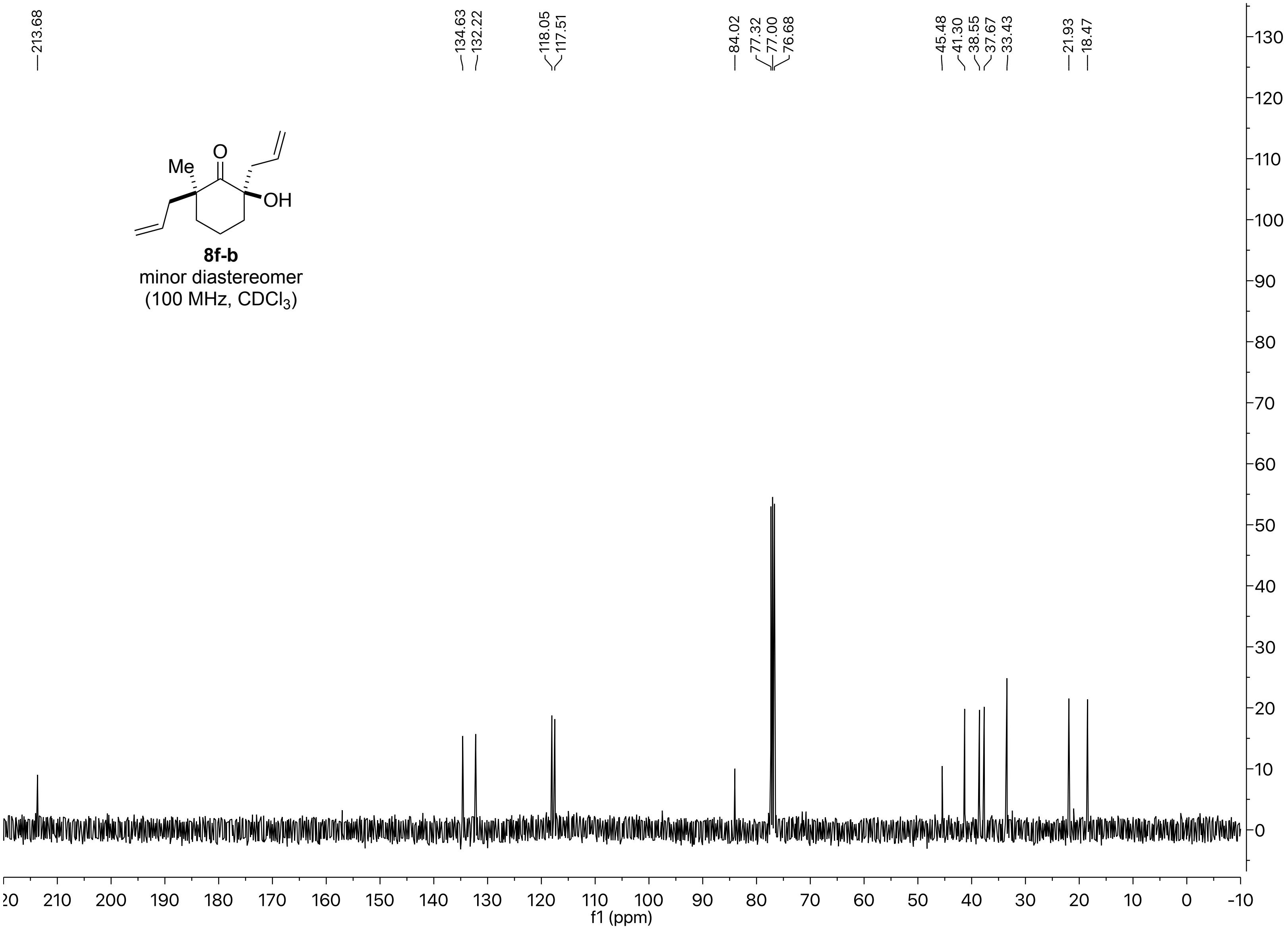
213.68
134.63
132.22
118.05
117.51
84.02
77.32
77.00
76.68
45.48
41.30
38.55
37.67
33.43
21.93
18.47
Me
O
OH
8f-b
minor diastereomer
(100 MHz, CDCl3)
f1 (ppm)

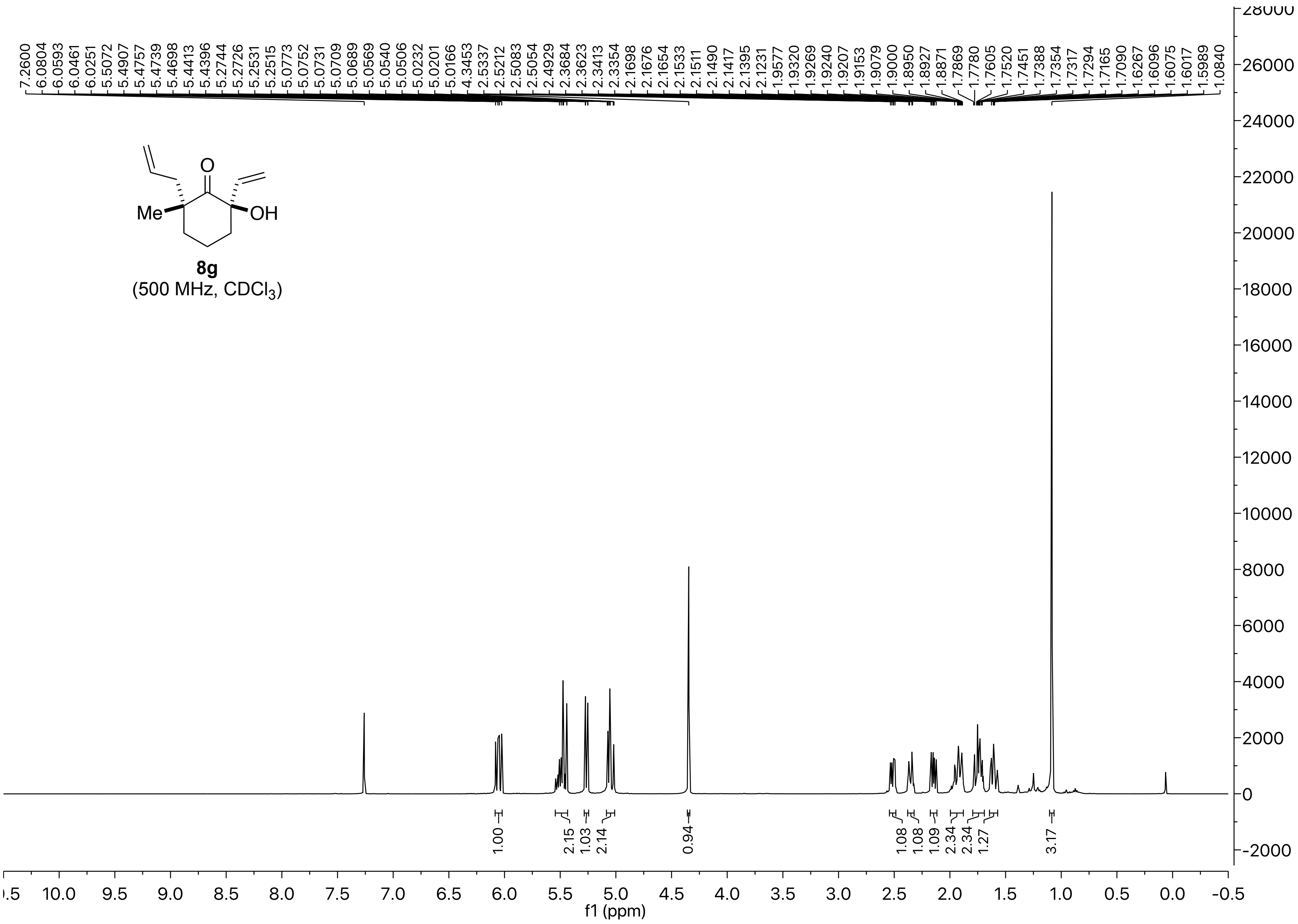

8g
(500 MHz, $CDCl_3$)

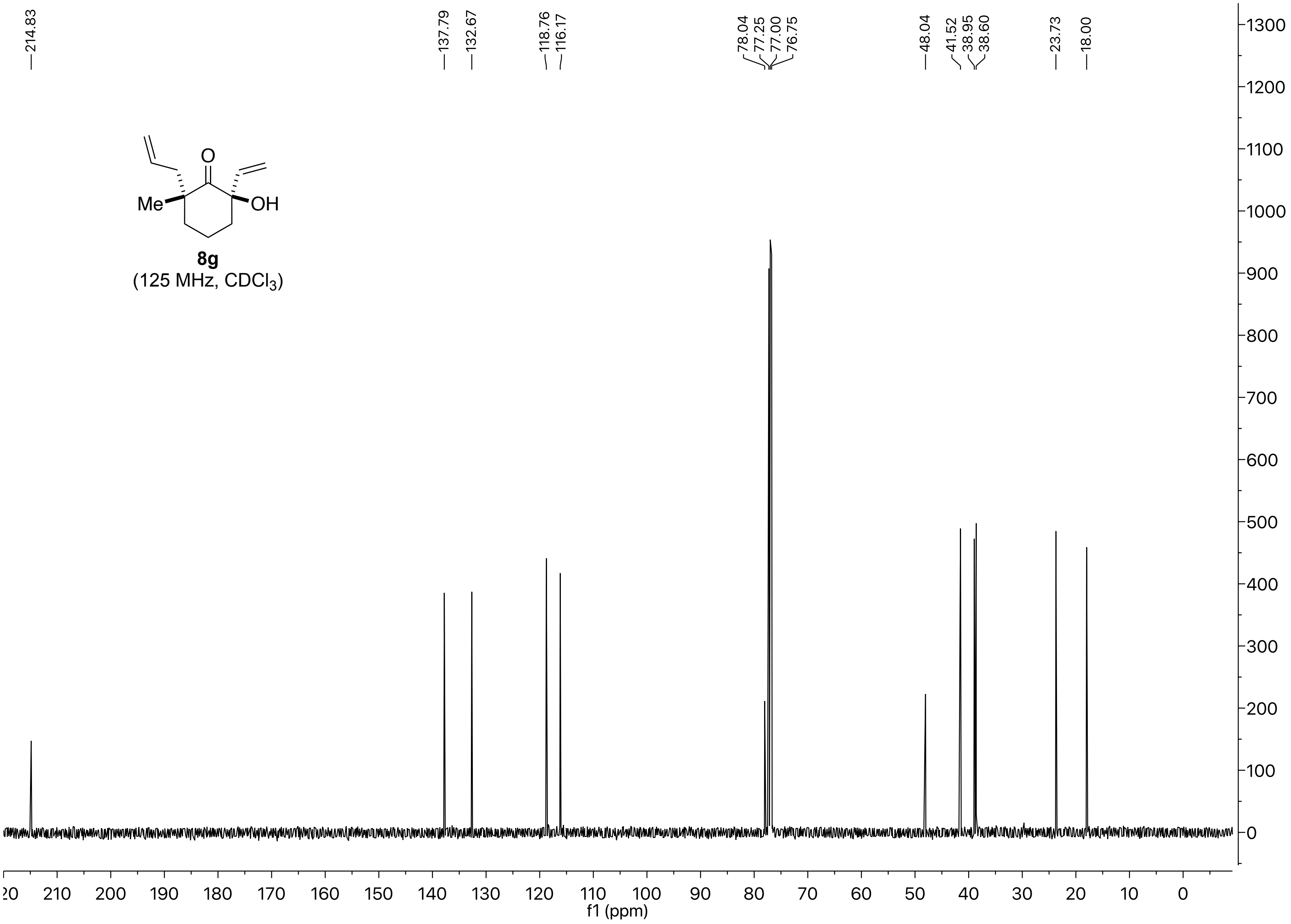

214.83
137.79
132.67
118.76
116.17
78.04
77.25
77.00
76.75
48.04
41.52
38.95
38.60
23.73
18.00
O
Me
OH
8g
(125 MHz, CDCl3)
f1 (ppm)

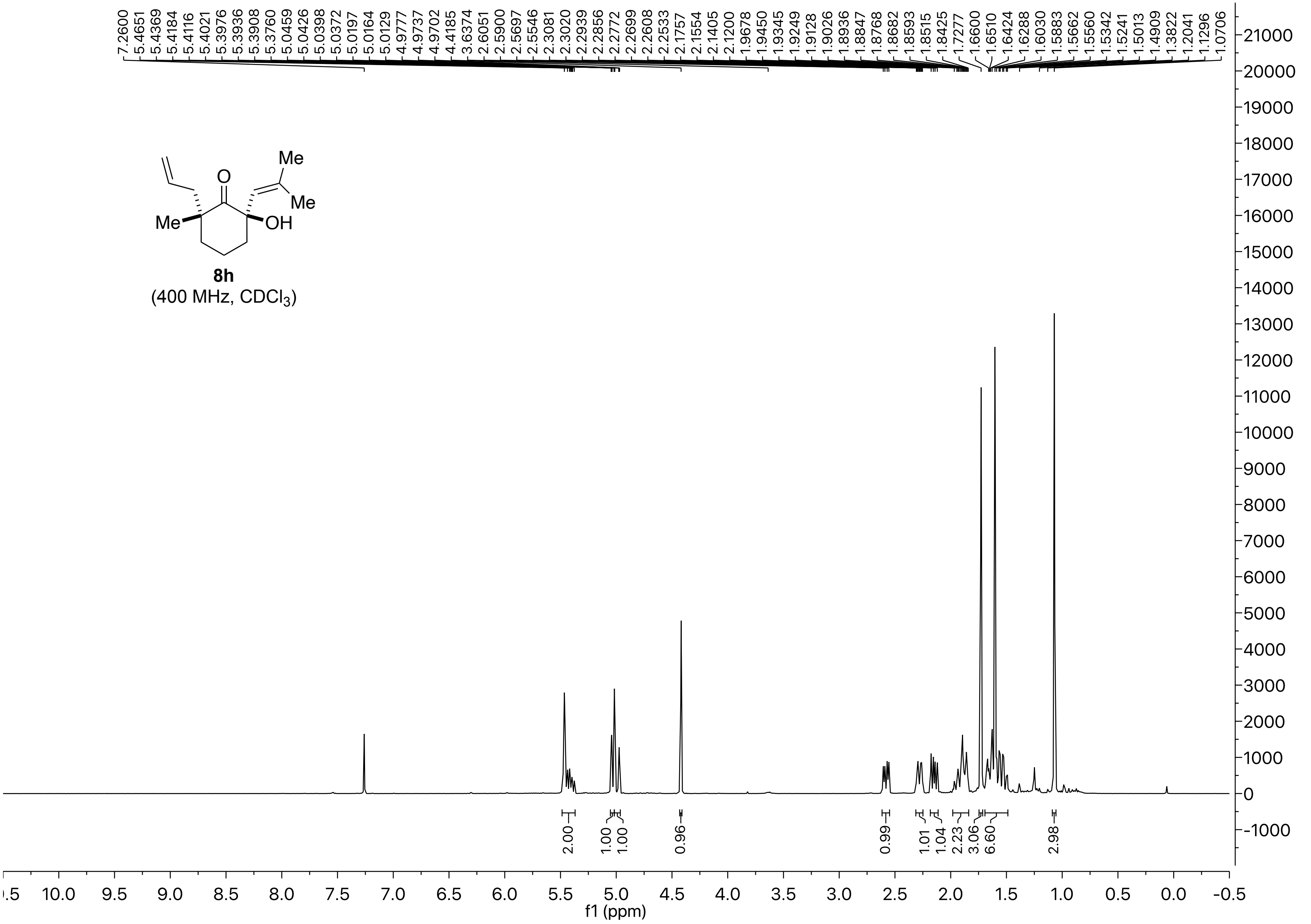

8h
(400 MHz, $CDCl_3$)

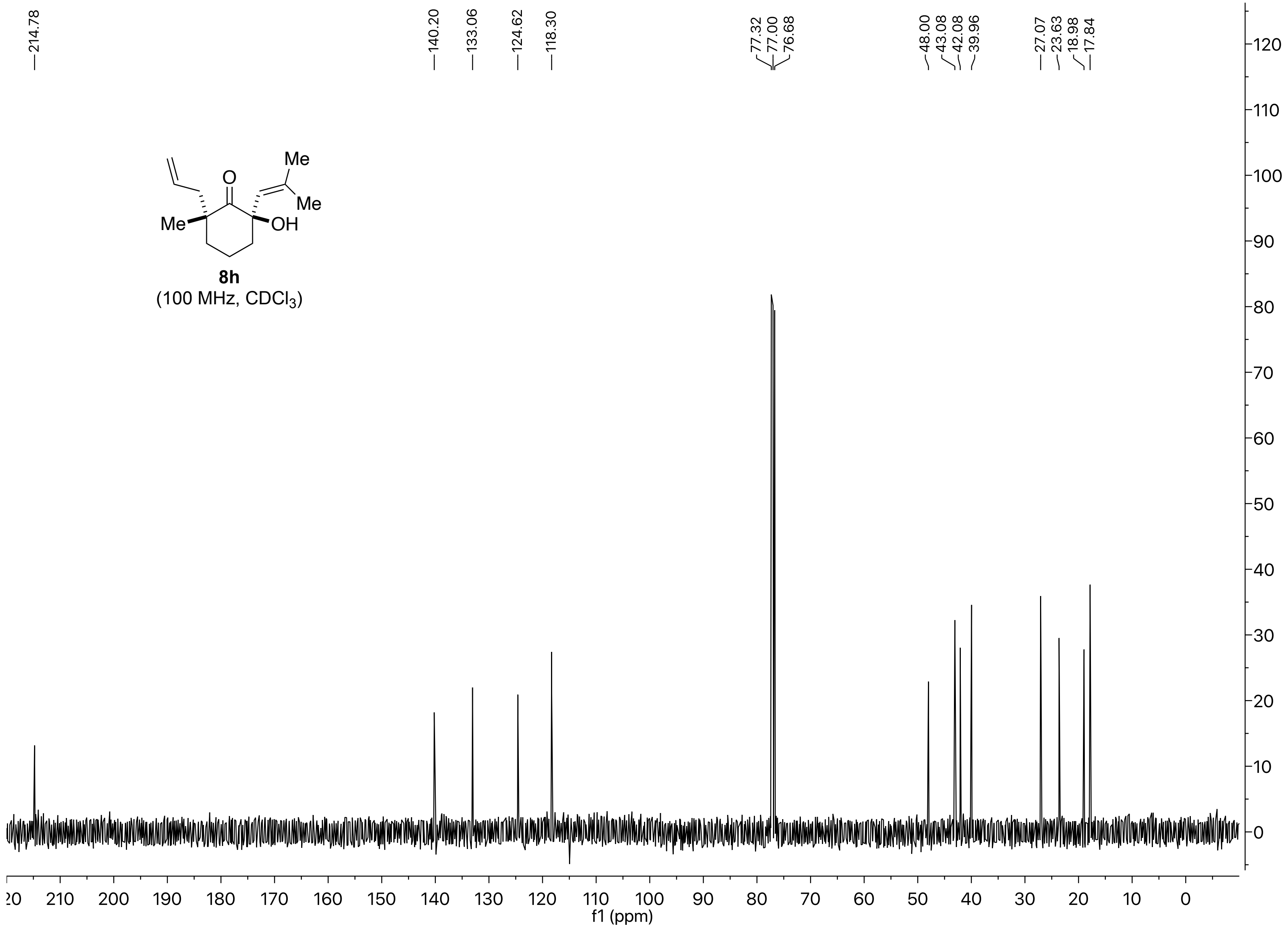
214.78
140.20
133.06
124.62
118.30
77.32
77.00
76.68
48.00
43.08
42.08
39.96
27.07
23.63
18.98
17.84
Me
O
Me
Me
OH
8h
(100 MHz, CDCl3)
f1 (ppm)

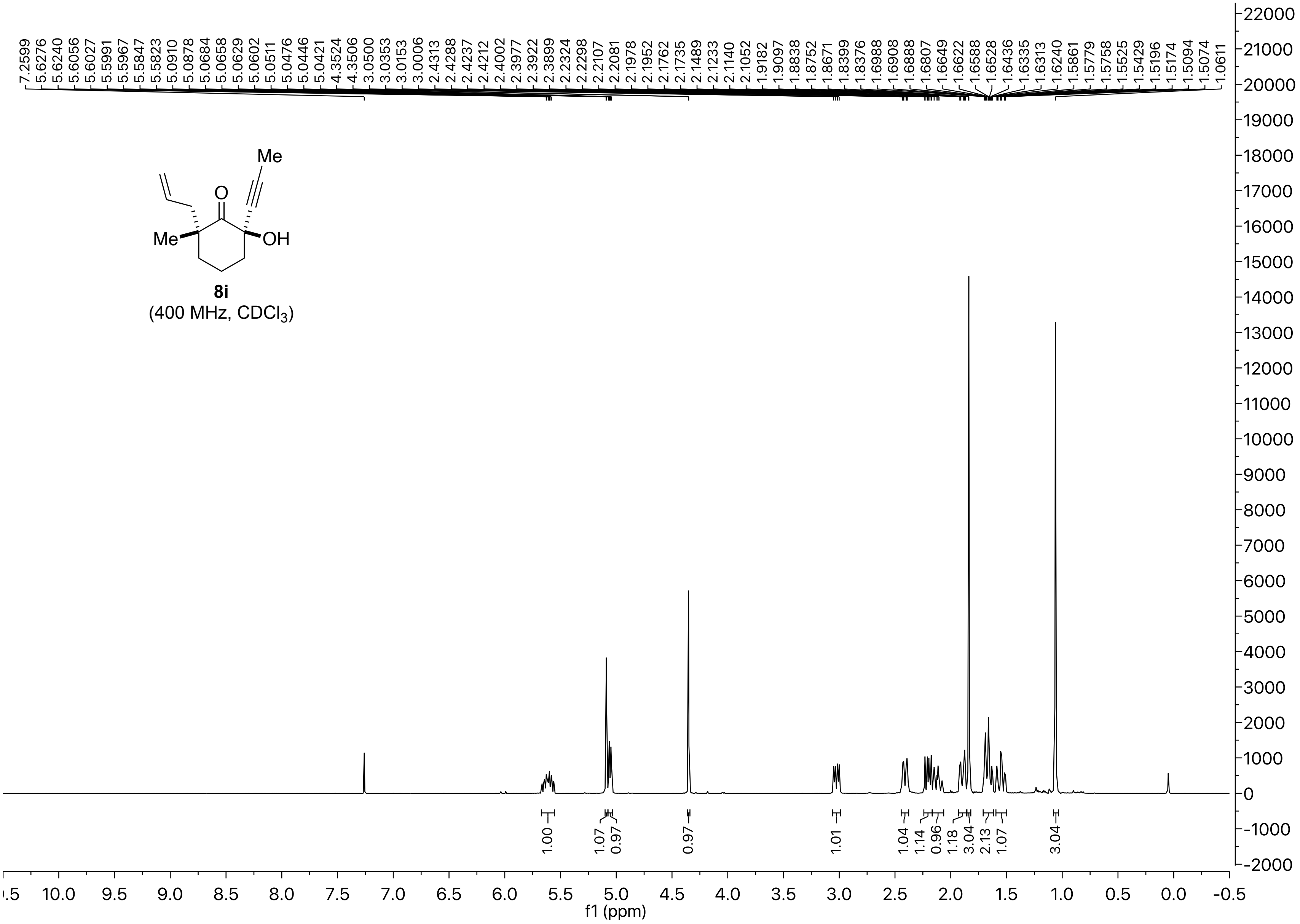

Me
O
Me
OH
8i
(400 MHz, CDCl3)
f1 (ppm)

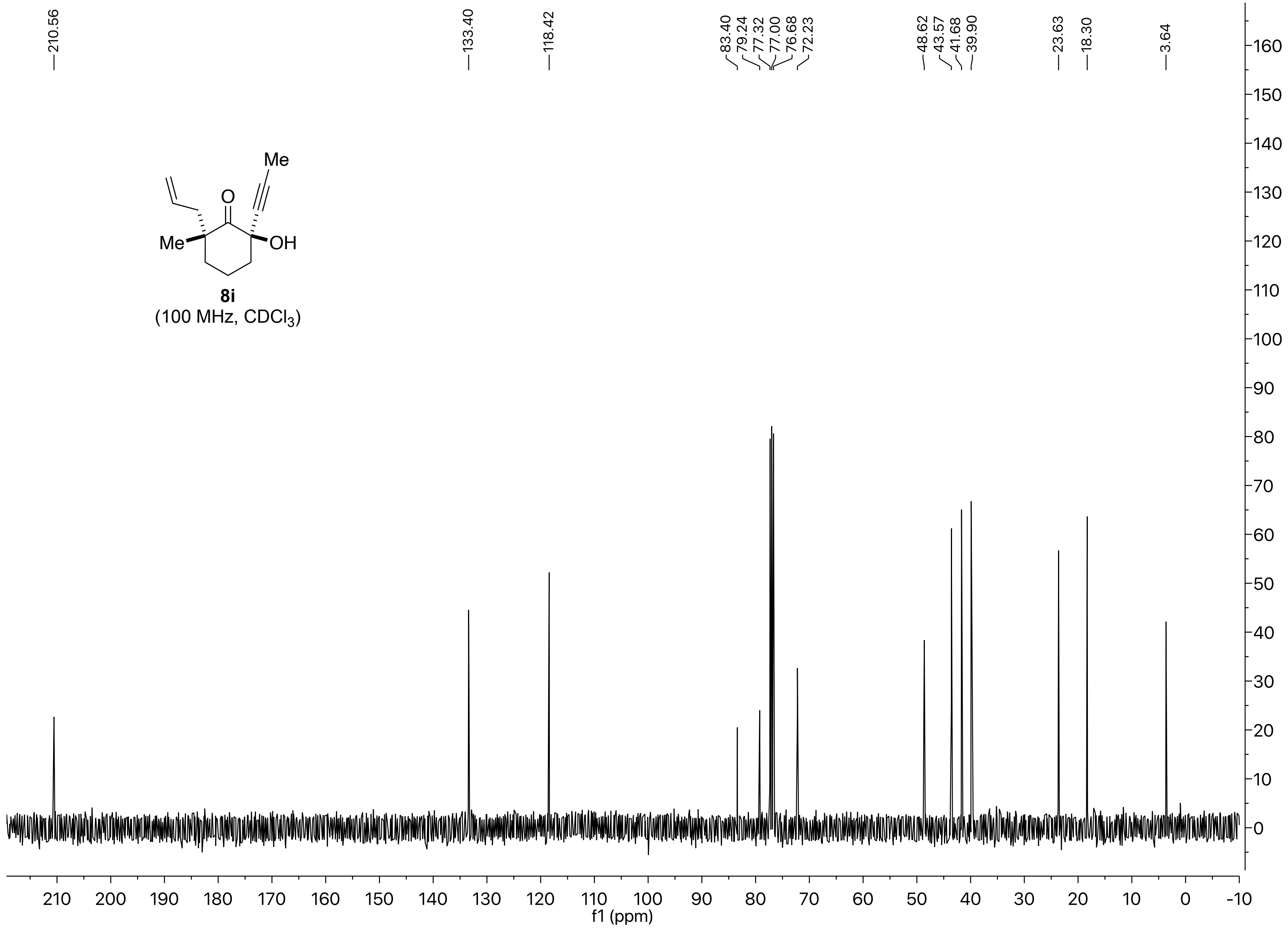

210.56
133.40
118.42
83.40
79.24
77.32
77.00
76.68
72.23
48.62
43.57
41.68
39.90
23.63
18.30
3.64
Me
O
Me
OH
8i
(100 MHz, CDCl3)
f1 (ppm)

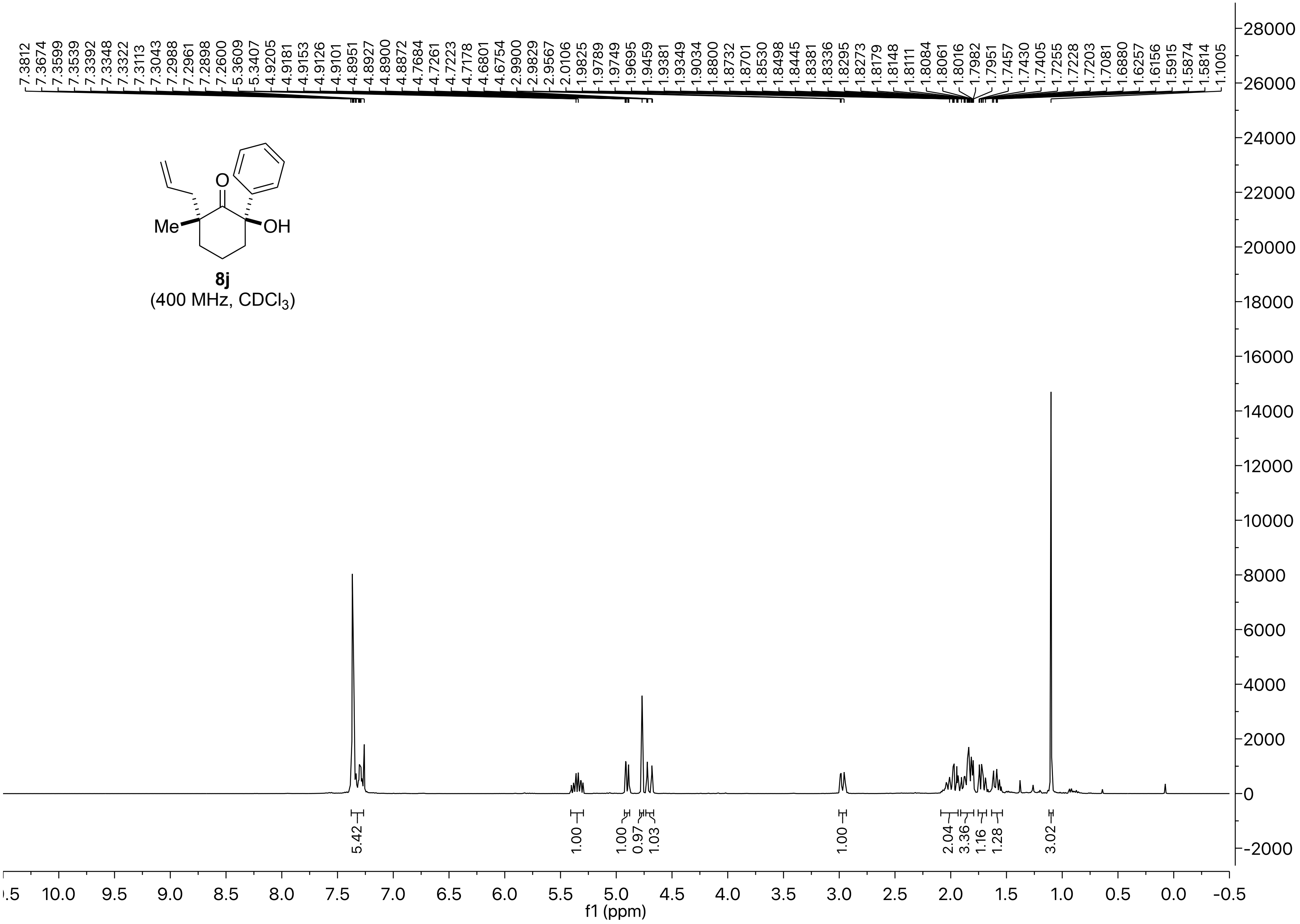

8j
(400 MHz, $CDCl_3$)

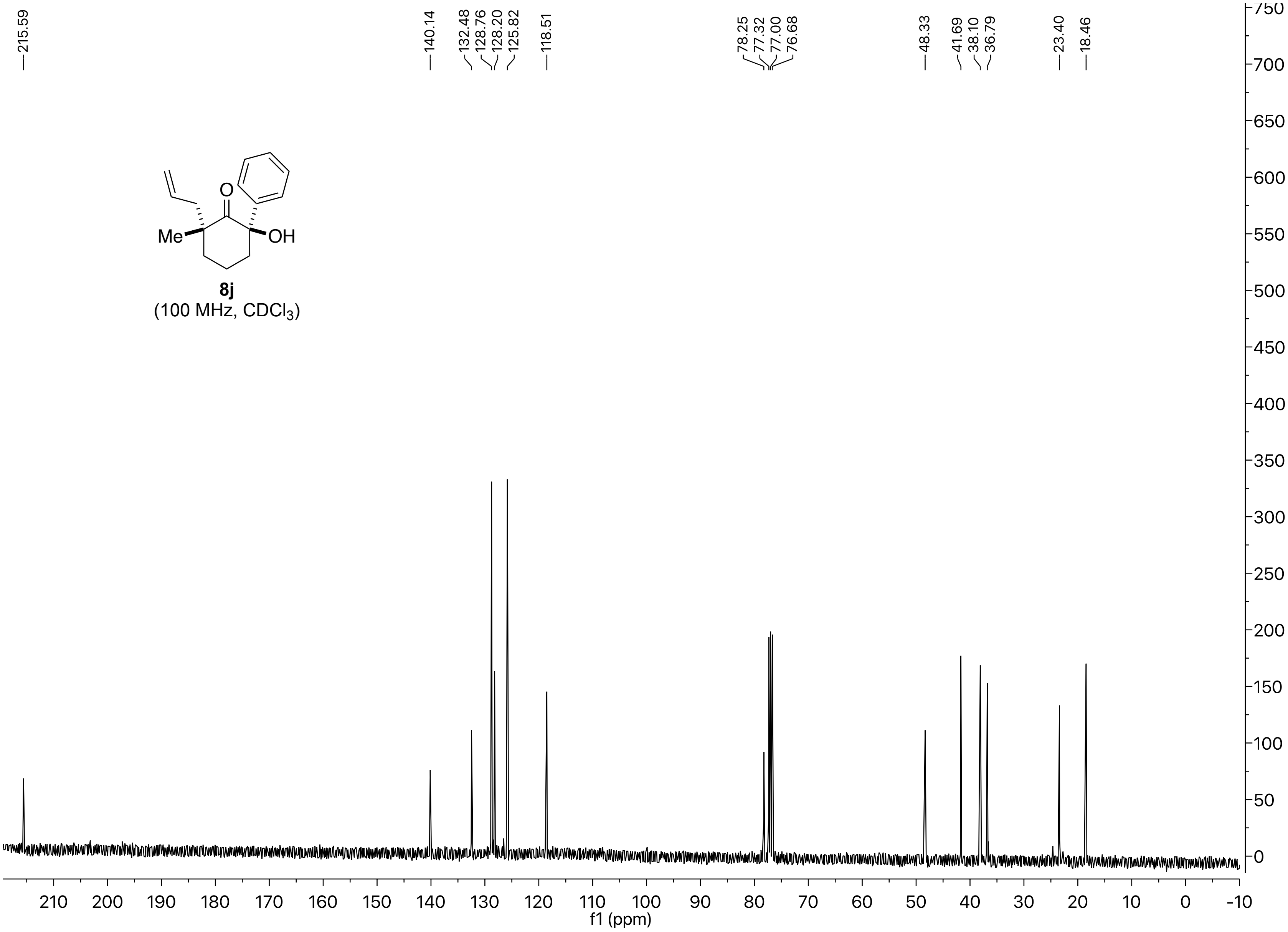

215.59
140.14
132.48
128.76
128.20
125.82
118.51
78.25
77.32
77.00
76.68
48.33
41.69
38.10
36.79
23.40
18.46
O
Me
OH
8j
(100 MHz, CDCl3)
f1 (ppm)

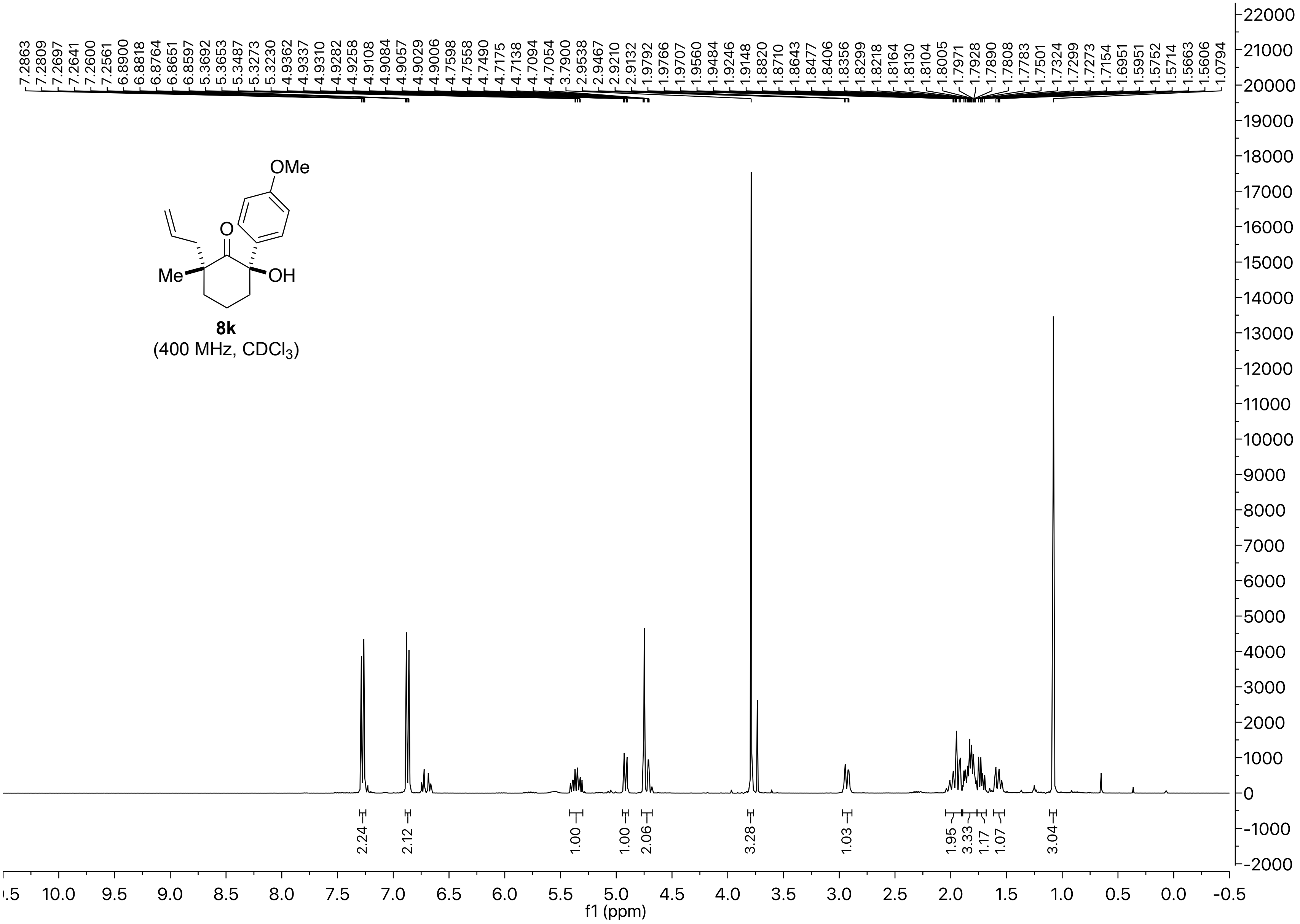
8k
(400 MHz, $CDCl_3$)
f1 (ppm)

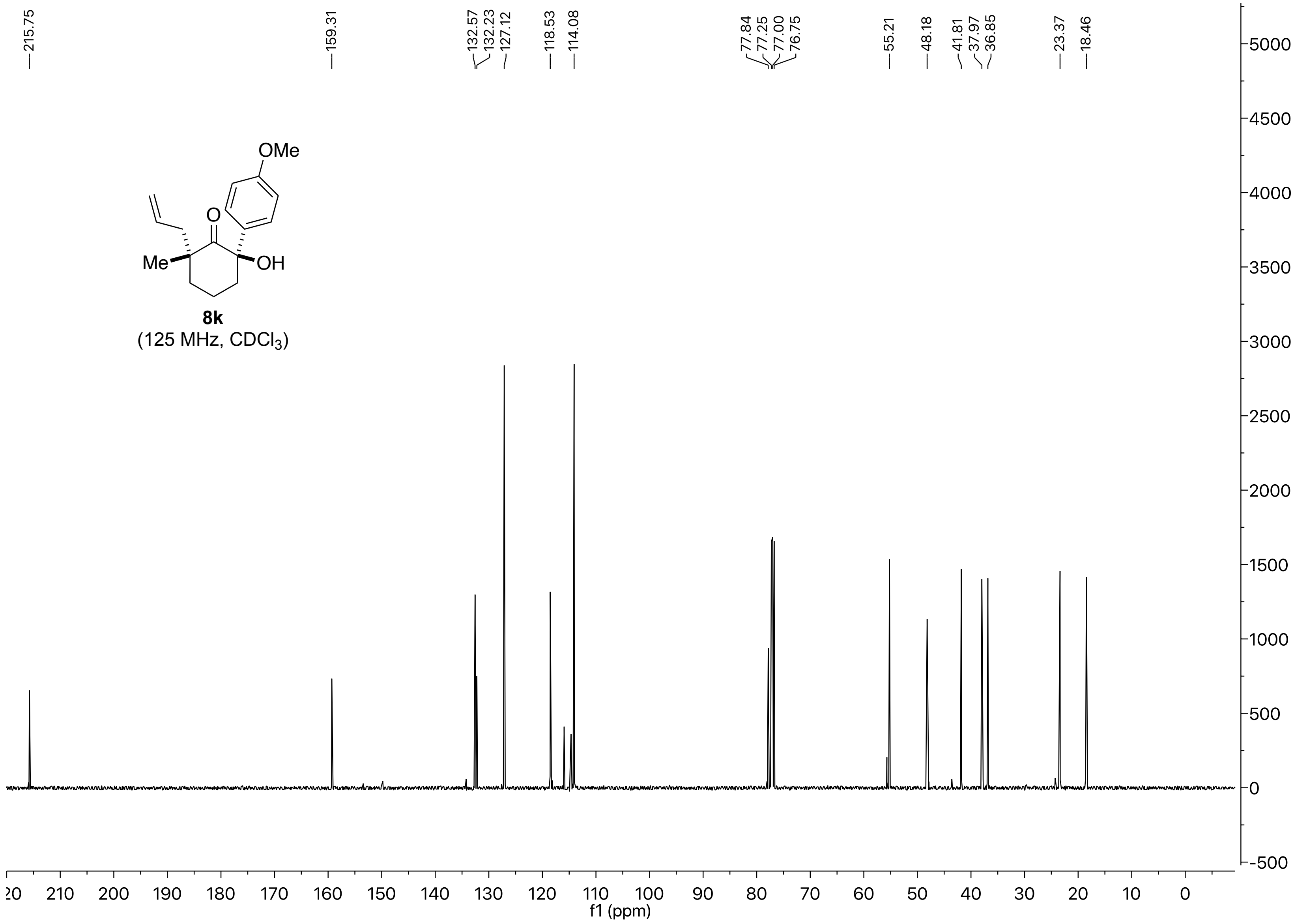
215.75
159.31
132.57
132.23
127.12
118.53
114.08
77.84
77.25
77.00
76.75
55.21
48.18
41.81
37.97
36.85
23.37
18.46
OMe
O
Me
OH
8k
(125 MHz, CDCl3)
f1 (ppm)

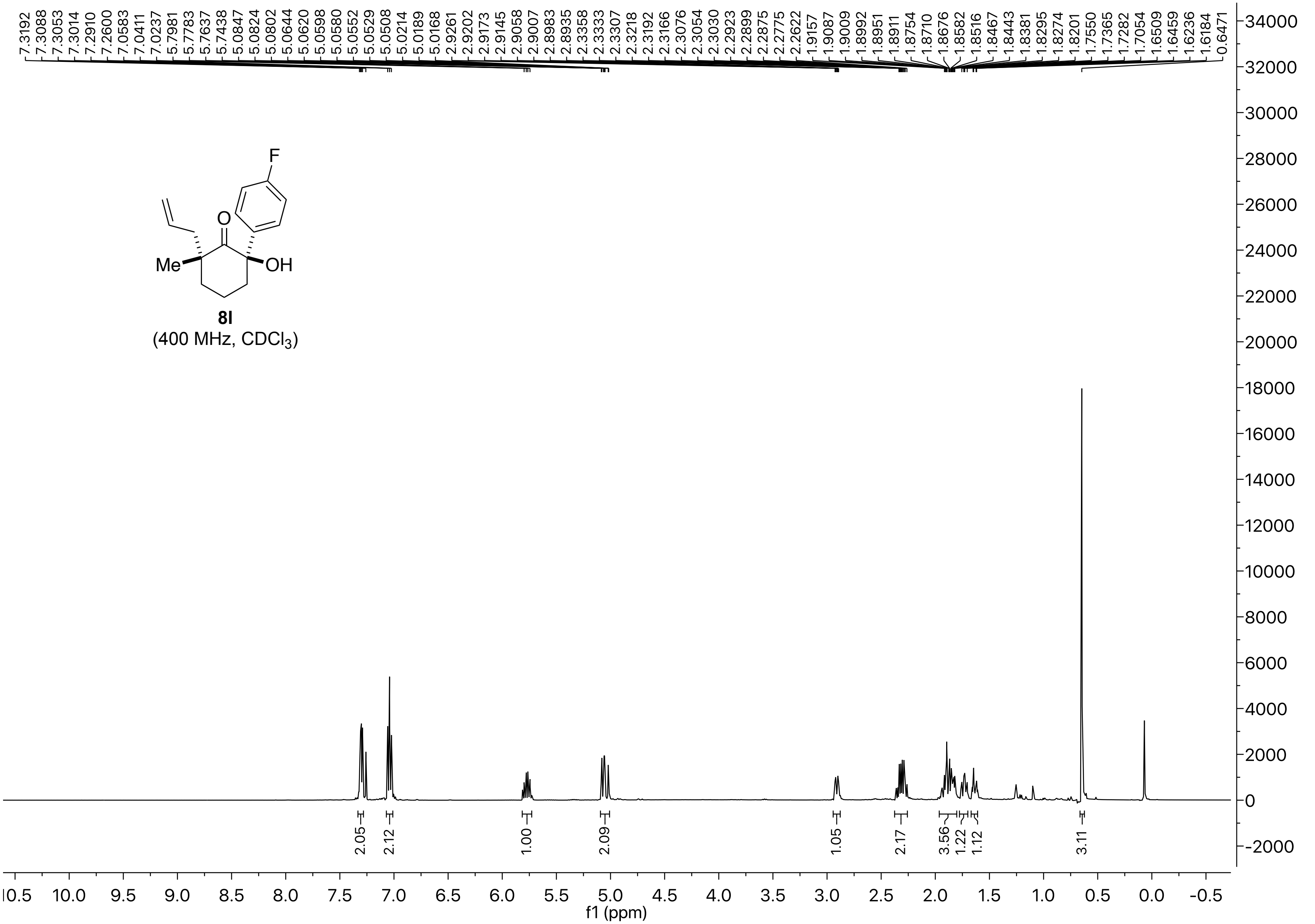

Me
OH
O
F
8l
(400 MHz, CDCl3)
f1 (ppm)

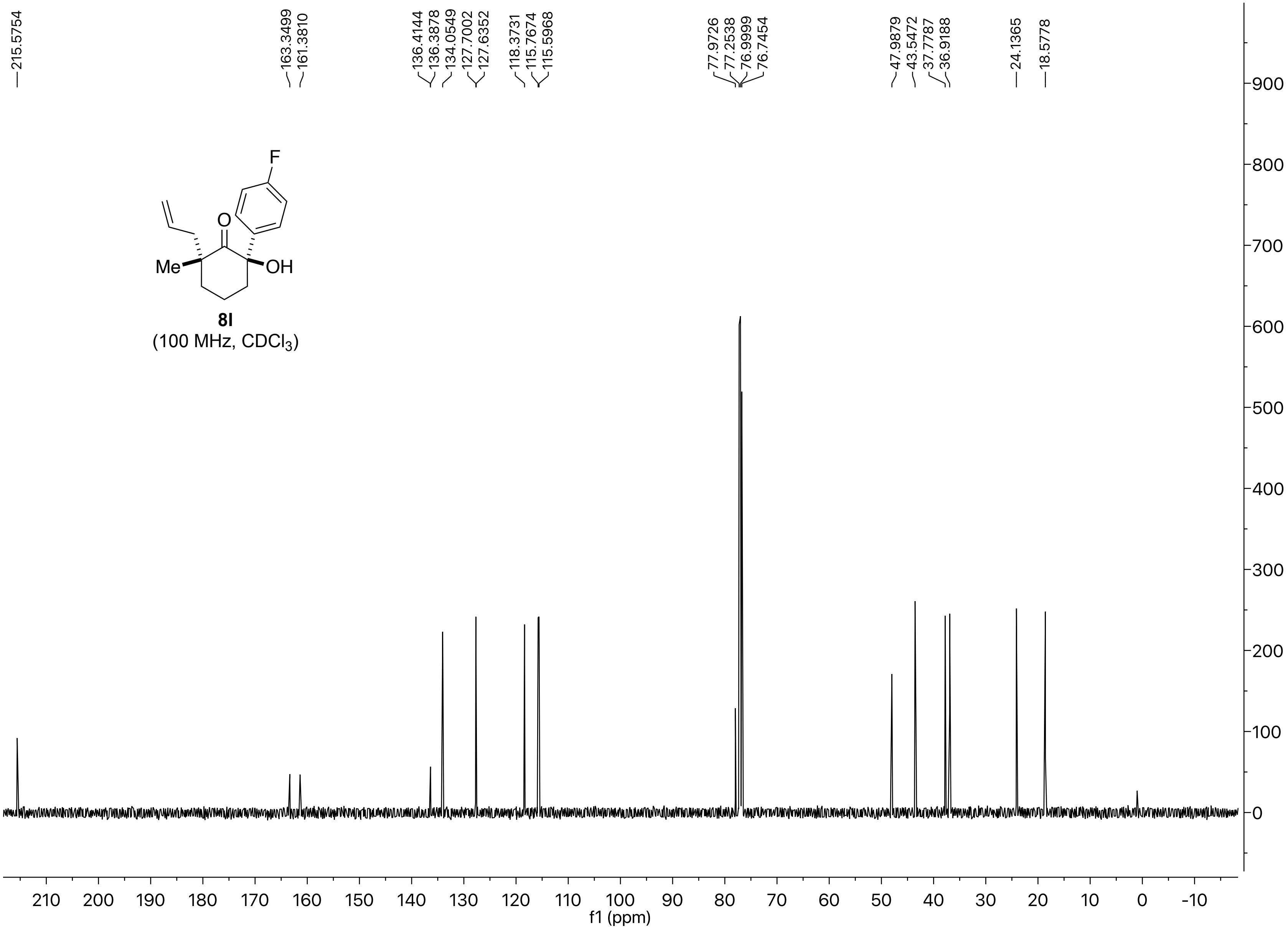
215.5754
163.3499
161.3810
136.4144
136.3878
134.0549
127.7002
127.6352
118.3731
115.7674
115.5968
77.9726
77.2538
76.9999
76.7454
47.9879
43.5472
37.7787
36.9188
24.1365
18.5778
F
O
Me
OH
8l
(100 MHz, $CDCl_3$)
f1 (ppm)

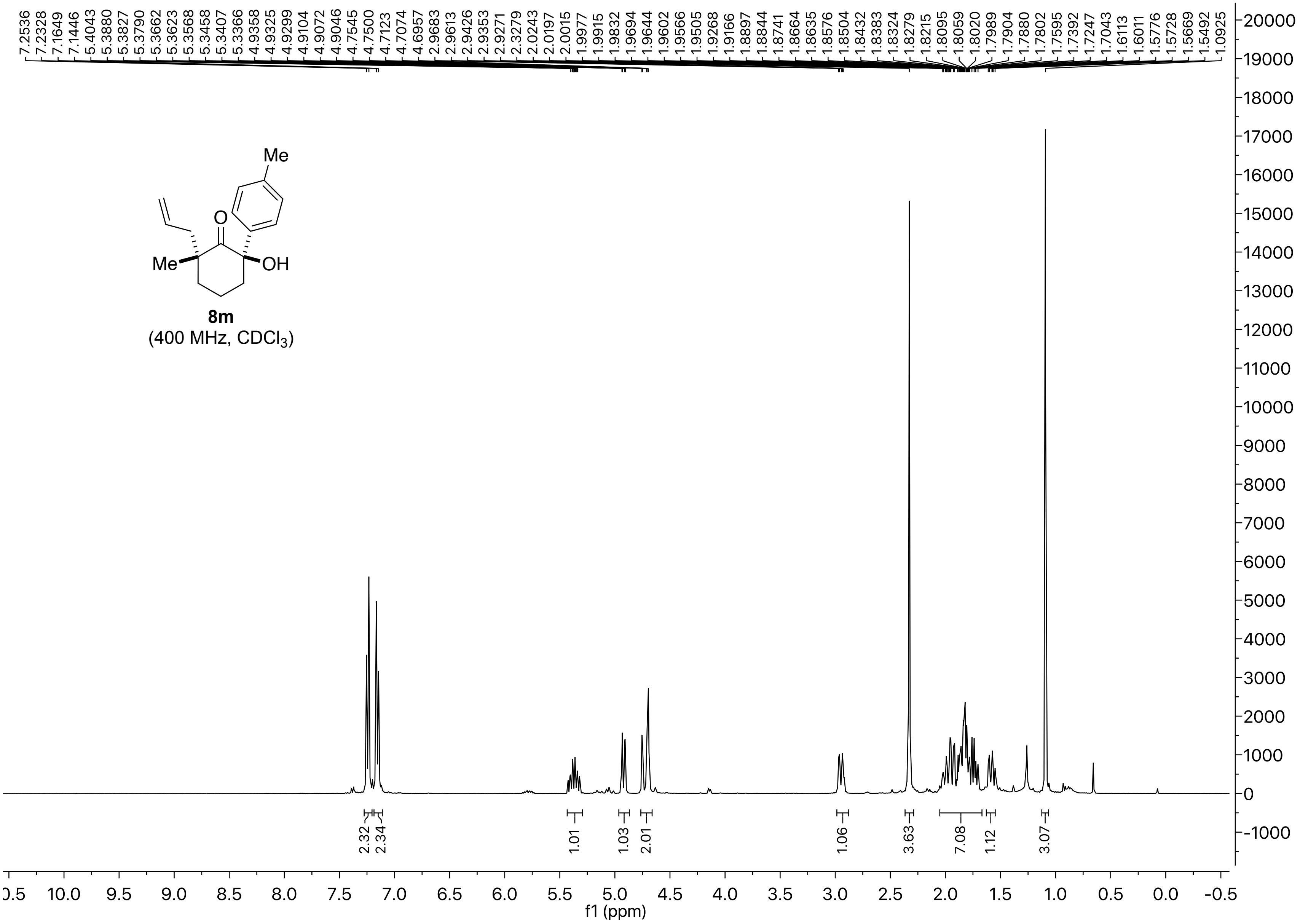
Me
O
Me
OH
8m
(400 MHz, $CDCl_3$)
f1 (ppm)

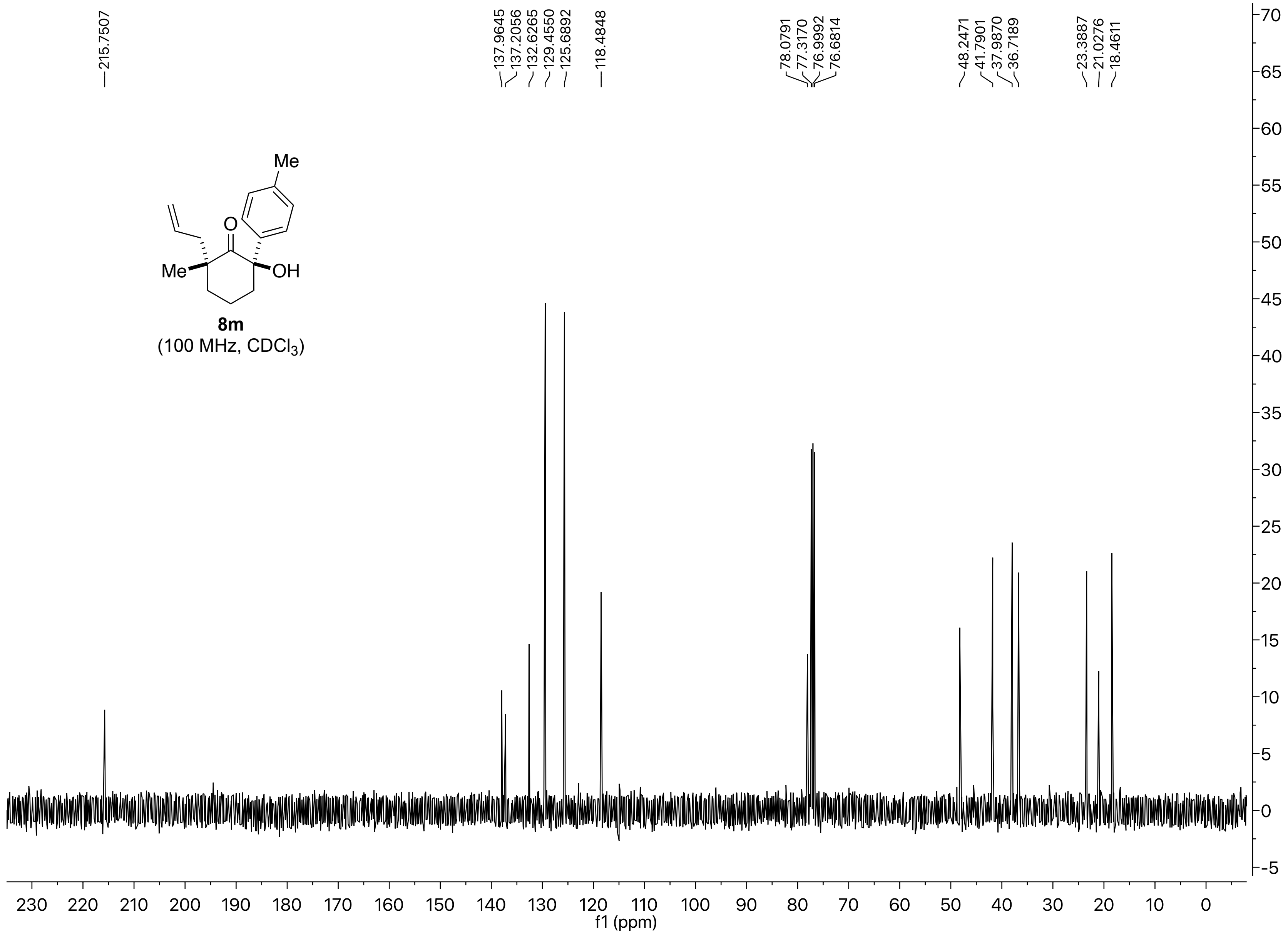
215.7507
137.9645
137.2056
132.6265
129.4550
125.6892
118.4848
78.0791
77.3170
76.9992
76.6814
48.2471
41.7901
37.9870
36.7189
23.3887
21.0276
18.4611
Me
O
Me
OH
8m
(100 MHz, CDCl3)
f1 (ppm)

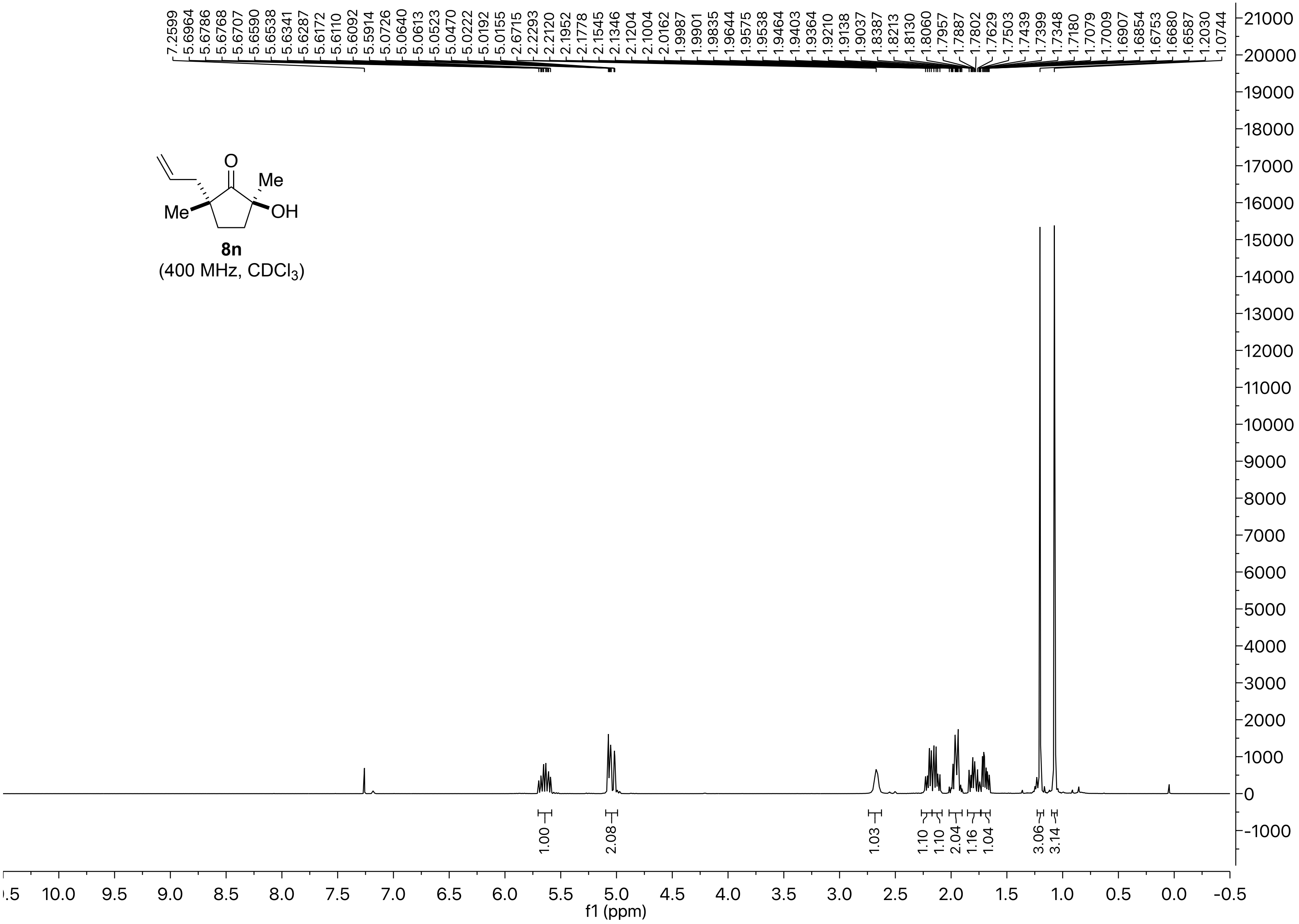

8n
(400 MHz, $CDCl_3$)

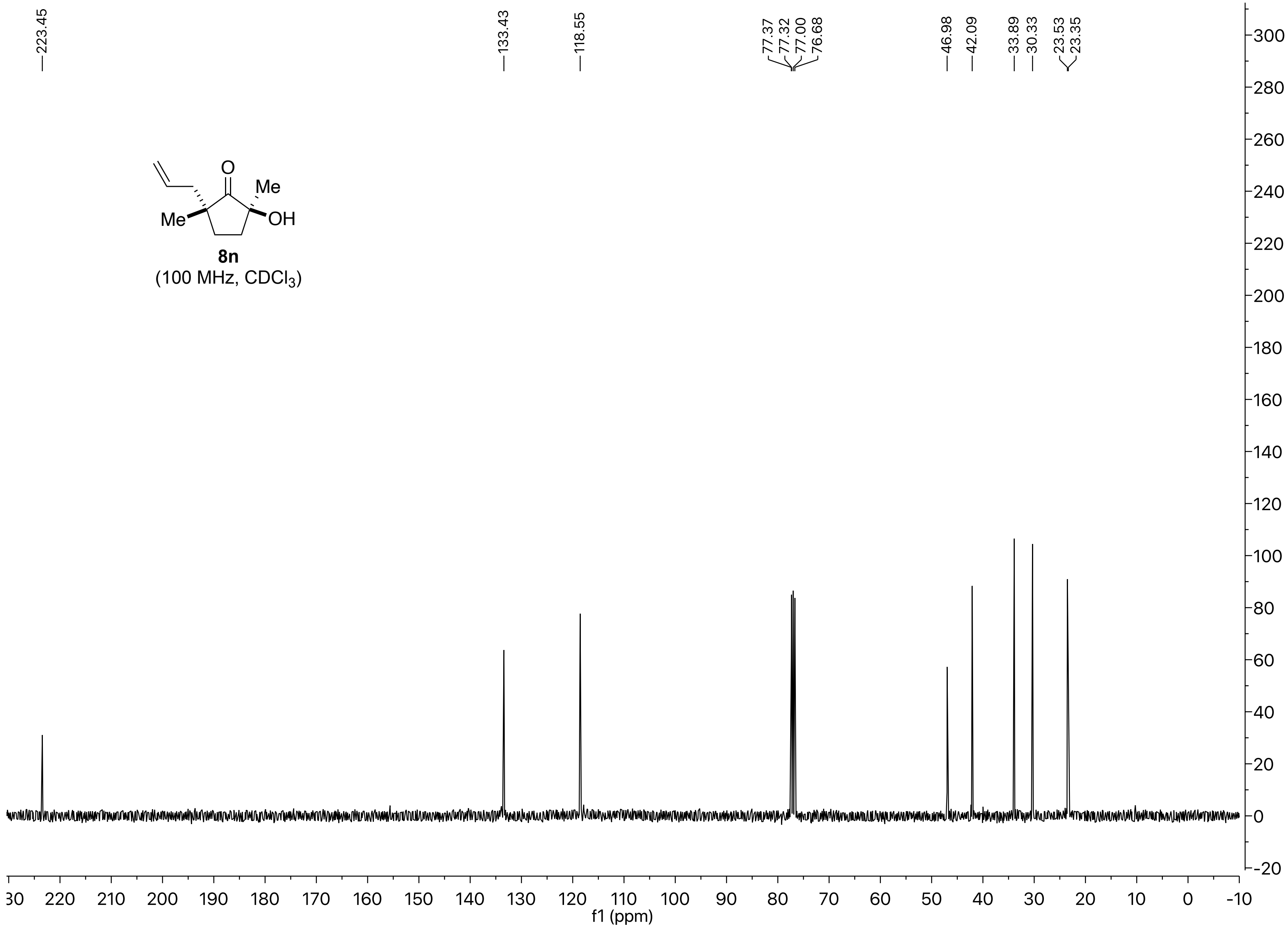

223.45
133.43
118.55
77.37
77.32
77.00
76.68
46.98
42.09
33.89
30.33
23.53
23.35
O
Me
Me
OH
8n
(100 MHz, CDCl3)
f1 (ppm)

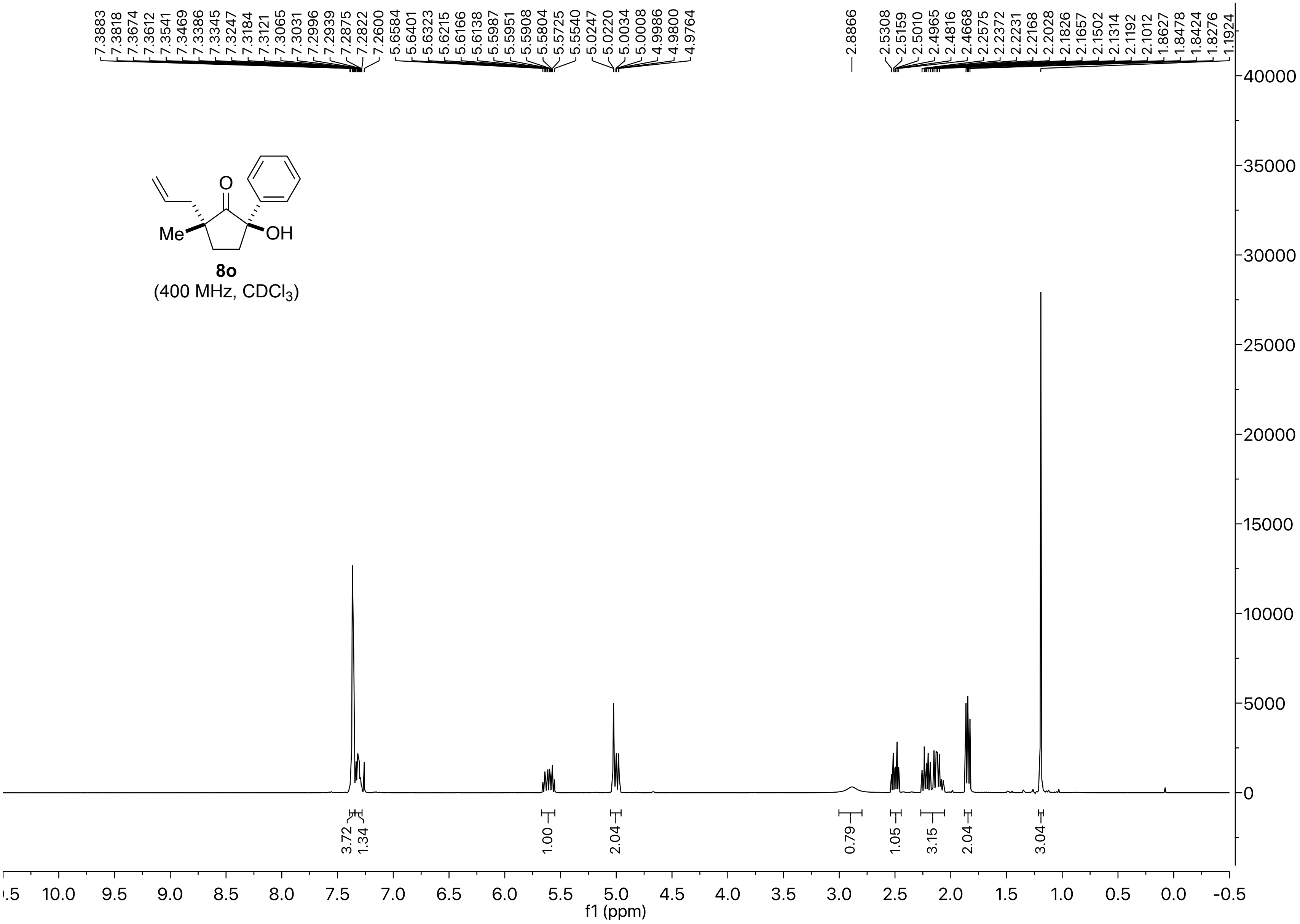

8o

(400 MHz, $CDCl_3$)

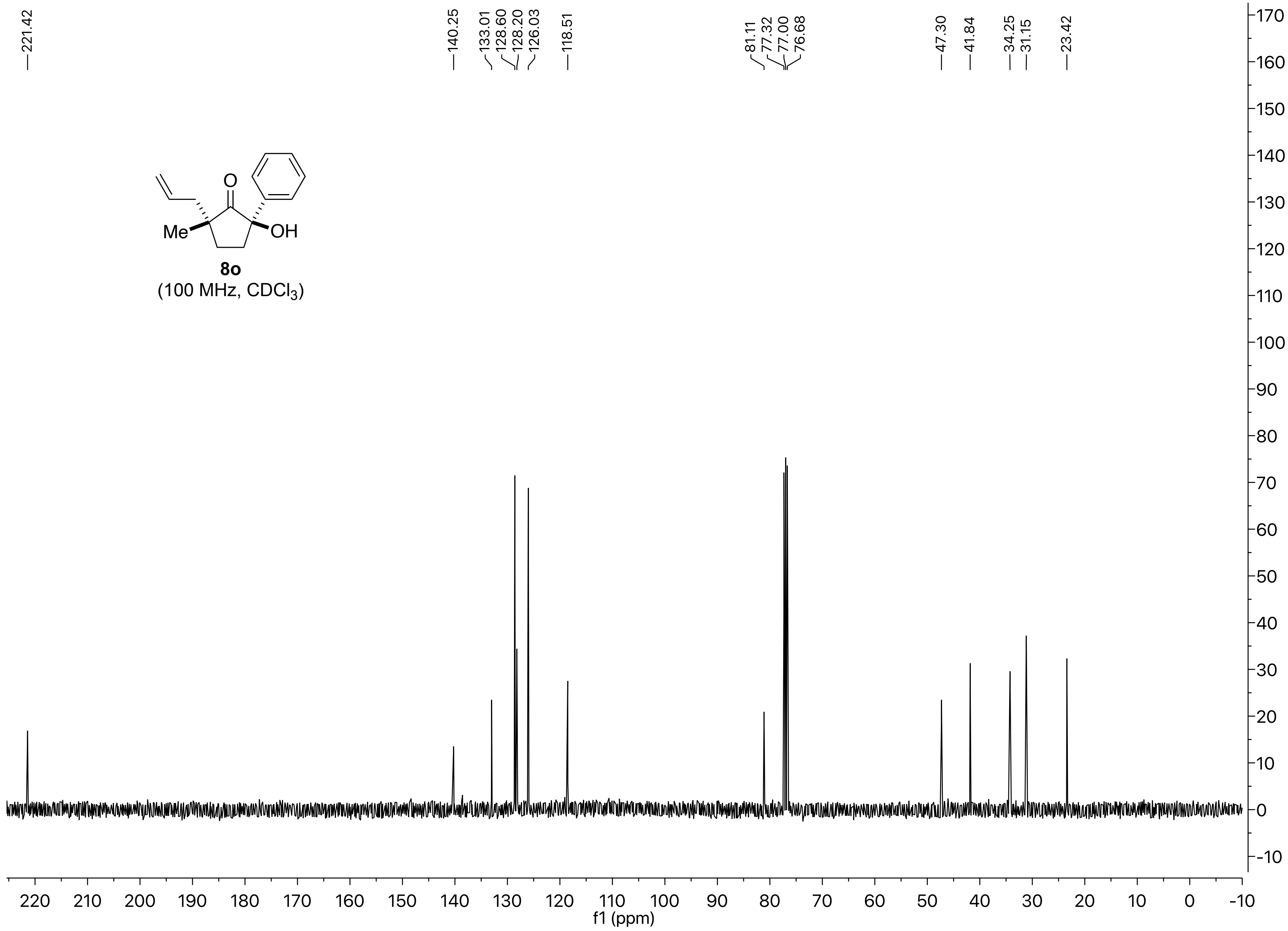
221.42
140.25
133.01
128.60
128.20
126.03
118.51
81.11
77.32
77.00
76.68
47.30
41.84
34.25
31.15
23.42
O
Me
OH
8o
(100 MHz, CDCl$_3$)
f1 (ppm)

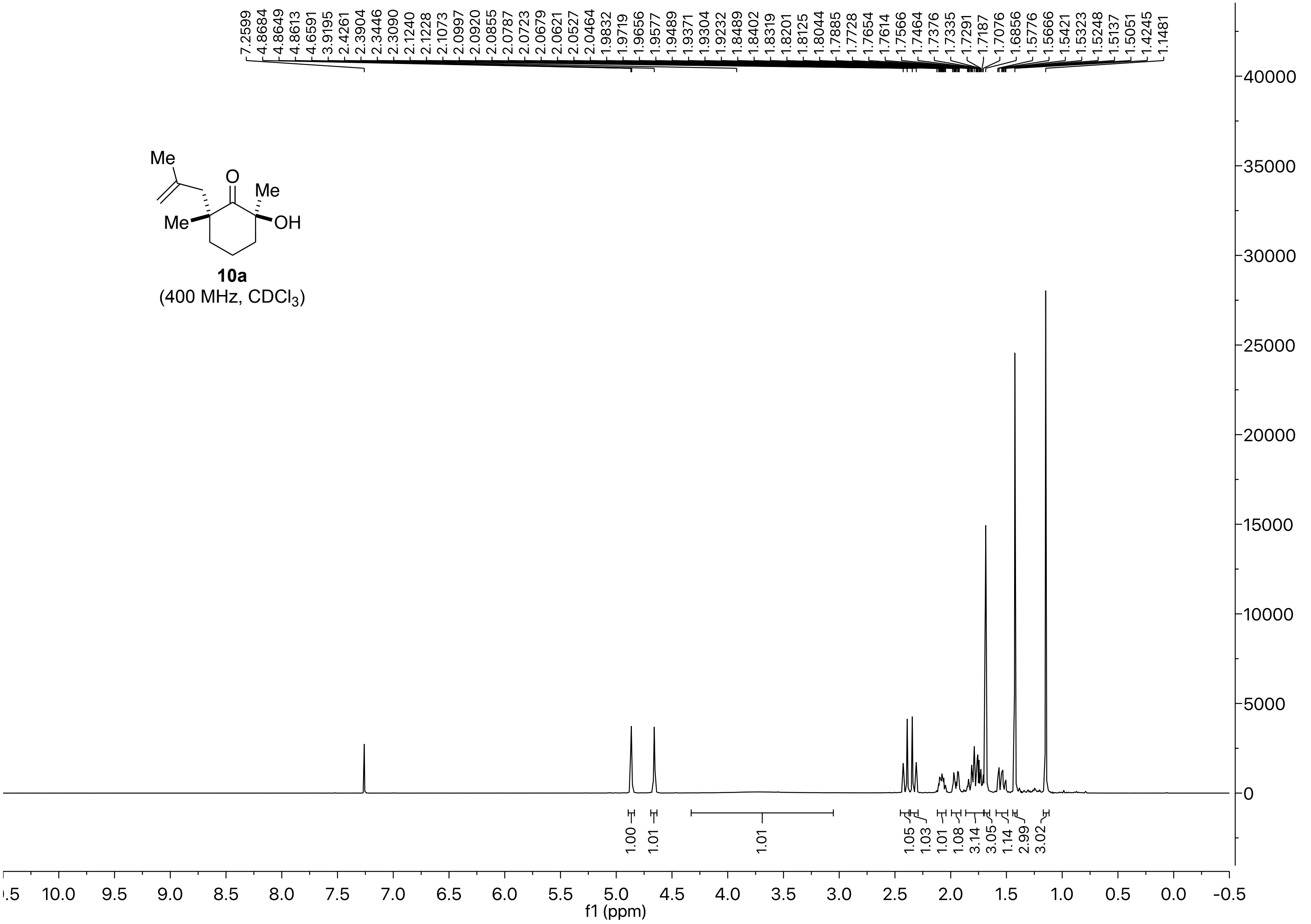

10a
(400 MHz, $CDCl_3$)

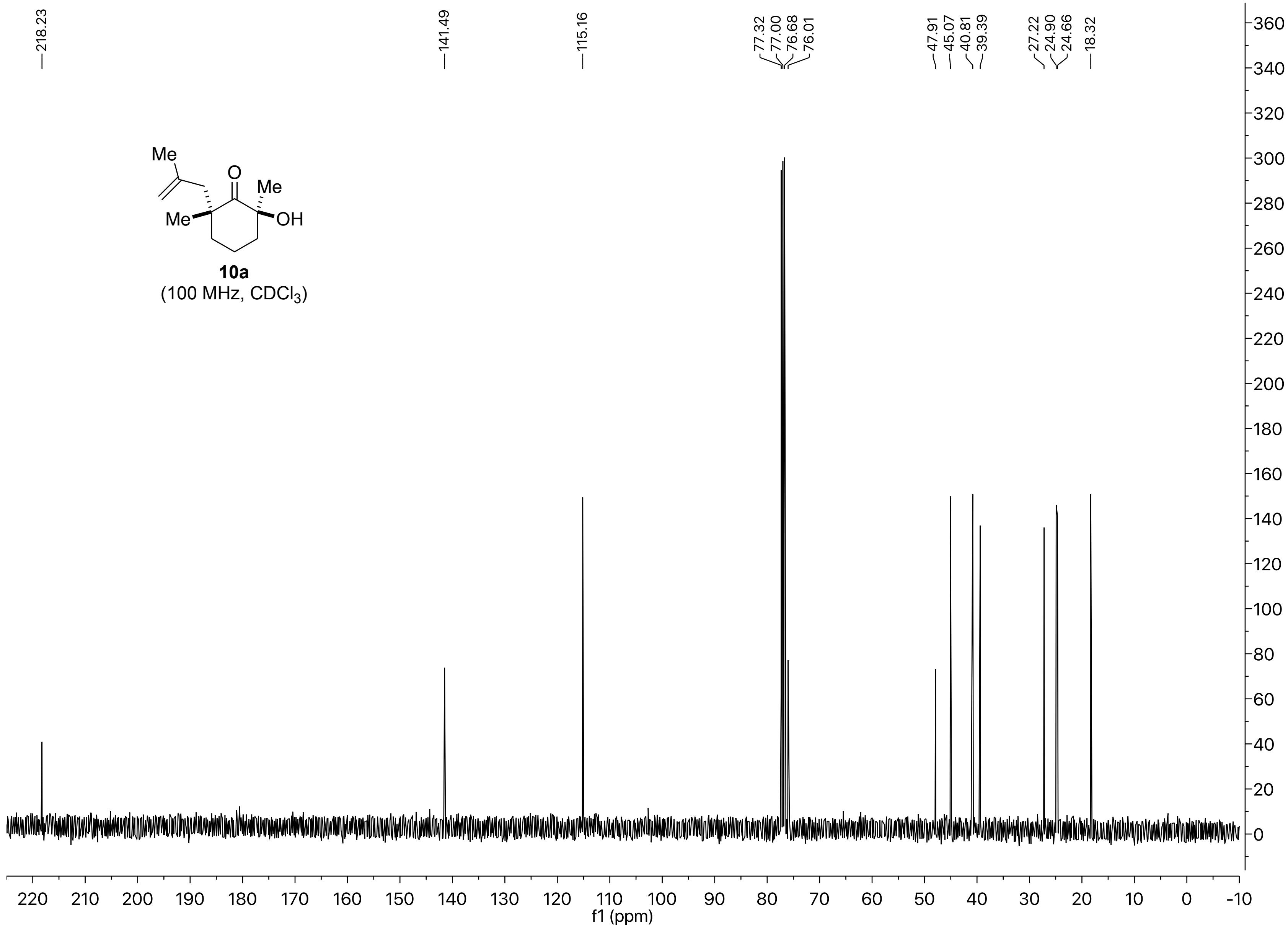

218.23
141.49
115.16
77.32
77.00
76.68
76.01
47.91
45.07
40.81
39.39
27.22
24.90
24.66
18.32
Me
O
Me
Me
OH
10a
(100 MHz, CDCl3)
f1 (ppm)

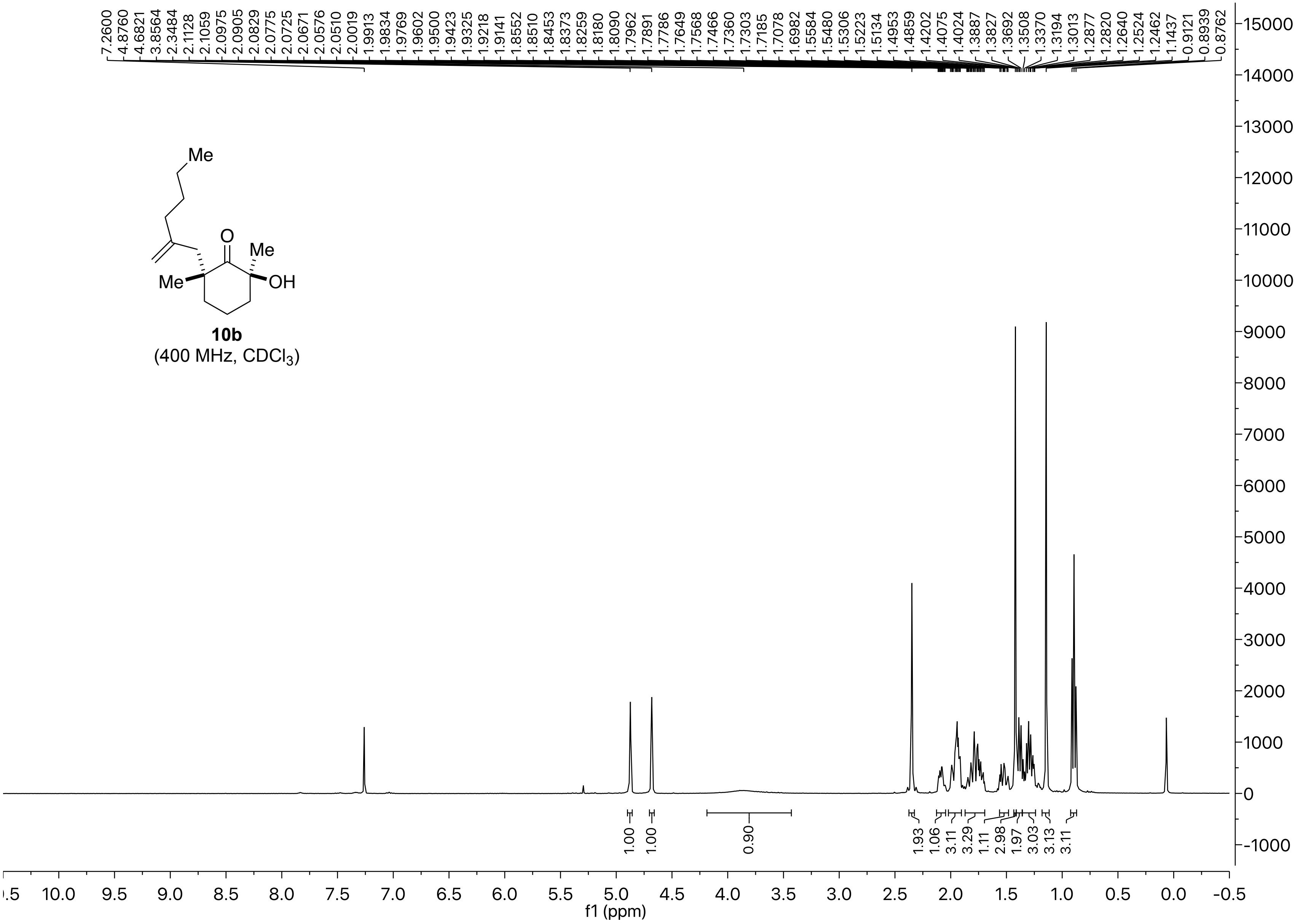
Me
O
Me
Me
OH
10b
(400 MHz, CDCl3)
f1 (ppm)

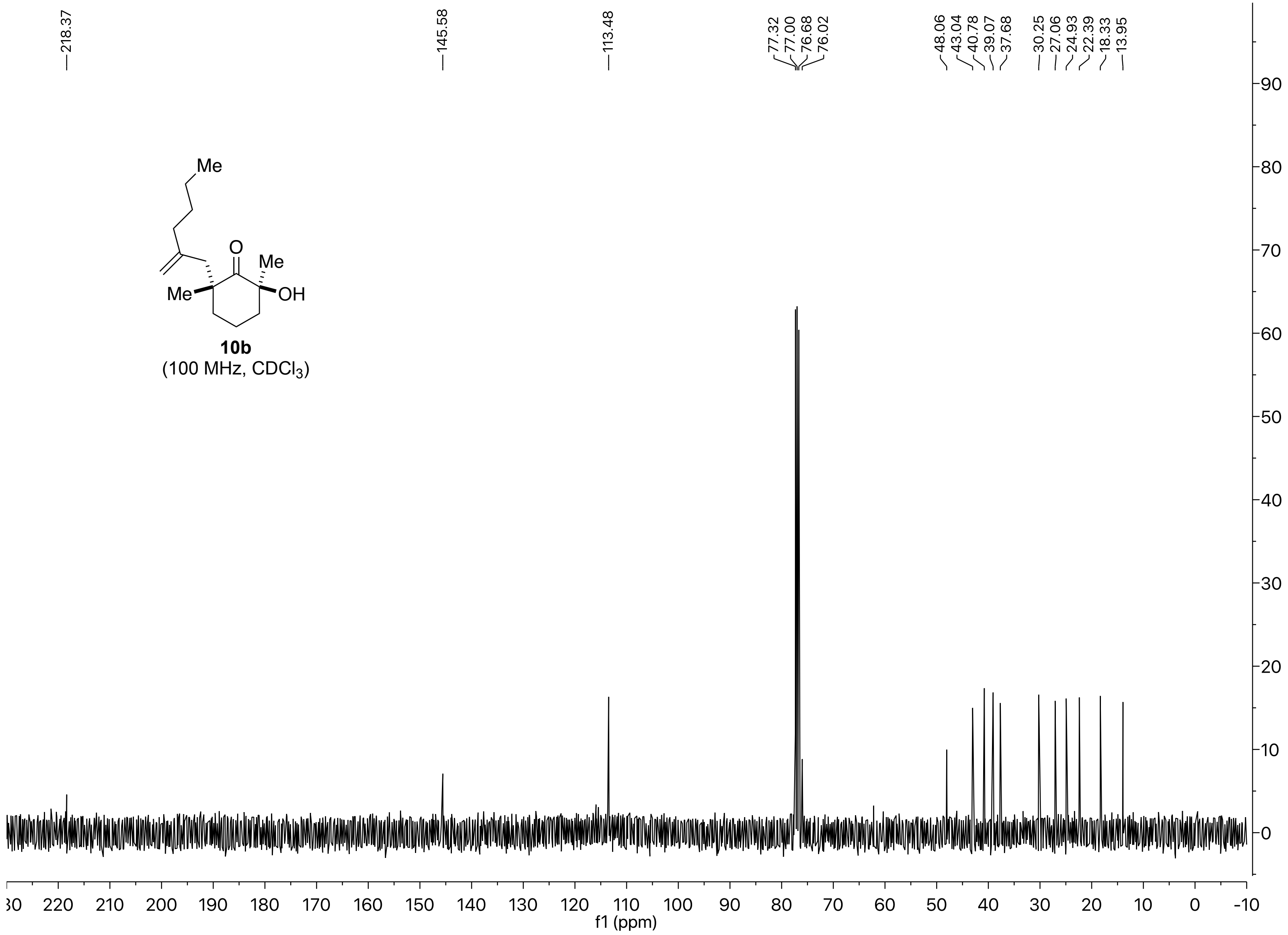
218.37
145.58
113.48
77.32
77.00
76.68
76.02
48.06
43.04
40.78
39.07
37.68
30.25
27.06
24.93
22.39
18.33
13.95
Me
O
Me
Me
OH
10b
(100 MHz, CDCl3)
f1 (ppm)

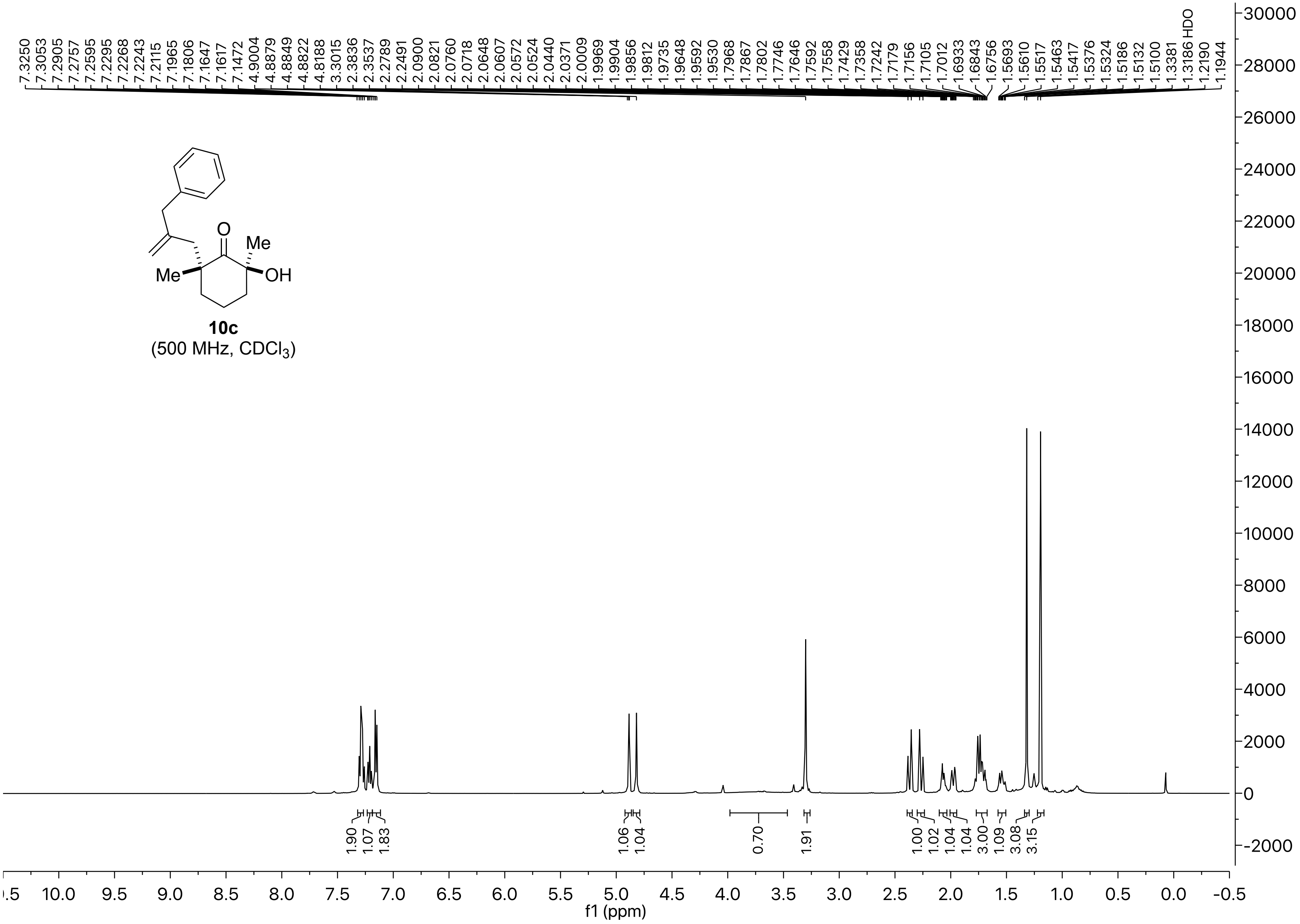
7.3250
7.3053
7.2905
7.2757
7.2595
7.2295
7.2268
7.2243
7.2115
7.1965
7.1806
7.1647
7.1617
7.1472
4.9004
4.8879
4.8849
4.8822
4.8188
3.3015
2.3836
2.3537
2.2789
2.2491
2.0900
2.0821
2.0760
2.0718
2.0648
2.0607
2.0572
2.0524
2.0440
2.0371
2.0009
1.9969
1.9904
1.9856
1.9812
1.9735
1.9648
1.9592
1.9530
1.7968
1.7867
1.7802
1.7746
1.7646
1.7592
1.7558
1.7429
1.7358
1.7242
1.7179
1.7156
1.7105
1.7012
1.6933
1.6843
1.6756
1.5693
1.5610
1.5517
1.5463
1.5417
1.5376
1.5324
1.5186
1.5132
1.5100
1.3381
1.3186 HDO
1.2190
1.1944
O
Me
Me
OH
10c
(500 MHz, CDCl3)
1.90
1.07
1.83
1.06
1.04
0.70
1.91
1.00
1.02
1.04
1.04
3.00
1.09
3.08
3.15
f1 (ppm)

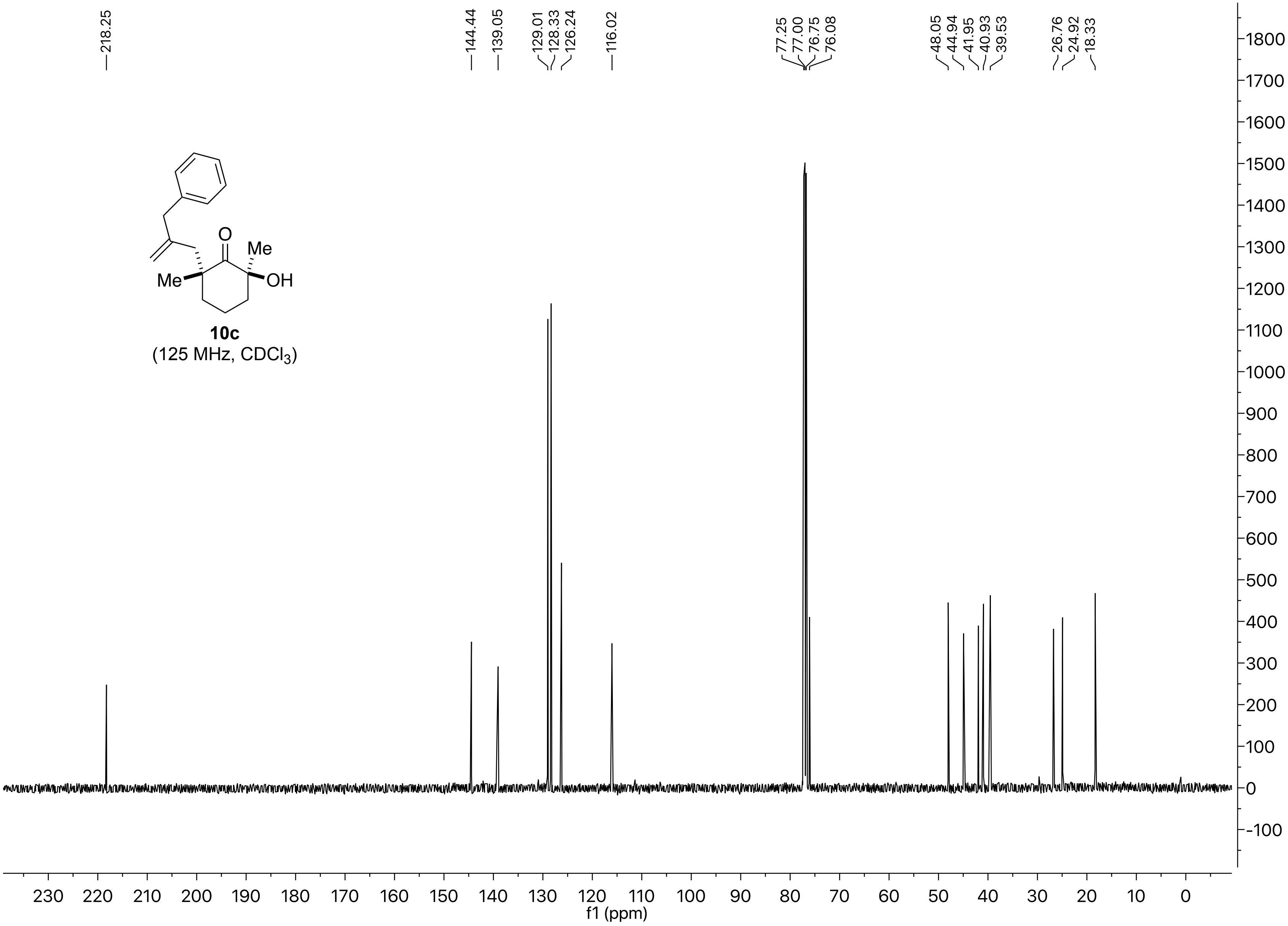

218.25
144.44
139.05
129.01
128.33
126.24
116.02
77.25
77.00
76.75
76.08
48.05
44.94
41.95
40.93
39.53
26.76
24.92
18.33
O
Me
Me
OH
10c
(125 MHz, CDCl3)
1800
1700
1600
1500
1400
1300
1200
1100
1000
900
800
700
600
500
400
300
200
100
0
-100
230 220 210 200 190 180 170 160 150 140 130 120 110 100 90 80 70 60 50 40 30 20 10 0
f1 (ppm)

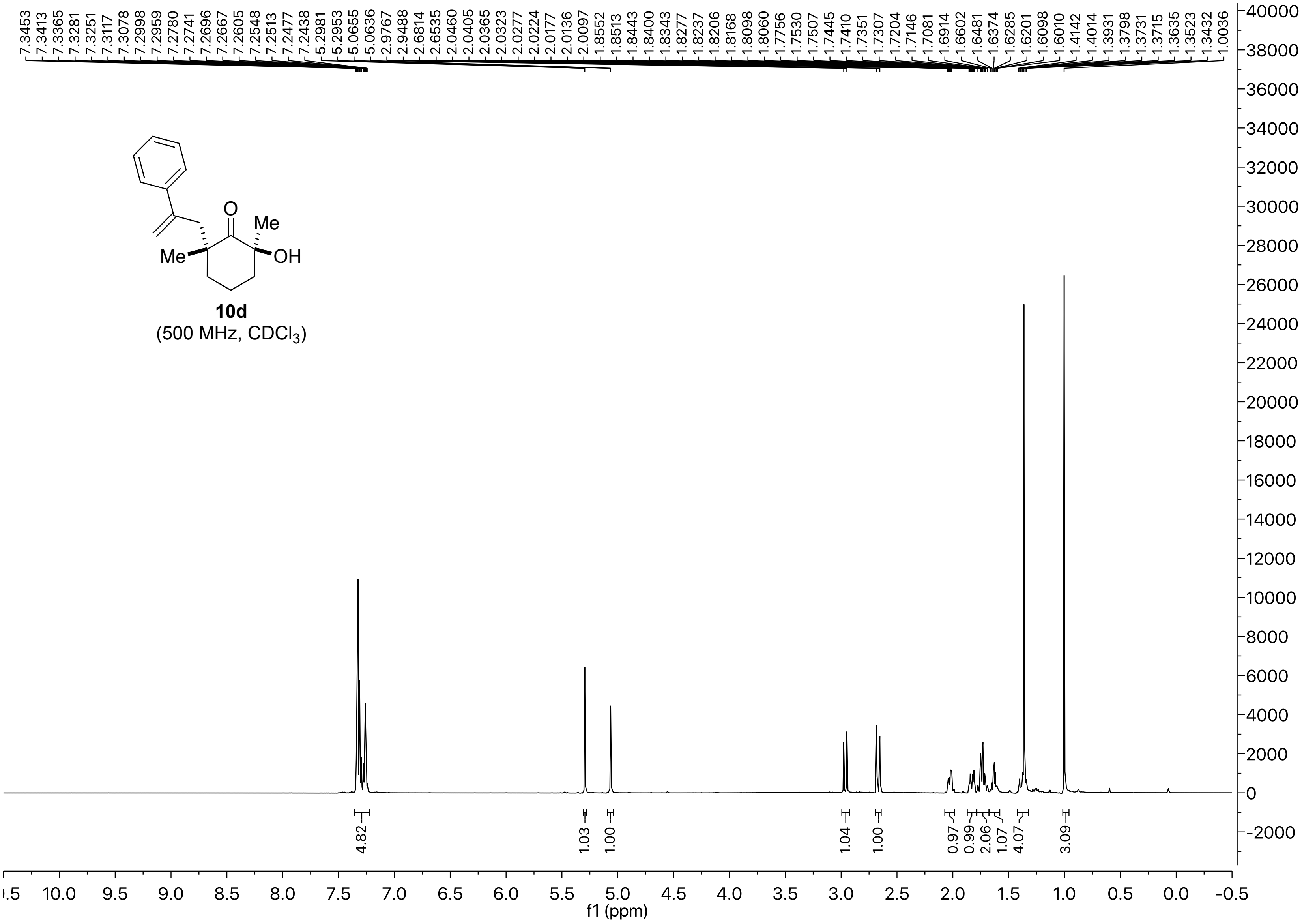

O
Me
OH
Me
10d
(500 MHz, CDCl3)
f1 (ppm)

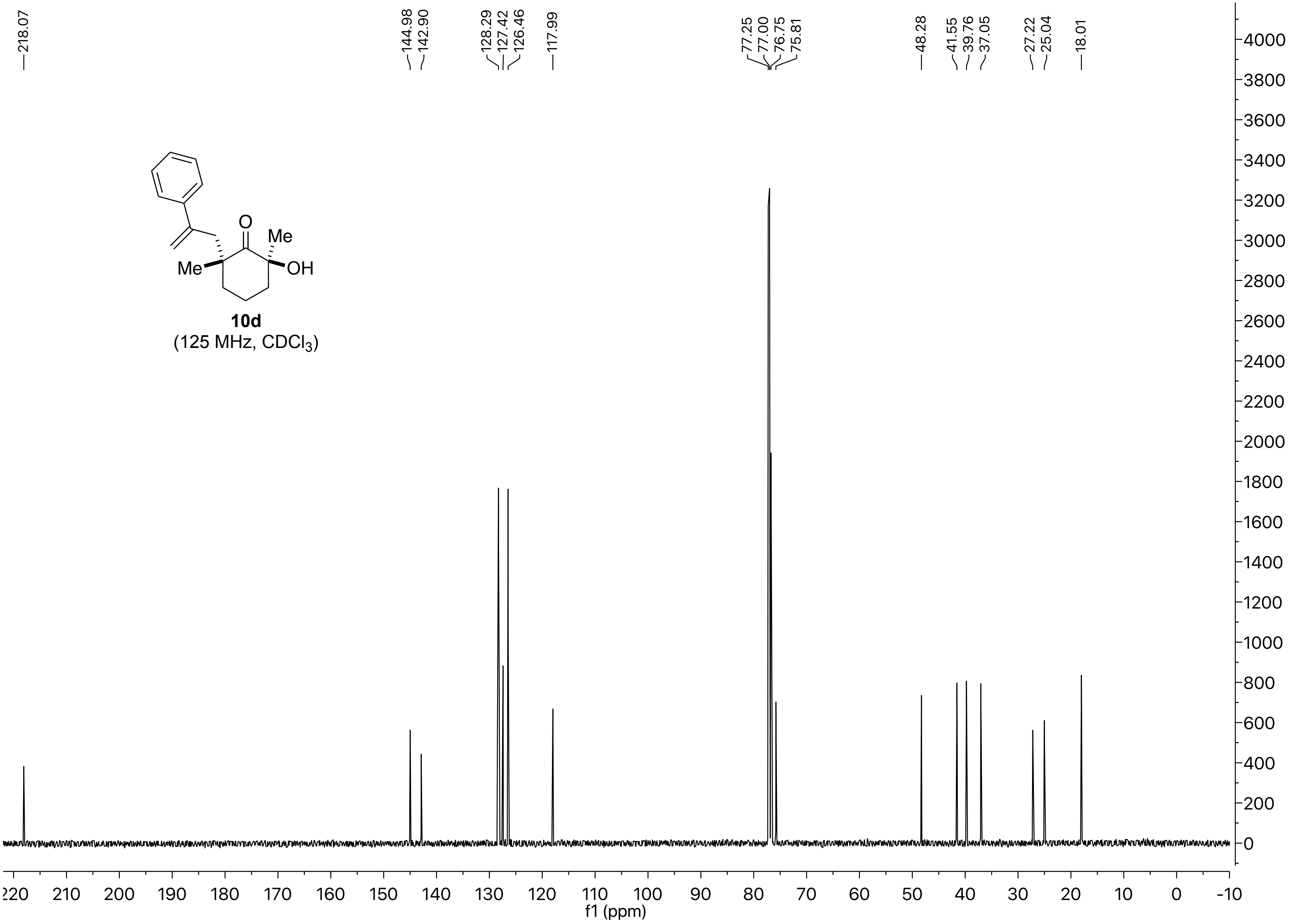
218.07
144.98
142.90
128.29
127.42
126.46
117.99
77.25
77.00
76.75
75.81
48.28
41.55
39.76
37.05
27.22
25.04
18.01
O
Me
Me
OH
10d
(125 MHz, CDCl3)
f1 (ppm)

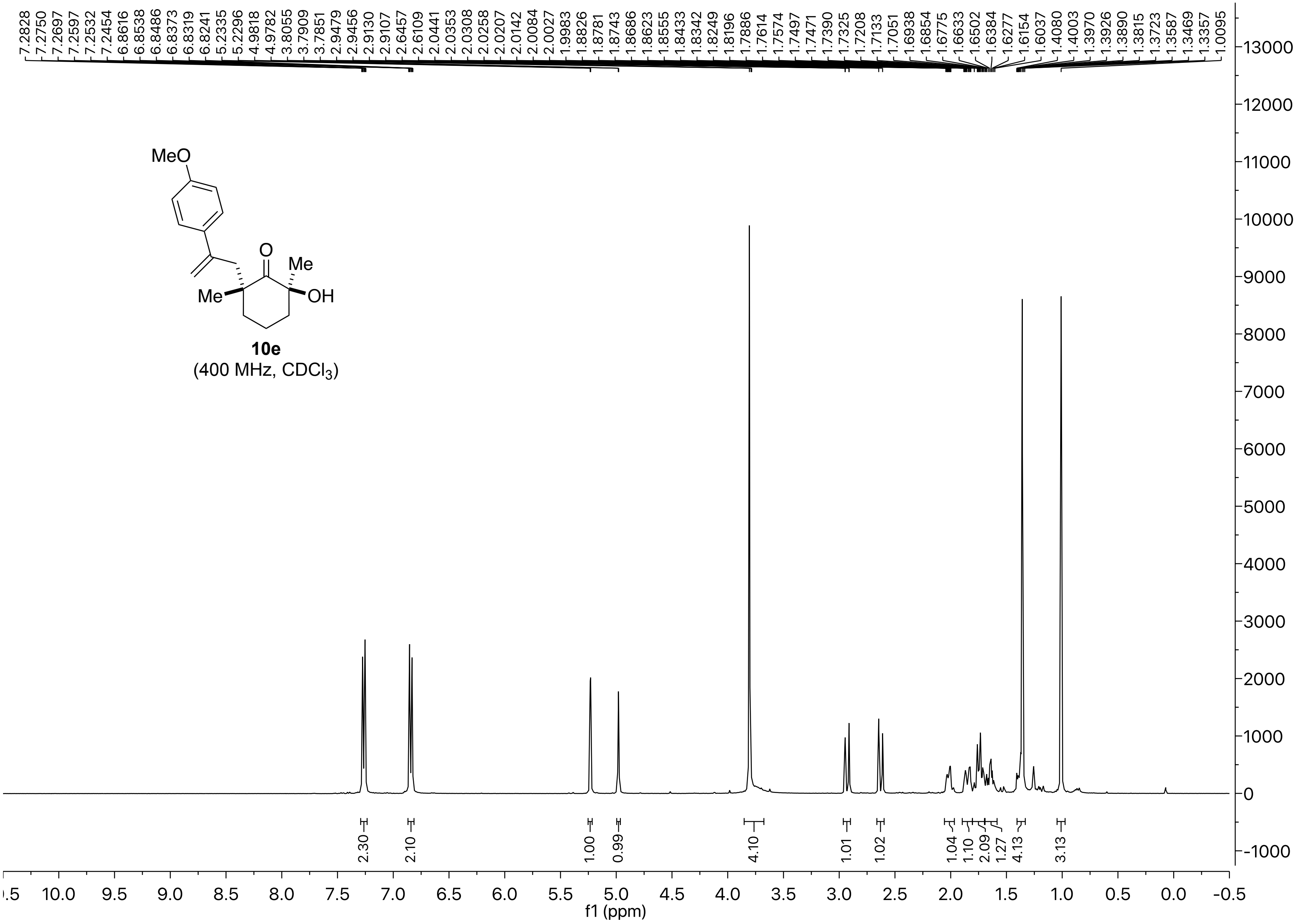
MeO
O
Me
Me
OH
10e
(400 MHz, CDCl3)
f1 (ppm)

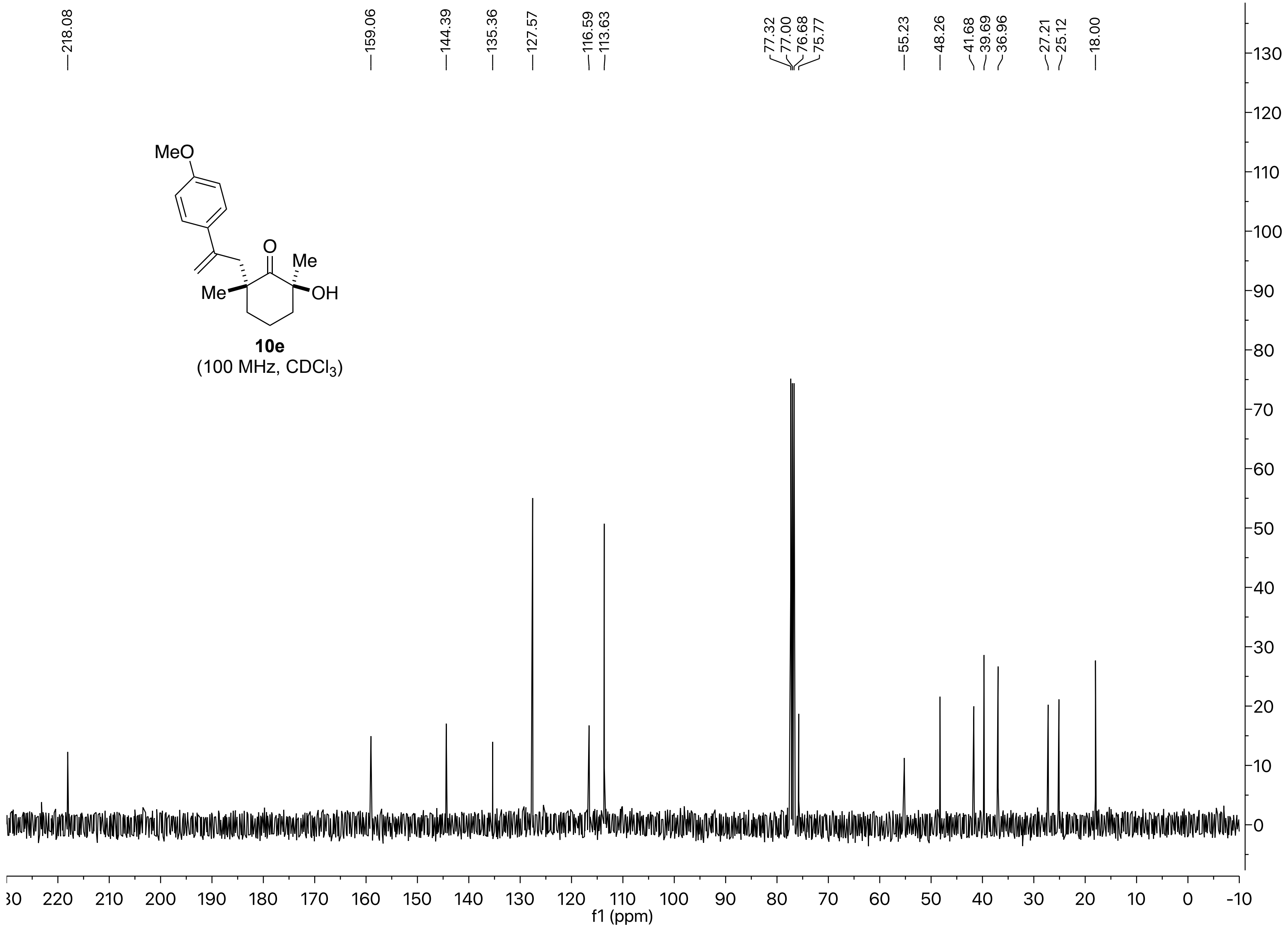

218.08
159.06
144.39
135.36
127.57
116.59
113.63
77.32
77.00
76.68
75.77
55.23
48.26
41.68
39.69
36.96
27.21
25.12
18.00
MeO
O
Me
Me
OH
10e
(100 MHz, CDCl3)
f1 (ppm)

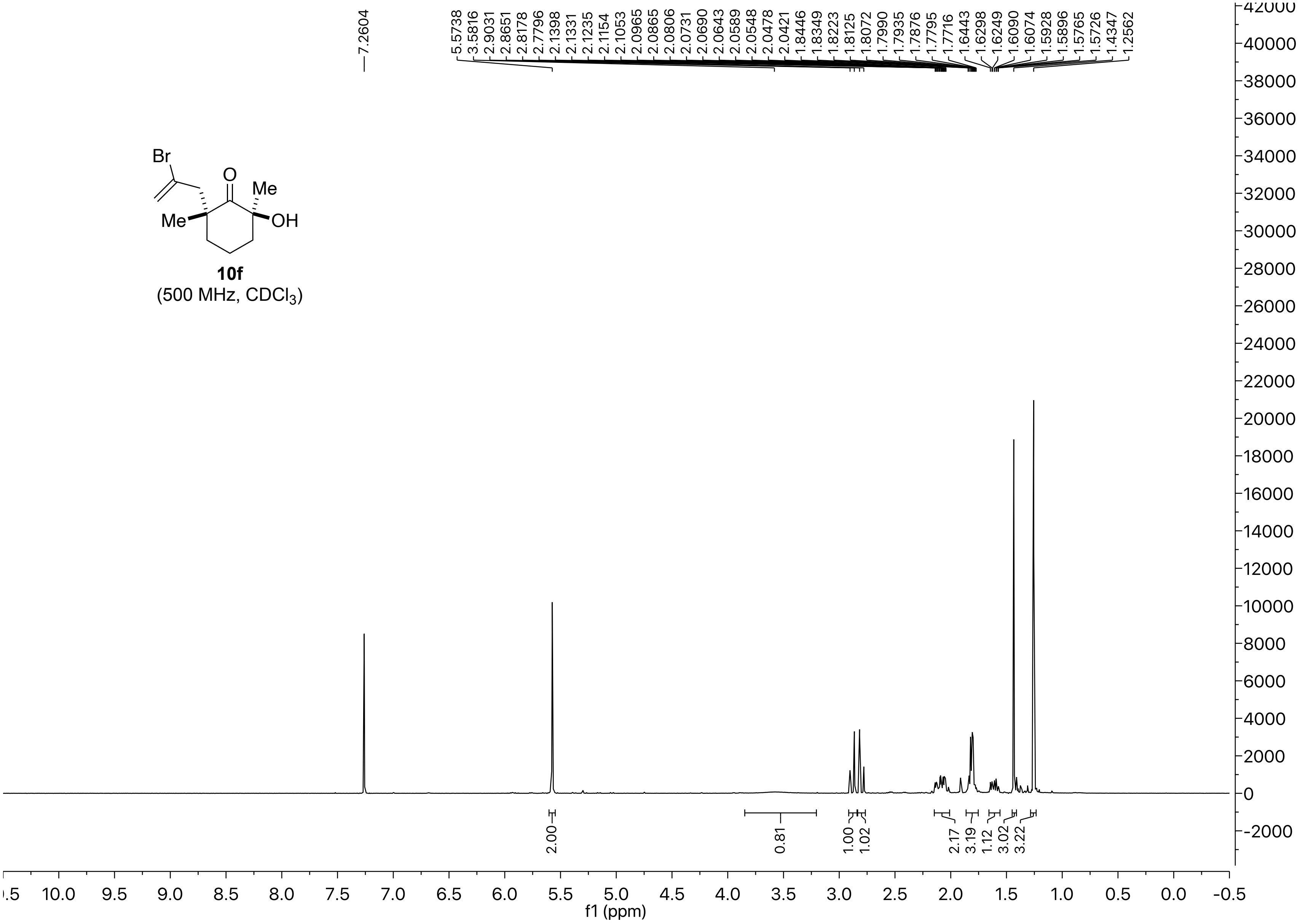

Br
O
Me
Me
OH
10f
(500 MHz, $CDCl_3$)
f1 (ppm)

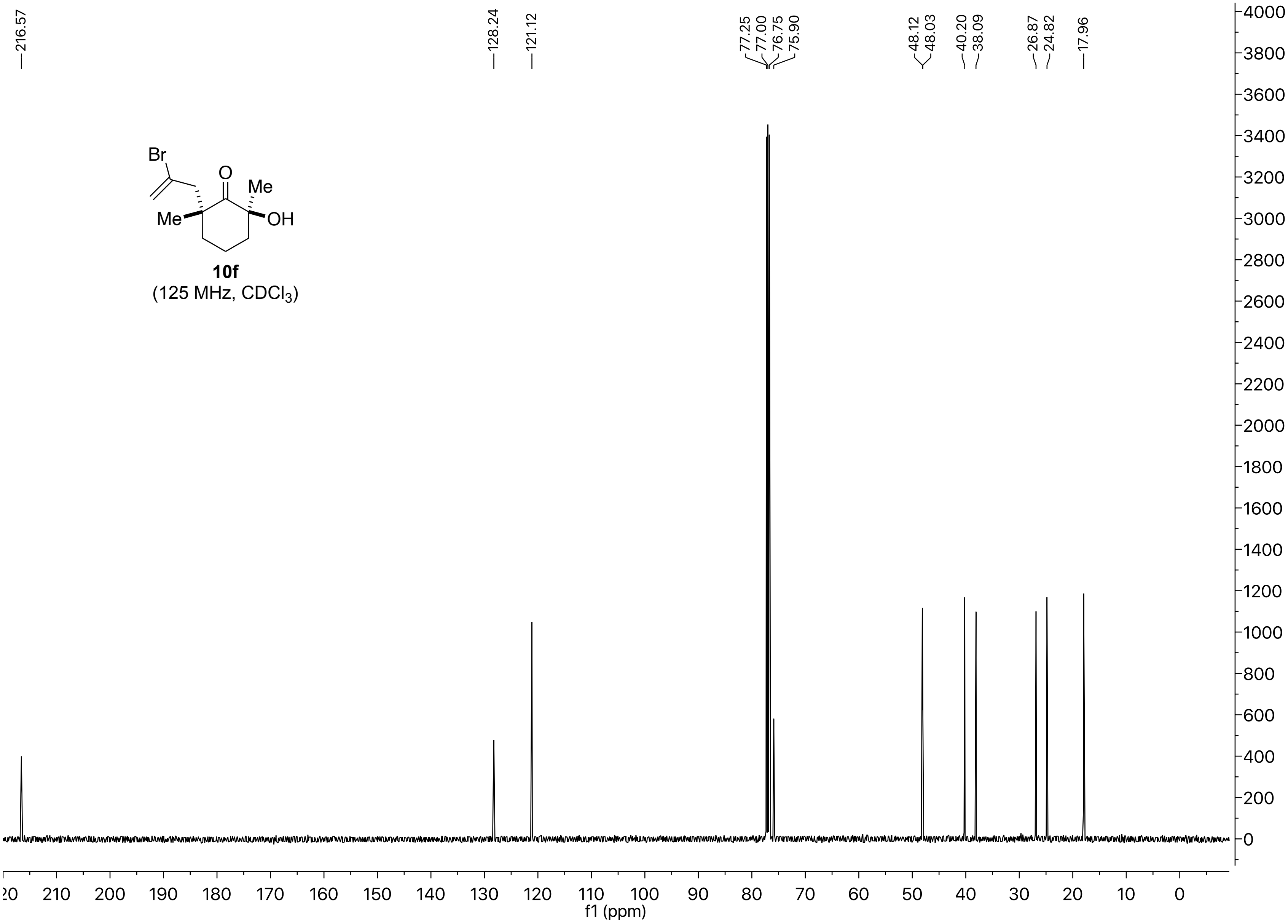
216.57
128.24
121.12
77.25
77.00
76.75
75.90
48.12
48.03
40.20
38.09
26.87
24.82
17.96
Br
O
Me
Me
OH
10f
(125 MHz, CDCl3)
f1 (ppm)

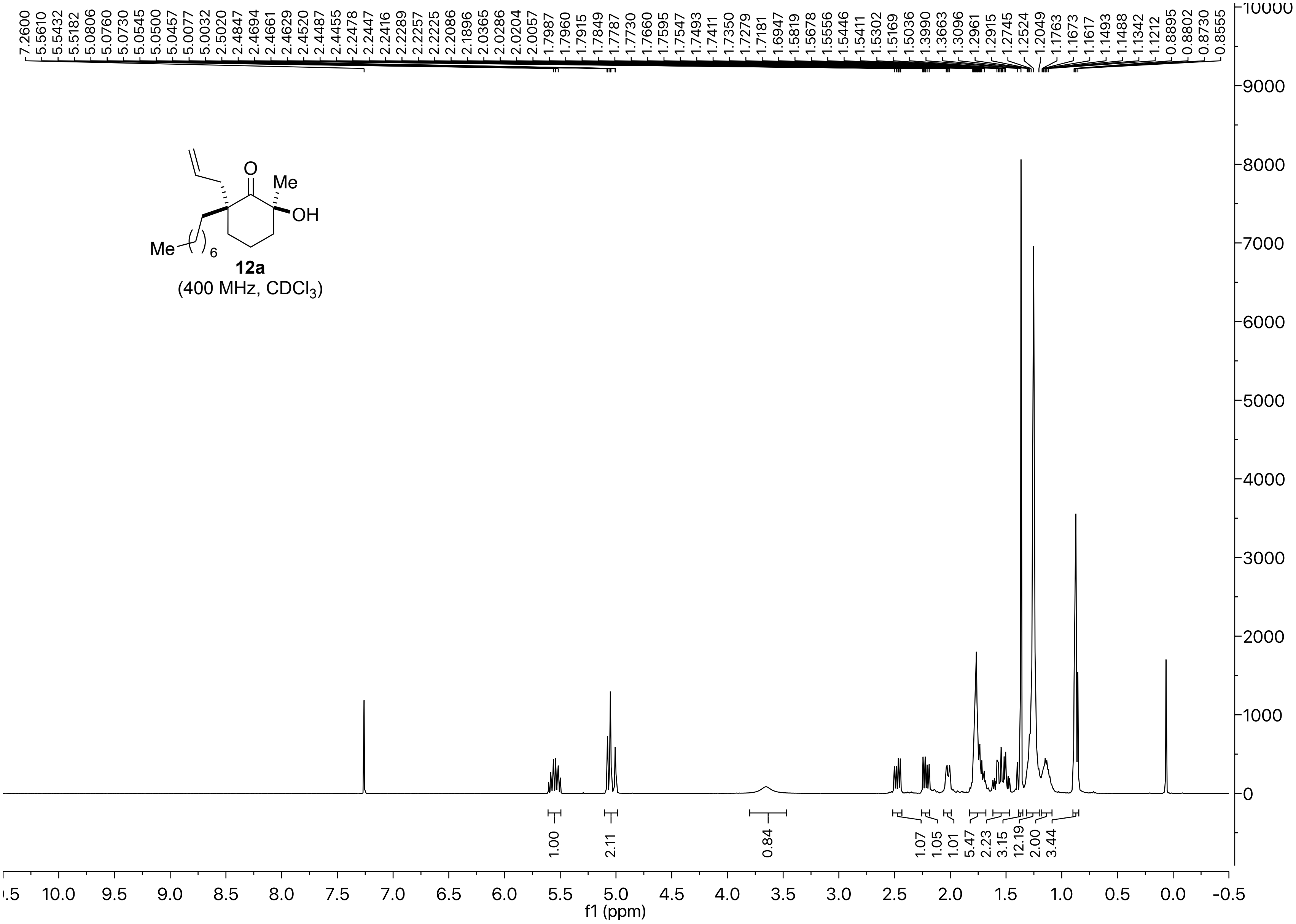
12a
(400 MHz, $CDCl_3$)
f1 (ppm)

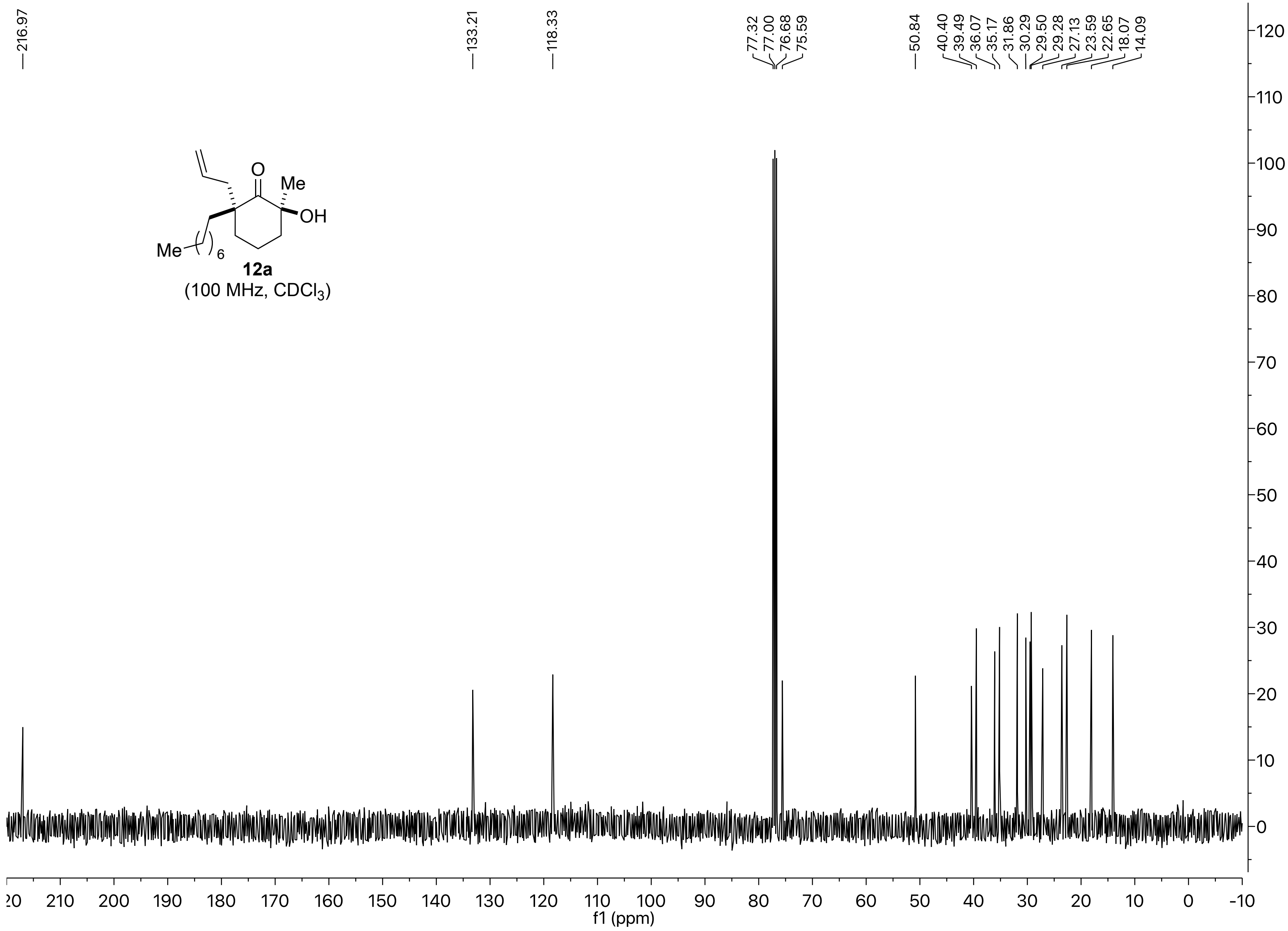
216.97
133.21
118.33
77.32
77.00
76.68
75.59
50.84
40.40
39.49
36.07
35.17
31.86
30.29
29.50
29.28
27.13
23.59
22.65
18.07
14.09
O
Me
OH
Me
6
12a
(100 MHz, $CDCl_3$)
f1 (ppm)

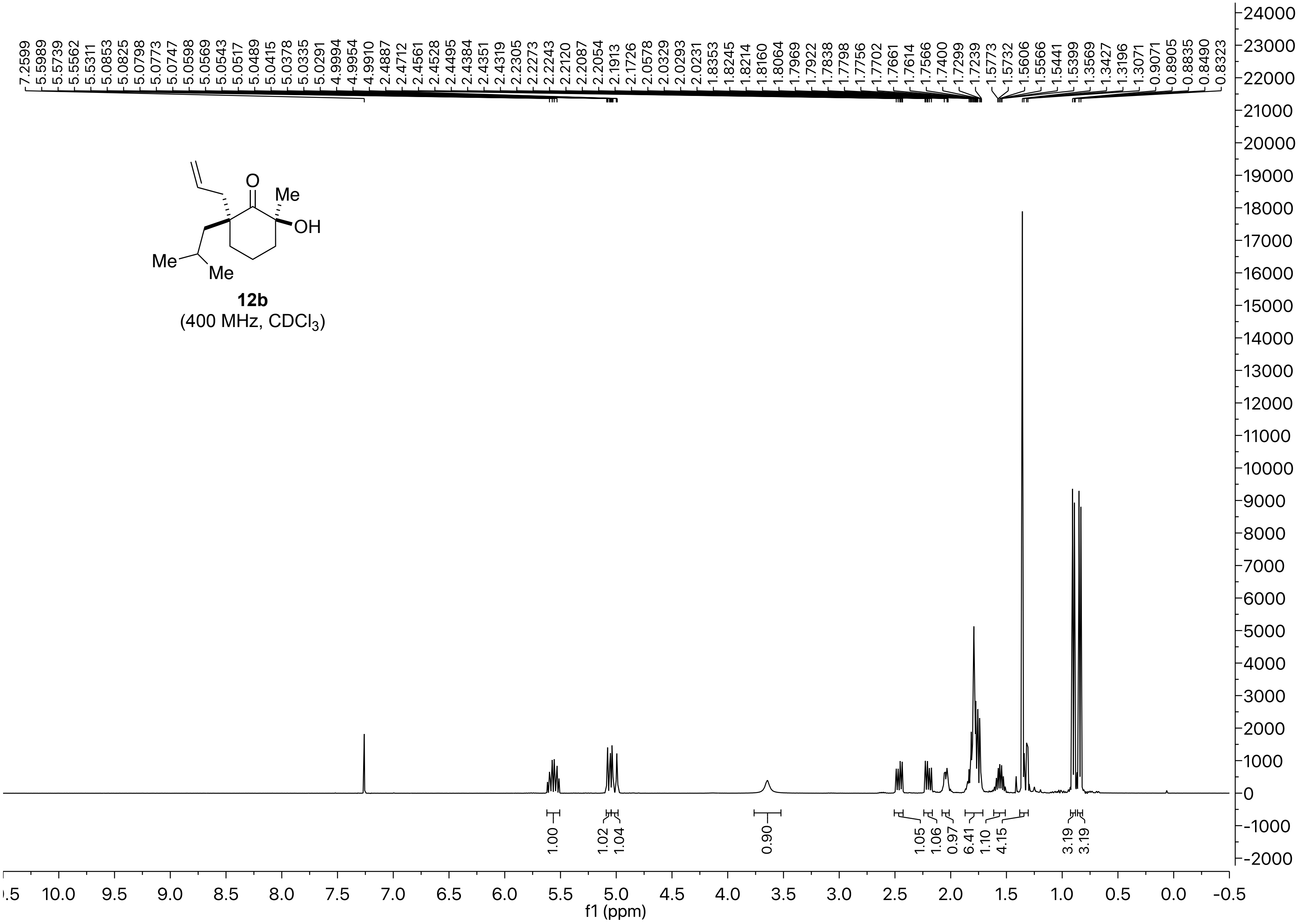
12b
(400 MHz, CDCl3)
f1 (ppm)

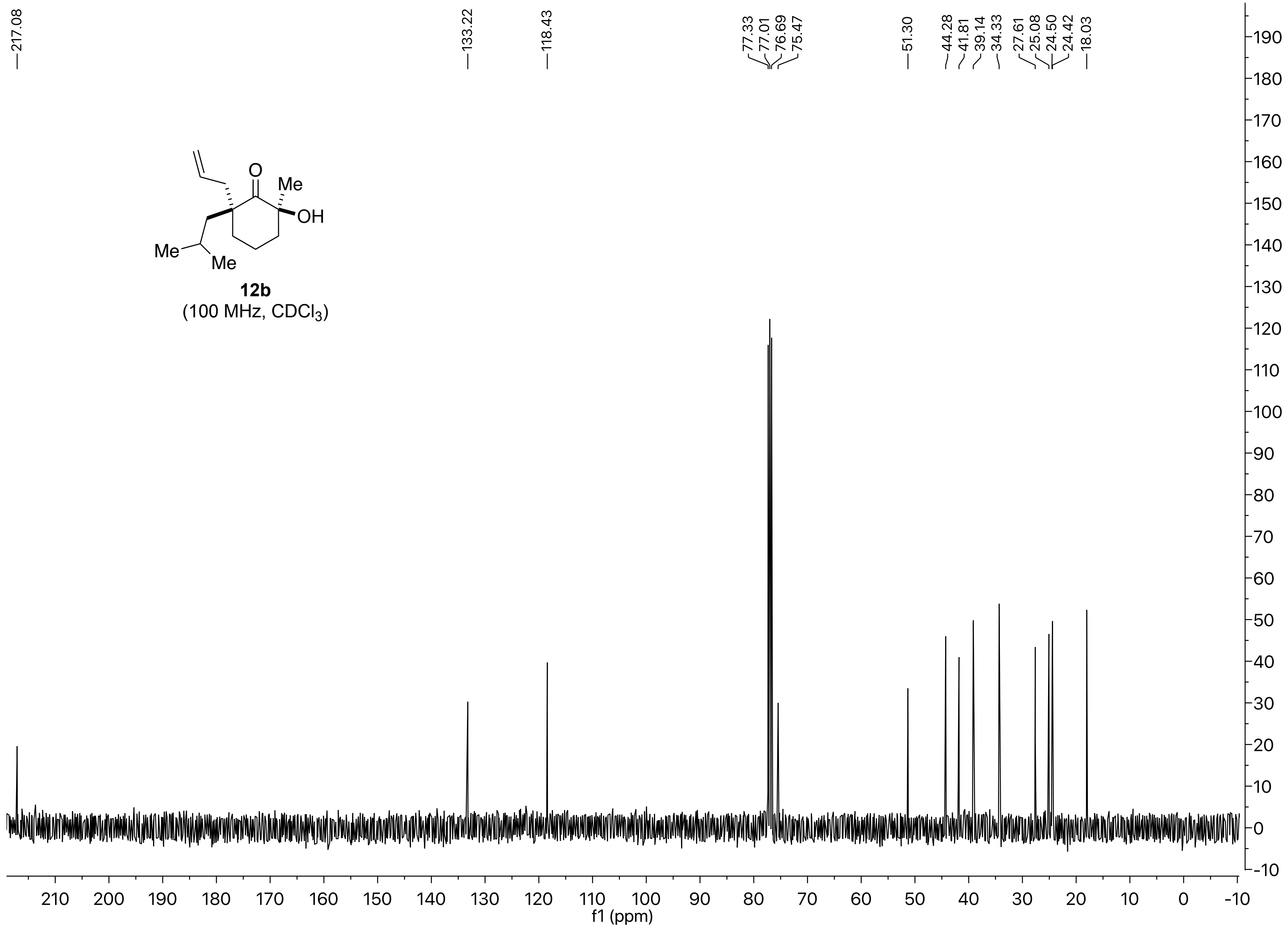
217.08
133.22
118.43
77.33
77.01
76.69
75.47
51.30
44.28
41.81
39.14
34.33
27.61
25.08
24.50
24.42
18.03
O
Me
OH
Me
Me
12b
(100 MHz, CDCl3)
f1 (ppm)

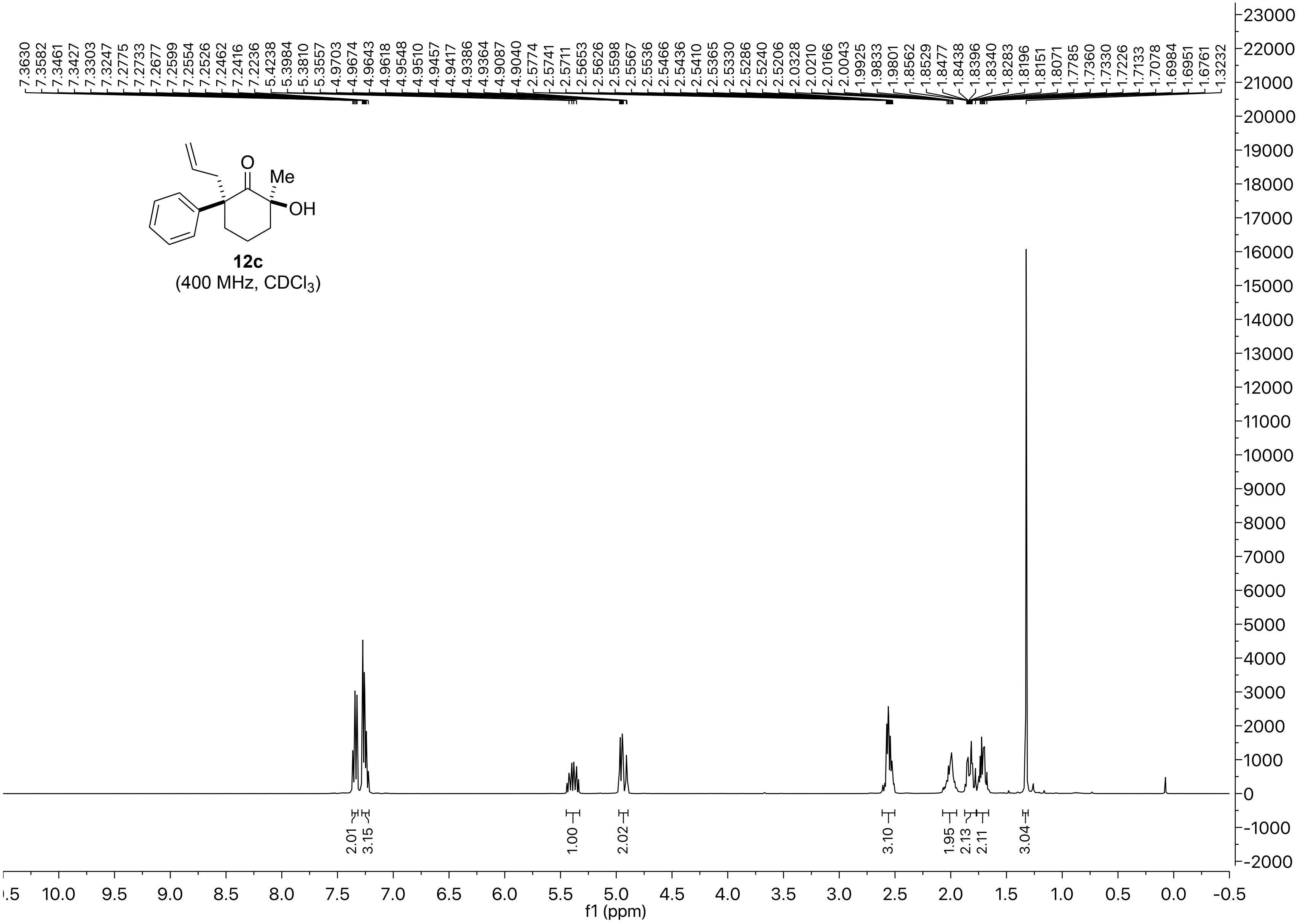

7.3630
7.3582
7.3461
7.3427
7.3303
7.3247
7.2775
7.2733
7.2677
7.2599
7.2554
7.2526
7.2462
7.2416
7.2236
5.4238
5.3984
5.3810
5.3557
4.9703
4.9674
4.9643
4.9618
4.9548
4.9510
4.9457
4.9417
4.9386
4.9364
4.9087
4.9040
2.5774
2.5741
2.5711
2.5653
2.5626
2.5598
2.5567
2.5536
2.5466
2.5436
2.5410
2.5365
2.5330
2.5286
2.5240
2.5206
2.0328
2.0210
2.0166
2.0043
1.9925
1.9833
1.9801
1.8562
1.8529
1.8477
1.8438
1.8396
1.8340
1.8283
1.8196
1.8151
1.8071
1.7785
1.7360
1.7330
1.7226
1.7133
1.7078
1.6984
1.6951
1.6761
1.3232
O
Me
OH
12c
(400 MHz, CDCl3)
2.01
3.15
1.00
2.02
3.10
1.95
2.13
2.11
3.04
f1 (ppm)

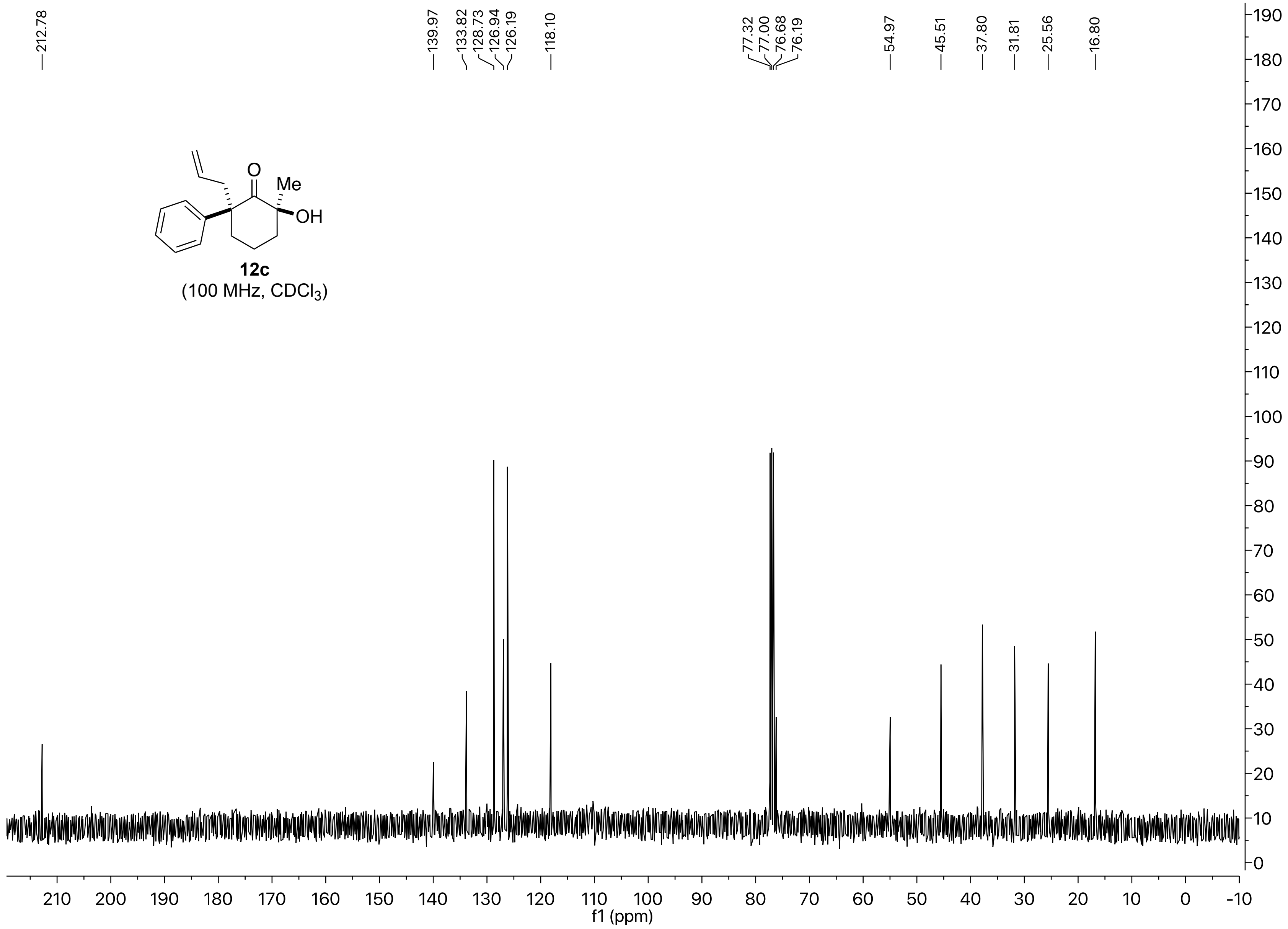
212.78
139.97
133.82
128.73
126.94
126.19
118.10
77.32
77.00
76.68
76.19
54.97
45.51
37.80
31.81
25.56
16.80
O
Me
OH
12c
(100 MHz, CDCl3)
f1 (ppm)

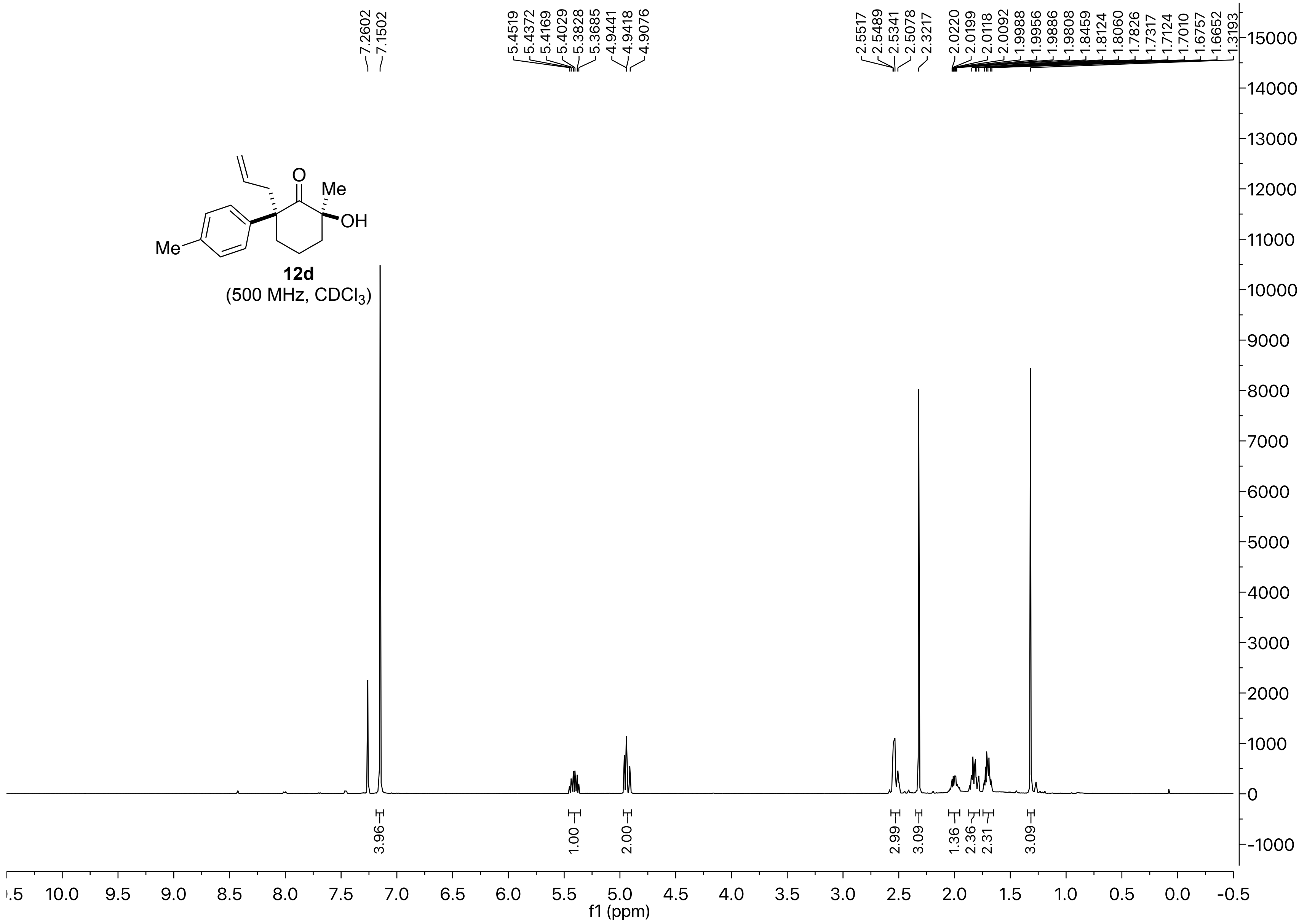
Me
O
Me
OH
12d
(500 MHz, CDCl3)
f1 (ppm)

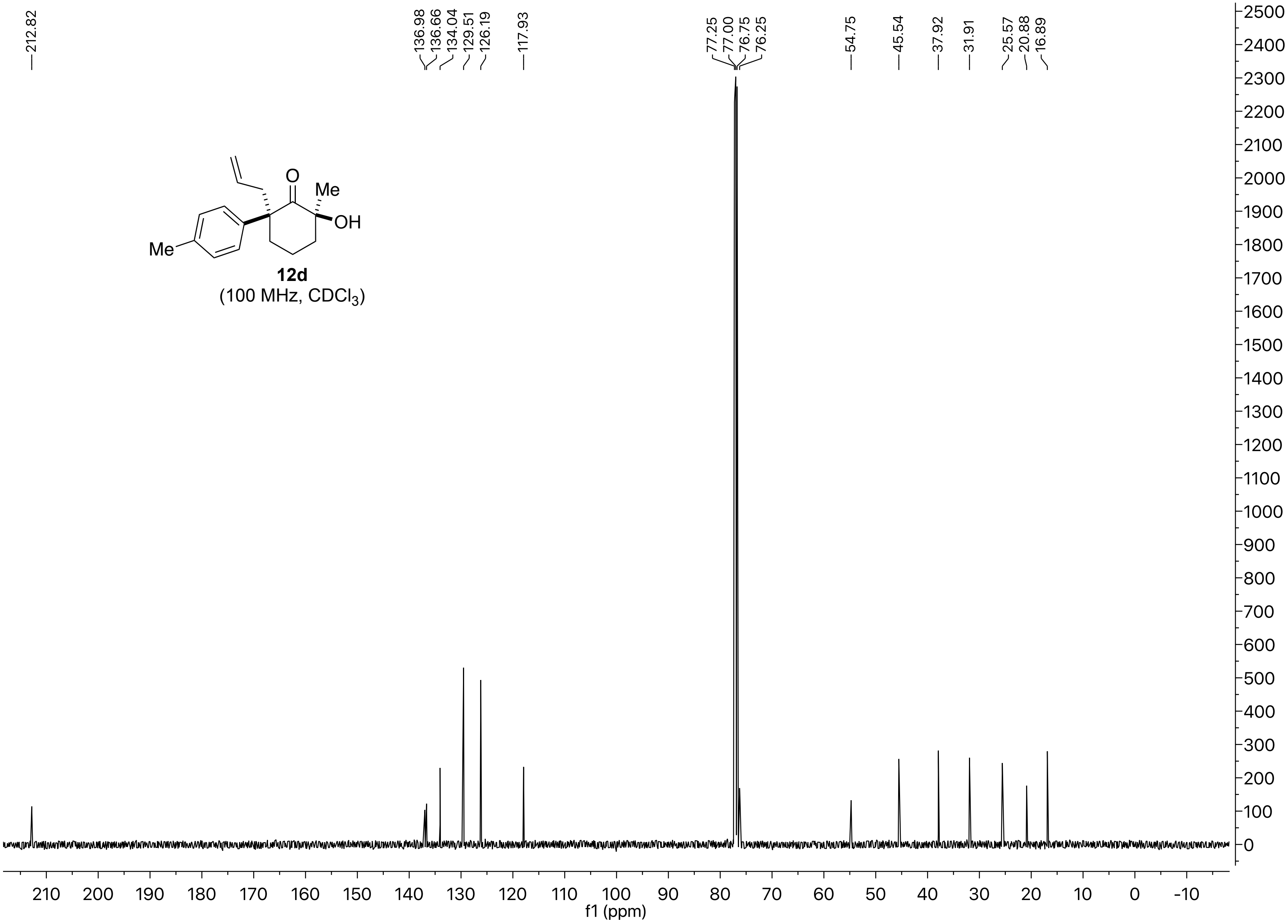
212.82
136.98
136.66
134.04
129.51
126.19
117.93
77.25
77.00
76.75
76.25
54.75
45.54
37.92
31.91
25.57
20.88
16.89
O
Me
OH
Me
12d
(100 MHz, CDCl3)
f1 (ppm)

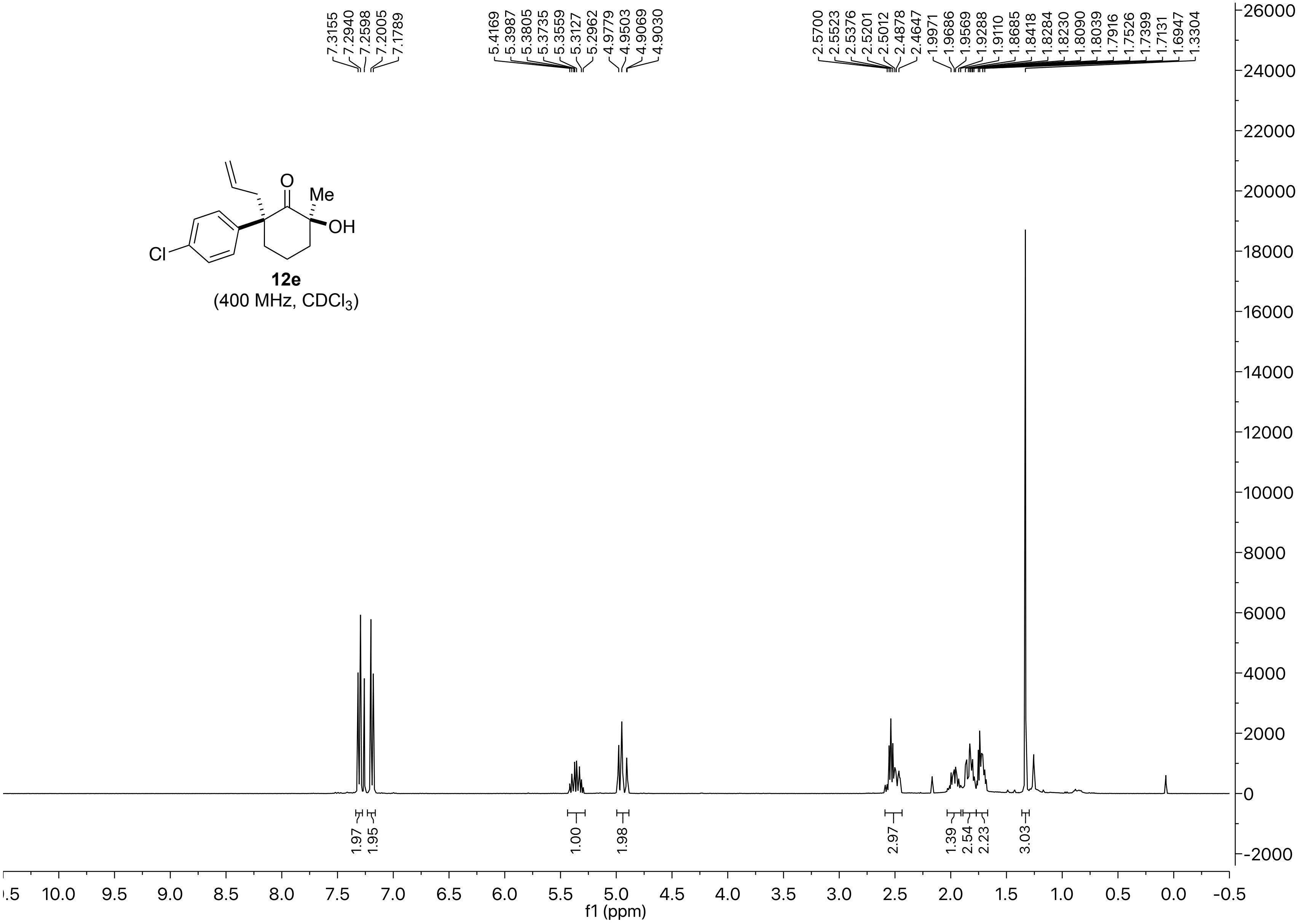

7.3155
7.2940
7.2598
7.2005
7.1789
5.4169
5.3987
5.3805
5.3735
5.3559
5.3127
5.2962
4.9779
4.9503
4.9069
4.9030
2.5700
2.5523
2.5376
2.5201
2.5012
2.4878
2.4647
1.9971
1.9686
1.9569
1.9288
1.9110
1.8685
1.8418
1.8284
1.8230
1.8090
1.8039
1.7916
1.7526
1.7399
1.7131
1.6947
1.3304
O
Me
OH
Cl
12e
(400 MHz, CDCl3)
1.97
1.95
1.00
1.98
2.97
1.39
2.54
2.23
3.03
f1 (ppm)

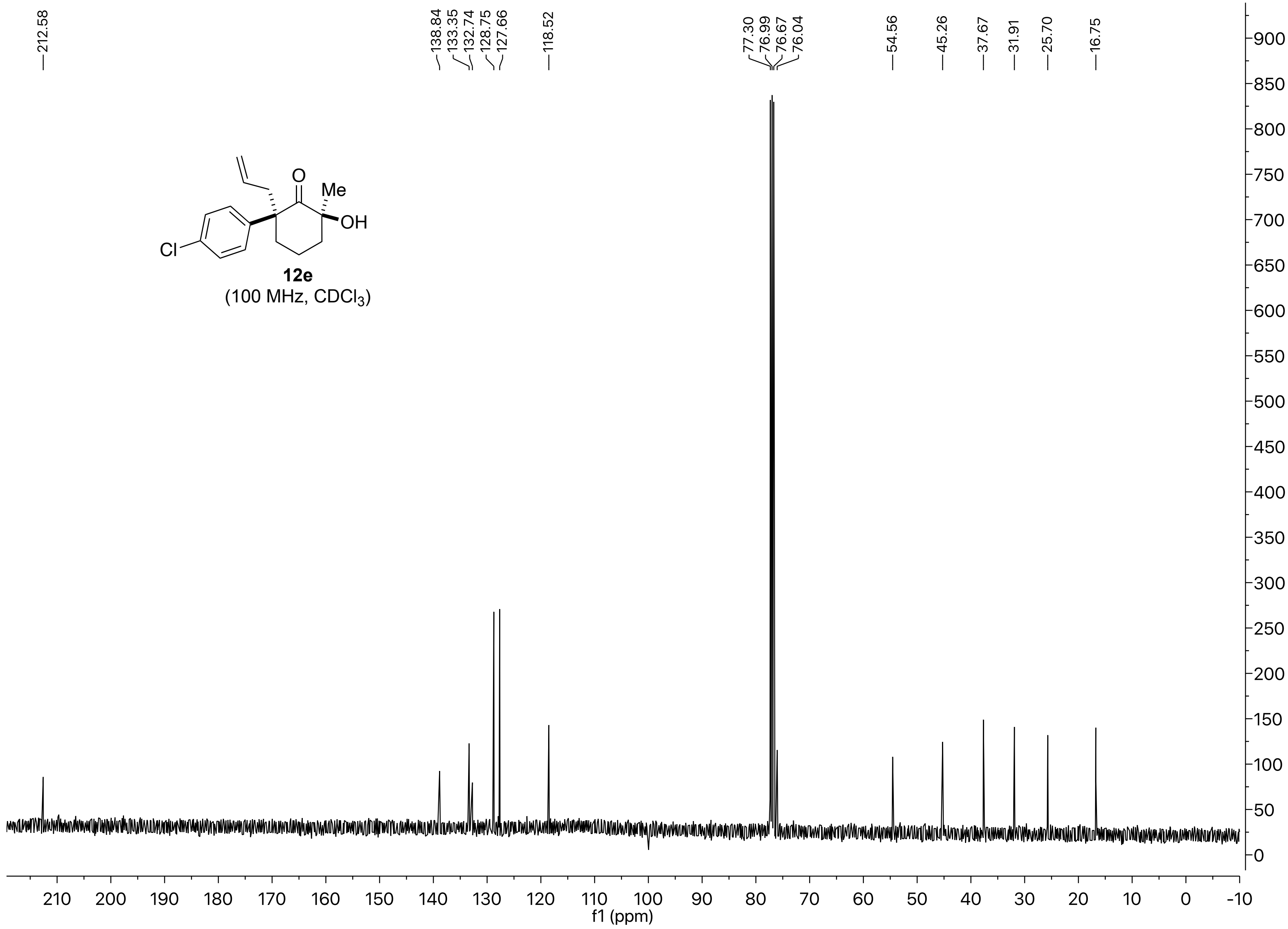
212.58
138.84
133.35
132.74
128.75
127.66
118.52
77.30
76.99
76.67
76.04
54.56
45.26
37.67
31.91
25.70
16.75
O
Me
OH
Cl
12e
(100 MHz, CDCl3)
f1 (ppm)

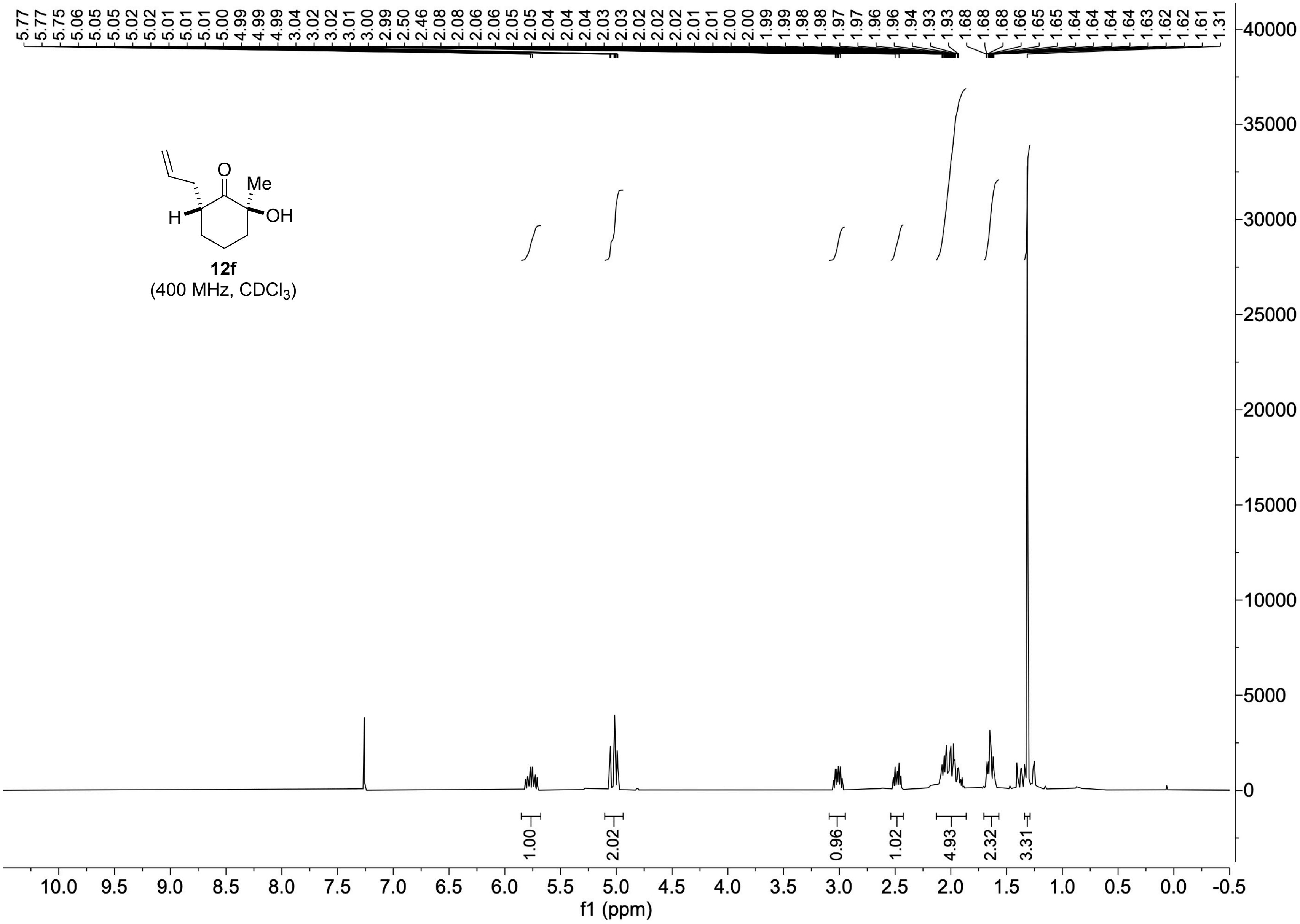

12f
(400 MHz, CDCl3)
f1 (ppm)

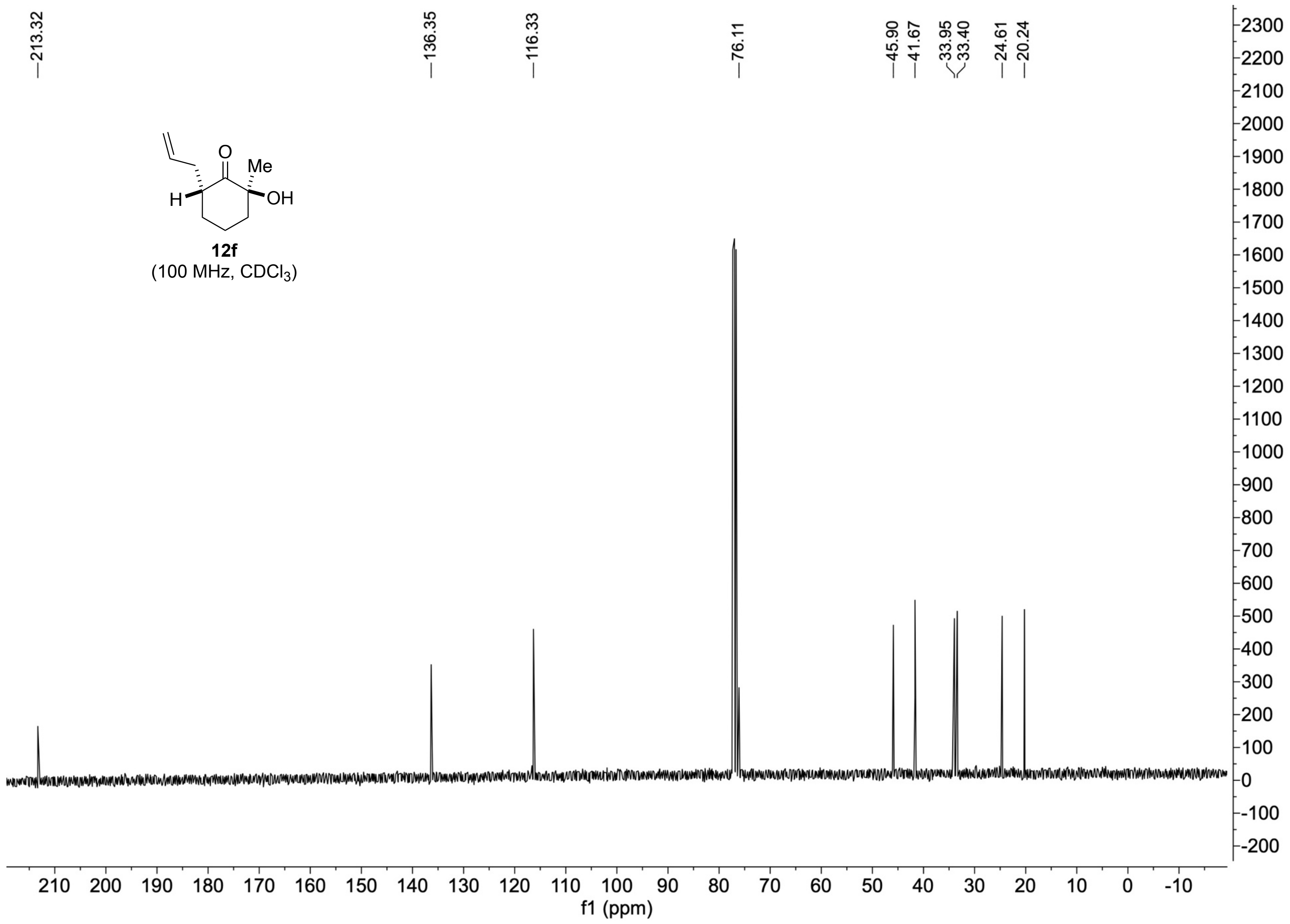
213.32
136.35
116.33
76.11
45.90
41.67
33.95
33.40
24.61
20.24
O
Me
H
OH
12f
(100 MHz, CDCl3)
f1 (ppm)

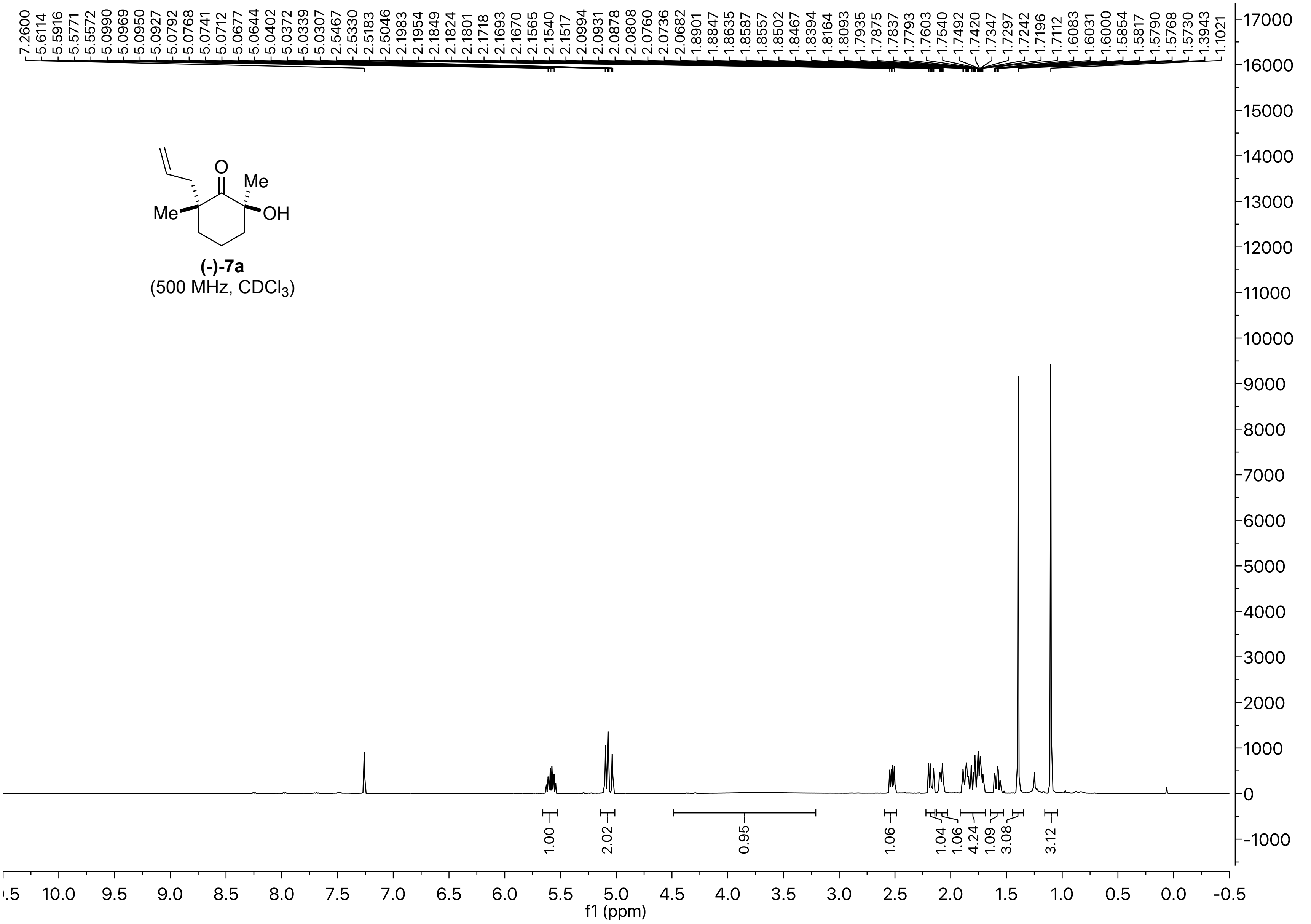

O
Me
Me
OH
(-)-7a
(500 MHz, CDCl3)
7.2600
5.6114
5.5916
5.5771
5.5572
5.0990
5.0969
5.0950
5.0927
5.0792
5.0768
5.0741
5.0712
5.0677
5.0644
5.0402
5.0372
5.0339
5.0307
2.5467
2.5330
2.5183
2.5046
2.1983
2.1954
2.1849
2.1824
2.1801
2.1718
2.1693
2.1670
2.1565
2.1540
2.1517
2.0994
2.0931
2.0878
2.0808
2.0760
2.0736
2.0682
1.8901
1.8847
1.8635
1.8587
1.8557
1.8502
1.8467
1.8394
1.8164
1.8093
1.7935
1.7875
1.7837
1.7793
1.7603
1.7540
1.7492
1.7420
1.7347
1.7297
1.7242
1.7196
1.7112
1.6083
1.6031
1.6000
1.5854
1.5817
1.5790
1.5768
1.5730
1.3943
1.1021
1.00
2.02
0.95
1.06
1.04
1.06
4.24
1.09
3.08
3.12
f1 (ppm)

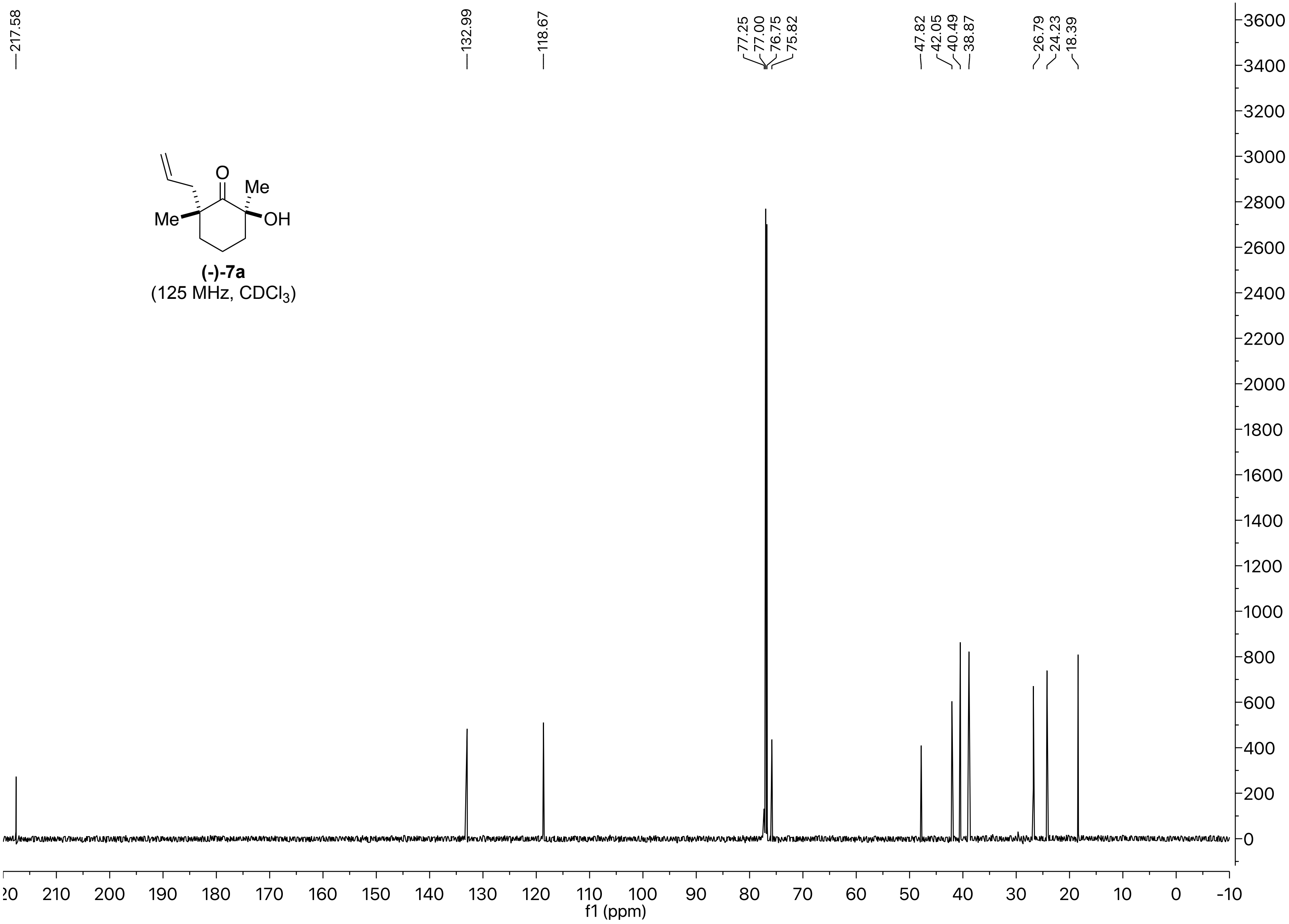
217.58
132.99
118.67
77.25
77.00
76.75
75.82
47.82
42.05
40.49
38.87
26.79
24.23
18.39
O
Me
Me
OH
(-)-7a
(125 MHz, CDCl3)
f1 (ppm)

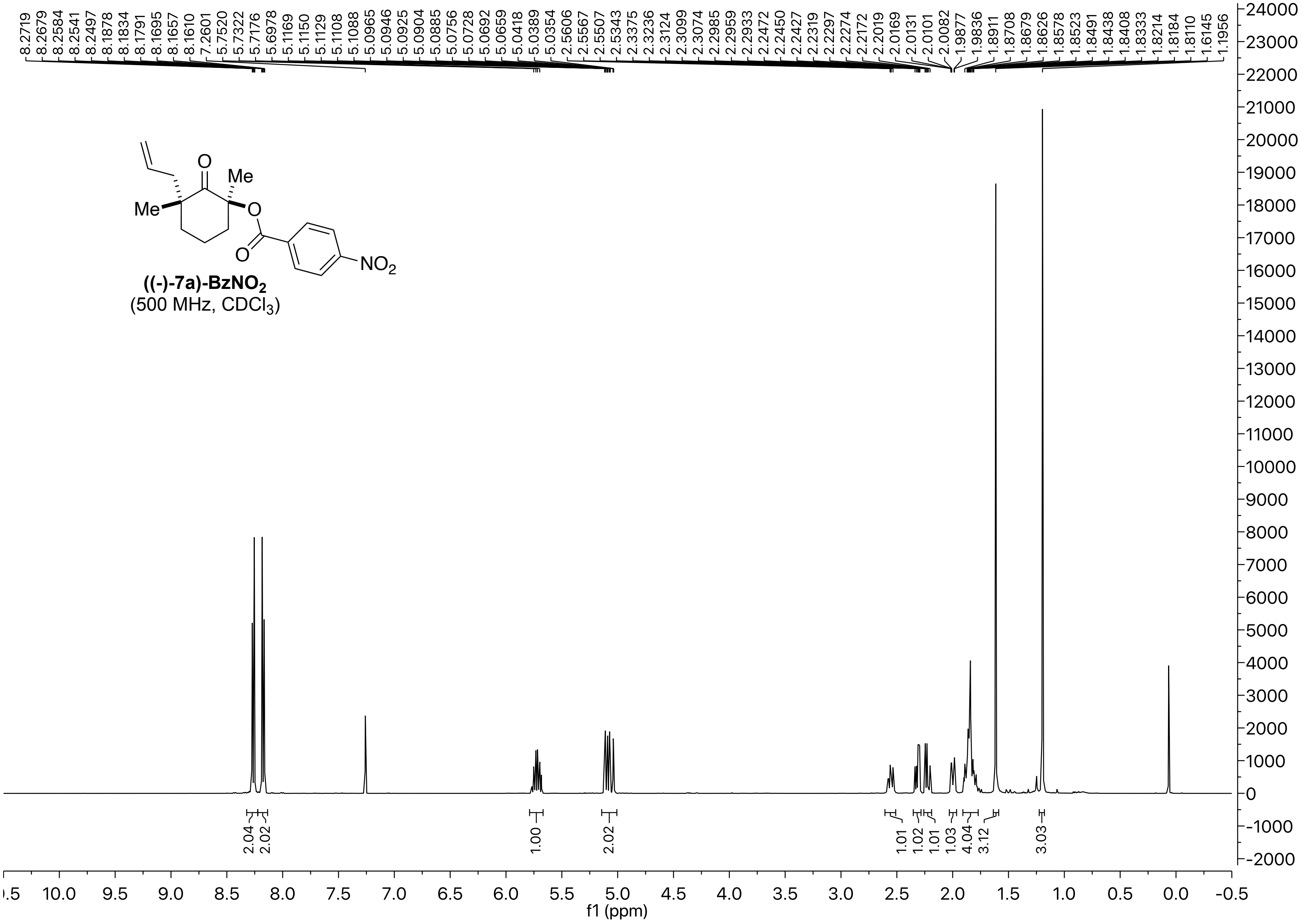
Me
O
Me
O
O
NO2
((-)-7a)-BzNO2
(500 MHz, CDCl3)
f1 (ppm)

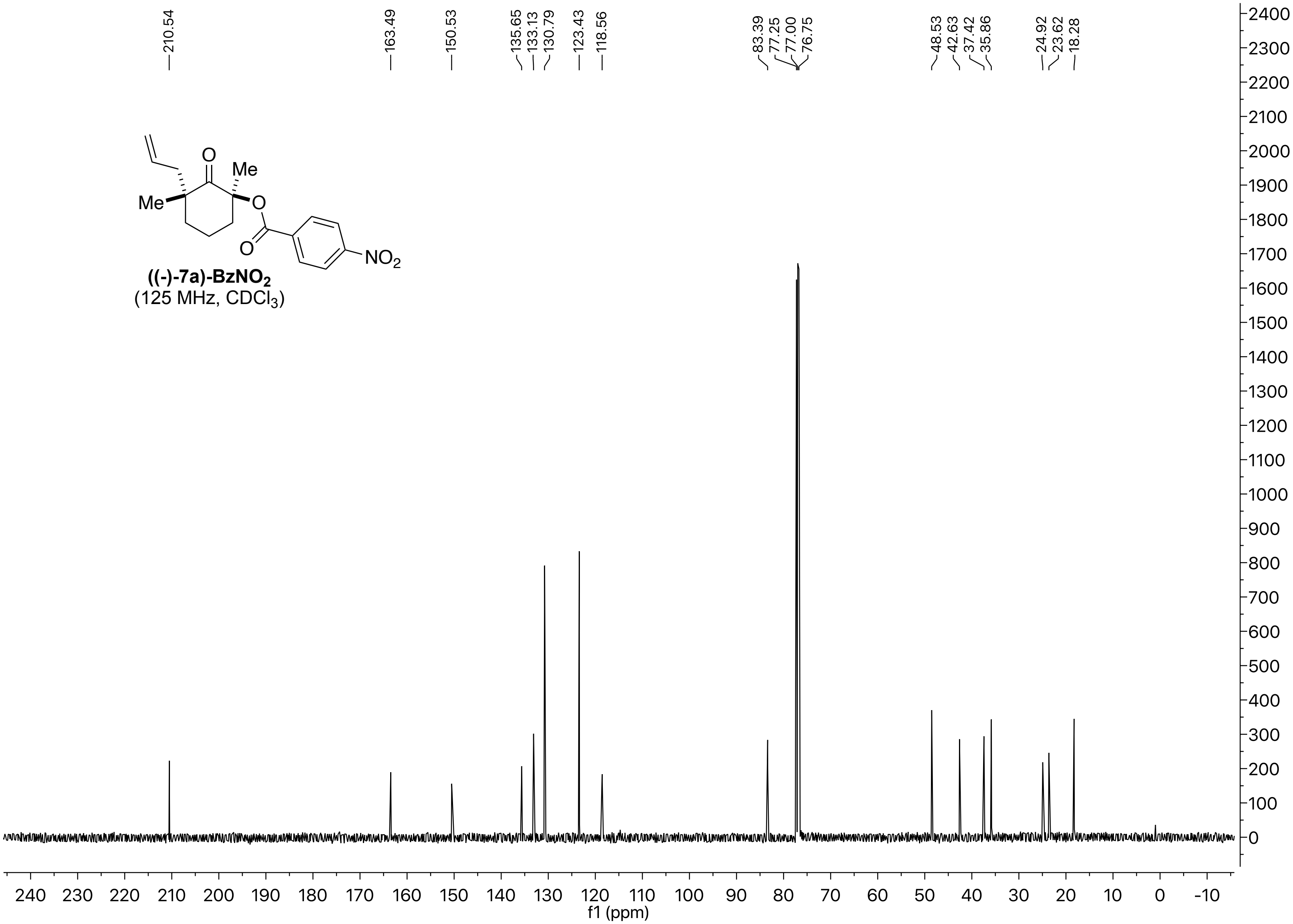

210.54
163.49
150.53
135.65
133.13
130.79
123.43
118.56
83.39
77.25
77.00
76.75
48.53
42.63
37.42
35.86
24.92
23.62
18.28
O
Me
Me
O
O
NO_2
((-)-7a)-BzNO$_2$
(125 MHz, CDCl$_3$)
f1 (ppm)

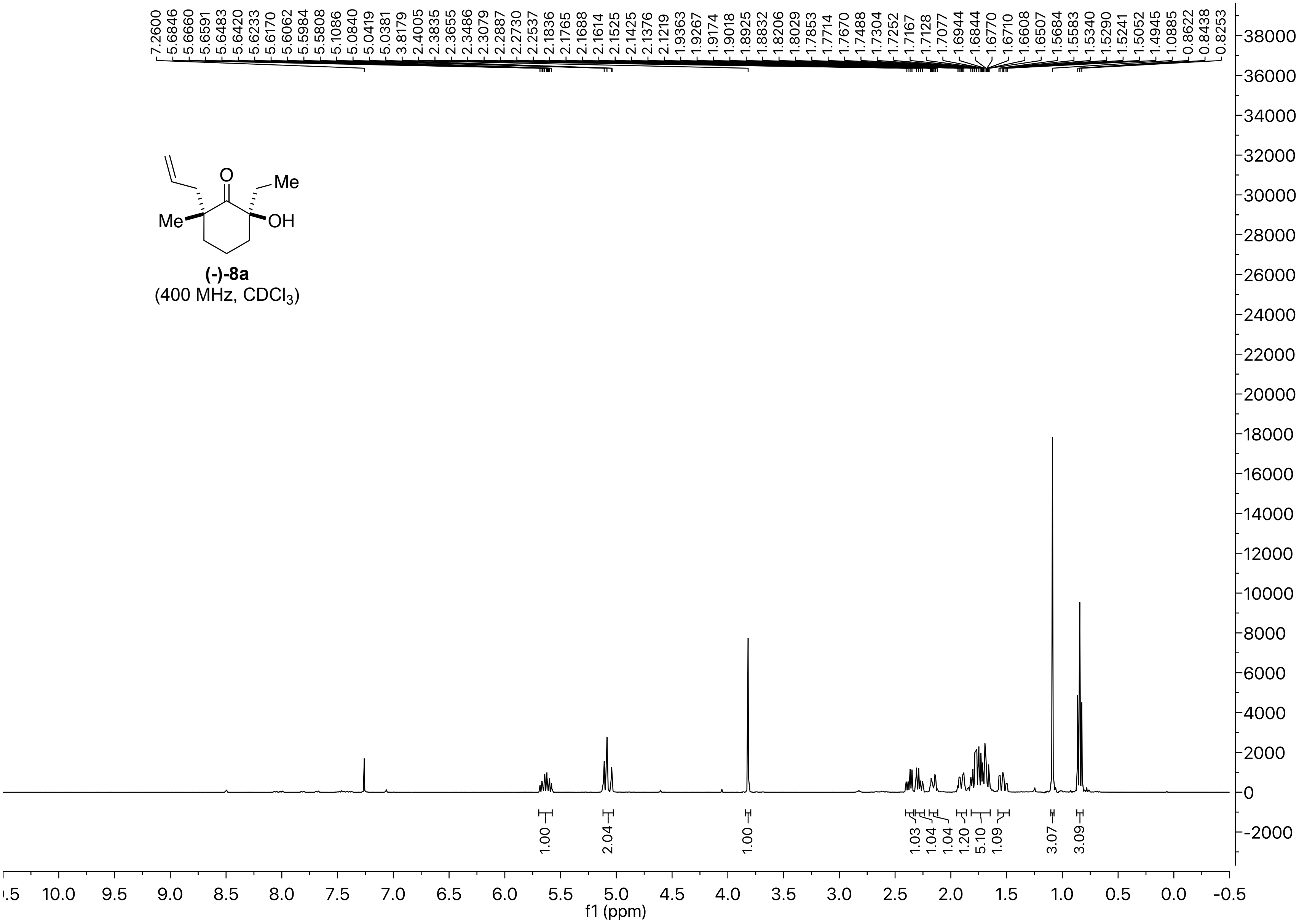
(-)-8a
(400 MHz, CDCl3)
f1 (ppm)

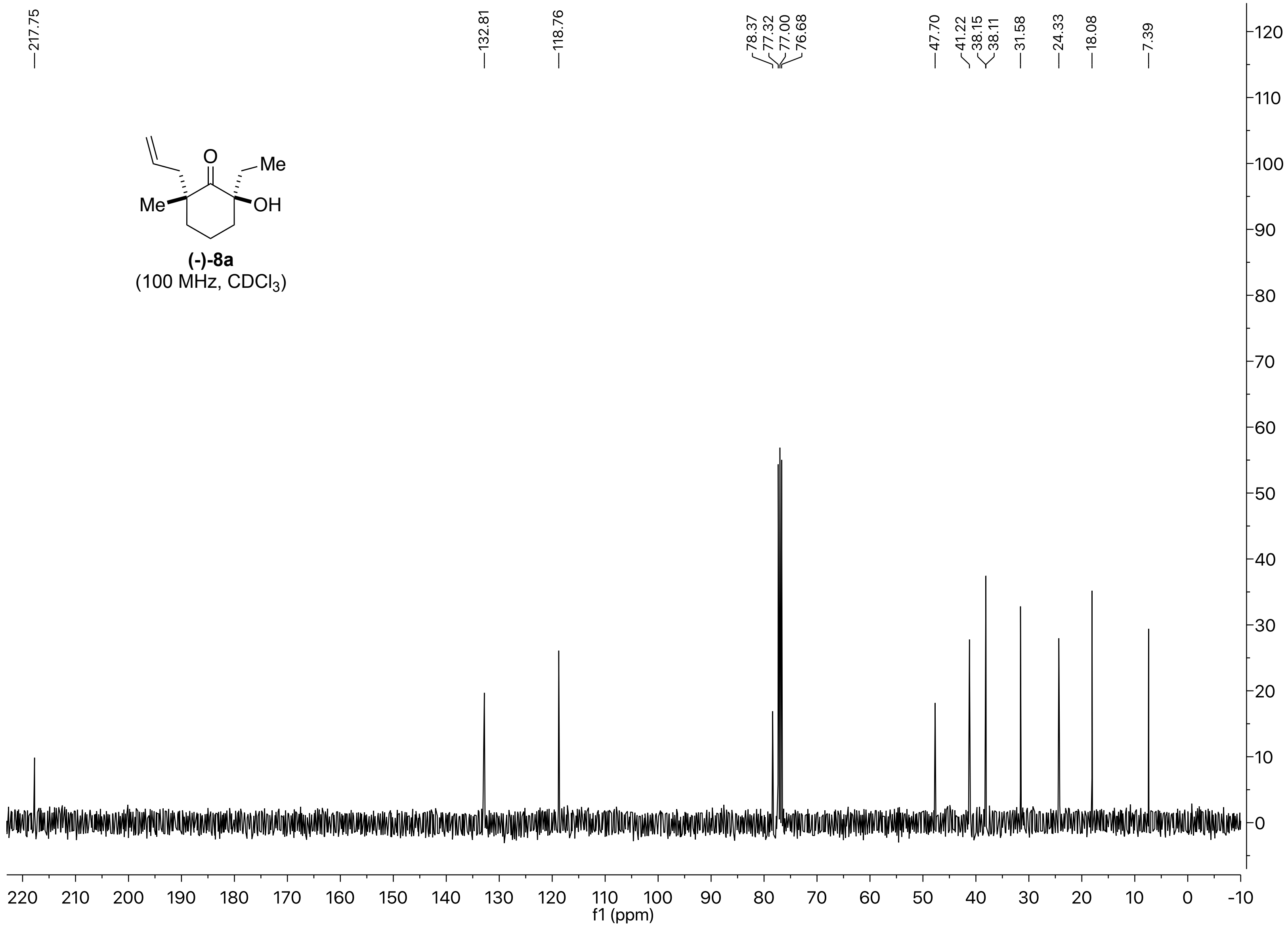

217.75
132.81
118.76
78.37
77.32
77.00
76.68
47.70
41.22
38.15
38.11
31.58
24.33
18.08
7.39
Me
OH
Me
O
(-)-8a
(100 MHz, CDCl3)
f1 (ppm)

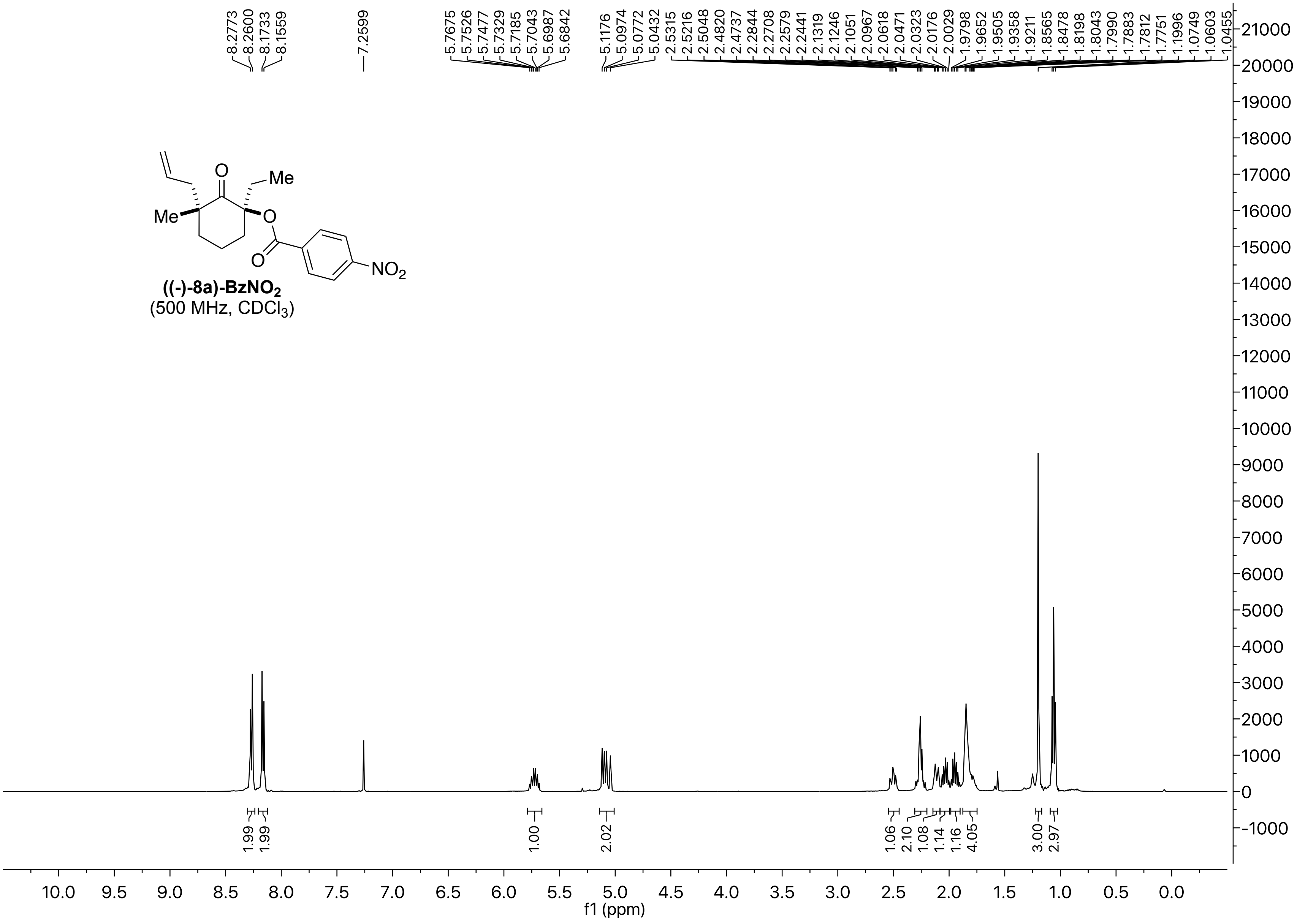
((-)-8a)-BzNO$_2$
(500 MHz, CDCl$_3$)
8.2773
8.2600
8.1733
8.1559
7.2599
5.7675
5.7526
5.7477
5.7329
5.7185
5.7043
5.6987
5.6842
5.1176
5.0974
5.0772
5.0432
2.5315
2.5216
2.5048
2.4820
2.4737
2.2844
2.2708
2.2579
2.2441
2.1319
2.1246
2.1051
2.0967
2.0618
2.0471
2.0323
2.0176
2.0029
1.9798
1.9652
1.9505
1.9358
1.9211
1.8565
1.8478
1.8198
1.8043
1.7990
1.7883
1.7812
1.7751
1.1996
1.0749
1.0603
1.0455
1.99
1.99
1.00
2.02
1.06
2.10
1.08
1.14
1.16
4.05
3.00
2.97
f1 (ppm)

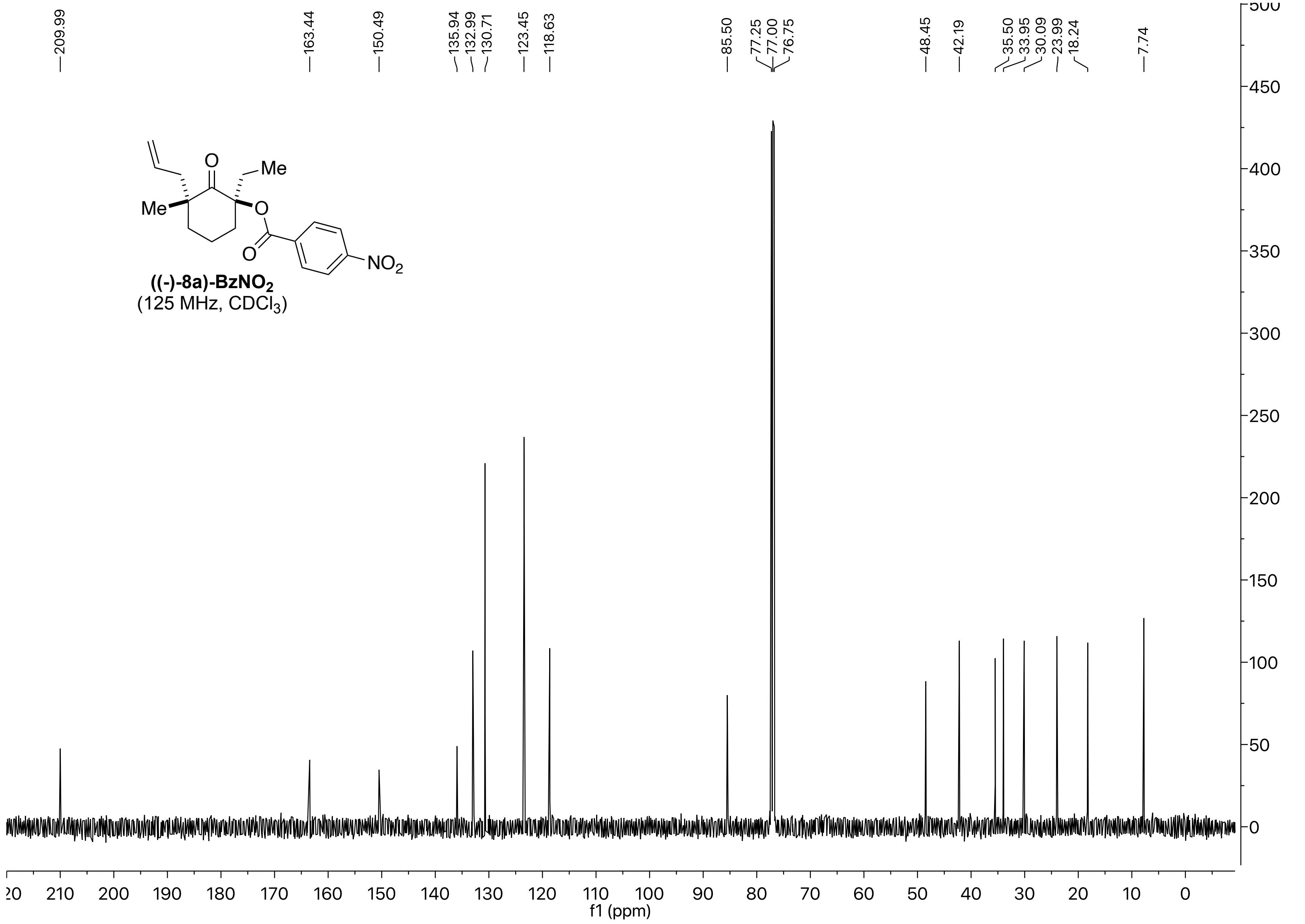

209.99
163.44
150.49
135.94
132.99
130.71
123.45
118.63
85.50
77.25
77.00
76.75
48.45
42.19
35.50
33.95
30.09
23.99
18.24
7.74
Me
O
Me
O
O
NO_2
((-)-8a)-$BzNO_2$
(125 MHz, $CDCl_3$)
f1 (ppm)

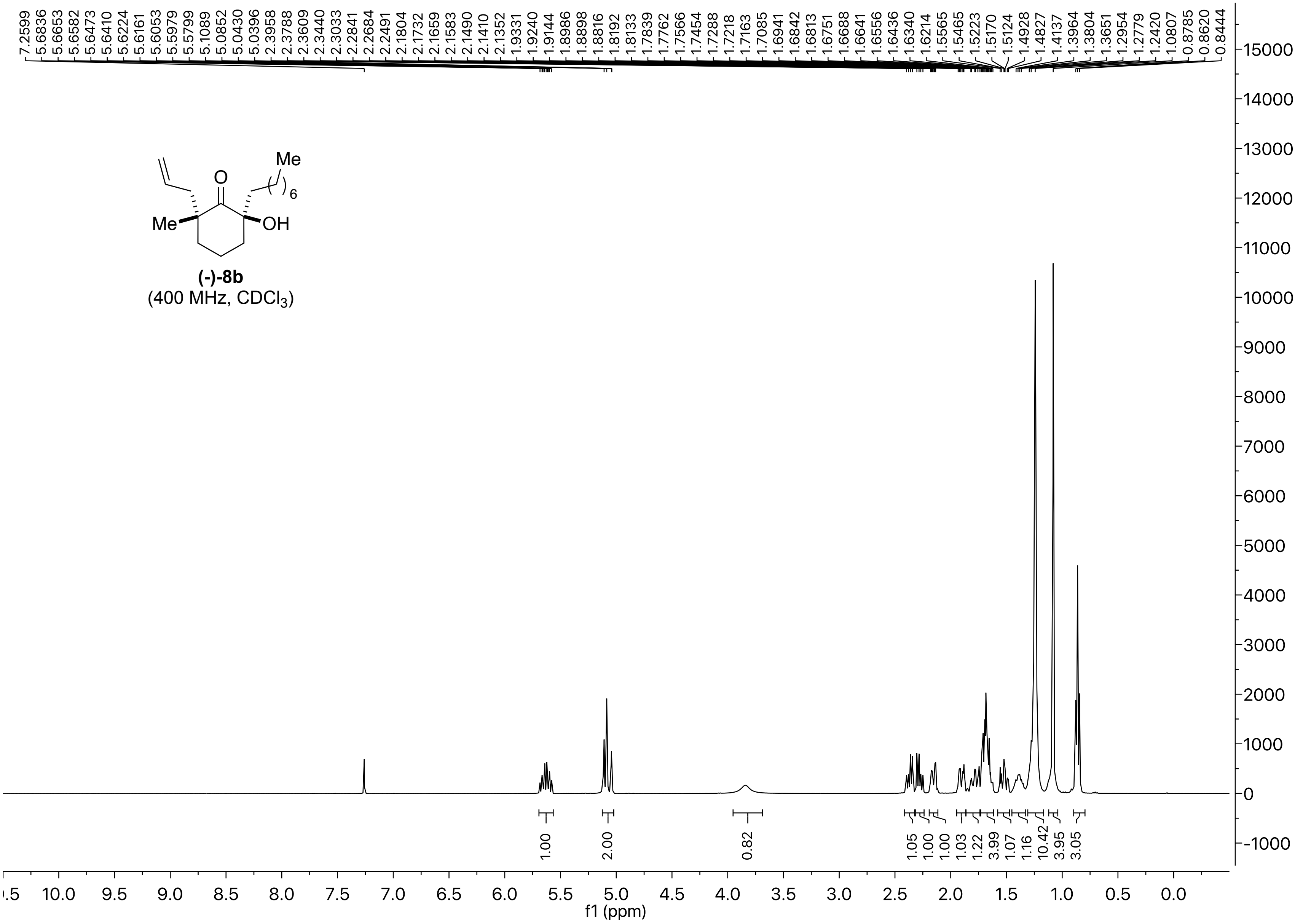
(-)-8b
(400 MHz, CDCl3)
f1 (ppm)

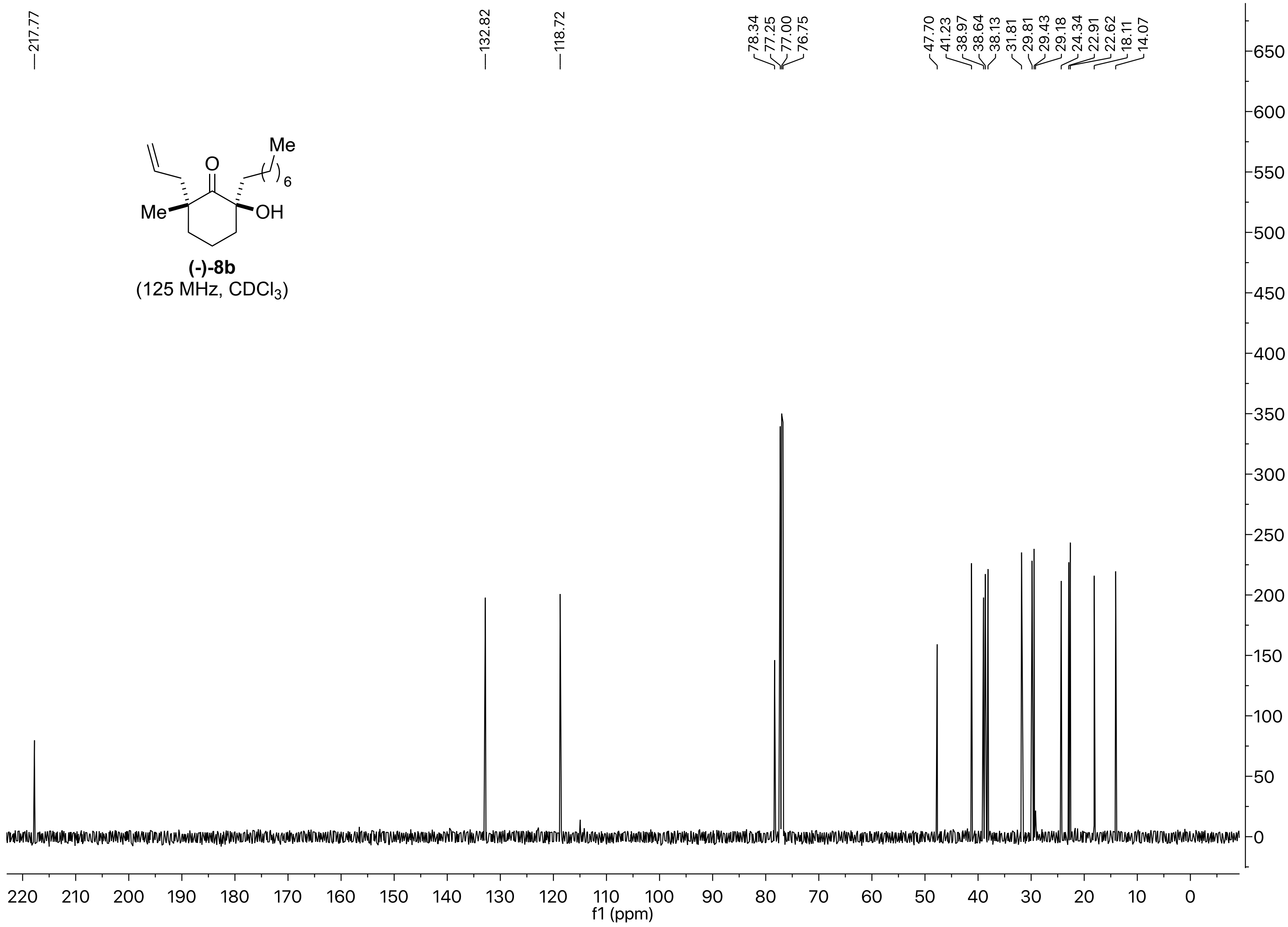

217.77
132.82
118.72
78.34
77.25
77.00
76.75
47.70
41.23
38.97
38.64
38.13
31.81
29.81
29.43
29.18
24.34
22.91
22.62
18.11
14.07
Me
O
Me
OH
6
(-)-8b
(125 MHz, CDCl3)
f1 (ppm)

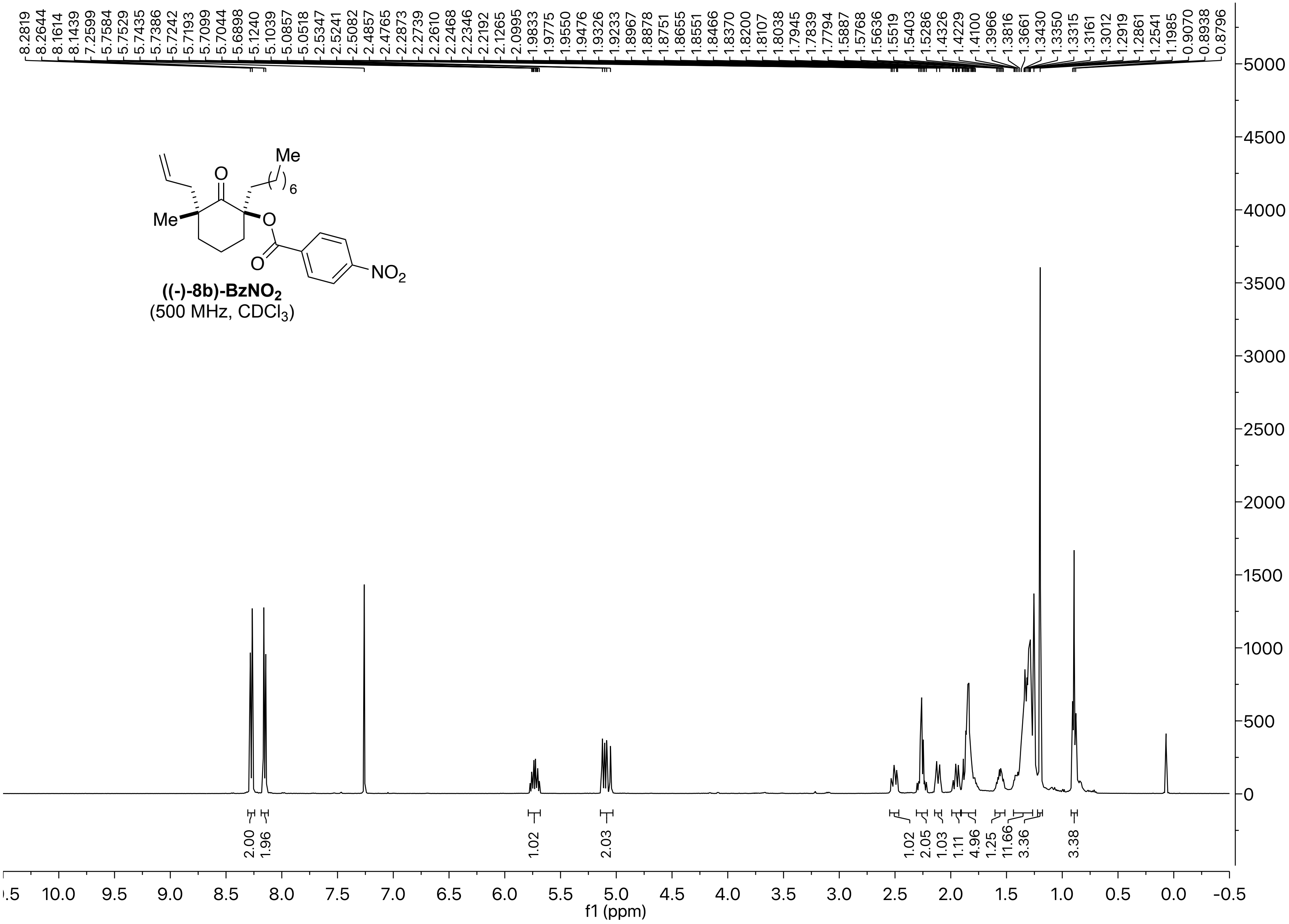

((-)-8b)-BzNO2
(500 MHz, CDCl3)
f1 (ppm)

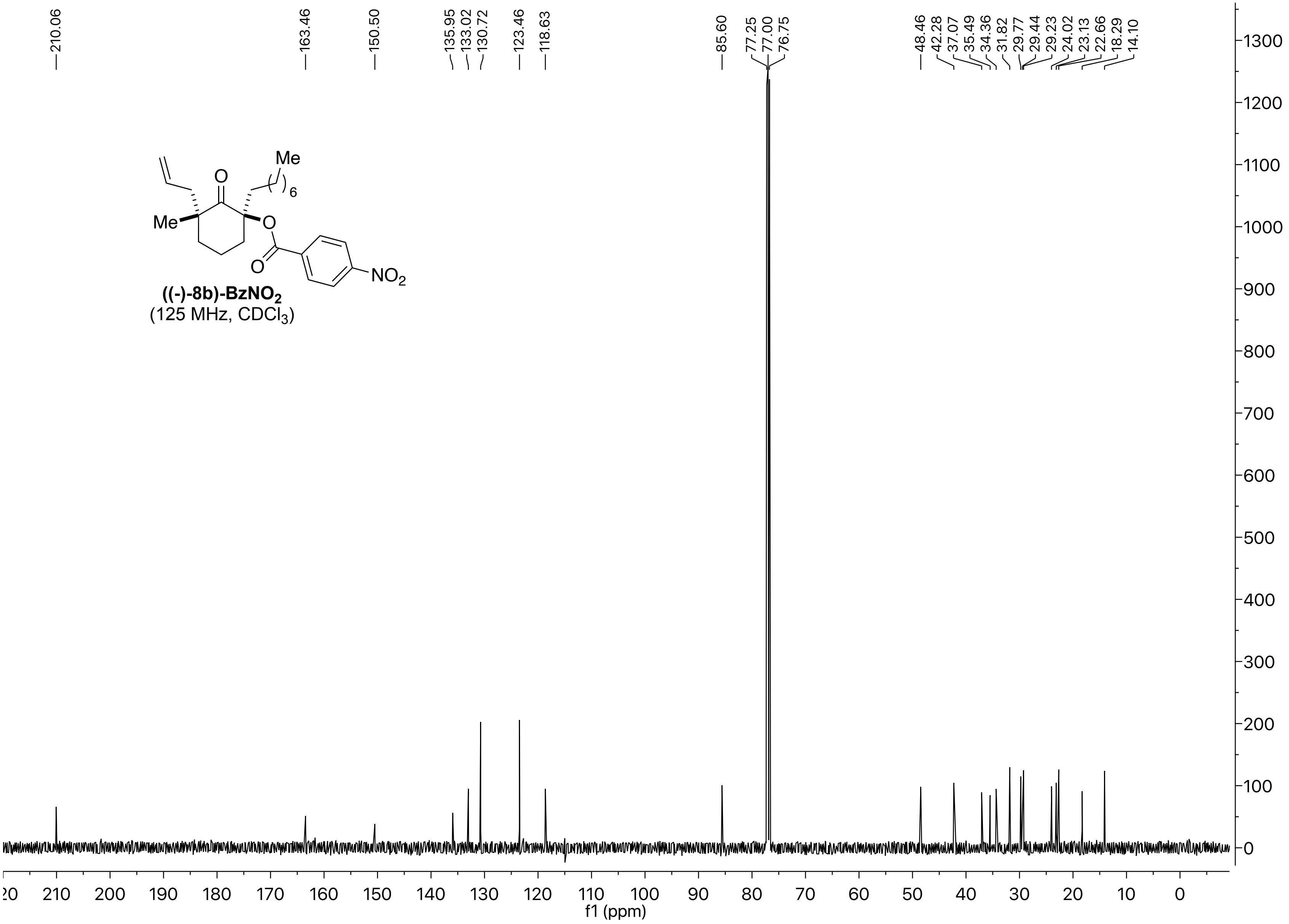

210.06
163.46
150.50
135.95
133.02
130.72
123.46
118.63
85.60
77.25
77.00
76.75
48.46
42.28
37.07
35.49
34.36
31.82
29.77
29.44
29.23
24.02
23.13
22.66
18.29
14.10
Me
O
6
Me
O
O
NO2
((-)-8b)-BzNO2
(125 MHz, CDCl3)
f1 (ppm)

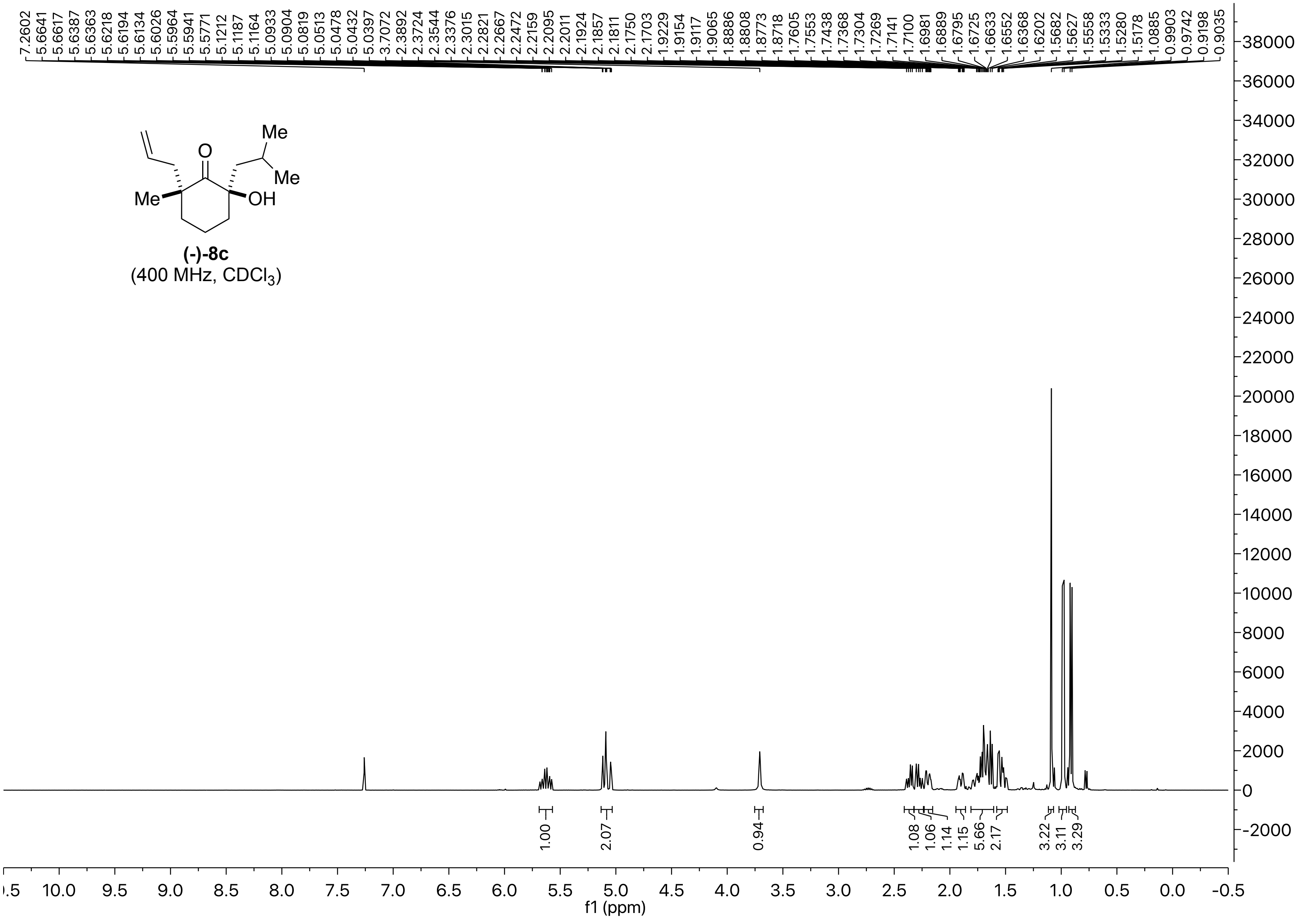

Me
O
Me
Me
OH
(-)-8c
(400 MHz, CDCl3)
f1 (ppm)

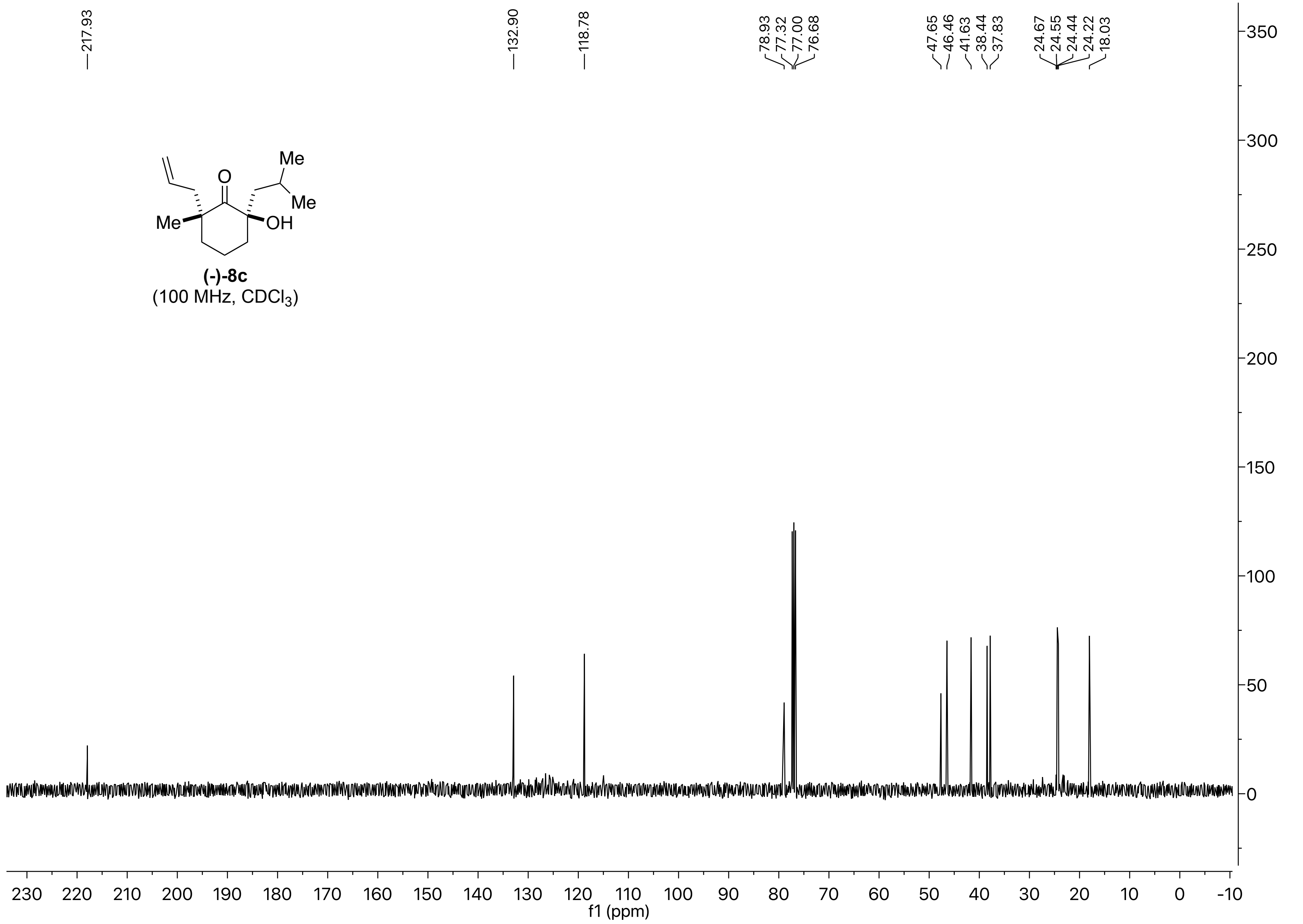

217.93
132.90
118.78
78.93
77.32
77.00
76.68
47.65
46.46
41.63
38.44
37.83
24.67
24.55
24.44
24.22
18.03
Me
O
Me
Me
OH
(-)-8c
(100 MHz, CDCl3)
f1 (ppm)

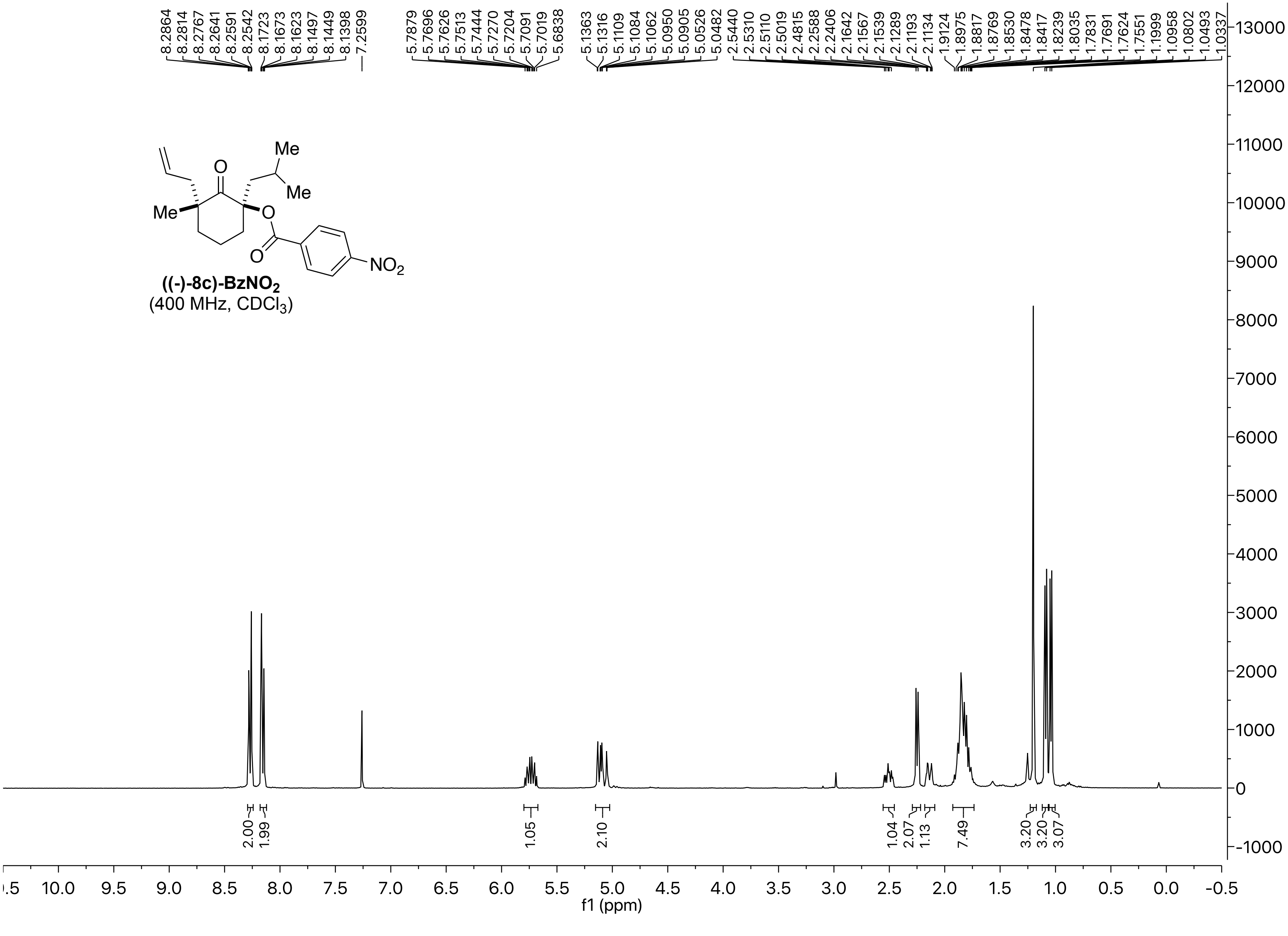

((-)-8c)-BzNO2
(400 MHz, CDCl3)
f1 (ppm)

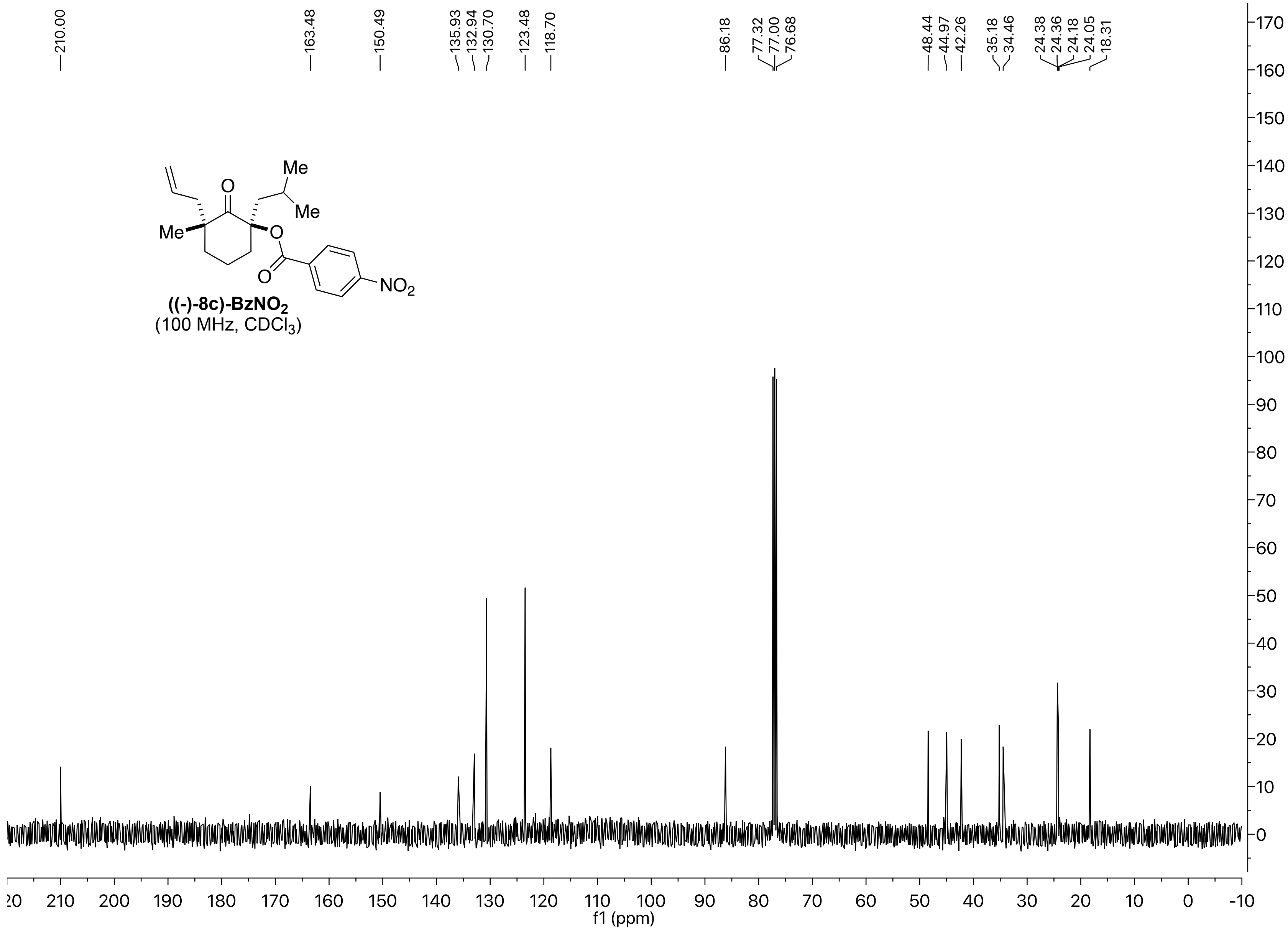
210.00
163.48
150.49
135.93
132.94
130.70
123.48
118.70
86.18
77.32
77.00
76.68
48.44
44.97
42.26
35.18
34.46
24.38
24.36
24.18
24.05
18.31
Me
O
Me
Me
O
O
NO_2
((-)-8c)-BzNO$_2$
(100 MHz, $CDCl_3$)
f1 (ppm)

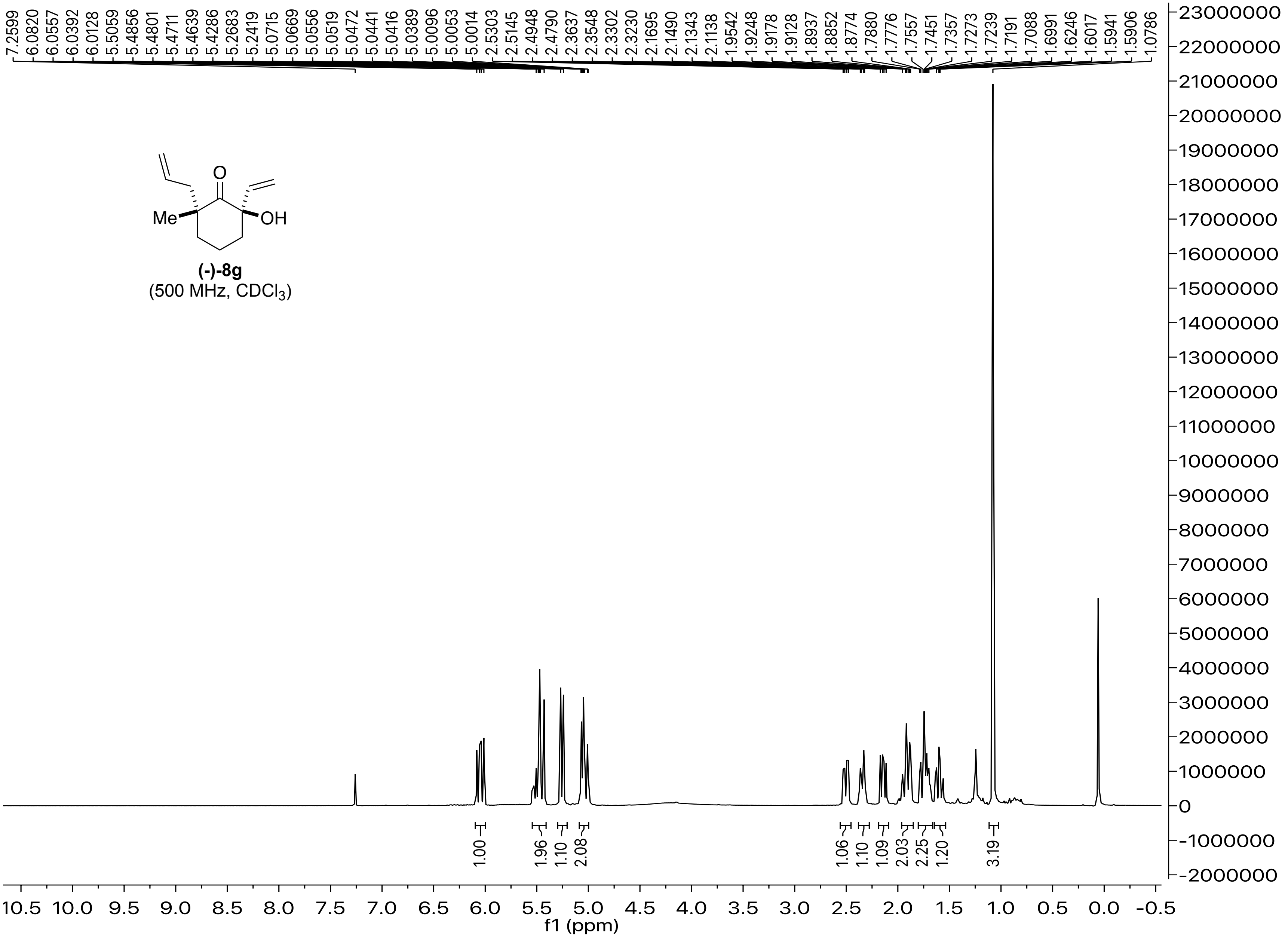

(-)-8g
(500 MHz, CDCl3)
f1 (ppm)

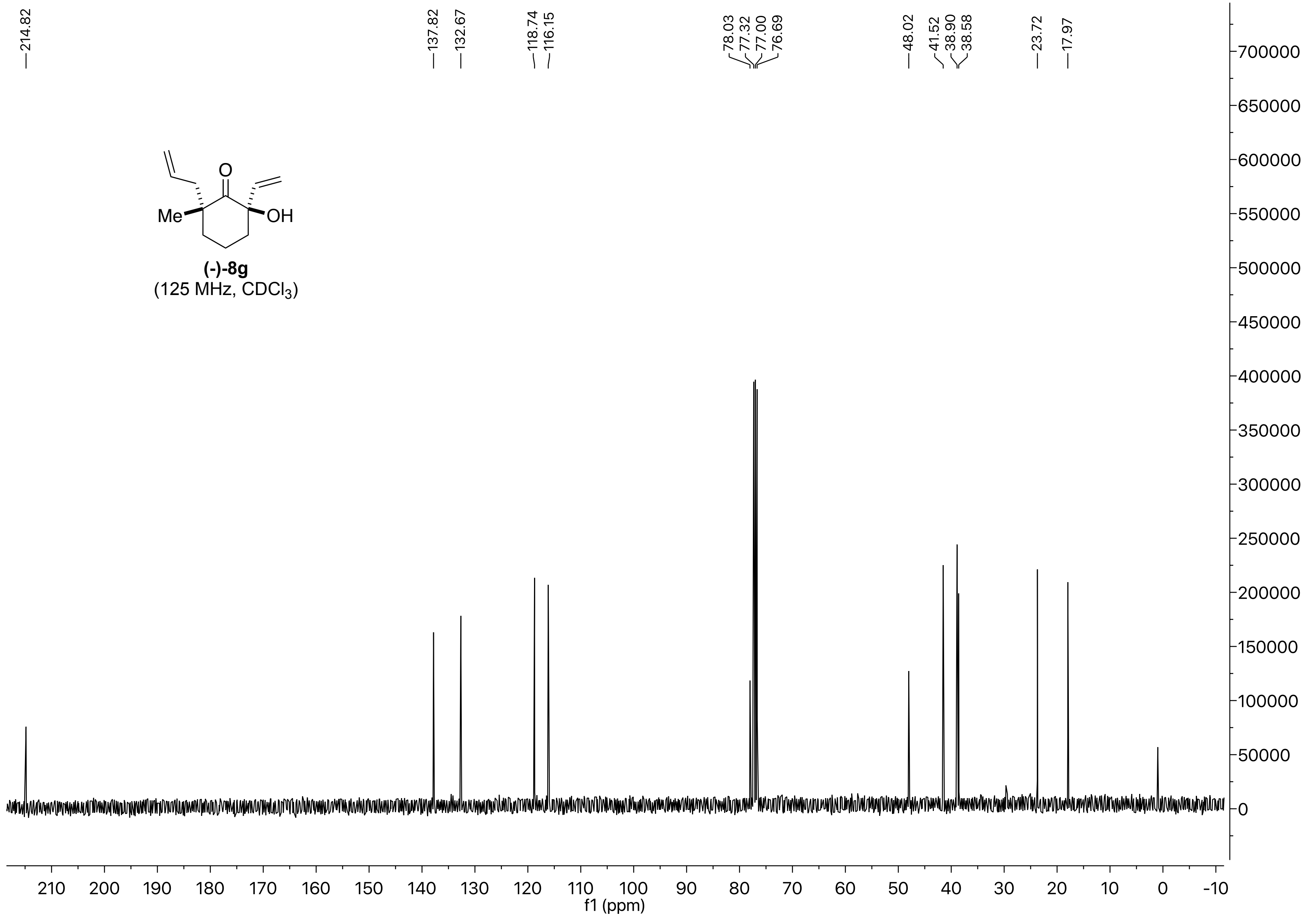
214.82
137.82
132.67
118.74
116.15
78.03
77.32
77.00
76.69
48.02
41.52
38.90
38.58
23.72
17.97
700000
650000
600000
550000
500000
450000
400000
350000
300000
250000
200000
150000
100000
50000
0
210
200
190
180
170
160
150
140
130
120
110
100
90
80
70
60
50
40
30
20
10
0
-10
f1 (ppm)
O
Me
OH
(-)-8g
(125 MHz, CDCl3)

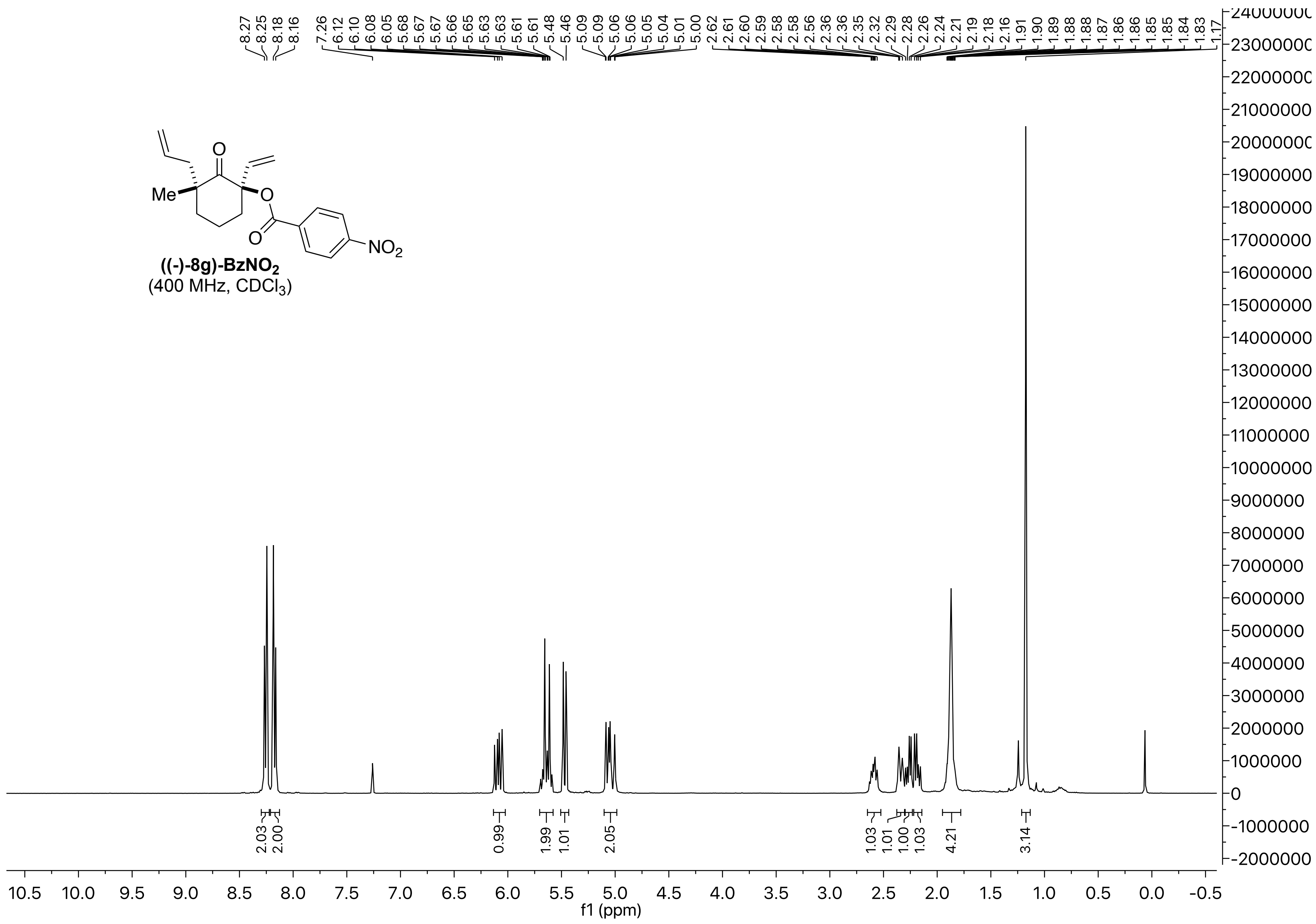

Me
O
O
O
NO2
((-)-8g)-BzNO2
(400 MHz, CDCl3)
f1 (ppm)

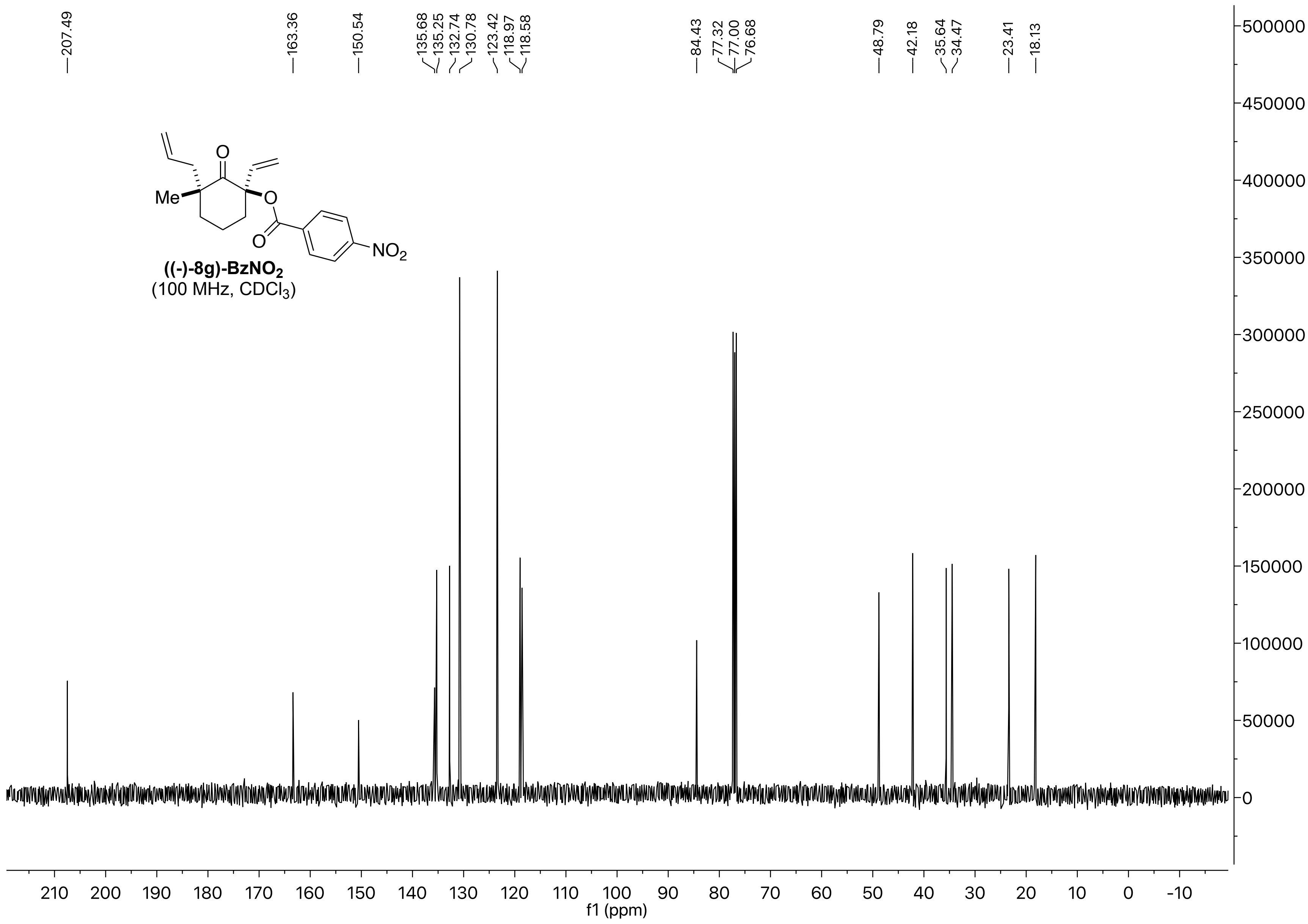
207.49
163.36
150.54
135.68
135.25
132.74
130.78
123.42
118.97
118.58
84.43
77.32
77.00
76.68
48.79
42.18
35.64
34.47
23.41
18.13
O
Me
O
O
NO2
((-)-8g)-BzNO2
(100 MHz, CDCl3)
500000
450000
400000
350000
300000
250000
200000
150000
100000
50000
0
210 200 190 180 170 160 150 140 130 120 110 100 90 80 70 60 50 40 30 20 10 0 -10
f1 (ppm)

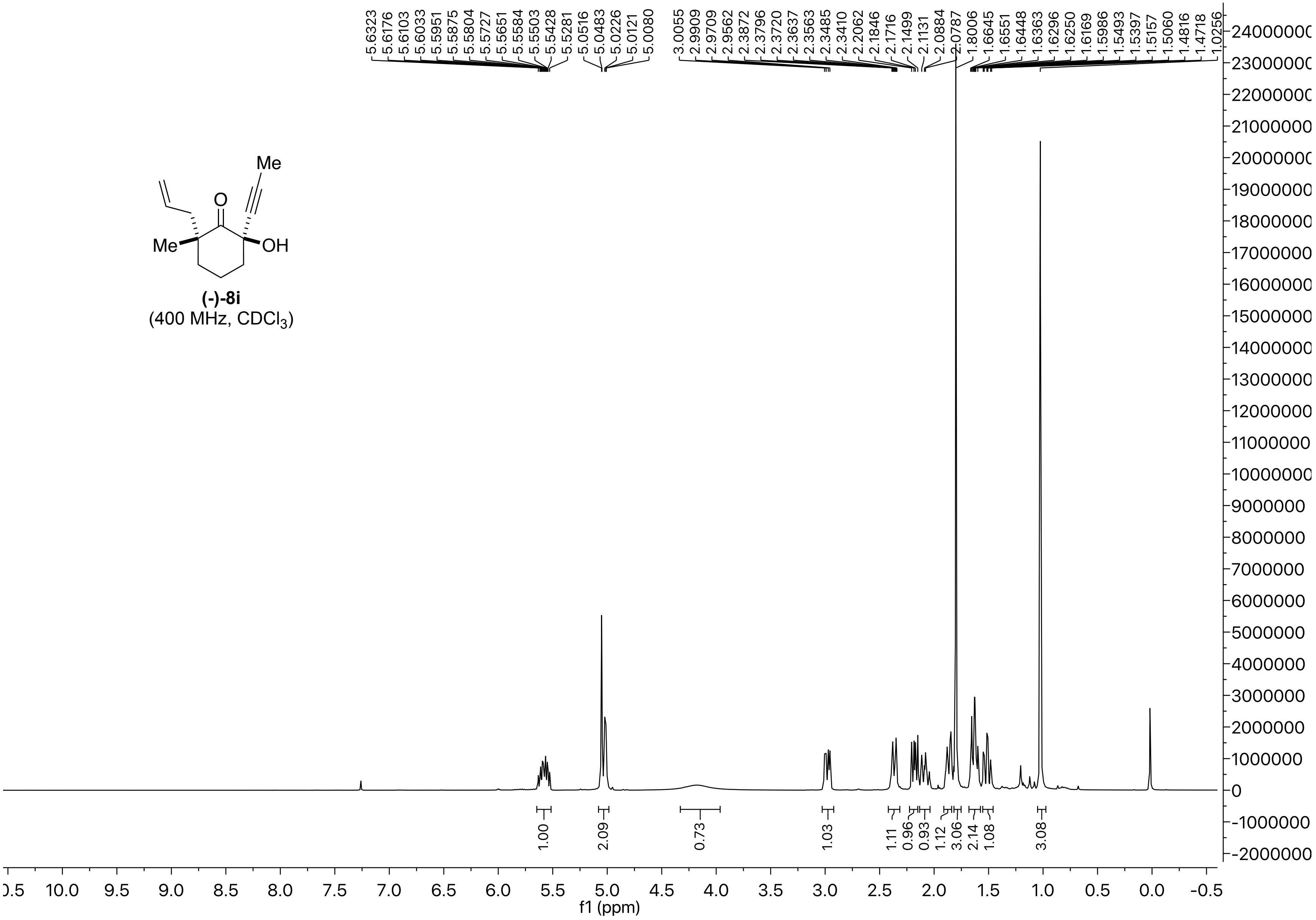

Me
O
Me
OH
(-)-8i
(400 MHz, CDCl3)
f1 (ppm)

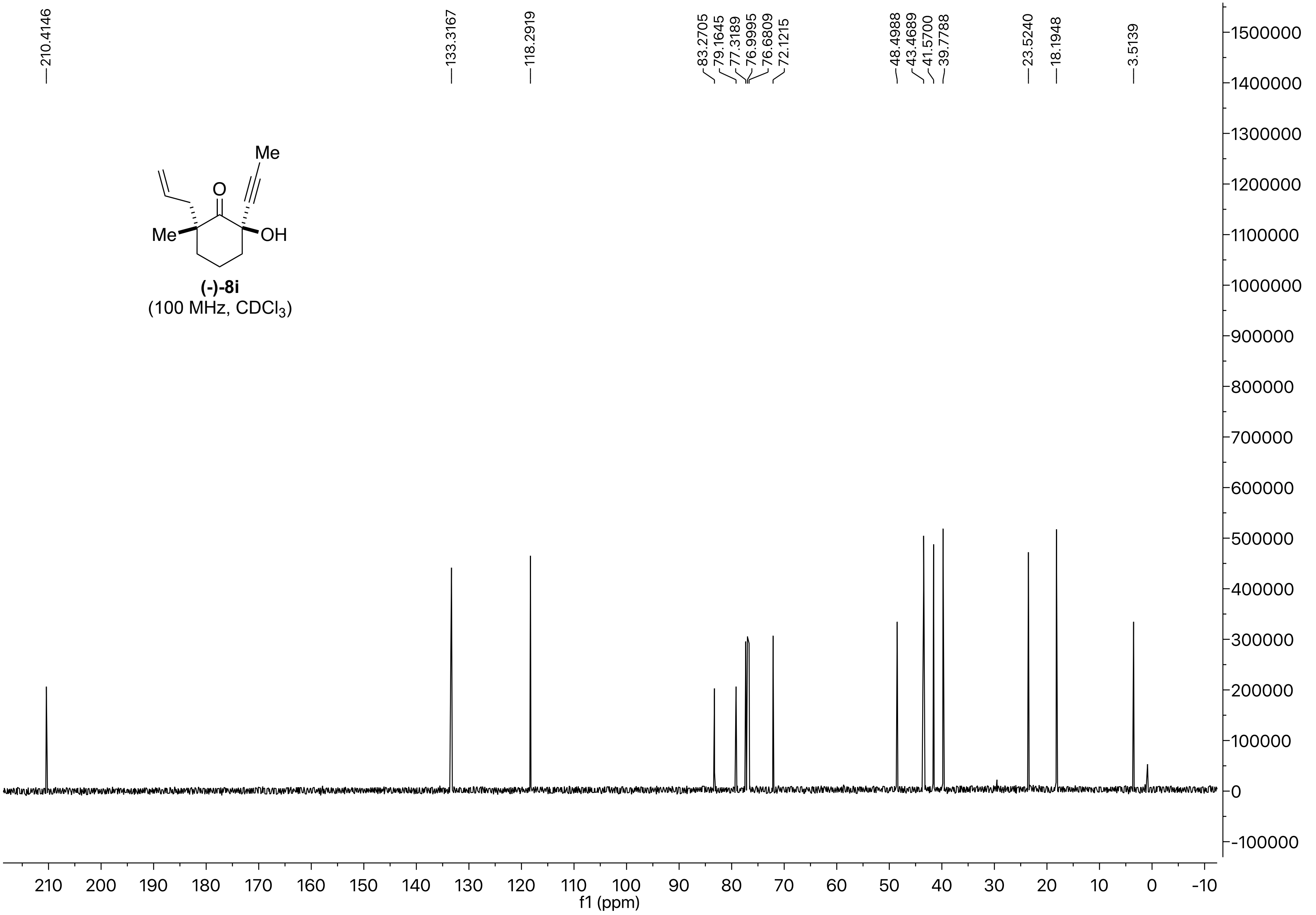

210.4146
133.3167
118.2919
83.2705
79.1645
77.3189
76.9995
76.6809
72.1215
48.4988
43.4689
41.5700
39.7788
23.5240
18.1948
3.5139
Me
O
Me
OH
(-)-8i
(100 MHz, CDCl3)
f1 (ppm)

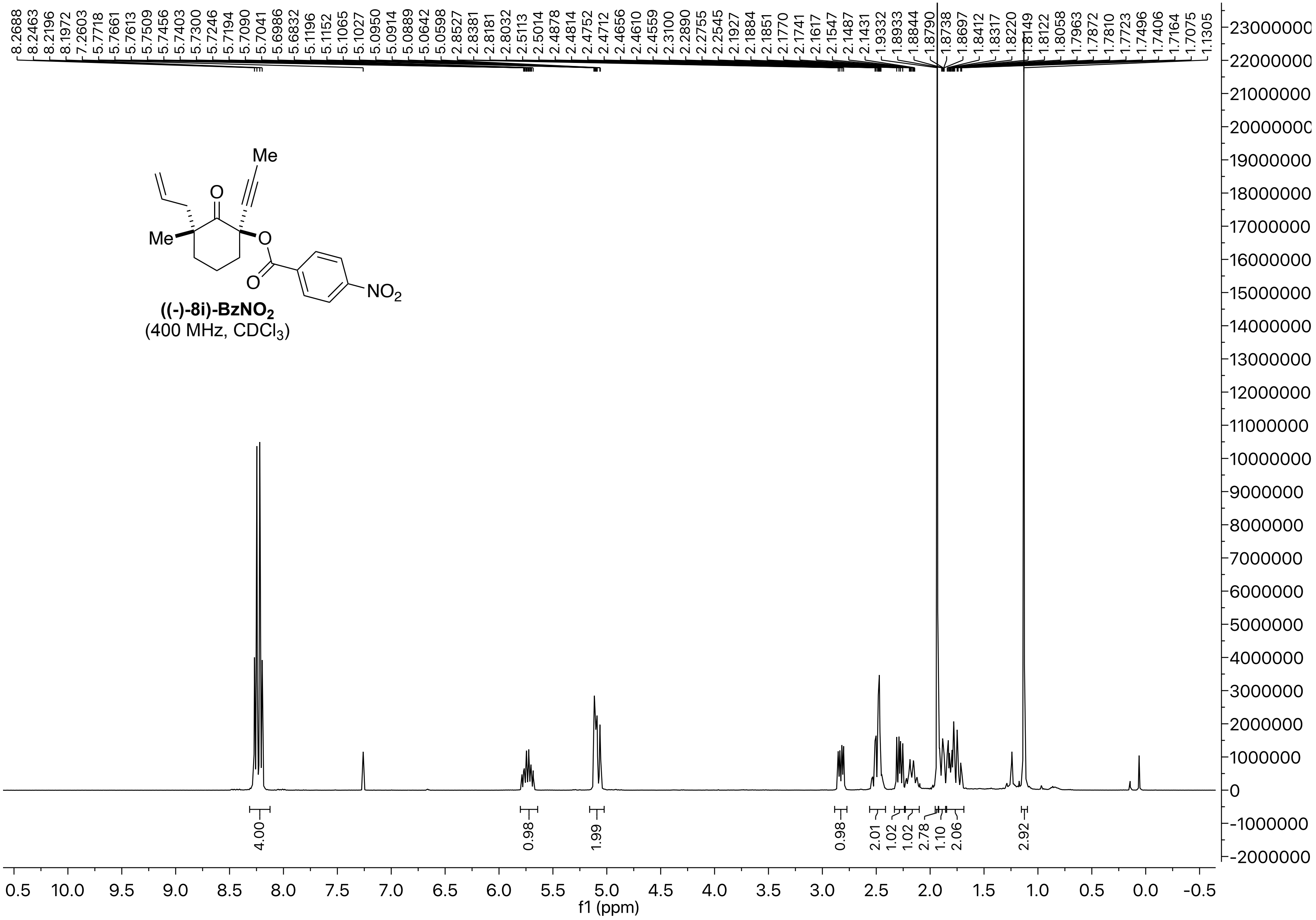
((-)-8i)-BzNO2
(400 MHz, CDCl3)
f1 (ppm)

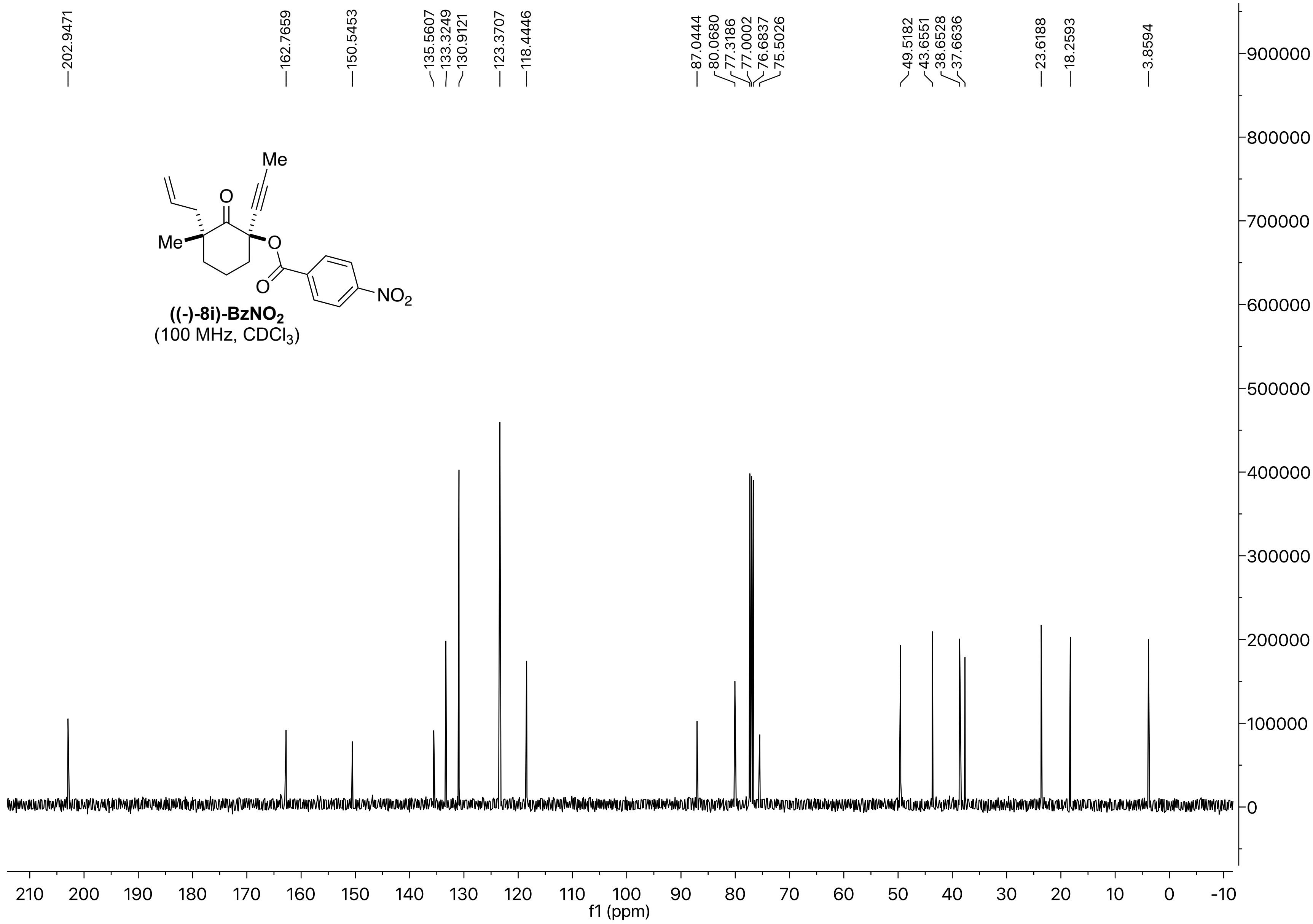

202.9471
162.7659
150.5453
135.5607
133.3249
130.9121
123.3707
118.4446
87.0444
80.0680
77.3186
77.0002
76.6837
75.5026
49.5182
43.6551
38.6528
37.6636
23.6188
18.2593
3.8594
Me
O
Me
O
O
NO2
((-)-8i)-BzNO2
(100 MHz, CDCl3)
f1 (ppm)

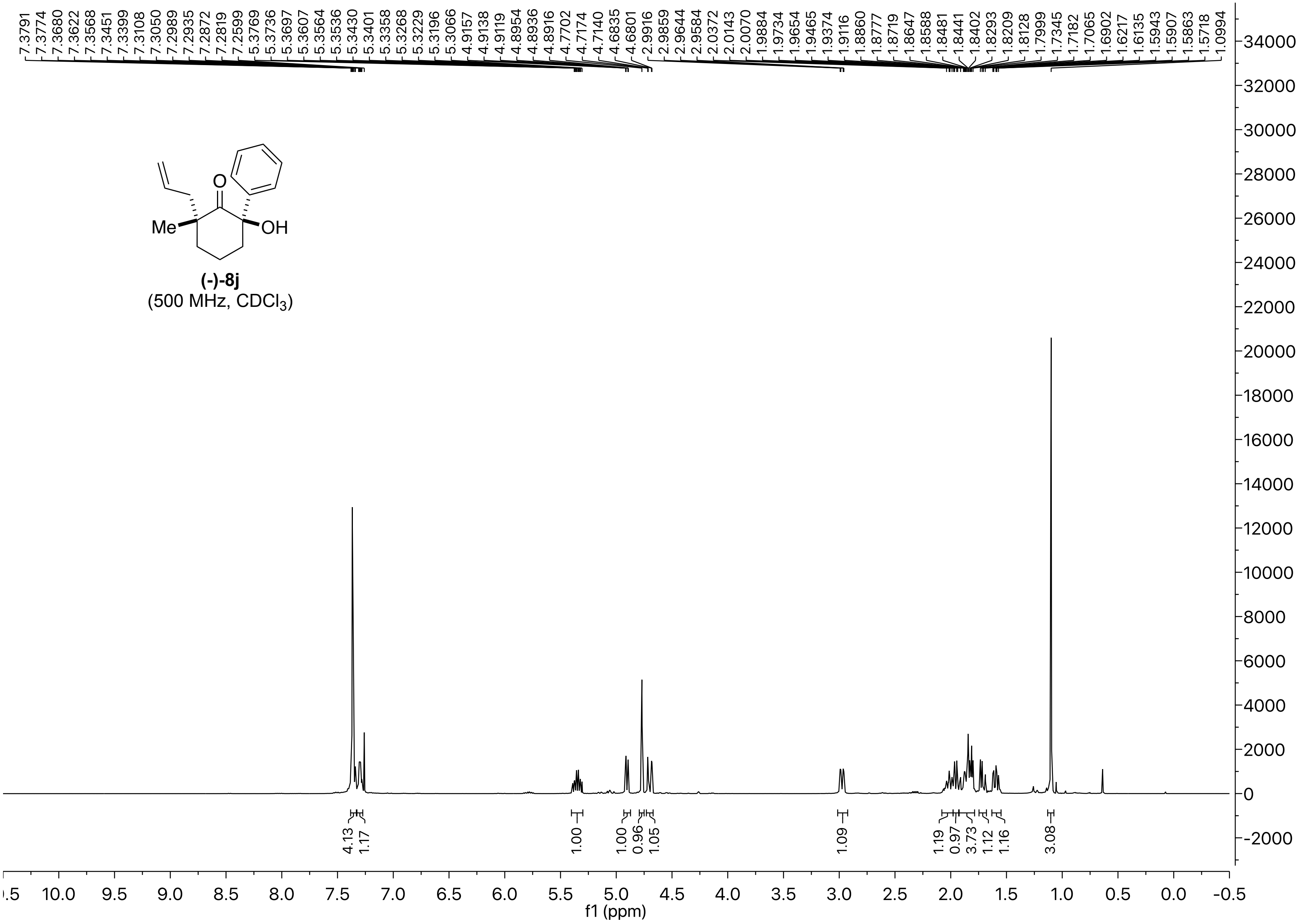

O
Me
OH
(-)-8j
(500 MHz, CDCl3)
f1 (ppm)

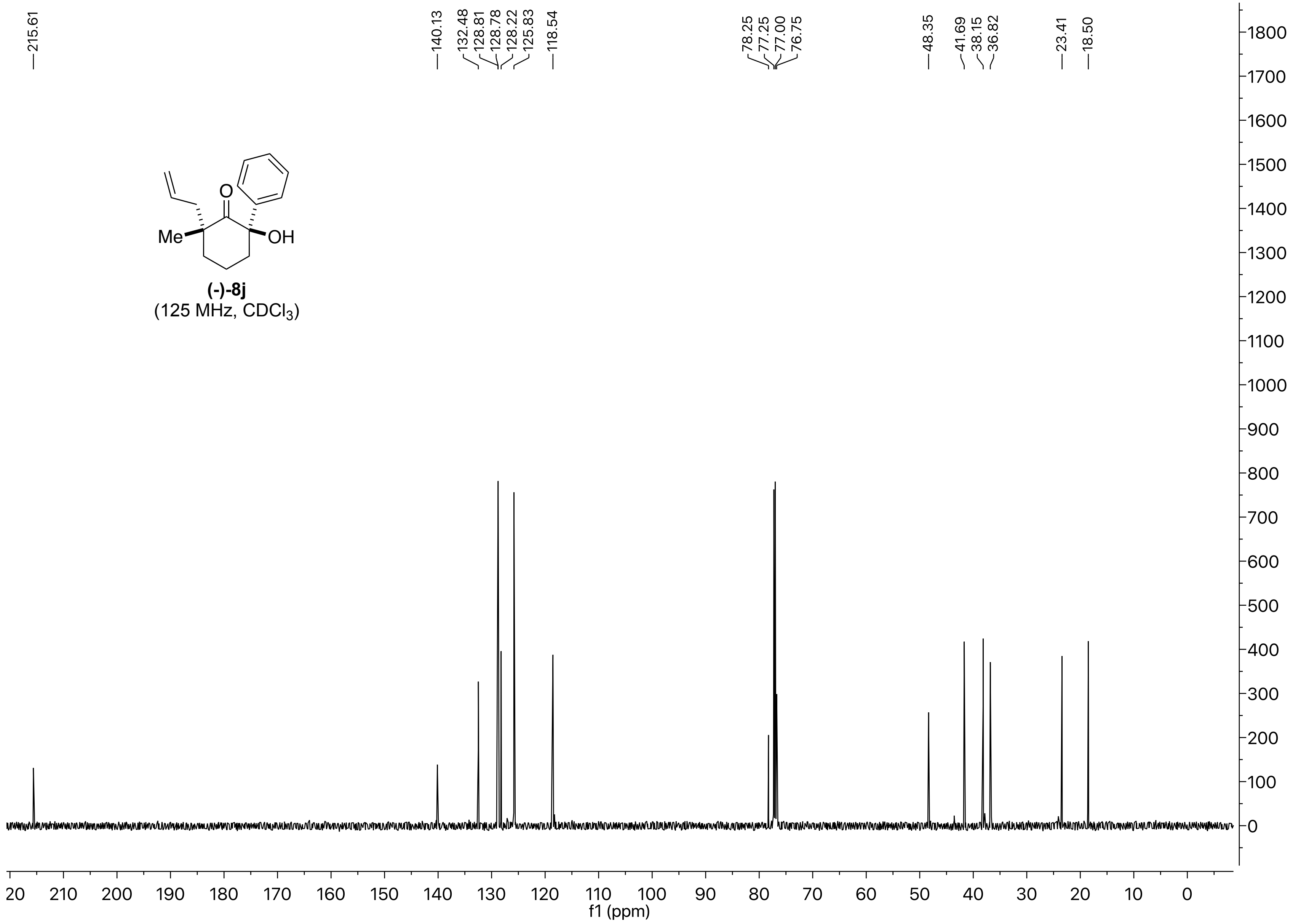

215.61
140.13
132.48
128.81
128.78
128.22
125.83
118.54
78.25
77.25
77.00
76.75
48.35
41.69
38.15
36.82
23.41
18.50
O
Me
OH
(-)-8j
(125 MHz, $CDCl_3$)
f1 (ppm)

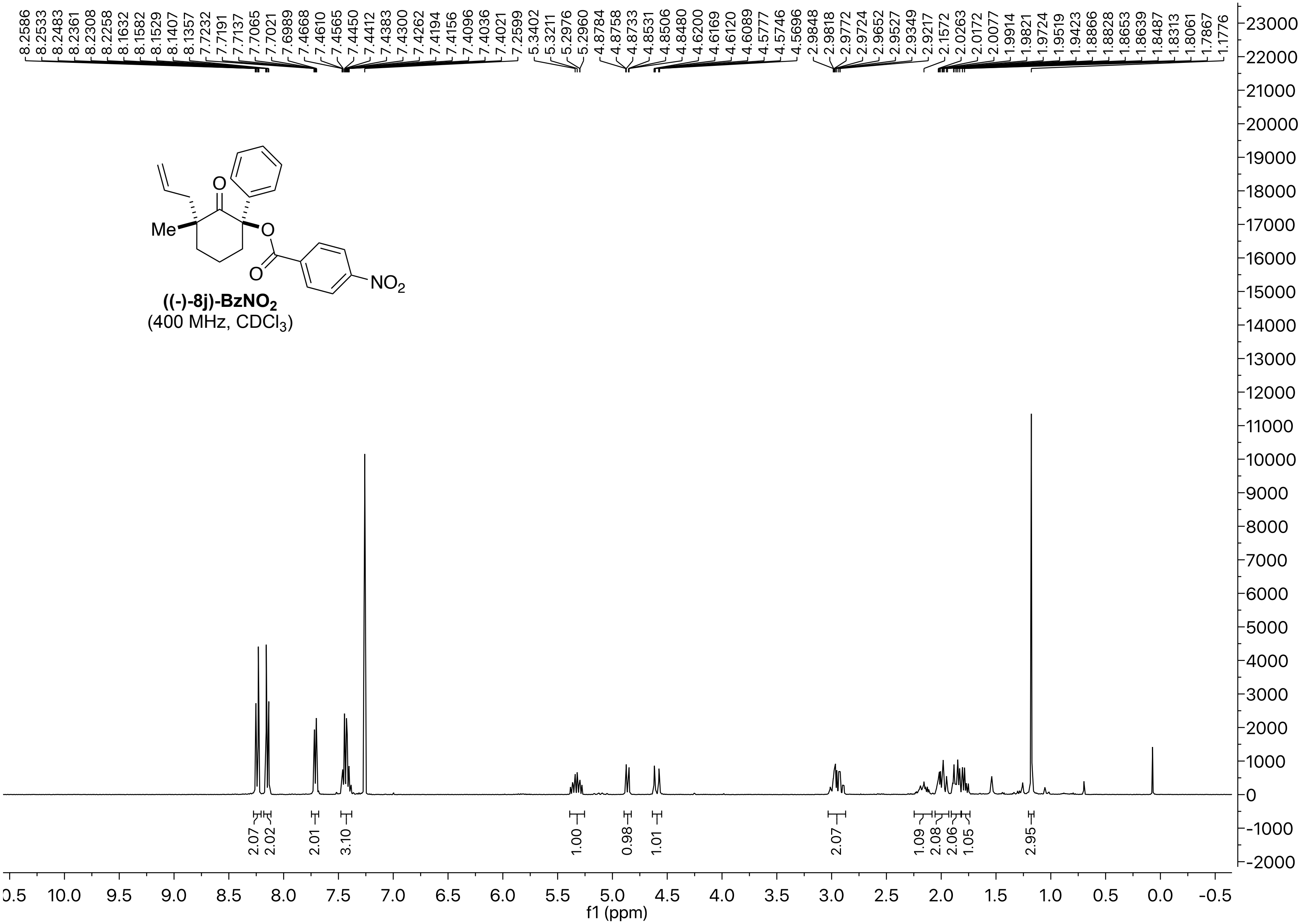

Me
O
O
O
NO2
((-)-8j)-BzNO2
(400 MHz, CDCl3)
f1 (ppm)

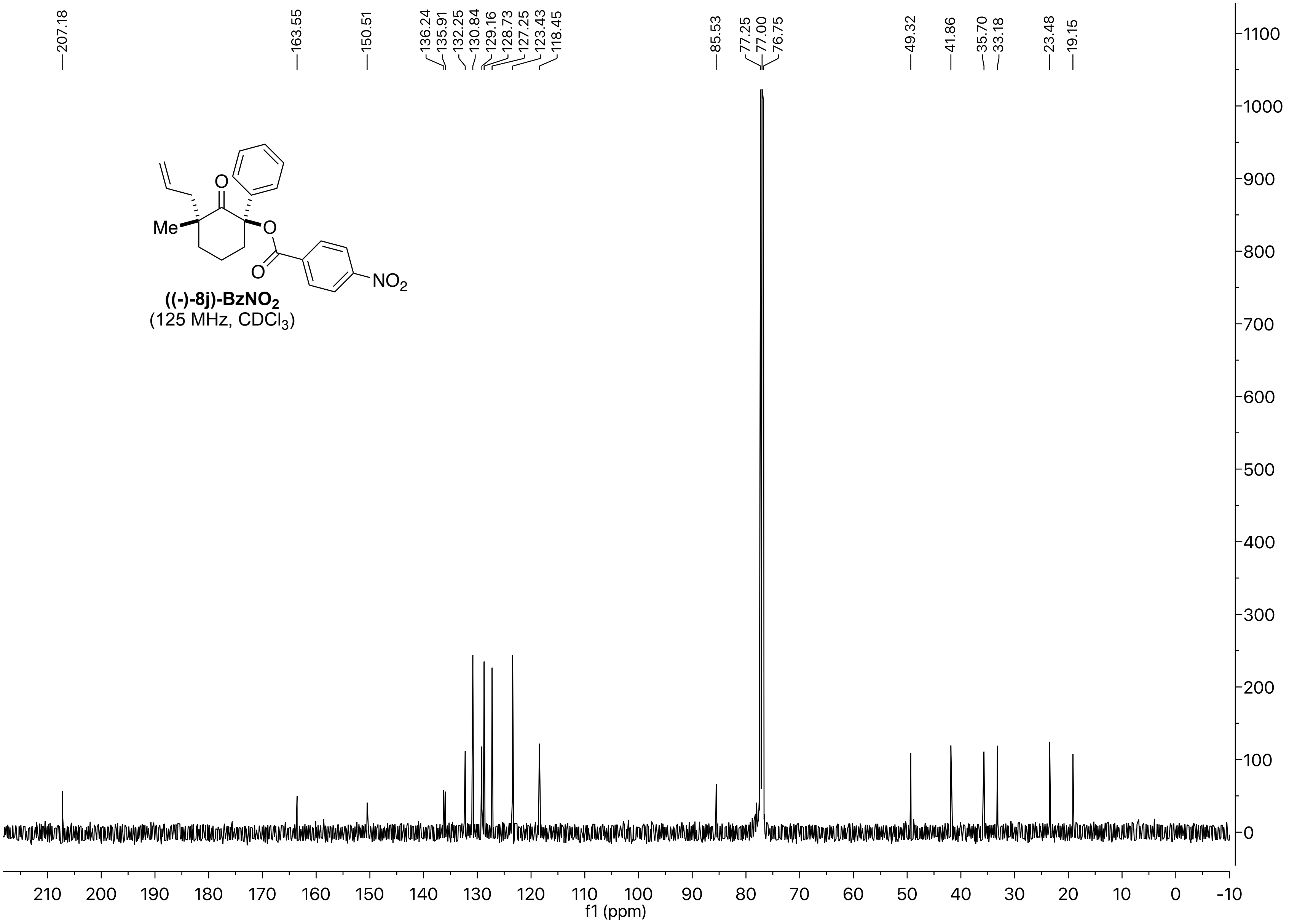
207.18
163.55
150.51
136.24
135.91
132.25
130.84
129.16
128.73
127.25
123.43
118.45
85.53
77.25
77.00
76.75
49.32
41.86
35.70
33.18
23.48
19.15
O
Me
O
O
NO2
((-)-8j)-BzNO2
(125 MHz, CDCl3)
f1 (ppm)

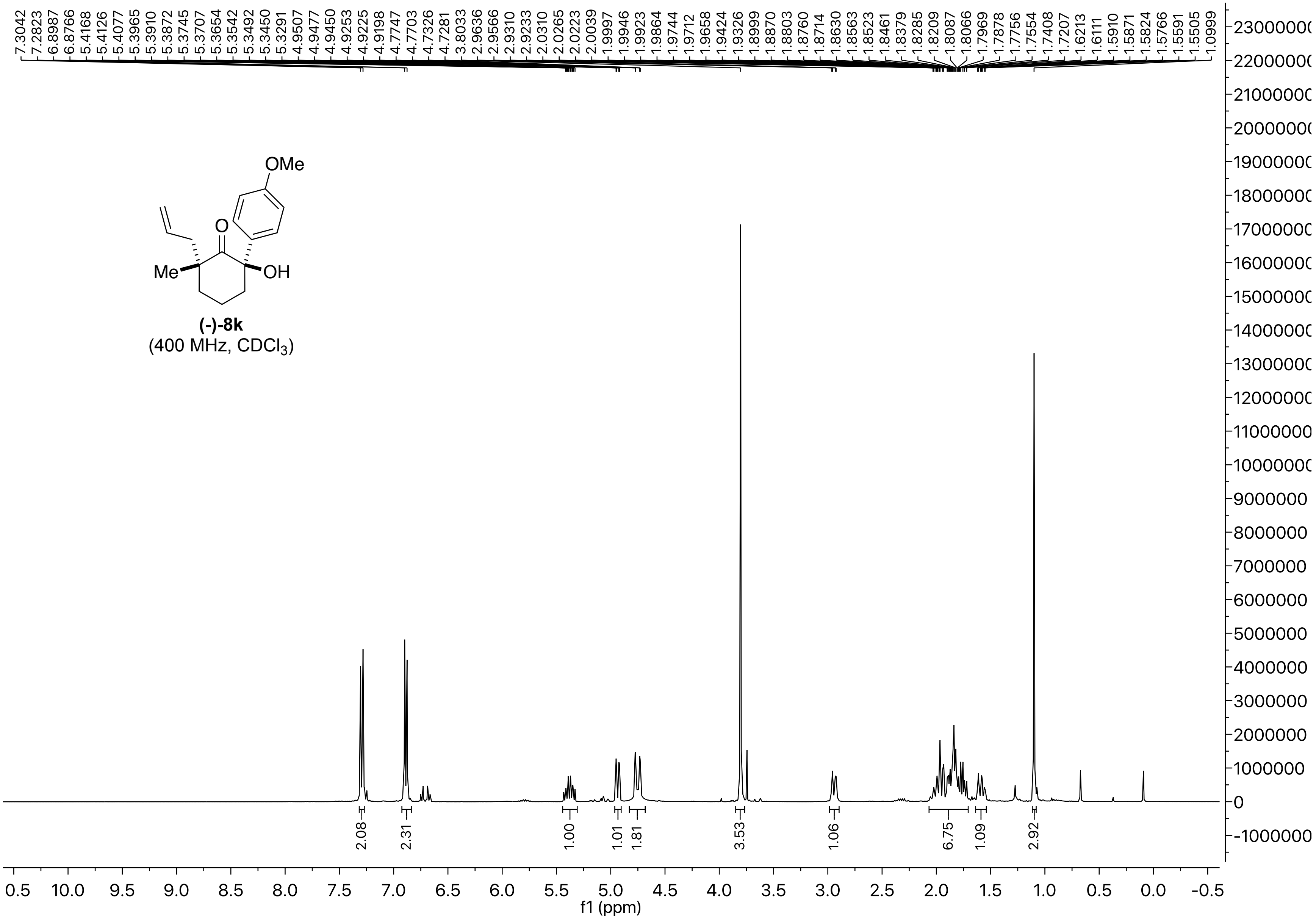

(-)-8k
(400 MHz, CDCl$_3$)

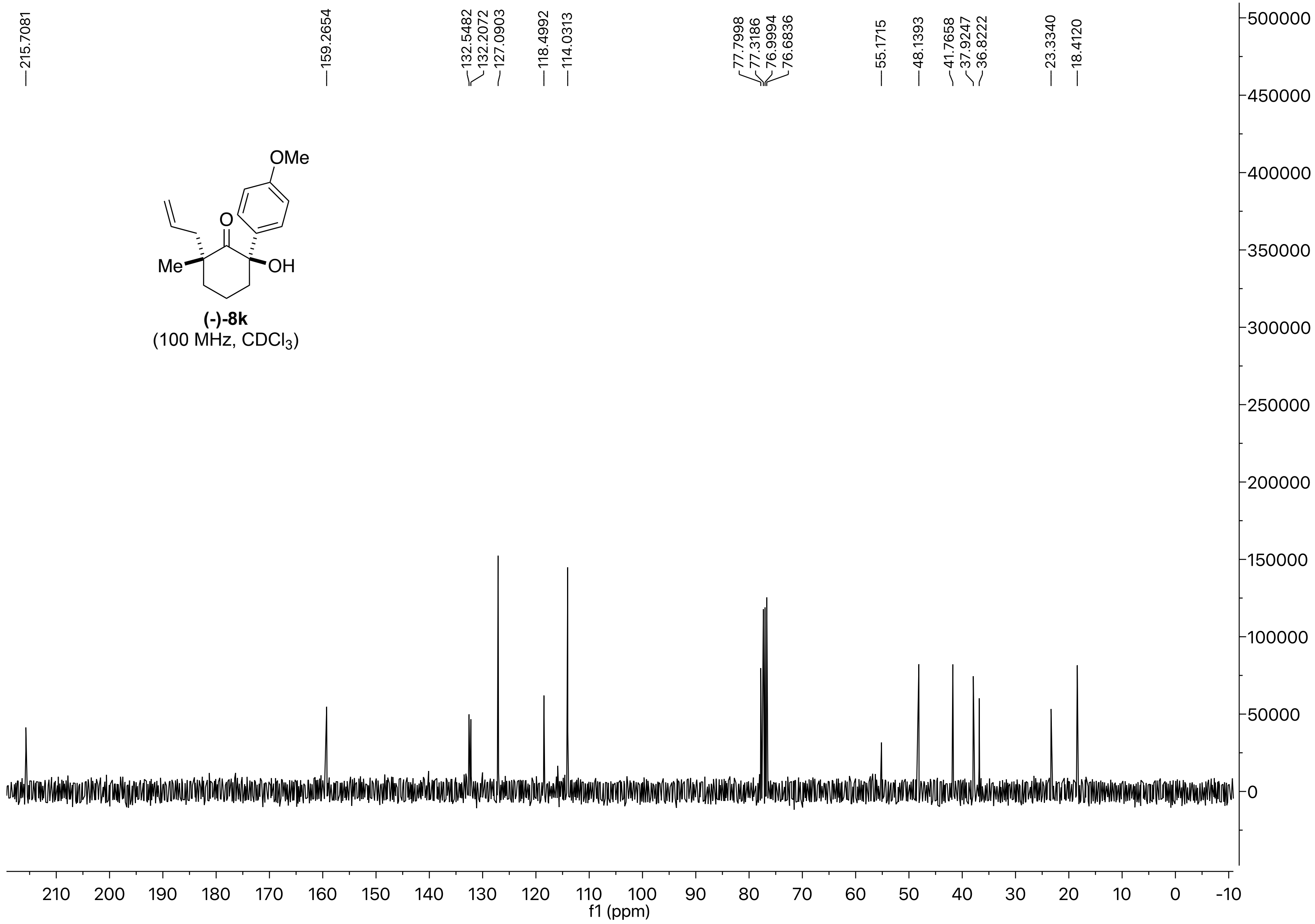
215.7081
159.2654
132.5482
132.2072
127.0903
118.4992
114.0313
77.7998
77.3186
76.9994
76.6836
55.1715
48.1393
41.7658
37.9247
36.8222
23.3340
18.4120
OMe
O
Me
OH
(-)-8k
(100 MHz, CDCl3)
f1 (ppm)

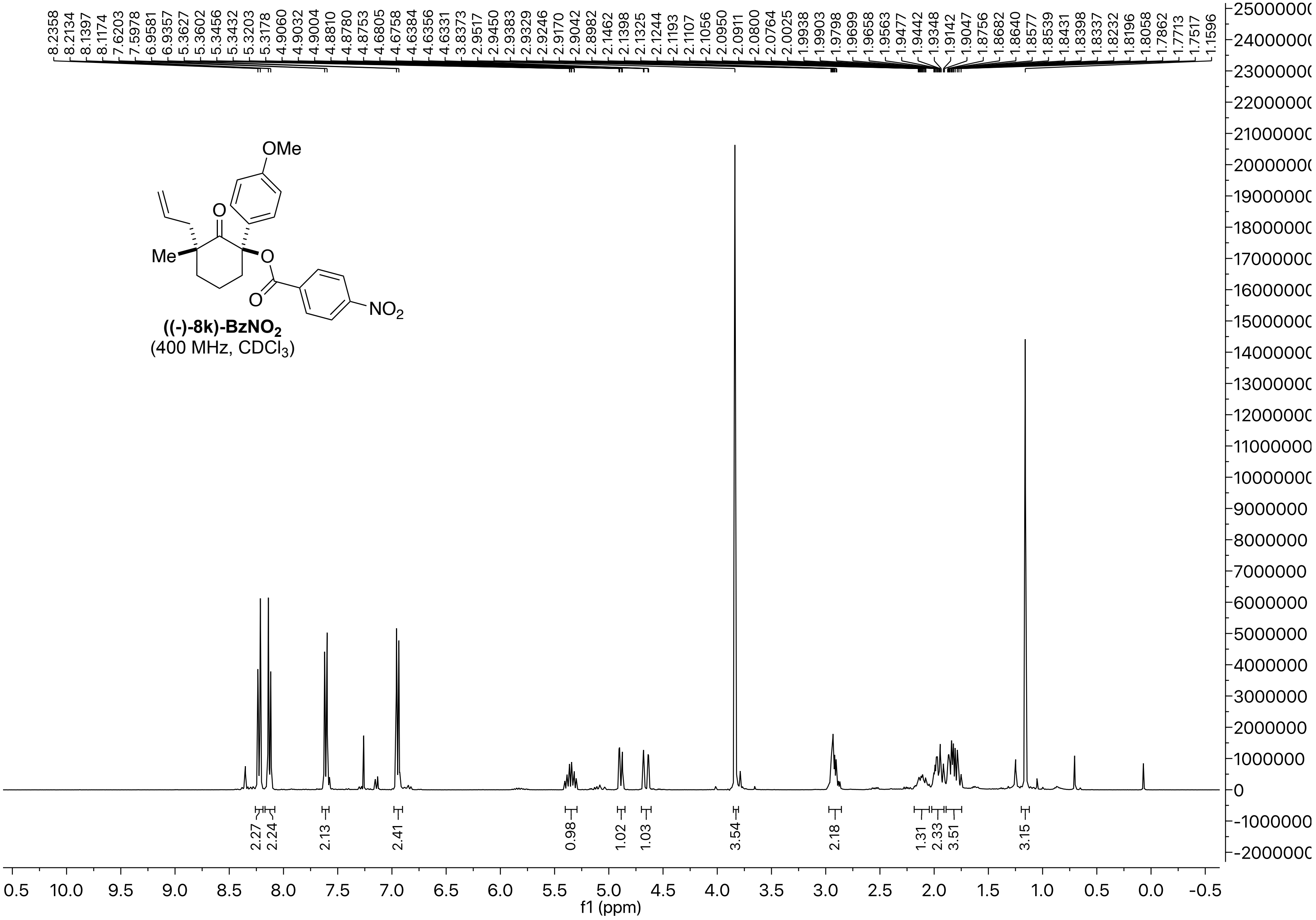

OMe
O
Me
O
O
NO2
((-)-8k)-BzNO2
(400 MHz, CDCl3)
f1 (ppm)

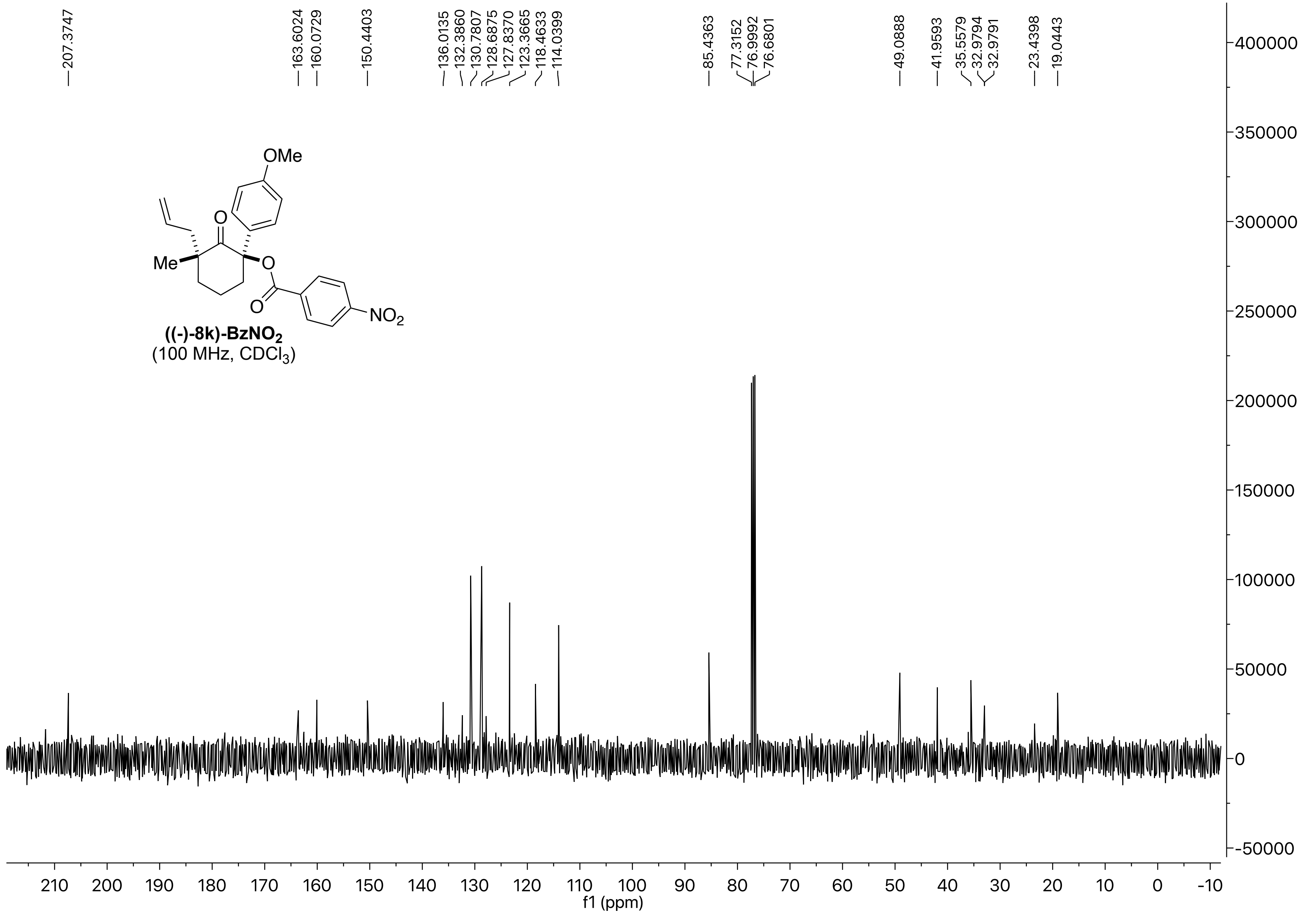
207.3747
163.6024
160.0729
150.4403
136.0135
132.3860
130.7807
128.6875
127.8370
123.3665
118.4633
114.0399
85.4363
77.3152
76.9992
76.6801
49.0888
41.9593
35.5579
32.9794
32.9791
23.4398
19.0443
OMe
O
Me
O
O
NO2
((-)-8k)-BzNO2
(100 MHz, CDCl3)
f1 (ppm)

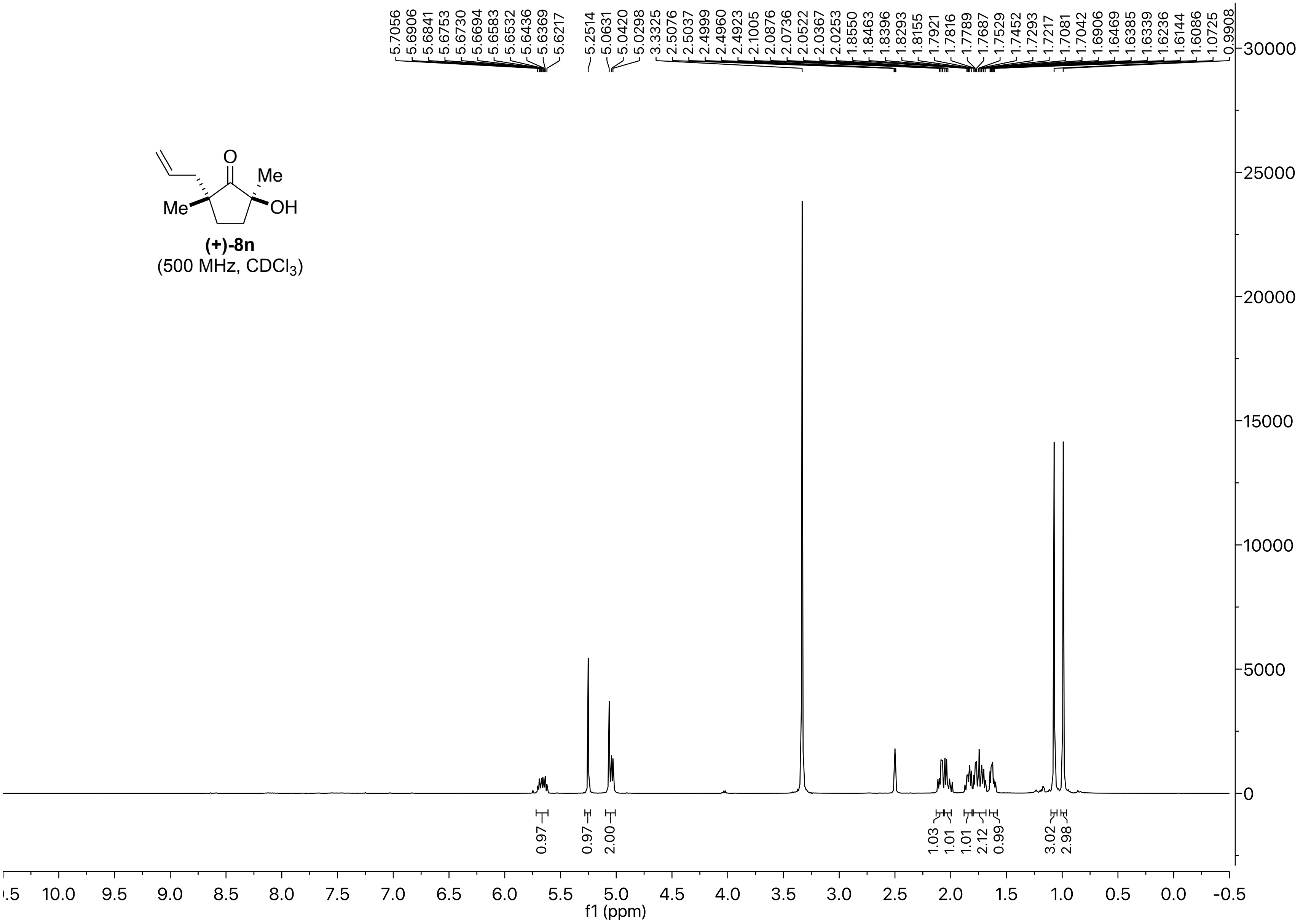

O
Me
Me
OH
(+)-8n
(500 MHz, CDCl$_3$)
5.7056
5.6906
5.6841
5.6753
5.6730
5.6694
5.6583
5.6532
5.6436
5.6369
5.6217
5.2514
5.0631
5.0420
5.0298
3.3325
2.5076
2.5037
2.4999
2.4960
2.4923
2.1005
2.0876
2.0736
2.0522
2.0367
2.0253
1.8550
1.8463
1.8396
1.8293
1.8155
1.7921
1.7816
1.7789
1.7687
1.7529
1.7452
1.7293
1.7217
1.7081
1.7042
1.6906
1.6469
1.6385
1.6339
1.6236
1.6144
1.6086
1.0725
0.9908
0.97
0.97
2.00
1.03
1.01
1.01
2.12
0.99
3.02
2.98
f1 (ppm)

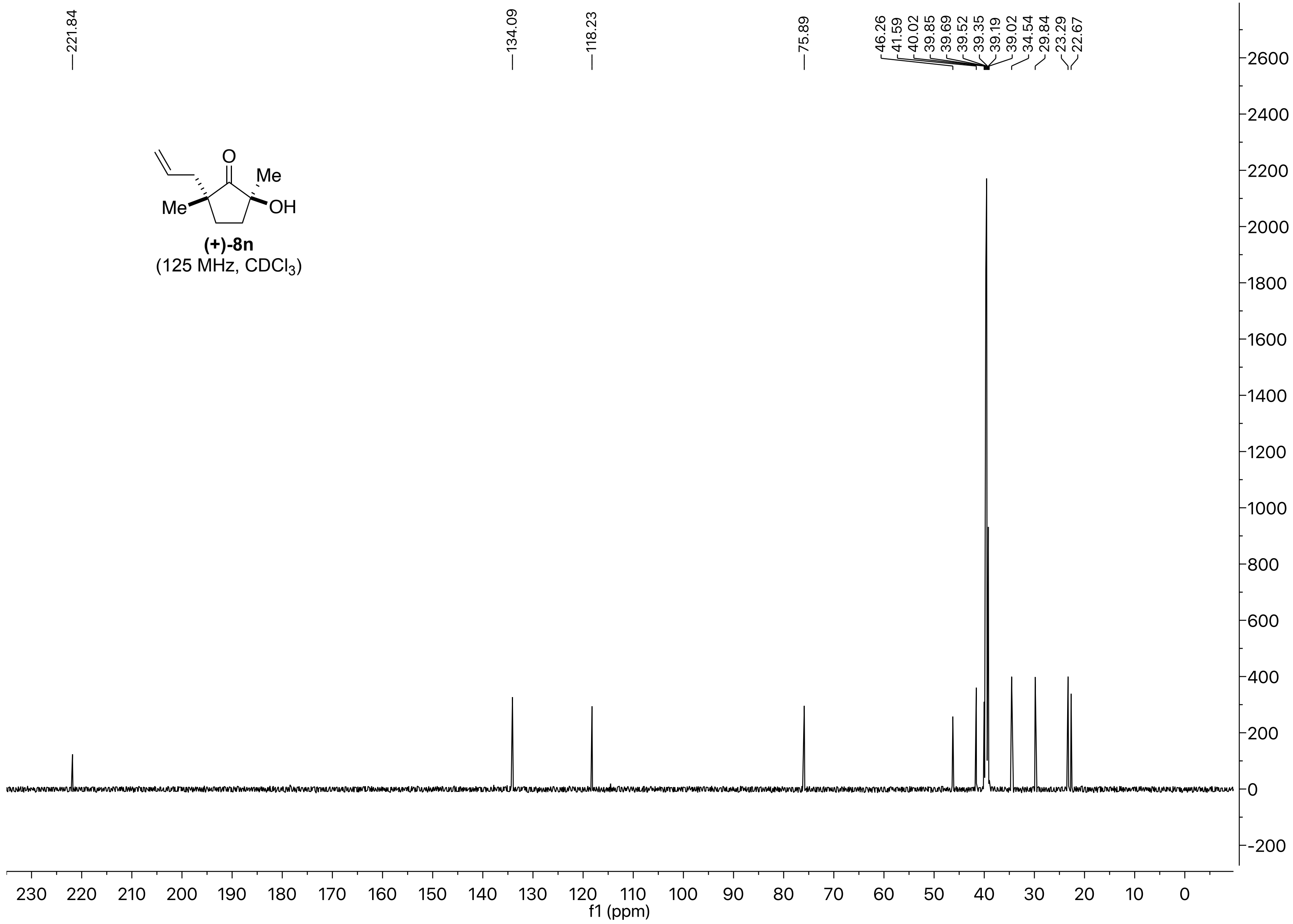
221.84
134.09
118.23
75.89
46.26
41.59
40.02
39.85
39.69
39.52
39.35
39.19
39.02
34.54
29.84
23.29
22.67
Me
O
OH
Me
(+)-8n
(125 MHz, $CDCl_3$)
f1 (ppm)

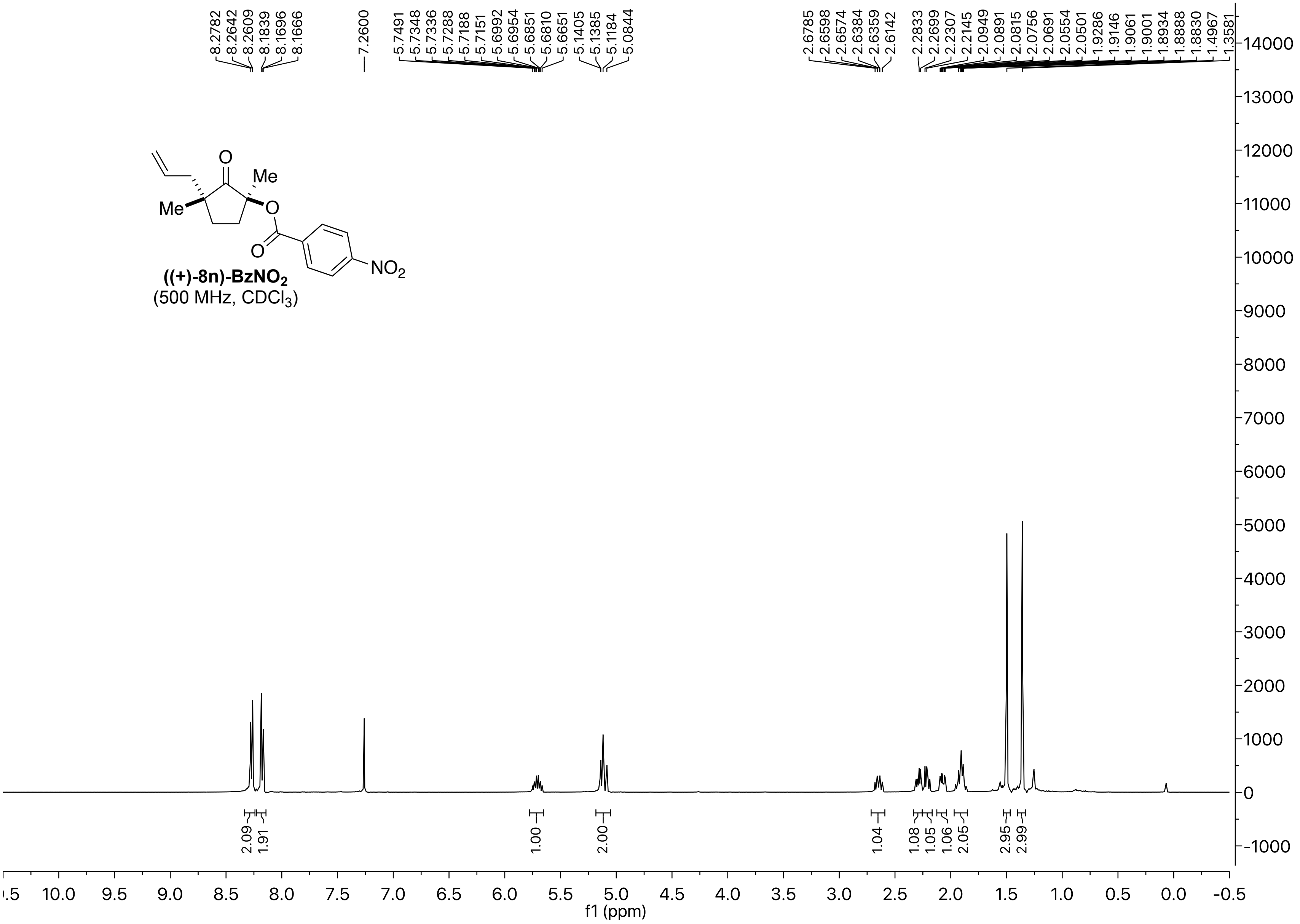

((+)-8n)-BzNO$_2$
(500 MHz, CDCl$_3$)

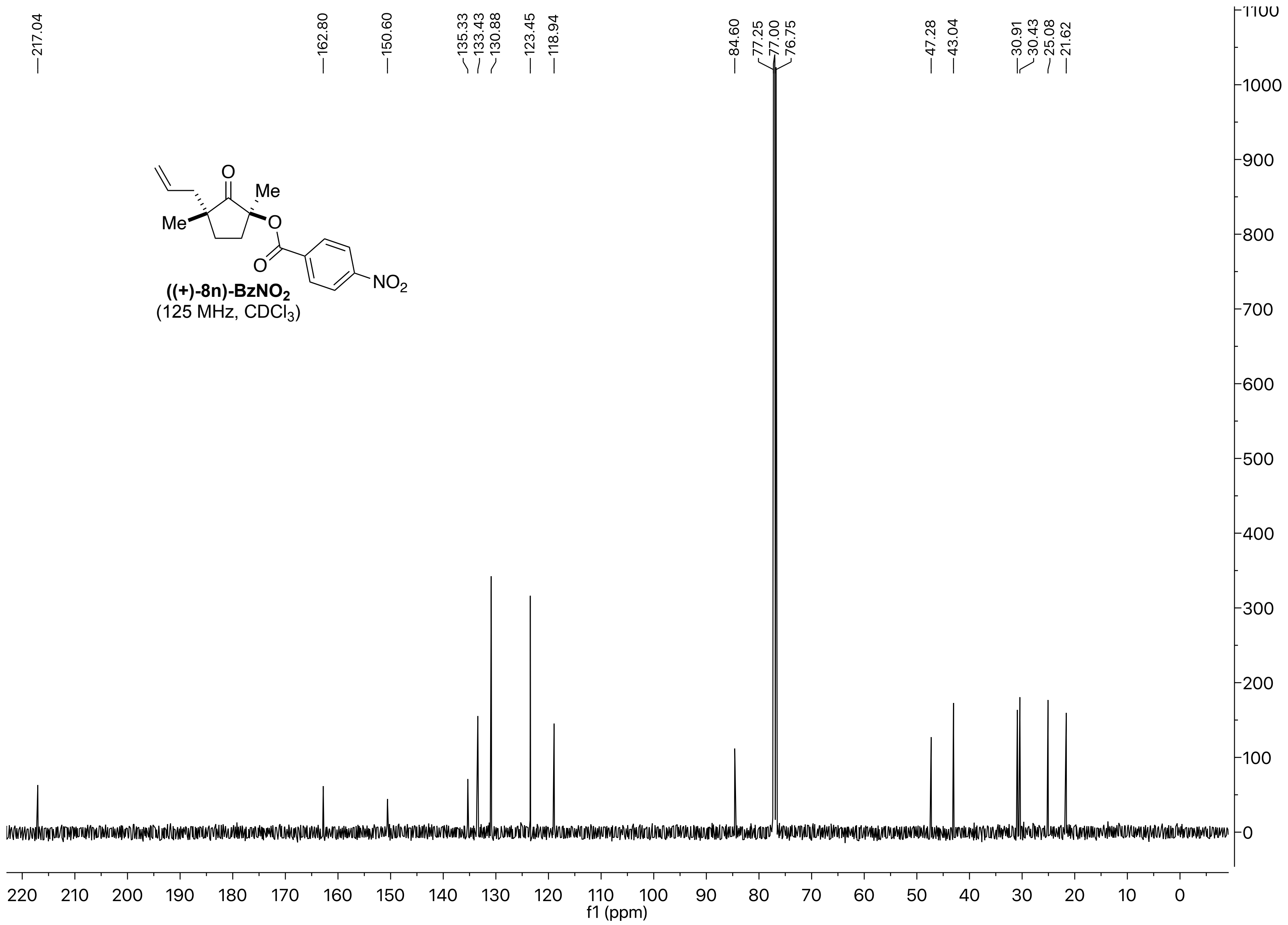
217.04
162.80
150.60
135.33
133.43
130.88
123.45
118.94
84.60
77.25
77.00
76.75
47.28
43.04
30.91
30.43
25.08
21.62
O
Me
Me
O
O
NO_2
((+)-8n)-BzNO$_2$
(125 MHz, CDCl$_3$)
f1 (ppm)

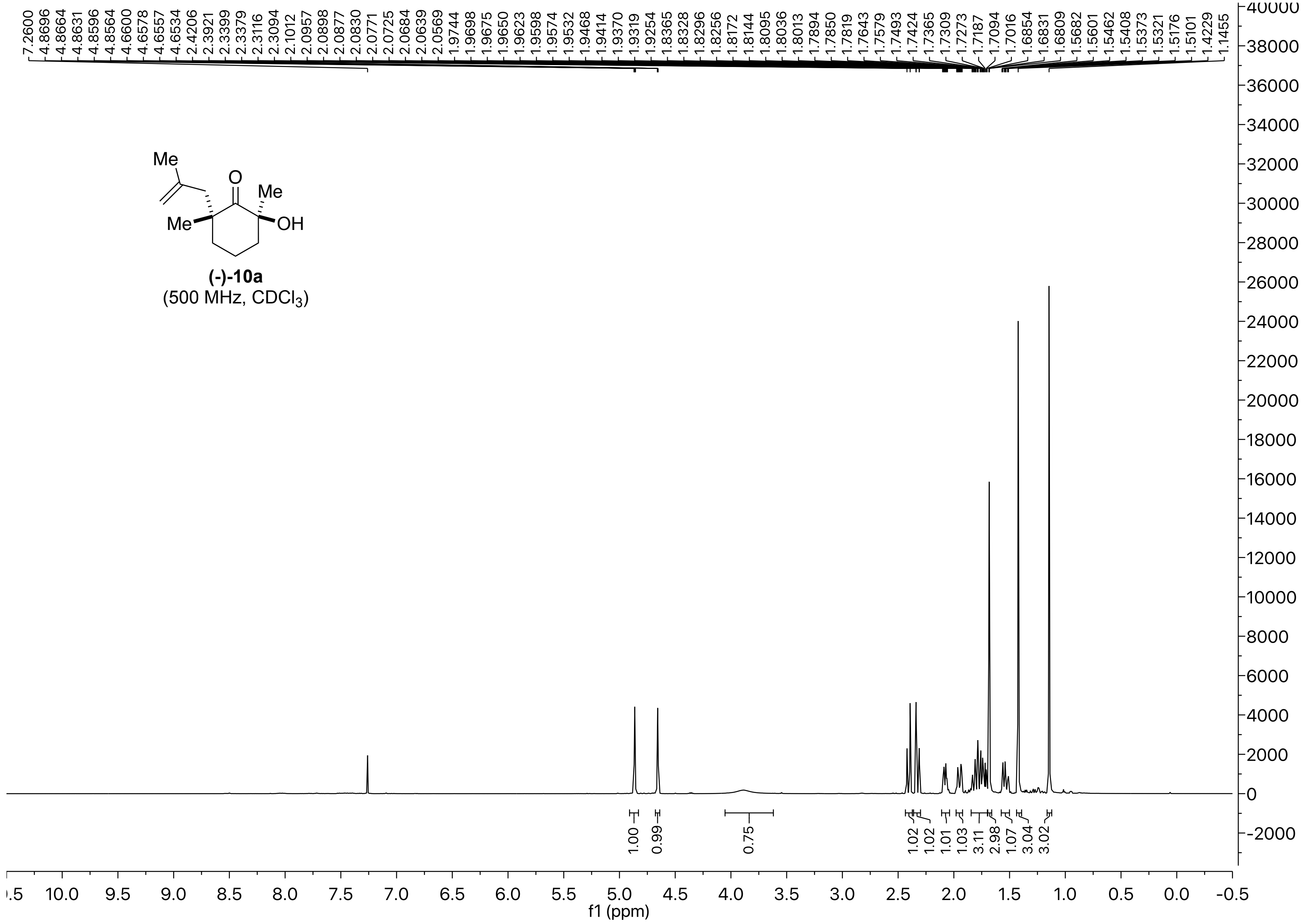

Me
O
Me
Me
OH
(-)-10a
(500 MHz, CDCl$_3$)
f1 (ppm)

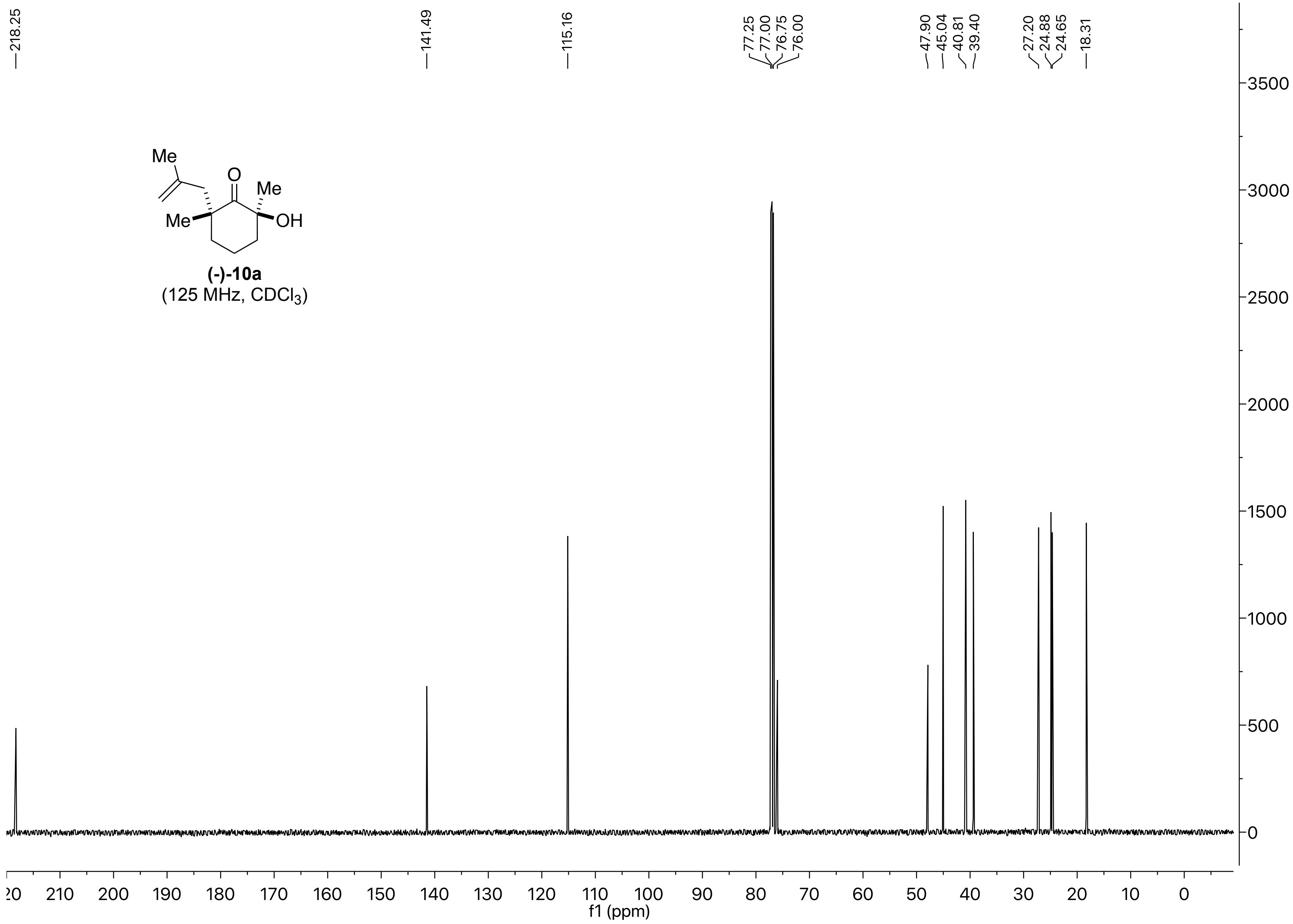
218.25
141.49
115.16
77.25
77.00
76.75
76.00
47.90
45.04
40.81
39.40
27.20
24.88
24.65
18.31
Me
O
Me
Me
OH
(-)-10a
(125 MHz, CDCl3)
f1 (ppm)

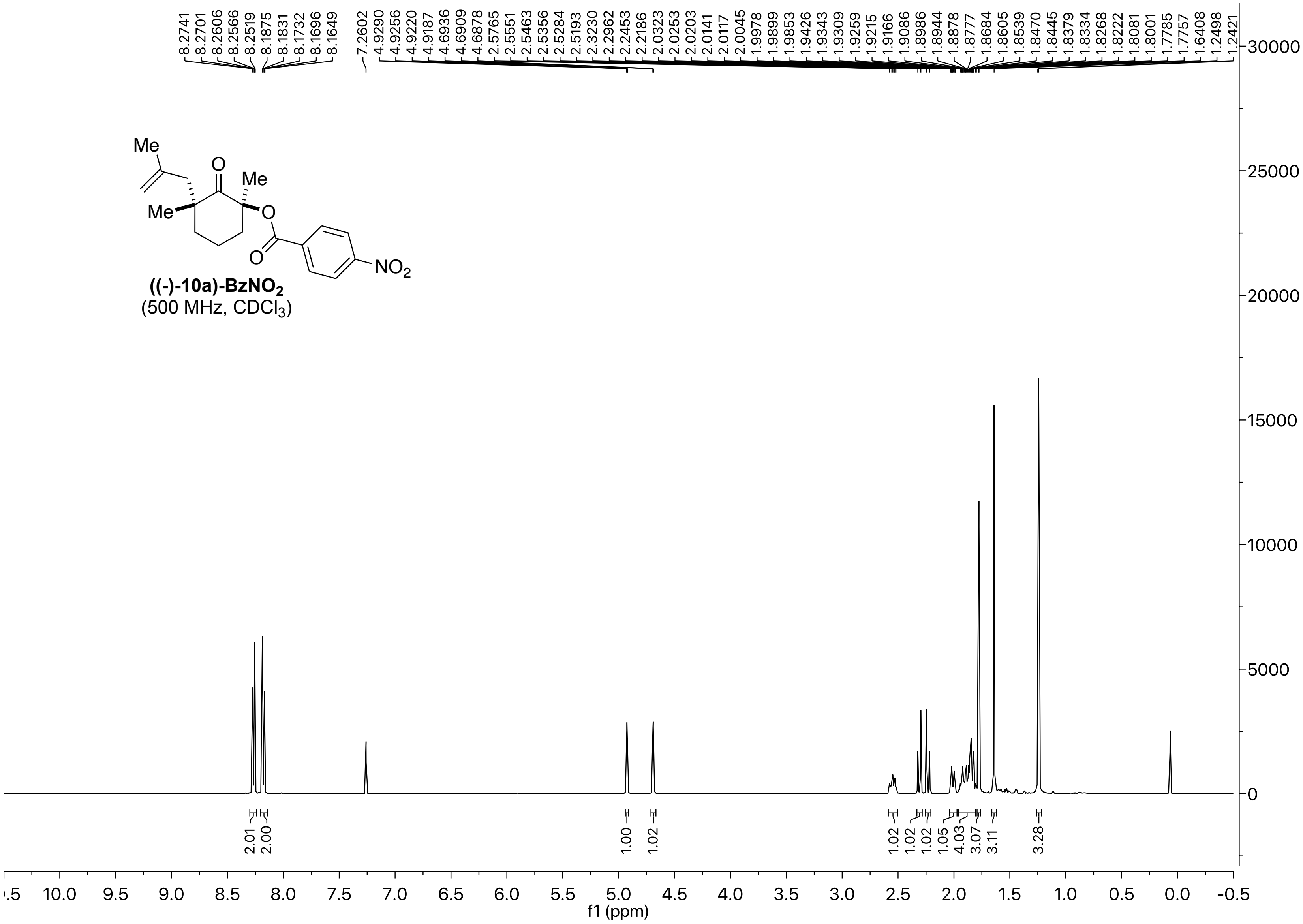
((-)-10a)-BzNO2
(500 MHz, CDCl3)
f1 (ppm)

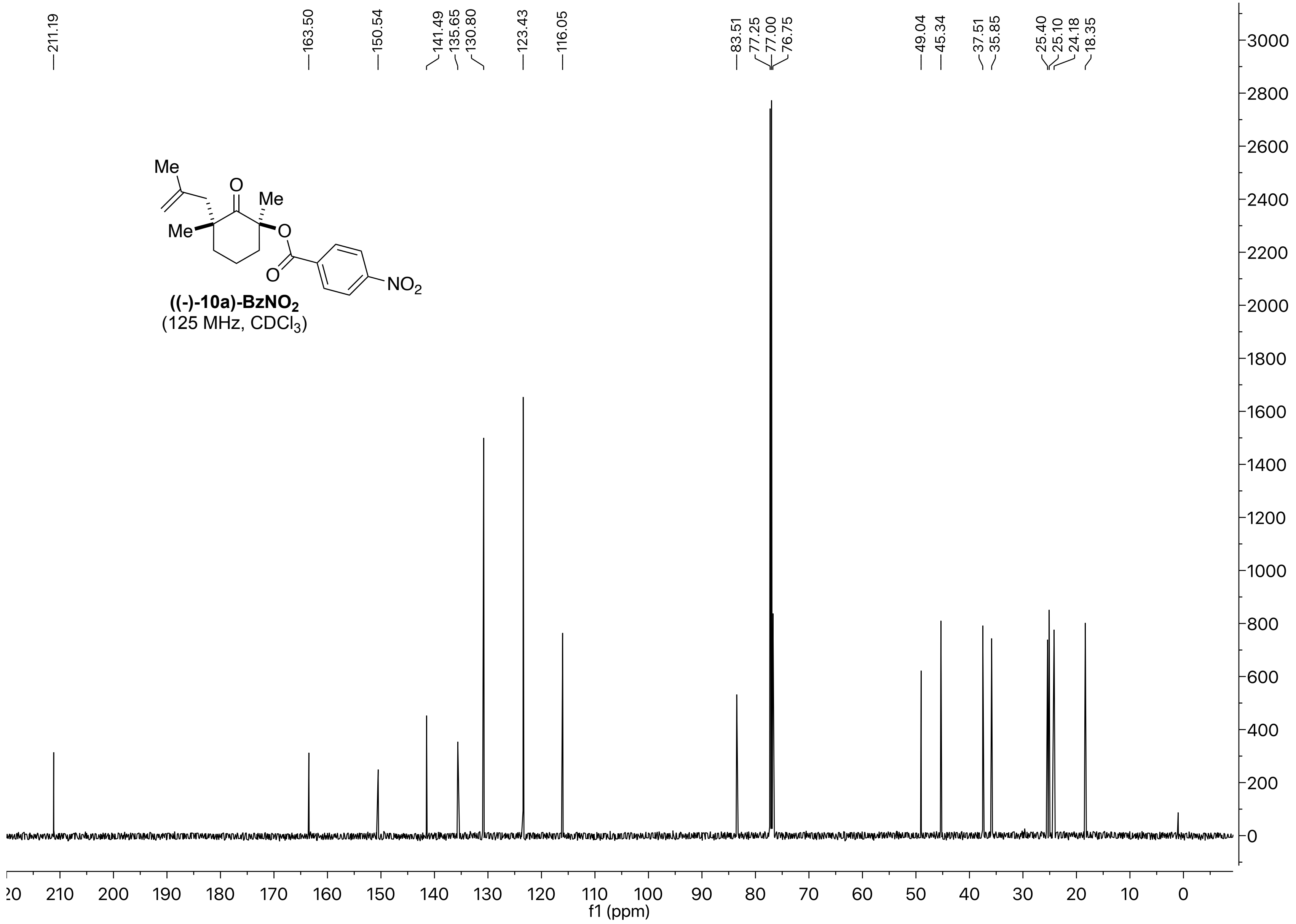
211.19
163.50
150.54
141.49
135.65
130.80
123.43
116.05
83.51
77.25
77.00
76.75
49.04
45.34
37.51
35.85
25.40
25.10
24.18
18.35
Me
O
Me
Me
O
O
NO2
((-)-10a)-BzNO2
(125 MHz, CDCl3)
f1 (ppm)

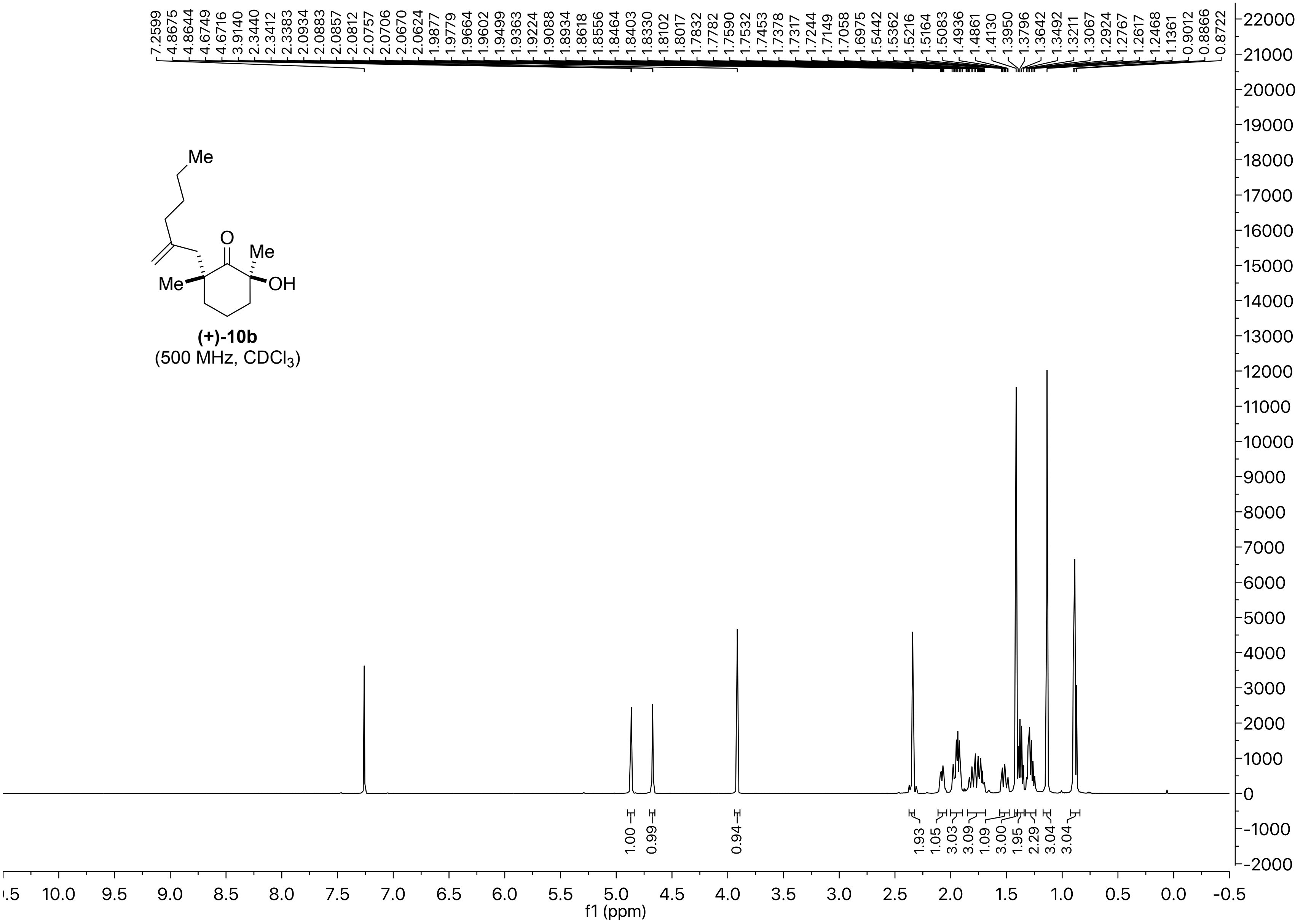
Me
O
Me
Me
OH
(+)-10b
(500 MHz, CDCl3)
f1 (ppm)

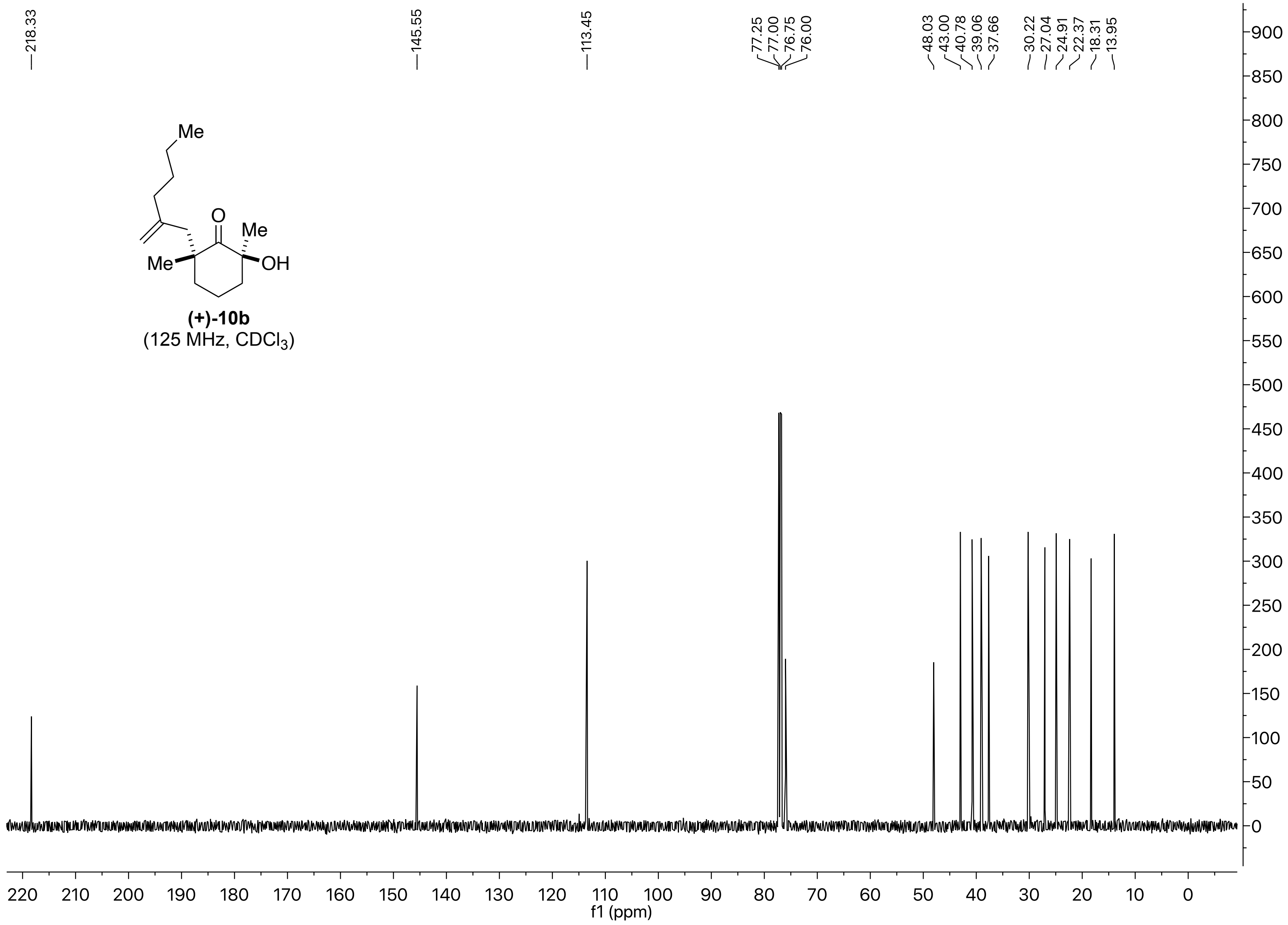
218.33
145.55
113.45
77.25
77.00
76.75
76.00
48.03
43.00
40.78
39.06
37.66
30.22
27.04
24.91
22.37
18.31
13.95
Me
O
Me
Me
OH
(+)-10b
(125 MHz, CDCl3)
f1 (ppm)

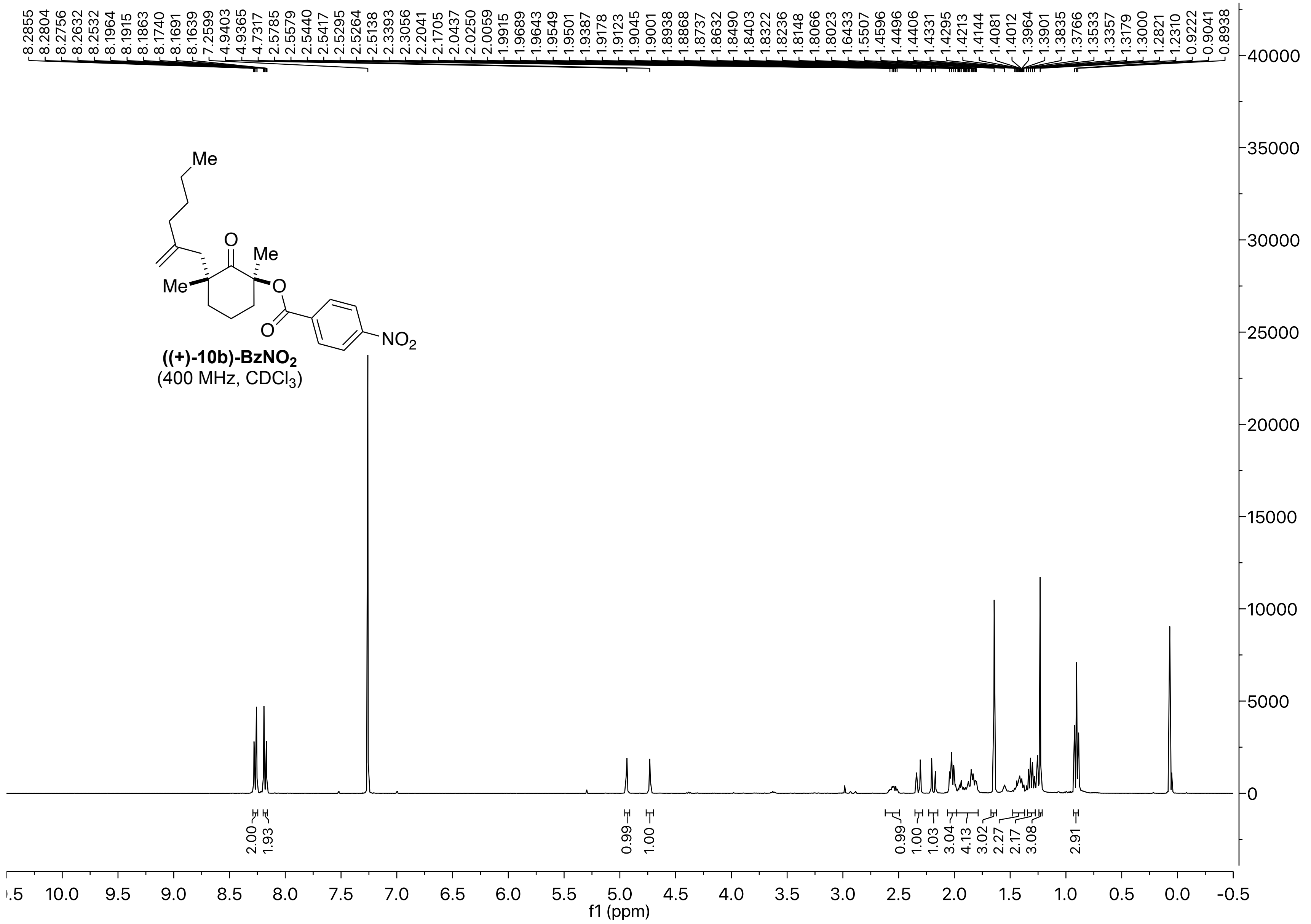

Me
O
Me
Me
O
O
NO2
((+)-10b)-BzNO2
(400 MHz, CDCl3)
f1 (ppm)

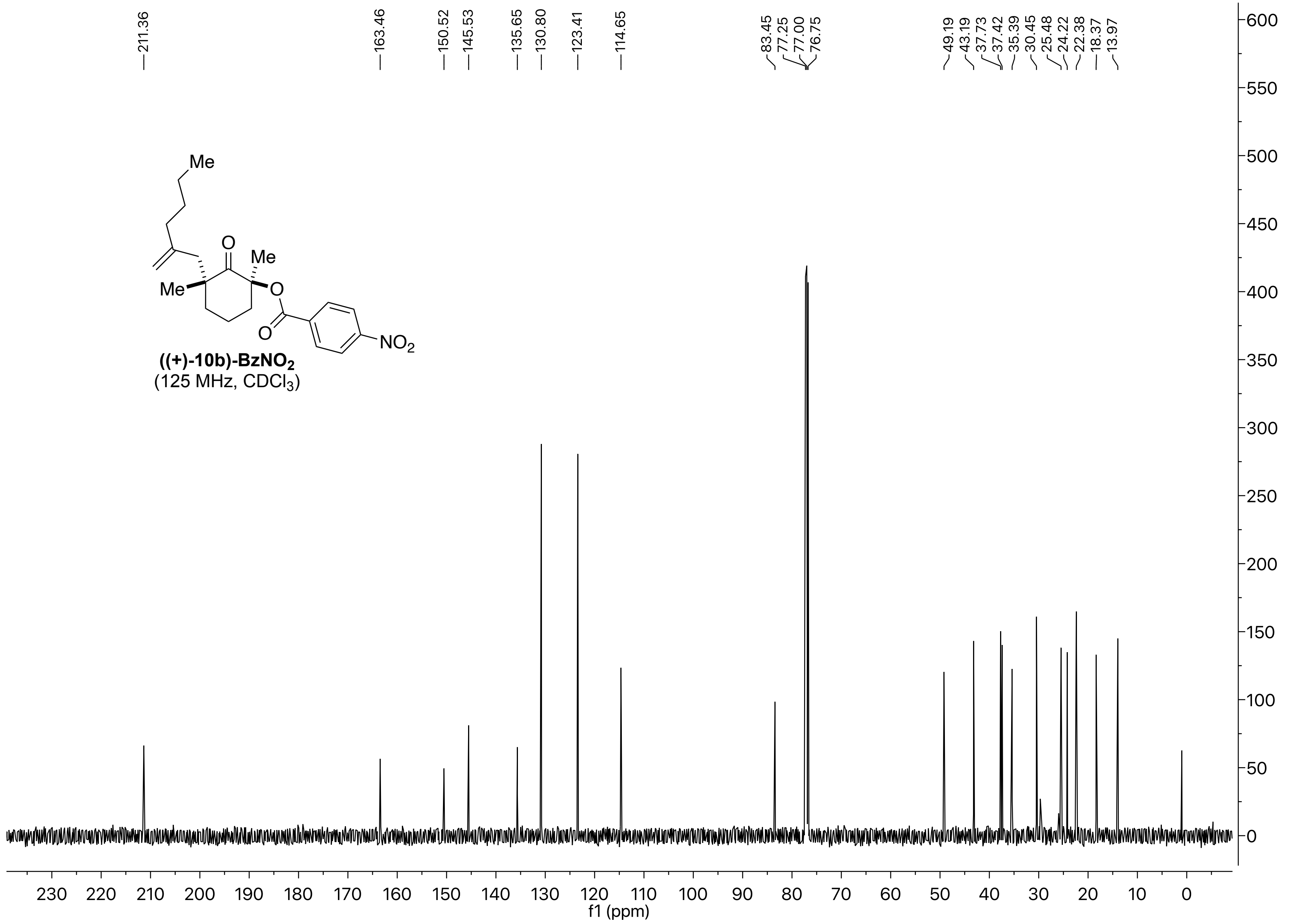
211.36
163.46
150.52
145.53
135.65
130.80
123.41
114.65
83.45
77.25
77.00
76.75
49.19
43.19
37.73
37.42
35.39
30.45
25.48
24.22
22.38
18.37
13.97
Me
O
Me
Me
O
O
O
NO2
((+)-10b)-BzNO2
(125 MHz, CDCl3)
f1 (ppm)

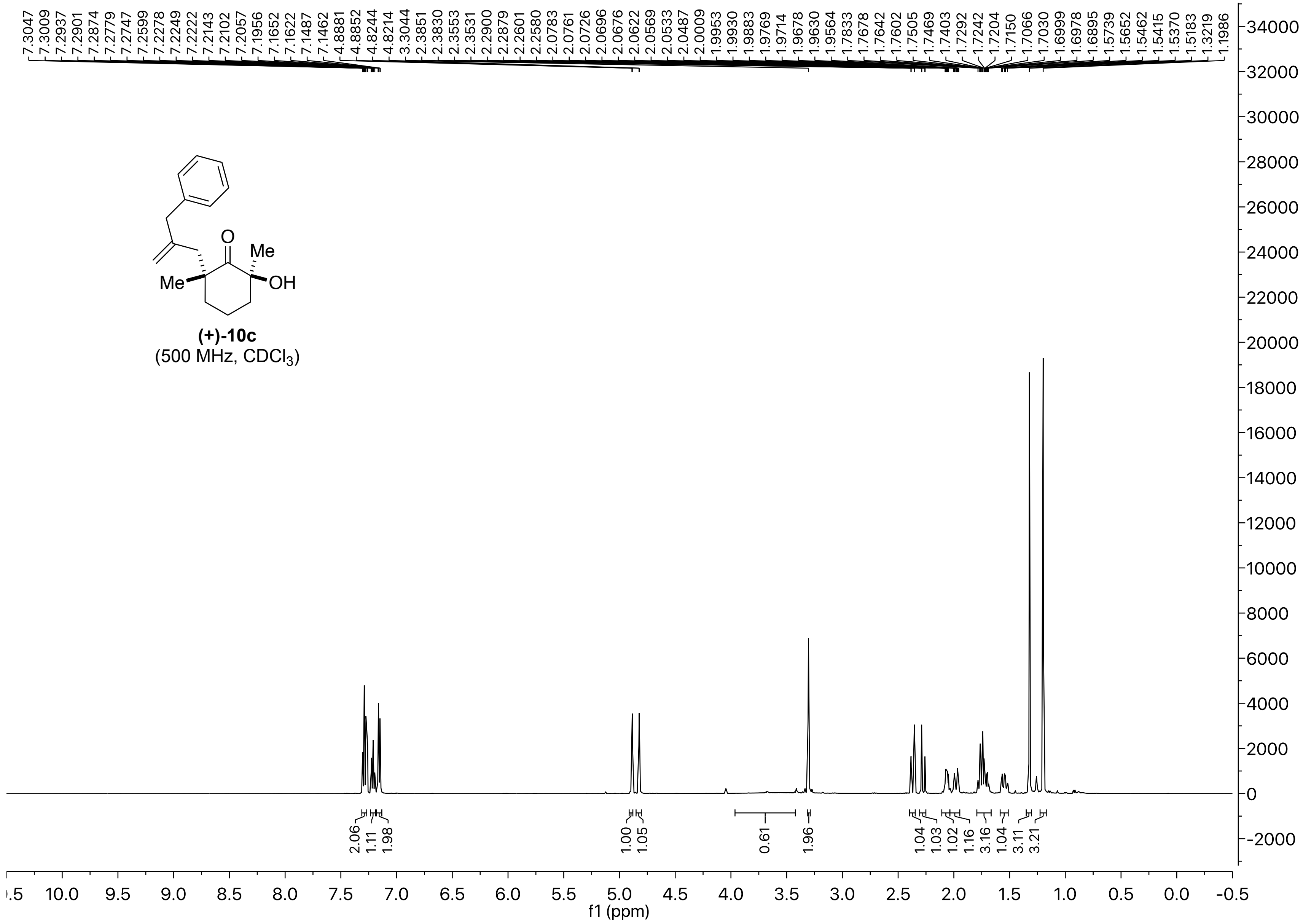
(+)-10c
(500 MHz, CDCl3)
Me
OH
O
f1 (ppm)

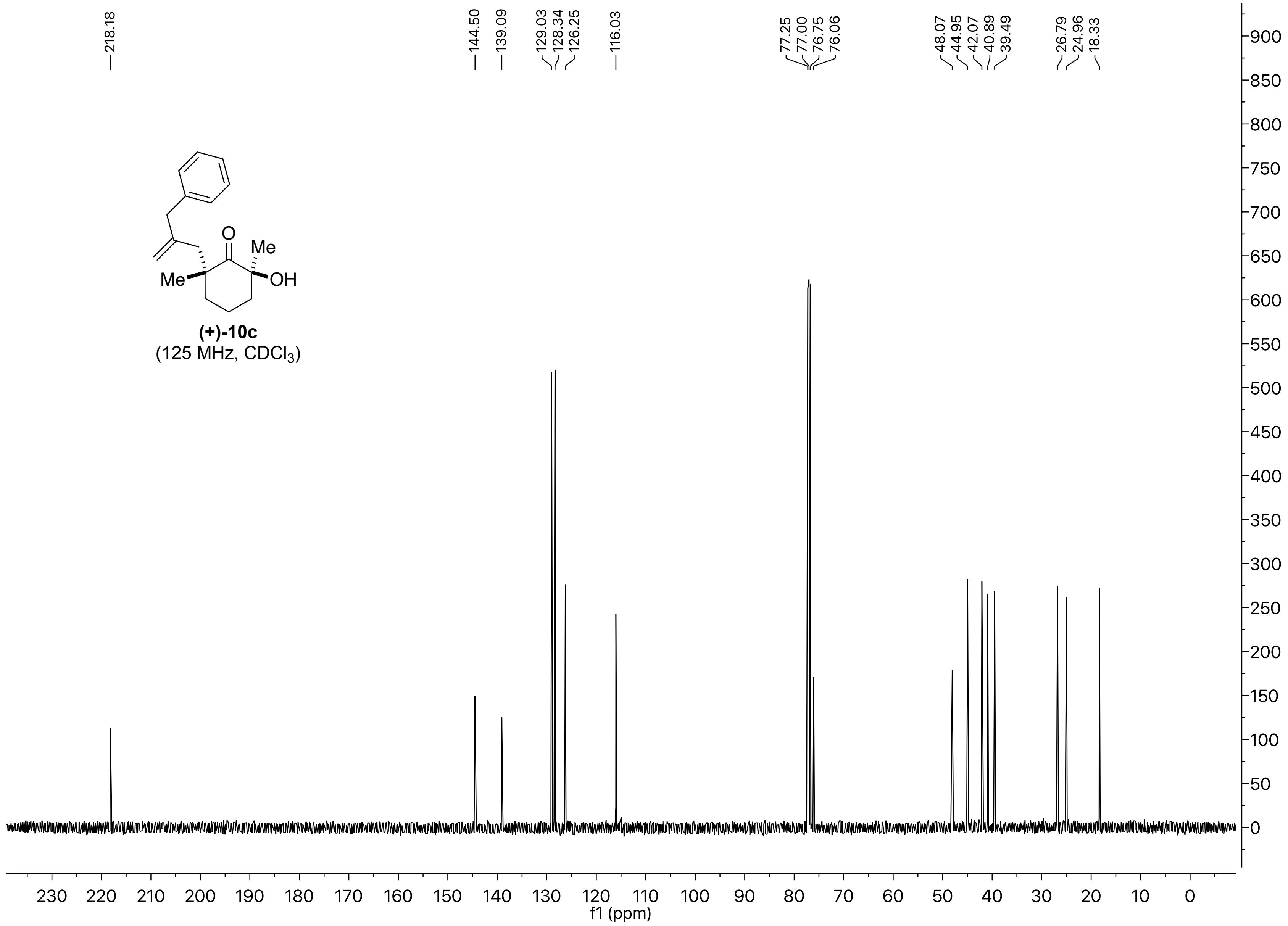

218.18
144.50
139.09
129.03
128.34
126.25
116.03
77.25
77.00
76.75
76.06
48.07
44.95
42.07
40.89
39.49
26.79
24.96
18.33
O
Me
OH
Me
(+)-10c
(125 MHz, CDCl3)
f1 (ppm)

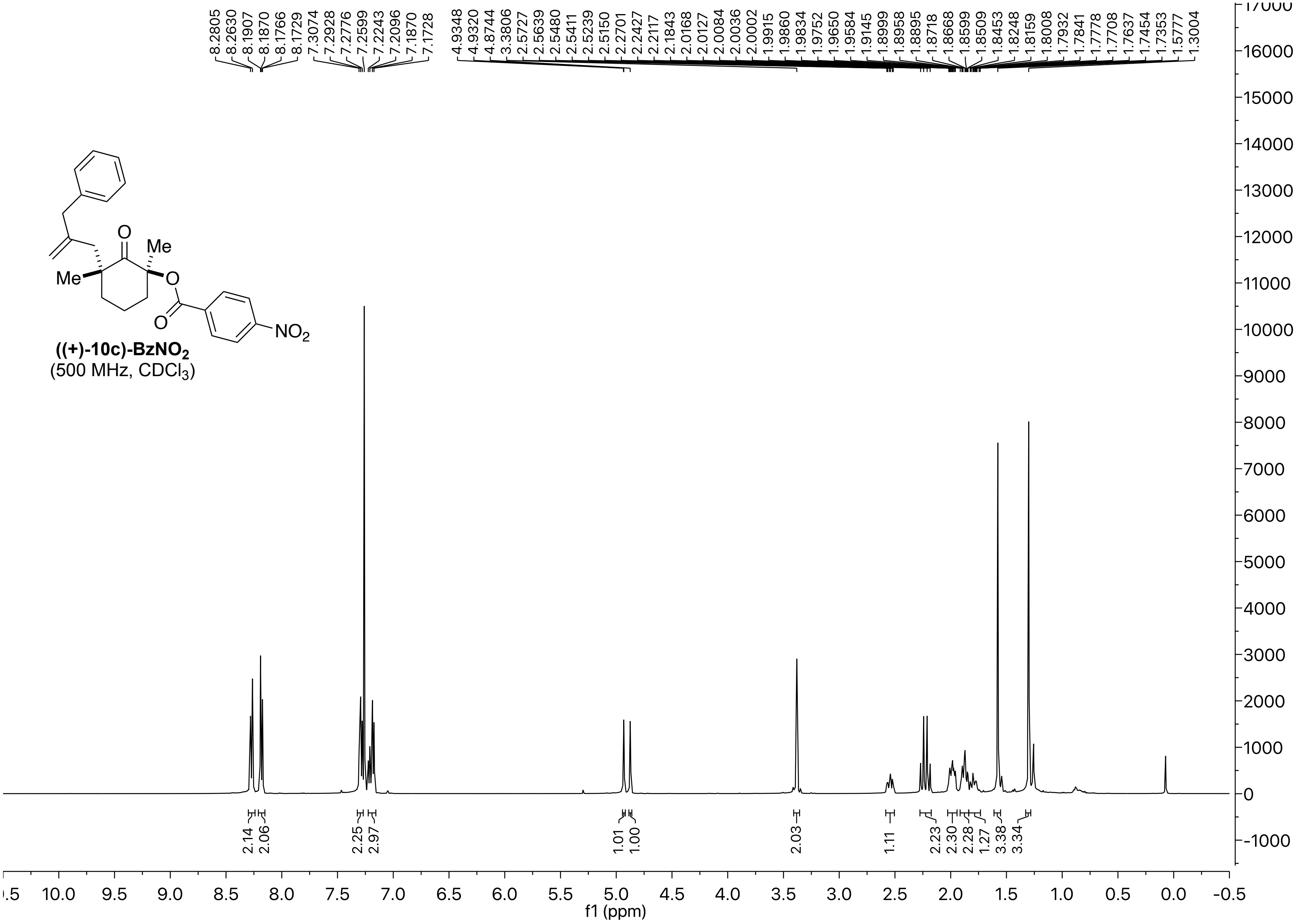

((+)-10c)-BzNO2
(500 MHz, CDCl3)
f1 (ppm)

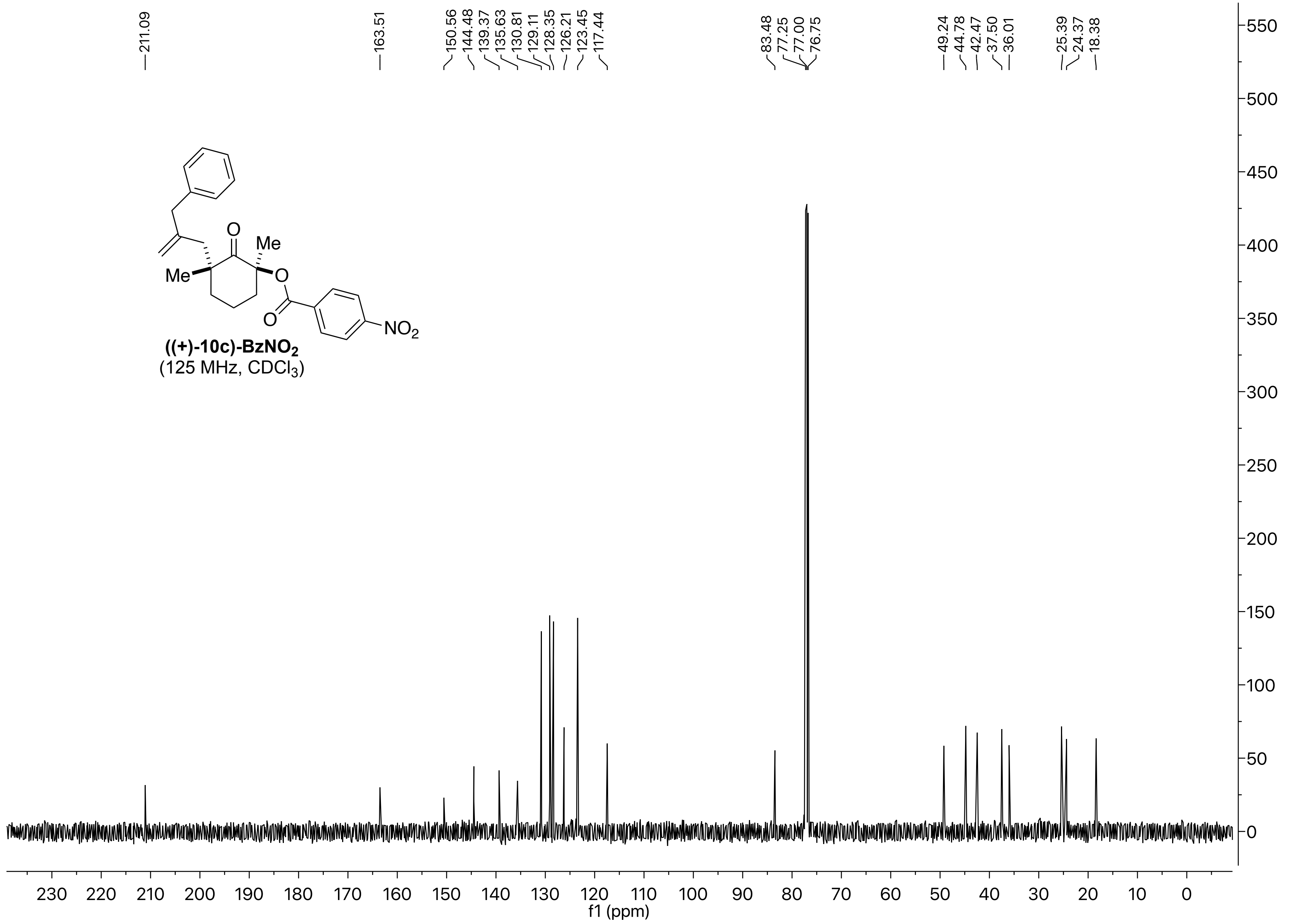

211.09
163.51
150.56
144.48
139.37
135.63
130.81
129.11
128.35
126.21
123.45
117.44
83.48
77.25
77.00
76.75
49.24
44.78
42.47
37.50
36.01
25.39
24.37
18.38
Me
O
Me
O
O
NO2
((+)-10c)-BzNO2
(125 MHz, CDCl3)
f1 (ppm)

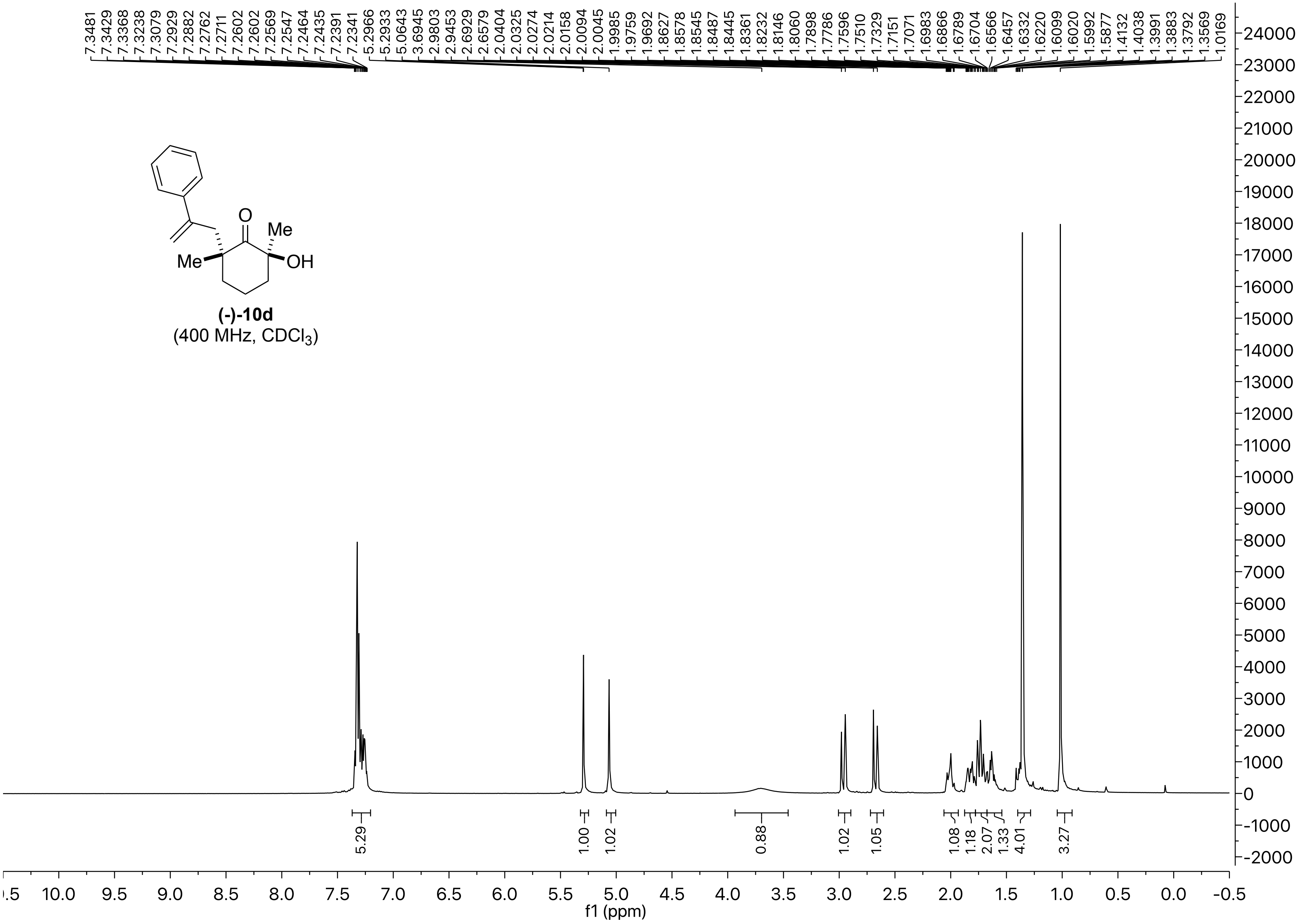
7.3481
7.3429
7.3368
7.3238
7.3079
7.2929
7.2882
7.2762
7.2711
7.2602
7.2602
7.2569
7.2547
7.2464
7.2435
7.2391
7.2341
5.2966
5.2933
5.0643
3.6945
2.9803
2.9453
2.6929
2.6579
2.0404
2.0325
2.0274
2.0214
2.0158
2.0094
2.0045
1.9985
1.9759
1.9692
1.8627
1.8578
1.8545
1.8487
1.8445
1.8361
1.8232
1.8146
1.8060
1.7898
1.7786
1.7596
1.7510
1.7329
1.7151
1.7071
1.6983
1.6866
1.6789
1.6704
1.6566
1.6457
1.6332
1.6220
1.6099
1.6020
1.5992
1.5877
1.4132
1.4038
1.3991
1.3883
1.3792
1.3569
1.0169
O
Me
Me
OH
(-)-10d
(400 MHz, CDCl3)
5.29
1.00
1.02
0.88
1.02
1.05
1.08
1.18
2.07
1.33
4.01
3.27
f1 (ppm)

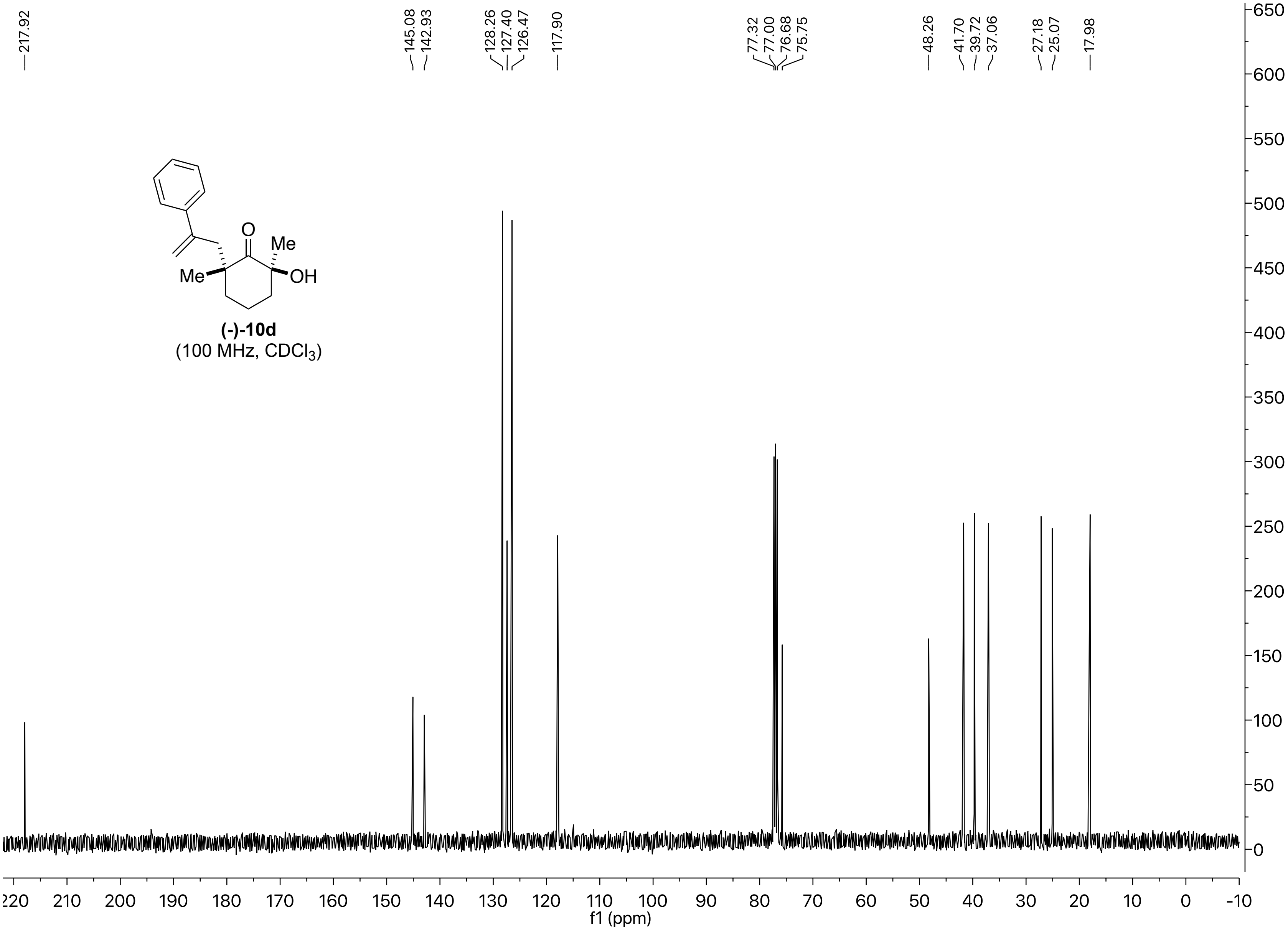

217.92
145.08
142.93
128.26
127.40
126.47
117.90
77.32
77.00
76.68
75.75
48.26
41.70
39.72
37.06
27.18
25.07
17.98
O
Me
Me
OH
(-)-10d
(100 MHz, CDCl3)
f1 (ppm)

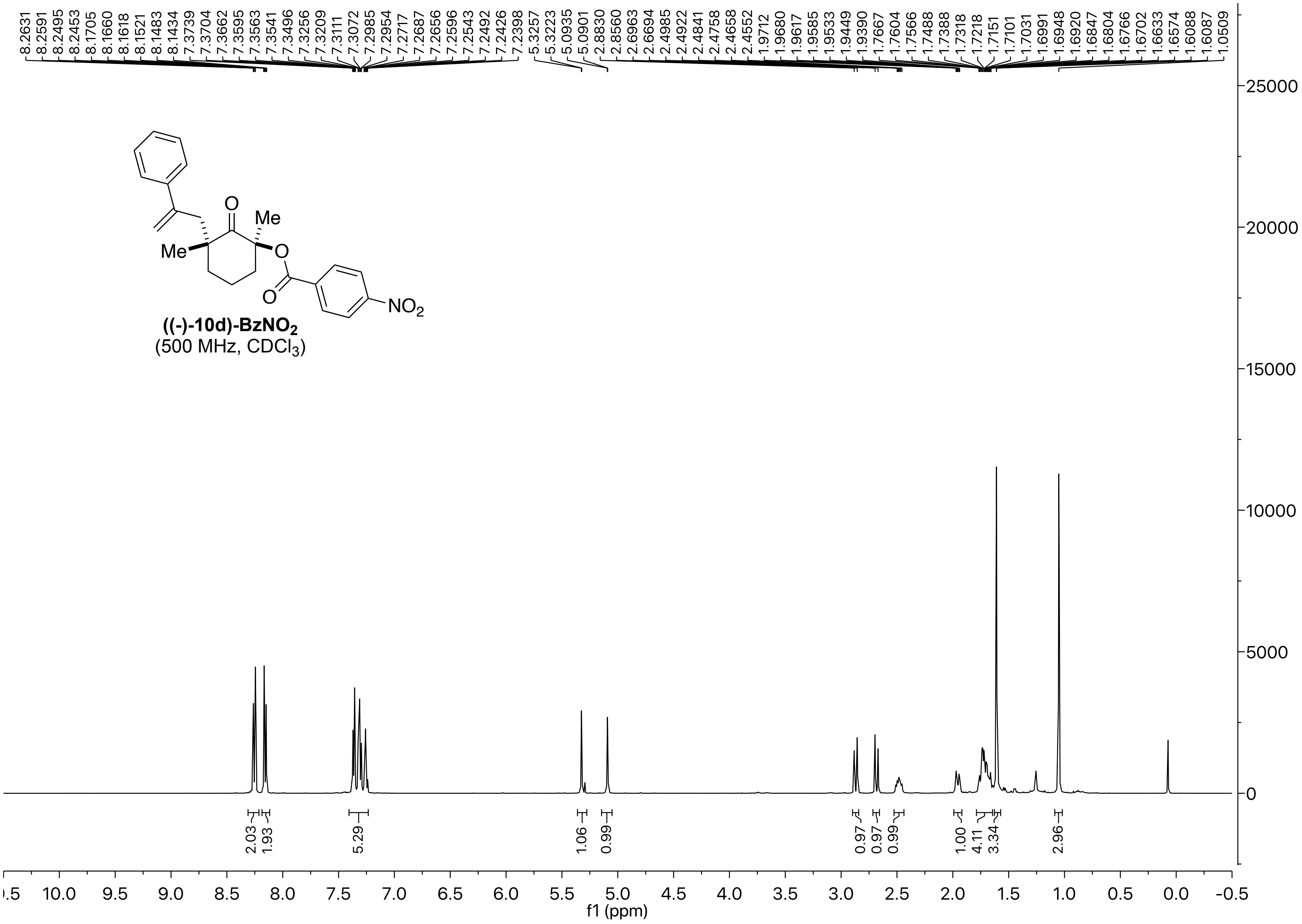

((-)-10d)-BzNO2
(500 MHz, CDCl3)
f1 (ppm)

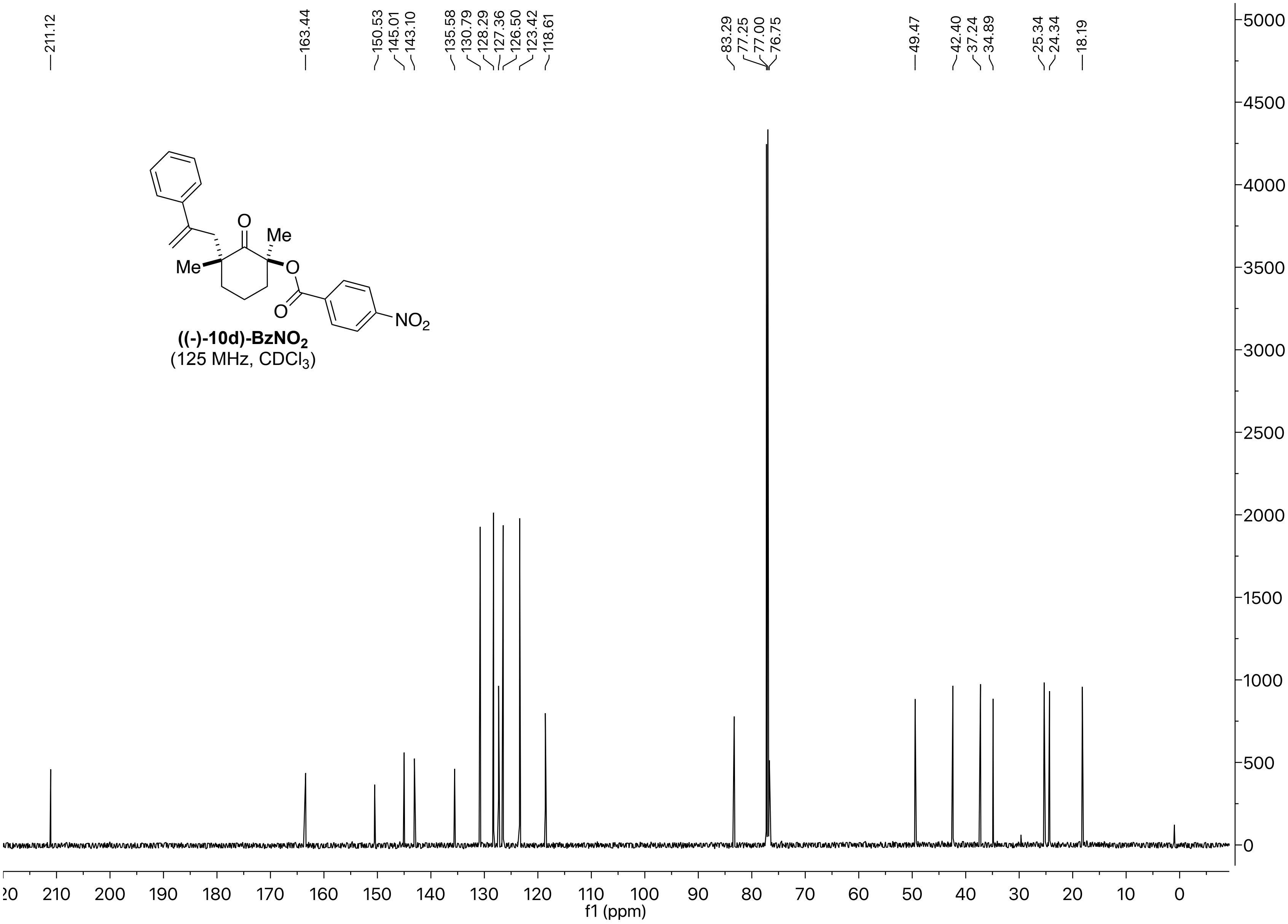

211.12
163.44
150.53
145.01
143.10
135.58
130.79
128.29
127.36
126.50
123.42
118.61
83.29
77.25
77.00
76.75
49.47
42.40
37.24
34.89
25.34
24.34
18.19
Me
O
Me
O
O
NO2
((-)-10d)-BzNO2
(125 MHz, CDCl3)
f1 (ppm)

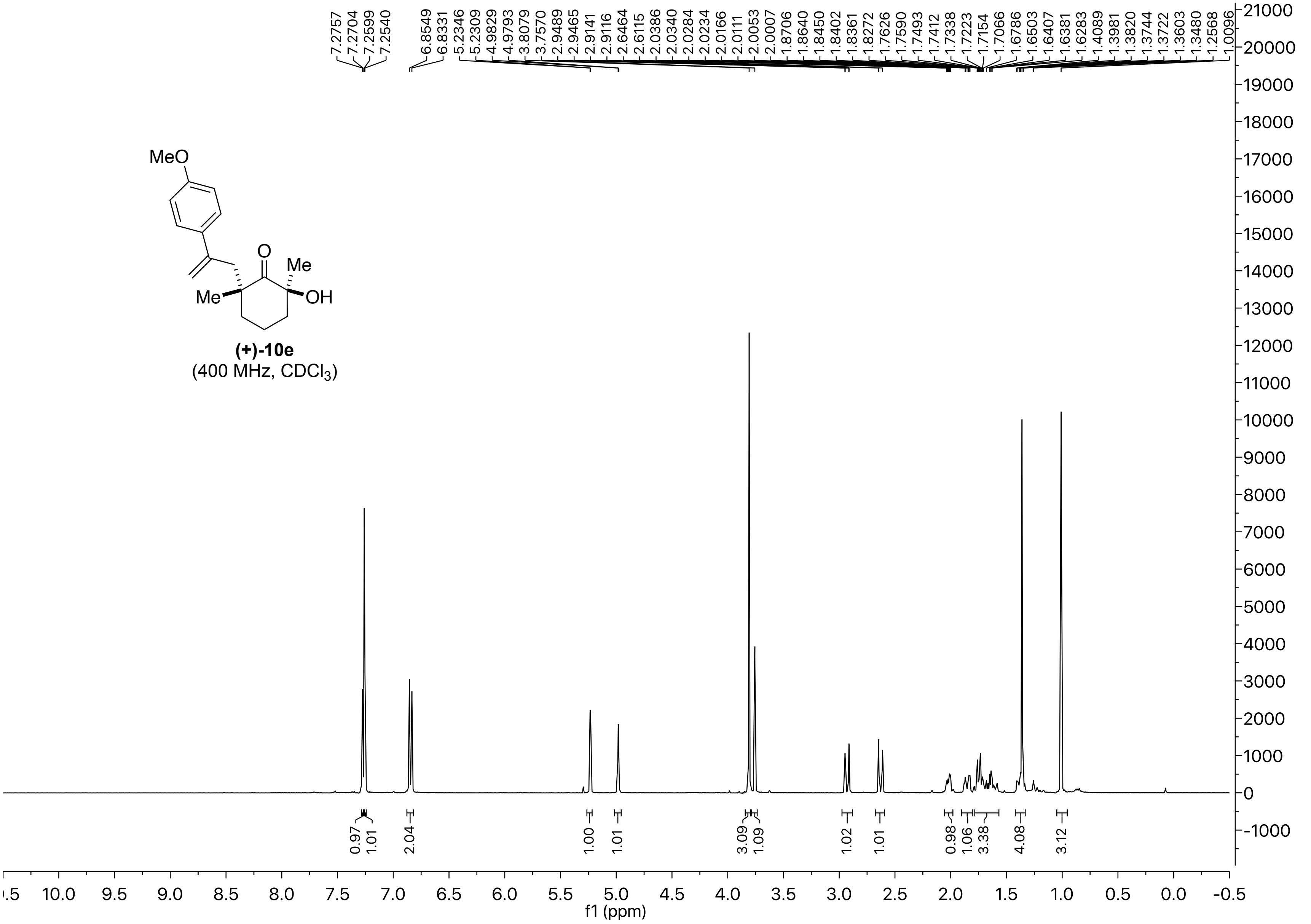
(+)-10e
(400 MHz, CDCl3)
f1 (ppm)

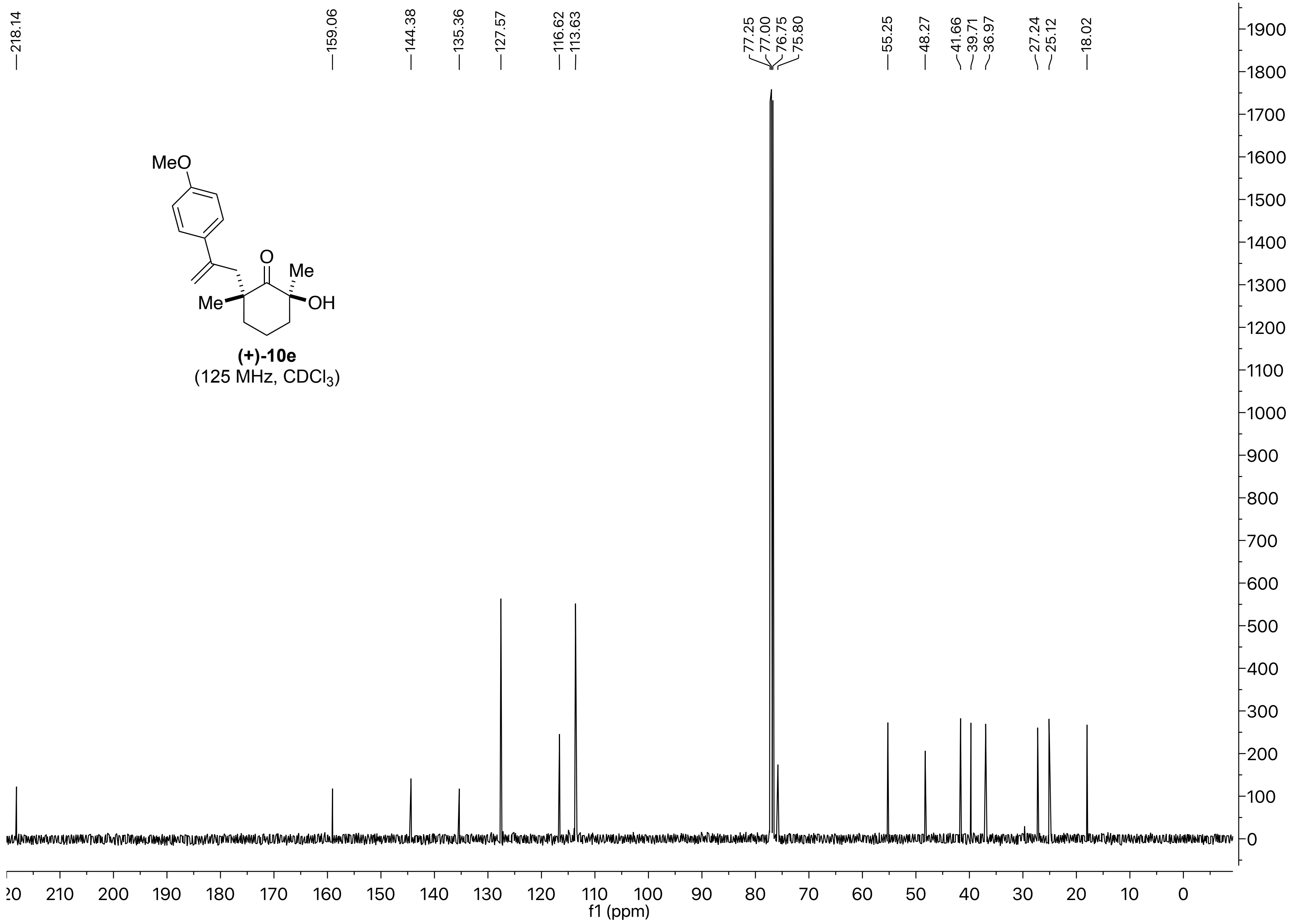
218.14
159.06
144.38
135.36
127.57
116.62
113.63
77.25
77.00
76.75
75.80
55.25
48.27
41.66
39.71
36.97
27.24
25.12
18.02
MeO
O
Me
Me
OH
(+)-10e
(125 MHz, CDCl3)
f1 (ppm)

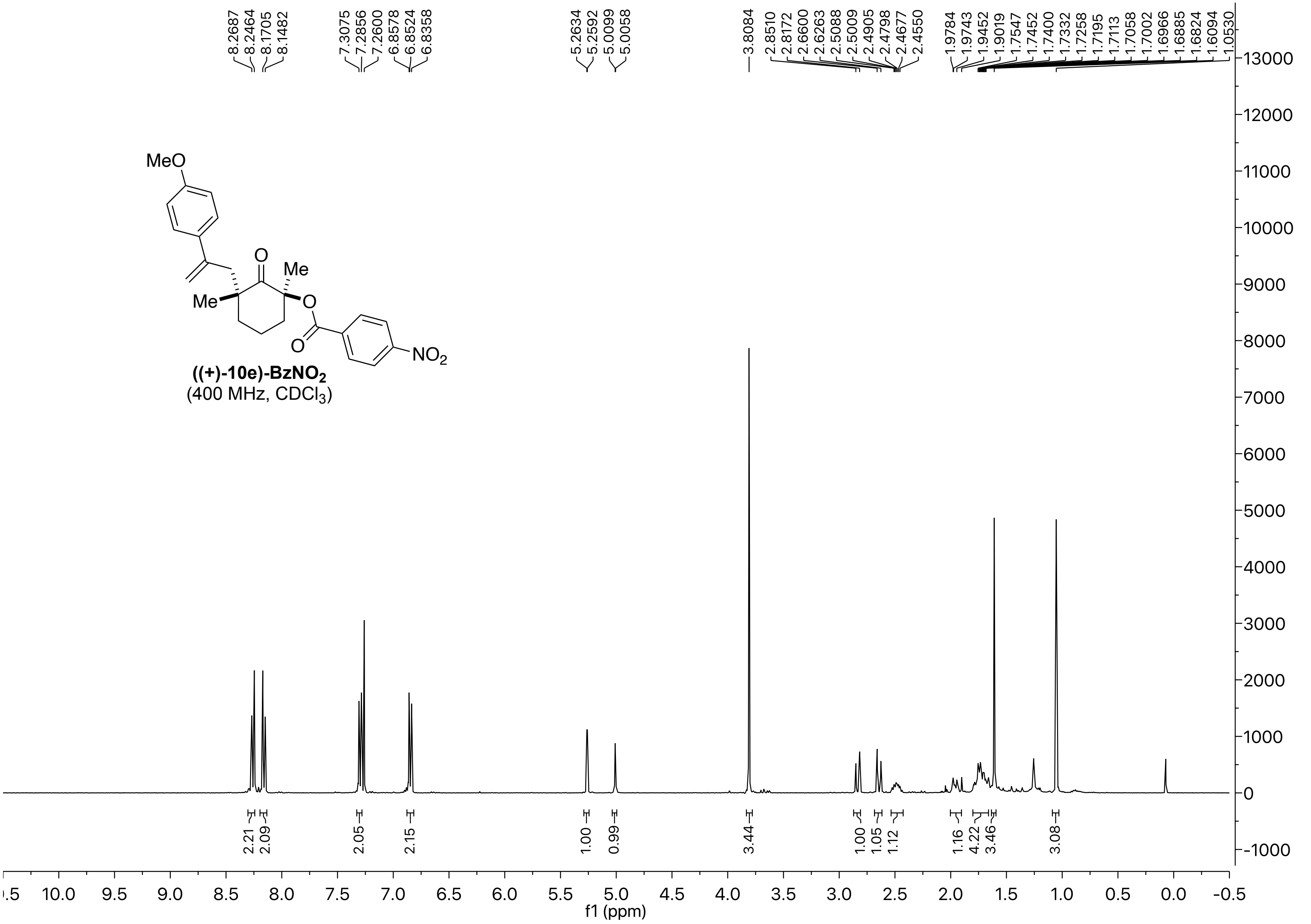

((+)-10e)-BzNO$_2$
(400 MHz, $CDCl_3$)

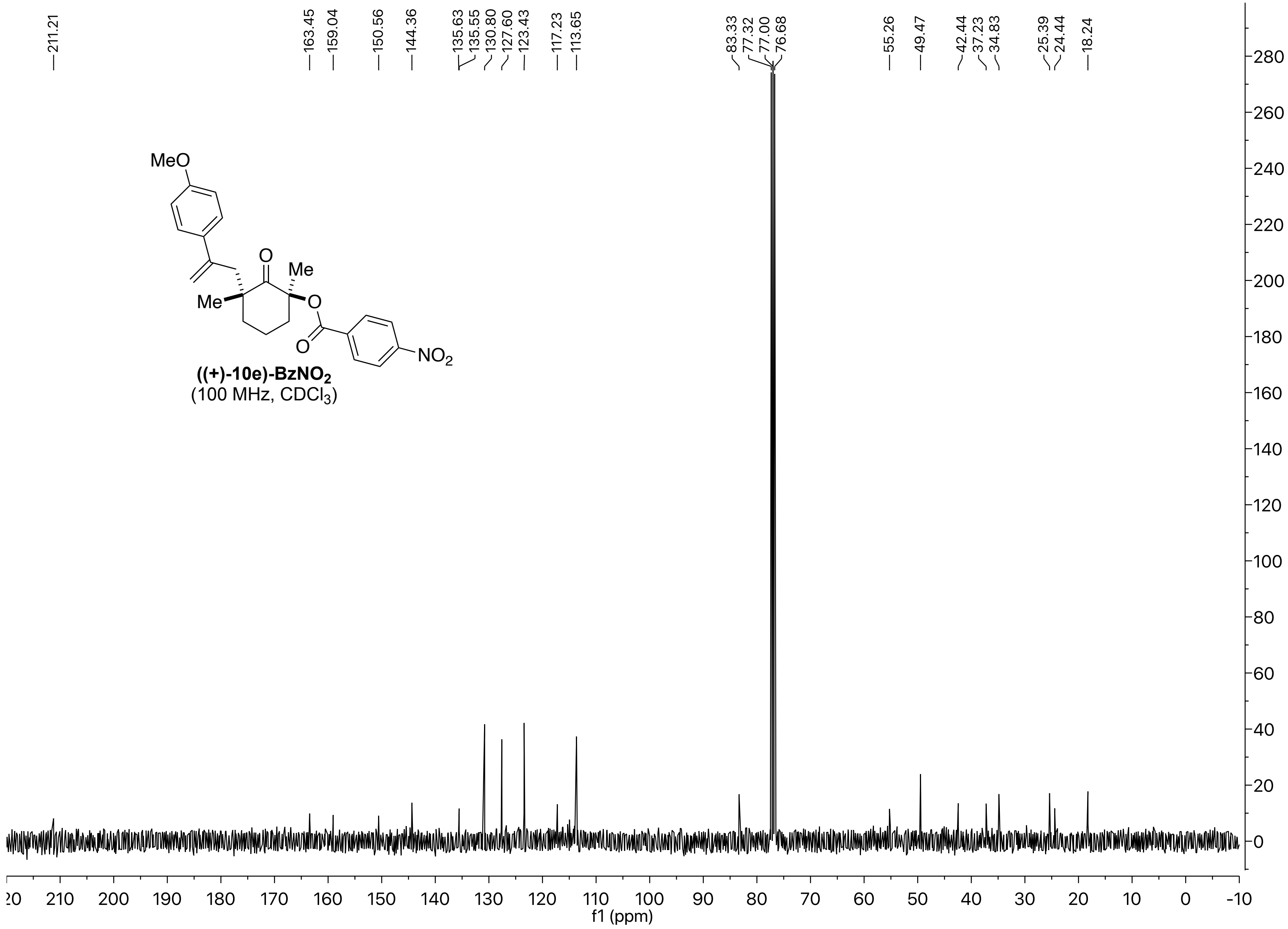
211.21
163.45
159.04
150.56
144.36
135.63
135.55
130.80
127.60
123.43
117.23
113.65
83.33
77.32
77.00
76.68
55.26
49.47
42.44
37.23
34.83
25.39
24.44
18.24
MeO
O
Me
Me
O
O
NO2
((+)-10e)-BzNO2
(100 MHz, CDCl3)
f1 (ppm)

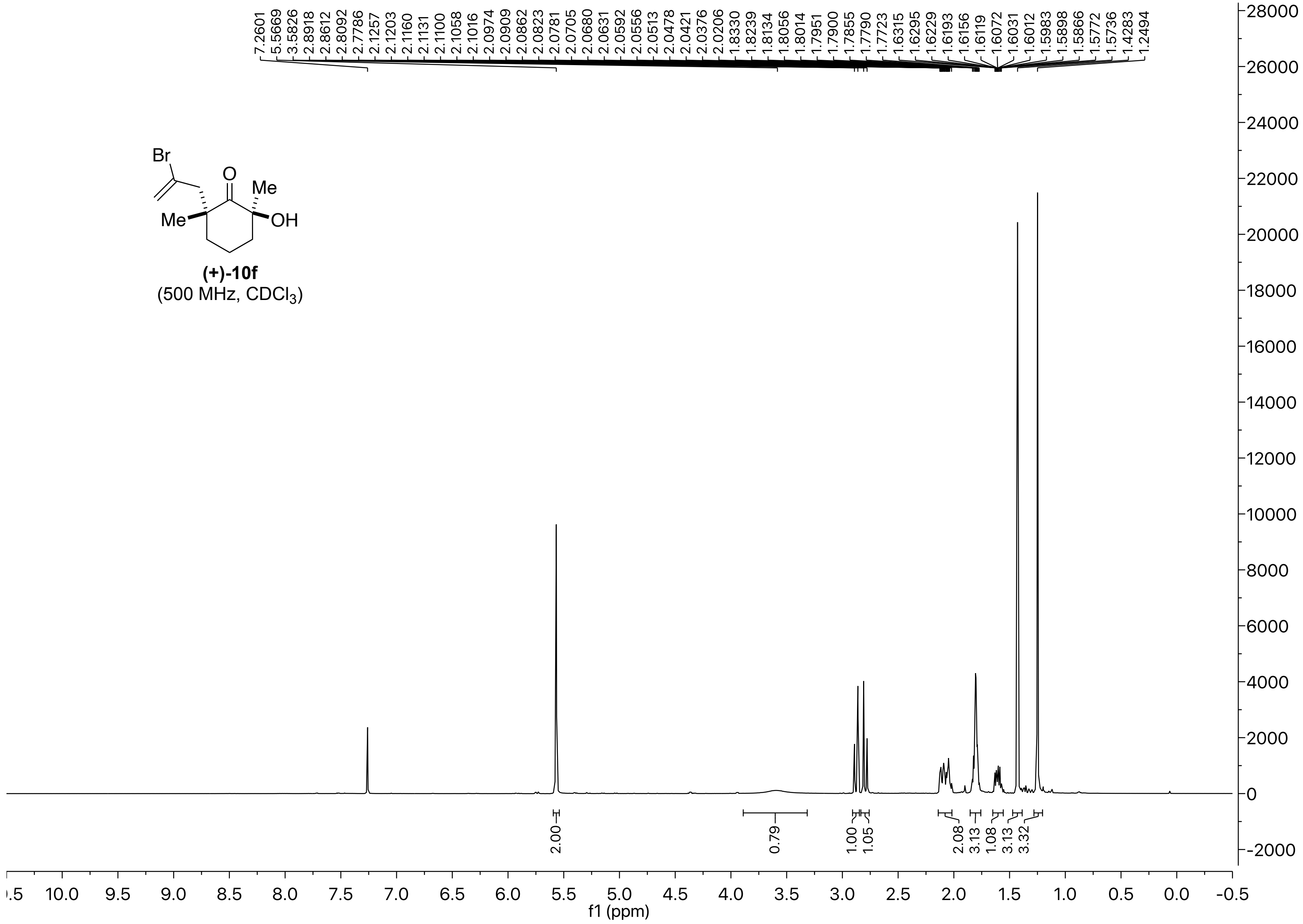

Br
O
Me
Me
OH
(+)-10f
(500 MHz, CDCl3)
f1 (ppm)

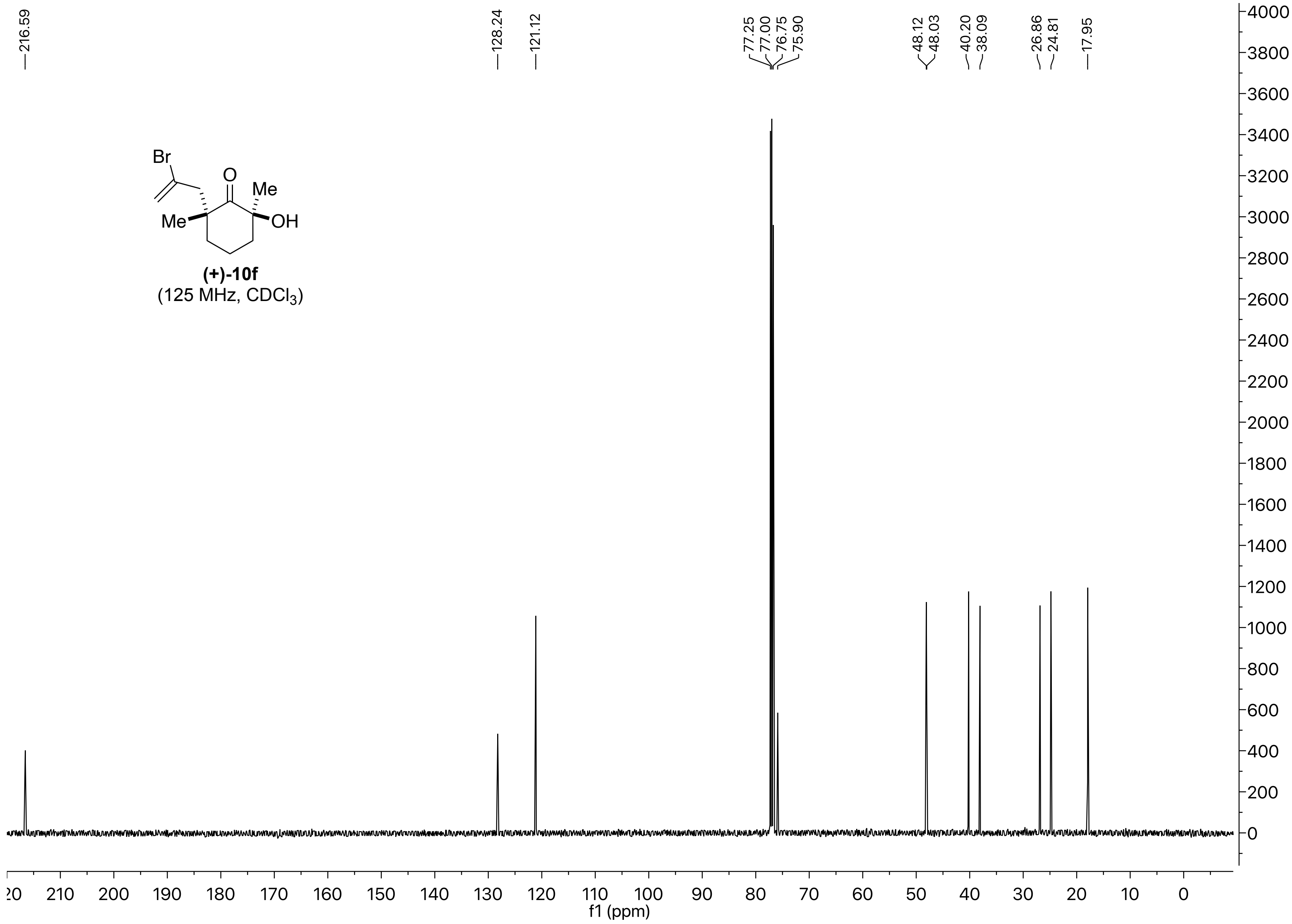

216.59
128.24
121.12
77.25
77.00
76.75
75.90
48.12
48.03
40.20
38.09
26.86
24.81
17.95
Br
O
Me
Me
OH
(+)-10f
(125 MHz, CDCl3)
f1 (ppm)

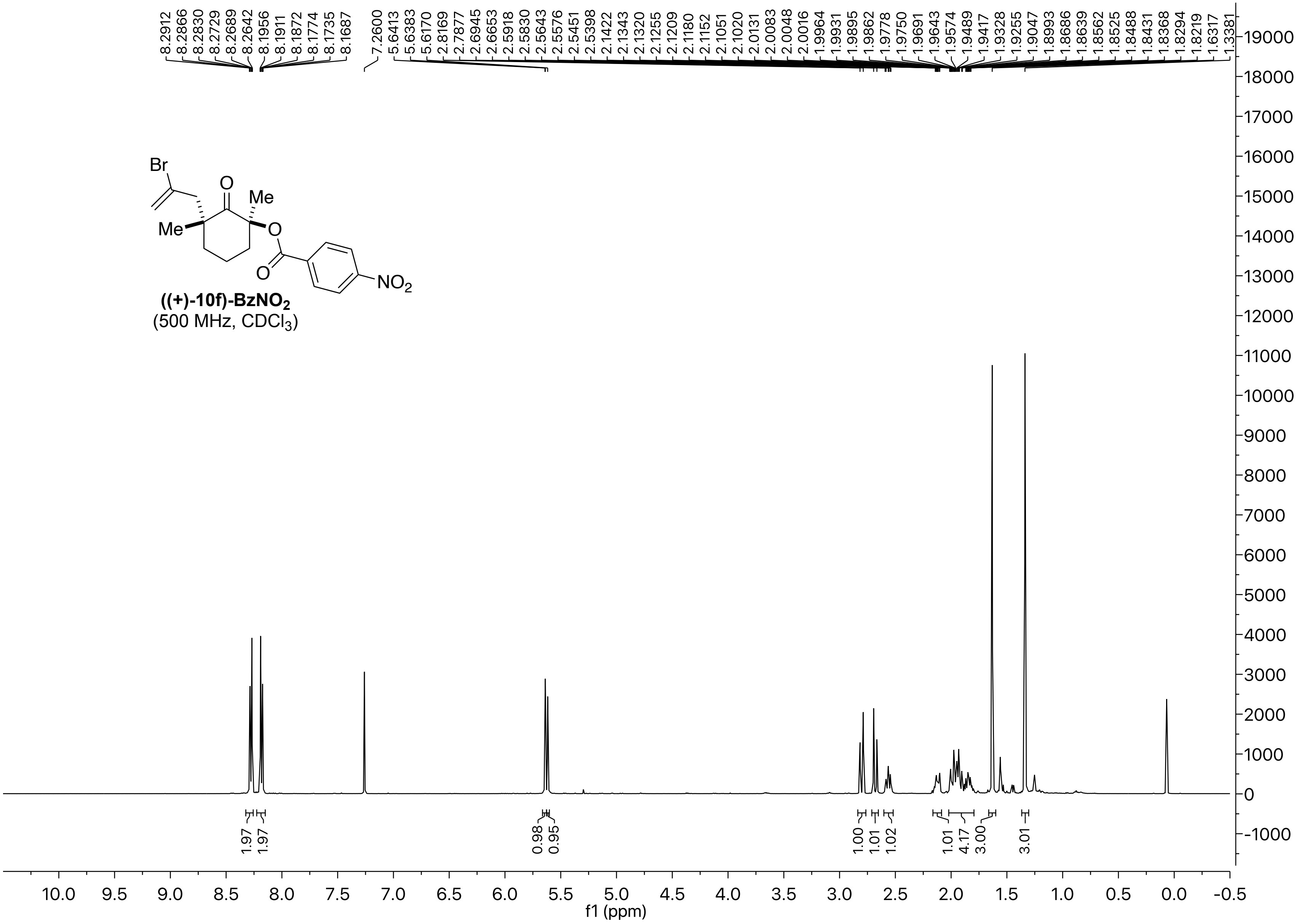
8.2912
8.2866
8.2830
8.2729
8.2689
8.2642
8.1956
8.1911
8.1872
8.1774
8.1735
8.1687
7.2600
5.6413
5.6383
5.6170
2.8169
2.7877
2.6945
2.6653
2.5918
2.5830
2.5643
2.5576
2.5451
2.5398
2.1422
2.1343
2.1320
2.1255
2.1209
2.1180
2.1152
2.1051
2.1020
2.0131
2.0083
2.0048
2.0016
1.9964
1.9931
1.9895
1.9862
1.9778
1.9750
1.9691
1.9643
1.9574
1.9489
1.9417
1.9328
1.9255
1.9047
1.8993
1.8686
1.8639
1.8562
1.8525
1.8488
1.8431
1.8368
1.8294
1.8219
1.6317
1.3381
Br
O
Me
Me
O
O
NO2
((+)-10f)-BzNO2
(500 MHz, CDCl3)
1.97
1.97
0.98
0.95
1.00
1.01
1.02
1.01
4.17
3.00
3.01
f1 (ppm)

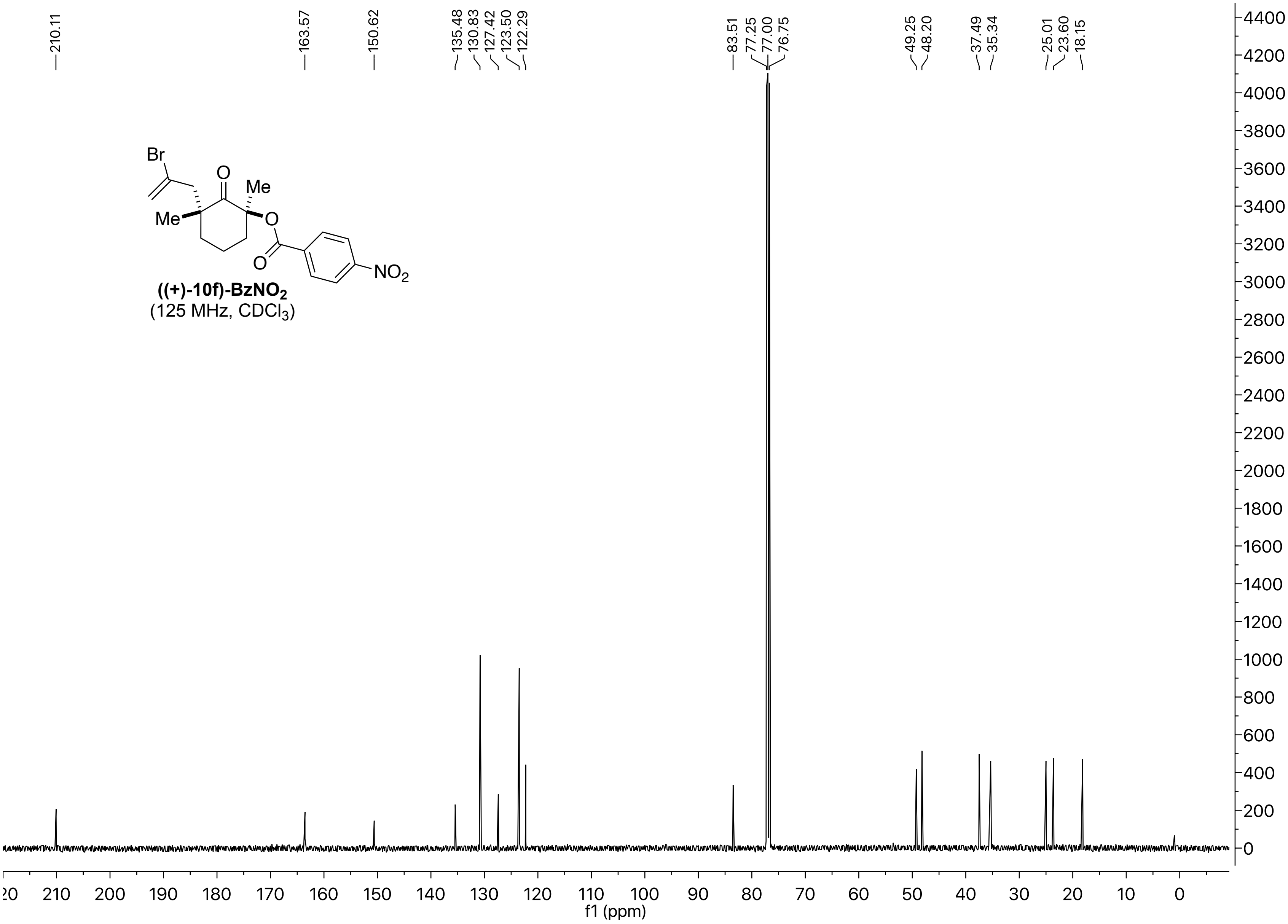

210.11
163.57
150.62
135.48
130.83
127.42
123.50
122.29
83.51
77.25
77.00
76.75
49.25
48.20
37.49
35.34
25.01
23.60
18.15
Br
O
Me
Me
O
O
NO2
((+)-10f)-BzNO2
(125 MHz, CDCl3)
f1 (ppm)

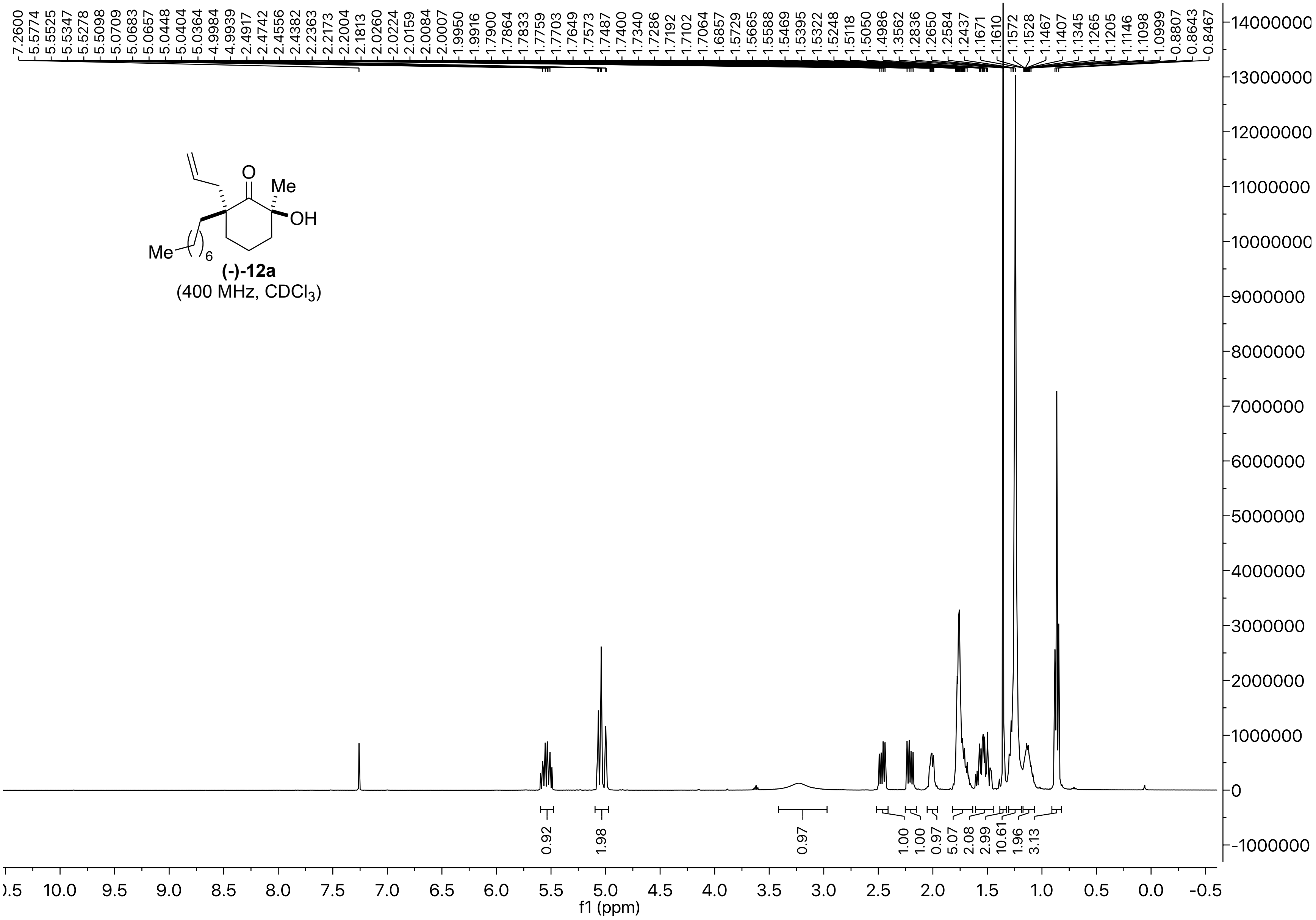

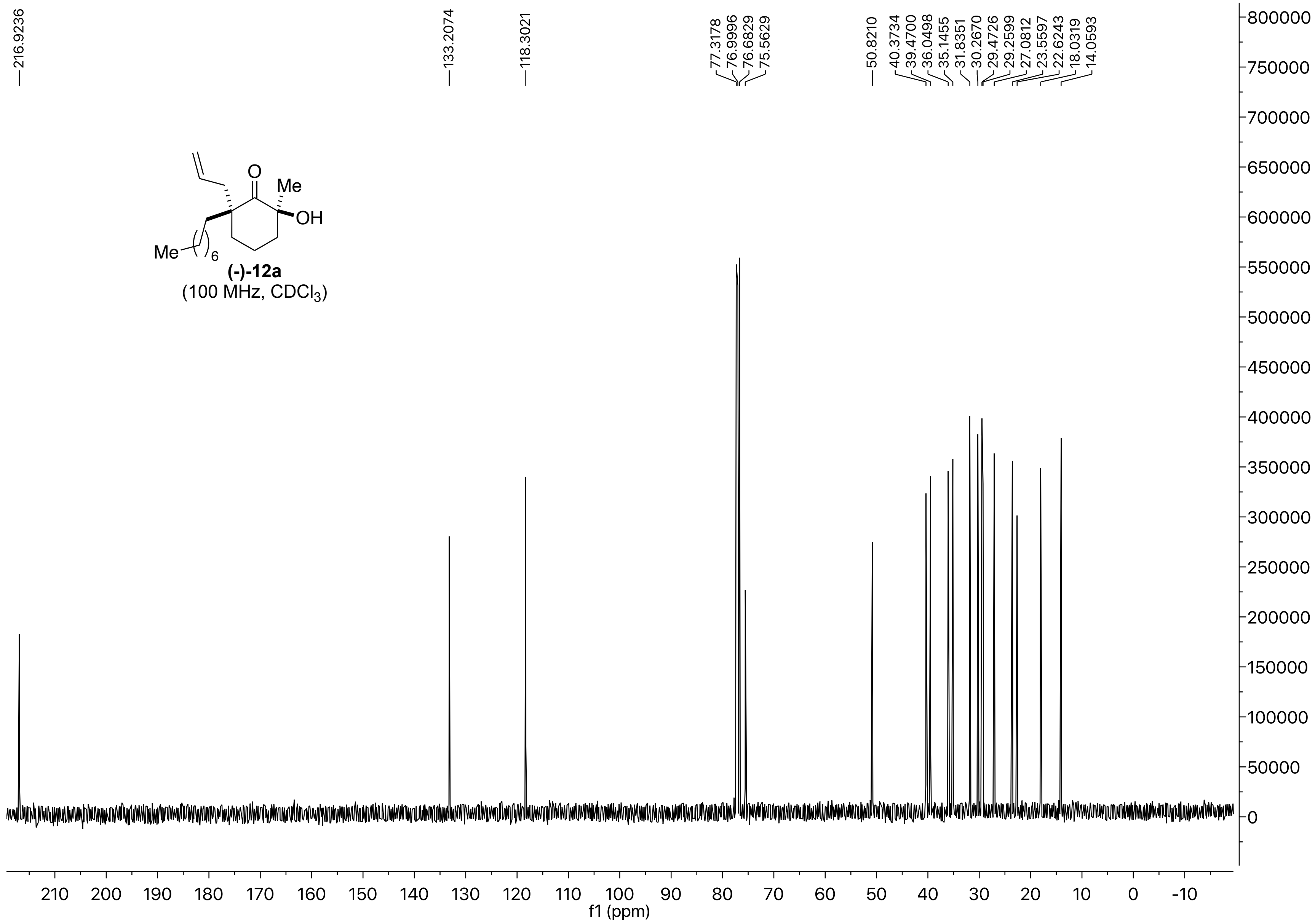

216.9236
133.2074
118.3021
77.3178
76.9996
76.6829
75.5629
50.8210
40.3734
39.4700
36.0498
35.1455
31.8351
30.2670
29.4726
29.2599
27.0812
23.5597
22.6243
18.0319
14.0593
O
Me
OH
Me
6
(-)-12a
(100 MHz, $CDCl_3$)
f1 (ppm)

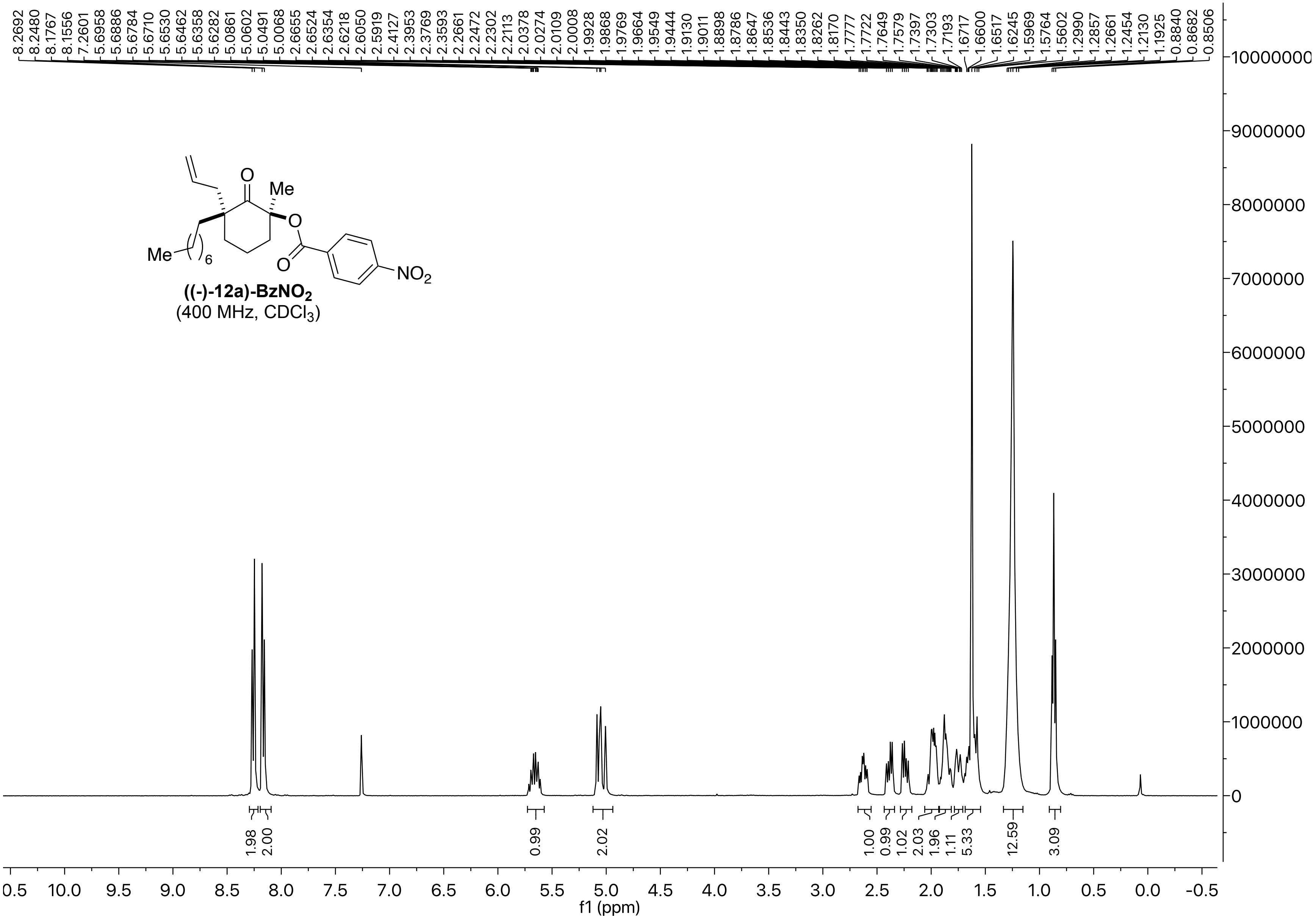
((-)-12a)-BzNO2
(400 MHz, CDCl3)
f1 (ppm)

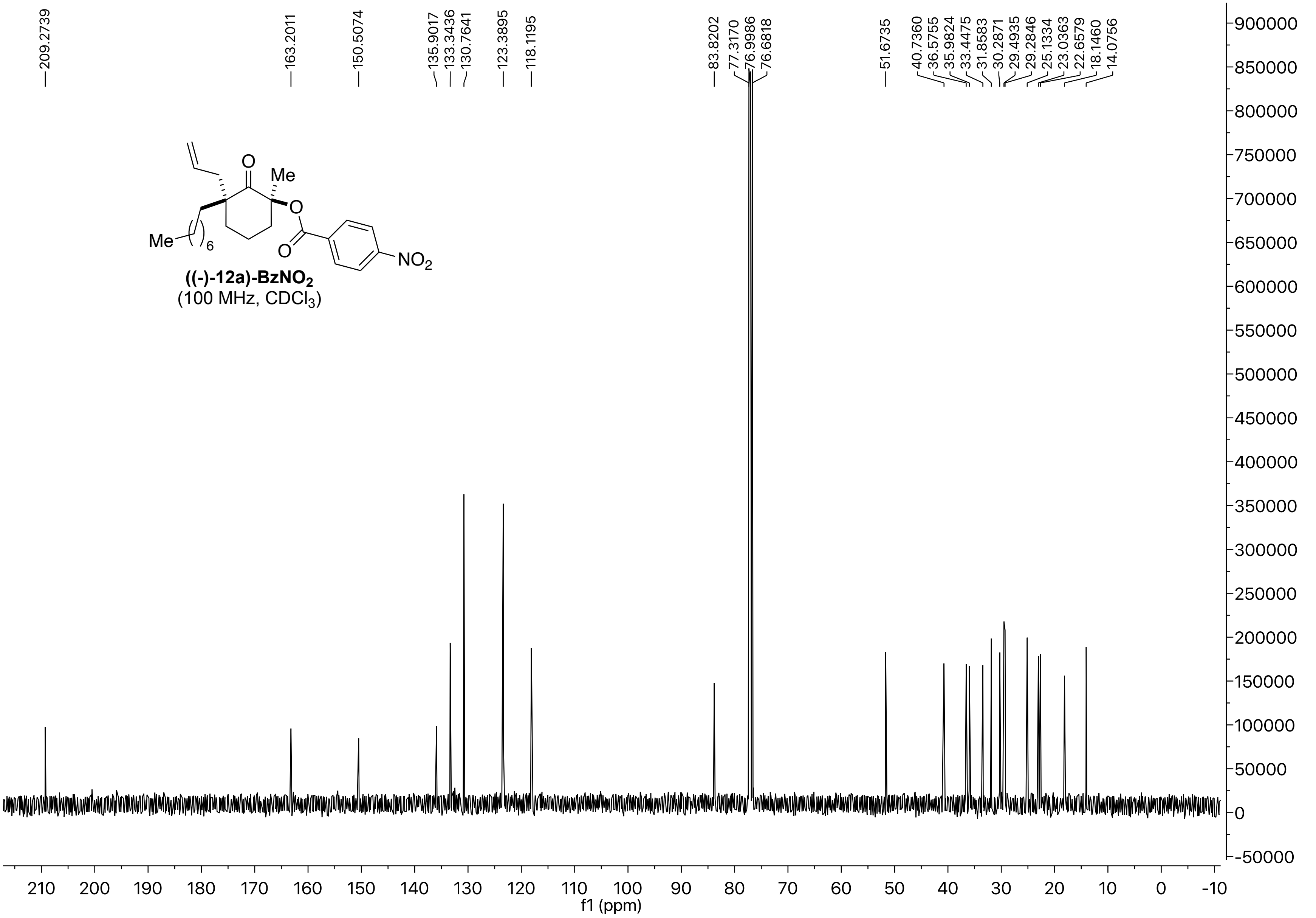

((-)-12a)-BzNO$_2$
(100 MHz, $CDCl_3$)

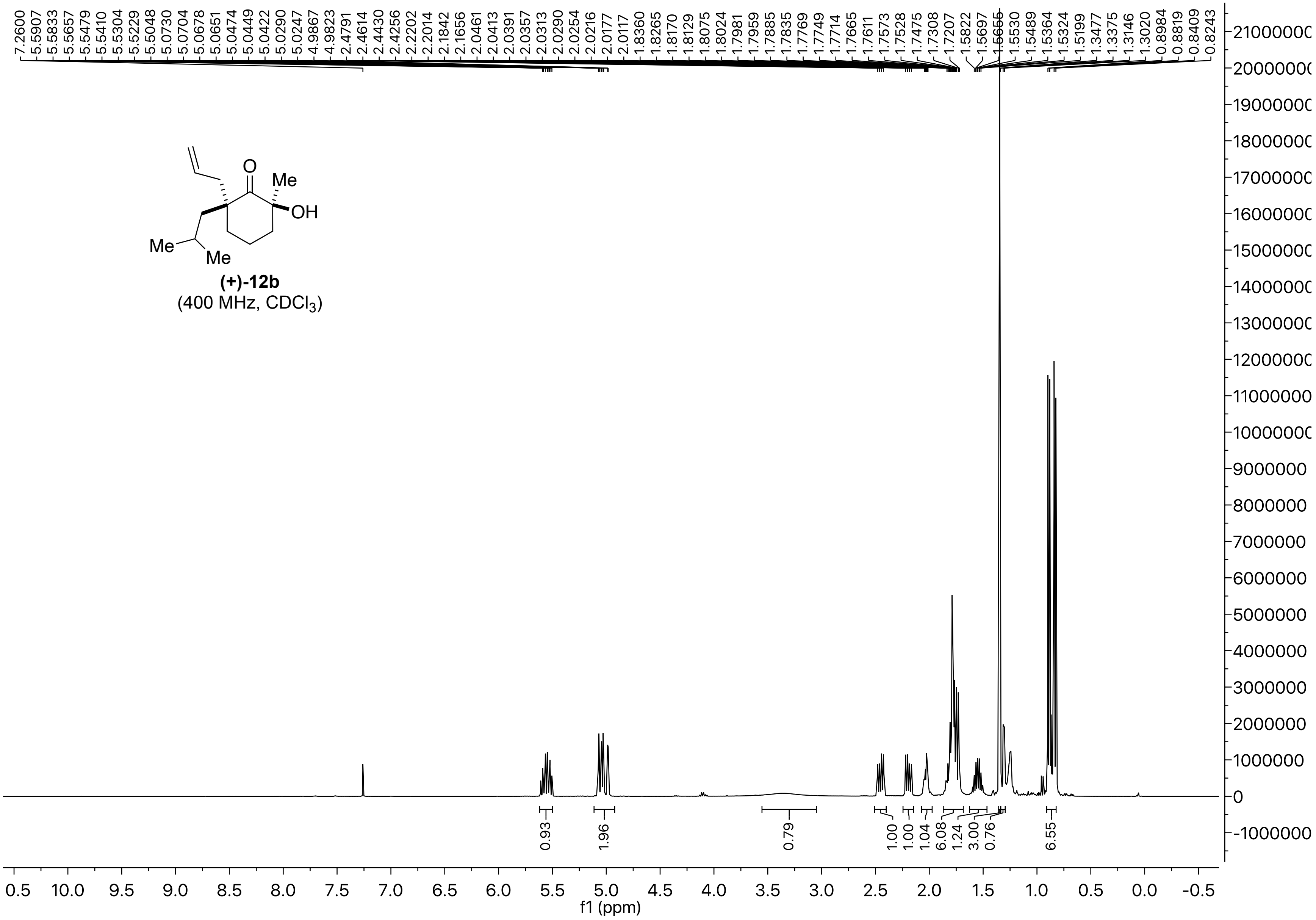

(+)-12b
(400 MHz, $CDCl_3$)

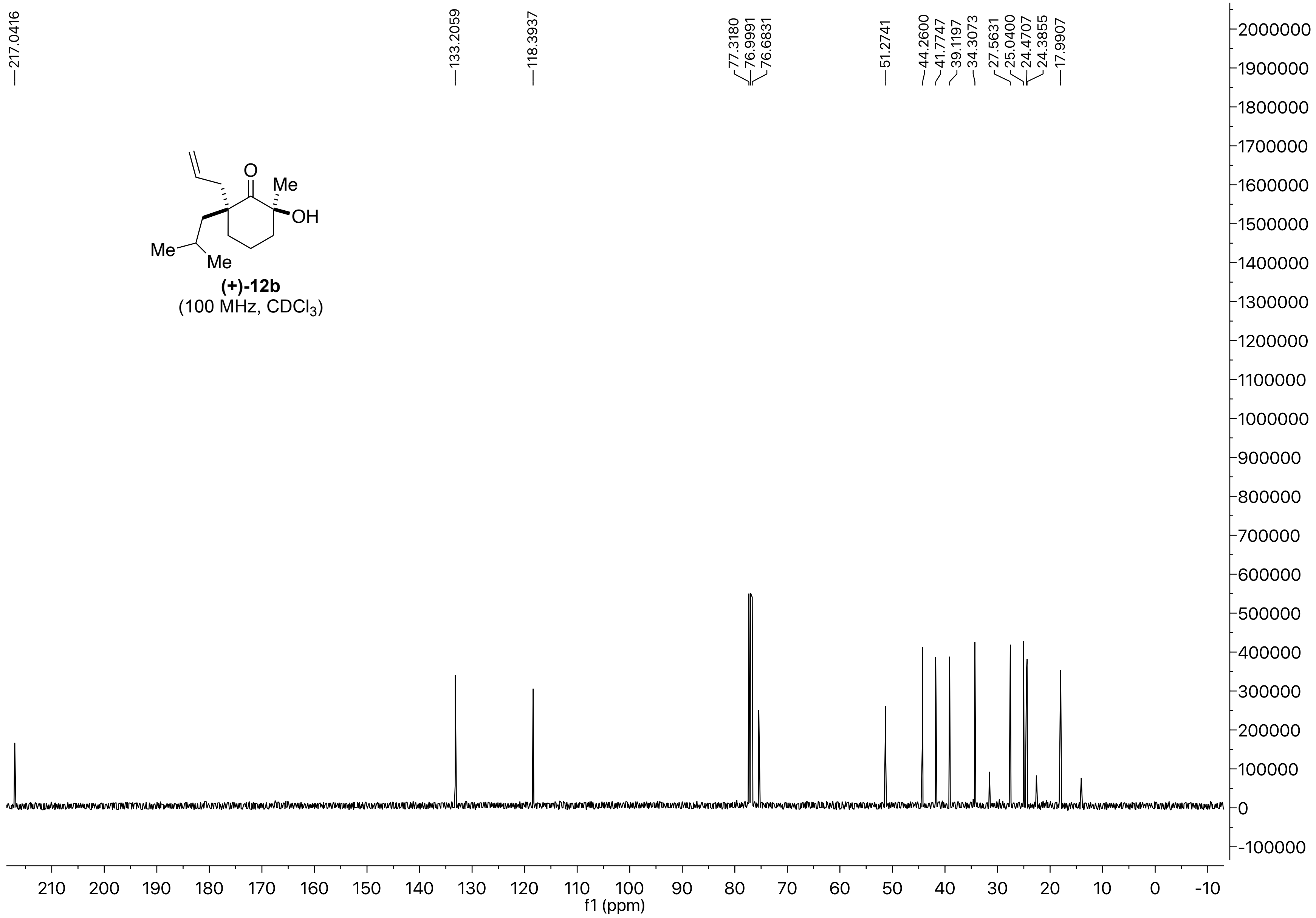

217.0416
133.2059
118.3937
77.3180
76.9991
76.6831
51.2741
44.2600
41.7747
39.1197
34.3073
27.5631
25.0400
24.4707
24.3855
17.9907
O
Me
OH
Me
Me
(+)-12b
(100 MHz, CDCl3)
f1 (ppm)

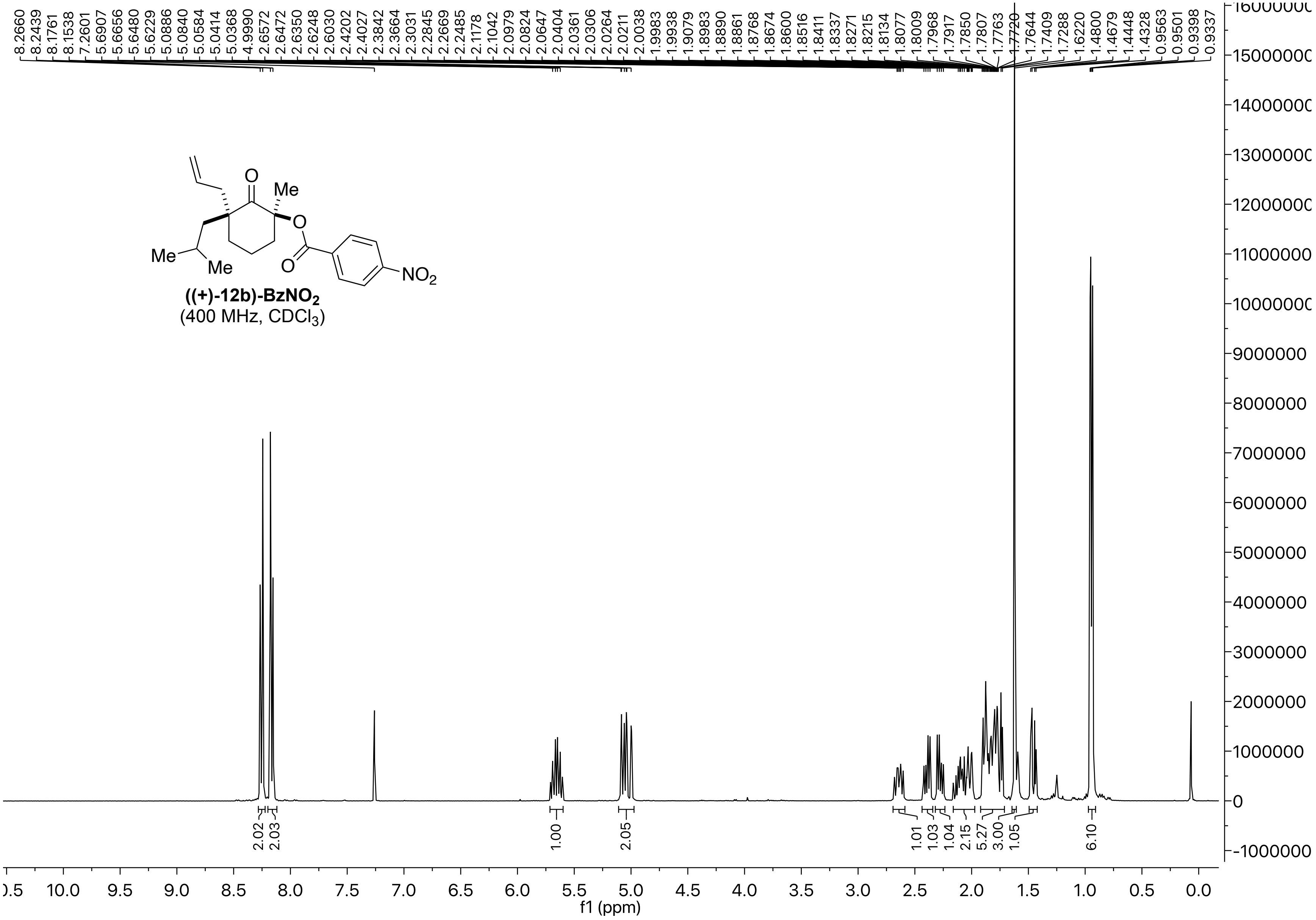
Me
O
Me
O
O
O
NO2
Me
((+)-12b)-BzNO2
(400 MHz, CDCl3)
f1 (ppm)

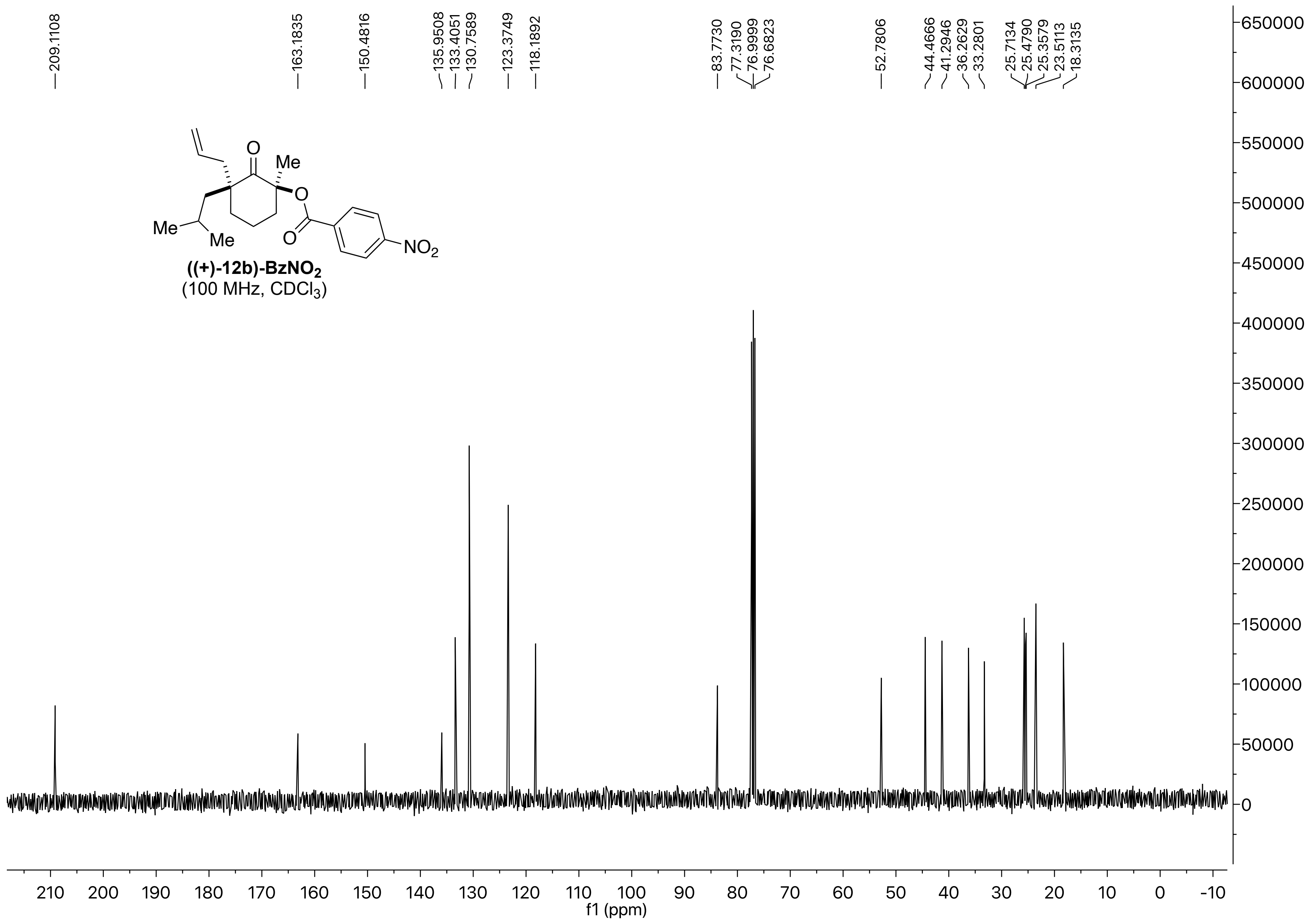
209.1108
163.1835
150.4816
135.9508
133.4051
130.7589
123.3749
118.1892
83.7730
77.3190
76.9999
76.6823
52.7806
44.4666
41.2946
36.2629
33.2801
25.7134
25.4790
25.3579
23.5113
18.3135
Me
O
Me
O
Me
O
NO_2
((+)-12b)-$BzNO_2$
(100 MHz, $CDCl_3$)
f1 (ppm)

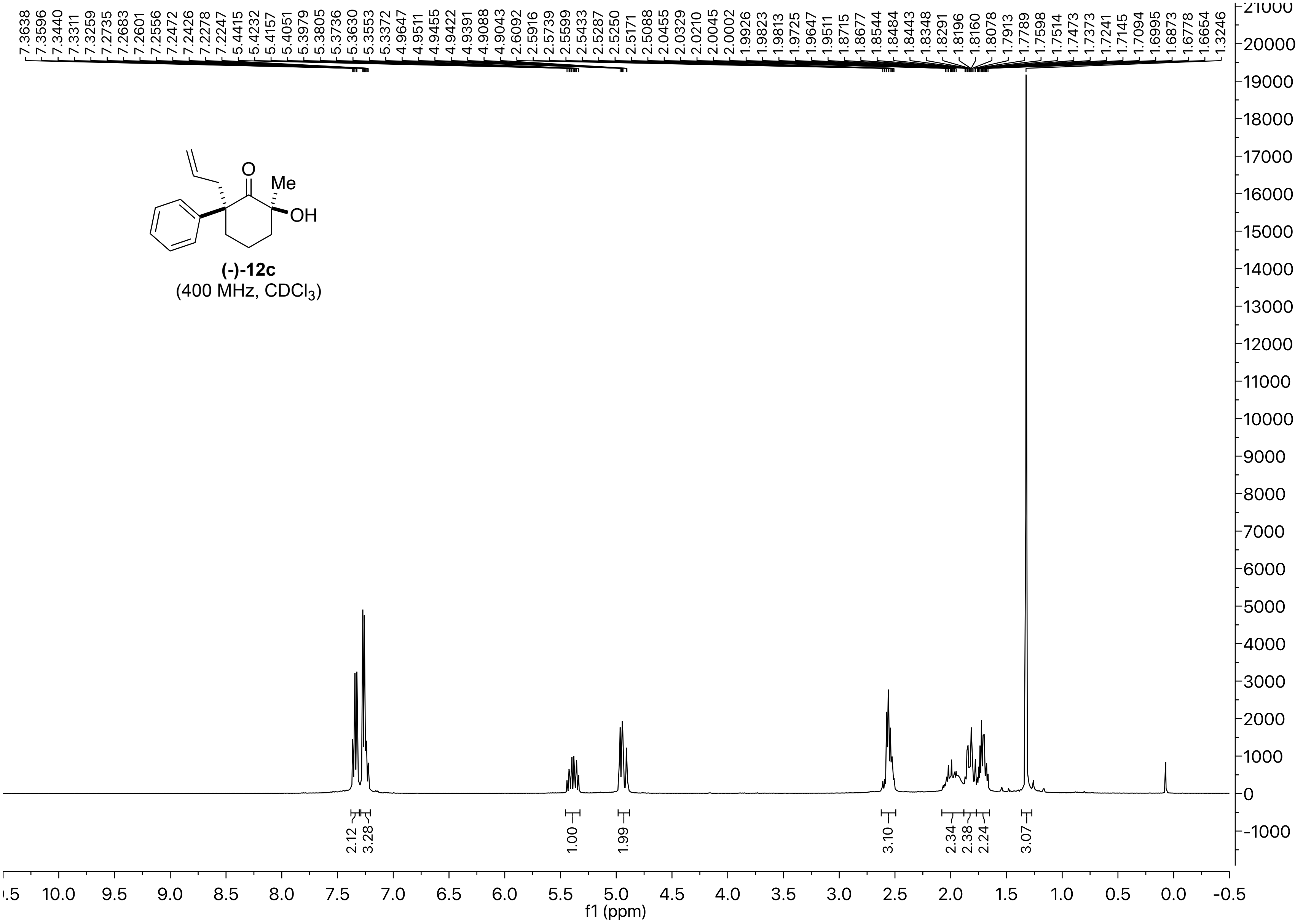
O
Me
OH
(-)-12c
(400 MHz, CDCl3)
f1 (ppm)

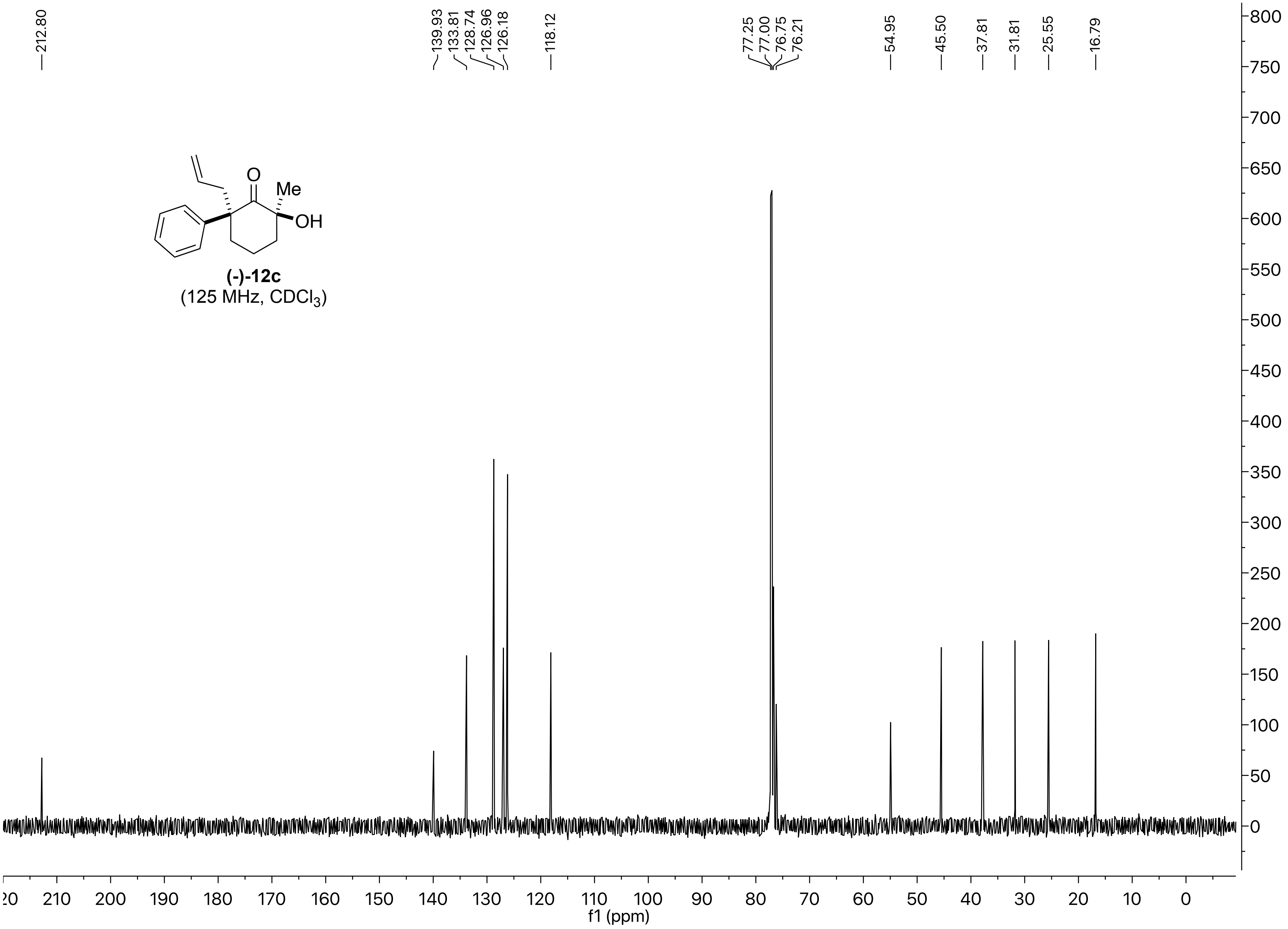

212.80
139.93
133.81
128.74
126.96
126.18
118.12
77.25
77.00
76.75
76.21
54.95
45.50
37.81
31.81
25.55
16.79
O
Me
OH
(-)-12c
(125 MHz, CDCl3)
f1 (ppm)

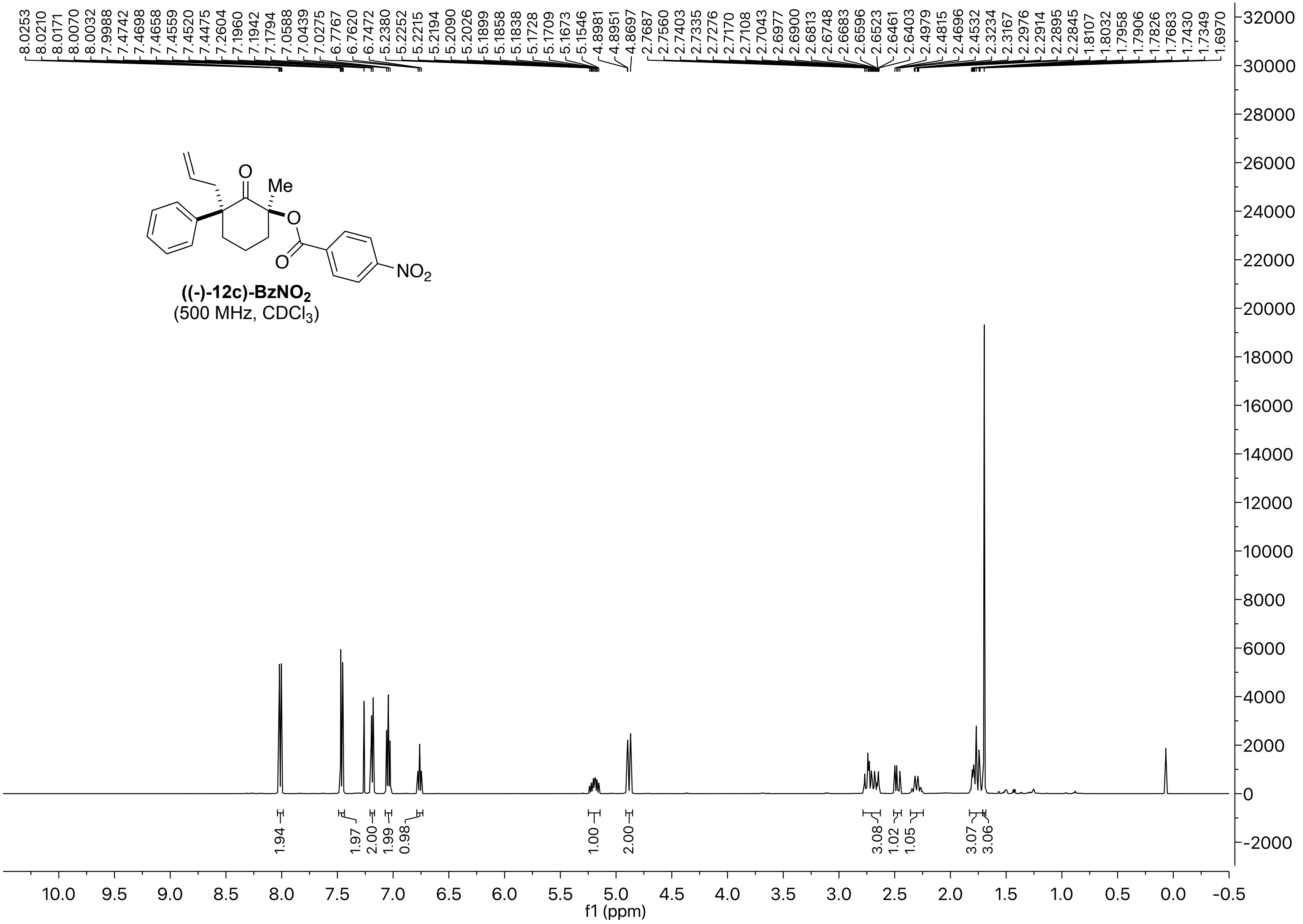
8.0253
8.0210
8.0171
8.0070
8.0032
7.9988
7.4742
7.4698
7.4658
7.4559
7.4520
7.4475
7.2604
7.1960
7.1942
7.1794
7.0588
7.0439
7.0275
6.7767
6.7620
6.7472
5.2380
5.2252
5.2215
5.2194
5.2090
5.2026
5.1899
5.1858
5.1838
5.1728
5.1709
5.1673
5.1546
4.8981
4.8951
4.8697
2.7687
2.7560
2.7403
2.7335
2.7276
2.7170
2.7108
2.7043
2.6977
2.6900
2.6813
2.6748
2.6683
2.6596
2.6523
2.6461
2.6403
2.4979
2.4815
2.4696
2.4532
2.3234
2.3167
2.2976
2.2914
2.2895
2.2845
1.8107
1.8032
1.7958
1.7906
1.7826
1.7683
1.7430
1.7349
1.6970
O
Me
O
O
NO2
((-)-12c)-BzNO2
(500 MHz, CDCl3)
1.94
1.97
2.00
1.99
0.98
1.00
2.00
3.08
1.02
1.05
3.07
3.06
f1 (ppm)

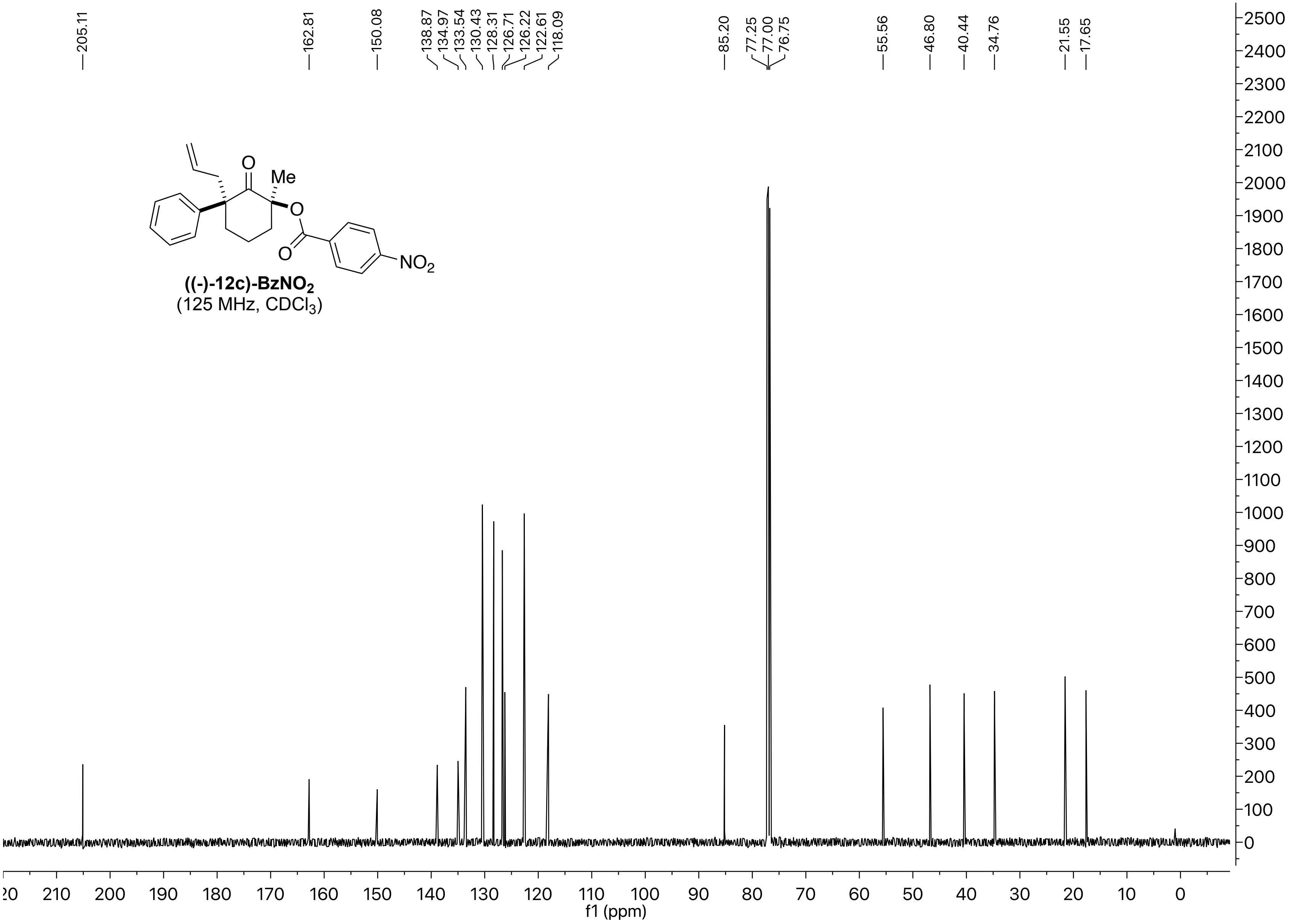

205.11
162.81
150.08
138.87
134.97
133.54
130.43
128.31
126.71
126.22
122.61
118.09
85.20
77.25
77.00
76.75
55.56
46.80
40.44
34.76
21.55
17.65
O
Me
O
O
NO2
((-)-12c)-BzNO2
(125 MHz, CDCl3)
f1 (ppm)

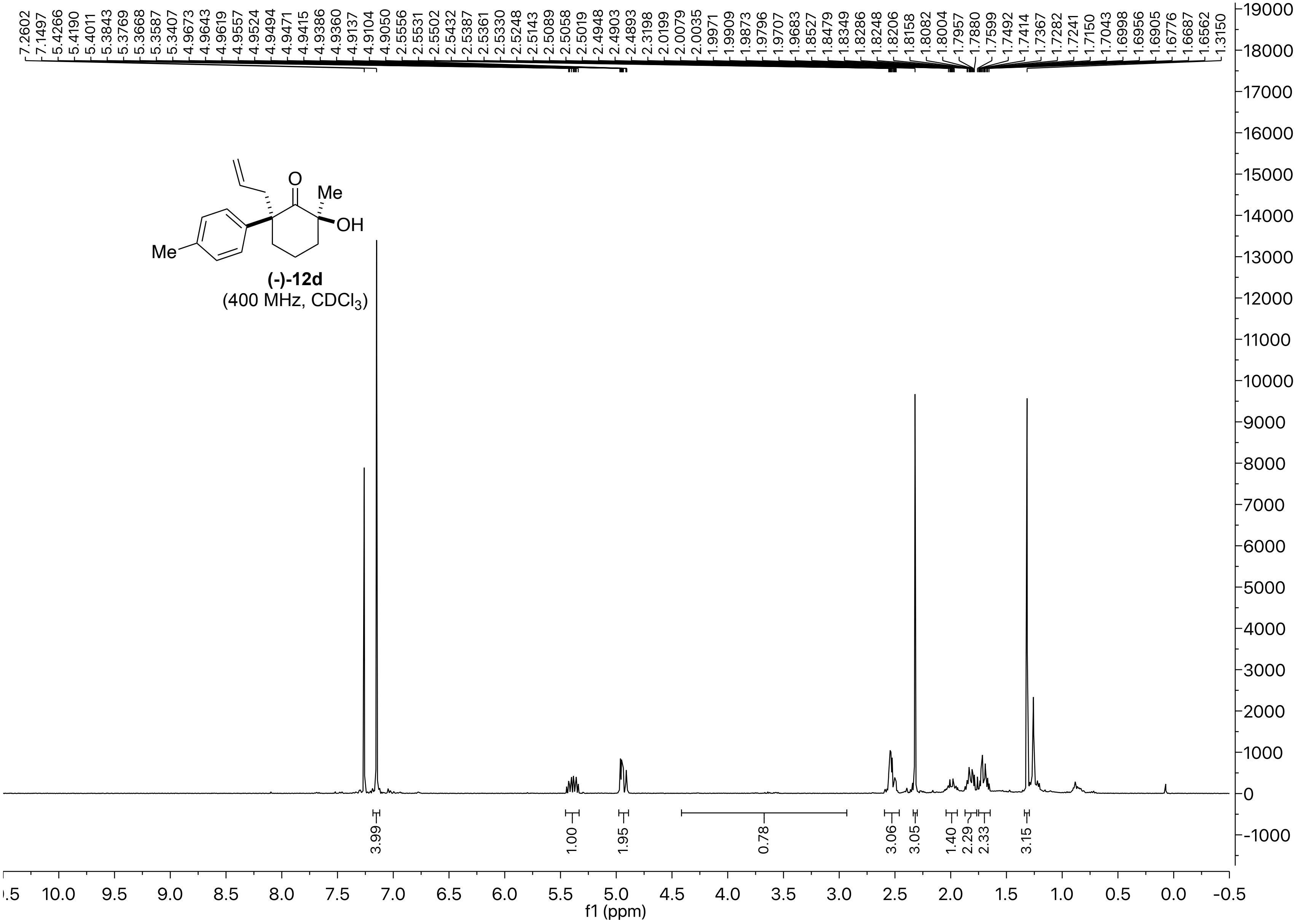

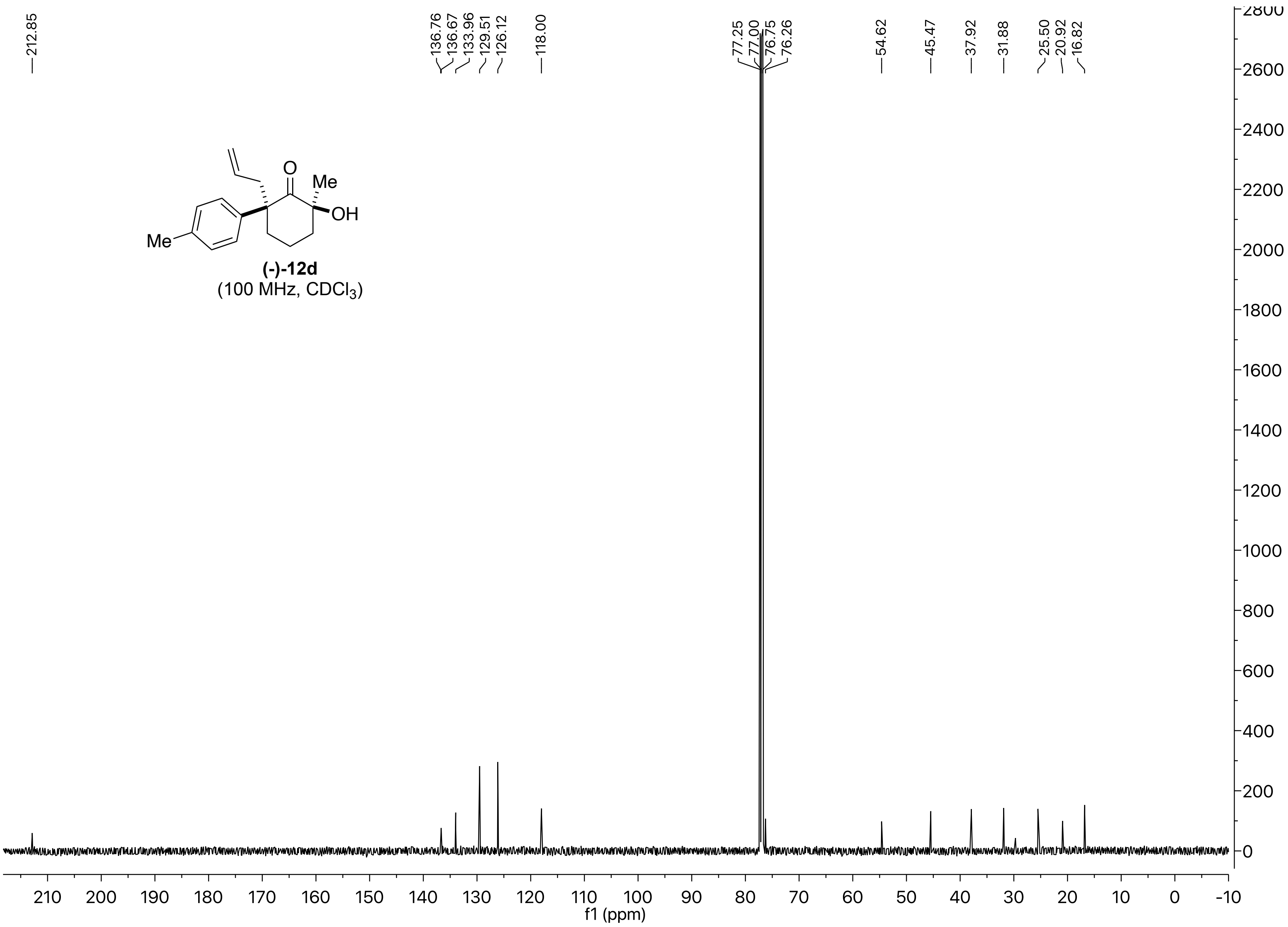
212.85
136.76
136.67
133.96
129.51
126.12
118.00
77.25
77.00
76.75
76.26
54.62
45.47
37.92
31.88
25.50
20.92
16.82
O
Me
OH
Me
(-)-12d
(100 MHz, $CDCl_3$)
f1 (ppm)

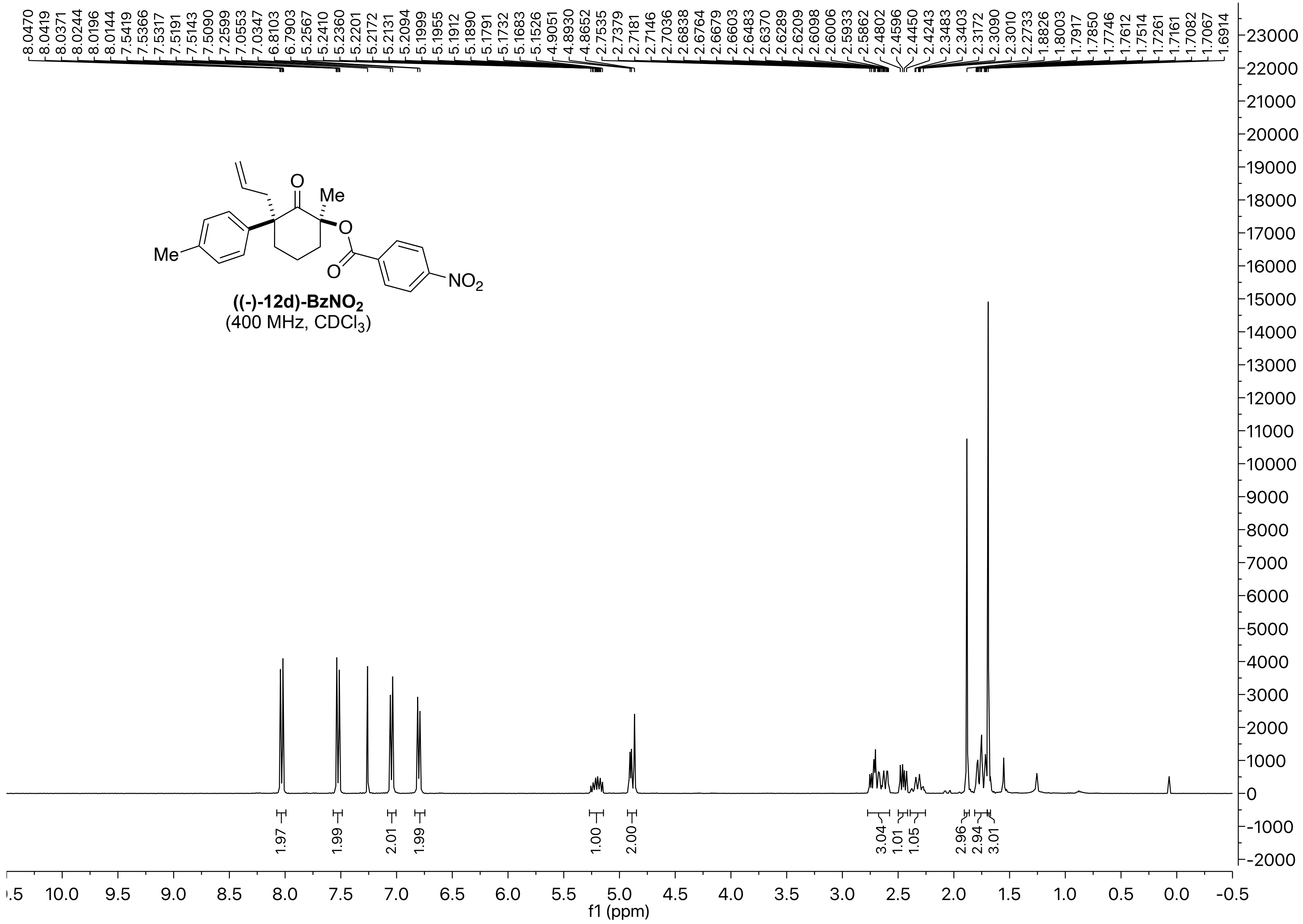
8.0470 8.0419 8.0371 8.0244 8.0196 8.0144 7.5419 7.5366 7.5317 7.5191 7.5143 7.5090 7.2599 7.0553 7.0347 6.8103 6.7903 5.2567 5.2410 5.2360 5.2201 5.2172 5.2131 5.2094 5.1999 5.1955 5.1912 5.1890 5.1791 5.1732 5.1683 5.1526 4.9051 4.8930 4.8652 2.7535 2.7379 2.7181 2.7146 2.7036 2.6838 2.6764 2.6679 2.6603 2.6483 2.6370 2.6289 2.6209 2.6098 2.6006 2.5933 2.5862 2.4802 2.4596 2.4450 2.4243 2.3483 2.3403 2.3172 2.3090 2.3010 2.2733 1.8826 1.8003 1.7917 1.7850 1.7746 1.7612 1.7514 1.7261 1.7161 1.7082 1.7067 1.6914
O
Me
O
Me
O
O
NO2
((-)-12d)-BzNO2
(400 MHz, CDCl3)
1.97
1.99
2.01
1.99
1.00
2.00
3.04
1.01
1.05
2.96
2.94
3.01
f1 (ppm)

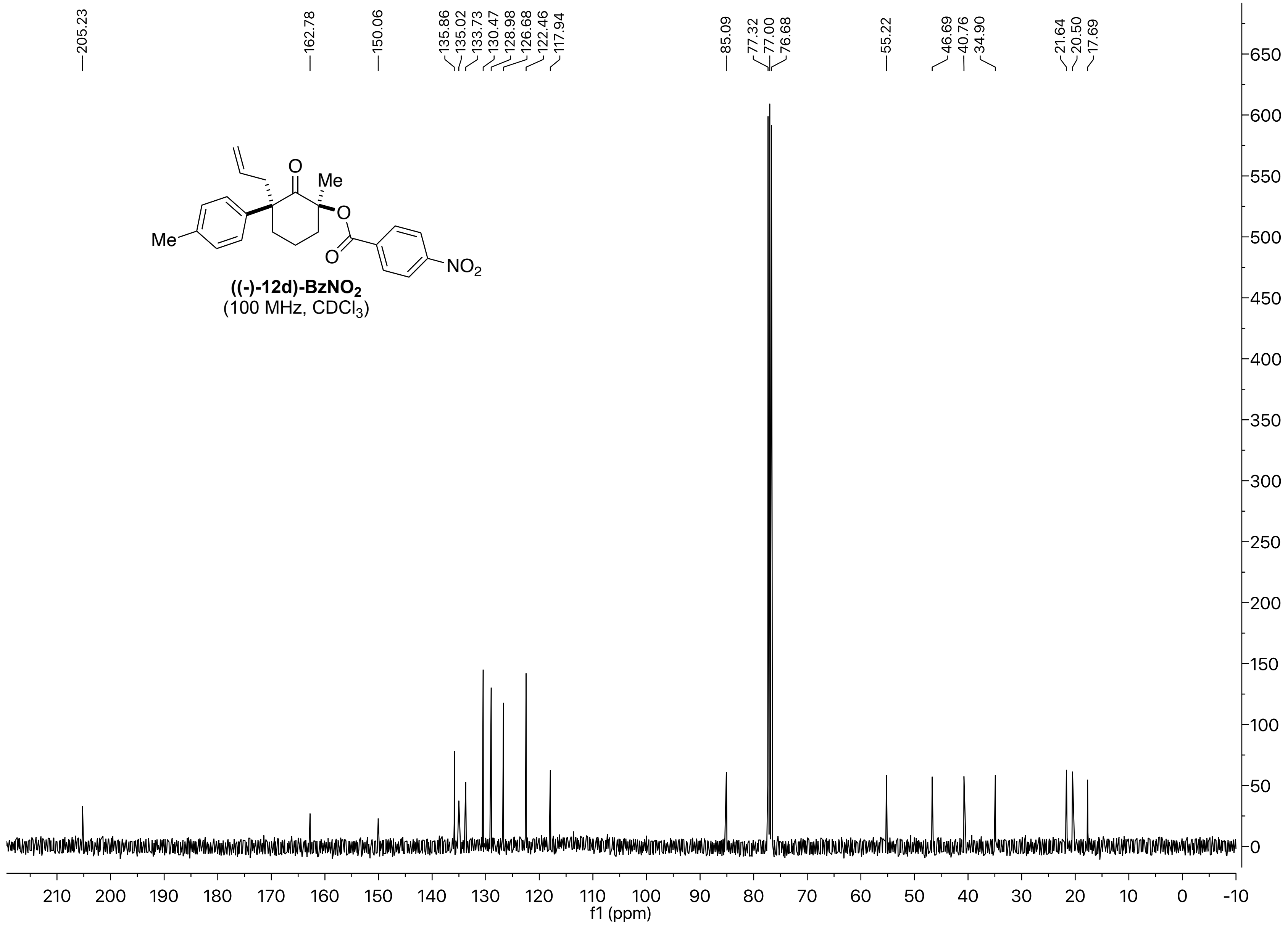

205.23
162.78
150.06
135.86
135.02
133.73
130.47
128.98
126.68
122.46
117.94
85.09
77.32
77.00
76.68
55.22
46.69
40.76
34.90
21.64
20.50
17.69
O
Me
O
Me
O
NO2
((-)-12d)-BzNO2
(100 MHz, CDCl3)
f1 (ppm)

(-)-12e
(400 MHz, $CDCl_3$)

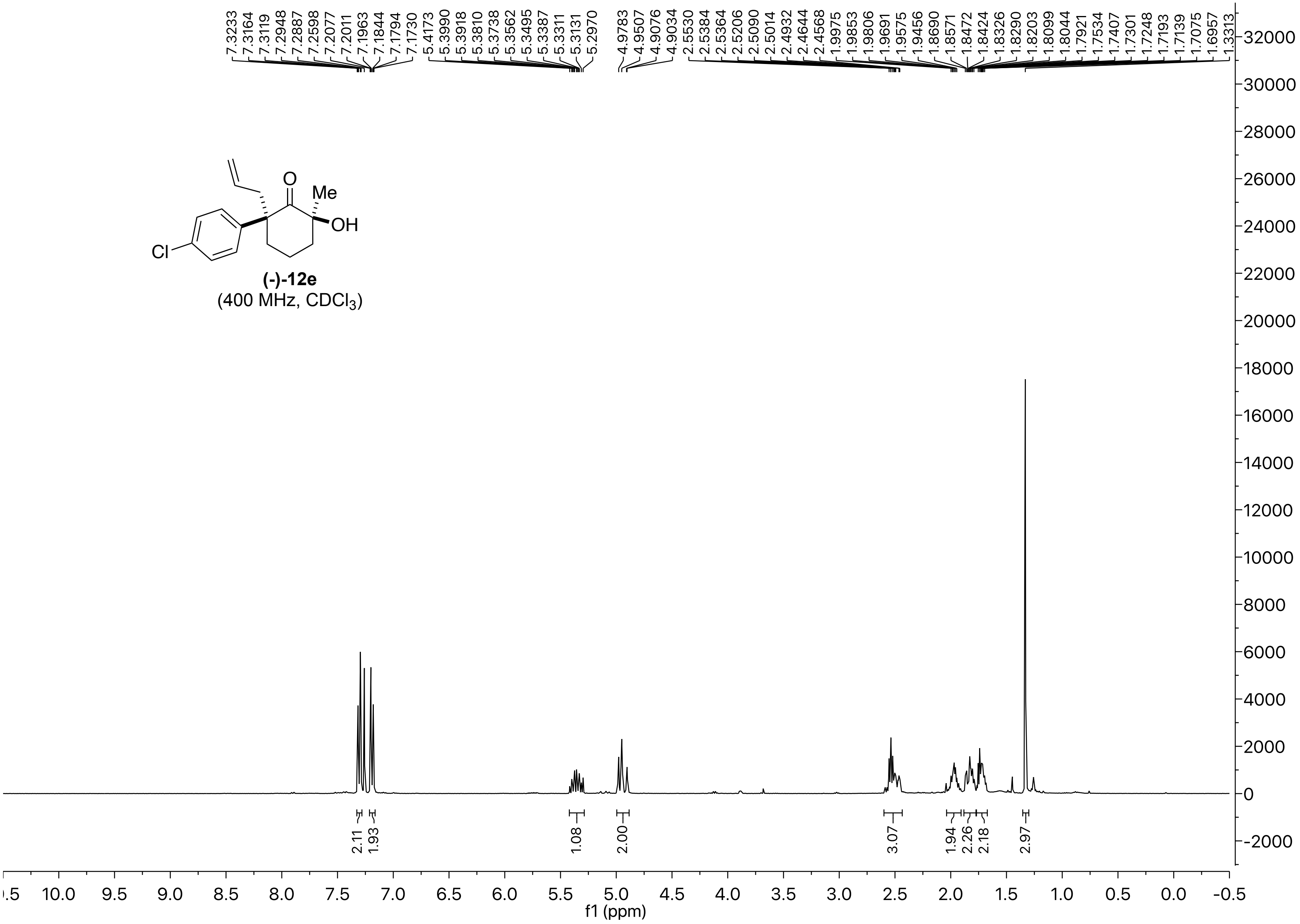

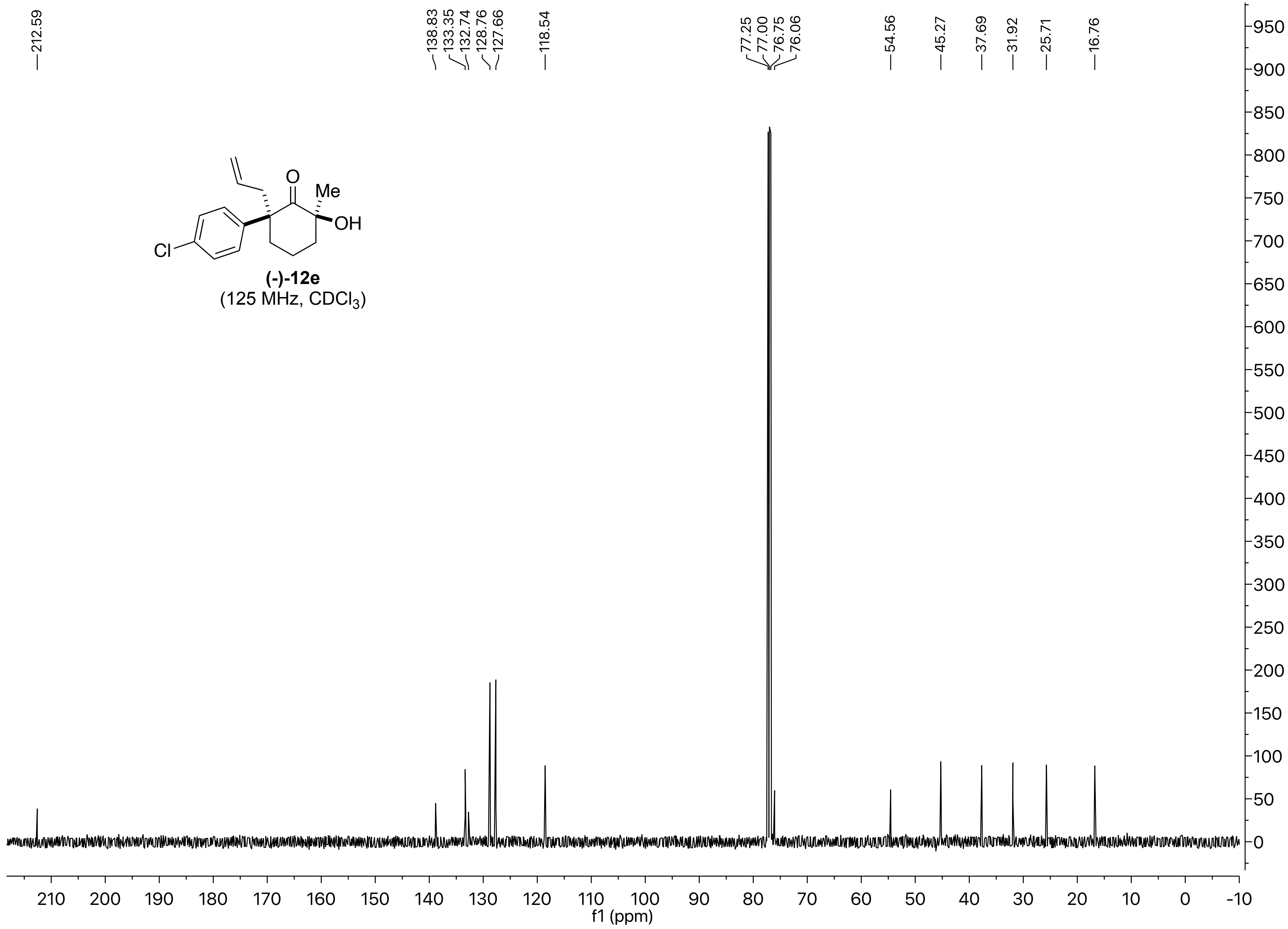
212.59
138.83
133.35
132.74
128.76
127.66
118.54
77.25
77.00
76.75
76.06
54.56
45.27
37.69
31.92
25.71
16.76
Me
OH
O
Cl
(-)-12e
(125 MHz, CDCl3)
f1 (ppm)

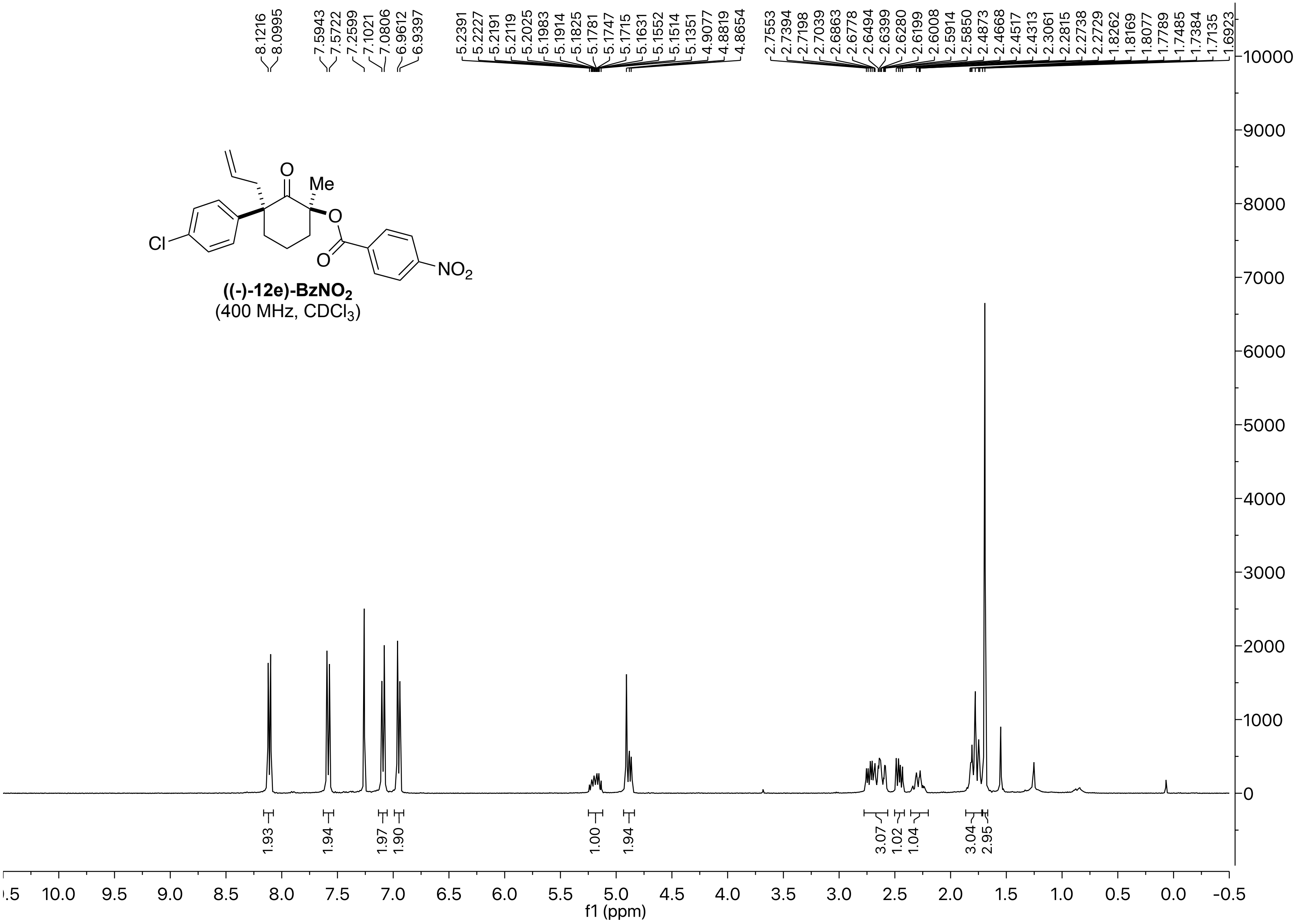

((-)-12e)-BzNO$_2$
(400 MHz, CDCl$_3$)
Me
O
Cl
NO$_2$
f1 (ppm)

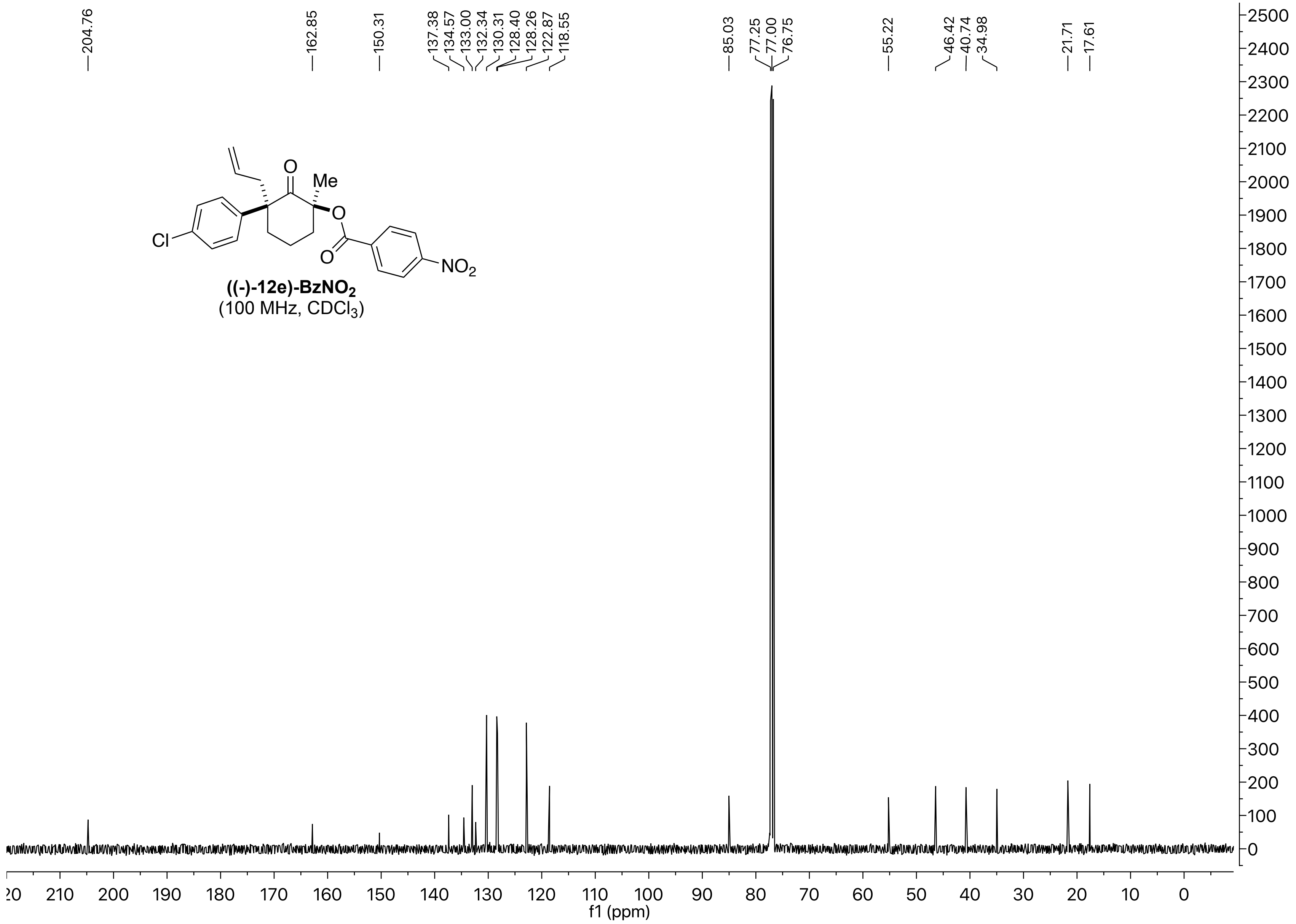

204.76
162.85
150.31
137.38
134.57
133.00
132.34
130.31
128.40
128.26
122.87
118.55
85.03
77.25
77.00
76.75
55.22
46.42
40.74
34.98
21.71
17.61
Me
O
Cl
NO2
((-)-12e)-BzNO2
(100 MHz, CDCl3)
f1 (ppm)

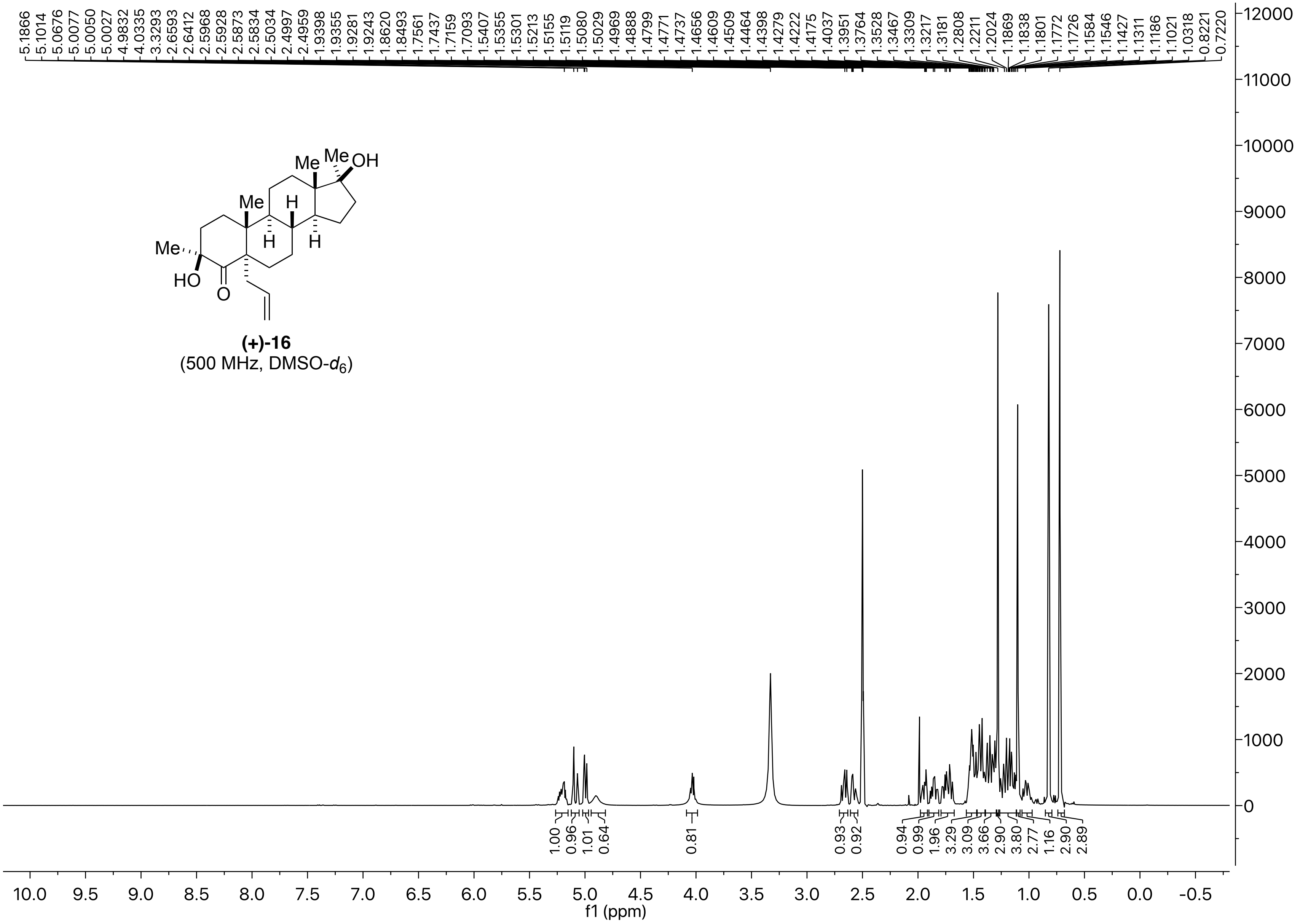
Me
Me
OH
Me
H
H
H
Me
HO
O
(+)-16
(500 MHz, DMSO-d_6)
f1 (ppm)

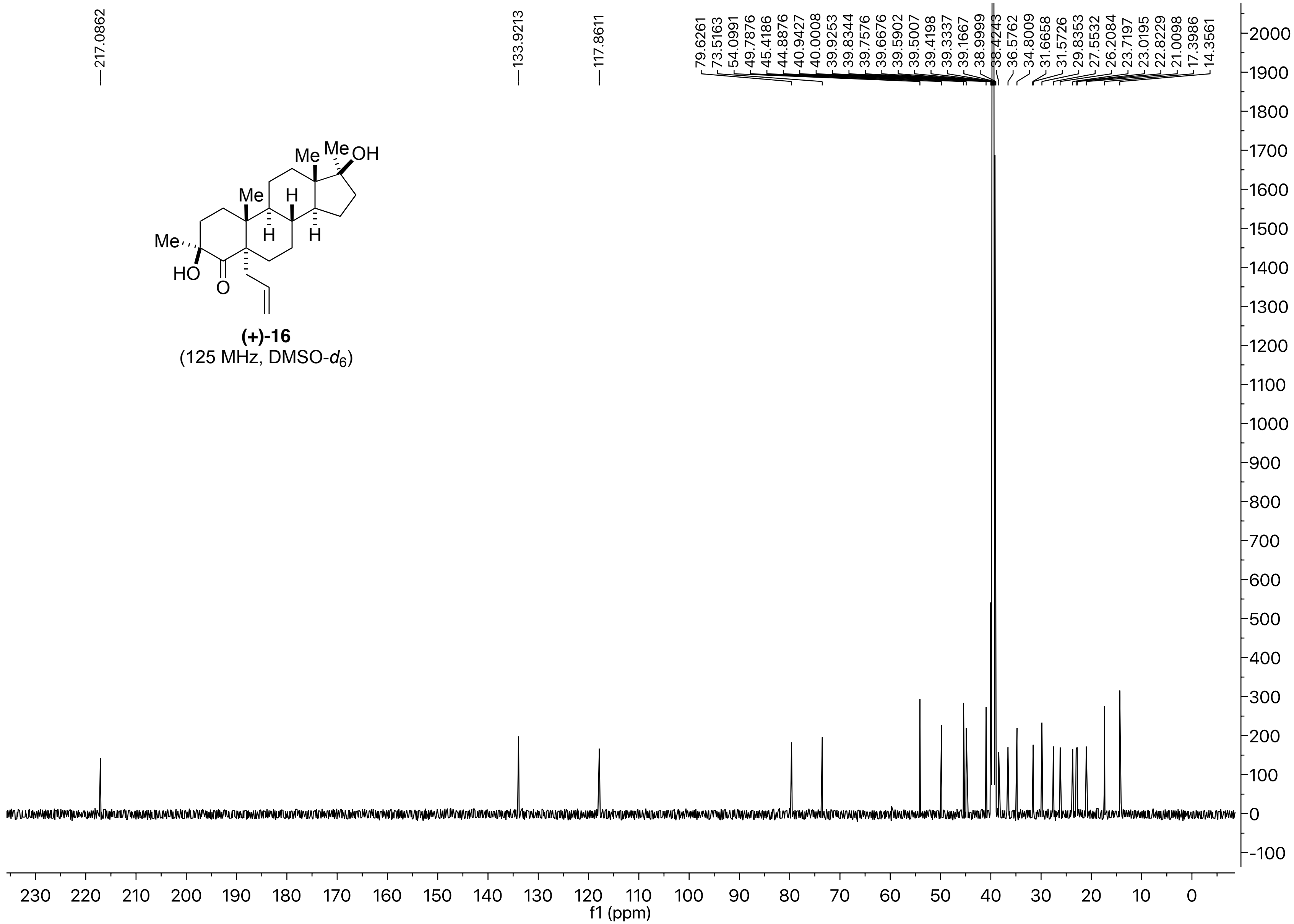

217.0862
133.9213
117.8611
79.6261
73.5163
54.0991
49.7876
45.4186
44.8876
40.9427
40.0008
39.9253
39.8344
39.7576
39.6676
39.5902
39.5007
39.4198
39.3337
39.1667
38.9999
38.4243
36.5762
34.8009
31.6658
31.5726
29.8353
27.5532
26.2084
23.7197
23.0195
22.8229
21.0098
17.3986
14.3561
(+)-16
(125 MHz, DMSO-d_6)
f1 (ppm)

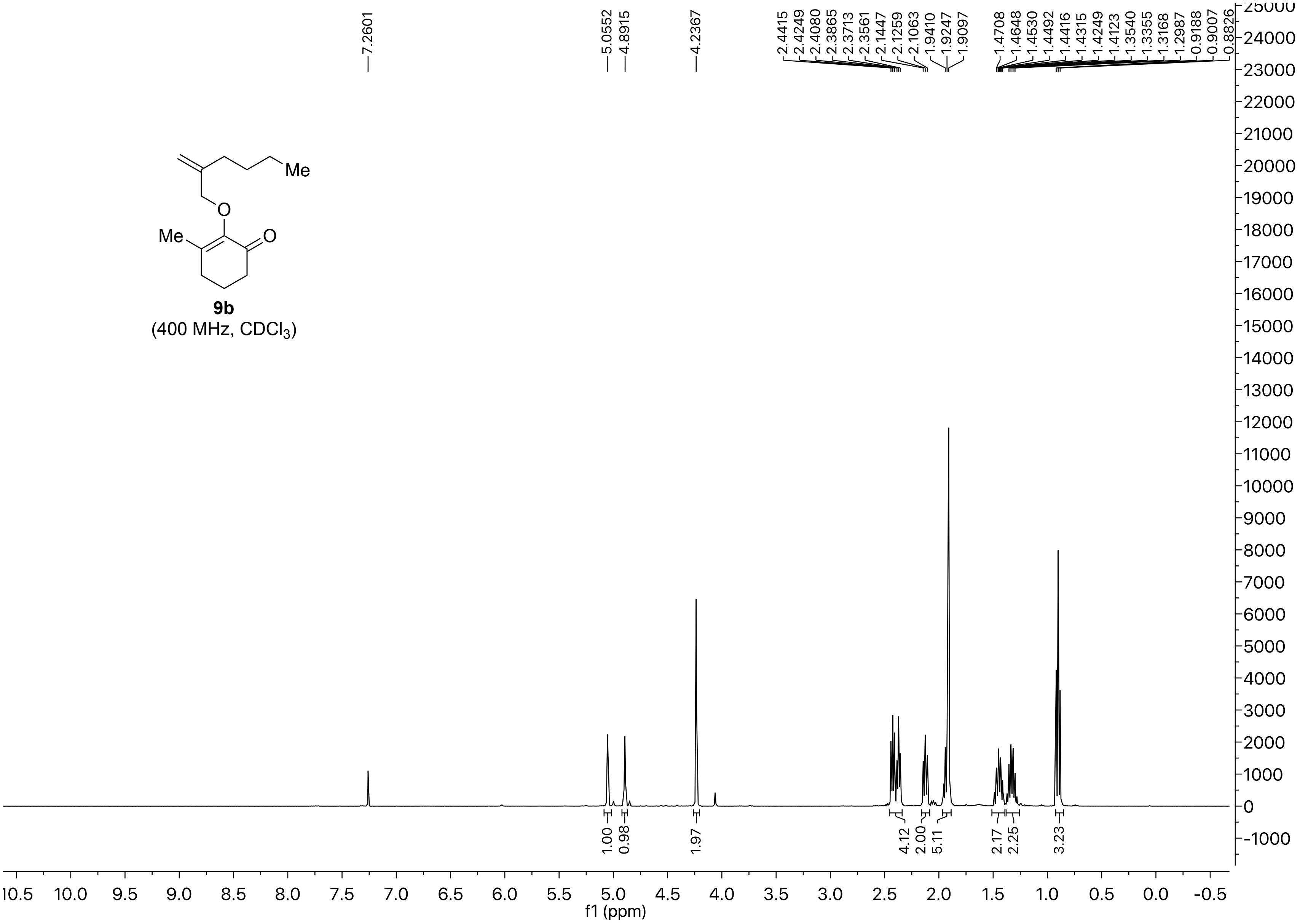

9b
(400 MHz, $CDCl_3$)
7.2601
5.0552
4.8915
4.2367
2.4415
2.4249
2.4080
2.3865
2.3713
2.3561
2.1447
2.1259
2.1063
1.9410
1.9247
1.9097
1.4708
1.4648
1.4530
1.4492
1.4416
1.4315
1.4249
1.4123
1.3540
1.3355
1.3168
1.2987
0.9188
0.9007
0.8826
1.00
0.98
1.97
4.12
2.00
5.11
2.17
2.25
3.23
f1 (ppm)

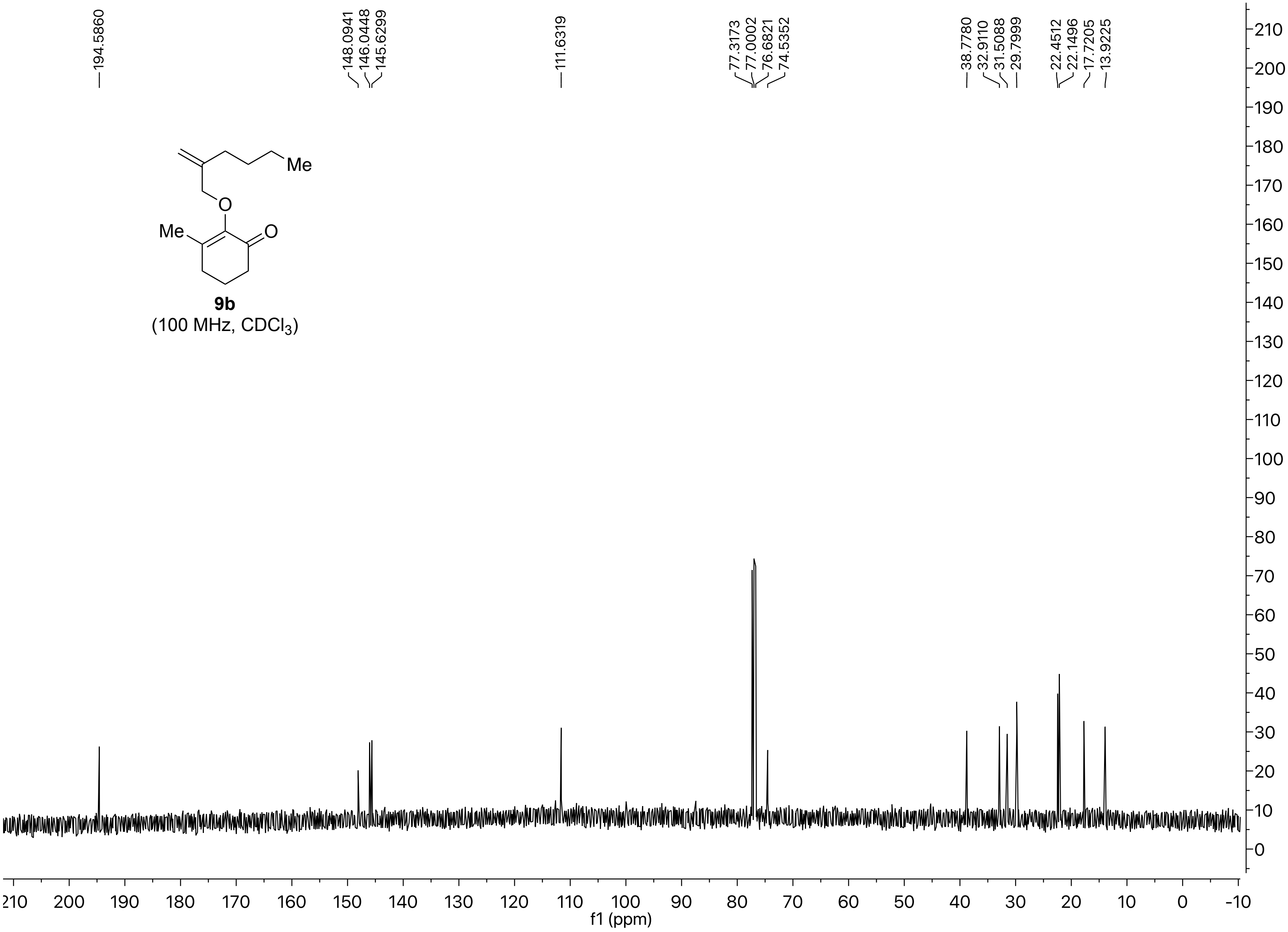
194.5860
148.0941
146.0448
145.6299
111.6319
77.3173
77.0002
76.6821
74.5352
38.7780
32.9110
31.5088
29.7999
22.4512
22.1496
17.7205
13.9225
Me
O
Me
O
9b
(100 MHz, CDCl3)
f1 (ppm)

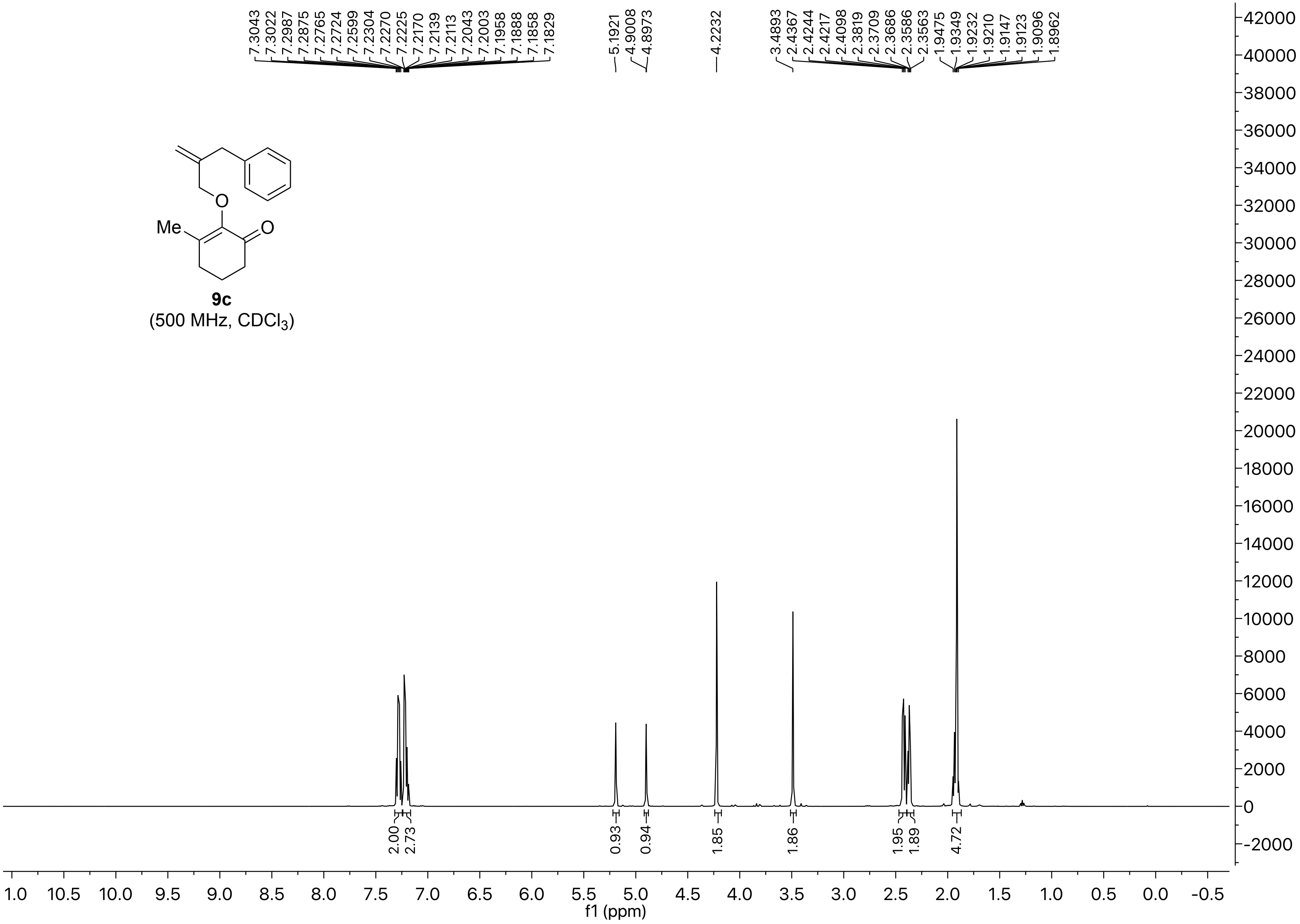
Me
O
O
9c
(500 MHz, CDCl3)
7.3043
7.3022
7.2987
7.2875
7.2765
7.2724
7.2599
7.2304
7.2270
7.2225
7.2170
7.2139
7.2113
7.2043
7.2003
7.1958
7.1888
7.1858
7.1829
5.1921
4.9008
4.8973
4.2232
3.4893
2.4367
2.4244
2.4217
2.4098
2.3819
2.3709
2.3686
2.3586
2.3563
1.9475
1.9349
1.9232
1.9210
1.9147
1.9123
1.9096
1.8962
2.00
2.73
0.93
0.94
1.85
1.86
1.95
1.89
4.72
f1 (ppm)

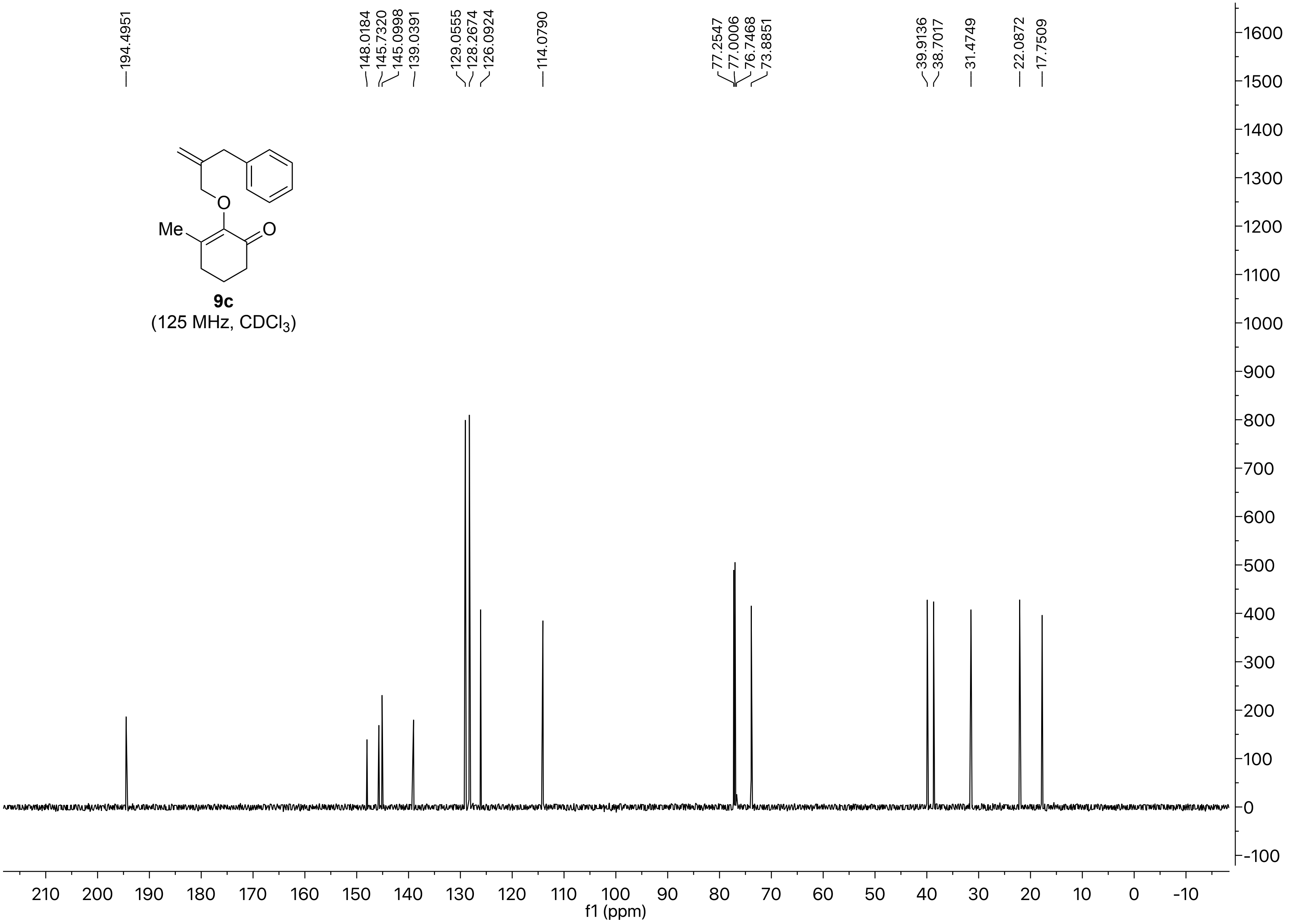

9c
(125 MHz, $CDCl_3$)

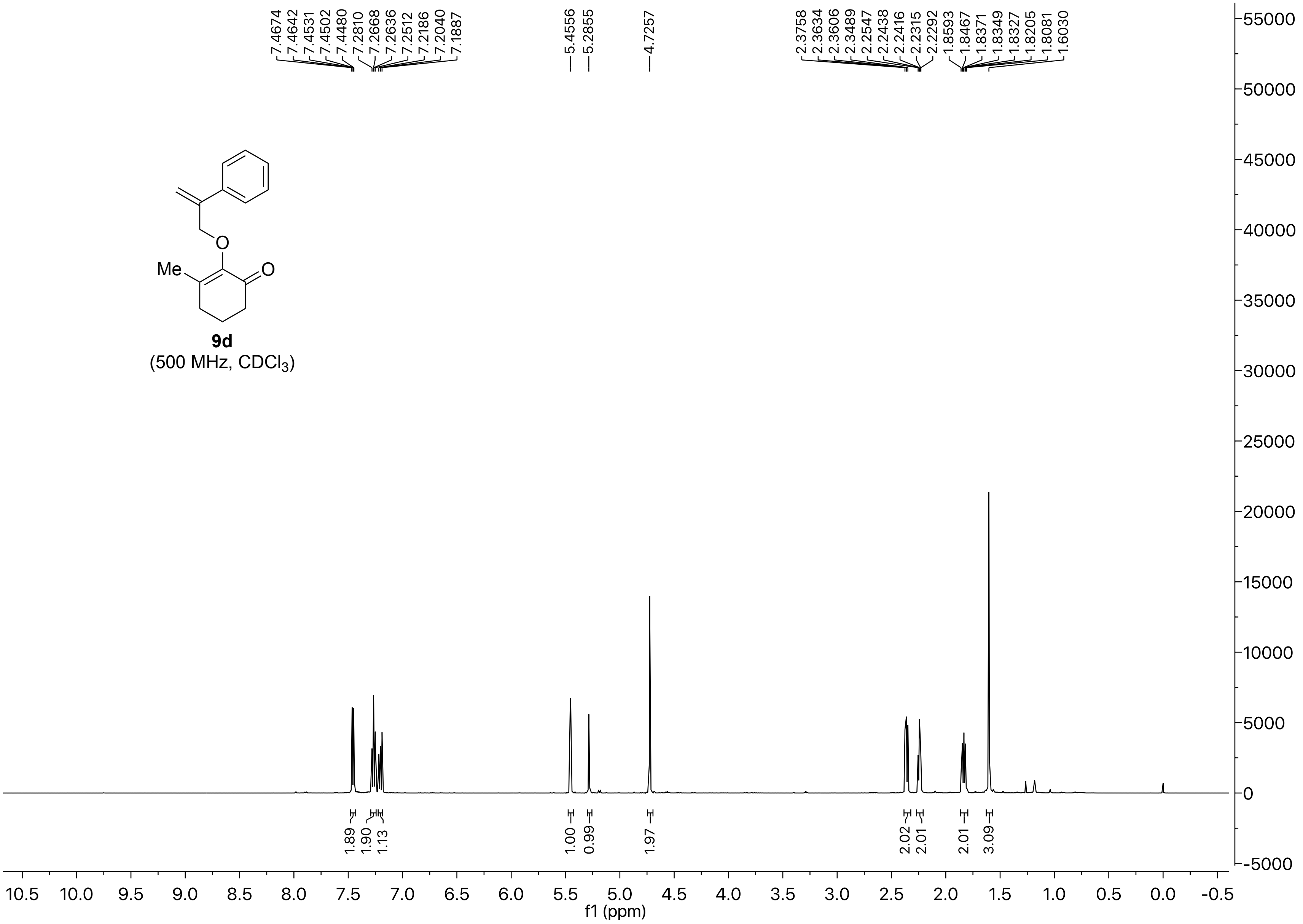
7.4674
7.4642
7.4531
7.4502
7.4480
7.2810
7.2668
7.2636
7.2512
7.2186
7.2040
7.1887
5.4556
5.2855
4.7257
2.3758
2.3634
2.3606
2.3489
2.2547
2.2438
2.2416
2.2315
2.2292
1.8593
1.8467
1.8371
1.8349
1.8327
1.8205
1.8081
1.6030
Me
O
O
9d
(500 MHz, CDCl3)
1.89
1.90
1.13
1.00
0.99
1.97
2.02
2.01
2.01
3.09
10.5 10.0 9.5 9.0 8.5 8.0 7.5 7.0 6.5 6.0 5.5 5.0 4.5 4.0 3.5 3.0 2.5 2.0 1.5 1.0 0.5 0.0 -0.5
f1 (ppm)
55000
50000
45000
40000
35000
30000
25000
20000
15000
10000
5000
0
-5000

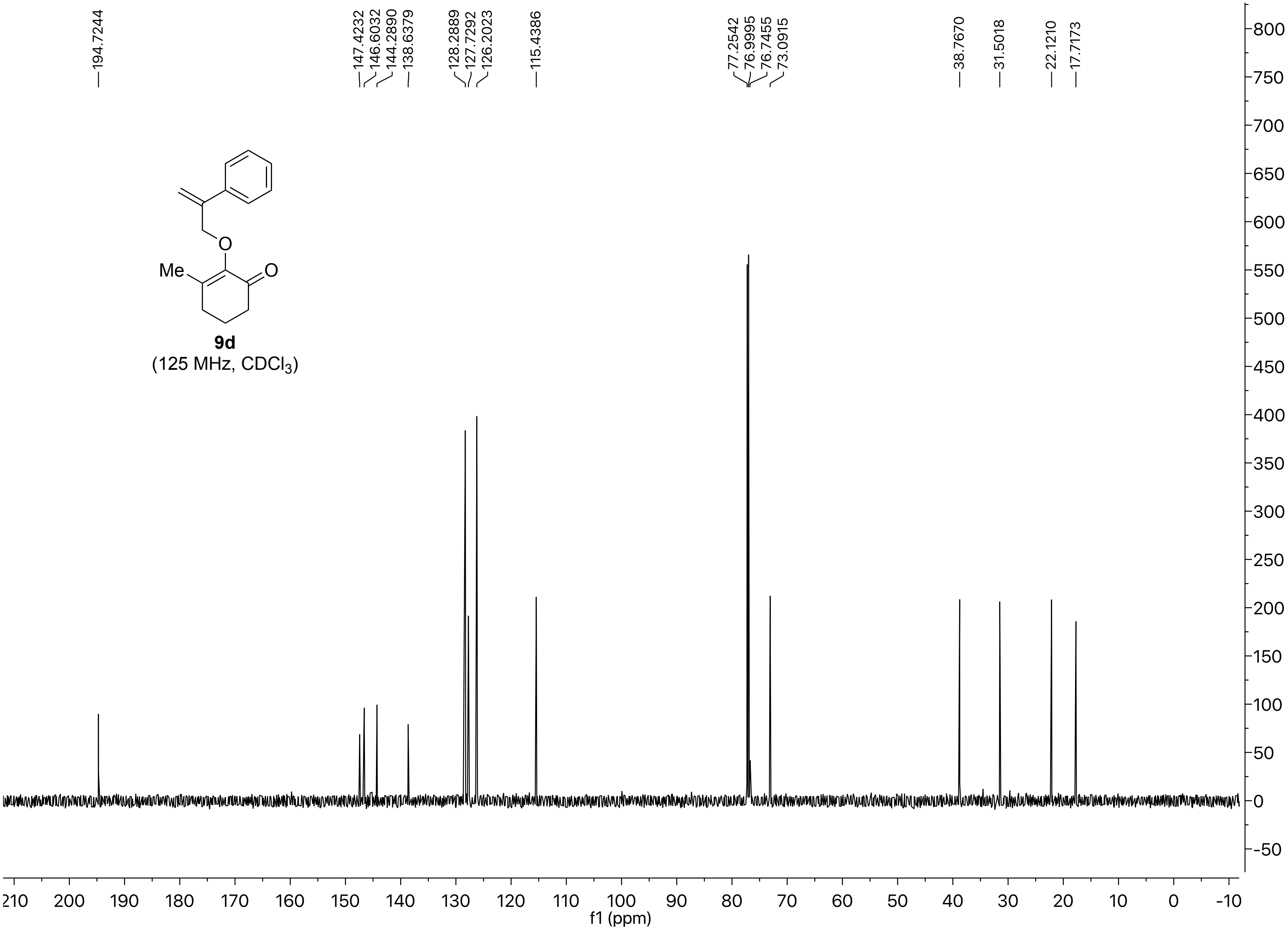
194.7244
147.4232
146.6032
144.2890
138.6379
128.2889
127.7292
126.2023
115.4386
77.2542
76.9995
76.7455
73.0915
38.7670
31.5018
22.1210
17.7173
Me
O
O
9d
(125 MHz, CDCl3)
f1 (ppm)

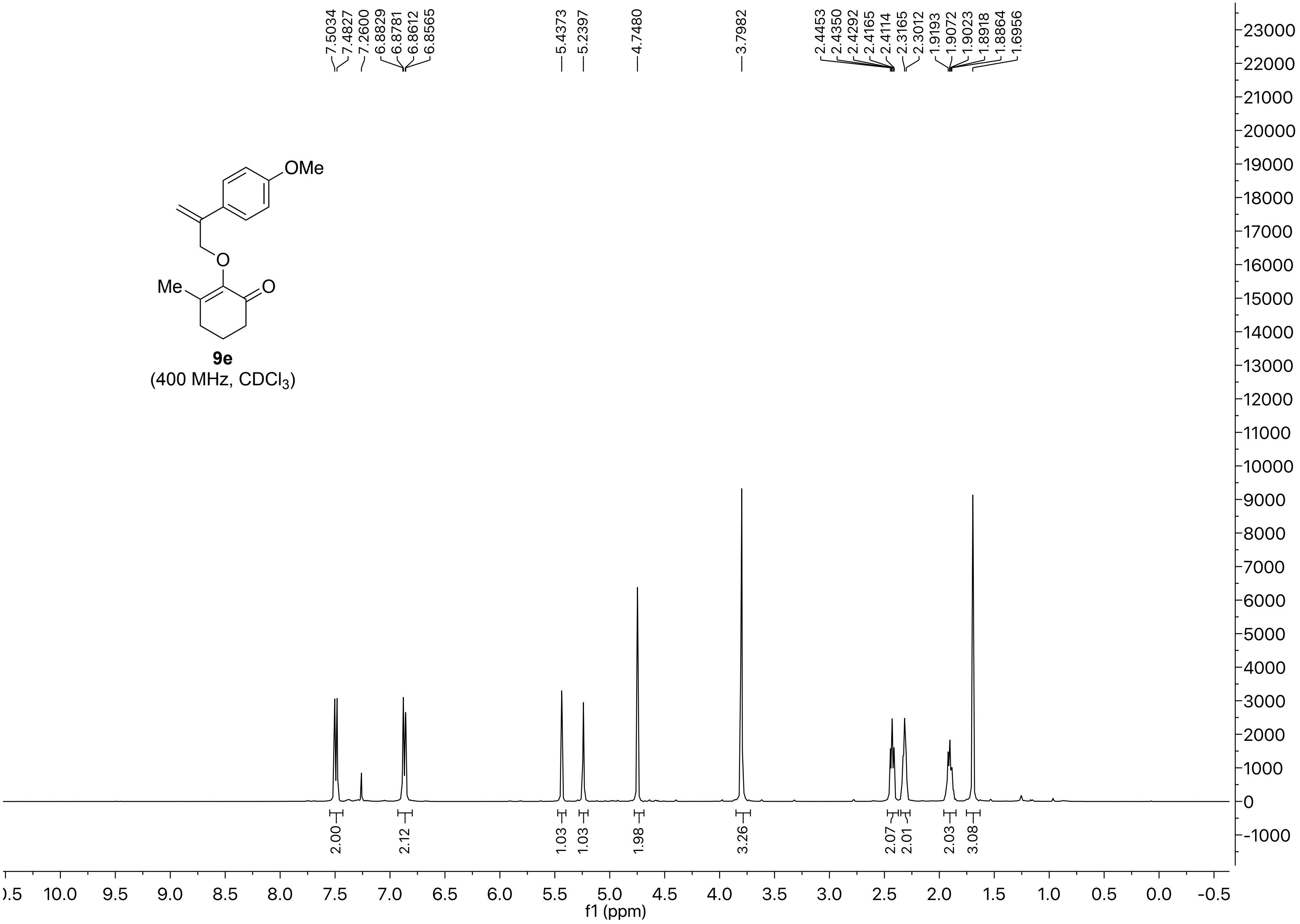

9e
(400 MHz, $CDCl_3$)

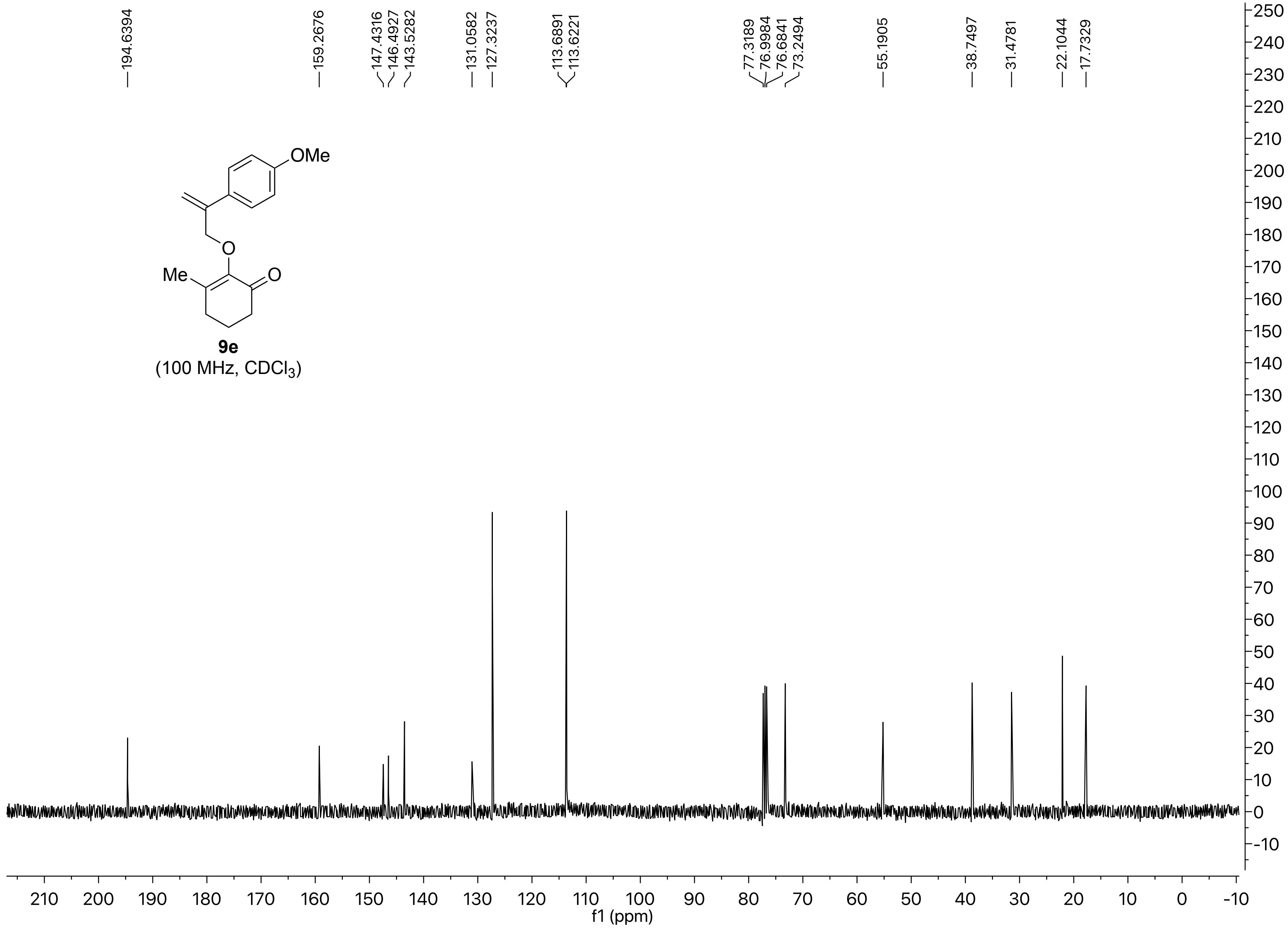

194.6394
159.2676
147.4316
146.4927
143.5282
131.0582
127.3237
113.6891
113.6221
77.3189
76.9984
76.6841
73.2494
55.1905
38.7497
31.4781
22.1044
17.7329
OMe
O
Me
O
9e
(100 MHz, CDCl3)
f1 (ppm)

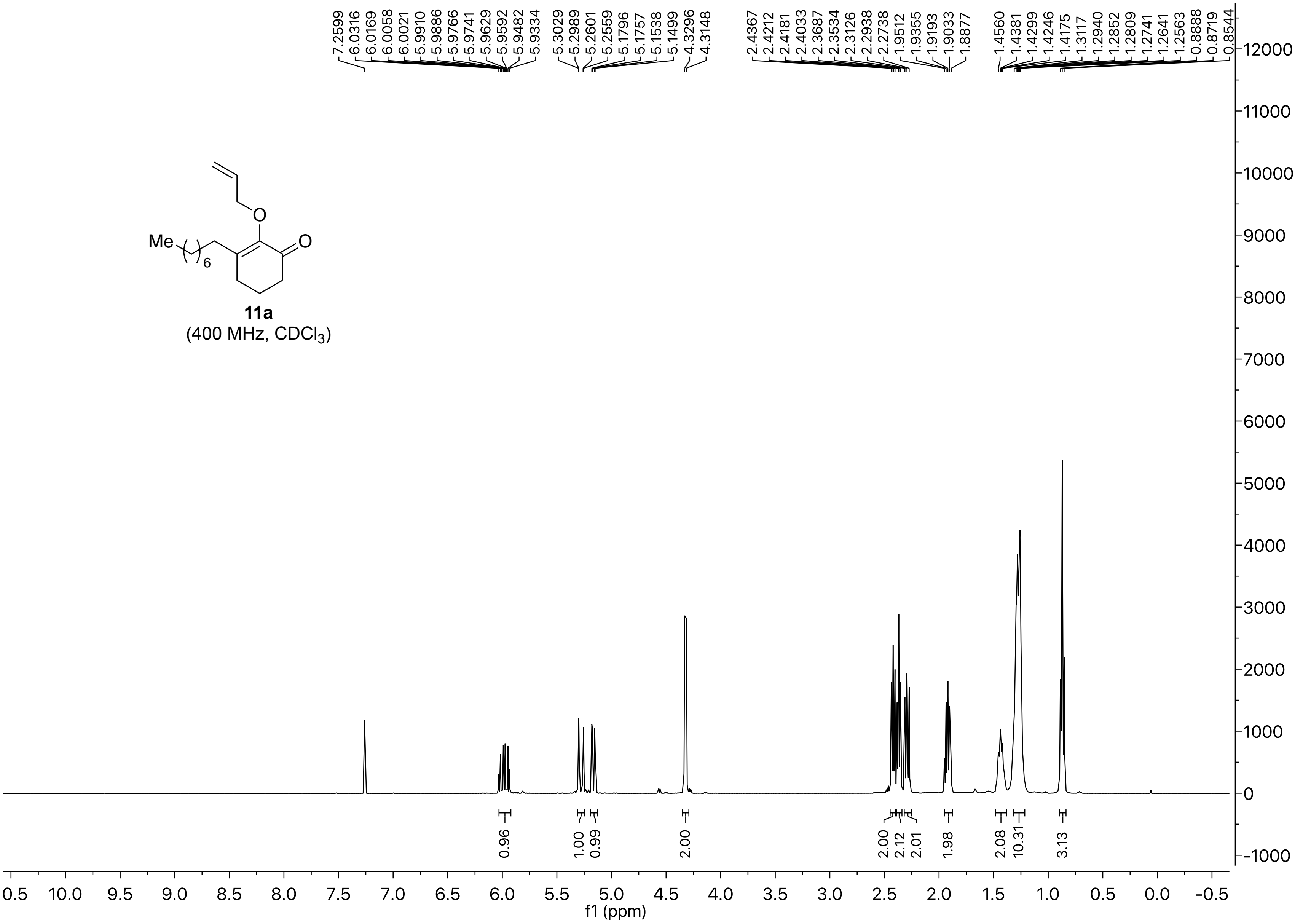
Me
6
O
O
11a
(400 MHz, CDCl3)
f1 (ppm)

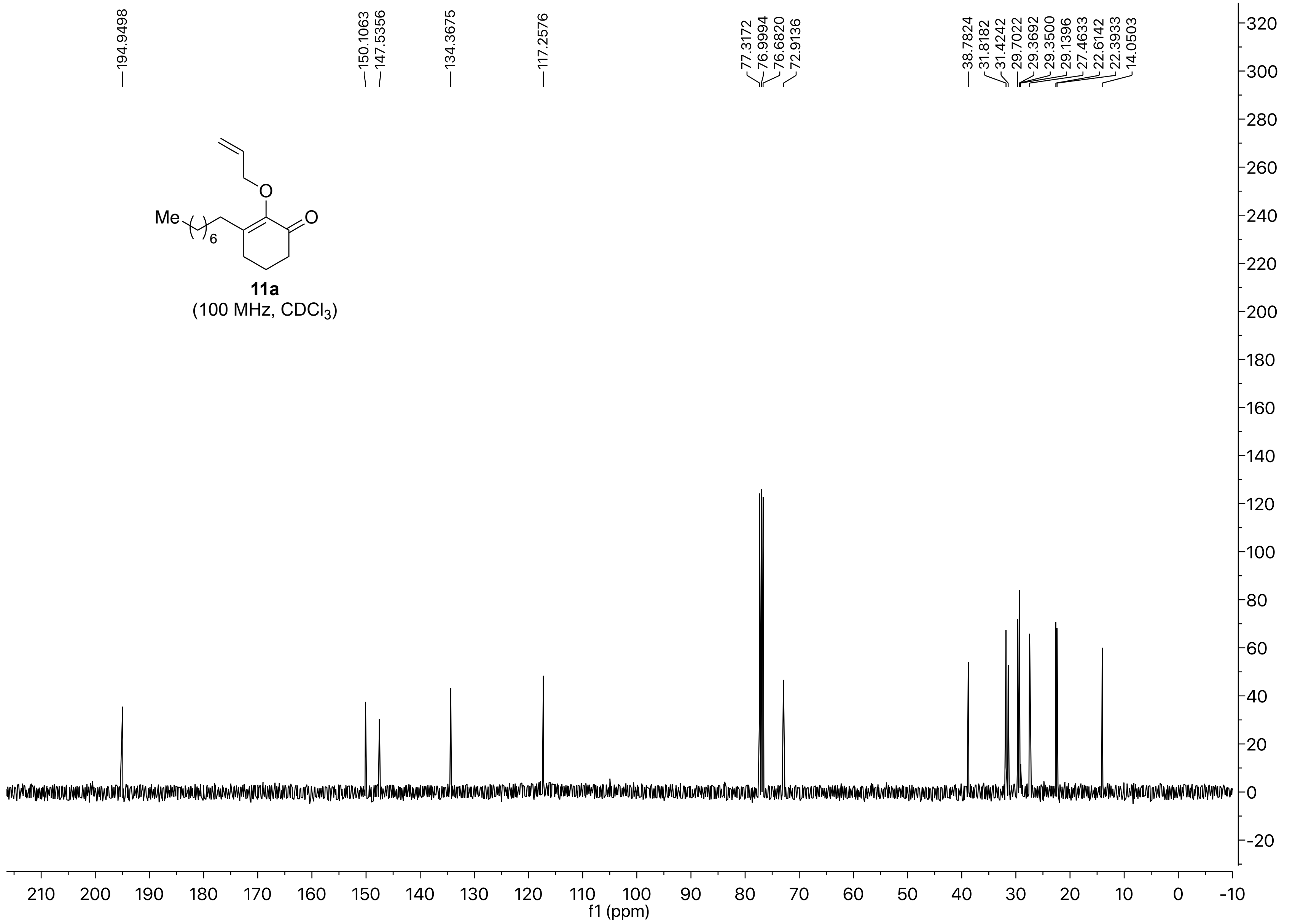

194.9498
150.1063
147.5356
134.3675
117.2576
77.3172
76.9994
76.6820
72.9136
38.7824
31.8182
31.4242
29.7022
29.3692
29.3500
29.1396
27.4633
22.6142
22.3933
14.0503
Me
6
O
O
11a
(100 MHz, CDCl3)
f1 (ppm)

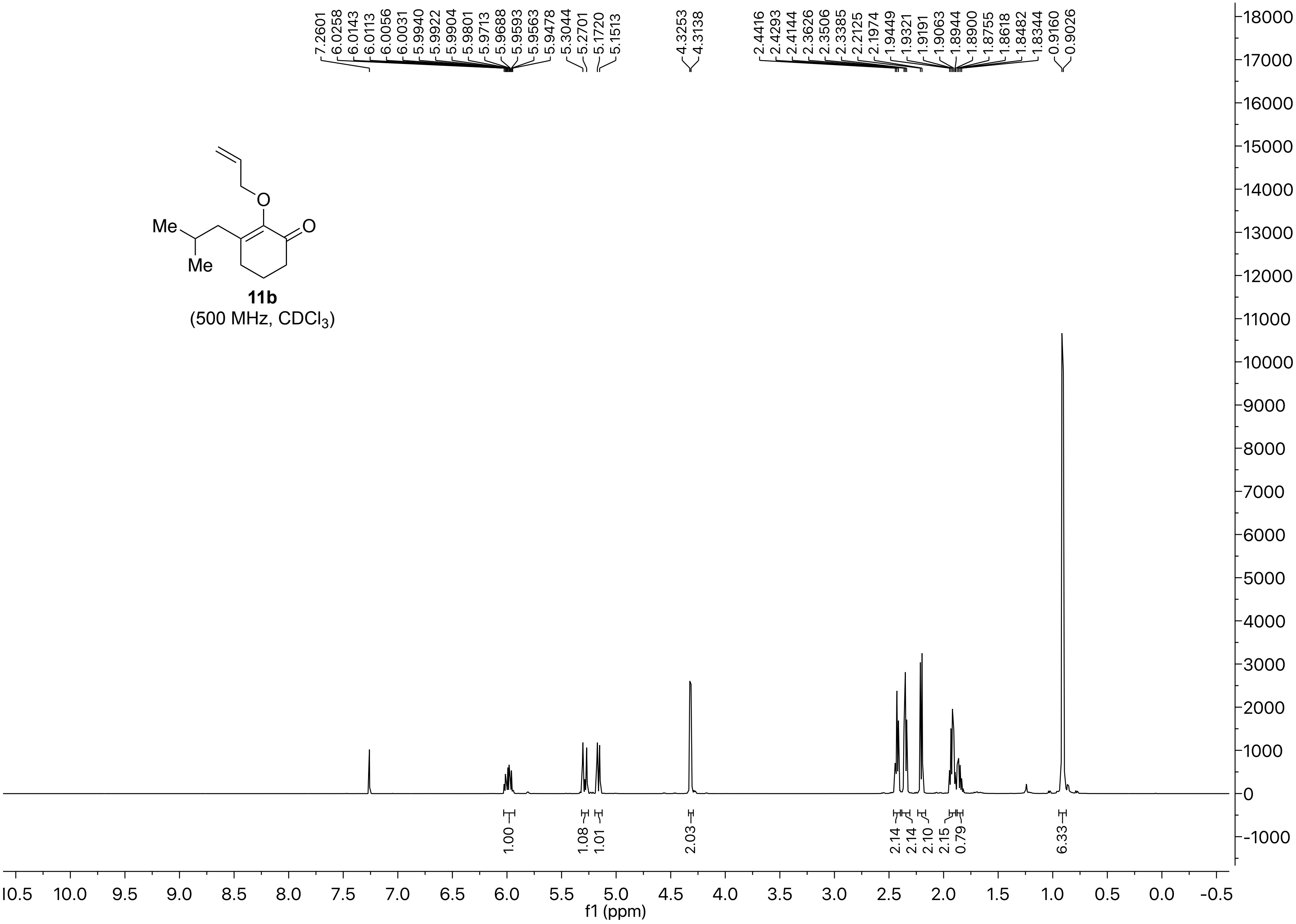
11b
(500 MHz, CDCl3)
f1 (ppm)

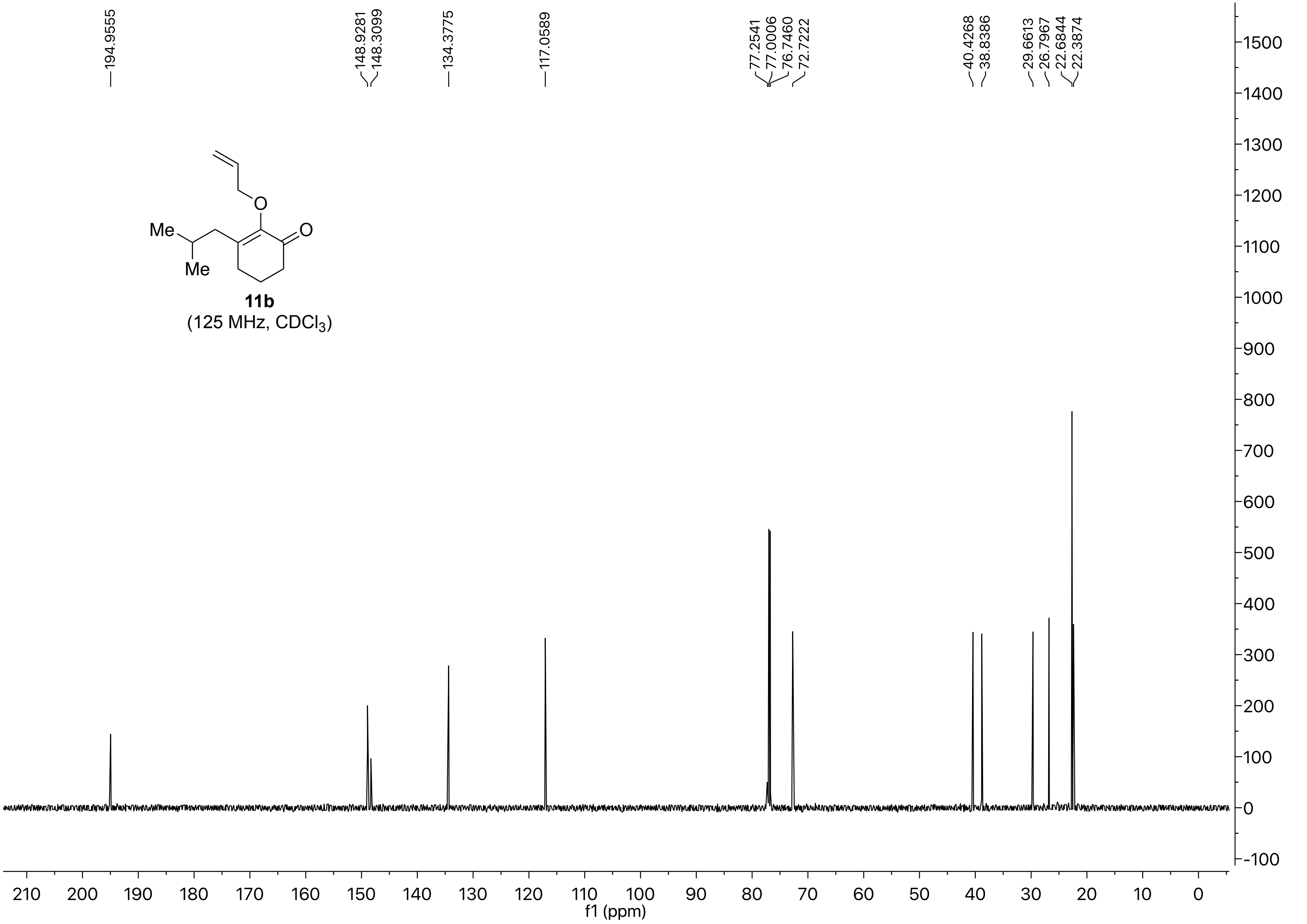
194.9555
148.9281
148.3099
134.3775
117.0589
77.2541
77.0006
76.7460
72.7222
40.4268
38.8386
29.6613
26.7967
22.6844
22.3874
Me
Me
O
O
11b
(125 MHz, CDCl3)
f1 (ppm)

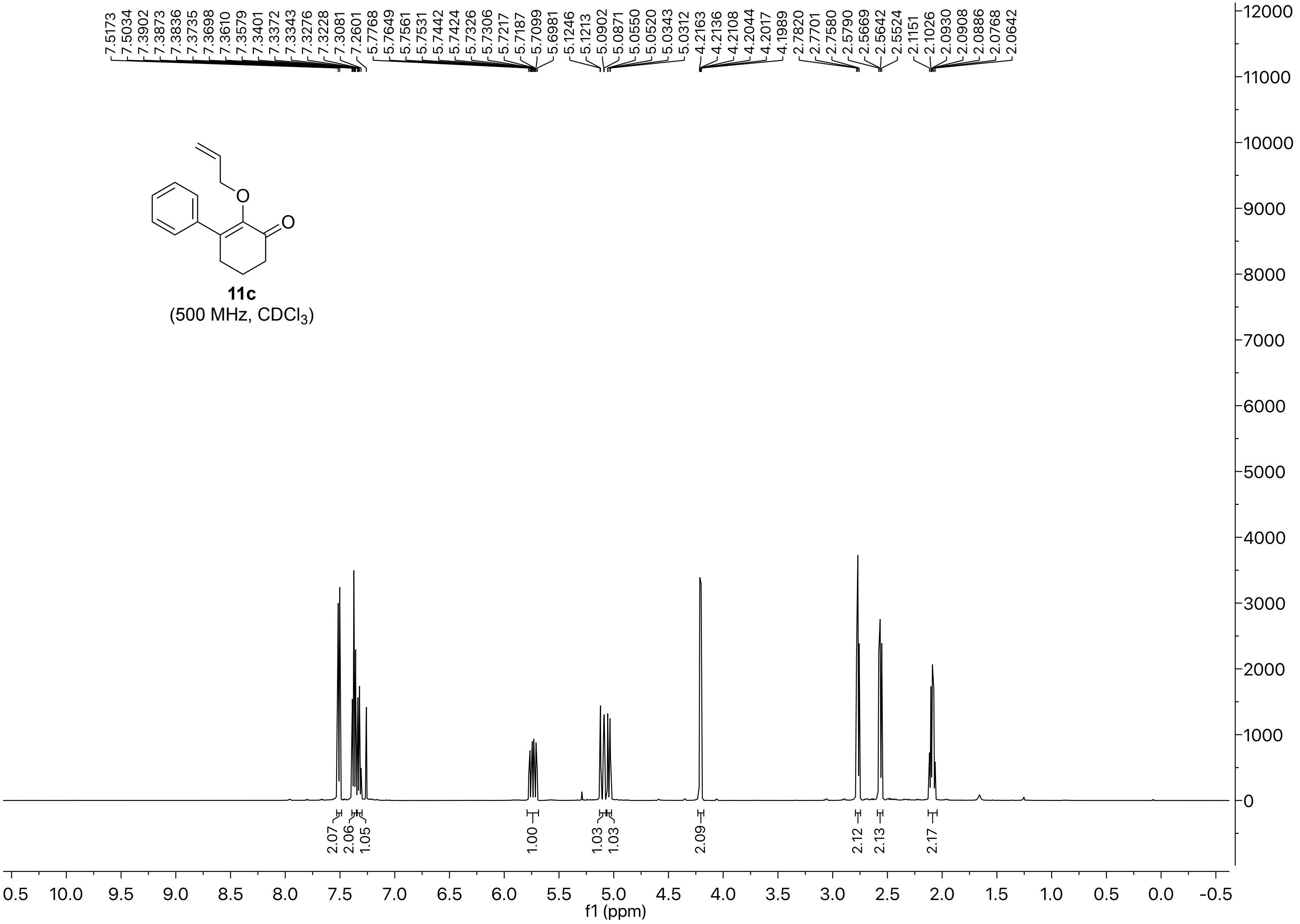
7.5173
7.5034
7.3902
7.3873
7.3836
7.3735
7.3698
7.3610
7.3579
7.3401
7.3372
7.3343
7.3276
7.3228
7.3081
7.2601
5.7768
5.7649
5.7561
5.7531
5.7442
5.7424
5.7326
5.7306
5.7217
5.7187
5.7099
5.6981
5.1246
5.1213
5.0902
5.0871
5.0550
5.0520
5.0343
5.0312
4.2163
4.2136
4.2108
4.2044
4.2017
4.1989
2.7820
2.7701
2.7580
2.5790
2.5669
2.5642
2.5524
2.1151
2.1026
2.0930
2.0908
2.0886
2.0768
2.0642
O
O
11c
(500 MHz, CDCl3)
2.07
2.06
1.05
1.00
1.03
1.03
2.09
2.12
2.13
2.17
f1 (ppm)

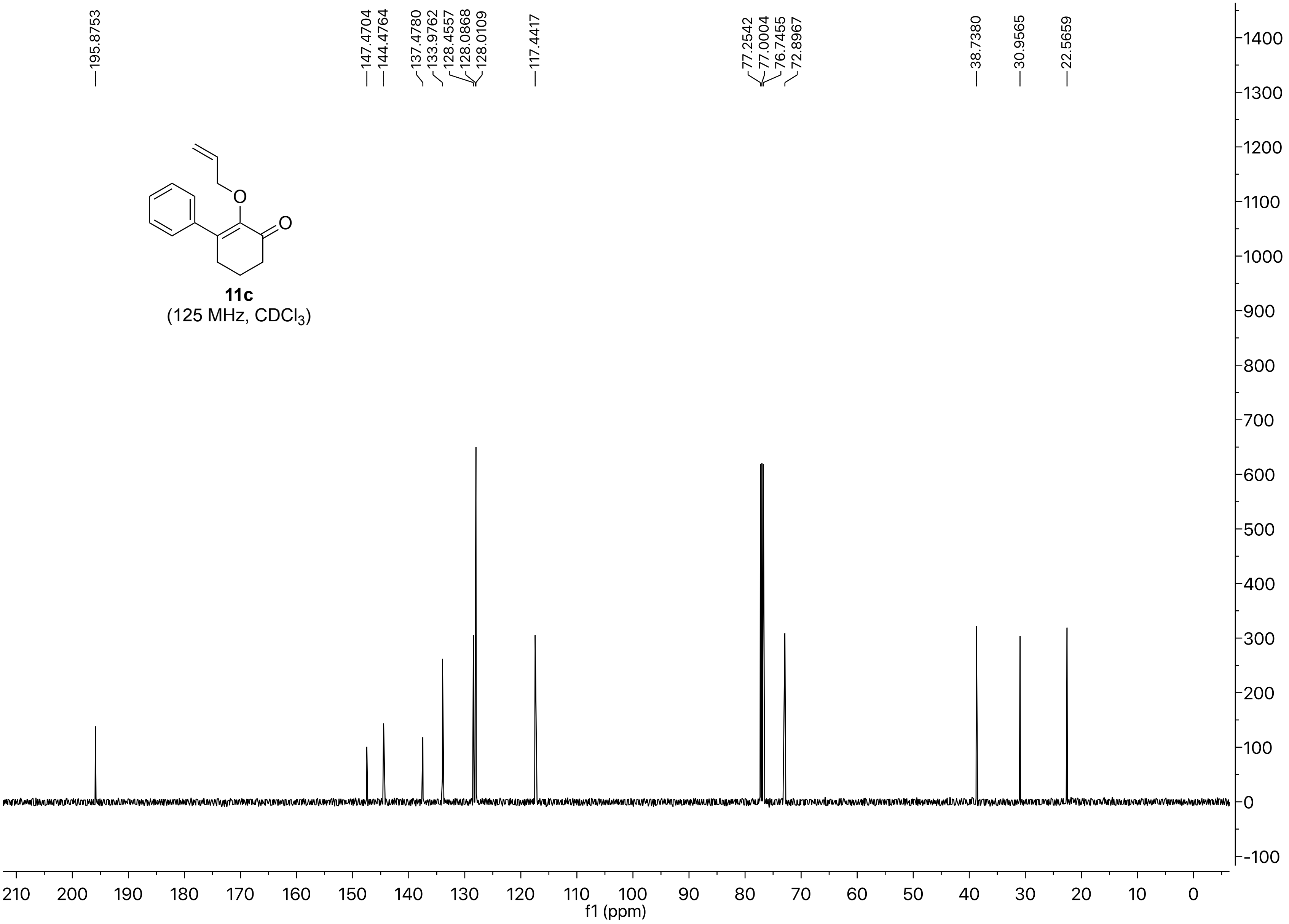
195.8753
147.4704
144.4764
137.4780
133.9762
128.4557
128.0868
128.0109
117.4417
77.2542
77.0004
76.7455
72.8967
38.7380
30.9565
22.5659
O
O
11c
(125 MHz, CDCl3)
1400
1300
1200
1100
1000
900
800
700
600
500
400
300
200
100
0
-100
210 200 190 180 170 160 150 140 130 120 110 100 90 80 70 60 50 40 30 20 10 0
f1 (ppm)

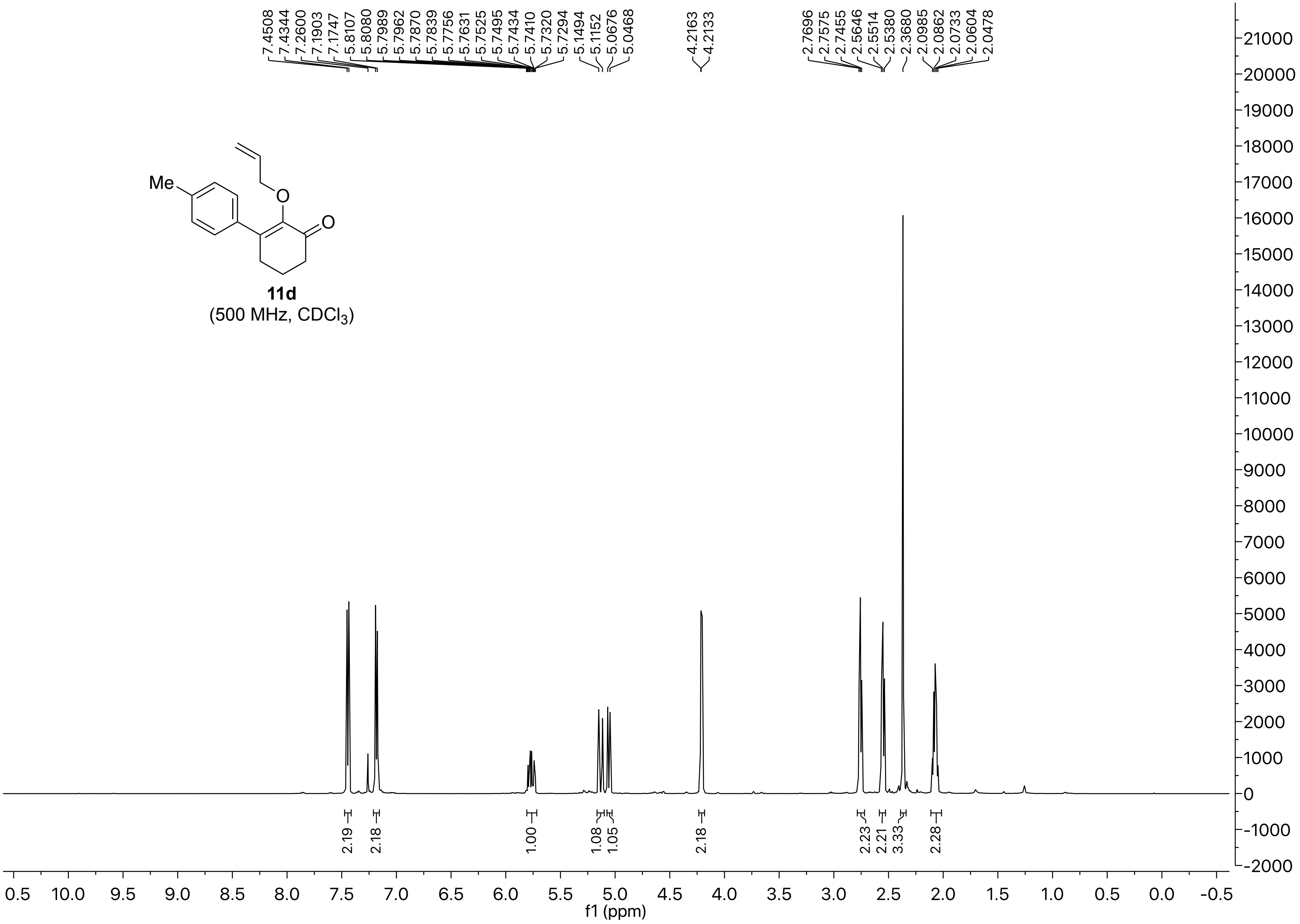
7.4508
7.4344
7.2600
7.1903
7.1747
5.8107
5.8080
5.7989
5.7962
5.7870
5.7839
5.7756
5.7631
5.7525
5.7495
5.7434
5.7410
5.7320
5.7294
5.1494
5.1152
5.0676
5.0468
4.2163
4.2133
2.7696
2.7575
2.7455
2.5646
2.5514
2.5380
2.3680
2.0985
2.0862
2.0733
2.0604
2.0478
Me
O
O
11d
(500 MHz, $CDCl_3$)
2.19
2.18
1.00
1.08
1.05
2.18
2.23
2.21
3.33
2.28
f1 (ppm)

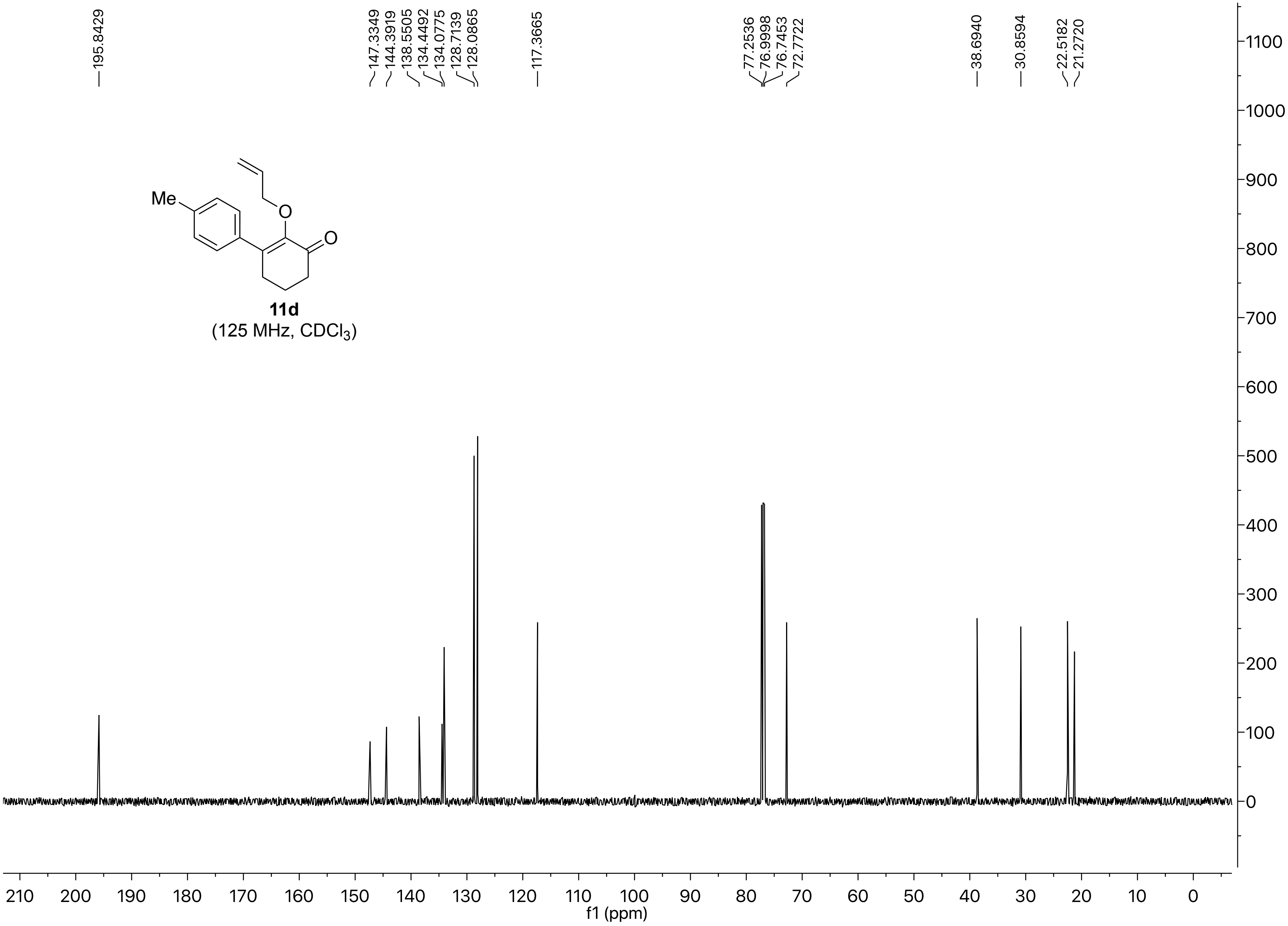
195.8429
147.3349
144.3919
138.5505
134.4492
134.0775
128.7139
128.0865
117.3665
77.2536
76.9998
76.7453
72.7722
38.6940
30.8594
22.5182
21.2720
Me
O
O
11d
(125 MHz, CDCl3)
f1 (ppm)

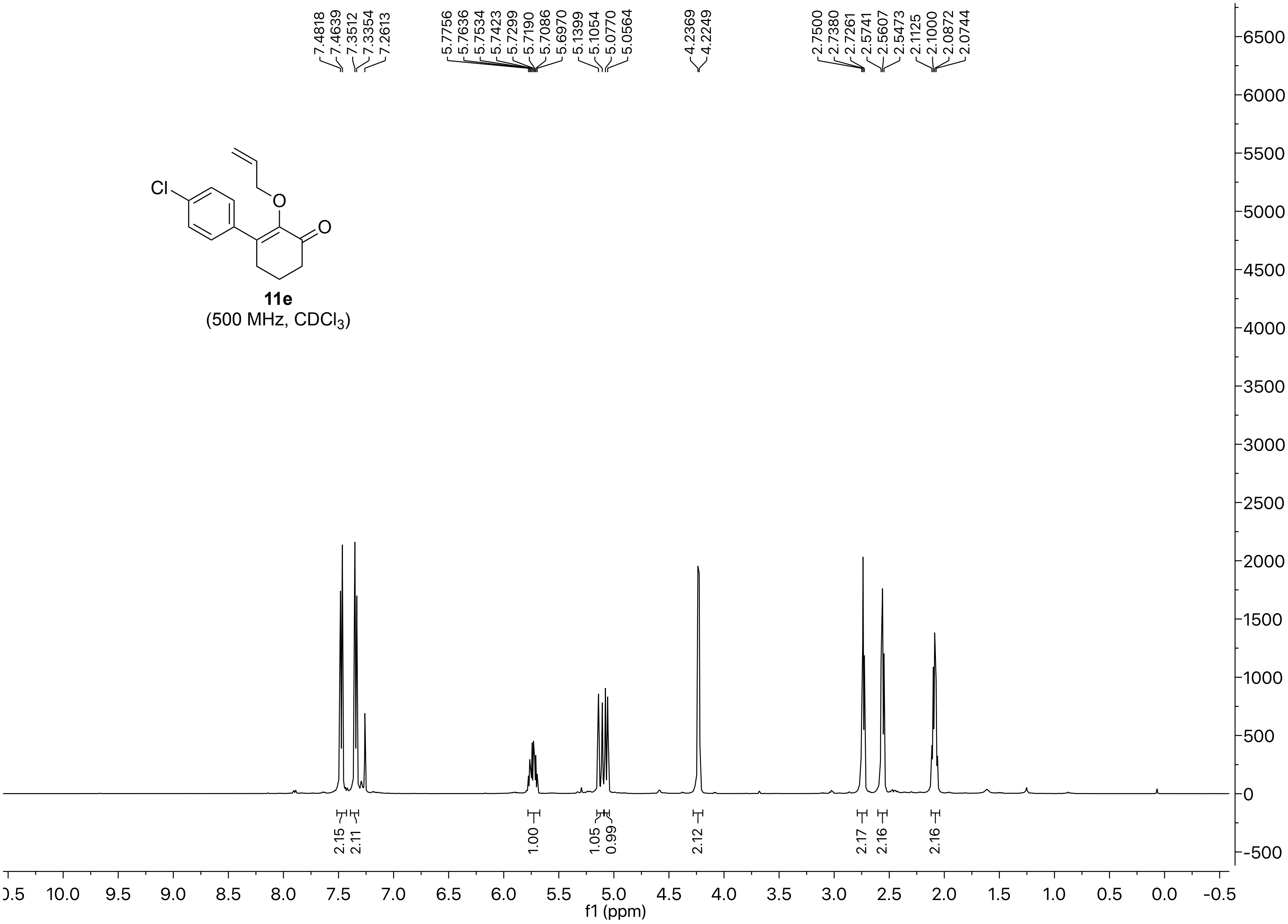

11e
(500 MHz, $CDCl_3$)

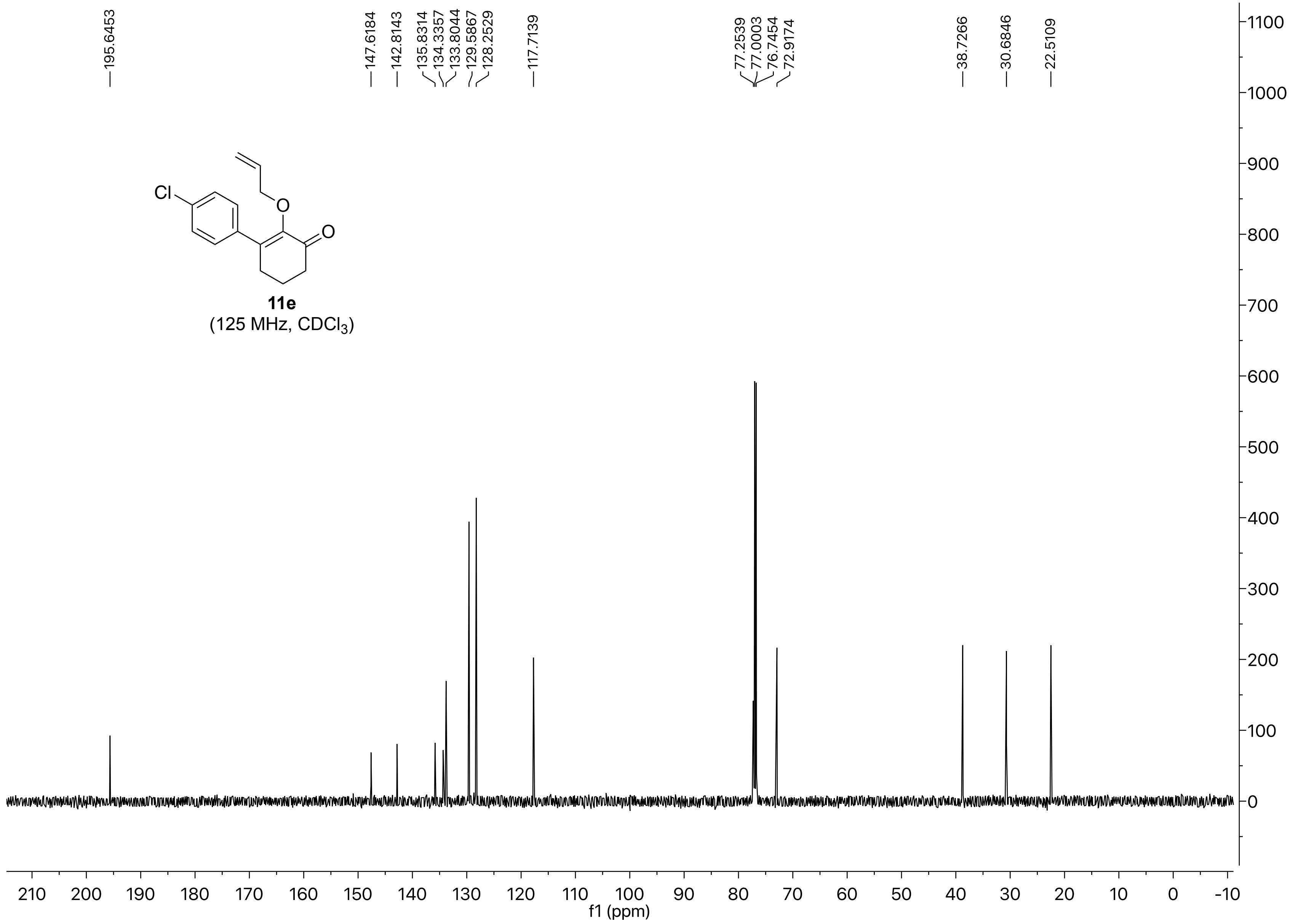

195.6453
147.6184
142.8143
135.8314
134.3357
133.8044
129.5867
128.2529
117.7139
77.2539
77.0003
76.7454
72.9174
38.7266
30.6846
22.5109
Cl
O
O
11e
(125 MHz, CDCl3)
f1 (ppm)

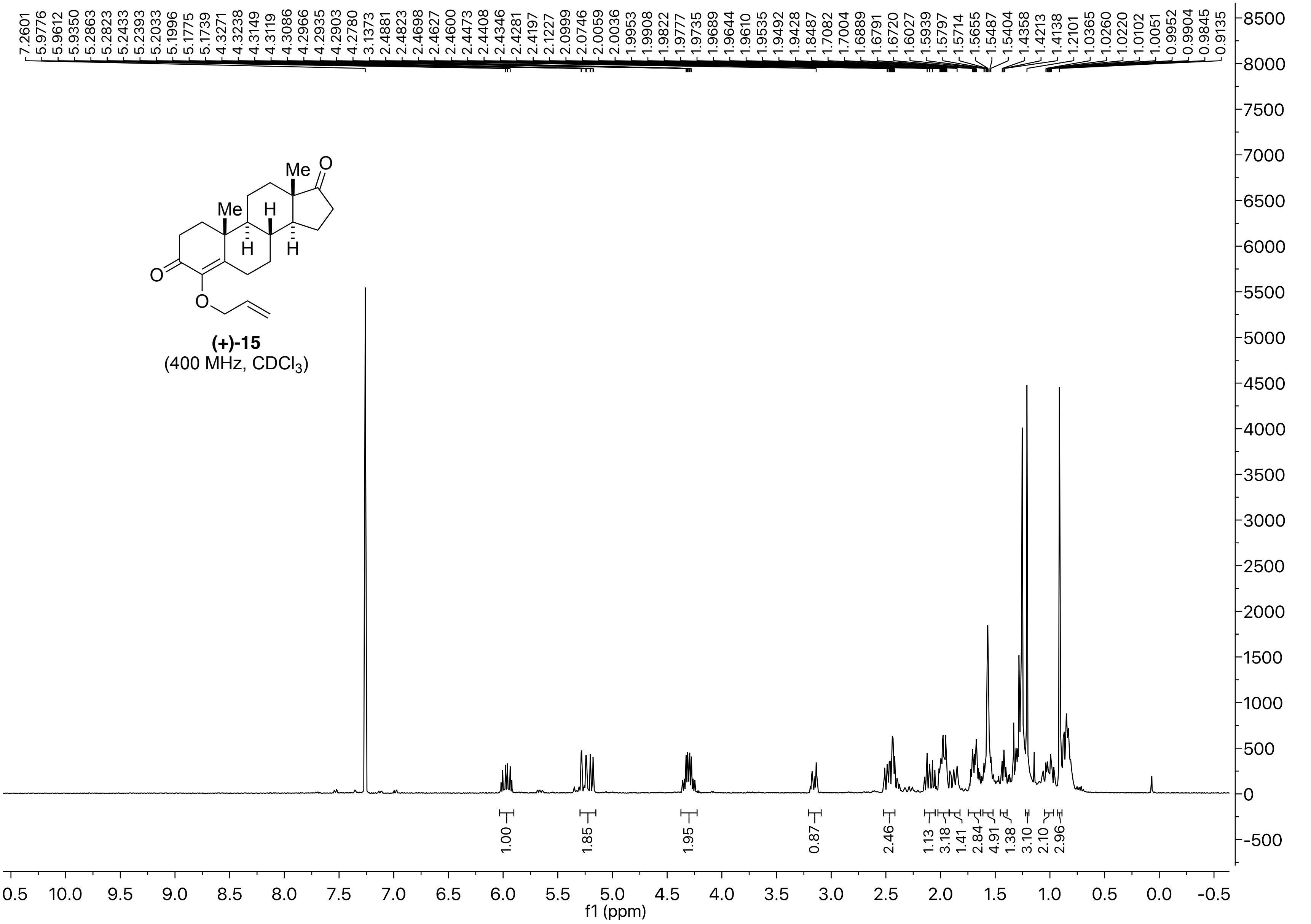

Me
O
Me
H
H
H
O
O
(+)-15
(400 MHz, CDCl3)
f1 (ppm)

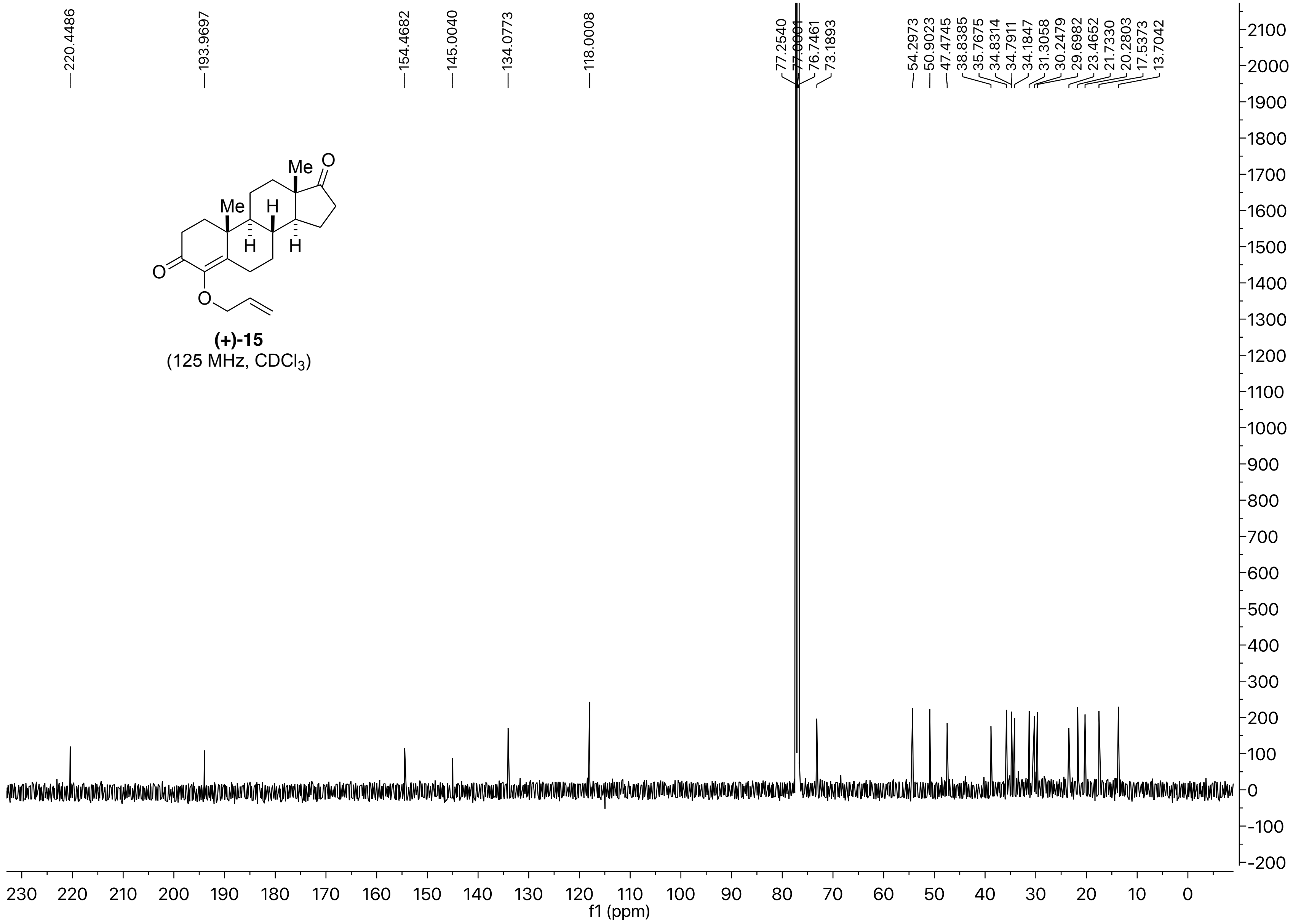

220.4486
193.9697
154.4682
145.0040
134.0773
118.0008
77.2540
77.0001
76.7461
73.1893
54.2973
50.9023
47.4745
38.8385
35.7675
34.8314
34.7911
34.1847
31.3058
30.2479
29.6982
23.4652
21.7330
20.2803
17.5373
13.7042
Me
O
Me
H
H
H
O
O
(+)-15
(125 MHz, $CDCl_3$)
f1 (ppm)
